# Exclusive Solvent-Controlled Regioselective Catalytic Synthesis of Potentially Bioactive Imidazolidineiminodithiones: NMR Analysis, Computational Studies and X-ray Crystal Structures

**DOI:** 10.3390/molecules29163958

**Published:** 2024-08-22

**Authors:** Ziad Moussa, Sara Saada, Alejandro Perez Paz, Ahmed Alzamly, Zaher M. A. Judeh, Aaesha R. Alshehhi, Aisha Khudhair, Salama A. Almheiri, Harbi Tomah Al-Masri, Saleh A. Ahmed

**Affiliations:** 1 Department of Chemistry, College of Science, United Arab Emirates University, Al Ain P.O. Box 15551, United Arab Emirates202102195@uaeu.ac.ae (A.R.A.); 202050063@uaeu.ac.ae (A.K.);; 2 School of Chemical and Biomedical Engineering, Nanyang Technological University, 62 Nanyang Drive, N1.2-B1-14, Singapore 637459, Singapore; 3 Department of Chemistry, Faculty of Sciences, Al al-Bayt University, P.O. Box 130040, Mafraq 25113, Jordan; 4 Department of Chemistry, Faculty of Applied Sciences, Umm Al-Qura University, Makkah 21955, Saudi Arabia

**Keywords:** *N*-arylcyanothioformamides, isothiocyanates, imidazolidineiminodithiones, density functional theory, thiazolidinethiones

## Abstract

Herein, we describe the first consistent regiospecific reaction of isothiocyanates with a variety of substituted *N*-arylcyanothioformamides in a 1:1 molar ratio to generate a series of imidazolidineiminodithiones decorated with a multitude of functional groups on both aromatic rings. The reaction is carried out at room temperature using a 20 mol% catalytic amount of triethylamine with DMF as the solvent to selectively form the mentioned products with exclusive regioselectivity. The methodology features wide substrate scope, no requirement for chromatography, and good to high reaction yields. The products were isolated by simple ether/brine extraction and the structures were verified by multinuclear NMR spectroscopy and high accuracy mass measurements. The first conclusive molecular structure elucidation of the observed regioisomer was established by single-crystal X-ray diffraction analysis. Likewise, the tautomer of the *N*-arylcyanothioformamide reactant was proven by X-ray diffraction analysis. Density functional theory computations at the B3LYP-D4/def2-TZVP level in implicit DMF solvent were conducted to support the noted regiochemical outcome and proposed mechanism.

## 1. Introduction

*N*-arylcyanothioformamides are a class of organic compounds with the chemical formula RNHC(S)CN, where R is an aryl group [1,2]. These compounds contain both an electrophilic cyano group (-CN) and a nucleophilic thioamide group (-C(S)NH-) in their molecular structure. As such, and apart from being intrinsically bioactive as herbicides [3] and gastropodicides [4], they have served as versatile reagents in a wide variety of transformations and “one-pot” heterocyclic annulation reactions to generate various types of useful intermediates and bioactive heterocycles [5,6,7,8,9,10,11,12,13,14,15,16,17,18,19,20,21]. One attractive feature associated with *N*-arylcyanothioformamides is the ease of their preparation by various methods [22,23,24,25,26,27,28,29,30,31,32,33,34,35,36]. 

**Scheme 1:**
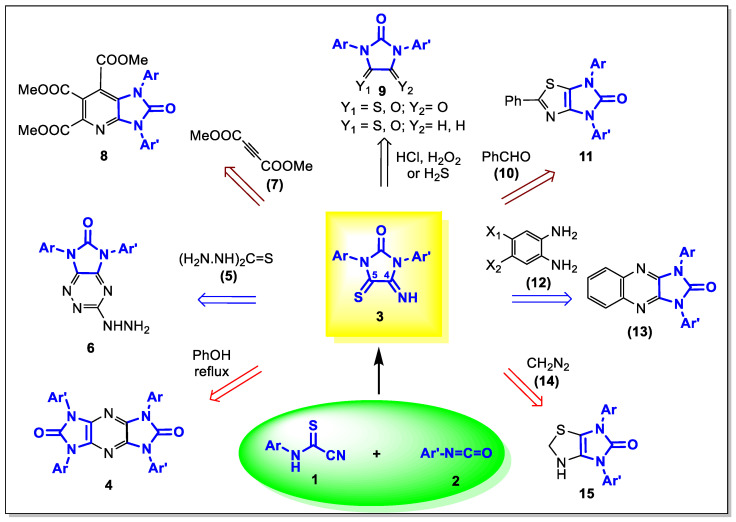
Previous preparation and some selected reactions of imidazolidineiminothiones **3**.

We and others have previously reported on the preparation, derivatization, and bioactivity of imidazolidineiminothiones **3** [7,10,18,20,37]. Apart from their therapeutic properties against tumor cell lines, they also demonstrate promising antiviral, antimicrobial, and antifungal activity. They are synthesized from *N*-arylcyanothioformamides **1** by ring closing reactions with various isocyanates **2** that yield heterocycles with adjacent imino and thione functions in positions 4 and 5, respectively (Scheme 1). These groups are suspected to be the pharmacophore responsible for such compounds’ wide range of biological effects. This arrangement effectively serves as a reactive template in subsequent ring-closing reactions during which one or both of the exocyclic functional groups have been incorporated into further fused heterocyclic rings, producing heterocycles such as **4**, **6**, **8**, **9**, **11**, **13**, and **15** from various reagents like phenol, thiocarbohydrazide (**5**), DMAD (**7**), PhCHO (**10**), ODP (**12**), and diazomethane (**14**), among others, as shown in Scheme 1. 

**Scheme 2:**
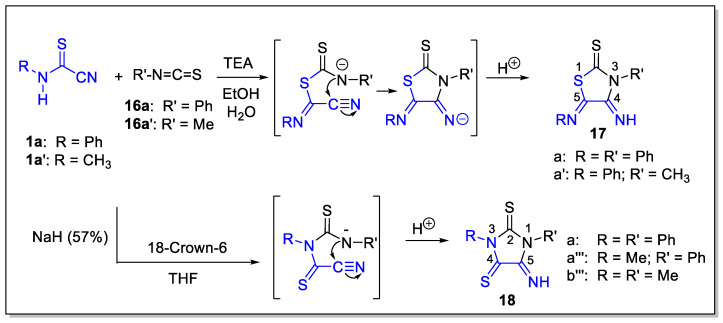
Ketcham’s ring-closing reactions of *N*-phenylcyanothioformamide (**1a**) and *N*-methyl-(**1a′**) with aryl (**16a**) and alkyl (**16a′**) under various conditions.

Recognizing the usefulness of **3** as bioactive species that could be further used to construct various heterocyclic rings (Scheme 1), the related imidazolidineiminodithiones emerged as interesting targets that could potentially be prepared from **1** and isothiocyanates **16**. Indeed, such reactions were first investigated by Ketcham et al., although, surprisingly, **1** did not react exclusively through the nitrogen atom in an analogous manner to their reaction with isocyanates (Scheme 2) [38,39]. This revelation initiated a more thorough investigation of the annulation reactions of both phenyl **16a** and methyl isothiocyanates (**16a′**) with *N*-phenyl-(**1a**) and *N*-methylcyanothioformamides (**1a′**) under various conditions. Astonishingly, the reactions of both phenyl isothiocyanates (**16a**) and methyl (**16a′**) with either *N*-phenylcyanothioformamide (**1a**) or *N*-methyl-(**1a′**) gave exclusively the 5-(phenylimino)-4-imino-2-thiazolidinethiones **17** (Scheme 2). This product is generated from nucleophilic attack by the sulfur atom of the ambident anion generated from the deprotonation of **1** with triethylamine (TEA). 

Under the aforementioned conditions, the polar protic ethanol-water mixture possibly solvates the nucleophilic nitrogen anion of the cyanothioformanilide **1**, promoting the larger sulfur anion to emerge as the more effective nucleophilic species because of hydrogen bonding of the solvent with the nitrogen center. Therefore, it was intuitive to investigate conditions that promote the formation of the free, uncoordinated nucleophile from the cyanothioformamide **1** in an aprotic environment, where the more basic nitrogen center would behave as the better nucleophile. Thus, when catalytic amounts of NaH and 18-crown-6 ether in THF were employed, the imino-l,3-diphenyl-2,4-imidazolidinedithione **18a** formed as the only product from the reaction of **1a** with **16a**. Unfortunately, while in this case it was possible to direct the reactivity of the ambident nucleophile **1**, the ability to selectively control the reaction site (*N* vs. *S*) was lost when changing the substitution pattern on either the isothiocyanate **16** and/or the cyanothioformanilide **1**. Huang and Graves described the impact of temperature, base and the type and position of the substituent on the course of the reaction to produce either imidazolidineiminodithiones or thiazolidinethiones [40]. Although some imidazolidineiminodithiones could be exclusively generated by regulating the temperature, the overall application to the preparation of this regioisomeric species was restricted mainly due to the strong dependence on the location and type of the substituents on **1**. As such, the outcome of such a transformation would be an inseparable mixture of regioisomers (**17** and **18**) or uncontrolled formation of one of the two possible products (**17** or **18**).

Since 1987, there has been no attempts to develop reliable synthetic methods to prepare imidazolidineiminodithiones **18**. In view of the wide interest in the bioactivity profile of imidazolidineiminothiones [1,2] and due to the unavailability of reliable synthetic protocols to prepare the analogous dithiones **18**, we aimed to develop reliable synthetic conditions to prepare the latter species and prove the consistency of the protocol by preparing and characterizing a large number of variously substituted new, pure imidazolidineiminodithiones. Developing a reliable methodology for the synthesis of pure imidazolidineiminodithiones will offer the scientific community an opportunity, as never before, to access these compounds and explore their biological activities and chelation to metals, among other properties, and to investigate their derivatization chemistry. Heretofore, the number of reported dithiones **18** stands at 23 since their discovery. However, three compounds are mixtures of isomers and ten are not characterized and therefore cannot be confirmed. Thus, the number of pure dithiones stands at ten so far. The biological activity of such compounds has been hampered by their scant availability since such compounds almost always form as inseparable mixtures with regioisomer **17** due to the ambident nucleophilic character of the precursor *N*-arylcyanothioformamides that react via the nitrogen or sulfur atom. 

Over a decade ago, we observed an impressive bioactivity profile with three imidazolidineiminodithiones **18** [7] which comprised the major products in regioisomeric mixtures with **17**. Notably, the bioactivity observed with **18** against various microbes and cancer cell lines was much superior to anything seen with its oxygen analogues **3**. This provided the impetus to further explore the preparation of the former in pure form. 

Based on earlier observations, the substituents on the aryl isothiocyanate appear to have negligible effects on the reaction pathway (regioselectivity) and only impact the reaction rate [40]. On the contrary, the type and location of the functional group on **1** as well as the reaction temperature strongly influence the reaction pathway generating either **17** or **18**, or a mixture thereof. 

## 2. Results and Discussion

### 2.1. Chemistry

#### 2.1.1. Solvent Screening Study

Surprisingly, one critical parameter, the impact of solvent, was never considered by any of the researchers in their endeavors to control the formation of either regioisomer (**17** vs. **18**). Consequently, we became interested in re-examining the reaction between *N*-arylcyanothioformamides **1** and isothiocyanates **16** and optimizing the conditions to control the formation of **18**. Thus, using *N*-phenylcyanothioformamide (**1a**) and phenyl isothiocyanate (**16a**) as model unsubstituted reactants, the heterocyclization reaction was conducted in 15 different solvents at room temperature using 20 mol% of TEA as a catalyst (see Supporting Information Sections S321–S365). The reaction progress was monitored by ^1^HNMR spectroscopy, which indicated that 21–24 h was the ideal time for complete conversion in the screened solvents. Upon reaction completion, the regioisomeric product ratio of the crude residue was measured using ^1^HNMR by integrating the appropriate well-resolved signals for each regioisomer and taking the mean value for most accurate measurements. As a representative signal to compare the product distribution of the imidazolidineiminodithione **18a** versus the thiazolidinethione product **17a**, the broad N-H resonance, which absorbs furthest downfield in the proton NMR, has been used to illustrate the observed results with various solvents. 

**Figure 1:**
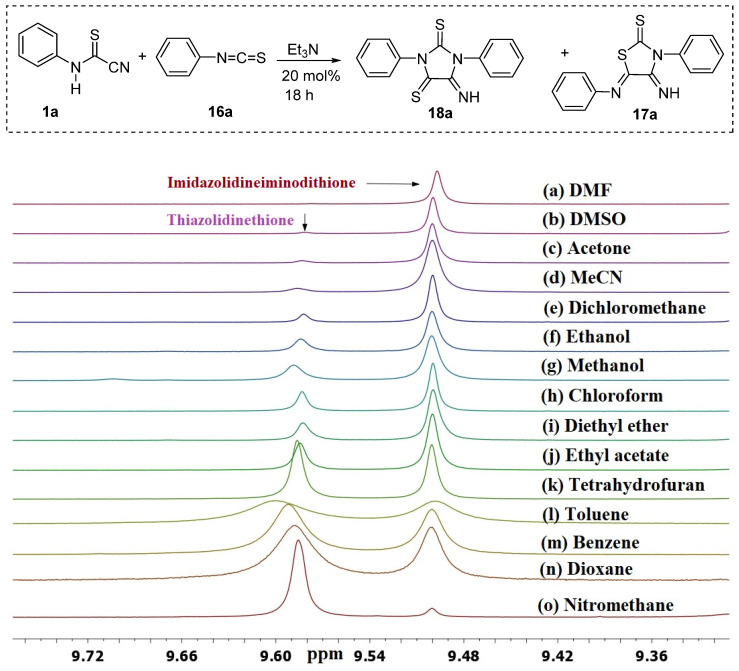
Expanded and truncated ^1^HNMR spectra showing the N-H protons as representative signals to demonstrate product distribution of the imidazolidineiminodithione **18a** vs. the thiazolidinethione product **17a** using various solvents.

The results are illustrated in Figure 1, which shows expanded and truncated ^1^HNMR spectra displaying the N-H protons where the N-H of **17a** absorbs at δ 9.59 ppm and that of **18a** resonates slightly upfield at δ 9.50 ppm. It is quite clear that the formation of the two regioisomers with different solvents as shown in Figure 1 is not a reflection of kinetic vs. thermodynamic control. While many of the solvents like THF, toluene, benzene, and dioxane (Figure 1, spectra k–n), among others, produced mixtures of regioisomers, the overall trend favored the preferential formation of the **18a** with ethyl acetate, diethyl ether, chloroform, methanol, ethanol, dichloromethane, acetonitrile, acetone, DMSO, and DMF (Figure 1, spectra a–j). While traces of **17a** are detected in DMSO (Figure 1, spectrum b), exclusive formation of **18a** occurred in DMF (Figure 1, spectrum a). With THF, toluene, benzene, dioxane, and nitromethane (spectra k-o), the trend favored the formation of **17a** as the major isomer, with nitromethane giving the best ratio (90:10). The gradual shift toward the formation of the desired isomer **18a** with different solvents represents a significant breakthrough in controlling the selective production of this isomer. As the main focus of this study was the preparation of isomer **18**, any further investigations aimed at optimizing the formation of the kinetic isomer **17** in nitromethane were outside the scope of this work.

To further explore the impact of the solvent on the product distribution, we expanded the solvent screening study using two different pairs of reactants: *N*-(2-fluorophenyl)cyanothioformamide (**1b**) and phenyl isothiocyanate (**16a**), as well as *N*-phenylcyanothioformamide (**1a**) and 2-fluorophenyl isothiocyanate (**16b**). The objective was to investigate the consistency of the solvent control and test the impact of the same substituent (i.e., fluorine atom) on the course of the reaction. The summarized results of the product distribution **18/17** for the two pairs of reactants (**1a/16b** and **1b**/**16a**), as well as the first unsubstituted pair (**1a** and **16a**), are presented in Table 1. Clearly, the course of reaction leading to either **18** or **17**, or a mixture of both, is influenced to some extent by the substituent. In general, the formation of the thiazolidinethione isomer **17** was not controlled by any specific solvent. For example, 90% of this isomer was formed in nitromethane with the unsubstituted reactants (Entry 1), while 57% and 71% were formed with the fluorinated reactants (Entry 1). Moreover, none of the solvents afforded such an isomer in any ratio exceeding 71%. On the other hand, several solvents consistently produced the desired imidazolidineiminodithione **18** isomer exclusively or as the major product. In particular, acetonitrile afforded 90–100% (Entry 12) of this isomer, whereas the aprotic solvents, acetone, DMSO, and DMF (Entries 13–15), were the best. It is noteworthy that DMF exhibited the remarkable ability to override both the substituent control and kinetic/thermodynamic factors, resulting in the exclusive generation of the desired regioisomer in all cases. 

Our observations contradict earlier reports [40] and indicate that the substituent on the isothiocyanate can indeed influence regioselectivity in addition to reaction rate. For instance, when 4-nitrophenyl isothiocyanate (**16c**) was used as the reactant (see Table 1, Entries 5–8, 10, and 13–15), we observed exclusive formation of one isomer (**18**) in seven solvents. Interestingly, even in the presence of solvents such as ethanol (Entry 10) that produced a mixture (94:6), DMF still promoted the exclusive formation of one isomer.

#### 2.1.2. Catalyst mol% Screening Study

The impact of the type of base used in the reaction on the product distribution of the two isomers (**17** vs. **18**) was not investigated previously. However, since DMF has resolved the outstanding challenge related to the selective synthesis of **18**, screening different bases became unnecessary. However, exploring the use of the base in catalytic amounts was warranted to guarantee clean reactions. Thus, as shown in Table 2, the reaction of *N*-phenylcyanothioformamide (**1a**) with phenyl isothiocyanate (**16a**) in DMF was explored using various amounts (10–100 mol%) of TEA and the progress of the reaction was followed by TLC and ^1^HNMR spectroscopy. 

After stirring at room temperature for 7.5 h, ^1^HNMR analysis of aliquots taken from the reaction mixtures indicated that those containing >50 mol% TEA had completely converted to **18** (Entries 9–14). While the reaction with 50 mol% TEA generated the purest product, those with 60–100 mol% TEA afforded reaction mixtures with some minor unidentified side products possibly due to slow degradation of **18** caused by the excess TEA. Interestingly, TEA has been reported to isomerize the kinetic thiazolidinethione **17** to the thermodynamically controlled regioisomer **18** [40], though in our hands, we observed the formation of additional side products, suggesting that the use of excessive amounts of TEA should be avoided. Using 10 mol%, 20 mol%, 30 mol%, and 40 mol% TEA, respective conversions of 60%, 70%, 80%, and 90% were achieved after 7.5 h. After 24 h, complete conversion and a notably clean product was observed in all four cases. Since 10–50 mol% of TEA was the ideal range to produce products with no signs of any degradation, we opted to use 20 mol% TEA and DMF for all reactions as optimum conditions.

#### 2.1.3. Preparation of Reactants 

**Scheme 3:**
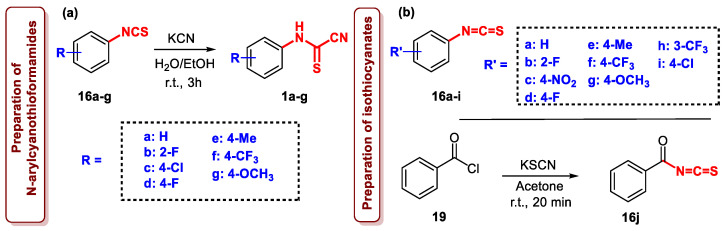
(**a**) Preparation of variously substituted *N*-arylcyanothioformamides **1a**–**g** and (**b**) commercial isothiocyanates **16a**–**i** coupling partners and preparation of isothiocyanate **16j**.

To establish the selectivity of the method and prove the dominant role of the solvent over the substituent effect when the optimized conditions were utilized, the same seven identical substituents (phenyl, 4-chlorophenyl, 2-fluorophenyl, 4-tolyl, 4-methoxy, 4-(trifluoromethyl)phenyl, and 4-fluorophenyl) were deliberately incorporated into both reactants **1** and **16**. Two other substituents (Bz, 4-NO_2_ and 3-CF_3_) were included in the isothiocyanate series **16**. Accordingly, a series of *N*-arylcyanothioformamides **1a**–**g** was synthesized according to our previously published protocol [7] (Scheme 3). The isothiocyanate coupling partners **16a**–**i** were acquired commercially, with the exception of **16j**, which was synthesized through the reaction of benzoyl chloride (**19**) and potassium thiocyanate in acetone (95% yield).

#### 2.1.4. Preparation of Imidazolidineiminodithiones **18a**–**z**, **18a′**–**z′**, and **18a″**–**18e″**

**Scheme 4:**
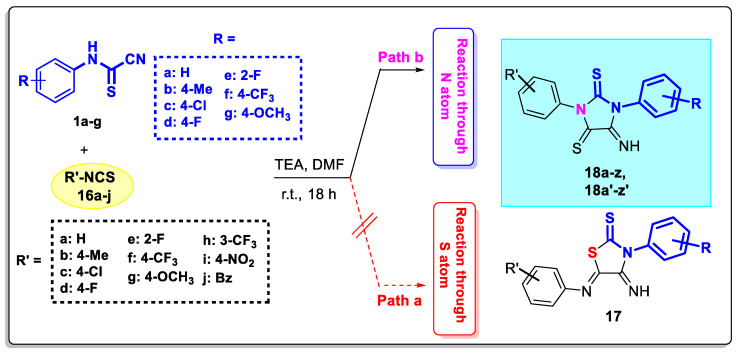
Synthetic approach to prepare different derivatives of variously substituted regioisomerically pure imidazolidineiminodithiones **18a**–**z**, **18a′**–**z′**, and **18a″**–**18e″**.

Next, we explored the generality of the optimized conditions, although with a shorter duration of time (1:1 equimolar amounts of **1**, **16**, and 20mol% TEA, DMF, rt, 24 h) (Scheme 4). While a reaction through the nitrogen atom of **1** produces regioisomer **17** through path “a”, alternatively, a reaction through path “b” would produce the other isomer **18** through the reaction of the sulfur atom.

**Figure 2:**
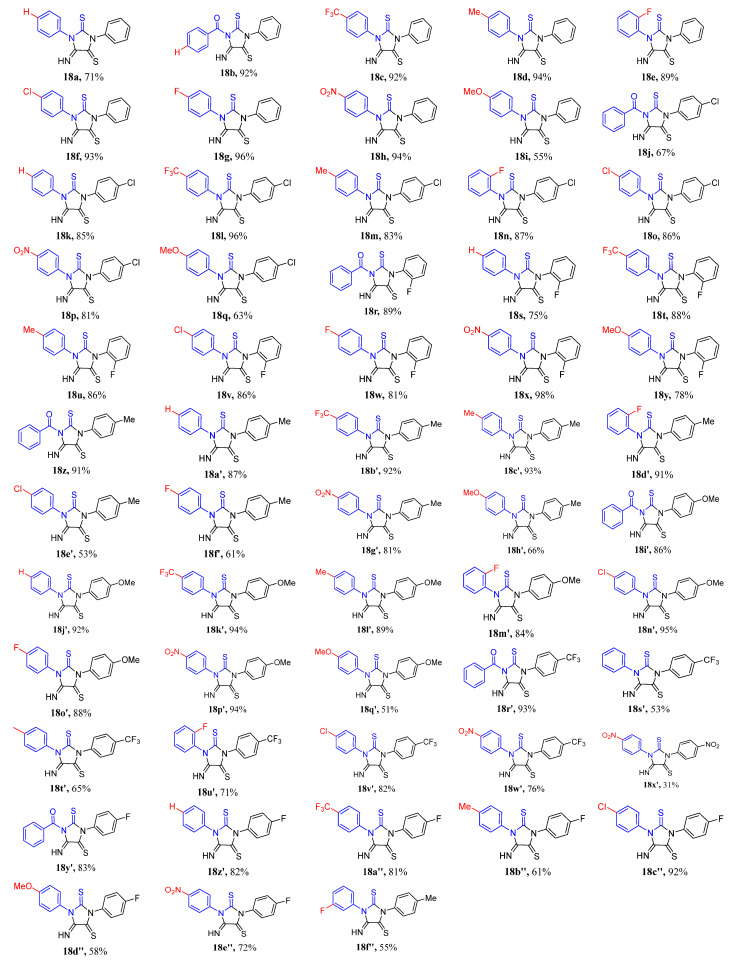
Preparation of variously substituted imidazolidineiminodithiones derivatives **18a**–**z**, **18a′**–**z′**, and **18a″**–**18f″**. All products shown were isolated from the reaction mixture by ether/aqueous workup.

Based on Figure 2, **18i**, **18q**, **18h′**, **18q′**, **18d″**, and **18y**, obtained from **16g**, were consistently obtained in relatively lower yields than the **18i′**, **18j′**, **18k**′, **18l′**, **18m′**, **18n′**, **18o′**, and **18p**′ obtained from **1g**. In all cases of **18i** vs. **18j′**, **18q** vs. **18n′**, **18h′** vs **18l′**, **18d′** vs. **18o′**, and **18y** vs. **18m′**, the yield of the constitutional isomer could not be rectified by any of the aryl substituents installed on **1**. Mechanistically, this suggests that **16** acts as the electrophile since it is partially deactivated by +M groups. On the contrary, the presence of strong electron withdrawing groups (EWG) on **16** (benzyl, nitro, 2-F, and 4-F) mostly resulted in high product yields regardless of the nature of the substituent on **1** with the exception of **18j** and **18x′**. As examples, Bz (**18b**,**r**,**z**/**18i′**,**r′**,**y′**), 2-F (**18e**,**n**/**18d′**,**m′**,**n′**), 4-F (**18g**,**w**/**18f′**,**o′**), and 4-NO_2_ (**18h**,**p**,**x**/**18g′**,**p′**) were obtained in 81–98% yields. Weaker EWG’s like 4-CF_3_ (**18c**,**l**,**t**,**v**/**18b′**,**k′,a″**) and 4-Cl (**18f**,**v**/**18n′**,**v′/18c″**) still produced a good yield range (96–81%). However, **18j** gave the lowest yield for **18x′,** suggesting that a strong EWG with a negative mesomeric effect significantly attenuates the nucleophilicity of **1**. This is supported by the generally observed diminished yields obtained for products from **1** substituted with EWG’s compared to unsubstituted ones like **18f** vs **18o**, **18b** vs. **18j**, **18a** vs **18s′**, **18e** vs. **18u′**, **18f** vs. **18v′**, **18h** vs. **18w′**, and **18h** vs. **18p**. On the other hand, the presence of a strong electron donating group with a positive mesomeric effect significantly enhances the nucleophilicity of **1**. In particular, the 4-methoxy group resulted in a consistently high yield of methoxylated products **18i′**–**18p′** (84–95%) and was synergetic with EWG’s on **16**. As expected, the methoxy group had an opposing effect when used on both reactants (**18q′**). Reactions involving **16b**–**d**,**i**,**j** reached completion as early as 15 min for the benzoyl substituent (**18b**) within a range of 15–120 min, and the time ranged between 24–48 min for the complete conversion to the nitrated products **18x**,**g′**,**p′**,**w′**,**e″**, and 1.6–6 h for formation of the 4-F, 2-F and 4-CF_3_ products (**18o′,f′,e**,**n**,**c**,**t**,**l**,**b′**,**d′**,**m′**,**u′**) (monitored by ^1^HNMR). Even the 4-chloro group accelerated the reaction to afford **18v**,**o**,**f**,**e′**,**n′** within 1.3–3 h. Accordingly, the general order of reactivity is NO_2_ > Bz > 2-F > 4-F > 4-Cl > H > Me > OMe for reactant **16**. We also compared the order of reactivity of the slowest isothiocyanate **16g** (4-MeOC_6_H_4_NCS) with cyanothioformanilide **1** and found the order to be 4-Cl > 4-Me > 4-OMe > Ph > 2-F >> NO_2_.

The synthesis of **18** was amenable to scale-up to gram quantities as demonstrated by the synthesis of **18l**, **18q** and **18g′** on a large scale from **16f/1c**, **16g/1c**, and **16l/1b** (12 mmol scale). The products were isolated in 93%, 61%, and 78%, respectively, with yields comparable to those obtained during small-scale preparation. Regarding reactivity, the isothiocyanates and *N*-arylcyanothioformamides, sterically hindered derivatives, were not prepared and therefore the extent of the impact on yield, stereo- and regiochemistry from the nature, size, or position of the aromatic ring substituent (i.e., *ortho* position) could not be determined and is a topic of current investigation. 

#### 2.1.5. 1D/2D NMR Structural Analysis of a Typical Imidazolidineiminodithione **18**

**Figure 3:**
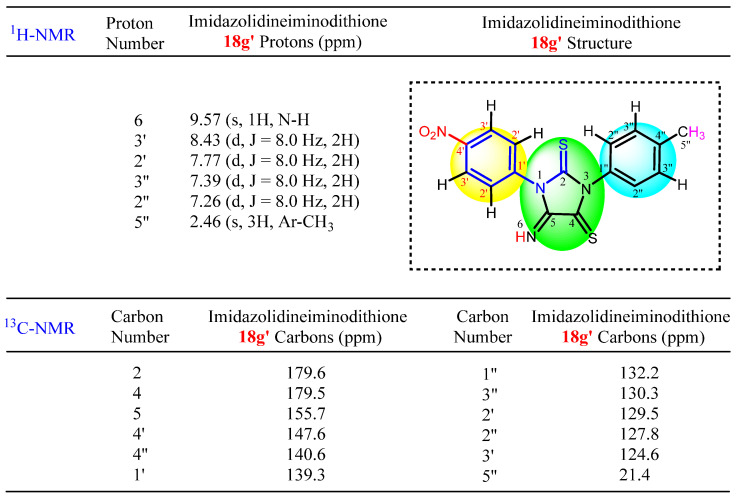
Assigned ^1^H- and ^13^C-NMR chemical shifts of imidazolidineiminodithione **18g′**.

The chemical structures of all compounds (**18a**–**z**, **18a′**–**z′**, and **18a″**–**18e″**) (Figure 3) and formation of the key N-C=S and N-C=NH bonds and imidazolidine ring were confirmed and fully characterized by standard spectroscopic and analytical techniques (MP, IR, 1D NMR, and HRMS). In addition, 2D homonuclear and heteronuclear NMR (^1^H-^1^H-gDQFCOSY, ^1^H-^13^C-gHSQC, and ^1^H-^13^C-gHMBC) was performed on all examples to trace the ^1^H-^1^H and ^1^H-^13^C connectivity and identify the chemical shifts of each carbon and proton (see Supporting Information Sections S3–S320 for related spectra). The physical and spectral data of the above chemical compounds have not been reported in the literature and these are useful references for future comparisons with related compounds. The most distinctive IR signals of **18** are those of the NH and C=N groups, which appear around 3270–3225 cm^−1^ and 1660–1662 in the IR region, respectively. 

Next, using 5-imino-1-(4-nitrophenyl)-3-(p-tolyl)imidazolidine-2,4-dithione (**18g′**) as a representative model example, the relevant NMR spectra (Section S371) and discussion that were used for structural elucidation and chemical shift assignments are shown in the Supporting Information Sections S370–S371. The complete assigned ^1^H- and ^13^C-NMR chemical shifts of **18g′** are shown in Figure 3. 

#### 2.1.6. Crystal Structure Determination of the Imidazolidineiminodithiones **18**

**Figure 4:**
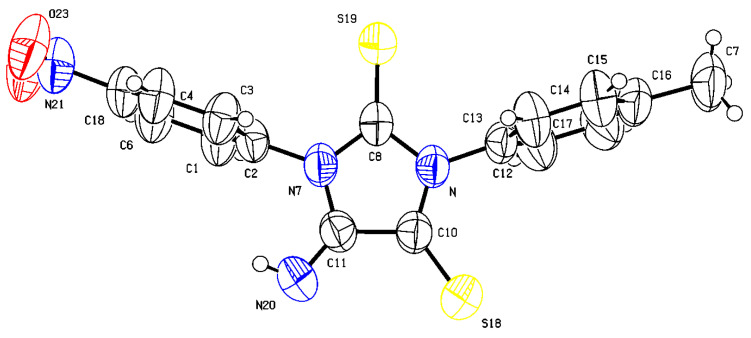
Thermal ellipsoid plots of 5-imino-1-(4-nitrophenyl)-3-(p-tolyl)imidazolidine-2,4-dithione (**18g′**) (Deposition Number 2246320) with ellipsoids drawn at the 50% probability level. For the bond distances (Å) and angles (deg), see the Supporting Information Section.

Although 1D and 2D NMR analysis provided strong evidence to corroborate the formation of the imidazolidineiminodithione scaffold, pleasingly, crystals suitable for X-ray diffraction study were successfully grown and analyzed. Thus, structural validation of **18g′** was also provided by single crystal X-ray crystallography (Figure 4) (see Supporting Information Sections S372–S377 for the compete crystal data). The X-ray structure of **18g′** exhibits a 5-membered imidazolidine ring substituted with 4-tolyl and 4-nitrophenyl groups, as well as two exocyclic sulfur atoms and an imine function. More significantly, the regiochemistry of the observed isomer has been unequivocally established as the dithione. 

The X-ray structure of 5-imino-1-(4-nitrophenyl)-3-phenylimidazolidine-2,4-dithione (**18h**) has also been obtained and reveals a similar molecular framework to its analog **18g′** except for the imine geometry (see Supporting Information S378–S384 for the compete crystal data and a detailed discussion). The imine adopts a *E* geometry, contrary to the observed configuration in **18g′**, with the H-atom oriented away from the aromatic group. Due to the small and exchangeable nature of the imine proton, indicated by its broad absorption in the ^1^H NMR spectrum, steric factors are negligible, allowing for the possibility of either configuration. This observation of the exchangeable nature of the N-H has significant implications for its reactivity. The imine is expected to be highly prone to deprotonation, acting as a reactive nucleophile.

#### 2.1.7. Proposed Mechanism of Formation of the Imidazolidineiminodithiones **18**

**Scheme 5:**
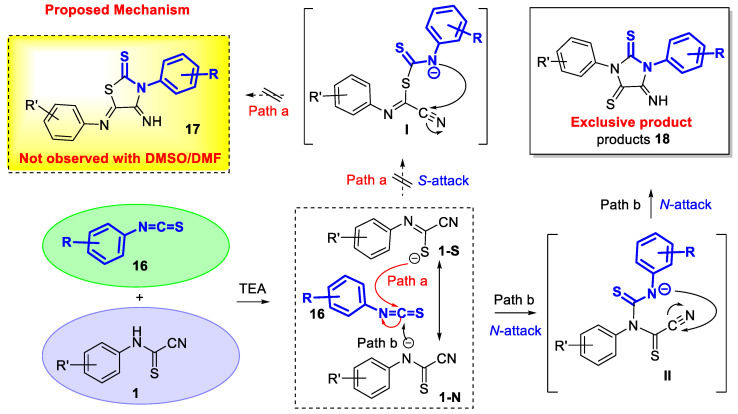
Proposed mechanism for the formation of substituted imidazolidineiminodithiones **18** from isothiocyanates **16** and *N*-arylcyanothioformamides **1**. The reaction “path b” is dominant.

In a recent study on the regioselective synthesis of 5-arylimino-1,3,4-thiadiazoles, we investigated the reaction between *N*-arylcyanothioformamides and hydrazonoyl chlorides [41]. Our findings indicate that hydrazonoyl chlorides act as the electrophile in the initial step of the reaction, while *N*-arylcyanothioformamides serve as the precursor to the nucleophilic species, capable of reacting through either the nitrogen or sulfur atom to produce two potential regioisomers. Scheme 5 illustrates the proposed mechanism of heterocyclization, using phenylcarbamothioyl cyanide (**1a**) as a representative example. The cyanothioformanilide precursor **1a** exists as a mixture of tautomers in solution and undergoes rapid deprotonation by the triethylamine (TEA) base to form the corresponding anion as a triethylammonium salt. This intermediate has been identified and characterized using ^1^H and ^13^C NMR in different solvents. 

The ^1^H NMR analysis of phenylcarbamothioyl cyanide (**1a**) precursor in CDCl_3_ showed a tautomeric ratio of 71:31. Interestingly, the triethylammonium phenylcarbamothioyl cyanide (**1-S** and **1-N**) salt in CDCl_3_ exhibited almost the same resonance ratio of 69:31. However, when different deuterated solvents were used, a significant shift in ratio was observed. In MeOD, the ratio for the same salts was 90:10, while in DMSO-d6, it was 98:2. These results indicate a preference toward the formation of a single major resonance intermediate. Control experiments, on the other hand, have demonstrated that phenylisothiocyanate does not react with TEA. This indirectly confirms that the initial mechanistic step involves deprotonation of the cyanothioformanilide **1**, which then acts as a nucleophile. The nitrogen and sulfur atoms of the deprotonated triethylammonium phenylcarbamothioyl cyanide **1-N/1-S** can both participate in reactions, and the regioisomeric ratio of the products formed depends on the relative resonance ratios between the **1-S** and **1-N** anionic reactants. When **1-S** reacts with the electrophilic carbon of the isothiocyanate **16** via the sulfur anion (path a), the resulting anion **I** undergoes cyclization when the aniline nitrogen attacks the electrophilic nitrile carbon. The resulting regioisomer, in this case, is the undesired thiazolidinethione **17**. Meanwhile, *N*-attack of the **1-N** resonance form on the isothiocyanate **16** (via *path b*) affords **II**. It seems therefore that the exclusive path of cyclization in DMF or DMSO is through intermediate **II**. As shown in path b, intermediate **II** undergoes cyclization via the more basic *N*-attack onto the nitrile function to afford the desired imidazolidineiminodithione **18**. 

Based on the known chemistry of nucleophilic addition reactions, protic solvents are expected to impede reactivity via the nitrogen atom due to strong hydrogen bonding and solvation of the *N*-anion. However, protic solvents like methanol and ethanol (Table 1, Entries 9 and 10) clearly show that the isomeric ratio and preferential formation of a particular isomer is arbitrary and they do not promote a reaction pathway involving cyclization via the more polarized and larger sulfur anion. However, it appears that switching to aprotic solvents like acetonitrile, acetone, DMSO or DMF endorses the preferential formation of the imidazolidineiminodithione in the cases of MeCN, acetone, and DMSO, and its exclusive formation in the case of DMF. The regiochemical outcome of the reaction in DMF suggests that solvation of the triethylammonium cation may be the key to controlling the ambident nucleophilic nature of the cyanothioformanilide and its reaction via the more basic nitrogen atom. Therefore, a single regioisomer forms regardless of the substituents used and the solvent overrides the electronic, kinetic, and thermodynamic properties of the reaction. 

### 2.2. Computational Details 

To rationalize the experimental findings and confirm the proposed mechanism of Scheme 5, we conducted density functional theory (DFT) calculations at the B3LYP-D4/def2-TZVP level in DMF as the implicit solvent using the ORCA software (version 5.0.3) [42] following the computational setup adopted in our previous publications [43]. The atomic charges, electrostatic potential, molecular orbitals, and several DFT global descriptors were computed to understand the most susceptible sites in the reactants. In the following, we will focus on the prototype phenyl thiocyanate **16** (R = H) and phenyl cyanothioformamide **1** (R’=H) compounds (see Scheme 5). Our aim here is to explain the first step in the reaction (i.e., elucidate the initial electron flow) between molecules **1** and **16** leading to species II. The computational analysis is completed with the theoretical study of the reaction mechanism between the aforementioned molecules and the computation of the energetics and barriers. To this end, the nudge elastic band method of Henkelman and coworkers [44] was used to locate the transition states. The wavefunction analysis was carried out with the program Multiwfn [45]. All optimized structures are given in the Supporting Materials. The discussion of the relevant optimized internal coordinates is provided in the Supporting Information as well as the atomic charge and bond order analysis.

#### 2.2.1. Conceptual DFT Global Descriptors

Table 3 shows some conceptual DFT global descriptors derived from the frontier MO eigenvalues at the B3LYP-D4/def2-TZVP level in the implicit DMF solvent. The first descriptor to be considered is the electronic chemical potential, which is the most basic indicator of the electron flow between reactant species. According to Table 3, the chemical potential of compound **1** (μ = −3.302 eV) is higher than compound **16** (−3.821 eV), suggesting an initial electron flow from **1** to **16**, supporting our proposed mechanism in Scheme 5. 

Another powerful descriptor is the global nucleophilicity index *N*, which is the HOMO energy of the compound relative to the reference compound tetracyanoethylene (TCE), with all energies in eV, and both energies were computed at the same level of theory. This index was used in the literature to classify organic molecules as strong (*N* > 3.00 eV), moderate (2.00 eV < *N* < 3.00 eV), and marginal nucleophiles (*N* < 2.00 eV) in polar reactions. According to Table 3, we see that both reactants are nucleophiles, with compound **1** (*N* = 3.327 eV) being a significantly stronger nucleophile than reactant **16** (*N* = 2.379 eV), consistent with our chemical potential analysis above. This also confirms the initial nucleophilic attack of anion **1** on compound **16**.

Our proposed initial electron flow is further confirmed by the global electrophilicity index ω, which quantifies the propensity to accept electrons, with values of ω = 1.296 and 1.440 eV for molecules **1** and **16**, respectively. 

Moreover, from the analysis of the DFT Frontier Molecular Orbital (FMO) eigenvalues, we see that from the HOMO (**1**) => LUMO (**16**) cross gap of 4.119 eV, the electron transfer is more energetically favorable than the HOMO (**16**) => LUMO (**1**) transfer, which features a wider cross gap of 5.156 eV. Finally, the global softness (*s*), defined as the reciprocal of the chemical hardness (*s* = 1/η), predicts that compound **1** (*s* = 0.238 eV^−1^) is slightly softer than compound **16** (*s* = 0.197 eV^−1^). We note that polarizable species have diffuser electron clouds, which make them better suited to initiate the flow of electrons.

Therefore, the analysis of some DFT global descriptors supports the initial electron flow from **1** to **16** upon reaction. However, we must remind the reader that although these DFT descriptors are often useful in qualitative discussions, sometimes they may yield contradictory or wrong predictions, which does not seem to be the case here. Also, the global character of these descriptors makes them devoid of local information about the regioselectivity of the reaction. The above predictions and the regioselectivity is definitely confirmed by looking at the molecular electrostatic potential, the molecular orbitals (discussed in the Supplementary Information), and the energetics (following section).

#### 2.2.2. Mechanism, Energetics, Barriers and Solvent Effect 

We carried out a nudge elastic band (NEB) calculation [44] using as endpoint structures the reactant complex (Supplementary “RS.xyz”) and product (“18.xyz”), and 13 interpolated images between these endpoints, with all at the B3LYP-D4/def2-TZVP level in DMF as the implicit solvent. The Supporting Information shows an electron density isosurface representation of the reactant (RS), transition (TS) and product (PS) states. The calculated minimum energy path (MEP) trajectory (see converged “NEB.xyz” in the Supporting Materials) predicts that firstly one of the 2p lone pair of the negative N5 atom of **1** attacks the electropositive C8 of **16** (see Supplementary Section S390 for the atom labels). The transition state (TS) of the reaction path was successfully found (see Supplementary “TS.xyz”) and features the incipient bond formation between N5 of the thioformamide group of anion **1** and the C8 atom of isothiocyanate **16** with an interatom distance of about 2.086 Å and a longer interatomic distance N6 of **16** with C10 of **1** of about 2.944 Å. A single imaginary frequency at 285.68*i* cm^−1^ was detected for this TS and the associated normal mode is consistent with the formation of an incipient N5-C8 sigma bond (see Supplementary Materials for an xyz trajectory of this normal mode under the name “opt.hess.v006.xyz”). The potential energy profile features a late TS. Upon N5-C8 bond formation, there immediately follows another bond formation between the N6 and C10 atoms leading to an imidazole 5-membered ring (Supplementary “18.xyz”). The driving force is the gain in (local) aromaticity due to the formation of the imidazole ring. At room conditions, the computed reaction Gibbs free energy is exergonic by −2.61 kcal/mol with a forward activation barrier of 12.74 kcal/mol at the B3LYP-D4/def2-TZVP level of theory in the implicit solvent DMF. We expect that in a polar or protic solvent the nucleophilicity of site N5 will be drastically reduced due to intermolecular hydrogen bonds formed with the solvent. This will slow down the kinetics of this reaction pathway (“path b”) and open up the appearance of side products. 

To finish, we carried out a second NEB run in which species **16** is attacked by anion **1** via the S atom (see “path a” in Scheme 5) rather than via the N atom. The calculated barrier turns out to be 1.84 kcal/mol (2.29 in terms of Gibbs free energy at room T) higher than the barrier calculated for the N-attack (12.74 kcal/mol), thus supporting “path b” as the preferred mechanism in DMF. The transition state (Supplementary “TS2.xyz”) features an incipient bond S3–C8 of 2.360 Å and exhibits a single imaginary frequency at −234.33*i* cm^−1^ (see Supplementary trajectory “opt.hess.v006a.xyz” for the animation of this mode). This leads to a stable reaction intermediate I (see Scheme 5 and Supplementary file “I.xyz”) with a S3–C8 bond distance of 1.826 Å, which does not proceed spontaneously to its final product **17,** suggesting the existence of a further barrier. This could be due to the somewhat large separation (2.718 Å) between the N6 and C10 atoms that need to bond to form **17**. We also note that species **17** (see its relaxed structure in Supplementary file “17.xyz”) is about 12 kcal/mol higher in energy than **18**. Thus, compound **18** is more stable thermodynamically, rendering “path b” (see Scheme 5) the preferred mechanism in the DMF solvent, ruling out “path a”. 

## 3. Experimental Section

### 3.1. General Information

All experiments were carried out with magnetic stirring in pre-dried glassware. Reagents and solvents (all acquired from MilliporeSigma, Burlington, MA, USA) were used as supplied without further purification. The progress of the reactions was monitored using analytical thin-layer chromatography (TLC) on HSGF 254 silica gel plates, visualized under UV light at 254 nm. ^1^H and ^13^C NMR spectra were recorded in CDCl_3_ and DMSO-d_6_ on a Varian 400 MHz NMR spectrometer. NMR chemical shifts (δ) are reported in ppm, referenced to residual solvent peaks (^1^H-NMR δ 7.26/2.50 for CDCl_3_/DMSO-d_6_; δ 77.0/39.52 for CDCl_3_/DMSO-d_6_). Peak multiplicities are designated as follows: br s = broad signal, s = singlet, d = doublet, t = triplet, m = multiplet. IR spectra were recorded on a Thermo Nicolet Nexus 470 FT-IR spectrometer. High-resolution mass spectrometry (HRMS) was performed using a Waters Q-TOF Premier mass spectrometer with electrospray ionization (ESI). Single-crystal X-ray diffraction data (SC-XRD) were collected using a Rigaku Oxford Diffraction XtaLAB Synergy-S, equipped with two microfocus PhotonJet-S sources, Cu Kα radiation (λ = 1.54184 Å), Mo Kα radiation (λ = 0.71073 Å), and a HyPix-6000HE hybrid photon counting detector. Data processing and refinement were carried out using CrysAlisPro and Olex2 software (https://rigaku.com/products/crystallography/x-ray-diffraction/crysalispro and https://www.olexsys.org/olex2/, both accessed on 18 August 2024), respectively. All compounds measured with X-ray were crystallized from diethyl ether. For the computational methods and additional references, see the Supporting Information.

#### General Synthesis of Imidazolidineiminodithiones Derivatives **18a**–**z**, **18a′**–**z′**, and **18a″**–**18f″**

A stirred solution of the *N*-arylcyanothioformamide **1** (1 mmol) and isothiocyanate **16** (1 mmol) in DMF (10 mL) was treated with 20 mol% triethylamine (TEA) (28 µL, 0.2 mmol) and the mixture was stirred for 24 h at ambient temperature (22 °C). The resulting dark green mixture was poured into 20 mL of diethyl ether, washed with saturated NaCl solution (3 × 20 mL), dried (Na_2_SO_4_), and concentrated in vacuo to afford the desired imidazolidineiminodithiones **18a**–**z**, **18a′**–**z′**, and **18a″**–**18e″** in the reported yields. No further purification was needed as indicated by NMR analysis.

### 3.2. Experimental Data

5-imino-1,3-diphenylimidazolidine-2,4-dithione (**18a**). 

Brown solid (71% yield); IR (KBr) 3232 (NH), 1652 (C=N), 1593, 1494 (C=S), 1415, 1391, 1287, 1159, 1065 cm^−1^; ^1^H NMR (CDCl_3_, 400 MHz) δ 9.50 (s, 1H, NH), 7.62–7.48 (m, 8H, Ar-H), 7.42–7.37 (m, 2H, Ar-H) ppm; ^13^C NMR (CDCl_3_, 100 MHz) δ 180.4 (C=S), 180.1 (C=S), 156.5 (**C**=NH), 135.9 (*N*_(1)_-**C_q_**), 134.7 (*N*_(3)_-**C_q_**), 129.6 (CH), 129.3 (2xCH), 129.1 (2xCH), 129.0 (CH), 128.8 (2xCH), 128.7 (2xCH) ppm; ^1^H NMR (DMSO-d_6_, 400 MHz) δ 9.88 (s, 1H, NH), 7.62–7.42 (m, 10H, Ar-H) ppm; ^13^C NMR (DMSO-d_6_, 100 MHz) δ 181.2 (C=S), 181.1 (C=S), 156.3 (**C**=NH), 135.2 (*N*_(1)_-**C_q_**), 133.9 (*N*_(3)_-**C_q_**), 130.0 (CH), 129.6 (CH), 129.5 (2xCH), 129.4 (2xCH), 128.3 (2xCH), 128.2 (2xCH) ppm; HRMS (ESI^+^): *m*/*z* [M + H]^+^ calcd for C_15_H_12_N_3_S_2_: 298.0473; found: 298.0465. 

(5-imino-3-phenyl-2,4-dithioxoimidazolidin-1-yl)(phenyl)methanone (**18b**). 

Brown solid (92% yield); IR (KBr) 3240 (NH), 1668 (C=O), 1597 (C=N), 1496 (C=S), 1384, 1314, 1292, 1249, 1128, 1052 cm^−1^; ^1^H NMR (CDCl_3_, 400 MHz) δ 9.58 (s, 1H, NH), 8.04 (d, *J* = 8.0 Hz, 2H, Ar-H), 7.73 (t, *J* = 8.0 Hz, 1H, Ar-H), 7.62–7.53 (m, 5H, Ar-H), 7.37 (d, *J* = 8.0 Hz, 2H, Ar-H) ppm; ^13^C NMR (CDCl_3_, 100 MHz) δ 179.6 (C=S), 178.1 (C=S), 167.0 (C=O), 155.0 (**C**=NH), 135.6 (CH), 134.3 (O=C-**C_q_**), 131.2 (2xCH), 131.1 (*N*_(3)_-**C_q_**), 130.2 (CH), 129.6 (2xCH), 129.2 (2xCH), 128.2 (2xCH) ppm; HRMS (ESI^+^): *m*/*z* [M + H]^+^ calcd for C_16_H_12_N_3_OS_2_: 326.0422; found: 326.0433. 

5-imino-3-phenyl-1-(4-(trifluoromethyl)phenyl)imidazolidine-2,4-dithione (**18c**).

Dark brown solid (92% yield); IR (KBr) 3227 (NH), 1659 (C=N), 1612, 1497 (C=S), 1416, 1386, 1320, 1281, 1185, 1125, 1070 cm^−1^; ^1^H NMR (CDCl_3_, 400 MHz) δ 9.55 (s, 1H, NH), 7.84 (d, *J* = 8.0 Hz, 2H, Ar-H_p-(trifluoromethyl)phenyl_), 7.69 (d, *J* = 8.0 Hz, 2H, Ar-H_p-(trifluoromethyl)phenyl_), 7.63–7.53 (m, 3H, Ar-H), 7.42–7.36 (m, 2H, Ar-H) ppm; ^13^C NMR (CDCl_3_, 100 MHz) δ 179.9 (C=S), 179.7 (C=S), 156.0 (**C**=NH), 136.9 (*N*_(1)_-**C_q_**), 135.0 (*N*_(3)_-**C_q_**), 131.3 (q, *J* = 33.0 Hz, **C**-CF_3_), 130.2 (CH), 129.6 (2xCH), 128.9 (2xCH), 128.2 (2xCH), 126.6 (q, *J* = 4.0 Hz, 2xCH), 123.5 (q, *J* = 271.0 Hz, CF_3_) ppm; HRMS (ESI^+^): *m*/*z* [M + H]^+^ calcd for C_16_H_11_N_3_F_3_S_2_: 366.0347; found: 366.0358.

5-imino-3-phenyl-1-(p-tolyl)imidazolidine-2,4-dithione (**18d**). 

Brown solid (94% yield); IR (KBr) 3209 (NH), 1654 (C=N), 1592, 1515 (C=S), 1497, 1415, 1390, 1275, 1129, 1071 cm^−1^; ^1^H NMR (CDCl_3_, 400 MHz) δ 9.48 (s, 1H, NH), 7.61–7.53 (m, 3H, Ar-H), 7.42–7.35 (m, 6H, Ar-H), 2.43 (s, CH_3_) ppm; ^13^C NMR (CDCl_3_, 100 MHz) δ 180.5 (C=S), 180.1 (C=S), 156.5 (**C**=NH), 139.7 (CH_3_-**C**), 135.2 (*N*_(3)_-**C_q_**), 131.2 (*N*_(1)_-**C_q_**), 130.1 (2xCH), 129.9 (CH), 129.4 (2xCH), 128.2 (2xCH), 127.8 (2xCH), 21.3 (CH_3_) ppm; HRMS (ESI^+^): *m*/*z* [M + H]^+^ calcd for C_16_H_14_N_3_S_2_: 312.0629; found: 312.0635. 

1-(2-fluorophenyl)-5-imino-3-phenylimidazolidine-2,4-dithione (**18e**).

Reddish brown solid (89% yield); IR (KBr) 3247 (NH), 1659 (C=N), 1594, 1509 (C=S), 1497, 1460, 1412, 1390, 1305, 1165, 1066 cm^−1^; ^1^H NMR (DMSO-d_6_, 400 MHz) δ 10.09 (s, 1H, NH), 7.69–7.39 (m, 9H, Ar-H) ppm; ^13^C NMR (DMSO-d_6_, 100 MHz) δ 180.8 (C=S), 180.5 (C=S), 157.6 (d, *J* = 250.0 Hz, C-F), 155.1 (**C**=NH), 135.6 (*N*_(3)_-**C_q_**), 131.8 (d, *J* = 8.0 Hz, CH), 131.3 (2xCH), 129.8 (CH), 129.4 (2xCH), 128.6 (CH), 125.2 (d, *J* = 4.0 Hz, CH), 121.0 (d, *J* = 12.0 Hz, *N*_(1)_-**C_q_**), 116.6 (d, *J* = 19.0 Hz, CH) ppm; HRMS (ESI^+^): *m*/*z* [M + H]^+^ calcd for C_15_H_11_N_3_FS_2_: 316.0378; found: 316.0371. 

1-(4-chlorophenyl)-5-imino-3-phenylimidazolidine-2,4-dithione (**18f**). 

Light brown solid (93% yield); IR (KBr) 3234 (NH), 1656 (C=N), 1593, 1493 (C=S), 1413, 1382, 1289, 1258, 1159, 1066 cm^−1^; ^1^H NMR (CDCl_3_, 400 MHz) δ 9.51 (s, 1H, NH), 7.62–7.55 (m, 3H, Ar-H), 7.53 (d, *J* = 8.0 Hz, 2H, Ar-H_p-chlorophenyl_), 7.46 (d, *J* = 8.0 Hz, 2H, Ar-H_p-chlorophenyl_), 7.40–7.36 (m, 2H, Ar-H) ppm; ^13^C NMR (CDCl_3_, 100 MHz) δ 180.1 (C=S), 179.9 (C=S), 156.2 (**C**=NH), 135.4 (**C**-Cl), 135.1 (*N*_(1)_-**C_q_**), 132.3 (*N*_(3)_-**C_q_**), 130.1 (CH), 129.7 (2xCH_p-chlorophenyl_), 129.6 (2xCH_p-chlorophenyl_), 129.5 (2xCH), 128.2 (2xCH) ppm; HRMS (ESI^+^): *m*/*z* [M + H]^+^ calcd for C_15_H_11_N_3_ClS_2_: 332.0083; found: 332.0074. 

1-(4-fluorophenyl)-5-imino-3-phenylimidazolidine-2,4-dithione (**18g**). 

Brown solid (96% yield); IR (KBr) 3233 (NH), 1657 (C=N), 1511 (C=S), 1417, 1385, 1287, 1252, 1067 cm^−1^; ^1^H NMR (CDCl_3_, 400 MHz) δ 9.50 (s, 1H, NH), 7.62–7.54 (m, 3H, Ar-H_phenyl_), 7.52–7.45 (m, 2H, Ar-H_phenyl_), 7.42–7.35 (m, 2H, Ar-H_phenyl_), 7.30–7.22 (m, 2H, Ar-H_phenyl_) ppm; ^13^C NMR (CDCl_3_, 100 MHz) δ 180.3 (C=S), 180.0 (C=S), 162.7 (d, *J* = 248.0 Hz, C-F), 156.4 (**C**=NH), 135.1 (*N*_(3)_-**C_q_**), 130.2 (CH_phenyl_), 130.1 (d, *J* = 8.0 Hz, 2xCH_p-fluorophenyl_), 129.7 (d, *J* = 3.0 Hz, *N*_(1)_-**C_q_**), 129.5 (2xCH_phenyl_), 128.2 (2xCH_phenyl_), 116.6 (d, *J* = 23.0 Hz, 2xCH_p-fluorophenyl_) ppm; HRMS (ESI^+^): *m*/*z* [M + H]^+^ calcd for C_15_H_11_FN_3_S_2_: 316.0378; found: 316.0388.

5-imino-1-(4-nitrophenyl)-3-phenylimidazolidine-2,4-dithione (**18h**). 

Dark green solid (94% yield); IR (KBr) 3236 (NH), 1658 (C=N), 1593, 1529 (C=S), 1382, 1349, 1301, 1162, 1066 cm^−1^; ^1^H NMR (CDCl_3_, 400 MHz) δ 9.58 (s, 1H, NH), 8.43 (d, *J* = 8.0 Hz, 2H, Ar-H_p-nitrophenyl_), 7.78 (d, *J* = 8.0 Hz, 2H, Ar-H_p-nitrophenyl_), 7.63–7.54 (m, 3H, Ar-H_ph_), 7.40–7.35 (m, 2H, Ar-H_ph_) ppm; ^13^C NMR (CDCl_3_, 100 MHz) δ 179.5 (C=S), 179.4 (C=S), 155.7 (**C**=NH), 147.6 (NO_2_-**C_q_**), 139.2 (*N*_(1)_-**C_q_**), 124.9 (*N*_(3)_-**C_q_**), 130.2 (CH), 129.6 (2xCH_p-nitrophenyl_), 129.5 (2xCH_ph_), 128.2 (2xCH_ph_), 124.6 (2xCH_p-nitrophenyl_) ppm; HRMS (ESI^+^): *m*/*z* [M + H]^+^ calcd for C_15_H_11_N_4_O_2_S_2_: 343.0323; found: 343.0329. 

5-imino-1-(4-methoxyphenyl)-3-phenylimidazolidine-2,4-dithione (**18i**). 

Brown solid (55% yield); IR (KBr) 3215 (NH), 1656 (C=N), 1591, 1514 (C=S), 1391, 1293, 1247, 1126, 1073 cm^−1^; ^1^H NMR (CDCl_3_, 400 MHz) δ 9.46 (s, 1H, NH), 7.59–7.53 (m, 3H, Ar-H_ph_), 7.40 (d, *J* = 8.0 Hz, 2H, Ar-H_p-methoxyphenyl_), 7.39–7.36 (m, 2H, Ar-H_ph_), 7.06 (d, *J* = 8.0 Hz, 2H, Ar-H_p-methoxyphenyl_), 3.85 (s, 3H, OCH_3_) ppm; ^13^C NMR (CDCl_3_, 100 MHz) δ 180.5 (C=S), 180.1 (C=S), 159.9 (OCH_3_-**C_q_**), 156.5 (**C**=NH), 135.1 (*N*_(3)_-**C_q_**), 129.8 (CH_ph_), 129.3 (2xCH_ph_), 129.2 (2xCH_p-methoxyphenyl_), 128.1 (2xCH_ph_), 126.1 (*N*_(1)_-**C_q_**), 114.4 (2xCH_p-methoxyphenyl_), 55.3 (OCH_3_) ppm; HRMS (ESI^+^): *m*/*z* [M + H]^+^ calcd for C_16_H_14_N_3_OS_2_: 328.0578; found: 328.0583.

(3-(4-chlorophenyl)-5-imino-2,4-dithioxoimidazolidin-1-yl)(phenyl)methanone (**18j**). 

Light brown solid (67% yield); IR (KBr) 3227 (NH), 1726 (C=O), 1662 (C=N), 1599, 1494 (C=S), 1399, 1288, 1185, 1015 cm^−1^; ^1^H NMR (CDCl_3_, 400 MHz) δ 9.59 (s, 1H, NH), 8.02 (d, *J* = 8.0 Hz, 2H, Ar-H), 7.72 (t, *J* = 8.0 Hz, 1H, Ar-H), 7.58–7.51 (m, 4H, Ar-H), 7.32 (d, *J* = 8.0 Hz, 2H, Ar-H) ppm; ^13^C NMR (CDCl_3_, 100 MHz) δ 179.4 (C=S), 177.7 (C=S), 166.9 (C=O), 154.9 (**C**=NH), 136.3 (**C**-Cl), 135.7 (CH), 132.6 (*N*_(3)_-**C_q_**), 131.2 (2xCH), 131.1 (*N*_(1)_-**C_q_**), 130.0 (2xCH), 129.7 (2xCH), 129.2 (2xCH), ppm; HRMS (ESI^+^): *m*/*z* [M + H]^+^ calcd for C_16_H_11_ClN_3_OS_2_: 360.0032; found: 360.0025. 

3-(4-chlorophenyl)-5-imino-1-phenylimidazolidine-2,4-dithione (**18k**). 

Light brown solid (85% yield); IR (KBr) 3242 (NH), 1651 (C=N), 1595, 1492 (C=S), 1416, 1391, 1289, 1158, 1122, 1067 cm^−1^; ^1^H NMR (CDCl_3_, 400 MHz) δ 9.51 (s, 1H, NH), 7.64–7.45 (m, 7H, Ar-H), 7.35 (d, *J* = 8.0 Hz, 2H, Ar-H_p-chlorophenyl_) ppm; ^13^C NMR (CDCl_3_, 100 MHz) δ 180.0 (C=S), 179.9 (C=S), 156.4 (**C**=NH), 136.1 (**C**-Cl), 133.8 (*N*_(1)_-**C_q_**), 133.5 (*N*_(3)_-**C_q_**), 129.8 (2xCH_p-chlorophenyl_), 129.7 (2xCH_p-chlorophenyl_), 129.6 (CH), 129.5 (2xCH), 128.2 (2xCH) ppm; HRMS (ESI^+^): *m*/*z* [M + H]^+^ calcd for C_15_H_11_N_3_ClS_2_: 332.0083; found: 332.0071. 

3-(4-chlorophenyl)-5-imino-1-(4-(trifluoromethyl)phenyl)imidazolidine-2,4-dithione (**18l**). 

Dark green solid (96% yield); IR (KBr) 3226 (NH), 1660 (C=N), 1612, 1493 (C=S), 1410, 1383, 1319, 1286, 1185, 1127, 1070 cm^−1^; ^1^H NMR (CDCl_3_, 400 MHz) δ 9.56 (s, 1H, NH), 7.84 (d, *J* = 8.0 Hz, 2H, Ar-H_p-(trifluoromethyl)phenyl_), 7.67 (d, *J* = 8.0 Hz, 2H, Ar-H_p-(trifluoromethyl)phenyl_), 7.56 (d, *J* = 8.0 Hz, 2H, Ar-H_p-chlorophenyl_), 7.34 (d, *J* = 8.0 Hz, 2H, Ar-H_p-chlorophenyl_) ppm; ^13^C NMR (CDCl_3_, 100 MHz) δ 179.5 (C=S), 179.4 (C=S), 155.9 (**C**=NH), 136.8 (**C**-Cl), 136.2 (*N*_(1)_-**C_q_**), 133.3 (*N*_(3)_-**C_q_**), 131.4 (q, *J* = 32.0 Hz, **C**-CF_3_), 129.9 (2xCH_p-chlorophenyl_), 129.6 (2xCH_p-chlorophenyl_), 128.8 (2xCH_p-(trifluoromethyl)phenyl_), 126.6 (q, *J* = 4.0 Hz, 2xCH _p-(trifluoromethyl)phenyl_), 123.5 (q, *J* = 274.0 Hz, CF_3_) ppm; HRMS (ESI^+^): *m*/*z* [M + H]^+^ calcd for C_16_H_10_ClN_3_F_3_S_2_: 399.9957; found: 399.9948.

3-(4-chlorophenyl)-5-imino-1-(p-tolyl)imidazolidine-2,4-dithione (**18m**). 

Light brown solid (83% yield); IR (KBr) 3245 (NH), 1654 (C=N), 1513 (C=S), 1491, 1417, 1390, 1287, 1156, 1123, 1064 cm^−1^; ^1^H NMR (CDCl_3_, 400 MHz) δ 9.51 (s, 1H, NH), 7.54 (d, *J* = 8.0 Hz, 2H, Ar-H_p-chlorophenyl_), 7.41–7.36 (m, 4H, Ar-H_p-tolyl_), 7.35 (d, *J* = 8.0 Hz, 2H, Ar-H_p-chlorophenyl_), 2.44 (s, 3H, CH_3_) ppm; ^13^C NMR (CDCl_3_, 100 MHz) δ 180.2 (C=S), 180.0 (C=S), 156.5 (**C**=NH), 139.8 (**C_q_**-Me), 136.0 (**C**-Cl), 133.5 (*N*_3_-**C_q_**), 131.1 (*N*_1_-**C_q_**), 130.2 (2xCH_p-tolyl_), 129.8 (2xCH_p-chlorophenyl_), 129.7 (2xCH_p-chlorophenyl_), 127.8 (2xCH_p-tolyl_), 21.3 (CH_3_) ppm; HRMS (ESI^+^): *m*/*z* [M + H]^+^ calcd for C_16_H_13_N_3_ClS_2_: 346.0239; found: 346.0248.

3-(4-chlorophenyl)-1-(2-fluorophenyl)-5-iminoimidazolidine-2,4-dithione (**18n**).

Light brown solid (87% yield); IR (KBr) 3243 (NH), 1660 (C=N), 1591, 1508 (C=S), 1492, 1413, 1387, 1301, 1163, 1162, 1065 cm^−1^; ^1^H NMR (CDCl_3_, 400 MHz) δ 9.50 (s, 1H, NH), 7.55 (d, *J* = 8.0 Hz, 2H, Ar-H_p-chlorophenyl_), 7.53–7.46 (m, 2H, Ar-H), 7.36 (d, *J* = 8.0 Hz, 2H, Ar-H_p-chlorophenyl_), 7.34–7.28 (m, 2H, Ar-H) ppm; ^13^C NMR (CDCl_3_, 100 MHz) δ 179.9 (C=S), 179.4 (C=S), 157.8 (d, *J* = 252.0 Hz, C-F), 155.5 (**C**=NH), 136.1 (**C**-Cl), 133.4 (*N*_(3)_-**C_q_**), 131.9 (d, *J* = 8.0 Hz, CH), 130.3 (CH), 129.8 (2xCH), 129.7 (2xCH), 125.0 (d, *J* = 4.0 Hz, CH), 121.5 (d, *J* = 12.0 Hz, *N*_(1)_-**C_q_**), 117.1 (d, *J* = 19.0 Hz, CH) ppm; HRMS (ESI^+^): *m*/*z* [M + H]^+^ calcd for C_15_H_10_N_3_FClS_2_: 349.9989; found: 349.9978. 

1,3-bis(4-chlorophenyl)-5-iminoimidazolidine-2,4-dithione (**18o**). 

Light brown solid (86% yield); IR (KBr) 3233 (NH), 1652 (C=N), 1493 (C=S), 1395, 1293 1161, 1091 cm^−1^; ^1^H NMR (CDCl_3_, 400 MHz) δ 9.52 (s, 1H, NH), 7.55 (d, *J* = 8.0 Hz, 2H, Ar-H), 7.54 (d, *J* = 8.0 Hz, 2H, Ar-H), 7.44 (d, *J* = 8.0 Hz, 2H, Ar-H), 7.33 (d, *J* = 8.0 Hz, 2H, Ar-H) ppm; ^13^C NMR (CDCl_3_, 100 MHz) δ 179.7 (C=S), 179.6 (C=S), 156.1 (**C**=NH), 136.2 (**C**-Cl), 135.6 (**C**-Cl), 133.4 (*N*-**C_q_**), 132.2 (*N*-**C_q_**), 129.9 (2xCH), 129.8 (2xCH), 129.7 (2xCH), 129.5 (2xCH) ppm; HRMS (ESI^+^): *m*/*z* [M + H]^+^ calcd for C_15_H_10_N_3_Cl_2_S_2_: 365.9693; found: 365.9698. 

3-(4-chlorophenyl)-5-imino-1-(4-nitrophenyl)imidazolidine-2,4-dithione (**18p**).

Green solid (81% yield); IR (KBr) 3230 (NH), 1661 (C=N), 1595, 1522 (C=S), 1492, 1407, 1347, 1286, 1089, 856 cm^−1^; ^1^H NMR (DMSO-d_6_, 400 MHz) δ 10.12 (s, 1H, NH), 8.45 (d, *J* = 8.0 Hz, 2H, Ar-H_p-nitrophenyl_), 7.84 (d, *J* = 8.0 Hz, 2H, Ar-H_p-nitrophenyl_), 7.68 (d, *J* = 8.0 Hz, 2H, Ar-H_p-chlorophenyl_), 7.51 (d, *J* = 8.0 Hz, 2H, Ar-H_p-chlorophenyl_) ppm; ^13^C NMR (DMSO-d_6_, 100 MHz) δ 180.9 (C=S), 180.5 (C=S), 155.6 (**C**=NH), 147.3 (NO_2_-**C_q_**), 140.1 (*N*_(1)_-**C_q_**), 134.5 (C-Cl), 134.4 (*N*_(3)_-**C_q_**), 130.6 (2xCH_p-chlorophenyl_), 130.4 (2xCH_p-nitrophenyl_), 129.6 (2xCH_p-chlorophenyl_), 124.5 (2xCH_p-nitrophenyl_) ppm; HRMS (ESI^+^): *m*/*z* [M + H]^+^ calcd for C_15_H_10_ClN_4_O_2_S_2_: 376.9934; found: 376.9945. 

3-(4-chlorophenyl)-5-imino-1-(4-methoxyphenyl)imidazolidine-2,4-dithione (**18q**). 

Brown solid (63% yield); IR (KBr) 3217 (NH), 1654 (C=N), 1514 (C=S), 1493 (C=S), 1384, 1284, 1250 cm^−1^; ^1^H NMR (CDCl_3_, 400 MHz) δ 9.49 (s, 1H, NH), 7.54 (d, *J* = 8.0 Hz, 2H, Ar-H_p-chlorophenyl_), 7.39 (d, *J* = 8.0 Hz, 2H, Ar-H_p-methoxyphenyl_), 7.34 (d, *J* = 8.0 Hz, 2H, Ar-H_p-chlorophenyl_), 7.06 (d, *J* = 8.0 Hz, 2H, Ar-H_p-methoxyphenyl_), 3.86 (s, 3H, OCH_3_) ppm; ^13^C NMR (CDCl_3_, 100 MHz) δ 180.3 (C=S), 180.1 (C=S), 160.2 (OCH_3_-**C_q_**), 156.6 (**C**=NH), 136.0 (**C**-Cl), 133.6 (*N*_(3)_-**C_q_**), 129.8 (2xCH_p-chlorophenyl_), 129.7 (2xCH_p-chlorophenyl_), 129.3 (2xCH_p-methoxyphenyl_), 126.3 (*N*_(1)_-**C_q_**), 114.8 (2xCH_p-methoxyphenyl_), 55.5 (OCH_3_) ppm; HRMS (ESI^+^): *m*/*z* [M + H]^+^ calcd for C_16_H_13_N_3_ClOS_2_: 360.0189; found: 362.0176.

(3-(2-fluorophenyl)-5-imino-2,4-dithioxoimidazolidin-1-yl)(phenyl)methanone (**18r**).

Light brown solid (89% yield); IR (KBr) 3235 (NH), 1715 (C=O), 1667 (C=N), 1596, 1503 (C=S), 1385, 1310, 1190, 1025 cm^−1^; ^1^H NMR (CDCl_3_, 400 MHz) δ 9.60 (s, 1H, NH), 8.02 (d, *J* = 8.0 Hz, 2H, Ar-H), 7.72 (t, *J* = 8.0 Hz, 1H, Ar-H), 7.60–7.52 (m, 3H, Ar-H), 7.43–7.27 (m, 3H, Ar-H) ppm; ^13^C NMR (CDCl_3_, 100 MHz) δ 179.1 (C=S), 177.1 (C=S), 166.8 (C=O), 157.3 (d, *J* = 253.0 Hz, C-F), 154.9 (**C**=NH), 135.6 (CH), 132.5 (d, *J* = 8.0 Hz, CH), 131.2 (2xCH), 131.0 (**C_q_**), 130.2 (CH), 129.2 (2xCH), 125.0 (d, *J* = 4.0 Hz, CH), 122.0 (d, *J* = 13.0 Hz, *N*_(3)_-**C_q_**), 117.1 (d, *J* = 18.0 Hz, CH) ppm; HRMS (ESI^+^): *m*/*z* [M + H]^+^ calcd for C_16_H_11_N_3_FOS_2_: 344.0328; found: 344.0341. 

3-(2-fluorophenyl)-5-imino-1-phenylimidazolidine-2,4-dithione (**18s**). 

Red orange solid (75% yield); IR (KBr) 3223 (NH), 1658 (C=N), 1593, 1499 (C=S), 1414, 1389, 1302, 1227, 1127, 1067 cm^−1^; ^1^H NMR (CDCl_3_, 400 MHz) δ 9.52 (s, 1H, NH), 7.62–7.49 (m, 6H, Ar-H), 7.46–7.29 (m, 3H, Ar-H) ppm; ^13^C NMR (CDCl_3_, 100 MHz) δ 179.7 (C=S), 179.4 (C=S), 157.4 (d, *J* = 253.0 Hz, C-F), 156.4 (C=NH), 133.9 (*N*_(1)_-**C_q_**), 132.2 (d, *J* = 8.0 Hz, CH), 130.2 (CH), 129.6 (CH), 129.5 (2xCH), 128.2 (2xCH), 124.9 (d, *J* = 4.0 Hz, CH), 122.8 (d, *J* = 13.0 Hz, *N*_(3)_-**C_q_**), 117.0 (d, *J* = 18.0 Hz, CH) ppm; HRMS (ESI^+^): *m*/*z* [M + H]^+^ calcd for C_15_H_11_N_3_FS_2_: 316.0378; found: 316.0369. 

3-(2-fluorophenyl)-5-imino-1-(4-(trifluoromethyl)phenyl)imidazolidine-2,4-dithione (**18t**). 

Light brown solid (88% yield); IR (KBr) 3230 (NH), 1666 (C=N), 1596, 1505 (C=S), 1416, 1386, 1294, 1165, 1068 cm^−1^; ^1^H NMR (CDCl_3_, 400 MHz) δ 9.56 (s, 1H, NH), 7.84 (d, *J* = 8.0 Hz, 2H, Ar-H_p-(trifluoromethyl)phenyl_), 7.70 (d, *J* = 8.0 Hz, 2H, Ar-H_p-(trifluoromethyl)phenyl_), 7.62–7.54 (m, 1H, Ar-H_2-fluorophenyl_), 7.45–7.29 (m, 3H, Ar-H_2-fluorophenyl_) ppm; ^13^C NMR (CDCl_3_, 100 MHz) δ 179.2 (C=S), 178.9 (C=S), 157.4 (d, *J* = 253.0 Hz, C-F), 155.9 (**C**=NH), 136.8 (*N*_(1)_-**C_q_**), 132.4 (d, *J* = 8.0 Hz, CH_2-fluorophenyl_), 131.4 (q, *J* = 33.0 Hz, **C**-CF_3_), 130.2 (CH_2-fluorophenyl_), 128.8 (2xCH_p-(trifluoromethyl)phenyl_), 126.6 (q, *J* = 4.0 Hz, 2xCH _p-(trifluoromethyl)phenyl_), 125.0 (d, *J* = 4.0 Hz, CH_2-fluorophenyl_), 123.5 (q, *J* = 271.0 Hz, CF_3_), 122.7 (d, *J* = 13.0 Hz, *N*_(3)_-**C_q_**), 117.1 (d, *J* = 19.0 Hz, CH_2-fluorophenyl_) ppm; HRMS (ESI^+^): *m*/*z* [M + H]^+^ calcd for C_16_H_10_N_3_F_4_S_2_: 384.0252; found: 384.0257.

3-(2-fluorophenyl)-5-imino-1-(p-tolyl)imidazolidine-2,4-dithione (**18u**). 

Burgundy solid (86% yield); IR (KBr) 3234 (NH), 1655 (C=N), 1593, 1512 (C=S), 1416, 1387, 1300, 1127, 1064 cm^−1^; ^1^H NMR (CDCl_3_, 400 MHz) δ 9.49 (s, 1H, NH), 7.59–7.52 (m, 1H, Ar-H), 7.44–7.28 (m, 7H, Ar-H), 2.43 (s, 3H, CH_3_) ppm; ^13^C NMR (CDCl_3_, 100 MHz) δ 179.8 (C=S), 179.5 (C=S), 157.4 (d, *J* = 253.0 Hz, C-F), 156.5 (**C**=NH), 139.8 (**C_q_**-CH_3_), 132.2 (d, *J* = 8.0 Hz, CH), 131.1 (*N*_(1)_-**C_q_**), 130.2 (2xCH), 130.1 (CH), 127.9 (2xCH), 124.9 (d, *J* = 4.0 Hz, CH), 122.9 (d, *J* = 13.0 Hz, *N*_(3)_-**C_q_**), 117.0 (d, *J* = 19.0 Hz, CH), 21.3 (CH_3_) ppm; HRMS (ESI^+^): *m*/*z* [M + H]^+^ calcd for C_16_H_13_N_3_FS_2_: 330.0535; found: 330.0543. 

1-(4-chlorophenyl)-3-(2-fluorophenyl)-5-iminoimidazolidine-2,4-dithione (**18v**). 

Light brown solid (86% yield); IR (KBr) 3242 (NH), 1655 (C=N), 1594, 1494 (C=S), 1415, 1387, 1302, 1232, 1164, 1128, 1066 cm^−1^; ^1^H NMR (CDCl_3_, 400 MHz) δ 9.52 (s, 1H, NH), 7.54 (d, *J* = 8.0 Hz, 2H, Ar-H_p-chlorophenyl_), 7.60–7.51 (m, 1H, Ar-H), 7.47 (d, *J* = 8.0 Hz, 2H, Ar-H_p-chlorophenyl_), 7.44–7.27 (m, 3H, Ar-H) ppm; ^13^C NMR (CDCl_3_, 100 MHz) δ 179.4 (C=S), 179.1 (C=S), 157.4 (d, *J* = 253.0 Hz, C-F), 156.1 (**C**=NH), 135.5 (**C**-Cl), 132.3 (d, *J* = 8.0 Hz, CH), 132.2 (*N*_(1)_-**C_q_**), 130.2 (CH), 129.7 (2xCH), 129.6 (2xCH), 125.0 (d, *J* = 3.0 Hz, CH), 122.7 (d, *J* = 13.0 Hz, *N*_(3)_-**C_q_**), 117.1 (d, *J* = 19.0 Hz, CH) ppm; HRMS (ESI^+^): *m*/*z* [M + H]^+^ calcd for C_15_H_10_N_3_FClS_2_: 349.9989; found: 349.9980. 

3-(2-fluorophenyl)-1-(4-fluorophenyl)-5-iminoimidazolidine-2,4-dithione (**18w**). 

Green solid (81% yield); IR (KBr) 3233 (NH), 1657 (C=N), 1511 (C=S) cm^−1^; ^1^H NMR (CDCl_3_, 400 MHz) δ 9.52 (s, 1H, NH), 7.61–7.45 (m, 3H, Ar-H), 7.44–7.21 (m, 5H, Ar-H) ppm; ^13^C NMR (CDCl_3_, 100 MHz) δ 179.5 (C=S), 179.4 (C=S), 162.7 (d, *J* = 249.0 Hz, C-F), 157.4 (d, *J* = 252.0 Hz, C-F), 156.3 (**C**=NH), 132.3 (d, *J* = 8.0 Hz, 2xCH_p-fluorophenyl_), 130.2 (d, *J* = 3.0 Hz, CH_m-fluorophenyl_), 130.1 (CH_m-fluorophenyl_), 129.7 (d, *J* = 3.0 Hz, *N*_(1)_-**C_q_**), 125.0 (d, *J* = 8.0 Hz, CH_m-fluorophenyl_), 122.8 (d, *J* = 13.0 Hz, *N*_(3)_-**C_q_**), 117.0 (d, *J* = 19.0 Hz, CH_m-fluorophenyl_), 116.6 (d, *J* = 23.0 Hz, 2xCH_p-fluorophenyl_) ppm; HRMS (ESI^+^): *m*/*z* [M + H]^+^ calcd for C_15_H_10_F_2_N_3_S_2_: 334.0284; found: 334.0293.

3-(2-fluorophenyl)-5-imino-1-(4-nitrophenyl)imidazolidine-2,4-dithione (**18x**). 

Brown solid (98% yield); IR (KBr) 3227 (NH), 1663 (C=N), 1596, 1521 (C=S), 1387, 1344, 1309, 1129, 1068 cm^−1^; ^1^H NMR (DMSO-d_6_, 400 MHz) δ 9.60 (s, 1H, NH), 8.43 (d, *J* = 8.0 Hz, 2H, Ar-H_p-nitrophenyl_), 7.78 (d, *J* = 8.0 Hz, 2H, Ar-H_p-nitrophenyl_), 7.61–7.55 (m, 1H, Ar-H_2-fluorophenyl_), 7.44–7.30 (m, 3H, Ar-H_2-fluorophenyl_) ppm; ^13^C NMR (CDCl_3_, 100 MHz) δ 178.9 (C=S), 178.5 (C=S), 157.4 (d, *J* = 253.0 Hz, C-F), 155.6 (**C**=NH), 147.6 (NO_2_-**C_q_**), 139.1 (*N*_(1)_-**C_q_**), 132.5 (d, *J* = 8.0 Hz, CH), 130.1 (CH), 129.5 (2xCH_p-nitrophenyl_), 125.1 (d, *J* = 4.0 Hz, CH), 124.6 (2xCH_p-nitrophenyl_), 122.5 (d, *J* = 13.0 Hz, *N*_(3)_-**C_q_**), 117.1 (d, *J* = 18.0 Hz, CH) ppm; HRMS (ESI^+^): *m*/*z* [M + H]^+^ calcd for C_15_H_10_FN_4_O_2_S_2_: 361.0229; found: 361.0234. 

3-(2-fluorophenyl)-5-imino-1-(4-methoxyphenyl)imidazolidine-2,4-dithione (**18y**). 

Brown solid (78% yield); IR (KBr) 3222 (NH), 1659 (C=N), 1605, 1514 (C=S), 1392, 1299, 1247, 1024 cm^−1^; ^1^H NMR (CDCl_3_, 400 MHz) δ 9.49 (s, 1H, NH), 7.59–7.52 (m, 1H, Ar-H), 7.44–7.27 (m, 5H, Ar-H), 7.07 (d, *J* = 8.0 Hz, 2H, Ar-H_p-methoxyphenyl_), 3.86 (s, 3H, OCH_3_) ppm; ^13^C NMR (CDCl_3_, 100 MHz) δ 179.8 (C=S), 179.7 (C=S), 160.1 (OCH_3_-**C_q_**), 157.4 (d, *J* = 253.0 Hz, C-F), 156.8 (**C**=NH), 132.2 (d, *J* = 8.0 Hz, CH), 130.2 (CH), 129.3 (2xCH_p-methoxyphenyl_), 126.3 (*N*_(1)_-**C_q_**), 124.9 (d, *J* = 4.0 Hz, CH), 122.9 (d, *J* = 13.0 Hz, *N*_(3)_-**C_q_**), 117.0 (d, *J* = 19.0 Hz, CH), 114.8 (2xCH_p-methoxyphenyl_), 55.5 (OCH_3_) ppm; HRMS (ESI^+^): *m*/*z* [M + H]^+^ calcd for C_16_H_13_N_3_FOS_2_: 346.0484; found: 346.0495.

(5-imino-2,4-dithioxo-3-(p-tolyl)imidazolidin-1-yl)(phenyl)methanone (**18z**). 

Dark brown solid (91% yield); IR (KBr) 3228 (NH), 1728 (C=O), 1660 (C=N), 1599, 1513 (C=S), 1398, 1290, 1187, 1025 cm^−1^; ^1^H NMR (CDCl_3_, 400 MHz) δ 9.57 (s, 1H, NH), 8.03 (d, *J* = 8.0 Hz, 2H, Ar-H), 7.72 (t, *J* = 8.0 Hz, 1H, Ar-H), 7.56 (t, *J* = 8.0 Hz, 2H, Ar-H), 7.38 (d, *J* = 8.0 Hz, 2H, Ar-H), 7.24 (d, *J* = 8.0 Hz, 2H, Ar-H), 2.45 (s, 3H, CH_3_) ppm; ^13^C NMR (CDCl_3_, 100 MHz) δ 179.6 (C=S), 178.2 (C=S), 167.0 (C=O), 155.0 (**C**=NH), 140.5 (CH_3_-**C_q_**), 135.6 (CH), 131.6 (*N*_(3)_-**C_q_** and Bz-**C_q_**), 131.2 (2xCH), 130.3 (2xCH_p-tolyl_), 129.2 (2xCH), 127.8 (2xCH_p-tolyl_), 21.4 (CH_3_) ppm; HRMS (ESI^+^): *m*/*z* [M + H]^+^ calcd for C_17_H_14_N_3_OS_2_: 340.0578; found: 340.0589. 

5-imino-1-phenyl-3-(p-tolyl)imidazolidine-2,4-dithione (**18a′**). 

Dark brown solid (87% yield); IR (KBr) 3213 (NH), 1655 (C=N), 1592, 1512 (C=S), 1496, 1414, 1393, 1293, 1124, 1073 cm^−1^; ^1^H NMR (CDCl_3_, 400 MHz) δ 9.49 (s, 1H, NH), 7.61–7.54 (m, 2H, Ar-H), 7.54–7.47 (m, 3H, Ar-H), 7.39 (d, *J* = 8.0 Hz, 2H, Ar-H), 7.28 (d, *J* = 8.0 Hz, 2H, Ar-H), 2.46 (s, CH_3_) ppm; ^13^C NMR (CDCl_3_, 100 MHz) δ 180.6 (C=S), 180.3 (C=S), 156.6 (**C**=NH), 140.4 (CH_3_-**C**), 134.1 (*N*_(1)_-**C_q_**), 132.7 (*N*_(3)_-**C_q_**), 130.3 (2xCH), 129.6 (CH), 129.5 (2xCH), 128.3 (2xCH), 128.0 (2xCH), 21.5 (CH_3_) ppm; HRMS (ESI^+^): *m*/*z* [M + H]^+^ calcd for C_16_H_14_N_3_S_2_: 312.0629; found: 312.0637. 

5-imino-3-(p-tolyl)-1-(4-(trifluoromethyl)phenyl)imidazolidine-2,4-dithione (**18b′**). 

Light brown solid (92% yield); IR (KBr) 3233 (NH), 1656 (C=N), 1613, 1590, 1513 (C=S), 1417, 1390, 1322, 1277, 1126, 1071 cm^−1^; ^1^H NMR (CDCl_3_, 400 MHz) δ 9.53 (s, 1H, NH), 7.84 (d, *J* = 8.0 Hz, 2H, Ar-H_p-(trifluoromethyl)phenyl_), 7.68 (d, *J* = 8.0 Hz, 2H, Ar-H_p-(trifluoromethyl)phenyl_), 7.39 (d, *J* = 8.0 Hz, 2H, Ar-H_p-tolyl_), 7.27 (d, *J* = 8.0 Hz, 2H, Ar-H_p-tolyl_), 2.46 (s, 3H, CH_3_) ppm; ^13^C NMR (CDCl_3_, 100 MHz) δ 180.0 (C=S), 179.8 (C=S), 156.1 (**C**=NH), 140.5 (CH_3_-**C_q_**), 137.0 (*N*_(1)_-**C_q_**), 132.4 (*N*_(3)_-**C_q_**), 130.5 (q, *J* = 33.0 Hz, **C**-CF_3_), 130.3 (2xCH_p-tolyl_), 128.9 (2xCH_p-(trifluoromethyl)phenyl_), 127.9 (2xCH_p-tolyl_), 126.6 (q, *J* = 4.0 Hz, 2xCH_p-(trifluoromethyl)phenyl_), 123.5 (q, *J* = 271.0 Hz, CF_3_), 21.4 (CH_3_) ppm; HRMS (ESI^+^): *m*/*z* [M + H]^+^ calcd for C_17_H_13_N_3_F_3_S_2_: 380.0503; found: 380.0511.

5-imino-1,3-di-p-tolylimidazolidine-2,4-dithione (**18c′**). 

Brown solid (93% yield); IR (KBr) 3225 (NH), 1650 (C=N), 1512 (C=S), 1413, 1387, 1290, 1274, 1148, 1069 cm^−1^; ^1^H NMR (CDCl_3_, 400 MHz) δ 9.48 (s, 1H, NH), 7.38 (d, *J* = 8.0 Hz, 2H, Ar-H), 7.37–7.36 (m, 4H, Ar-H), 7.27 (d, *J* = 8.0 Hz, 2H, Ar-H), 2.45 (s, CH_3_), 2.43 (s, CH_3_) ppm; ^13^C NMR (CDCl_3_, 100 MHz) δ 180.6 (C=S), 180.2 (C=S), 156.6 (**C**=NH), 140.2 (CH_3_-**C**), 139.7 (CH_3_-**C**), 132.6 (*N*_(3)_-**C_q_**), 131.3 (*N*_(1)_-**C_q_**), 130.2 (2xCH), 130.1 (2xCH), 127.9 (2xCH), 127.8 (2xCH), 21.4 (CH_3_), 21.3 (CH_3_) ppm; HRMS (ESI^+^): *m*/*z* [M + H]^+^ calcd for C_17_H_16_N_3_S_2_: 326.0786; found: 326.0775. 

1-(2-fluorophenyl)-5-imino-3-(p-tolyl)imidazolidine-2,4-dithione (**18d′**). 

Brown solid (91% yield); IR (KBr) 3241 (NH), 1660 (C=N), 1592, 1506 (C=S), 1412, 1386, 1301, 1162, 1067cm^−1^; ^1^H NMR (CDCl_3_, 400 MHz) δ 9.48 (s, 1H, NH), 7.57–7.48 (m, 2H, Ar-H), 7.39 (d, *J* = 8.0 Hz, 2H, Ar-H_p-(chlorophenyl)_, 7.36–7.31 (m, 2H, Ar-H), 7.29 (d, *J* = 8.0 Hz, 2H, Ar-H_p-(chlorophenyl)_, 2.46 (s, 3H, CH_3_) ppm; ^13^C NMR (CDCl_3_, 100 MHz) δ 180.2 (C=S), 179.9 (C=S), 157.9 (d, *J* = 253.0 Hz, C-F), 155.7 (**C**=NH), 140.4 (**C_q_**-CH_3_), 132.5 (*N*_(3)_-**C_q_**), 131.8 (d, *J* = 8.0 Hz, CH), 130.4 (CH), 130.2 (2xCH), 127.9 (2xCH), 125.0 (d, *J* = 4.0 Hz, CH), 121.7 (d, *J* = 13.0 Hz, *N*_(1)_-**C_q_**), 117.1 (d, *J* = 19.0 Hz, CH), 21.4 (CH_3_) ppm; HRMS (ESI^+^): *m*/*z* [M + H]^+^ calcd for C_16_H_13_N_3_FS_2_: 330.0535; found: 330.0524. 

1-(4-chlorophenyl)-5-imino-3-(p-tolyl)imidazolidine-2,4-dithione (**18e′**).

Brown solid (91% yield); IR (KBr) 3225 (NH), 1654 (C=N), 1586, 1512 (C=S), 1495, 1413, 1383, 1333, 1271, 1123, 1067 cm^−1^; ^1^H NMR (CDCl_3_, 400 MHz) δ 9.49 (s, 1H, NH), 7.54 (d, *J* = 8.0 Hz, 2H, Ar-H_p-chlorophenyl_), 7.45 (d, *J* = 8.0 Hz, 2H, Ar-H_p-chlorophenyl_), 7.37 (d, *J* = 8.0 Hz, 2H, Ar-H_p-tolyl_), 7.25 (d, *J* = 8.0 Hz, 2H, Ar-H_p-tolyl_), 2.45 (s, 3H, CH_3_) ppm; ^13^C NMR (CDCl_3_, 100 MHz) δ 180.3 (C=S), 180.0 (C=S), 156.2 (**C**=NH), 140.4 (**C_q_**-Me), 135.4 (**C**-Cl), 132.5 (*N*_1_-**C_q_**), 132.4 (*N*_3_-**C_q_**), 130.3 (2xCH_p-tolyl_), 129.7 (2xCH_p-chlorophenyl_), 129.6 (2xCH_p-chlorophenyl_), 127.9 (2xCH_p-tolyl_), 21.4 (CH_3_) ppm; HRMS (ESI^+^): *m*/*z* [M + H]^+^ calcd for C_16_H_13_N_3_ClS_2_: 346.0239; found: 346.0244. 

1-(4-fluorophenyl)-5-imino-3-(p-tolyl)imidazolidine-2,4-dithione (**18f′**). 

Light brown solid (61% yield); IR (KBr) 3234 (NH), 1656 (C=N), 1510 (C=S), 1383, 1295, 1149, 1067 cm^−1^; ^1^H NMR (CDCl_3_, 400 MHz) δ 9.50 (s, 1H, NH), 7.52–7.45 (m, 2H, Ar-H), 7.39 (d, *J* = 8.0 Hz, 2H, Ar-H_tolyl_), 7.30–7.21 (m, 4H, Ar-H), 2.46 (s, 3H, Ar- CH_3_) ppm; ^13^C NMR (CDCl_3_, 100 MHz) δ 180.5 (C=S), 180.0 (C=S), 162.6 (d, *J* = 248.0 Hz, C-F), 156.4 (**C**=NH), 140.3 (C**_q_**-Me), 132.5 (*N*_(3)_-**C_q_**), 130.2 (2xCH_p-tolyl_), 130.2 (d, *J* = 9.0 Hz, 2xCH_p-fluorophenyl_), 129.8 (d, *J* = 3.0 Hz, *N*_(1)_-**C_q_**), 127.8 (2xCH_p-tolyl_), 116.5 (d, *J* = 23.0 Hz, 2xCH_p-fluorophenyl_), 21.4 (CH_3_) ppm; HRMS (ESI^+^): *m*/*z* [M + H]^+^ calcd for C_16_H_13_FN_3_S_2_: 330.0535; found: 330.0523.

5-imino-1-(4-nitrophenyl)-3-(p-tolyl)imidazolidine-2,4-dithione (**18g′**). 

Brown solid (81% yield); IR (KBr) 3229 (NH), 1661 (C=N), 1595, 1522 (C=S), 1382, 1348, 1298, 1157, 1072 cm^−1^; ^1^H NMR (CDCl_3_, 400 MHz) δ 9.57 (s, 1H, NH), 8.43 (d, *J* = 8.0 Hz, 2H, Ar-H_p-nitrophenyl_), 7.77 (d, *J* = 8.0 Hz, 2H, Ar-H_p-nitrophenyl_), 7.39 (d, *J* = 8.0 Hz, 2H, Ar-H_p-tolyl_), 7.26 (d, *J* = 8.0 Hz, 2H, Ar-H_p-tolyl_), 2.46 (s, 3H, CH_3_) ppm; ^13^C NMR (CDCl_3_, 100 MHz) δ 179.6 (C=S), 179.5 (C=S), 155.7 (**C**=NH), 147.6 (NO_2_-**C_q_**), 140.6 (CH_3_-**C_q_**), 139.3 (*N*_(1)_-**C_q_**), 132.2 (*N*_(3)_-**C_q_**), 130.3 (2xCH_p-tolyl_), 129.5 (2xCH_p-nitrophenyl_), 127.8 (2xCH_p-tolyl_), 124.6 (2xCH_p-nitrophenyl_), 21.4 (CH_3_) ppm; HRMS (ESI^+^): *m*/*z* [M + H]^+^ calcd for C_16_H_13_N_4_O_2_S_2_: 357.0480; found: 357.0471. 

5-imino-1-(4-methoxyphenyl)-3-(p-tolyl)imidazolidine-2,4-dithione (**18h′**). 

Brown solid (66% yield); IR (KBr) 3228 (NH), 1655 (C=N), 1608, 1513 (C=S), 1388, 1291, 1250, 1150, 1029 cm^−1^; ^1^H NMR (CDCl_3_, 400 MHz) δ 9.46 (s, 1H, NH), 7.40 (d, *J* = 8.0 Hz, 2H, Ar-H_p-methoxyphenyl_), 7.38 (d, *J* = 8.0 Hz, 2H, Ar-H_p-tolyl_), 7.27 (d, *J* = 8.0 Hz, 2H, Ar-H_p-tolyl_), 7.06 (d, *J* = 8.0 Hz, 2H, Ar-H_p-methoxyphenyl_), 3.86 (s, 3H, OCH_3_), 2.45 (s, 3H, ArCH_3_) ppm; ^13^C NMR (CDCl_3_, 100 MHz) δ 180.8 (C=S), 180.2 (C=S), 160.0 (OCH_3_-**C_q_**), 156.7(**C**=NH), 140.2 (**C**-Me), 132.5 (*N*_(3)_-**C_q_**), 130.1 (2xCH_p-tolyl_), 129.3 (2xCH_p-methoxyphenyl_), 127.8 (2xCH_p-tolyl_), 126.3 (*N*_(1)_-**C_q_**), 114.7 (2xCH_p-methoxyphenyl_), 55.4 (OCH_3_), 21.4 (ArCH_3_) ppm; HRMS (ESI^+^): *m*/*z* [M + H]^+^ calcd for C_17_H_16_N_3_OS_2_: 342.0735; found: 342.0727.

(5-imino-3-(4-methoxyphenyl)-2,4-dithioxoimidazolidin-1-yl)(phenyl)methanone (**18i′**). 

Brown solid (86% yield); IR (KBr) 3225 (NH), 1726 (C=O), 1661 (C=N), 1599, 1514 (C=S), 1402, 1290, 1168, 1027 cm^−1^; ^1^H NMR (CDCl_3_, 400 MHz) δ 9.56 (s, 1H, NH), 8.02 (d, *J* = 8.0 Hz, 2H, Ar-H), 7.72 (t, *J* = 8.0 Hz, 1H, Ar-H), 7.55 (d, *J* = 8.0 Hz, 2H, Ar-H), 7.28 (d, *J* = 8.0 Hz, 2H, Ar-H_p-methoxyphenyl_), 7.06 (d, *J* = 8.0 Hz, 2H, Ar-H_p-methoxyphenyl_), 3.87 (s, 3H, OCH_3_) ppm; ^13^C NMR (CDCl_3_, 100 MHz) δ 179.9 (C=S), 178.3 (C=S), 167.1 (C=O), 160.5 (OCH_3_-**C_q_**), 155.0 (**C**=NH), 135.6 (CH), 131.2 (2xCH), 130.4 (O=C-**C_q_**), 129.3 (2xCH_p-methoxyphenyl_), 129.2 (2xCH), 126.6 (*N*_(3)_-**C_q_**), 114.8 (2xCH_p-methoxyphenyl_), 55.5 (OCH_3_) ppm; HRMS (ESI^+^): *m*/*z* [M + H]^+^ calcd for C_17_H_14_N_3_O_2_S_2_: 356.0527; found: 356.0539.

5-imino-3-(4-methoxyphenyl)-1-phenylimidazolidine-2,4-dithione (**18j′**). 

Brown solid (92% yield); IR (KBr) 3214 (NH), 1653 (C=N), 1606, 1590, 1513 (C=S), 1499, 1393, 1302, 1280, 1248, 1129, 1078 cm^−1^; ^1^H NMR (CDCl_3_, 400 MHz) δ 9.49 (s, 1H, NH), 7.61–7.54 (m, 2H, Ar-H_ph_), 7.53–7.48 (m, 3H, Ar-H_ph_), 7.31 (d, *J* = 8.0 Hz, 2H, Ar-H_p-methoxyphenyl_), 7.07 (d, *J* = 8.0 Hz, 2H, Ar-H_p-methoxyphenyl_), 3.87 (s, 3H, OCH_3_) ppm; ^13^C NMR (CDCl_3_, 100 MHz) δ 180.7 (C=S), 180.3 (C=S), 160.3 (OCH_3_-**C_q_**), 156.5 (**C**=NH), 134.0 (*N*_(1)_-**C_q_**), 129.5 (CH_ph_), 129.5 (2xCH_p-methoxyphenyl_), 129.3 (2xCH_ph_), 128.2 (2xCH_ph_), 127.6 (*N*_(3)_-**C_q_**), 114.7 (2xCH_p-methoxyphenyl_), 55.4 (OCH_3_) ppm; HRMS (ESI^+^): *m*/*z* [M + H]^+^ calcd for C_16_H_14_N_3_OS_2_: 328.0578; found: 328.0583.

5-imino-3-(4-methoxyphenyl)-1-(4-(trifluoromethyl)phenyl)imidazolidine-2,4-dithione (**18k′**). 

Light brown solid (94% yield); IR (KBr) 3247 (NH), 1659 (C=N), 1612, 1590, 1512 (C=S), 1414, 1387, 1328, 1277, 1255, 1172, 1074 cm^−1^; ^1^H NMR (CDCl_3_, 400 MHz) δ 9.53 (s, 1H, NH), 7.83 (d, *J* = 8.0 Hz, 2H, Ar-H_p-(trifluoromethyl)phenyl_), 7.67 (d, *J* = 8.0 Hz, 2H, Ar-H_p-(trifluoromethyl)phenyl_), 7.30 (d, *J* = 8.0 Hz, 2H, Ar-H_p-methoxyphenyl_), 7.07 (d, *J* = 8.0 Hz, 2H, Ar-H_p-methoxyphenyl_), 3.88 (s, 3H, OCH_3_) ppm; ^13^C NMR (CDCl_3_, 100 MHz) δ 180.2 (C=S), 179.9 (C=S), 160.5 (OCH_3_-**C_q_**), 156.1 (**C**=NH), 137.1 (*N*_(1)_-**C_q_**), 131.3 (q, *J* = 33.0 Hz, **C**-CF_3_), 129.3 (2xCH_p-methoxyphenyl_), 128.9 (2xCH_p-(trifluoromethyl)phenyl_), 127.4 (*N*_(3)_-**C_q_**), 126.6 (q, *J* = 4.0 Hz, 2xCH_p-(trifluoromethyl)phenyl_), 123.5 (q, *J* = 271.0 Hz, CF_3_), 114.5 (2xCH_p-methoxyphenyl_), 55.5 (OCH_3_) ppm; HRMS (ESI^+^): *m*/*z* [M + H]^+^ calcd for C_17_H_13_N_3_F_3_OS_2_: 396.0452; found: 396.0461.

5-imino-3-(4-methoxyphenyl)-1-(p-tolyl)imidazolidine-2,4-dithione (**18l′**).

Reddish brown solid (89% yield); IR (KBr) 3235 (NH), 1652 (C=N), 1589, 1510 (C=S), 1390, 1289, 1247, 1156, 1068 cm^−1^; ^1^H NMR (CDCl_3_, 400 MHz) δ 9.48 (s, 1H, NH), 7.40–7.35 (m, 4H, Ar-H_p-tolyl_), 7.31 (d, *J* = 8.0 Hz, 2H, Ar-H_p-methoxyphenyl_), 7.07 (d, *J* = 8.0 Hz, 2H, Ar-H_p-methoxyphenyl_), 3.87 (s, 3H, OCH_3_), 2.43 (s, 3H, ArCH_3_) ppm; ^13^C NMR (CDCl_3_, 100 MHz) δ 180.9 (C=S), 180.4 (C=S), 160.4 (OCH_3_-**C_q_**), 156.6 (**C**=NH), 139.7 (**C**-Me), 131.4 (*N*_(1)_-**C_q_**), 130.2 (2xCH_p-tolyl_), 129.4 (2xCH_p-methoxyphenyl_), 127.9 (2xCH_p-tolyl_), 127.7 (*N*_(3)_-**C_q_**), 114.7 (2xCH_p-methoxyphenyl_), 55.5 (OCH_3_), 21.4 (ArCH_3_) ppm; HRMS (ESI^+^): *m*/*z* [M + H]^+^ calcd for C_17_H_16_N_3_OS_2_: 342.0735; found: 342.0723.

1-(2-fluorophenyl)-5-imino-3-(4-methoxyphenyl)imidazolidine-2,4-dithione (**18m′**). 

Reddish brown solid (84% yield); IR (KBr) 3245 (NH), 1656 (C=N), 1607, 1590, 1514 (C=S), 1412, 1389, 1310, 1253, 1161, 1067 cm^−1^; ^1^H NMR (CDCl_3_, 400 MHz) δ 9.47 (s, 1H, NH), 7.57–7.47 (m, 2H, Ar-H), 7.38–7.28 (m, 4H, Ar-H), 7.07 (d, *J* = 8.0 Hz, 2H, Ar-H_p-methoxyphenyl_), 3.87 (s, 3H, OCH_3_) ppm; ^13^C NMR (CDCl_3_, 100 MHz) δ 180.3 (C=S), 180.1 (C=S), 160.4 (OCH_3_-**C_q_**), 157.8 (d, *J* = 252.0 Hz, C-F), 155.7 (**C**=NH), 131.8 (d, *J* = 7.0 Hz, CH), 130.4 (CH), 129.3 (2xCH_p-methoxyphenyl_), 127.5 (*N*_(3)_-**C_q_**), 125.0 (d, *J* = 4.0 Hz, CH), 121.7 (d, *J* = 13.0 Hz, *N*_(1)_-**C_q_**), 117.0 (d, *J* = 19.0 Hz, CH), 114.7 (2xCH_p-methoxyphenyl_), 55.5 (OCH_3_) ppm; HRMS (ESI^+^): *m*/*z* [M + H]^+^ calcd for C_16_H_13_N_3_FOS_2_: 346.0484; found: 346.0474.

1-(4-chlorophenyl)-5-imino-3-(4-methoxyphenyl)imidazolidine-2,4-dithione (**18n′**). 

Brown solid (95% yield); IR (KBr) 3205 (NH), 1655 (C=N), 1590, 1513 (C=S), 1496, 1382, 1280, 1252, 1148, 1069 cm^−1^; ^1^H NMR (CDCl_3_, 400 MHz) δ 9.49 (s, 1H, NH), 7.53 (d, *J* = 8.0 Hz, 2H, Ar-H_p-chlorophenyl_), 7.45 (d, *J* = 8.0 Hz, 2H, Ar-H_p-chlorophenyl_), 7.29 (d, *J* = 8.0 Hz, 2H, Ar-H_p-methoxyphenyl_), 7.06 (d, *J* = 8.0 Hz, 2H, Ar-H_p-methoxyphenyl_), 3.87 (s, 3H, OCH_3_) ppm; ^13^C NMR (CDCl_3_, 100 MHz) δ 180.4 (C=S), 180.1 (C=S), 160.4 (OCH_3_-**C_q_**), 156.2 (**C**=NH), 135.4 (**C**-Cl), 132.3 (*N*_(1)_-**C_q_**), 129.7 (2xCH_p-chlorophenyl_), 129.6 (2xCH_p-chlorophenyl_), 129.3 (2xCH_p-methoxyphenyl_), 127.5 (*N*_(3)_-**C_q_**), 114.7 (2xCH_p-methoxyphenyl_), 55.5 (OCH_3_) ppm; HRMS (ESI^+^): *m*/*z* [M + H]^+^ calcd for C_16_H_13_N_3_ClOS_2_: 360.0189; found: 362.0174.

1-(4-fluorophenyl)-5-imino-3-(4-methoxyphenyl)imidazolidine-2,4-dithione (**18o′**). 

Brown solid (88% yield); IR (KBr) 3234 (NH), 1655 (C=N), 1607, 1514 (C=S), 1387, 1284, 1153, 1068 cm^−1^; ^1^H NMR (CDCl_3_, 400 MHz) δ 9.49 (s, 1H, NH), 7.51–7.43 (m, 2H, Ar-H), 7.32–7.22 (m, 4H, Ar-H), 7.07 (d, *J* = 8.0 Hz, 2H, Ar-H_p-methoxyphenyl_), 3.87 (s, 3H, OMe) ppm; ^13^C NMR (CDCl_3_, 100 MHz) δ 180.7 (C=S), 180.2 (C=S), 162.7 (d, *J* = 249.0 Hz, C-F), 160.4 (**C**-O), 156.4 (**C**=NH), 130.2 (d, *J* = 9.0 Hz, 2xCH_p-fluorophenyl_), 129.9 (d, *J* = 3.0 Hz, *N*_(1)_-**C_q_**), 129.3 (2xCH_p-methoxyphenyl_), 127.6 (*N*_(3)_-**C_q_**), 116.6 (d, *J* = 23.0 Hz, 2xCH_p-fluorophenyl_), 114.7 (2xCH_p-methoxyphenyl_), 55.5 (OCH_3_) ppm; HRMS (ESI^+^): *m*/*z* [M + H]^+^ calcd for C_16_H_13_FN_3_OS_2_: 346.0484; found: 346.0491.

5-imino-3-(4-methoxyphenyl)-1-(4-nitrophenyl)imidazolidine-2,4-dithione (**18p′**). 

Brown solid (94% yield); IR (KBr) 3237 (NH), 1659 (C=N), 1595, 1523 (C=S), 1391, 1349, 1296, 1157, 1069 cm^−1^; ^1^H NMR (CDCl_3_, 400 MHz) δ 9.57 (s, 1H, NH), 8.42 (d, *J* = 8.0 Hz, 2H, Ar-H_p-nitrophenyl_), 7.77 (d, *J* = 8.0 Hz, 2H, Ar-H_p-nitrophenyl_), 7.29 (d, *J* = 8.0 Hz, 2H, Ar-H_p-methoxyphenyl_), 7.07 (d, *J* = 8.0 Hz, 2H, Ar-H_p-methoxyphenyl_), 3.88 (s, 3H, OCH_3_) ppm; ^13^C NMR (CDCl_3_, 100 MHz) δ 179.8 (C=S), 179.7 (C=S), 160.5 (OCH_3_-**C_q_**), 155.7 (**C**=NH), 147.6 (NO_2_-**C_q_**), 139.3 (*N*_(1)_-**C_q_**), 129.5 (2xCH_p-nitrophenyl_), 129.3 (2xCH_p-methoxyphenyl_), 127.3 (*N*_(3)_-**C_q_**), 124.6 (2xCH_p-nitrophenyl_), 114.8 (2xCH_p-methoxyphenyl_), 55.5 (OCH_3_) ppm; HRMS (ESI^+^): *m*/*z* [M + H]^+^ calcd for C_16_H_13_N_4_O_3_S_2_: 373.0429; found: 373.0434. 

5-imino-1,3-bis(4-methoxyphenyl)imidazolidine-2,4-dithione (**18q′**). 

Brown solid (51% yield); IR (KBr) 3225 (NH), 1652 (C=N), 1608, 1513 (C=S), 1391, 1285, 1254, 1151, 1025 cm^−1^; ^1^H NMR (CDCl_3_, 400 MHz) δ 9.46 (s, 1H, NH), 7.40 (d, *J* = 8.0 Hz, 2H, Ar-H_p-methoxyphenyl_), 7.30 (d, *J* = 8.0 Hz, 2H, Ar-H_p-methoxyphenyl_), 7.08–7.03 (m, 4H, Ar-H_p-methoxyphenyl_), 3.87 (s, 3H, OCH_3_), 3.86 (s, 3H, OCH_3_) ppm; ^13^C NMR (CDCl_3_, 100 MHz) δ 180.9 (C=S), 180.3 (C=S), 160.2 (OCH_3_-**C_q_**), 159.9 (OCH_3_-**C_q_**), 156.6 (**C**=NH), 129.3 (2xCH_p-chlorophenyl_), 129.2 (2xCH_p-chlorophenyl_), 127.6 (*N*_(1)_-**C_q_**), 126.3 (*N*_(3)_-**C_q_**), 114.6 (2xCH_p-methoxyphenyl_), 114.5 (2xCH_p-methoxyphenyl_), 55.4 (OCH_3_), 55.3 (OCH_3_) ppm; HRMS (ESI^+^): *m*/*z* [M + H]^+^ calcd for C_17_H_16_N_3_O_2_S_2_: 358.0684; found: 358.0694.

(5-imino-2,4-dithioxo-3-(4-(trifluoromethyl)phenyl)imidazolidin-1-yl)(phenyl)methanone (**18r′**). 

Brown solid (93% yield); IR (KBr) 3272 (NH), 1633 (C=N), 1605 (C=N), 1507, cm^−1^; ^1^H NMR (CDCl_3_, 400 MHz) δ 9.61 (s, 1H, NH), 8.03 (d, *J* = 8.0 Hz, 2H, Ar-H_Bz_), 7.85 (d, *J* = 8.0 Hz, 2H, Ar-H_trifluoromethyl)phenyl_), 7.74 (t, *J* = 8.0 Hz, 1H, Ar-H_Bz_), 7.58 (d, *J* = 8.0 Hz, 2H, Ar-H_trifluoromethyl)phenyl_), 7.53 (d, *J* = 8.0 Hz, 2H, Ar-H_Bz_) ppm; ^13^C NMR (CDCl_3_, 100 MHz) δ 179.2 (C=S), 177.4 (C=S), 166.9 (C=O), 154.8 (**C**=NH), 137.2 (*N*_(3)_-**C_q_**), 135.8 (CH), 132.2 (q, *J* = 33.0 Hz, **C**-CF_3_), 131.2 (2xCH), 131.0 (O=C-**C_q_**), 129.3 (2xCH), 129.31 (2xCH), 126.8 (q, *J* = 3.0 Hz, 2xCH), 123.4 (q, *J* = 271.0 Hz, CF_3_) ppm; HRMS (ESI^+^): *m*/*z* [M + H]^+^ calcd for C_17_H_11_F_3_N_3_OS_2_: 394.0296; found: 394.0285. 

5-imino-1-phenyl-3-(4-(trifluoromethyl)phenyl)imidazolidine-2,4-dithione (**18s′**). 

Brownish oil (53% yield); IR (KBr) 3469 (NH), 1613 (C=N), 1397, 1325, 1170, 1065 cm^−1^; ^1^H NMR (CDCl_3_, 400 MHz) δ 9.52 (s, 1H, NH), 7.85 (d, *J* = 8.0 Hz, 2H, Ar-H_p-(trifluoromethyl)phenyl_), 7.61–7.46 (m, 7H, Ar-H) ppm; ^13^C NMR (CDCl_3_, 100 MHz) δ 179.8 (C=S), 179.7 (C=S), 156.3 (**C**=NH), 138.0 (*N*_(3)_-**C_q_**), 133.7 (*N*_(1)_-**C_q_**), 132.0 (q, *J* = 32.0 Hz, **C**-CF_3_), 129.7 (CH), 129.6 (2xCH), 129.1 (2xCH), 128.2 (2xCH), 126.7 (q, *J* = 3.0 Hz, 2xCH), 123.4 (q, *J* = 272.0 Hz, CF_3_) ppm; HRMS (ESI^+^): *m*/*z* [M + H]^+^ calcd for C_16_H_11_N_3_F_3_S_2_: 366.0347; found: 366.0356.

5-imino-1-(p-tolyl)-3-(4-(trifluoromethyl)phenyl)imidazolidine-2,4-dithione (**18t′**). 

Brownish oil (65% yield); IR (KBr) 3273 (NH), 1658 (C=N), 1613 (C=N), 1514 (C=S), 1393, 1325, 1169, 1055 cm^−1^; ^1^H NMR (CDCl_3_, 400 MHz) δ 9.49 (s, 1H, NH), 7.84 (d, *J* = 8.0 Hz, 2H, Ar-H_p-(trifluoromethyl)phenyl_), 7.56 (d, *J* = 8.0 Hz, 2H, Ar-H_p-(trifluoromethyl)phenyl_), 7.39–7.30 (m, 4H, Ar-H_p-tolyl_), 2.41 (s, 3H, CH_3_) ppm; ^13^C NMR (CDCl_3_, 100 MHz) δ 179.9 (C=S), 179.8 (C=S), 156.5 (**C**=NH), 140.1 (CH_3_-**C_q_**), 138.0 (*N*_(3)_-**C_q_**), 131.9 (q, *J* = 33.0 Hz, **C**-CF_3_), 131.0 (*N*_(1)_-**C_q_**), 130.3 (2xCH_p-tolyl_), 129.1 (2xCH_p-(trifluoromethyl)phenyl_), 127.9 (2xCH_p-tolyl_), 126.6 (q, *J* = 4.0 Hz, 2xCH_p-(trifluoromethyl)phenyl_), 123.4 (q, *J* = 271.0 Hz, CF_3_), 21.3 (CH_3_) ppm; HRMS (ESI^+^): *m*/*z* [M + H]^+^ calcd for C_17_H_13_N_3_F_3_S_2_: 380.0503; found: 380.0509.

1-(2-fluorophenyl)-5-imino-3-(4-(trifluoromethyl)phenyl)imidazolidine-2,4-dithione (**18u′**). 

Brownish oil (71% yield); IR (KBr) 3427 (NH), 1678 (C=N), 1614, 1507, 1393, 1326, 1170, 1065 cm^−1^; ^1^H NMR (CDCl_3_, 400 MHz) δ 9.52 (s, 1H, NH), 7.85 (d, *J* = 8.0 Hz, 2H, Ar-H_p-(trifluoromethyl)phenyl_), 7.57 (d, *J* = 8.0 Hz, 2H, Ar-H_p-(trifluoromethyl)phenyl_), 7.87–7.79 (m, 1H, Ar-H_2-fluorophenyl_), 7.38–7.27 (m, 3H, Ar-H_2-fluorophenyl_) ppm; ^13^C NMR (CDCl_3_, 100 MHz) δ 179.8 (C=S), 179.1 (C=S), 157.8 (d, *J* = 252.0 Hz, C-F), 155.5 (**C**=NH), 137.7 (*N*_(1)_-**C_q_**), 132.0 (d, *J* = 8.0 Hz, CH_2-fluorophenyl_), 132.0 (q, *J* = 33.0 Hz, **C**-CF_3_), 130.3 (CH_2-fluorophenyl_), 129.1 (2xCH_p-(trifluoromethyl)phenyl_), 126.7 (q, *J* = 4.0 Hz, 2xCH _p-(trifluoromethyl)phenyl_), 125.0 (d, *J* = 4.0 Hz, CH_2-fluorophenyl_), 123.4 (q, *J* = 271.0 Hz, CF_3_), 121.4 (d, *J* = 13.0 Hz, *N*_(3)_-**C_q_**), 117.1 (d, *J* = 19.0 Hz, CH_2-fluorophenyl_) ppm; HRMS (ESI^+^): *m*/*z* [M + H]^+^ calcd for C_16_H_10_N_3_F_4_S_2_: 384.0252; found: 384.0243.

1-(4-chlorophenyl)-5-imino-3-(4-(trifluoromethyl)phenyl)imidazolidine-2,4-dithione (**18v′**). 

Brownish oil (82% yield); IR (KBr) 3437 (NH), 1659 (C=N), 1493, 1387, 1324, 1168, 1065 cm^−1^; ^1^H NMR (CDCl_3_, 400 MHz) δ 9.53 (s, 1H, NH), 7.85 (d, *J* = 8.0 Hz, 2H, Ar-H_p-(trifluoromethyl)phenyl_), 7.55 (d, *J* = 8.0 Hz, 2H, Ar-H_p-(trifluoromethyl)phenyl_), 7.52 (d, *J* = 8.0 Hz, 2H, Ar-H_p-chlorophenyl_), 7.44 (d, *J* = 8.0 Hz, 2H, Ar-H_p-chlorophenyl_) ppm; ^13^C NMR (CDCl_3_, 100 MHz) δ 179.6 (C=S), 179.4 (C=S), 156.0 (**C**=NH), 137.9 (**C**-Cl), 135.7 (*N*_(3)_-**C_q_**), 132.0 (*N*_(1)_-**C_q_**), 132.0 (q, *J* = 33.0 Hz, **C**-CF_3_), 129.8 (2xCH_p-chlorophenyl_), 129.6 (2xCH_p-chlorophenyl_), 129.1 (2xCH_p-(trifluoromethyl)phenyl_), 126.7 (q, *J* = 4.0 Hz, 2xCH _p-(trifluoromethyl)phenyl_), 123.4 (q, *J* = 271.0 Hz, CF_3_) ppm; HRMS (ESI^+^): *m*/*z* [M + H]^+^ calcd for C_16_H_10_ClN_3_F_3_S_2_: 399.9957; found: 399.9952.

5-imino-1-(4-nitrophenyl)-3-(4-(trifluoromethyl)phenyl)imidazolidine-2,4-dithione (**18w′**). 

Brown solid (76% yield); IR (KBr) 3232 (NH), 1662 (C=N), 1595, 1521 (C=S), 1388, 1326, 1171, 1065 cm^−1^; ^1^H NMR (CDCl_3_, 400 MHz) δ 9.63 (s, 1H, NH), 8.43 (d, *J* = 8.0 Hz, 2H, Ar-H_p-nitrophenyl_), 7.86 (d, *J* = 8.0 Hz, 2H, Ar-H_trifluoromethyl)phenyl_), 7.77 (d, *J* = 8.0 Hz, 2H, Ar-H_p-nitrophenyl_), 7.55 (d, *J* = 8.0 Hz, 2H, Ar-H_trifluoromethyl)phenyl_) ppm; ^13^C NMR (CDCl_3_, 100 MHz) δ 179.1 (C=S), 178.9 (C=S), 155.5 (**C**=NH), 147.7 (NO_2_-**C_q_**), 139.0 (*N*_(1)_-**C_q_**), 137.8 (*N*_(3)_-**C_q_**), 132.2 (q, *J* = 33.0 Hz, **C**-CF_3_), 129.5 (2xCH_p-nitrophenyl_), 129.1 (2xCH), 126.8 (q, *J* = 4.0 Hz, 2xCH), 124.6 (2xCH_p-nitrophenyl_), 123.4 (q, *J* = 272.0 Hz, CF_3_) ppm; HRMS (ESI^+^): *m*/*z* [M + H]^+^ calcd for C_16_H_10_F_3_N_4_O_2_S_2_: 411.0197; found: 411.0188. 

5-imino-1,3-bis(4-nitrophenyl)imidazolidine-2,4-dithione (**18x′**). 

Dark green solid (31% yield); IR (KBr) 3435 (NH), 1660 (C=N), 1594, 1522 (C=S), 1495, 1387, 1348, 1287 cm^−1^; ^1^H NMR (DMSO-d_6_, 400 MHz) δ 10.22 (s, 1H, NH), 8.47 (d, *J* = 8.0 Hz, 2H, Ar-H), 8.46 (d, *J* = 8.0 Hz, 2H, Ar-H), 7.85 (d, *J* = 8.0 Hz, 2H, Ar-H), 7.81 (d, *J* = 8.0 Hz, 2H, Ar-H), ppm; ^13^C NMR (DMSO-d_6_, 100 MHz) δ 180.7 (C=S), 180.1 (C=S), 155.4 (**C**=NH), 148.0 (NO_2_-**C_q_**), 147.3 (NO_2_-**C_q_**), 141.0 (*N*_(1)_-**C_q_**), 139.9 (*N*_(3)_-**C_q_**), 130.5 (2xCH), 130.3 (2xCH), 129.6 (2xCH), 124.7 (2xCH), 124.4 (2xCH) ppm; HRMS (ESI^+^): *m*/*z* [M + H]^+^ calcd for C_15_H_10_N_5_O_4_S_2_: 388.0174; found: 388.0163. 

(3-(4-fluorophenyl)-5-imino-2,4-dithioxoimidazolidin-1-yl)(phenyl)methanone (**18y′**). 

Burgundy solid (83% yield); IR (KBr) 3226 (NH), 1726 (C=O), 1661 (C=N), 1600 (C=N), 1512 (C=S), 1400, 1296, 1239, 1050 cm^−1^; ^1^H NMR (CDCl_3_, 400 MHz) δ 9.58 (s, 1H, NH), 8.02 (d, *J* = 8.0 Hz, 2H, Ar-H), 7.73 (t, *J* = 8.0 Hz, 1H, Ar-H), 7.56 (t, *J* = 8.0 Hz, 2H, Ar-H), 7.38–7.33 (m, 2H, Ar-H_p-fluorophenyl_), 7.30–7.23 (m, 2H, Ar-H_p-fluorophenyl_) ppm; ^13^C NMR (CDCl_3_, 100 MHz) δ 179.6 (C=S), 177.9 (C=S), 167.0 (C=O), 163.1 (d, *J* = 250.0 Hz, C-F), 154.9 (**C**=NH), 135.7 (CH), 131.2 (2xCH), 131.1 (Ph-**C_q_**), 130.3 (d, *J* = 9.0 Hz, 2xCH_p-fluorophenyl_), 130.0 (d, *J* = 3.0 Hz, *N*_(3)_-**C_q_**), 129.2 (2xCH), 116.8 (d, *J* = 23.0 Hz, 2xCH_p-fluorophenyl_) ppm; HRMS (ESI^+^): *m*/*z* [M + H]^+^ calcd for C_16_H_11_FN_3_OS_2_: 344.0328; found: 344.0338.

3-(4-fluorophenyl)-5-imino-1-phenylimidazolidine-2,4-dithione (**18z′**). 

Brown solid (82% yield); IR (KBr) 3243 (NH), 1654 (C=N), 1595, 1509 (C=S), 1417, 1394, 1286, 1230, 1159, 1068 cm^−1^; ^1^H NMR (CDCl_3_, 400 MHz) δ 9.52 (s, 1H, NH), 7.61–7.47 (m, 5H, Ar-H_phenyl_), 7.41–7.36 (m, 2H, Ar-H_p-fluorophenyl_), 7.29–7.22 (m, 2H, Ar-H_p-fluorophenyl_) ppm; ^13^C NMR (CDCl_3_, 100 MHz) δ 180.3 (C=S), 180.1 (C=S), 163.0 (d, *J* = 249.0 Hz, C-F), 156.4 (**C**=NH), 133.9 (*N*_(1)_-**C_q_**), 131.0 (d, *J* = 4.0 Hz, *N*_(3)_-**C_q_**), 130.3 (d, *J* = 9.0 Hz, 2xCH_p-fluorophenyl_), 129.6 (CH), 129.5 (2xCH), 128.2 (2xCH), 116.7 (d, *J* = 23.0 Hz, 2xCH_p-fluorophenyl_) ppm; HRMS (ESI^+^): *m*/*z* [M + H]^+^ calcd for C_15_H_11_FN_3_OS_2_: 316.0378; found: 316.0393.

3-(4-fluorophenyl)-5-imino-1-(4-(trifluoromethyl)phenyl)imidazolidine-2,4-dithione (**18a″**) 

Brown solid (81% yield); IR (KBr) 3225 (NH), 1661 (C=N), 1611, 1511 (C=S), 1416, 1325, 1234, 1172, 1064 cm^−1^; ^1^H NMR (CDCl_3_, 400 MHz) δ 9.55 (s, 1H, NH), 7.84 (d, *J* = 8.0 Hz, 2H, Ar-H_trifluoromethyl)phenyl_), 7.67 (d, *J* = 8.0 Hz, 2H, Ar-H_trifluoromethyl)phenyl_), 7.40–7.35 (m, 2H, Ar-H_p-fluorophenyl_), 7.30–7.24 (m, 2H, Ar-H_p-fluorophenyl_) ppm; ^13^C NMR (CDCl_3_, 100 MHz) δ 179.8 (C=S), 179.9 (C=S), 163.1 (d, *J* = 250.0 Hz, C-F), 155.9 (**C**=NH), 136.9 (*N*_(1)_-**C_q_**), 131.4 (q, *J* = 33.0 Hz, **C**-CF_3_), 130.8 (d, *J* = 3.0 Hz, *N*_(3)_-**C_q_**), 130.3 (d, *J* = 9.0 Hz, 2xCH_p-fluorophenyl_), 128.8 (2xCH_trifluoromethyl)phenyl_), 126.6 (q, *J* = 3.0 Hz, 2xCH_trifluoromethyl)phenyl_), 123.5 (q, *J* = 271.0 Hz, CF_3_), 116.8 (d, *J* = 23.0 Hz, 2xCH_p-fluorophenyl_) ppm; HRMS (ESI^+^): *m*/*z* [M + H]^+^ calcd for C_16_H_10_F_4_N_3_S_2_: 384.0252; found: 384.0261.

3-(4-fluorophenyl)-5-imino-1-(p-tolyl)imidazolidine-2,4-dithione (**18b″**)

Brown solid (61% yield); IR (KBr) 3220 (NH), 1654 (C=N), 1598, 1510 (C=S), 1390, 1300, 1154, 1067 cm^−1^; ^1^H NMR (CDCl_3_, 400 MHz) δ 9.50 (s, 1H, NH), 7.42–7.34 (m, 6H, Ar-H), 7.29–7.22 (m, 2H, Ar-H_p-fluorophenyl_) ppm; ^13^C NMR (CDCl_3_, 100 MHz) δ 180.4 (C=S), 180.2 (C=S), 162.9 (d, *J* = 249.0 Hz, C-F), 156.5 (**C**=NH), 139.8 (C**_q_**-Me), 131.2 (*N*_(1)_-**C_q_**), 131.0 (d, *J* = 4.0 Hz, *N*_(3)_-**C_q_**), 130.3 (d, *J* = 9.0 Hz, 2xCH_p-fluorophenyl_), 130.2 (2xCH_p-tolyl_), 127.8 (2xCH_p-tolyl_), 116.7 (d, *J* = 23.0 Hz, 2xCH_p-fluorophenyl_), 21.3 (CH_3_) ppm; HRMS (ESI^+^): *m*/*z* [M + H]^+^ calcd for C_16_H_13_FN_3_S_2_: 330.0535; found: 330.0529.

1-(4-chlorophenyl)-3-(4-fluorophenyl)-5-iminoimidazolidine-2,4-dithione (**18c″**) 

Brown solid (92% yield); IR (KBr) 3230 (NH), 1655 (C=N), 1598, 1510 (C=S), 1494 (C=S), 1389, 1303, 1156, 1066 cm^−1^; ^1^H NMR (CDCl_3_, 400 MHz) δ 9.52 (s, 1H, NH), 7.54 (d, *J* = 8.0 Hz, 2xCH_p-chlorophenyl_), 7.45 (d, *J* = 8.0 Hz, 2xCH_p-chlorophenyl_), 7.39–7.33 (m, 2H, Ar-H_p-fluorophenyl_), 7.29–7.22 (m, 2H, Ar-H_p-fluorophenyl_) ppm; ^13^C NMR (CDCl_3_, 100 MHz) δ 180.0 (C=S), 179.9 (C=S), 163.0 (d, *J* = 250.0 Hz, C-F), 156.1 (**C**=NH), 135.5 (C-Cl), 132.2 (*N*_(1)_-**C_q_**), 130.9 (d, *J* = 4.0 Hz, *N*_(3)_-**C_q_**), 130.3 (d, *J* = 9.0 Hz, 2xCH_p-fluorophenyl_), 129.8 (2xCH_p-chlorophenyl_), 129.6 (2xCH_p-chlorophenyl_), 116.7 (d, *J* = 23.0 Hz, 2xCH_p-fluorophenyl_) ppm; HRMS (ESI^+^): *m*/*z* [M + H]^+^ calcd for C_15_H_10_FClN_3_S_2_: 349.9989; found: 349.9978.

3-(4-fluorophenyl)-5-imino-1-(4-methoxyphenyl)imidazolidine-2,4-dithione (**18d″**) 

Red brown solid (58% yield); IR (KBr) 3229 (NH), 1654 (C=N), 1607, 15101 (C=S), 1388, 1303, 1155, 1068 cm^−1^; ^1^H NMR (CDCl_3_, 400 MHz) δ 9.49 (s, 1H, NH), 7.421–7.35 (m, 4H, Ar-H), 7.28–7.22 (m, 2H, Ar-H_p-fluorophenyl_), 7.06 (d, *J* = 8.0 Hz, 2H, Ar-H_p-methoxyphenyl_), 3.86 (s, 3H, OMe) ppm; ^13^C NMR (CDCl_3_, 100 MHz) δ 180.6 (C=S), 180.3 (C=S), 162.9 (d, *J* = 250.0 Hz, C-F), 160.1 (**C**-O), 156.6 (**C**=NH), 131.0 (d, *J* = 4.0 Hz, *N*_(3)_-**C_q_**), 130.3 (d, *J* = 9.0 Hz, 2xCH_p-fluorophenyl_), 129.3 (2xCH_p-methoxyphenyl_), 126.3 (*N*_(1)_-**C_q_**), 116.7 (d, *J* = 23.0 Hz, 2xCH_p-fluorophenyl_), 114.8 (2xCH_p-methoxyphenyl_), 55.5 (CH_3_) ppm; HRMS (ESI^+^): *m*/*z* [M + H]^+^ calcd for C_16_H_13_FN_3_OS_2_: 346.0484; found: 346.0495.

3-(4-fluorophenyl)-5-imino-1-(4-nitrophenyl)imidazolidine-2,4-dithione (**18e″**) 

Green solid (72% yield); IR (KBr) 3231 (NH), 1660 (C=N), 1596, 1522 (C=S), 1418, 1386, 1285, 1073 cm^−1^; ^1^H NMR (CDCl_3_, 400 MHz) δ 9.60 (s, 1H, NH), 8.43 (d, *J* = 8.0 Hz, 2H, Ar-H_p-nitrophenyl_), 7.77 (d, *J* = 8.0 Hz, 2H, Ar-H_p-nitrophenyl_), 7.40–7.34 (m, 2H, Ar-H_p-fluorophenyl_), 7.31–7.24 (m, 2H, Ar-H_p-fluorophenyl_), ppm; ^13^C NMR (CDCl_3_, 100 MHz) δ 179.4 (C=S), 179.3 (C=S), 163.1 (d, *J* = 250.0 Hz, C-F), 155.6 (**C**=NH), 147.6 (NO_2_-**C_q_**), 139.2 (*N*_(1)_-**C_q_**), 130.6 (d, *J* = 3.0 Hz, *N*_(3)_-**C_q_**), 130.2 (d, *J* = 9.0 Hz, 2xCH_p-fluorophenyl_), 129.5 (2xCH_p-nitrophenyl_), 124.6 (2xCH_p-nitrophenyl_), 116.8 (d, *J* = 23.0 Hz, 2xCH_p-fluorophenyl_) ppm; HRMS (ESI^+^): *m*/*z* [M + H]^+^ calcd for C_15_H_10_FN_4_O_2_S_2_: 361.0229; found: 361.0238.

1-(3-fluorophenyl)-5-imino-3-(p-tolyl)imidazolidine-2,4-dithione (**18e″**) 

Brown solid (55% yield); IR (KBr) 3232 (NH), 1657 (C=N), 1599, 1511 (C=S), 1495 (C=S) cm^−1^; ^1^H NMR (CDCl_3_, 400 MHz) δ 9.51 (s, 1H, NH), 7.57–7.51 (m, 1H, Ar-H), 7.41–7.18 (m, 7H, Ar-H_m-fluorophenyl_), 2.46 (s, 3H, Ar- CH_3_) ppm; ^13^C NMR (CDCl_3_, 100 MHz) δ 180.2 (C=S), 179.9 (C=S), 162.7 (d, *J* = 248.0 Hz, C-F), 156.2 (**C**=NH), 140.4 (C**_q_**-Me), 135.1 (d, *J* = 10.0 Hz, *N*_(1)_-**C_q_**), 132.5 (*N*_(3)_-**C_q_**), 130.6 (d, *J* = 9.0 Hz, CH_m-fluorophenyl_), 130.3 (2xCH_p-tolyl_), 127.9 (2xCH_p-tolyl_), 124.2 (d, *J* = 9.0 Hz, CH_m-fluorophenyl_), 116.7 (d, *J* = 21.0 Hz, CH_m-fluorophenyl_), 116.1 (d, *J* = 24.0 Hz, CH_m-fluorophenyl_), 21.4 (CH_3_) ppm; HRMS (ESI^+^): *m*/*z* [M + H]^+^ calcd for C_16_H_13_FN_3_S_2_: 330.0535; found: 330.0529.

## 4. Conclusions

In summary, we successfully developed a TEA mediated regiospecific synthesis of 1,3-disbustituted-imidazolidine-5-imino-2,4-dithiones through the reaction of *N*-arylcyanothioformamides **1** and isothiocyanates **16** in DMF at room temperature. The reaction accepts a range of functional groups on both reactants including the typical halides, alkoxides, nitro, alkyl, and haloalky functions and affords a broad scope of products as demonstrated by the preparation of 57 derivatives of 1,3-disubstituted imidazolidine-5-imino-2,4-dithione. This strategy is operationally simple, catalytic, and provides the desired products in moderate to excellent yields in exclusive regioselectivity. Proof of the molecular structure of the isolated regioisomer was obtained for the first time by single-crystal X-ray diffraction analysis of two imidazolidineiminodithione derivatives. Compound **1** reacts exclusively via the nitrogen atom in DMF as the solvent. The protocol described herein represents the first reported approach to prepare **18** regioselectively under very mild conditions using ambient temperature and a weak base used in a catalytic amount. The products were isolated by an ether/brine workup and required no further purification by chromatography. To support our proposed mechanism and experimental observations regarding the regiochemical outcome, density functional theory (DFT) calculations were performed at the B3LYP-D4/def2-TZVP level in DMF as the implicit solvent. The current synthetic technique is promising for the rapid synthesis of imidazolidineiminodithione libraries, enabling medicinal chemists to explore their potential bioactivities.

## Figures and Tables

**Table 1:** Reaction of *N*-arylcyanothioformamides **1a**,**b** with aryl isothiocyanates **16a**–**c** in various solvents ^a.^

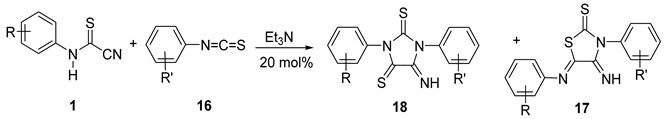						
Entry	Solvent	Time (h)	^b^ Product Distribution (%) ^a^ 18/17			
			^d^ PhNHCSCN (1a) + 2FPhNCS (16b)	^c^ PhNHCSCN (1a) + PhNCS (16a)	^d^ 2-FPhNHCSCN (1b) + PhNCS (16a)	^d^ PhNHCSCN (1a) + 4-NO_2_PhNCS (16c)
**1**	MeNO_2_	1	29:71	10:90	43:57	-
**2**	Dioxane	2	55:45	36:64	79:21	-
**3**	C_6_H_6_	22	62:38	39:61	52:48	-
**4**	Toluene	22	53:47	42:58	63:37	-
**5**	THF	1	43:57	46:54	98:2	100:0
**6**	EtOAc	1	38:62	65:35	69:31	100:0
**7**	Et_2_O	1	51:49	67:33	92:8	100:0
**8**	CHCl_3_	1	86:14	68:32	66:34	100:0
**9**	MeOH	1	44:56	70:30	86:14	-
**10**	EtOH	22	52:48	72:28	64:36	94:6
**11**	CH_2_Cl_2_	1	74:26	84:16	78:22	-
**12**	MeCN	1.4	96:4	90:10	100:0	-
**13**	Acetone	1	90:10	93:07	94:6	100:0
**14**	DMSO	1	97:03	97:03	97:3	100:0
**15**	DMF	1	100:0	100:0	100:0	100:0

^a^ Product was not isolated from the reaction mixture. ^b^ Product distribution was measured on the crude mixture by ^1^H NMR. ^c^ The reaction time used for this pair of reactants was 21–24 h. ^d^ The reaction time used for these pairs is as indicated in the table.

**Table 2:** Reaction of phenylcarbamothioyl cyanide **1a** with phenyl isothiocyanate **16a** in DMF at 18 °C with varying amounts of TEA.

Entry	Solvent	TEA (mol%)	Reaction Time (h)	^a^ Regioisomer	^b^ Conversion (%)
**1**	DMF	10	7.5	**18a**	60
**2**	DMF	20	7.5	**18a**	70
**3**	DMF	30	7.5	**18a**	80
**4**	DMF	40	7.5	**18a**	90
**5**	DMF	10	24	**18a**	100
**6**	DMF	20	24	**18a**	100
**7**	DMF	30	24	**18a**	100
**8**	DMF	40	24	**18a**	100
**9**	DMF	50	7.5	**18a**	100
**10**	DMF	60	7.5	**18a**	^c^ 100
**11**	DMF	70	7.5	**18a**	^c^ 100
**12**	DMF	80	7.5	**18a**	^c^ 100
**13**	DMF	90	7.5	**18a**	^c^ 100
**14**	DMF	100	7.5	**18a**	^c^ 100

^a^ Product was isolated from the reaction mixture by ether/brine workup and recrystallized from ethanol. ^b^ Percent conversion was measured on the crude mixture by ^1^H NMR. ^c^ Starting materials reacted completely but slow degradation of product **18a** with 50–100 mol% TEA afforded reaction mixtures with some minor unidentified side products.

**Table 3:** Global reactivity descriptors of the individual reactants calculated at the B3YLP-D4/def2-TZVP in implicit DMF as the solvent. All energies are in eV. The orbital numbering of the FMO is indicated in parentheses with MO indices starting at 0. The E_HOMO_ for the reference compound tetracyanoethylene (TCE) is −8.7335 eV, computed at the same level of theory.

Global Reactivity Descriptors	Reactant 1 (Cyano-Thioformamide Anion)	Reactant 16 Phenyl Isothiocyanate
E_HOMO_ (eV)	−5.406 (#41)	−6.354 (#34)
E_LUMO_ (eV)	−1.198 (#42)	−1.287 (#35)
HOMO-LUMO gap (eV) or chemical global hardness η = E_LUMO_ − E_HOMO_	4.208	5.067
softness s = 1/η (eV^−1^)	0.238	0.197
chemical potential μ (eV) = (E_HOMO_ + E_LUMO_)/2	−3.302	−3.821
global electrophilicity ω (eV) = μ^2^/(2η)	1.295	1.440
global nucleophilicity *N* (eV) = E_HOMO_ − E_HOMO_ (TCE)	3.327	2.379

## Data Availability

The data that support the findings of this study are available in the Supplementary Material of this article. Samples of the compounds are available from the authors.

## Supplementary Materials

The following supporting information can be downloaded at: https://www.mdpi.com/article/10.3390/molecules29163958/s1: The relaxed structures, the trajectories of the minimum energy path (“NEB.xyz”) and the imaginary normal mode (“opt.hess.v006.xyz”). All files use the xyz format and can be found in the zip file “structs.zip”.

## References

[1] Moussa Z., Judeh Z.M.A., Sh El-Sharief A.M. (2020). *N*-arylcyanothioformamides: Preparation methods and application in the synthesis of bioactive molecules. ChemsitrySelect.

[2] Abbas S.Y., Sh (2020). El-Sharief, M.A.M.; Salem, M.A.; Sh. El-Sharief, A.M. Utilization of cyanothioformamides in the syntheses of various types of imidazole derivatives. Synth. Commun..

[3] Olin J.F. (1966). Herbicidal Composition and Method Employing Thiooxanilonitriles. U.S. Patent.

[4] Olin J.F., Darlington W.A. (1971). Gastropodicidal 3,4-Dichloro-and 3,5-Dichloro-thiooxanilonitriles. U.S. Patent.

[5] Sh El-Sharief M.A.M., Moussa Z., Sh El-Sharief A.M. (2011). Synthesis, characterization, and derivatization of some novel types of fluorinated mono- and bis-imidazolidineiminothiones with antitumor, antiviral, antibacterial, and antifungal activities. J. Fluor. Chem..

[6] Kobayashi-Matsunaga Y., Ishii T., Hamaguchi T., Osada H., Sato M. (2005). Synthesis and characterization of a novel protein tyrosine phosphatase inhibitor, 2-(cyclobutylamino)-*N*-(2-furylmethyl)-2-thioxoacetamide. Lett. Drug Des. Discov..

[7] Sh El-Sharief A.M., Moussa Z. (2009). Synthesis, characterization and derivatization of some novel types of mono- and bis-imidazolidineiminothiones and imidazolidineiminodithiones with antitumor, antiviral, antibacterial and antifungal activities—Part I. Eur. J. Med. Chem..

[8] Friedrich J.D. (1987). Preparation and chemistry of the Diels-Alder adducts of levopimaric acid and activated thiocarbonyl dienophiles. J. Org. Chem..

[9] Friedrich K., Zamkanei M. (1979). En-reaktionen mit cyanthioformaniliden. Chem. Ber..

[10] Sh El-Sharief A.M., Mahmoud F.F., Taha N.M., Ahmed E.M. (2005). Reactions of cyanothioformamide and thiohydantoin derivatives with some arylidenes of cyanothioacetamide and other electrophilic and nucleophilic reagents. Phosphorus Sulfur Silicon Relat. Elem..

[11] El-Gaby M.S.A., Ammar Y.A., El-Sharief A.M. Sh., Zahran M.A., Khames A.A. (2002). Reactivity of cyanothioformamides and 3-(4-bromophenyl)-5-imino-4-oxazolidinethione toward *ortho*-substituted nucleophiles. Heteroat. Chem..

[12] Sh El-Sharief A.M., Ammar Y.A., Mohamed Y.A., El-Gaby M.S.A. (2002). A comparative study of the behavior of cyanothioformamide and oxazolidine (thiones or iminothiones) towards some binucleophiles. Heteroat. Chem..

[13] Sh El-Sharief A.M., Ammar Y.A., Zahran M.A., Sabet H.K. (2003). Utility of cyanothioformamides in synthesis of some bis(imidazole, oxazole, thiazole, oxadiazole, triazole, benzoxazinethione and quinazoline) derivatives. J. Chem. Res., Synop..

[14] Sh El-Sharief A.M., Ketcham R., Ries M., Schaumann E., Adiwidjajad G. (2010). Imidazoline-4-thiones from cyanothioformamides and aldehyde imines: Formation, aromatization, and acetylation. J. Heterocycl. Chem..

[15] Ketcham R., Schaumann E., Niemer T. (1980). Heterocyclic ring-closure reactions; VII. A facile synthesis of 5-imino-4-oxazolidinethiones and 4-thioxo-5-oxazolidinones. Synthesis.

[16] Deck L.M., Papadopoulos E.P., Smith K.A. (2003). Reactions of isocyanates with methyl N-(cyanothioformyl)anthranilate. J. Heterocycl. Chem..

[17] Tanaka K., Matsuo K., Nakanishi A., Jo M., Shiota H., Yamaguchi M., Yoshino S., Kawaguchi K. (1984). Syntheses and antimicrobial activities of five-membered heterocycles having a phenylazo substituent. Chem. Pharm. Bull..

[18] Sh El-Sharief A.M., Al-Amri A., Al-Raqa S.Y. (2006). Halogenated, alkylated and new types of imidazolidine, pyrrolidine, imidazotriazine and thienoimidazole derivatives with biological and antitumor activities. J. Sulfur Chem..

[19] Elagamey A.A., El-Taweel F.M.A., El-Enein R.A.N.A. (2006). New synthetic routes to 1,3,4-thiadiazole derivatives. Phosphorus Sulfur Silicon Relat. Elem..

[20] Al-Raqa S.Y., Sh El-Sharief A.M., Khalil S.M.E., Al-Amri A.M. (2006). Synthesis of some novel imidazolidine derivatives and their metal complexes with biological and antitumor activity. Heteroat. Chem..

[21] Grabenko A.D., Danchenko M.N., Pel’kis P.S.J. (Australia 1972). Journal of Organic Chemistry of the USSR (Engl. Transl.).

[22] Reißert A., Brüggemann K. (1924). Über die einwirkung des cyankaliums auf aromatische senföle. Chem. Ber..

[23] Reißert A. (1904). Die geschwefelten anilide der oxalsäure und ihre umwandlungsproducte. Chem. Ber..

[24] English R.F., Rakitin O.A., Rees C.W., Vlasova O.G. (1997). Conversion of imino-1,2,3-dithiazoles into 2-cyanobenzothiazoles, cyanoimidoyl chlorides and diatomic sulfur. J. Chem. Soc. Perkin Trans. 1.

[25] Lee H., Kim K. (1992). A new procedure to N-arylcyanothioformamides from 5-arylimino-4-chloro-5h-1,2,3-dithiazoles. Bull. Korean Chem. Soc..

[26] Besson T., Rees C.W. (1995). Some chemistry of 4,5-dichloro-1,2,3-dithiazolium chloride and its derivatives. J. Chem. Soc. Perkin Trans. 1.

[27] Besson T., Emayan K., Rees C.W. (1995). 3,1-Benzoxazin-4-ones, 3,1-benzothiazin-4-ones and N-arylcyanothioformamides. Chem. Commun..

[28] Besson T., Emayan K., Rees C.W. (1995). 1,2,3-Dithiazoles and new routes to 3,1-benzoxazin-4-ones, 3,1-benzothiazin-4-ones and N-arylcyanothioformamides. J. Chem. Soc. Perkin Trans. 1.

[29] Cottenceau G., Besson T., Gautier V.C., Rees C.W., Pons A.-M. (1996). Antibacterial evaluation of novel N-Arylimino-1,2,3-dithiazoles and N-arylcyanothioformamides. Bioorg. Med. Chem. Lett..

[30] Besson T., Cottenceau G., Pons A.-M. (1996). Antimicrobial evaluation of 3,1-benzoxazin-4-ones, 3,1-benzothiazin-4-ones, 4-alkoxyquinazolin-2-carbonitriles and N-arylimino-1,2,3-dithiazoles. Bioorg. Med. Chem. Lett..

[31] Besson T., Guillard J., Rees C.W. (2000). Rapid synthesis of 2-cyanobenzothiazole, isothiocyanate and cyanoformanilide derivatives of dapsone. J. Chem. Soc. Perkin Trans. 1.

[32] Steinhardt R.C., O‘Neill J.M., Rathbun C.M., McCutcheon D.C., Paley M.A., Prescher J.A. (2016). Design and synthesis of an alkynyl luciferin analogue for bioluminescence imaging. Chem. Eur. J..

[33] Lee H.-S., Kim K. (1996). Reactions of 5-arylimino-4-chloro-5H-1,2,3-dithiazoles with stable phosphoranes: Novel preparation of dithiomethylenephosphoranes. Tetrahedron Lett..

[34] Michaelidou S.S., Koutentis P.A. (2009). The Synthesis of 2-cyano-cyanothioformanilides from 2-(4-chloro-5h-1,2,3-dithiazol-5-ylideneamino)benzonitriles using DBU. Synthesis.

[35] McCutcheon D.C., Porterfield W.B., Prescher J.A. (2015). Rapid and scalable assembly of firefly luciferase substrates. Org. Biomol. Chem..

[36] Nilsson N.H., Senning A. (1972). C-Sulfonyl-thioformamide als thiocarbamoylierungsreagenzien. Synthesis.

[37] Sh El-Sharief A.M., Al Raqa S.Y. (2007). New types of mono and bis sulfonamides, tosylamino acids and thiosulfonic ester derived from xanthotoxin, bergapten and visnagin with biological interest. Phosphorus Sulfur Silicon Relat. Elem..

[38] Ketcham R., Schaumann E. (1980). Heterocyclic ring-closure reactions. 6. Preparation and further cyclization reactions of 5-imino-1,3-diphenyl-4-thioxo-2-imidazolidinone and 5-imino-1,3-diphenyl-2,4-imidazolidinedithione. J. Org. Chem..

[39] Khattak I., Ketcham R., Schaumann E., Adiwidjaja G. (1985). Heterocyclic ring-closure reactions. 8. Reactions of isothiocyanates with cyanothioformamides. Cyclization of 5-imino-4-thioxo-2-imidazolidones and 5-imino-2,4-imidazolidinedithiones to 5,7-dihydrodiimidazo[4,5-b:4’,5’-e]pyrazine-2,6(1H,3H)-diones and -dithiones. J. Org. Chem..

[40] Huang J., Graves M.D. (1987). Thiazolidinethiones vs. imidazolidinedithiones as products from cyanothioformanilides and phenyl isothiocyanates. J. Heterocycl. Chem..

[41] Moussa Z., Paz A.P., Judeh Z.M.A., Alzamly A., Saadeh H.A., Asghar B.H., Alsaedi S., Masoud B., Almeqbaali S., Estwani S. (2023). First X-ray Crystal Structure Characterization, Computational Studies, and Improved Synthetic Route to the Bioactive 5-Arylimino-1,3,4-thiadiazole Derivatives. Int. J. Mol. Sci..

[42] Neese F. (2012). The ORCA Program System. WIREs Comput. Mol. Sci..

[43] Moussa Z., Paz A.P., Khalaf M.A., Judeh Z.M.A., Alzamly A., Samadi A., Al-Fahemi J.H., Tatina M.B., Al-Masri H.T., Jassas R.S. (2023). First Exclusive Stereo- and Regioselective Preparation of 5-Arylimino-1,3,4-Selenadiazole Derivatives: Synthesis, NMR Analysis, and Computational Studies. Chem Asian J..

[44] Henkelman G., Uberuaga B.P., Jónsson H. (2000). A climbing image nudged elastic band method for finding saddle points and minimum energy paths. J. Chem. Phys..

[45] Lu T., Chen F. (2012). Multiwfn: A Multifunctional Wavefunction Analyzer. J. Comp. Chem..

