# Supplementary material for: Exclusive Solvent-Controlled Regioselective Catalytic Synthesis of Potentially Bioactive Imidazolidineiminodithiones: NMR Analysis, Computational Studies and X-ray Crystal Structures
Source: Molecules. 2024 Aug 22;29(16):3958. doi: 10.3390/molecules29163958 (PMC11357535; doi:10.3390/molecules29163958)
Supplement: Supplementary file 1 [file molecules-29-03958-s001.zip › molecules-3127678-supplementary.pdf]

Supporting Information for

Exclusive Solvent-Controlled Regioselective Catalytic Synthesis of Potentially Bioactive Imidazolidineiminodithiones: NMR analysis, Computational Studies and X-ray Crystal Structures

Ziad Moussa [1,*], Sara Saada [1], Alejandro Perez Paz [1,*], Ahmed Alzamly [1], Zaher M. A. Judeh [2], Aaesha R. Alshehhi [1], Aisha Khudhair [1], Salama A. Almheiri [1], Harbi Tomah Al-Masri [3] and Saleh A. Ahmed [4,*]

[1] Department of Chemistry, College of Science, United Arab Emirates University, Al Ain P. O. Box 15551, United Arab Emirates
[2] School of Chemical and Biomedical Engineering, Nanyang Technological University, 62 Nanyang Drive, N1.2–B1-14, Singapore 637459, Singapore
[3] Department of Chemistry, Faculty of Sciences, Al al-Bayt University, P. O. Box 130040, Mafraq 25113, Jordan
[4] Department of Chemistry, Faculty of Applied Sciences, Umm Al-Qura University, Makkah 21955, Saudi Arabia
* Correspondence: zmoussa@uaeu.ac.ae (Z.M.); aperez@uaeu.ac.ae (A.P.P.); saleh_63@hotmail.com (S.A.A.)

**Table of Contents**	S2
1D and 2D NMR spectra of **18a-z**, **18a'-z'**, and **18a''-18e''**	S3-S320
Regioisomeric ratios of **18**:**17** in various solvents and different substituents	S321-S365
Crystal-structure data for (4-methoxyphenyl)carbamothioyl cyanide (**1g**)	S366-S369
1D/2D NMR structural analysis of a typical imidazolidineiminodithione **18g'**	S370-371
Crystal-structure data for 5-imino-1-(4-nitrophenyl)-3-(p-tolyl)imidazolidine-2,4-dithione (**18g'**).	S372-S377
Crystal-structure data for 5-imino-1-(4-nitrophenyl)-3-phenylimidazolidine-2,4-dithione (**18h**).	S378-S384
Computational details	S385-S390

General Information. Reactions were conducted with magnetic stirring in air-dried glassware. All reagents and reaction solvents were used as received without any further purification. Analytical thin-layer chromatography (TLC) was used to follow the progress of reactions and was carried out on precoated silica gel plates (HSGF 254) and visualized under UV irradiation (254 nm). ^{1}H and ^{13}C NMR spectra were recorded in DMSO-d_6 or $CDCl_3$ on a Bruker DPX 300 and 75 MHz NMR spectrometer and on a Varian 400 and 100 MHz NMR spectrometer. The NMR chemical shifts (δ) are reported in parts per million (ppm) relative to the residual solvent peak (^{1}H-NMR δ 7.26 for $CDCl_3$, δ 2.50 for DMSO-d_6; ^{13}C-NMR δ 77.0 for $CDCl_3$, δ 39.52 for DMSO-d_6).

General procedure for the preparation of N-arylcyanothioformamides ***1a-g***

N-arylcyanothioformamides **1a-g** (Scheme 3) were synthesized according to our previously published protocol which can be found in reference 14 in the article. The reaction involved the equimolar reaction of different isothiocyanates with KCN in a mixture of water and ethanol at room temperature for a duration of 3 hours. Detailed physical and spectral data can be found in the Supporting Information section of reference 14 in the article.

General procedure for the preparation of isothiocyanate ***16j***

A mixture of benzoyl chloride (20 mmol) and potassium thiocyanate (20 mmol) in acetone (120 mL) was stirred at room temperature for 30 min. The resulting heterogeneous mixture was filtered through a cintered glass funnel to remove the KCl salt and separate the organics. Removal of the solvent under *vacuo* afforded **16j** as a yellow oil (97% yield). ^{1}H NMR ($CDCl_3$, 400 MHz) δ 7.90-7.81 (m,1H), 7.80-7.78 (m, 1H), 7.62-7.56 (m, 1H), 7.54-7.50 (m, 1H), 7.49-7.45 (m, 1H).

^{1}H-NMR ($CDCl_3$) spectrum of 5-imino-1,3-diphenylimidazolidine-2,4-dithione (18a)

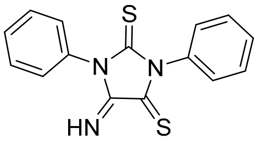

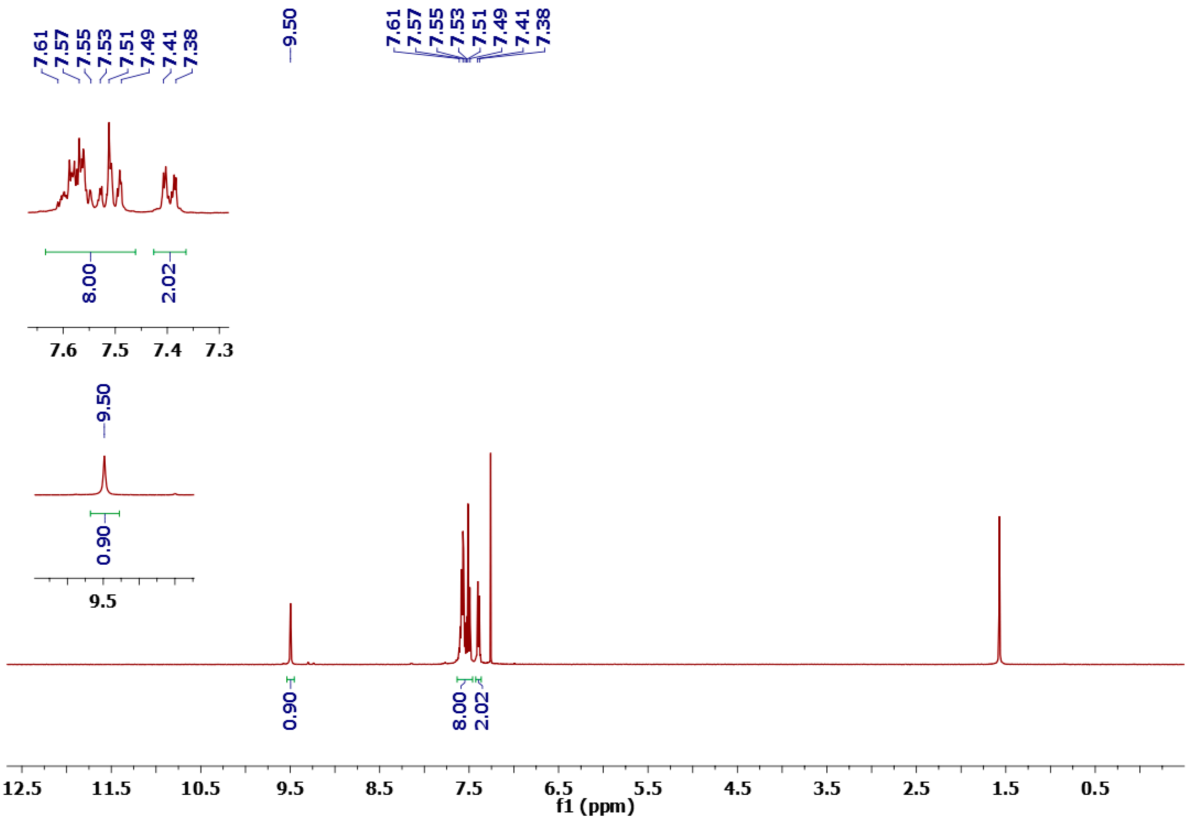

^{13}C NMR ($CDCl_3$) spectrum of 5-imino-1,3-diphenylimidazolidine-2,4-dithione

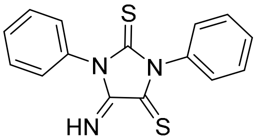

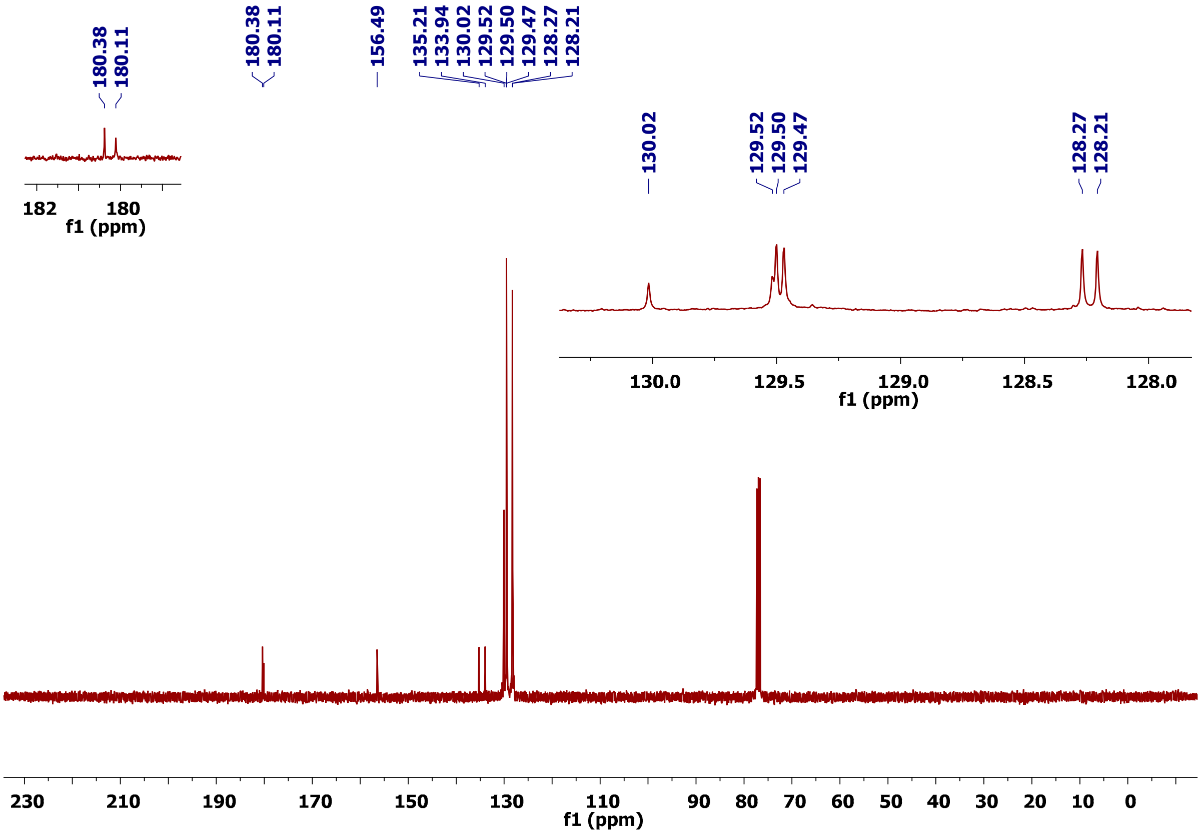

^{13}C-CRAPT NMR ($CDCl_3$) spectrum of 5-imino-1,3-diphenylimidazolidine-2,4-dithione

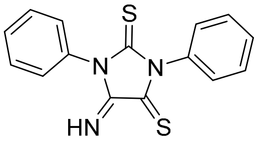

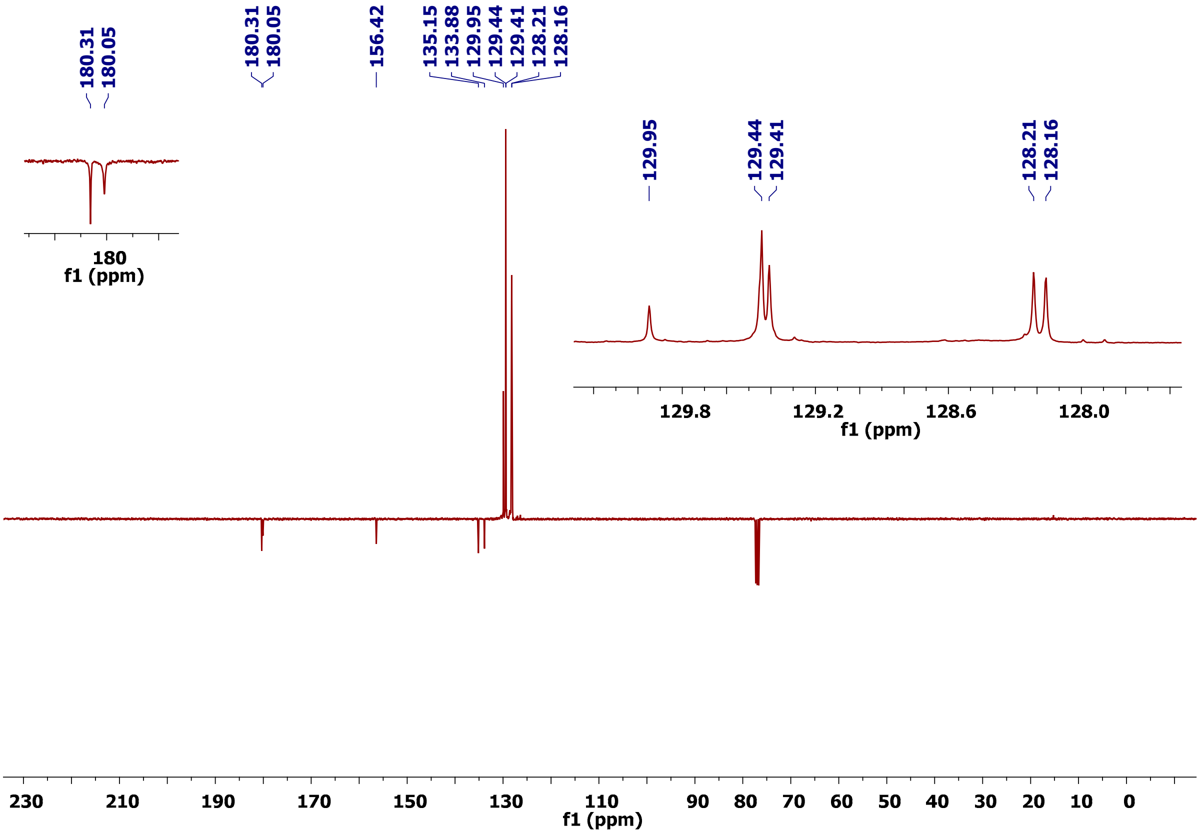

^{1}H-NMR (DMSO-d$_6$) spectrum of 5-imino-1,3-diphenylimidazolidine-2,4-dithione

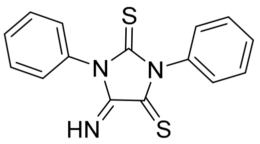

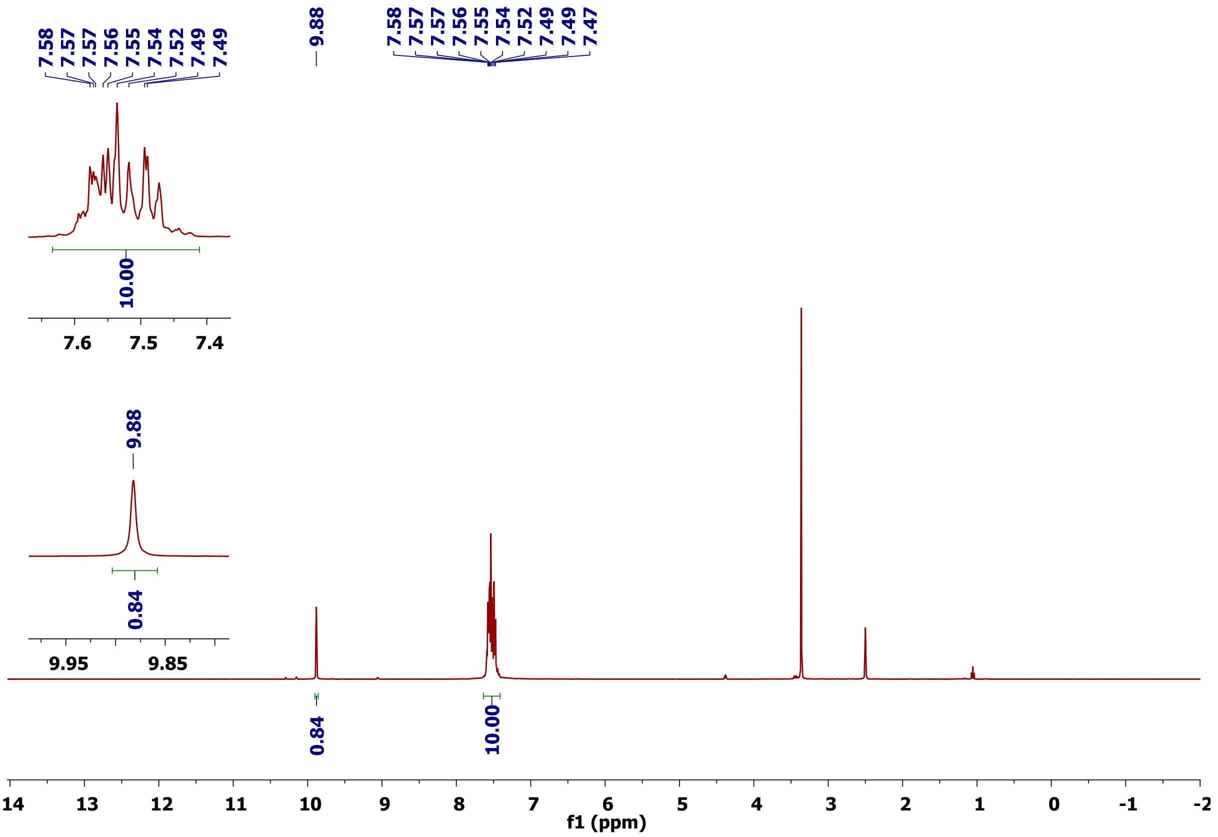

^{13}C NMR (DMSO-d_6) spectrum of 5-imino-1,3-diphenylimidazolidine-2,4-dithione

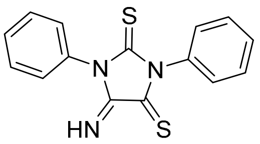

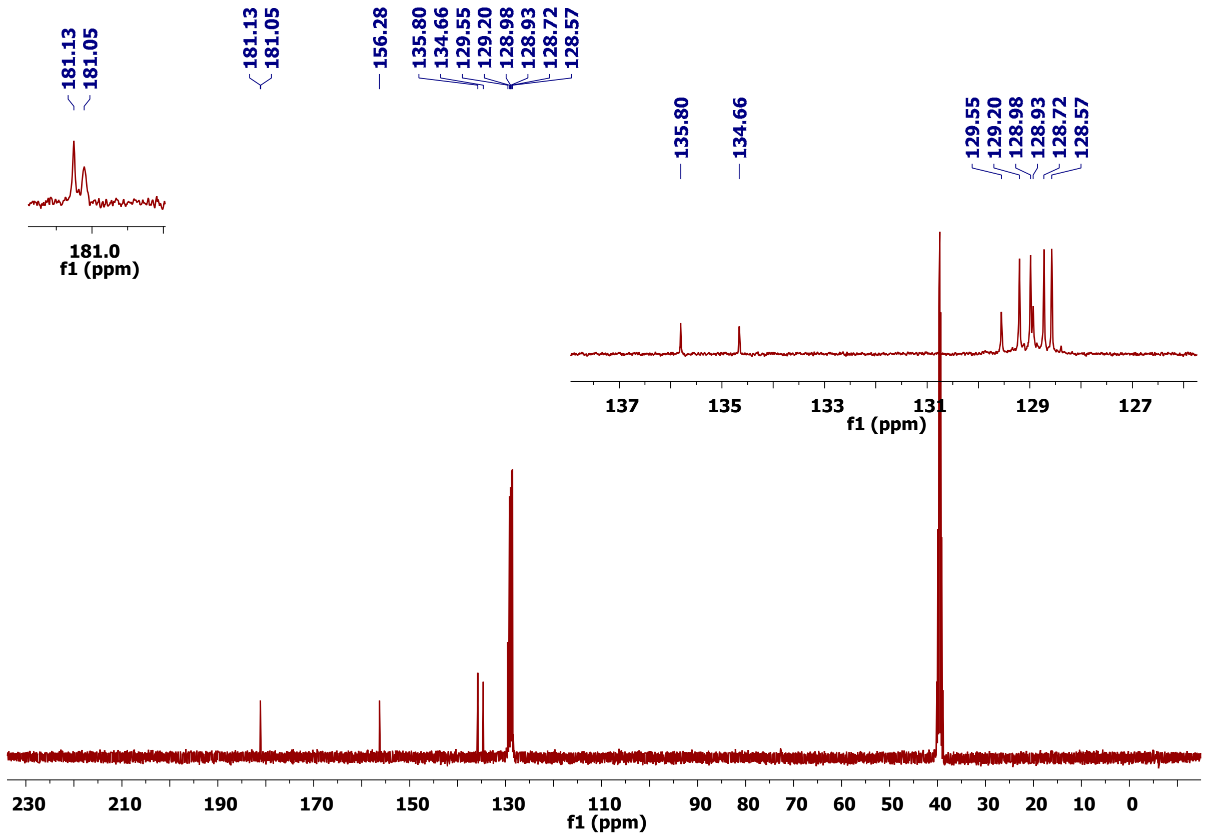

^{13}C-CRAPT NMR (DMSO-d_6) spectrum of 5-imino-1,3-diphenylimidazolidine-2,4-dithione

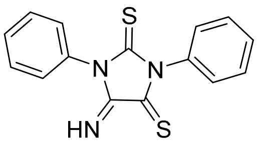

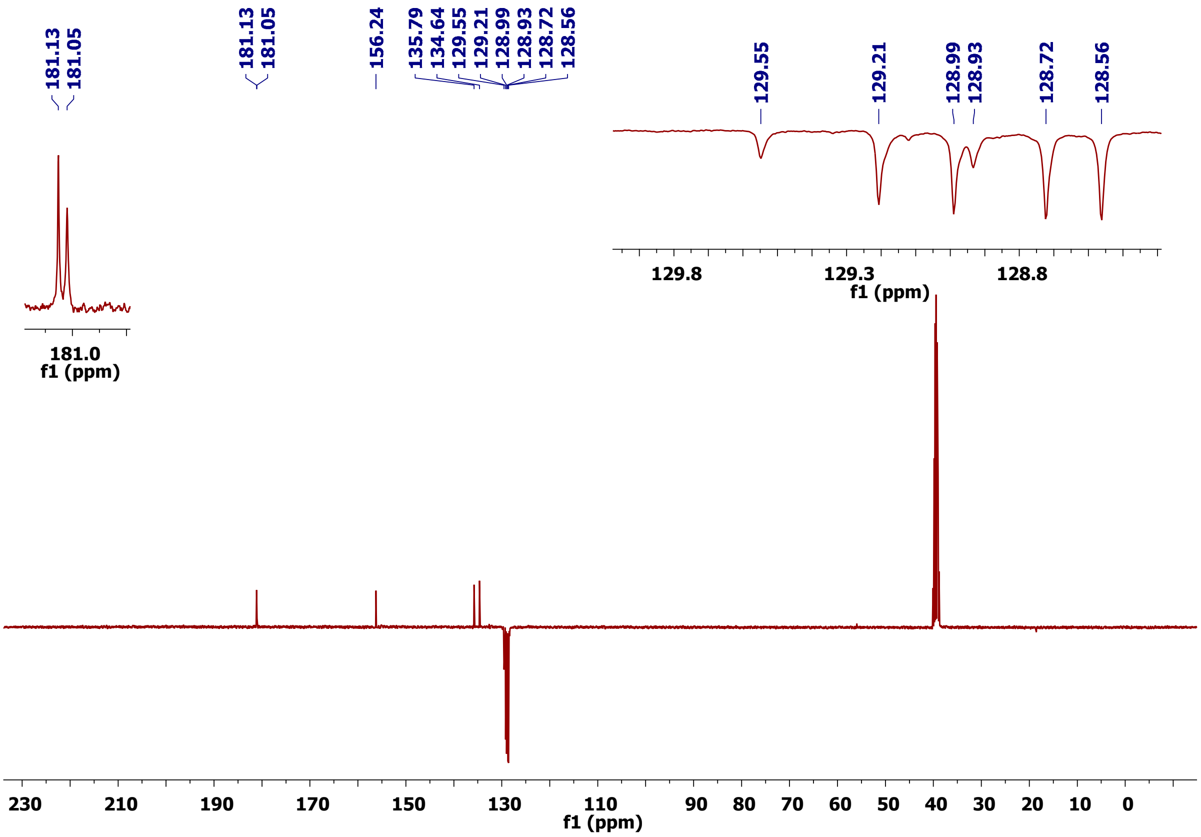

^{1}H NMR ($CDCl_3$) spectrum of (5-imino-3-phenyl-2,4-dithioxoimidazolidin-1-yl)(phenyl)methanone (18b)

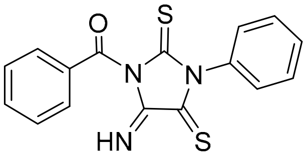

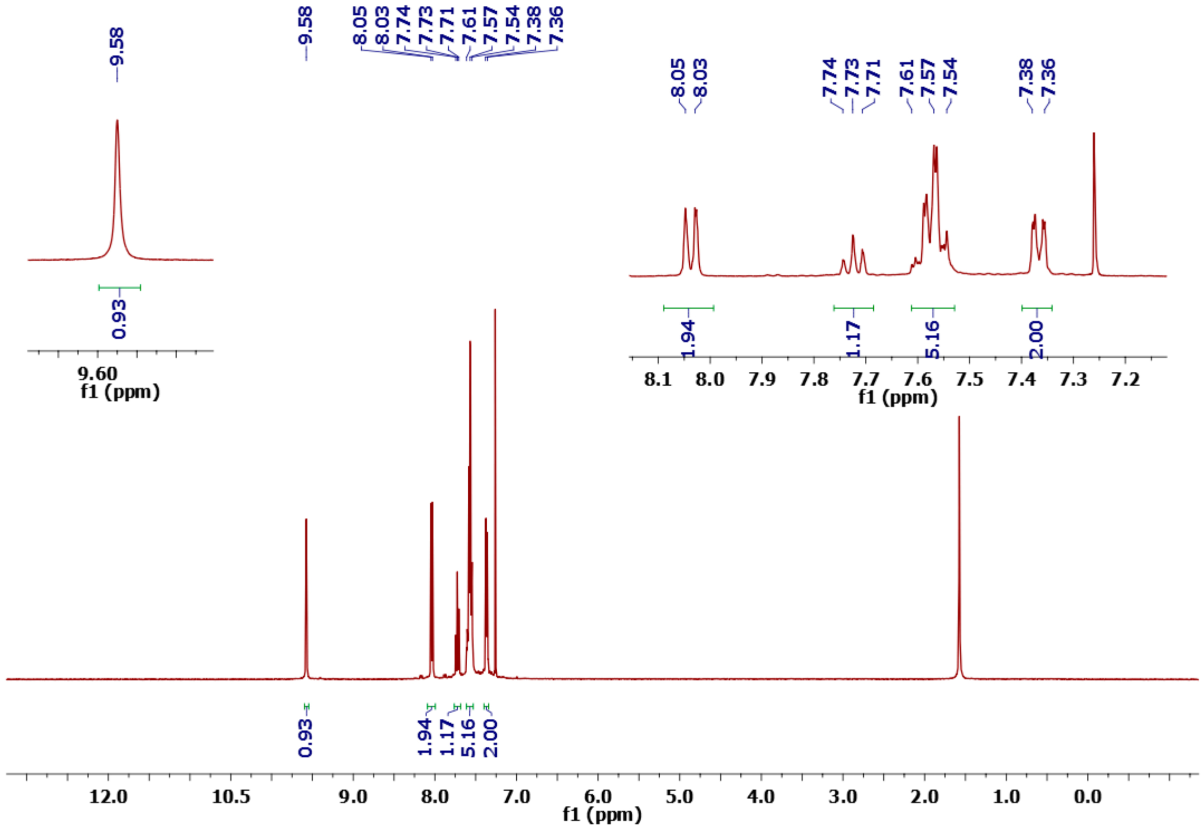

^{13}C NMR ($CDCl_3$) spectrum of (5-imino-3-phenyl-2,4-dithioxoimidazolidin-1-yl)(phenyl)methanone

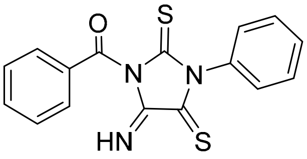

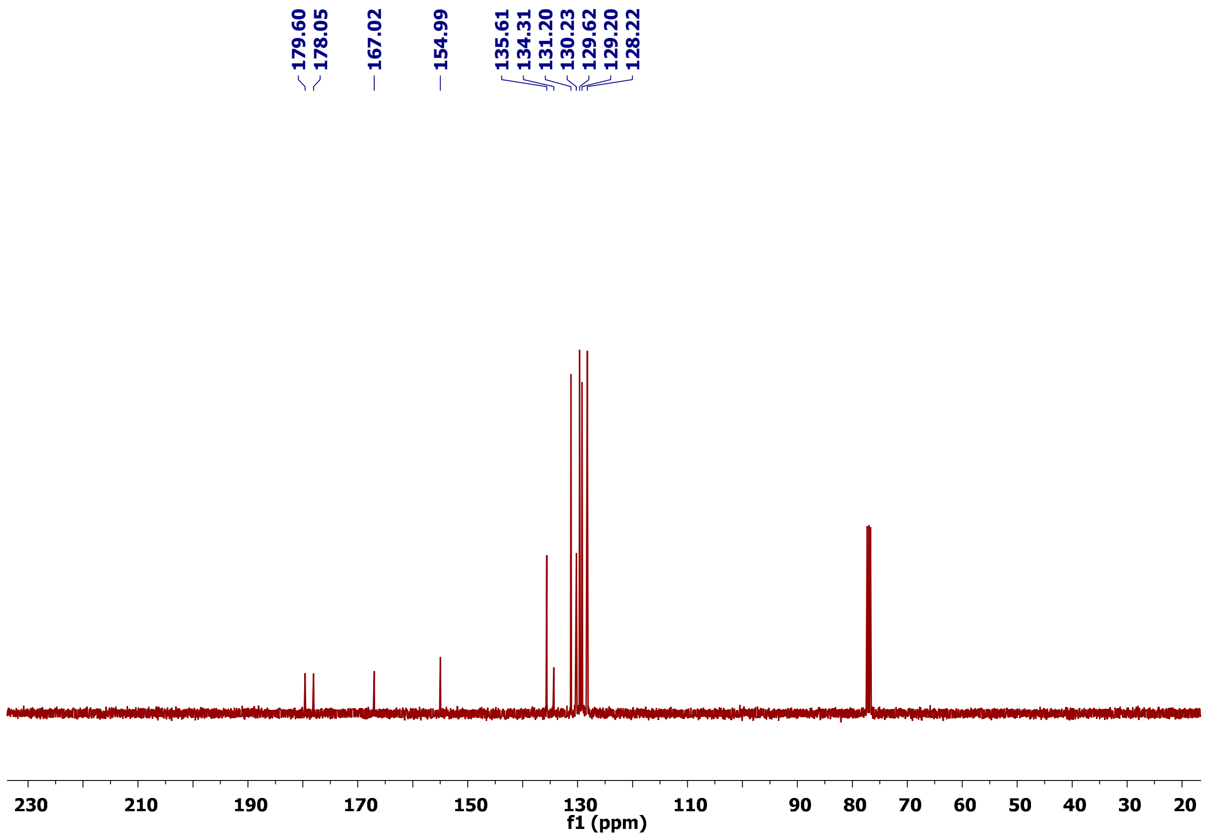

^{13}C-CRAPT NMR ($CDCl_3$) spectrum of (5-imino-3-phenyl-2,4-dithioxoimidazolidin-1-yl)(phenyl)methanone

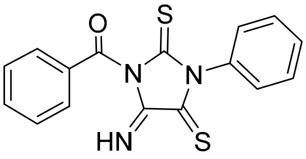

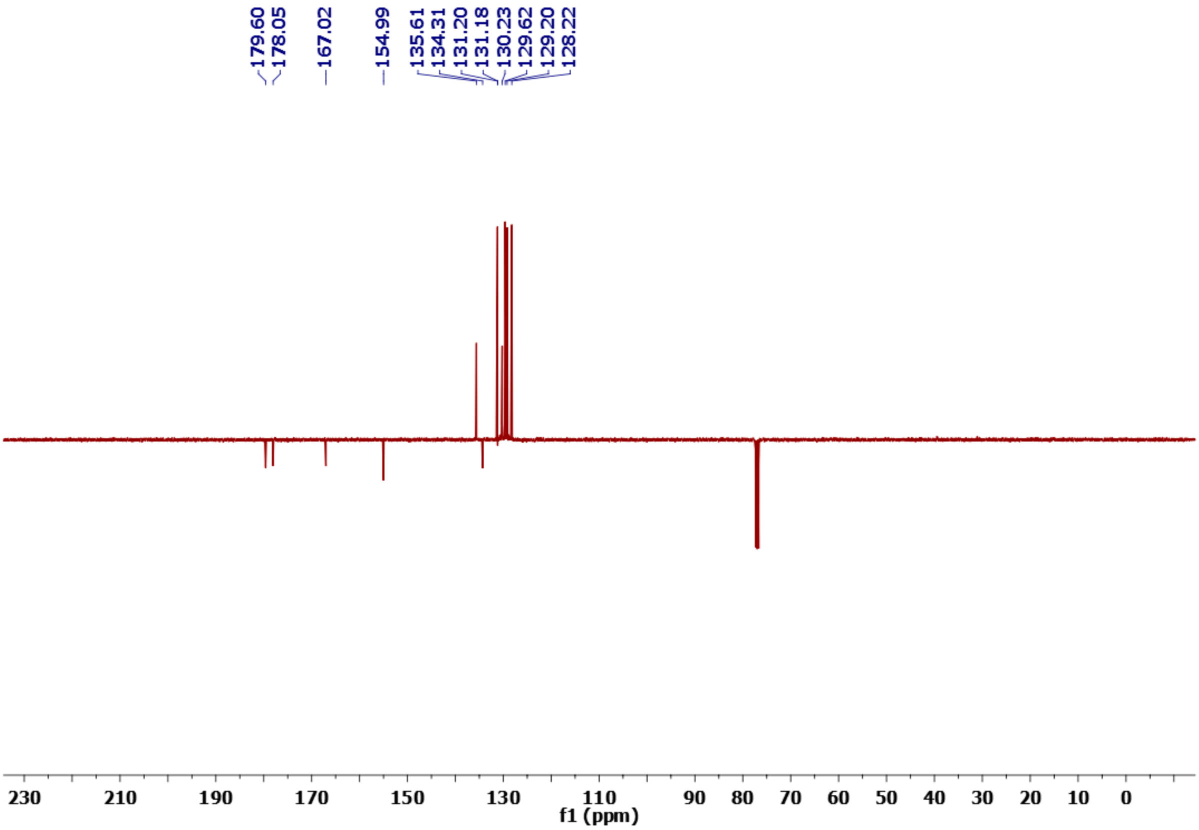

^{1}H-^{1}H-gCOSYAD NMR ($CDCl_3$) spectrum of (5-imino-3-phenyl-2,4-dithioxoimidazolidin-1-yl)(phenyl)methanone

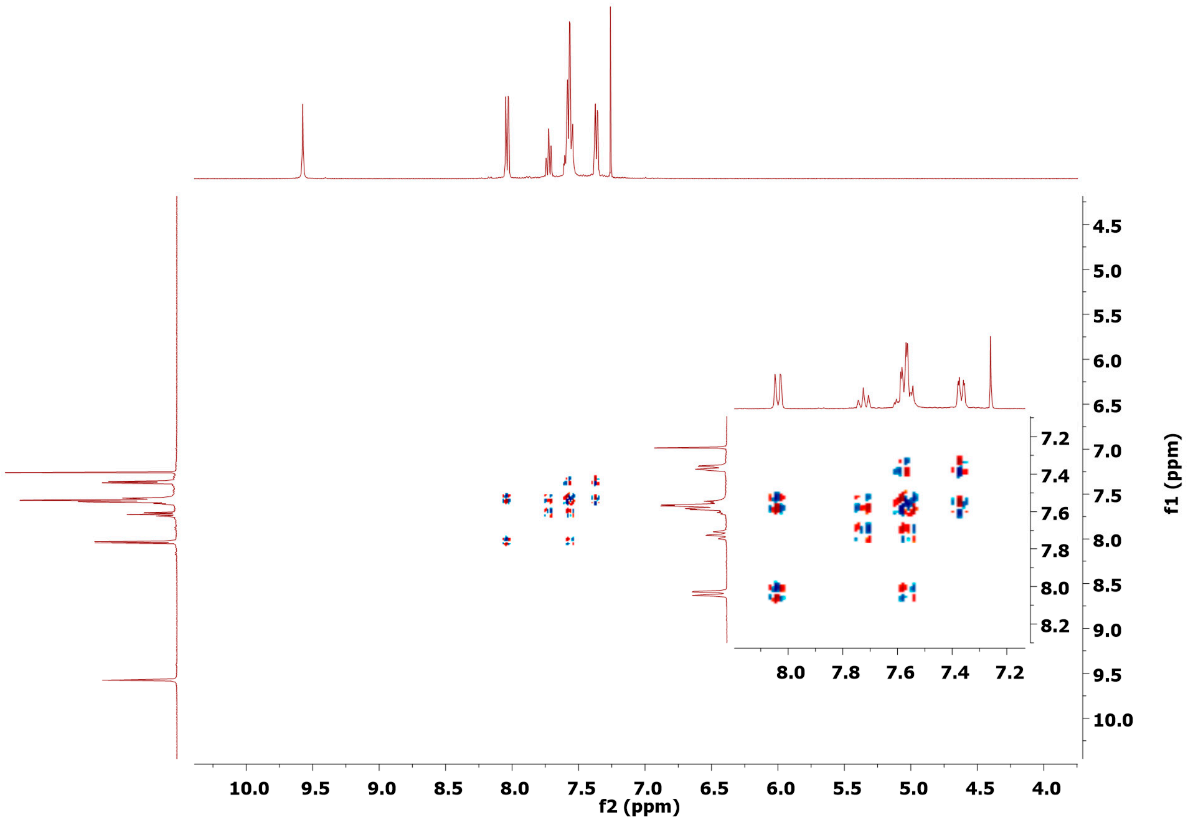

^{1}H-^{13}C-gHSQCAD NMR ($CDCl_3$) spectrum of (5-imino-3-phenyl-2,4-dithioxoimidazolidin-1-yl)(phenyl)methanone

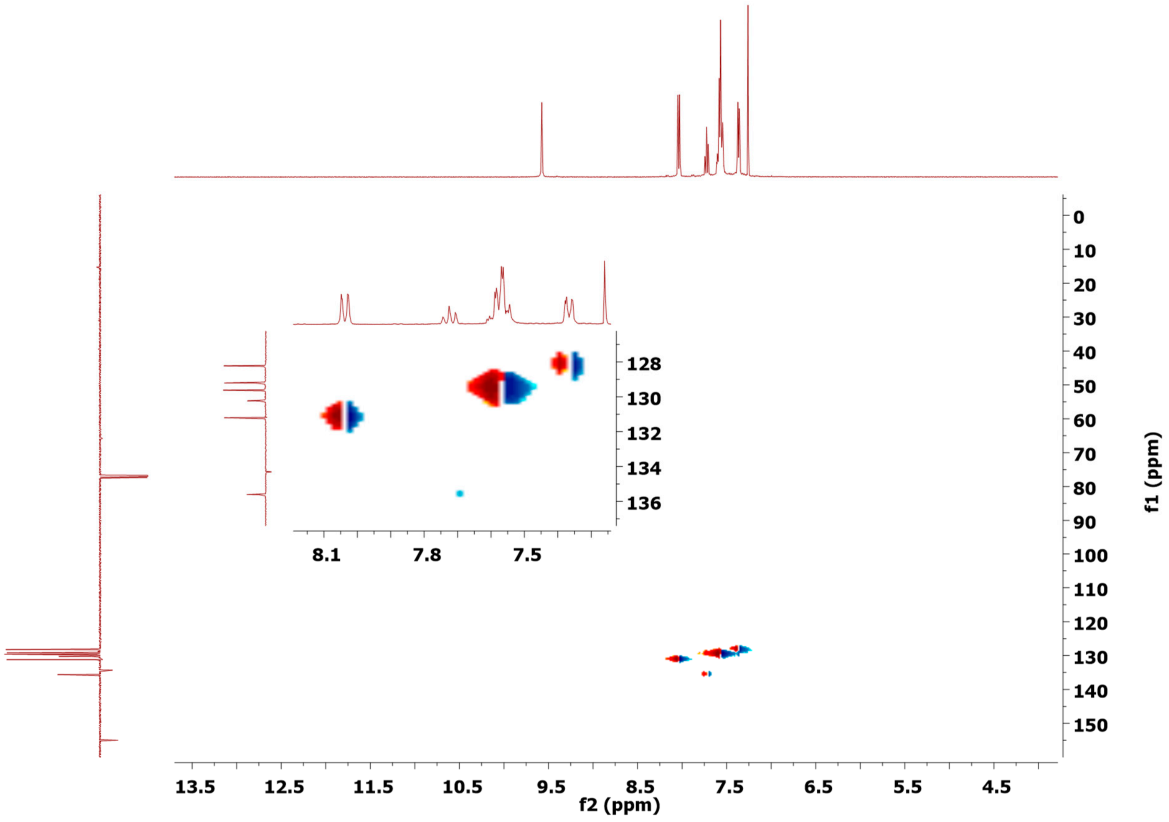

^{1}H-^{13}C-gHMBC NMR ($CDCl_3$) spectrum of (5-imino-3-phenyl-2,4-dithioxoimidazolidin-1-yl)(phenyl)methanone

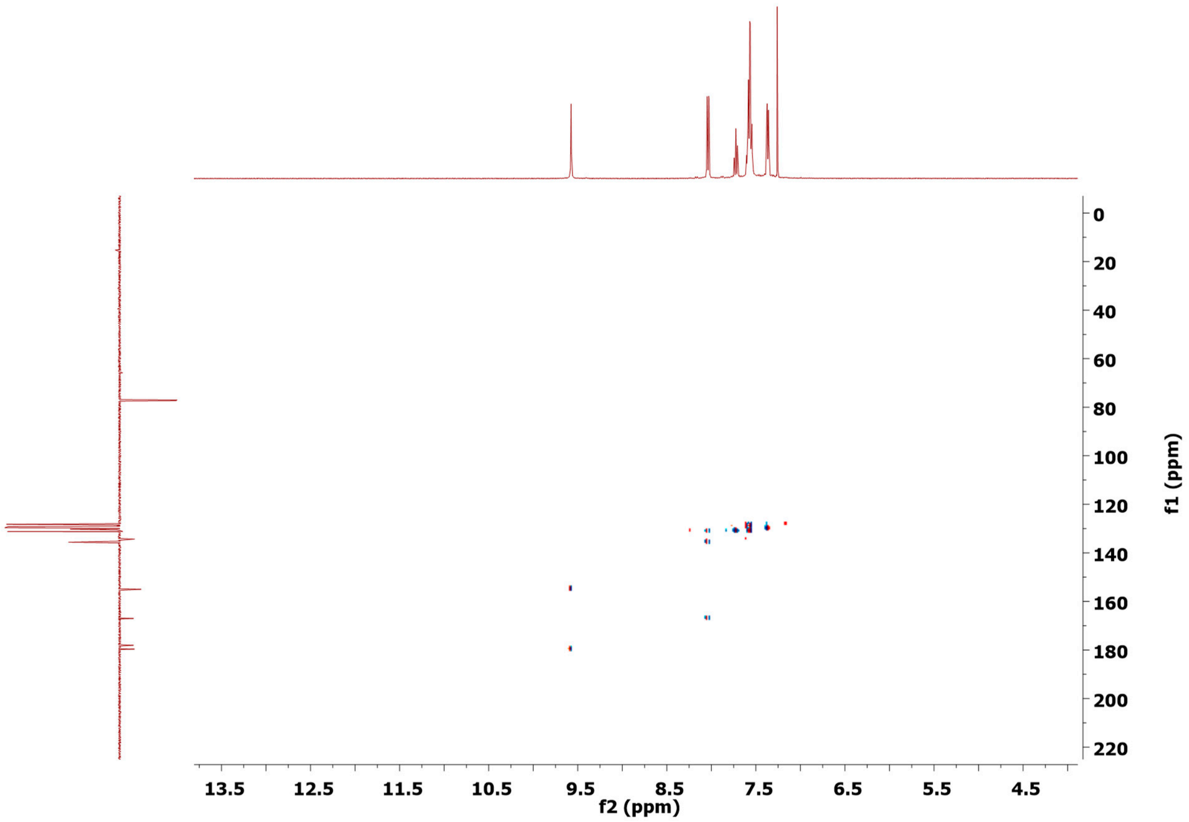

^{1}H NMR ($CDCl_3$) spectrum of 5-imino-3-phenyl-1-(4-(trifluoromethyl)phenyl)imidazolidine-2,4-dithione (18c)

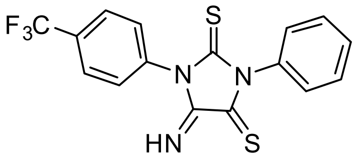

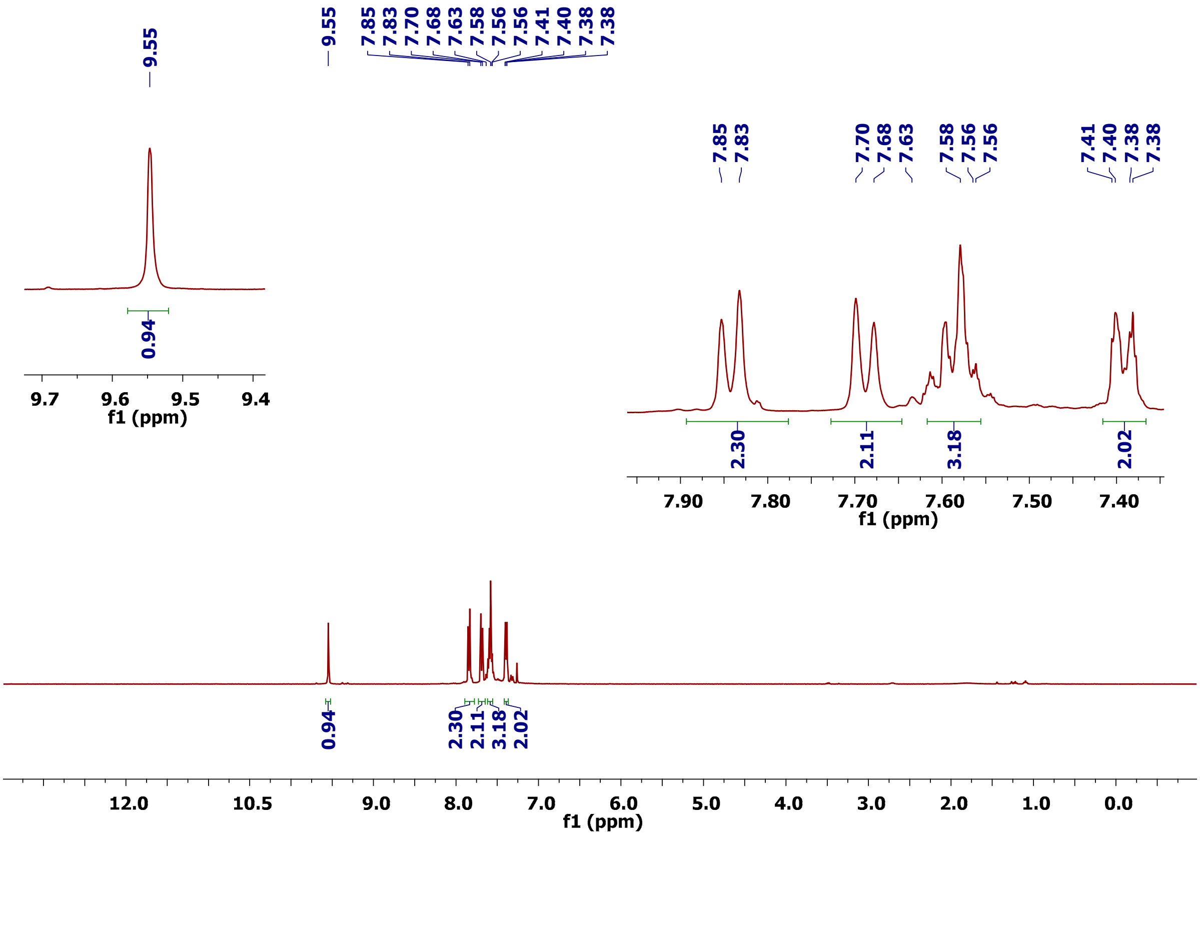

^{13}C-CRAPT NMR ($CDCl_3$) spectrum of 5-imino-3-phenyl-1-(4-(trifluoromethyl)phenyl)imidazolidine-2,4-dithione

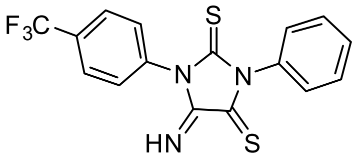

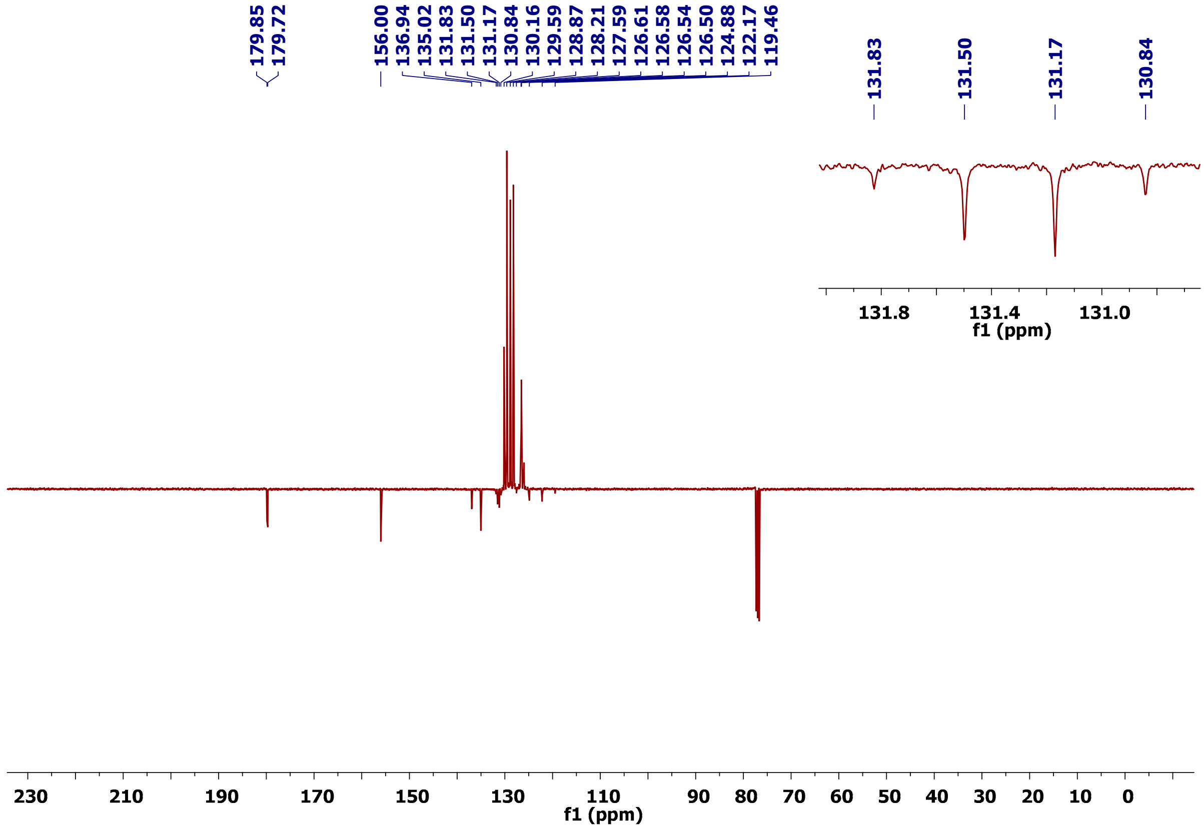

^{1}H-^{1}H-gCOSYAD NMR ($CDCl_3$) spectrum of 5-imino-3-phenyl-1-(4-(trifluoromethyl)phenyl)imidazolidine-2,4-dithione

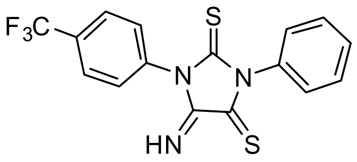

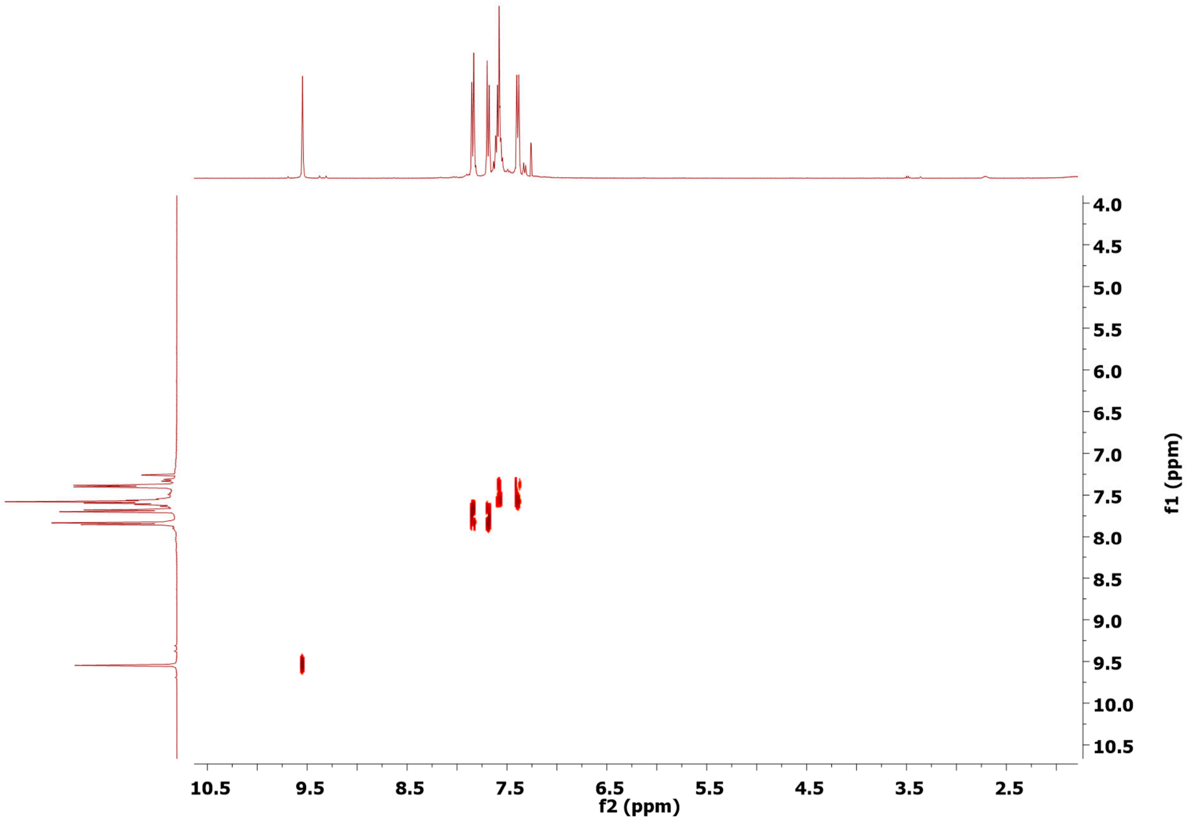

^{1}H-^{13}C-gHSQCAD NMR ($CDCl_3$) spectrum of 5-imino-3-phenyl-1-(4-(trifluoromethyl)phenyl)imidazolidine-2,4-dithione

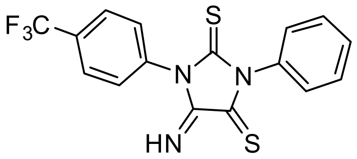

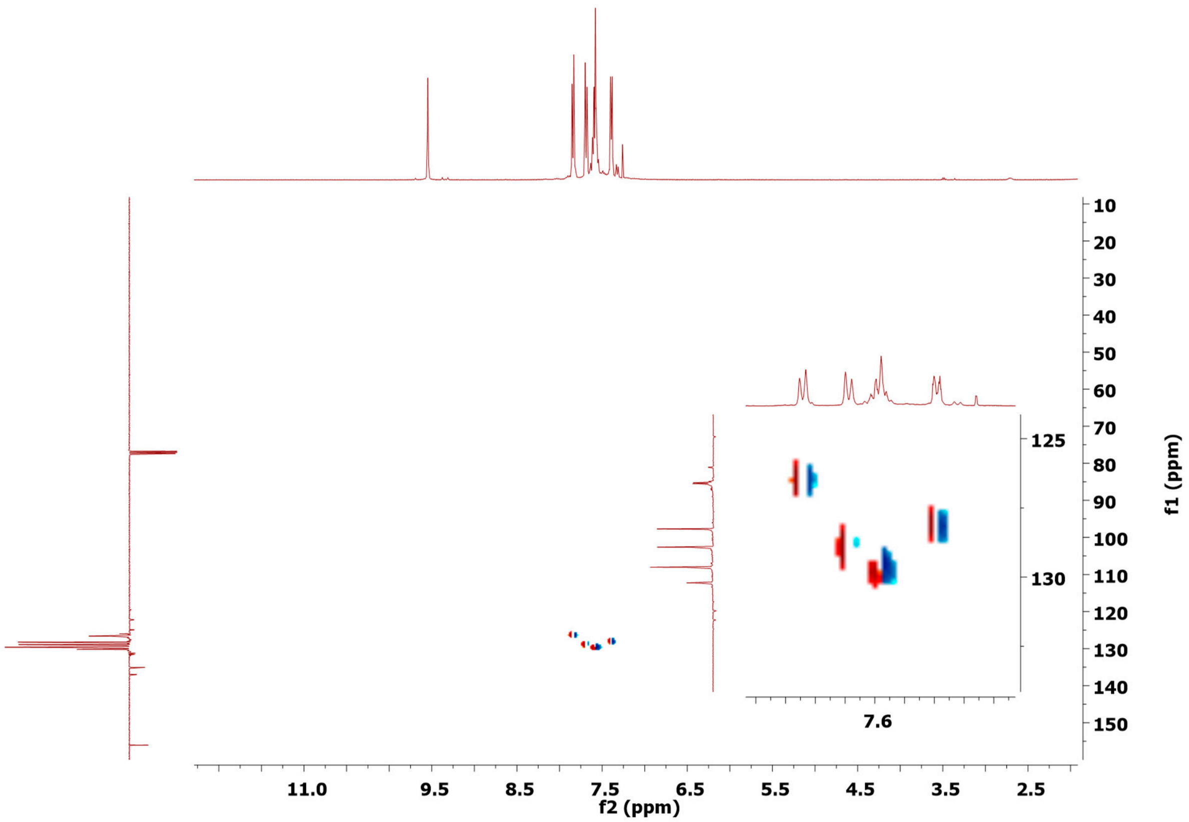

^{1}H NMR ($CDCl_3$) spectrum of 5-imino-3-phenyl-1-(p-tolyl)imidazolidine-2,4-dithione (18d)

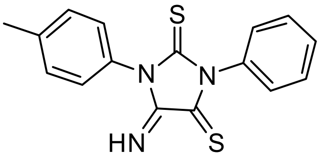

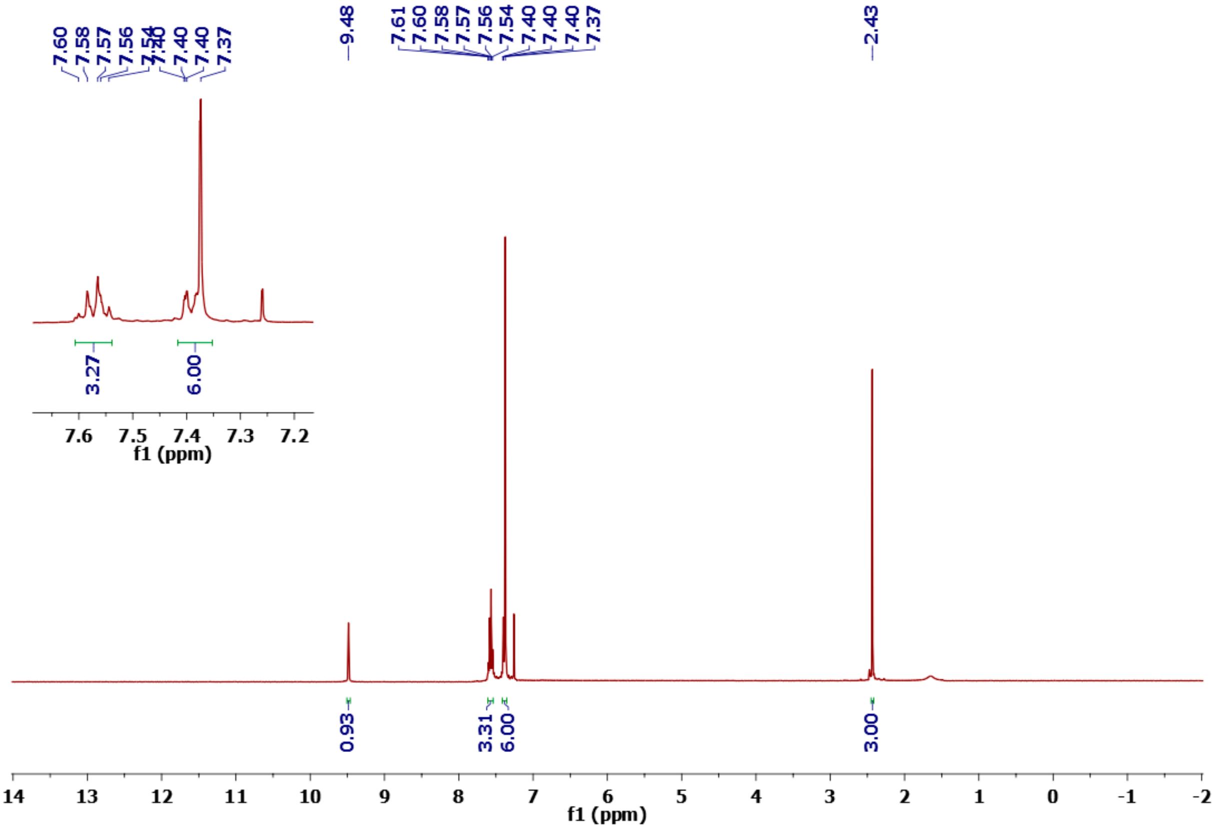

^{13}C NMR ($CDCl_3$) spectrum of 5-imino-3-phenyl-1-(p-tolyl)imidazolidine-2,4-dithione

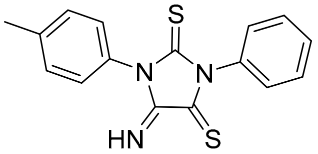

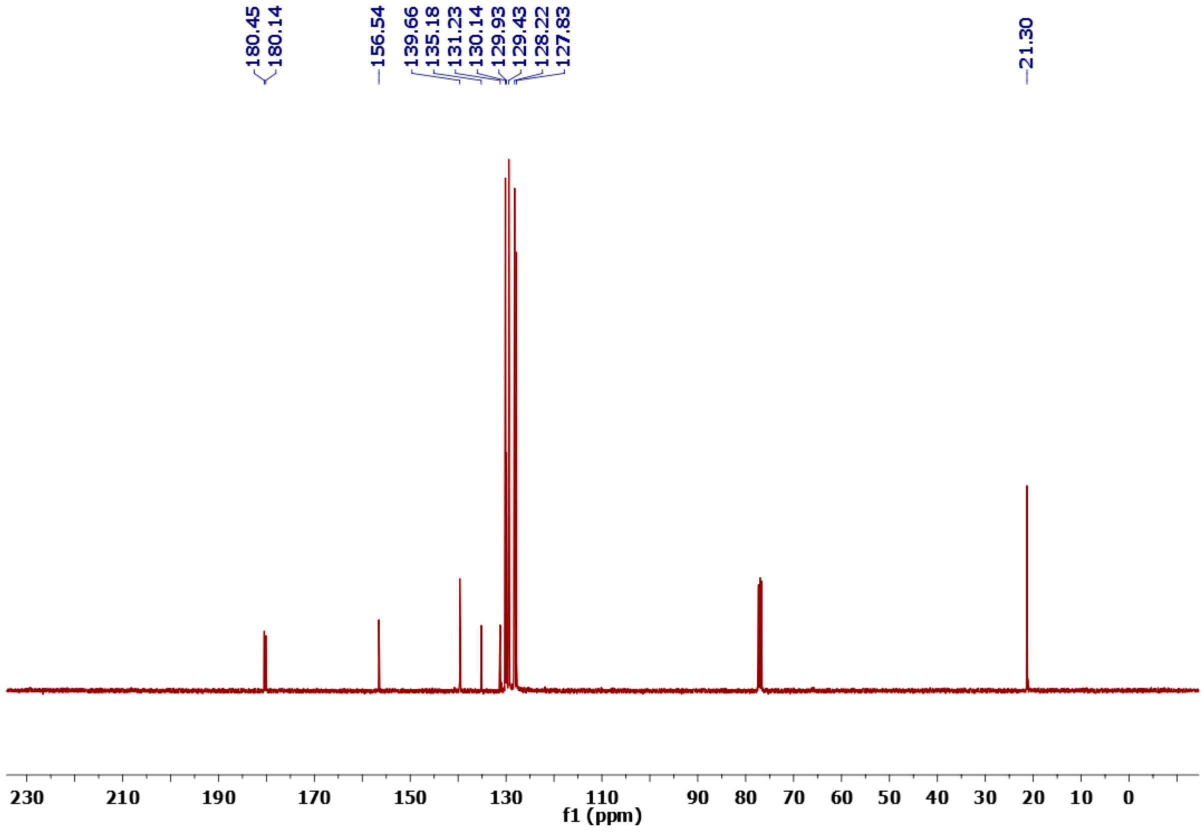

^{13}C-CRAPT NMR ($CDCl_3$) spectrum of 5-imino-3-phenyl-1-(p-tolyl)imidazolidine-2,4-dithione

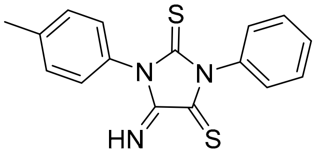

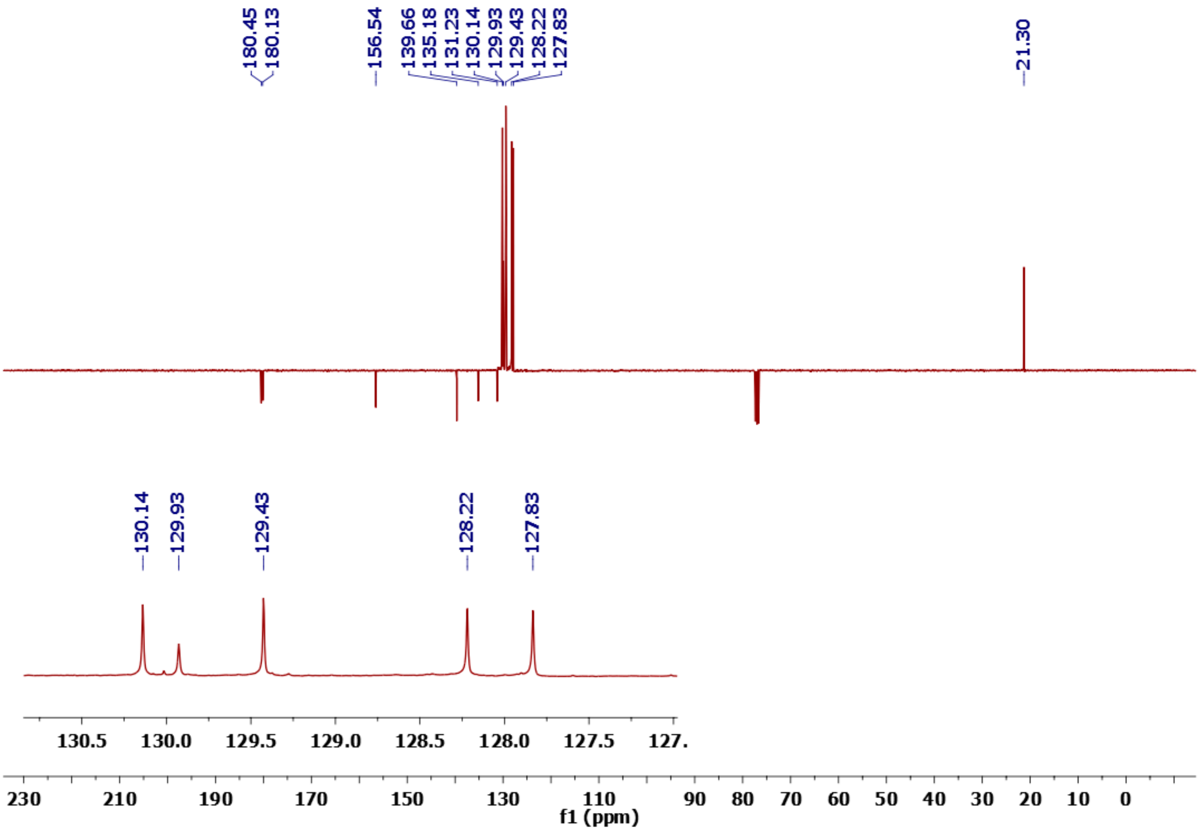

^1H-^1H-gCOSYAD NMR ($CDCl_3$) spectrum of 5-imino-3-phenyl-1-(p-tolyl)imidazolidine-2,4-dithione

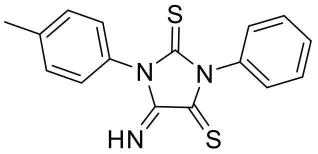

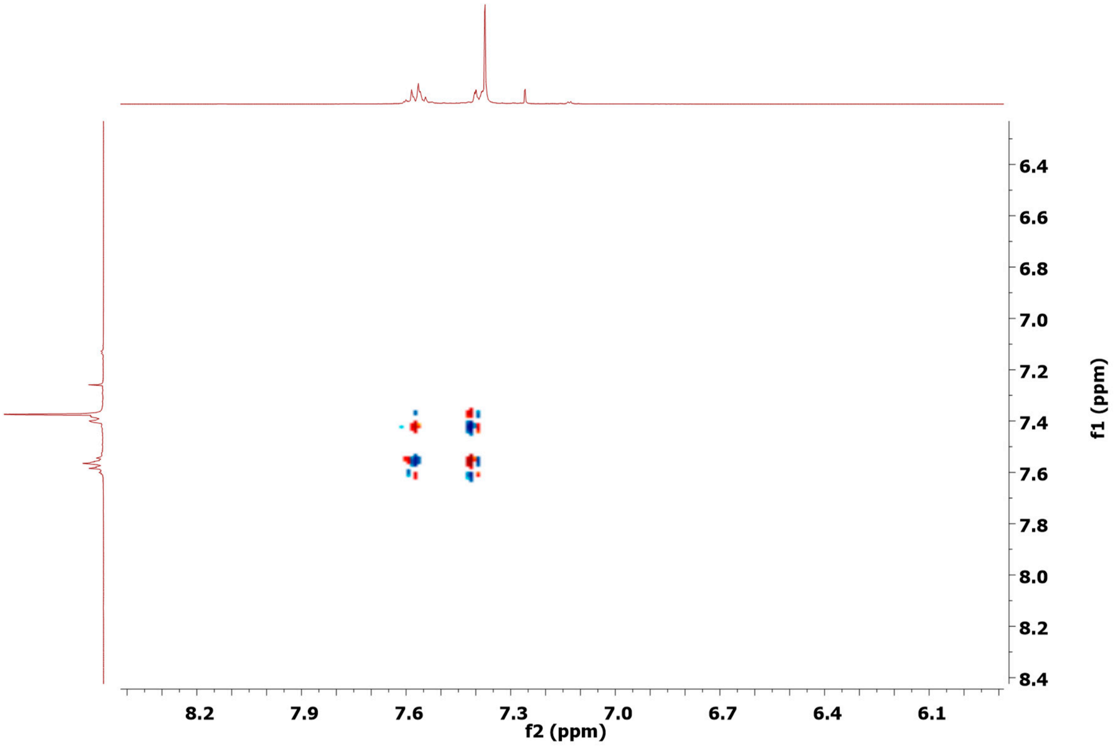

^{1}H-^{13}C-gHSQCAD NMR ($CDCl_3$) spectrum of 5-imino-3-phenyl-1-(p-tolyl)imidazolidine-2,4-dithione

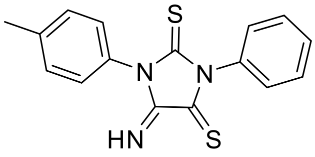

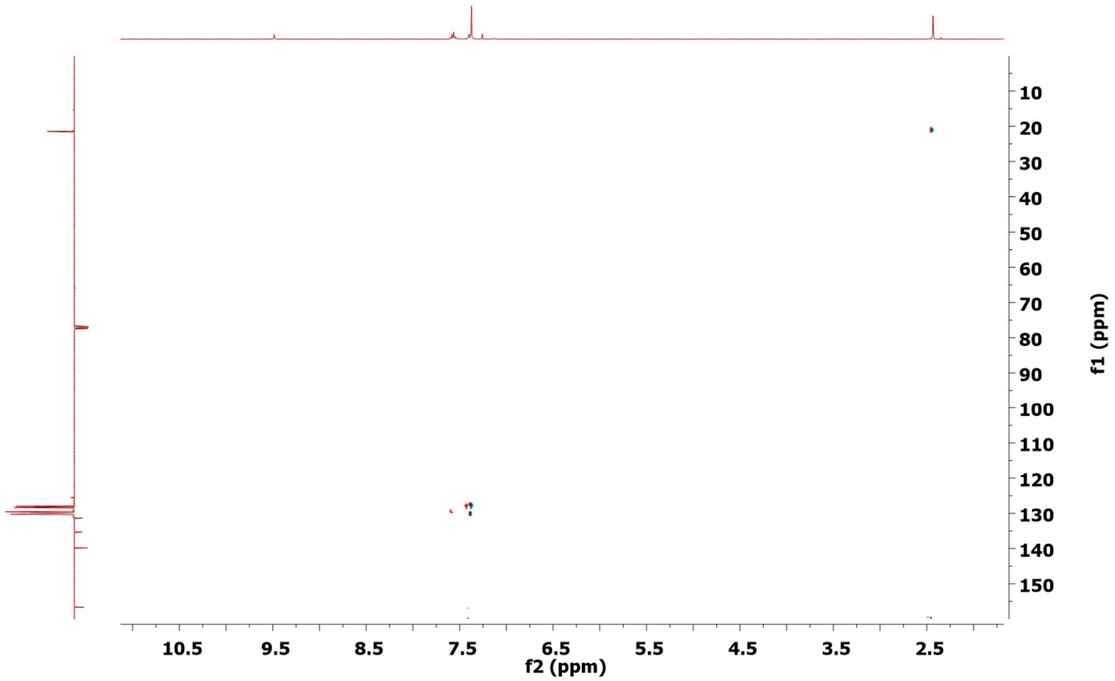

^{1}H-^{13}C-gHMBC NMR ($CDCl_3$) spectrum of 5-imino-3-phenyl-1-(p-tolyl)imidazolidine-2,4-dithione

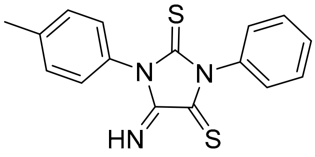

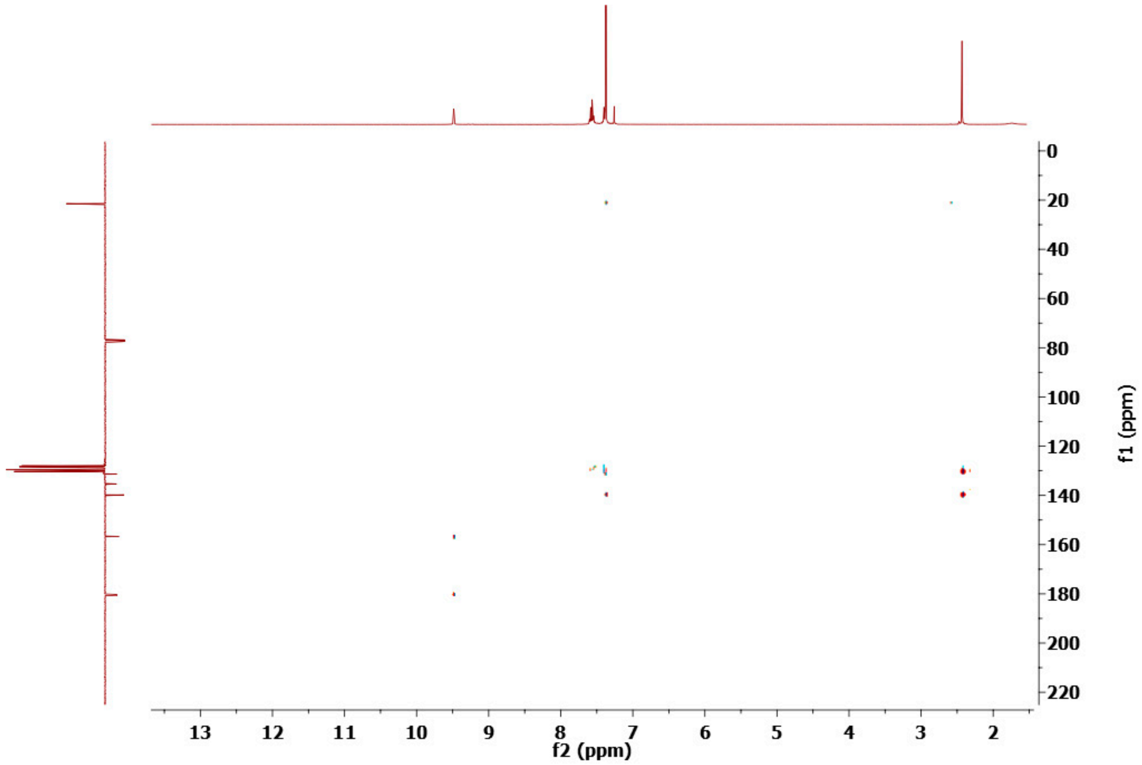

^{1}H NMR (DMSO-d$_6$) spectrum of 1-(2-fluorophenyl)-5-imino-3-phenylimidazolidine-2,4-dithione (18e)

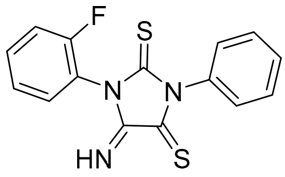

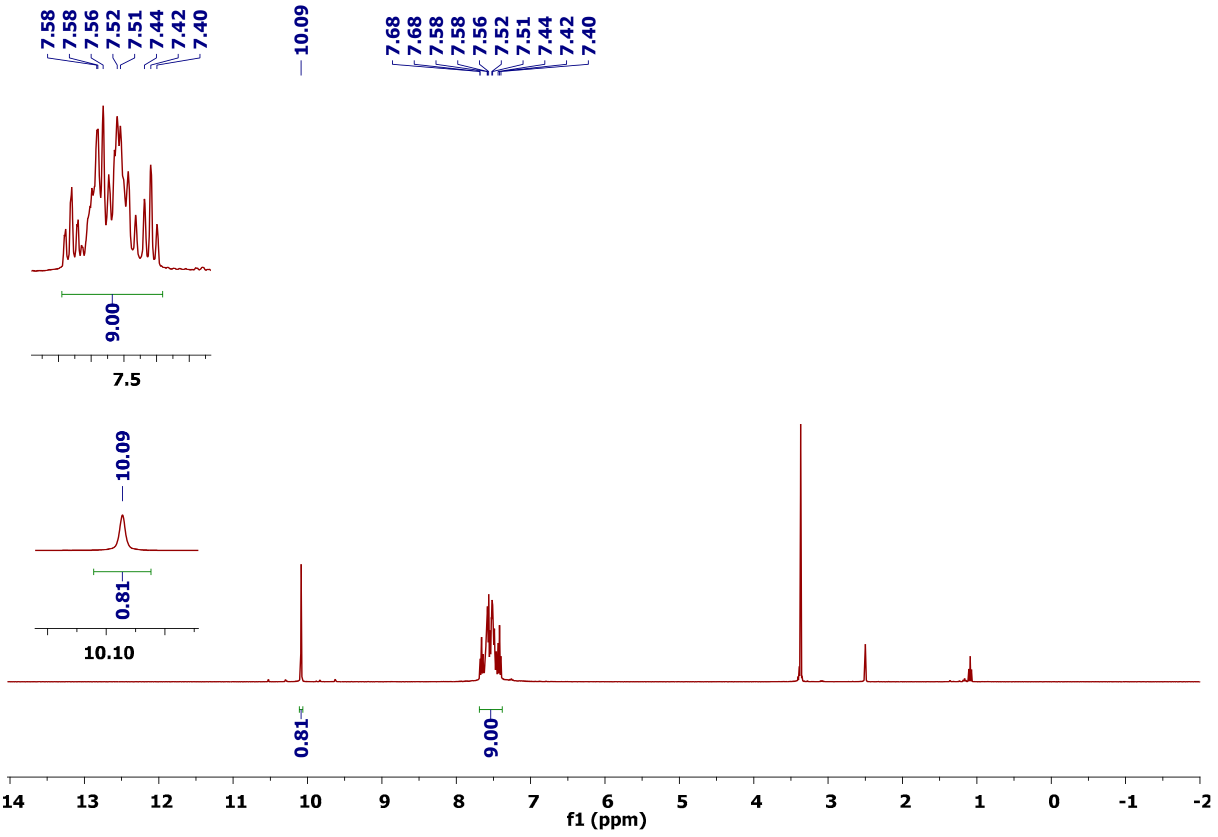

^{13}C NMR (DMSO-d_6) spectrum of 1-(2-fluorophenyl)-5-imino-3-phenylimidazolidine-2,4-dithione

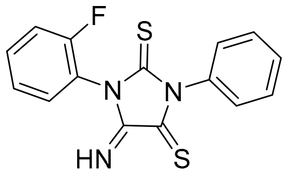

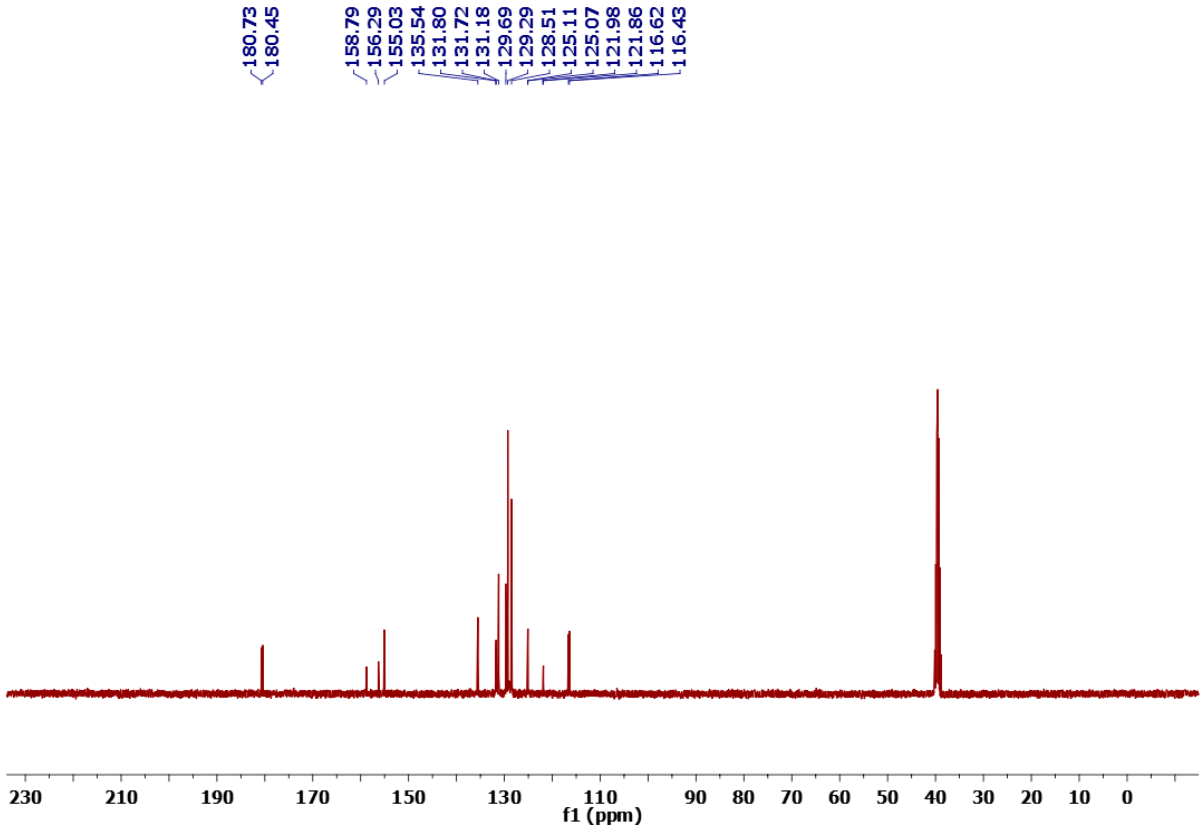

^{13}C-CRAPT NMR (DMSO-d_6) spectrum of 1-(2-fluorophenyl)-5-imino-3-phenylimidazolidine-2,4-dithione

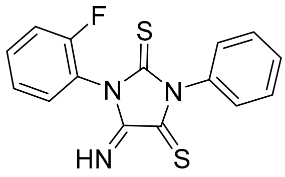

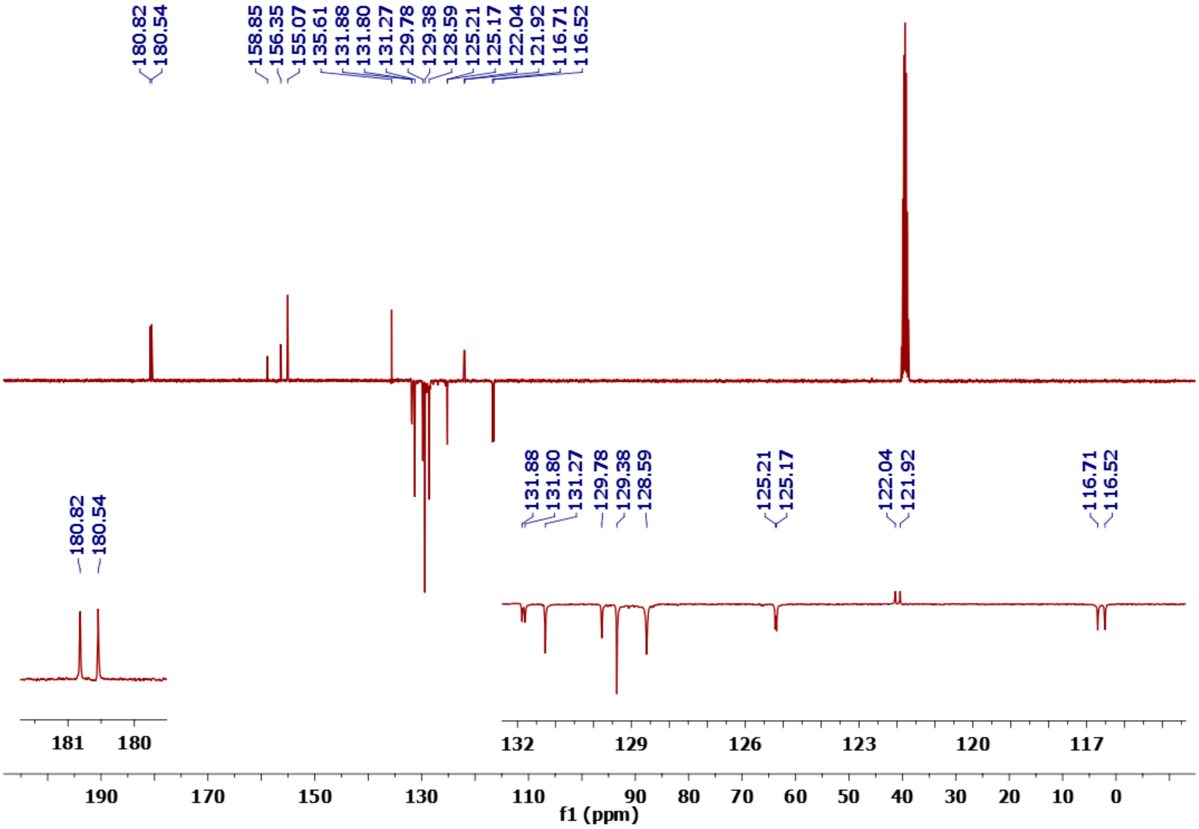

^{1}H NMR ($CDCl_3$) spectrum of 1-(4-chlorophenyl)-5-imino-3-phenylimidazolidine-2,4-dithione (18f)

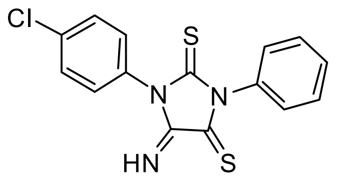

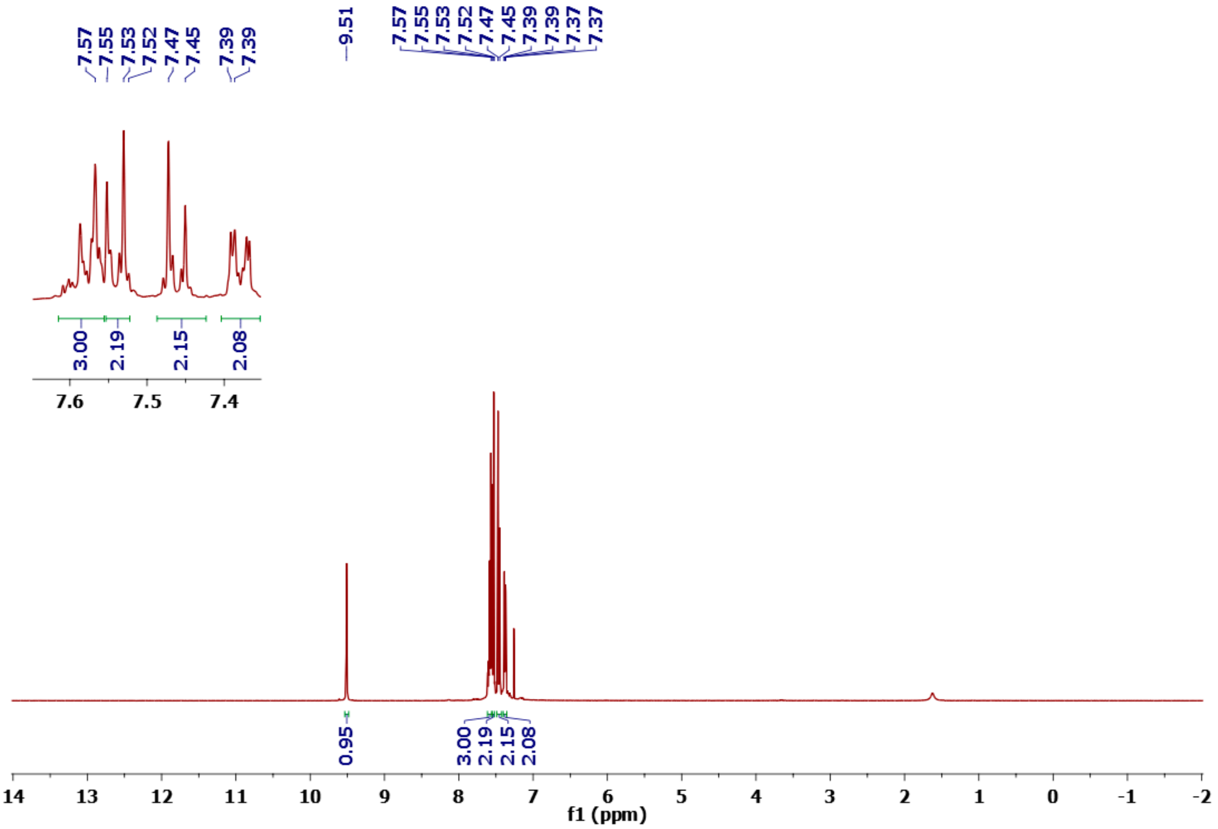

^{13}C NMR ($CDCl_3$) spectrum of 1-(4-chlorophenyl)-5-imino-3-phenylimidazolidine-2,4-dithione

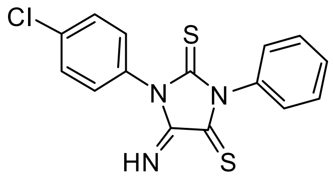

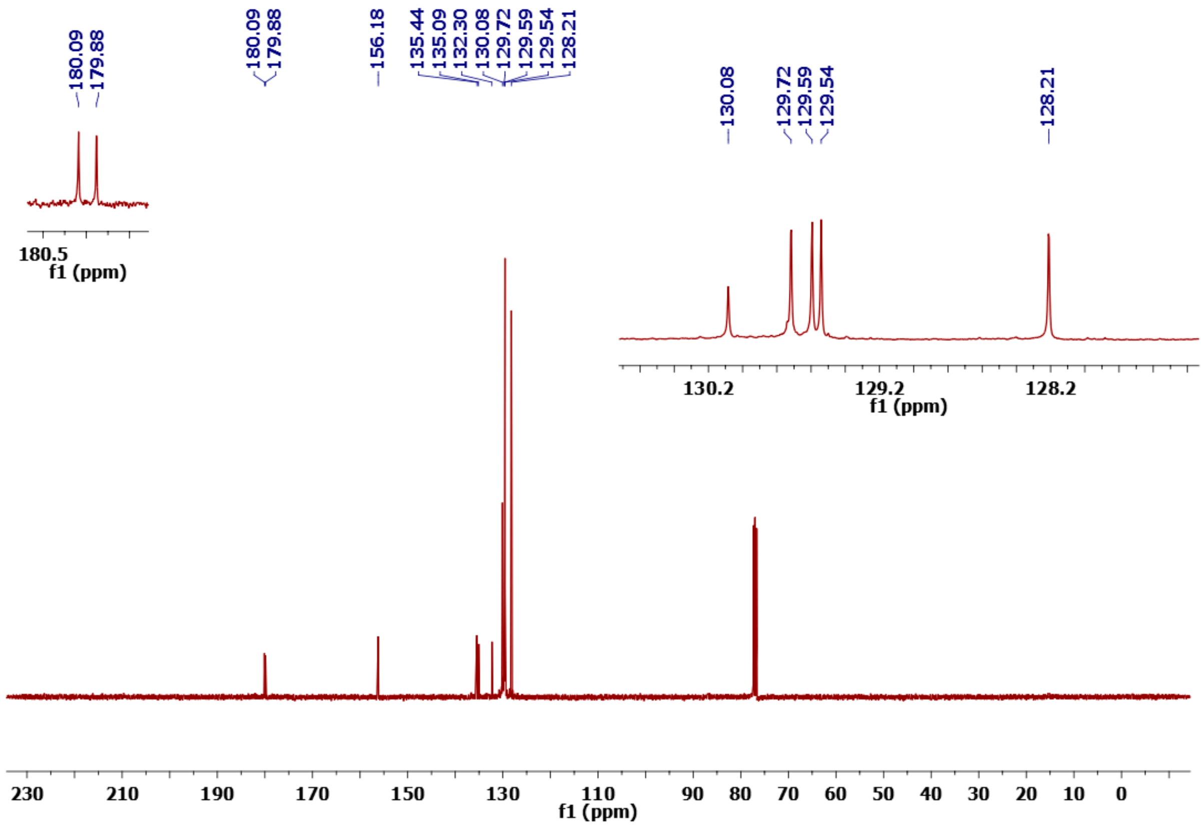

^{13}C-CRAPT NMR ($CDCl_3$) spectrum of 1-(4-chlorophenyl)-5-imino-3-phenylimidazolidine-2,4-dithione

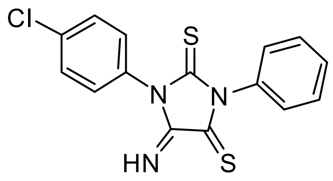

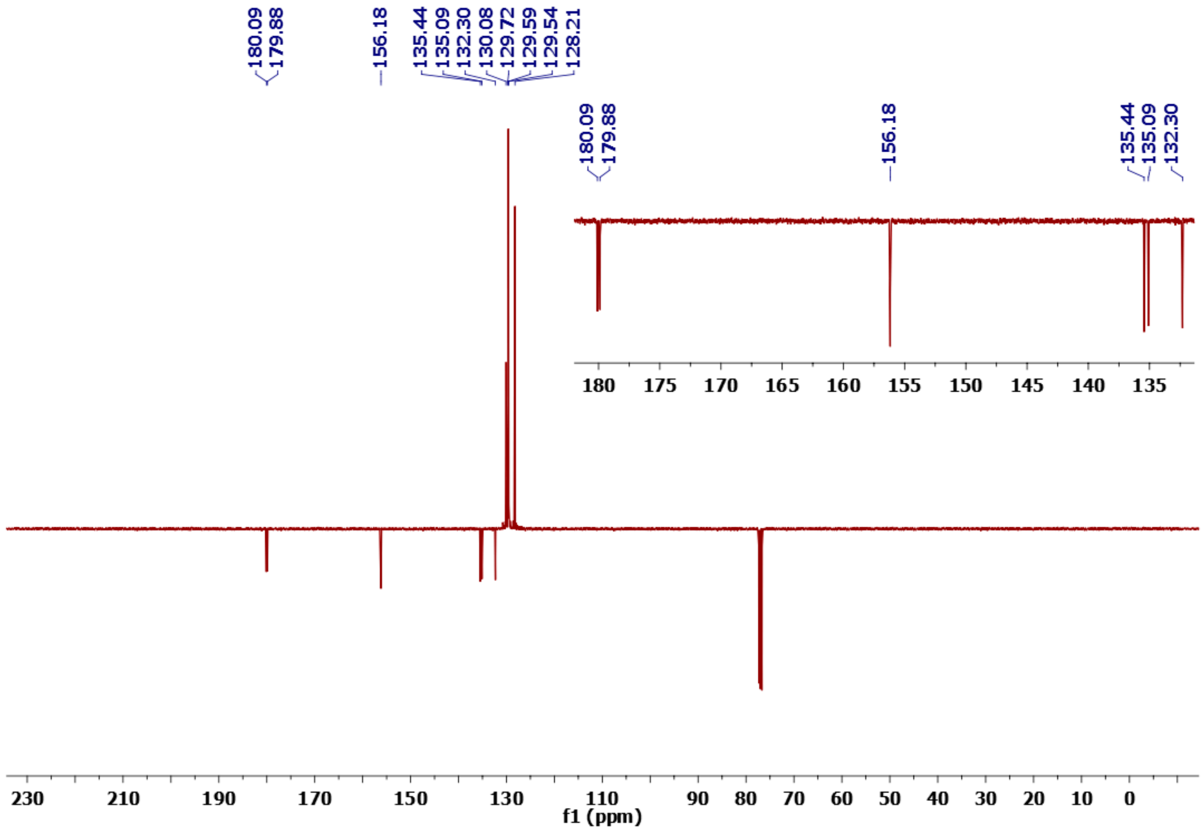

^{1}H-^{1}H-gCOSYAD NMR ($CDCl_3$) spectrum of 1-(4-chlorophenyl)-5-imino-3-phenylimidazolidine-2,4-dithione

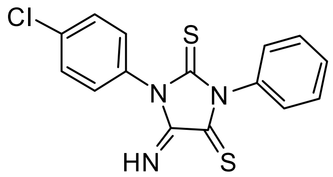

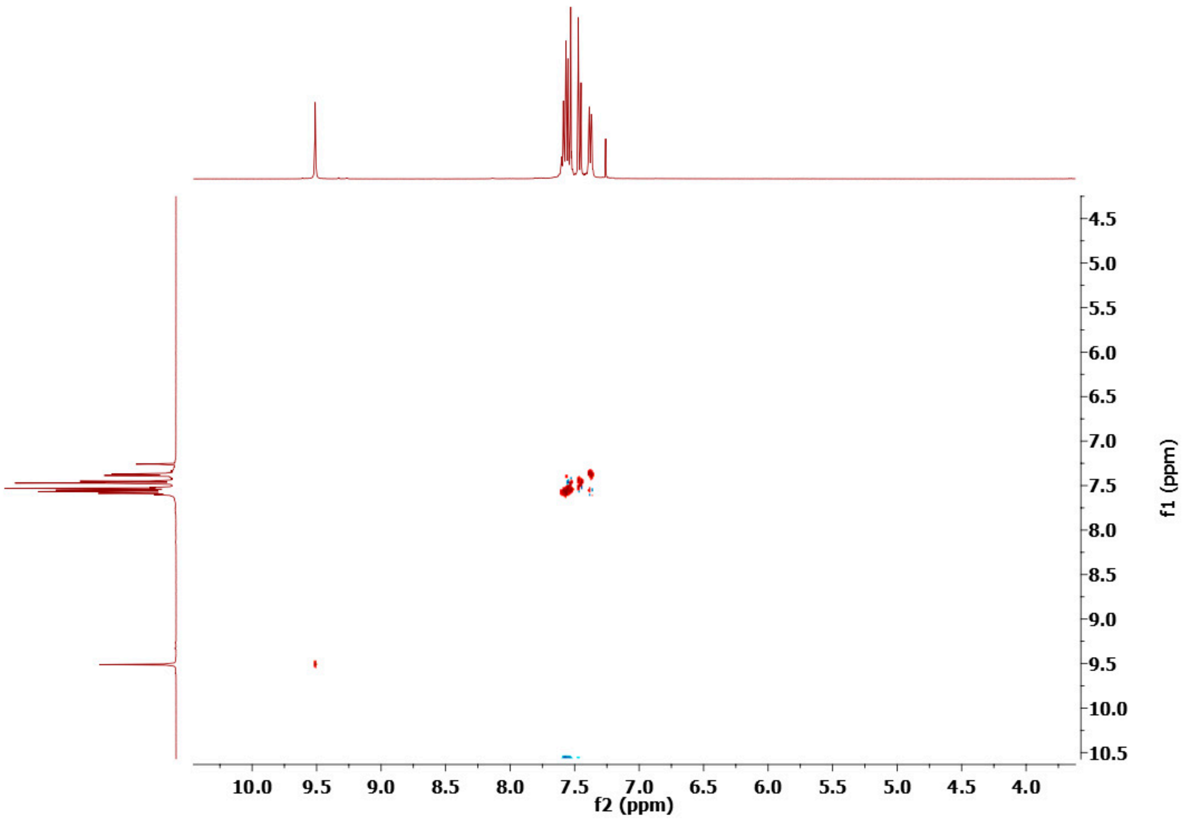

^{1}H-^{13}C-gHSQCAD NMR ($CDCl_3$) spectrum of 1-(4-chlorophenyl)-5-imino-3-phenylimidazolidine-2,4-dithione

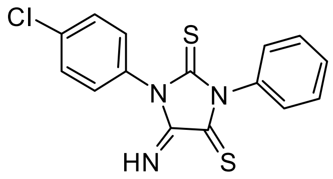

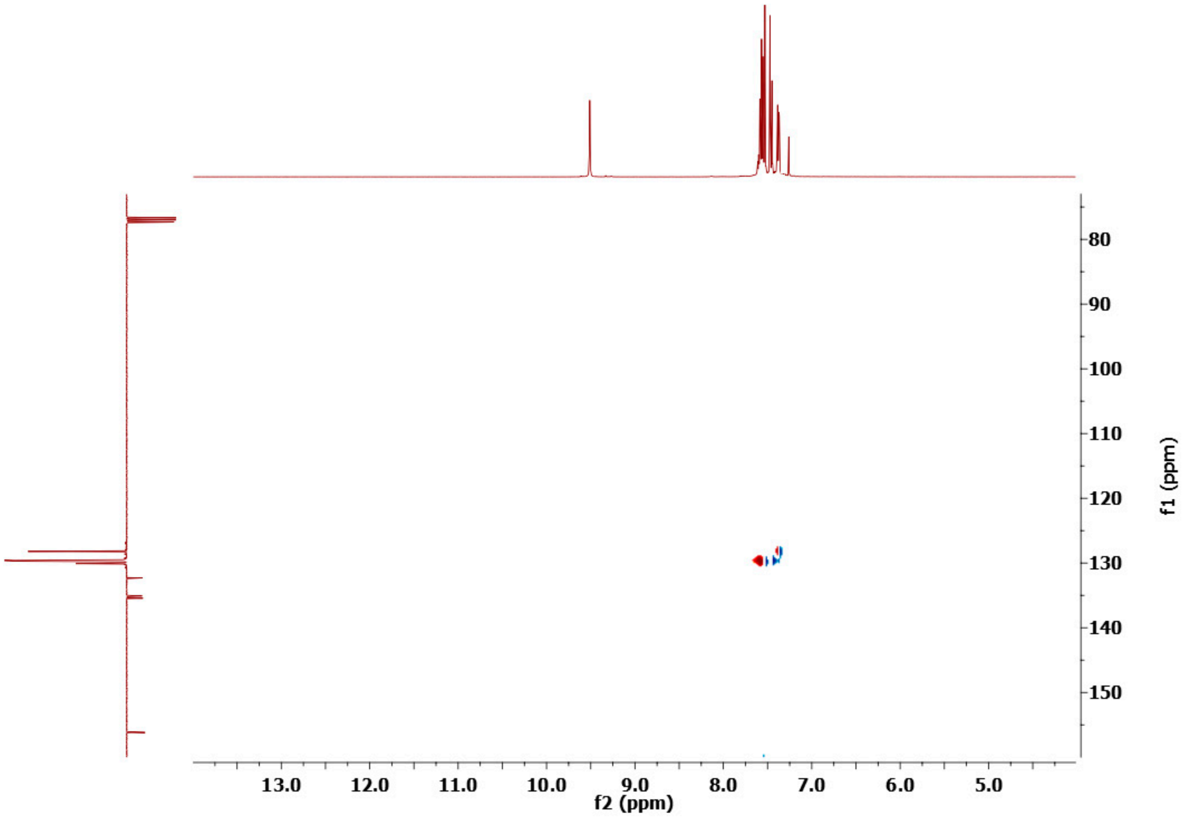

^{1}H-^{13}C-gHMBC NMR ($CDCl_3$) spectrum of 1-(4-chlorophenyl)-5-imino-3-phenylimidazolidine-2,4-dithione

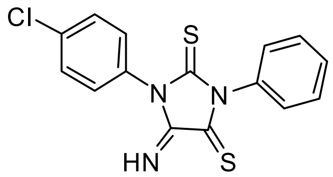

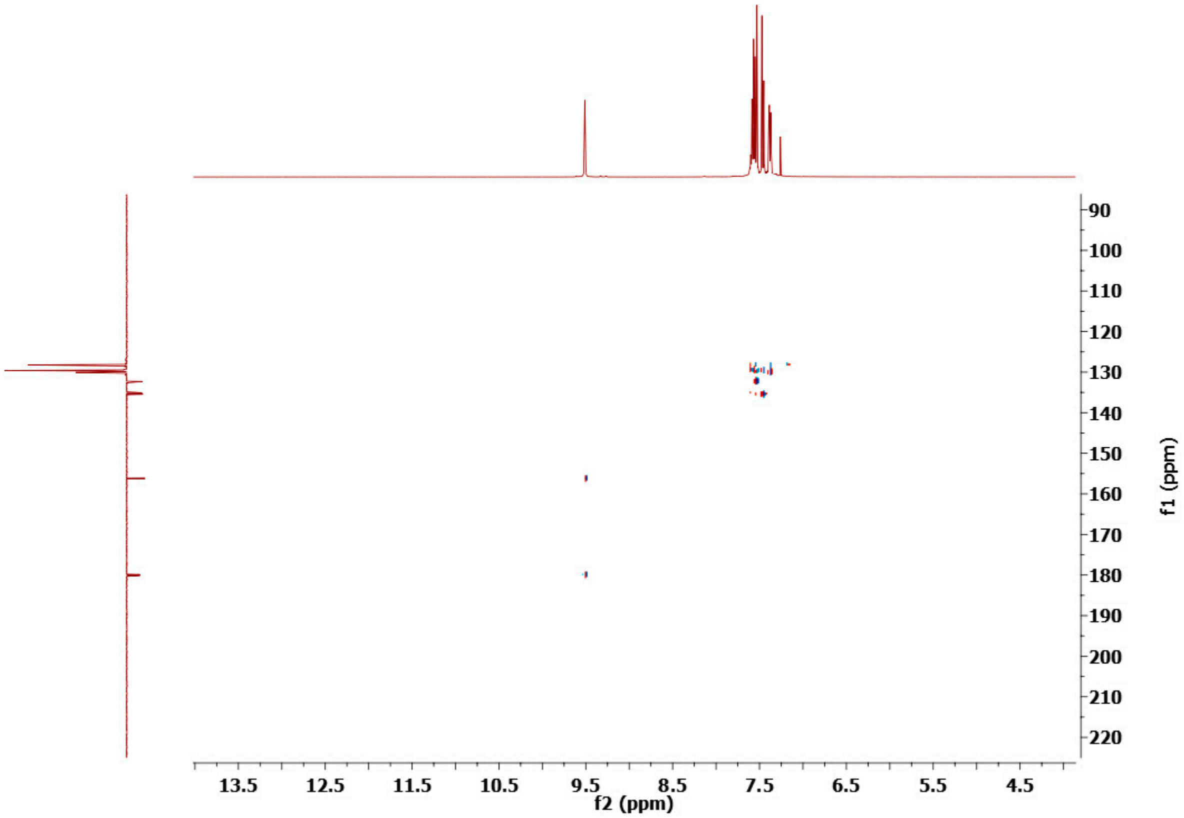

^{1}H NMR ($CDCl_3$) spectrum of 1-(4-fluorophenyl)-5-imino-3-phenylimidazolidine-2,4-dithione (18g)

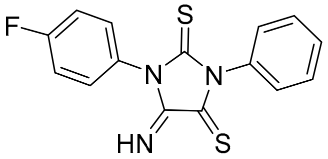

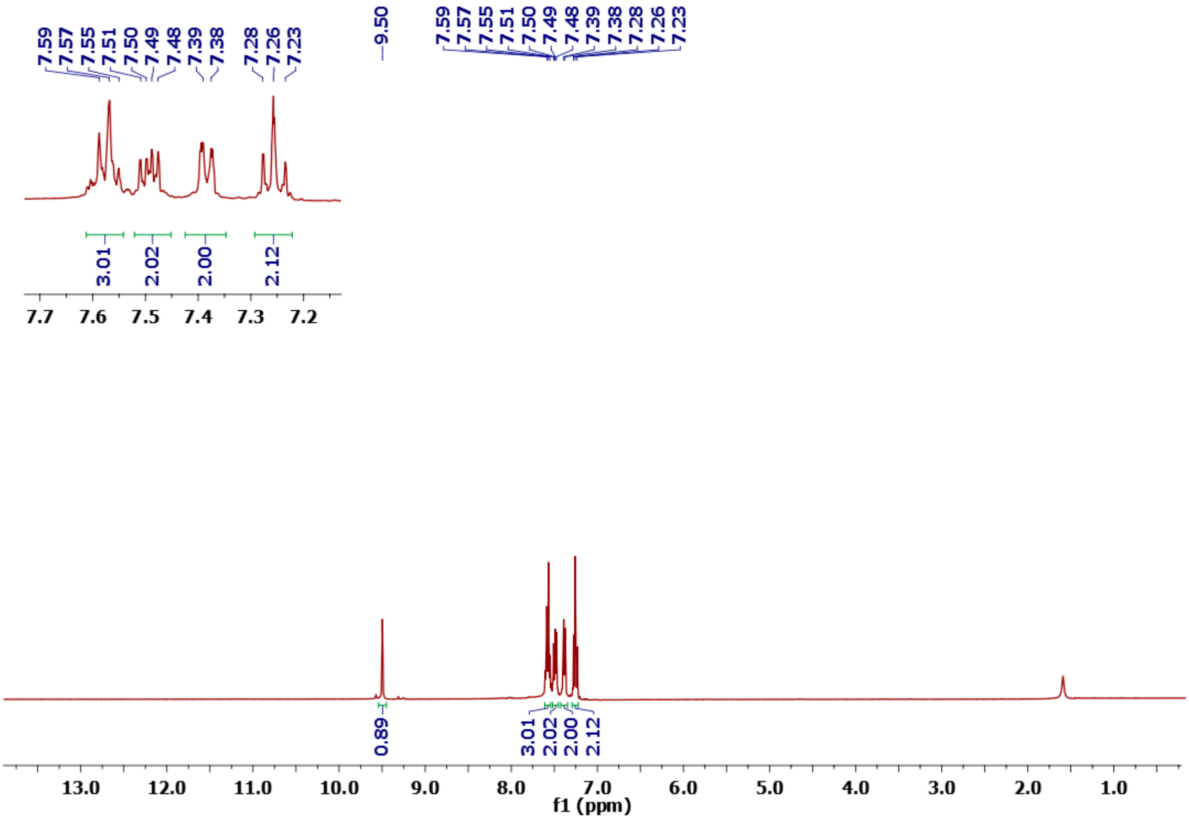

^{13}C NMR ($CDCl_3$) spectrum of 1-(4-fluorophenyl)-5-imino-3-phenylimidazolidine-2,4-dithione

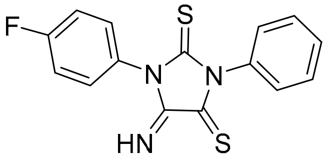

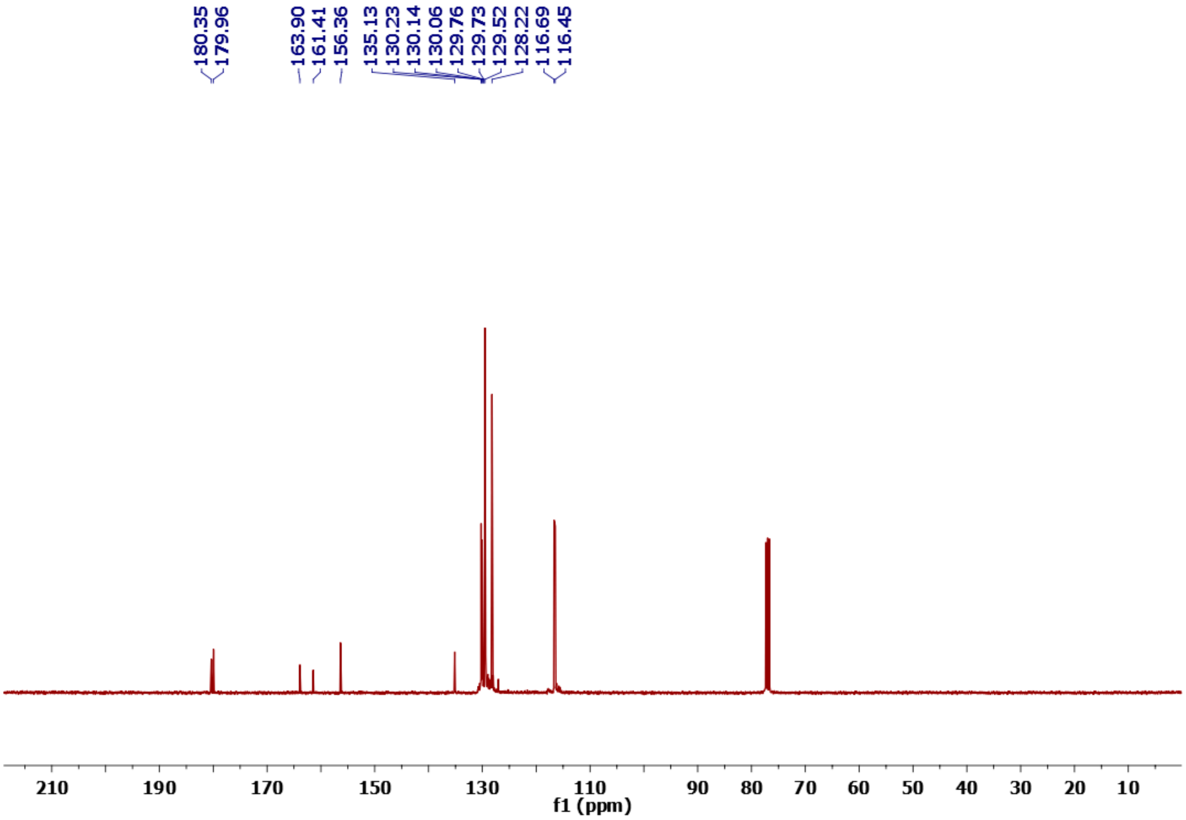

^{13}C-CRAPT NMR ($CDCl_3$) spectrum of 1-(4-fluorophenyl)-5-imino-3-phenylimidazolidine-2,4-dithione

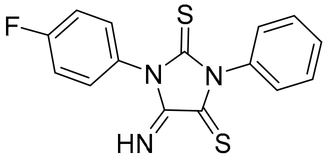

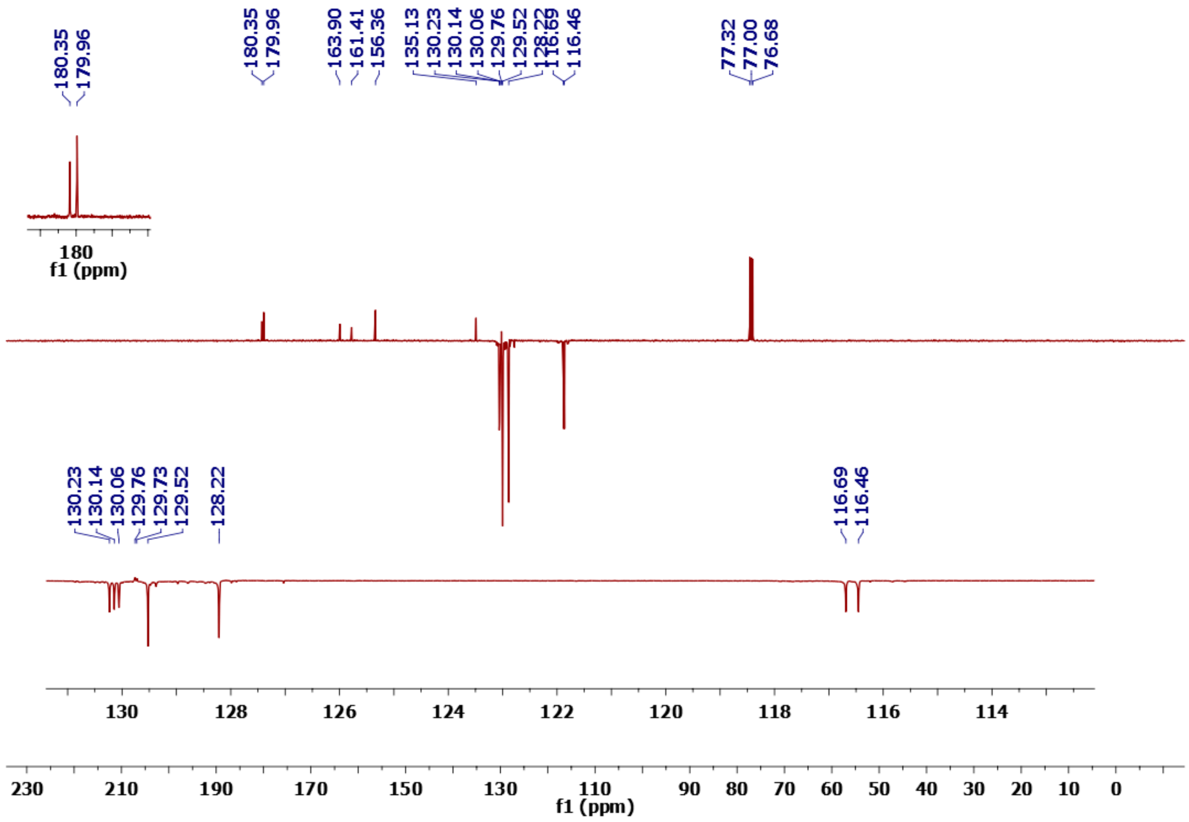

^{1}H-^{1}H-gCOSYAD NMR ($CDCl_3$) spectrum of 1-(4-fluorophenyl)-5-imino-3-phenylimidazolidine-2,4-dithione

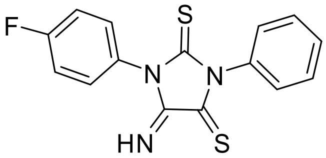

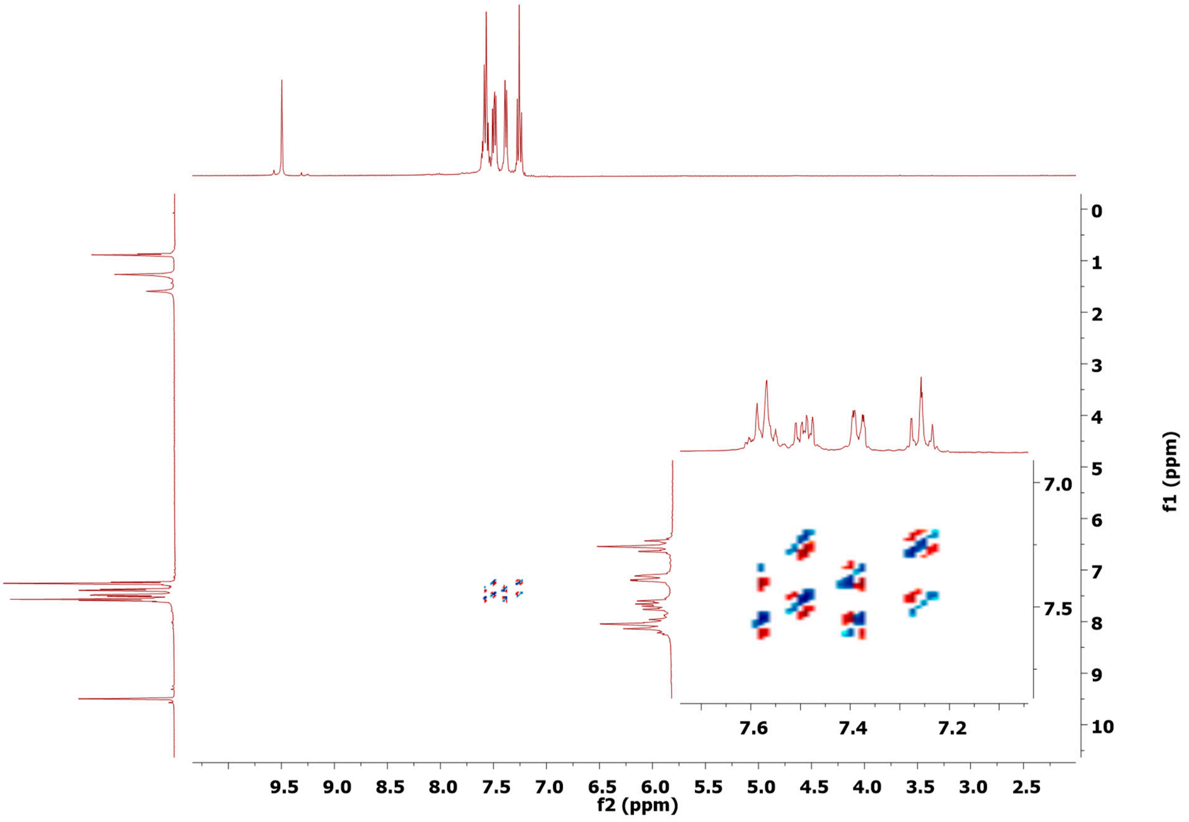

^{1}H-^{13}C-gHSQCAD NMR ($CDCl_3$) spectrum of 1-(4-fluorophenyl)-5-imino-3-phenylimidazolidine-2,4-dithione

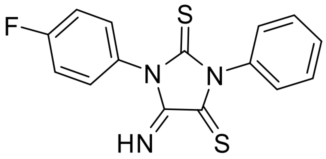

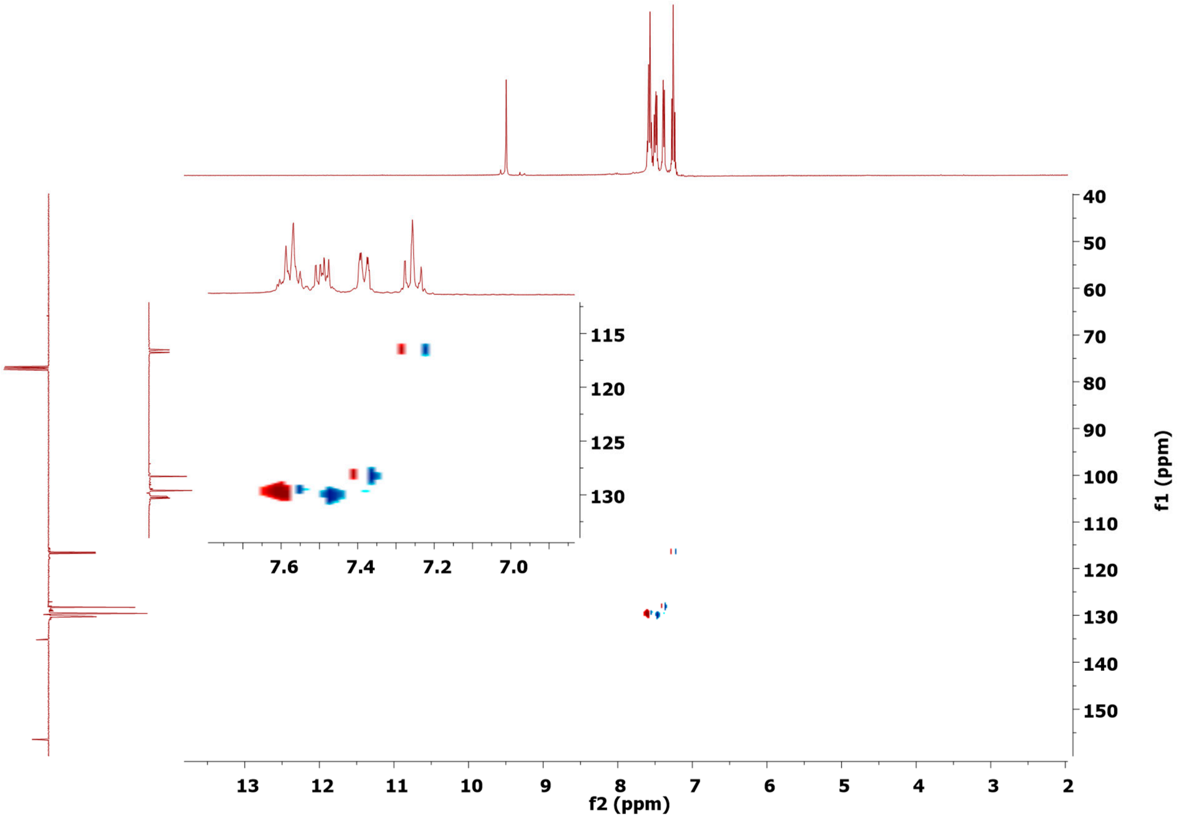

^{1}H-^{13}C-gHMBC NMR ($CDCl_3$) spectrum of 1-(4-fluorophenyl)-5-imino-3-phenylimidazolidine-2,4-dithione

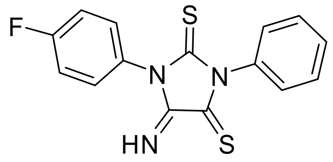

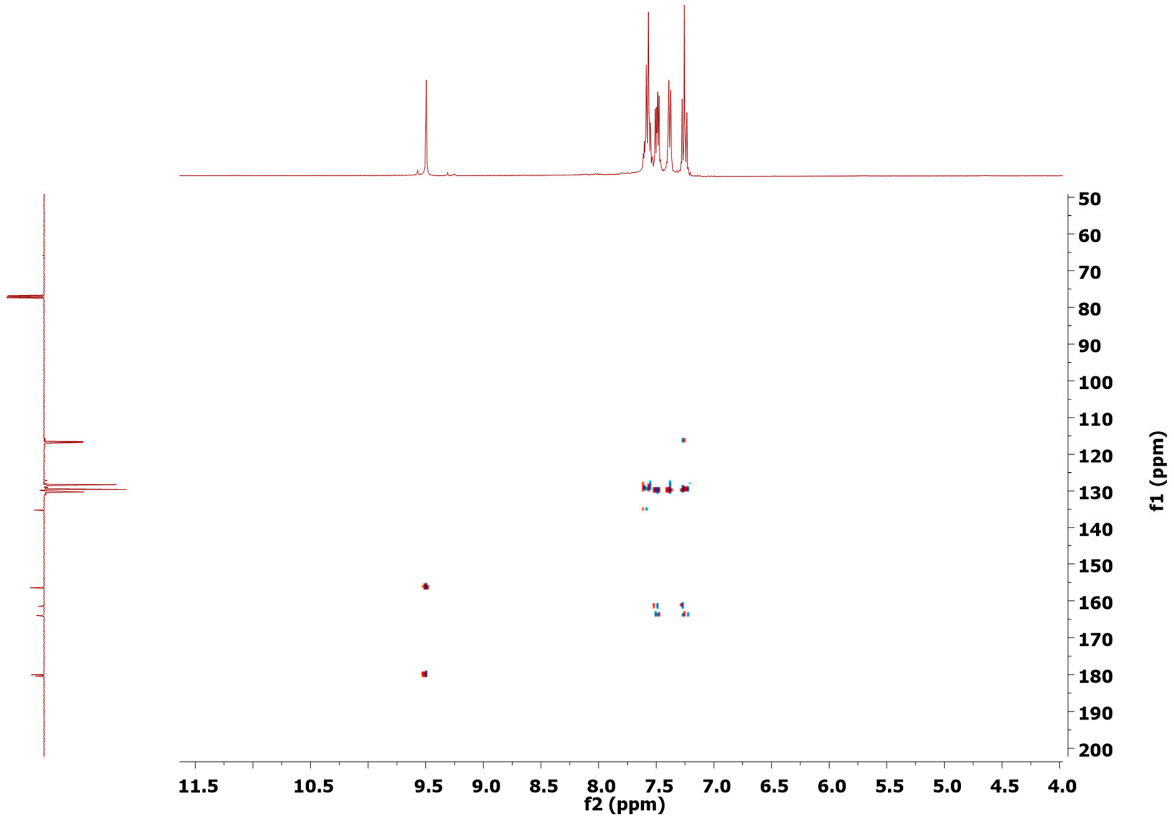

^{1}H-NMR ($CDCl_3$) spectrum of 5-imino-1-(4-nitrophenyl)-3-phenylimidazolidine-2,4-dithione (18h)

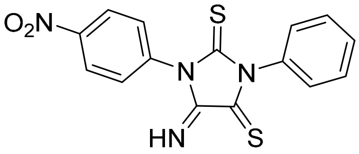

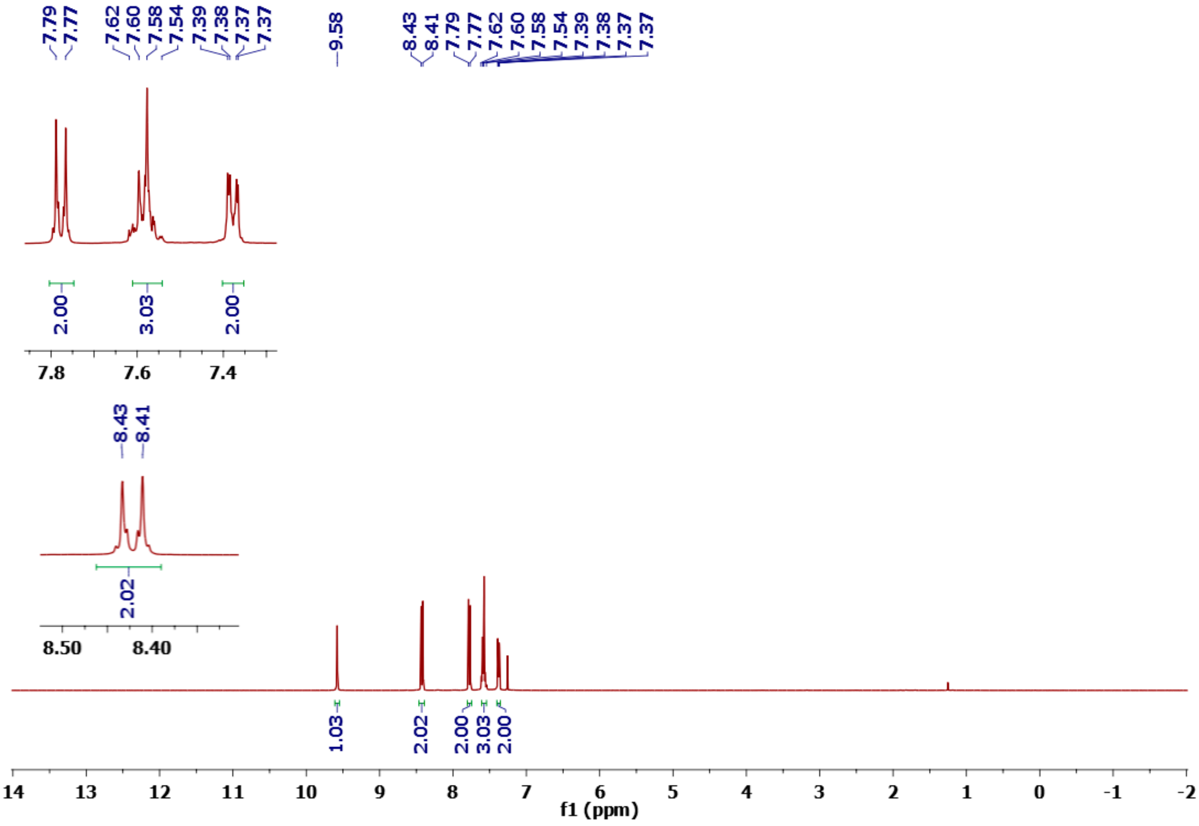

^{13}C NMR ($CDCl_3$) spectrum of 5-imino-1-(4-nitrophenyl)-3-phenylimidazolidine-2,4-dithione

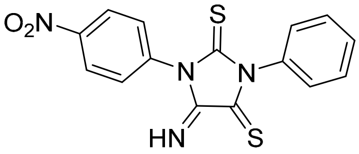

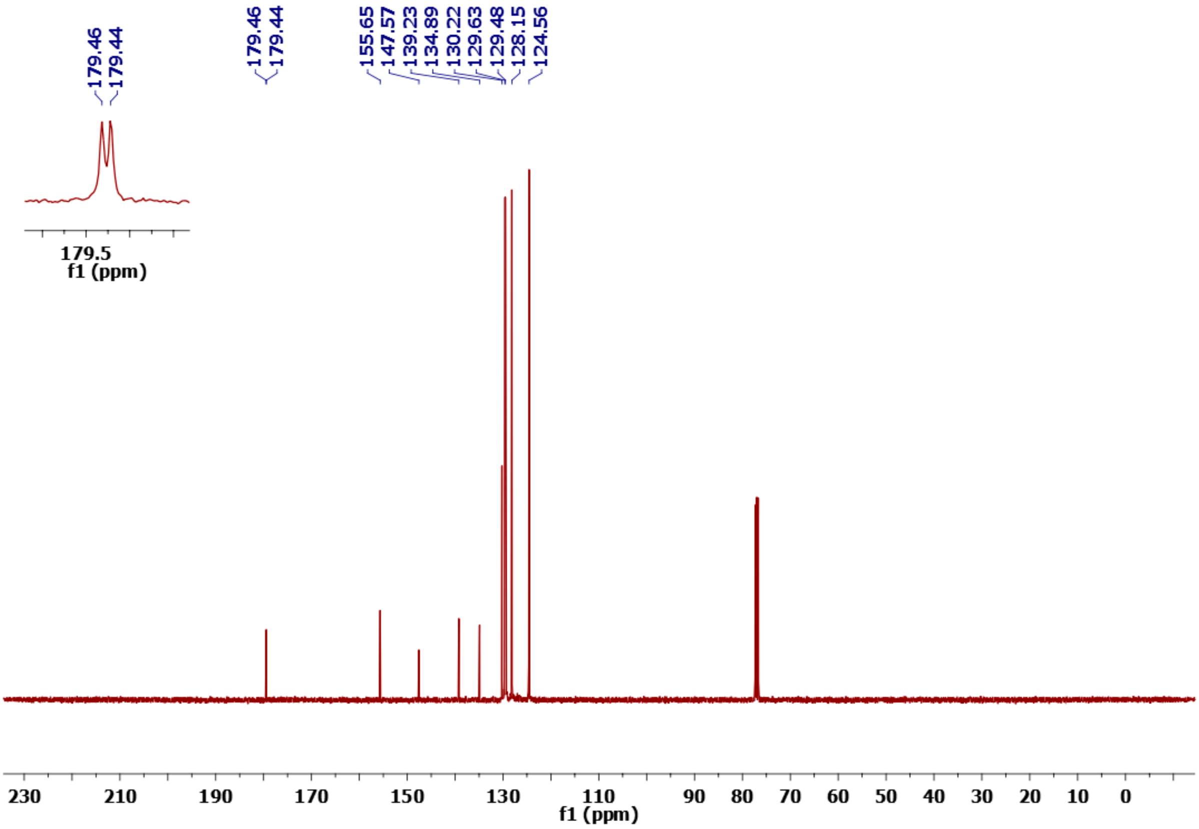

^{13}C-CRAPT NMR ($CDCl_3$) spectrum of 5-imino-1-(4-nitrophenyl)-3-phenylimidazolidine-2,4-dithione

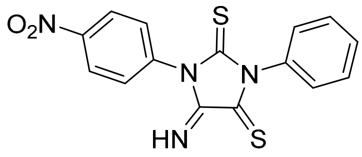

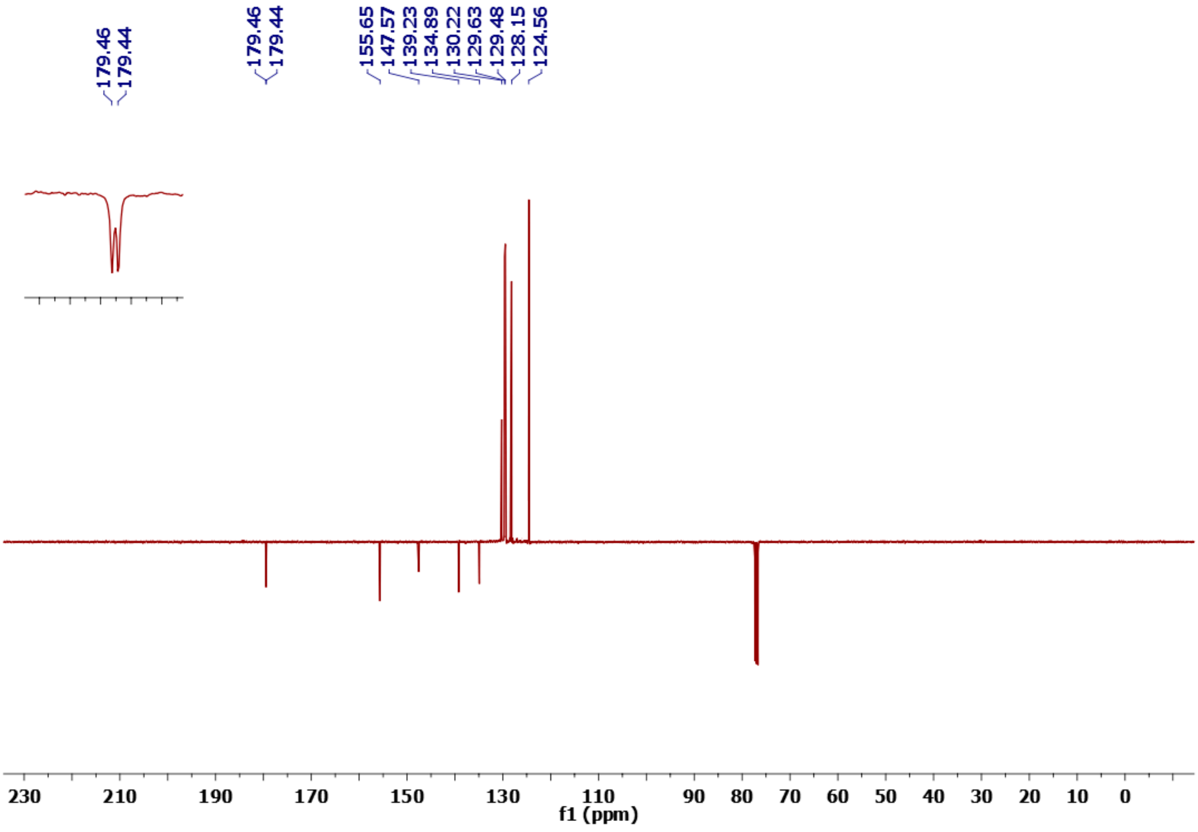

^{1}H-^{1}H-gCOSYAD NMR ($CDCl_3$) spectrum of 5-imino-1-(4-nitrophenyl)-3-phenylimidazolidine-2,4-dithione

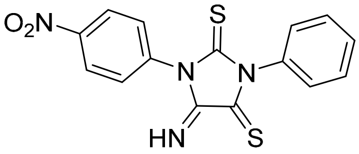

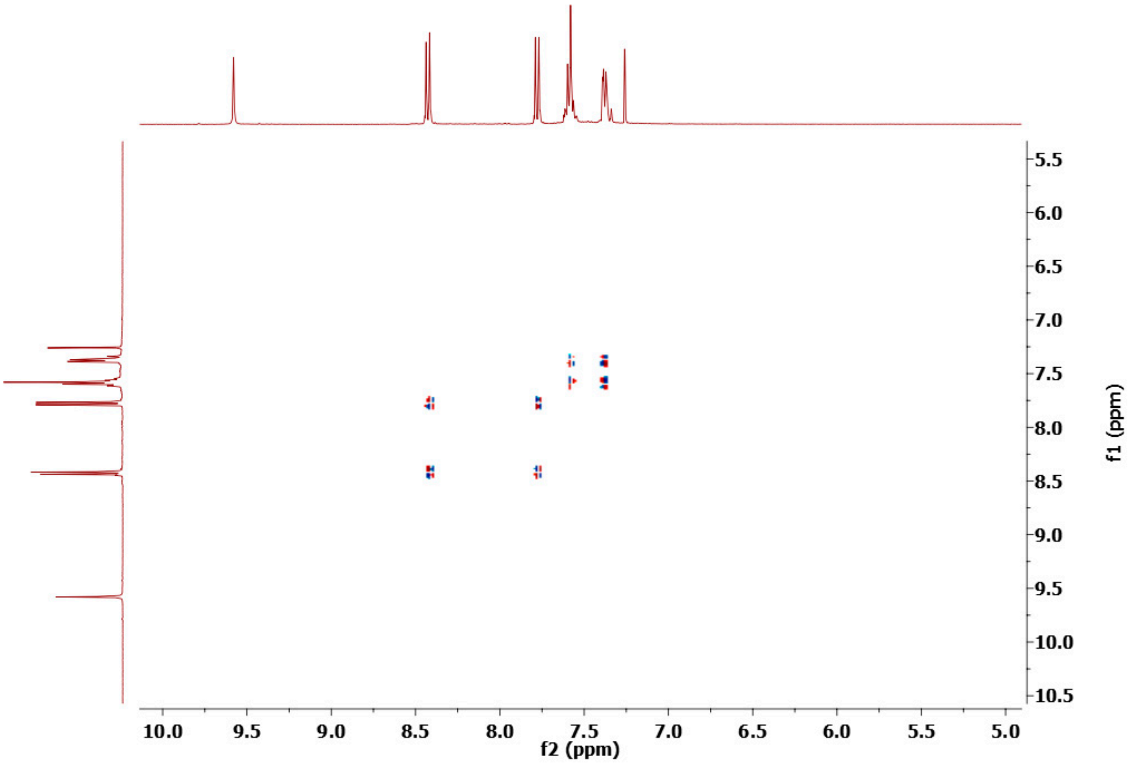

^{1}H-^{13}C-gHSQCAD NMR ($CDCl_3$) spectrum of 5-imino-1-(4-nitrophenyl)-3-phenylimidazolidine-2,4-dithione

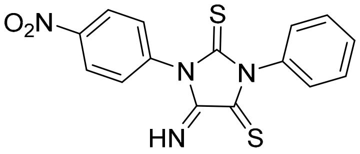

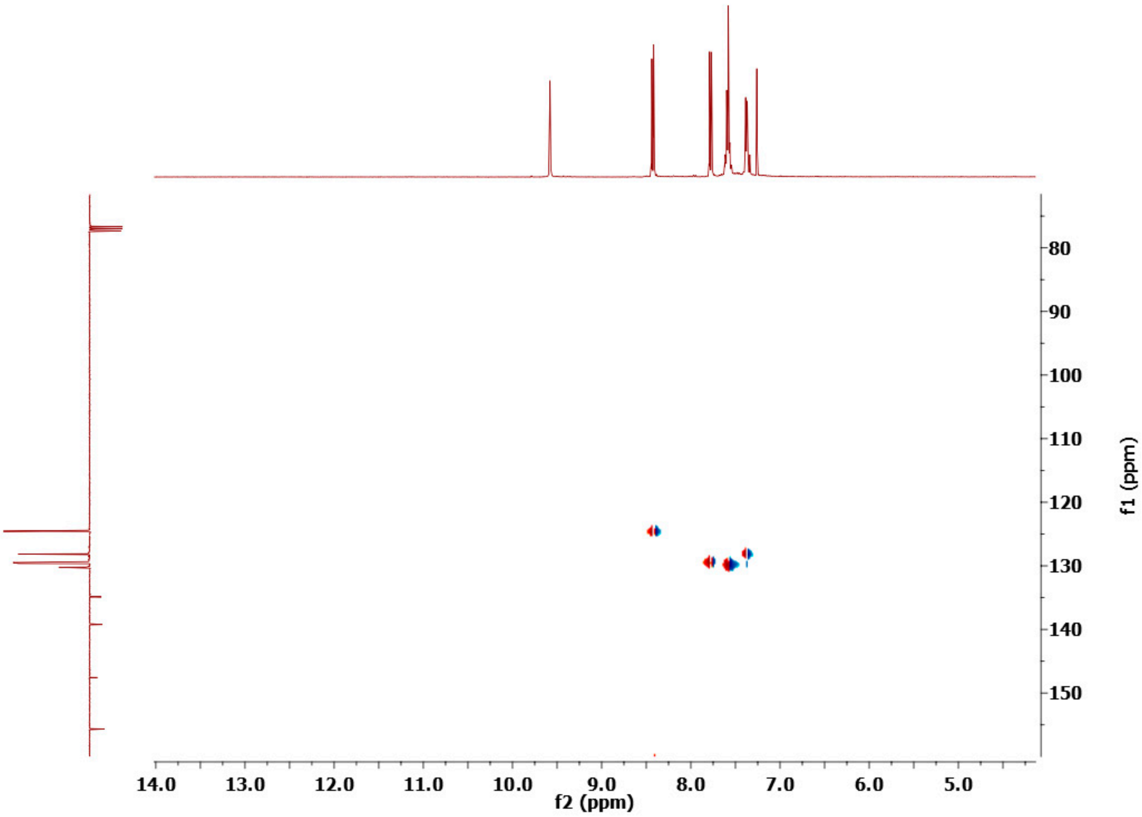

^{1}H-^{13}C-HMBC NMR ($CDCl_3$) spectrum of 5-imino-1-(4-nitrophenyl)-3-phenylimidazolidine-2,4-dithione

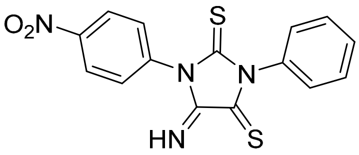

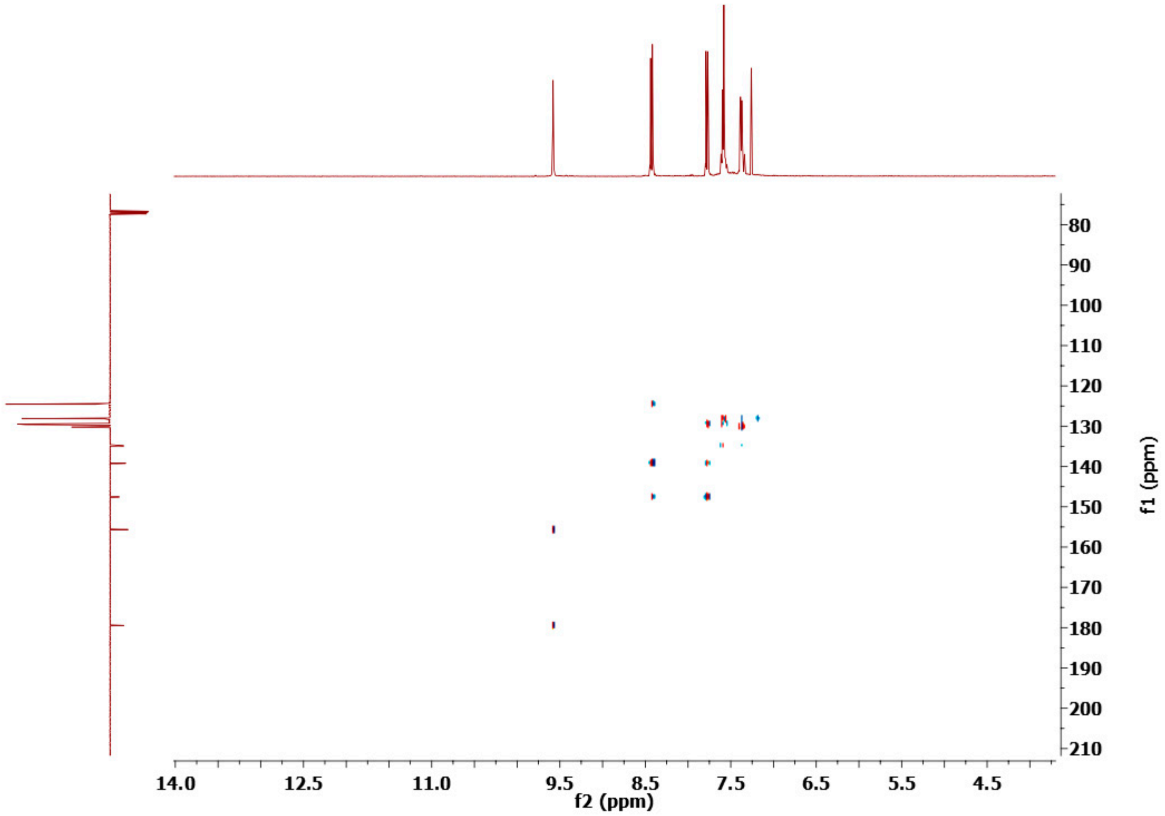

^{1}H NMR ($CDCl_3$) spectrum of 5-imino-1-(4-methoxyphenyl)-3-phenylimidazolidine-2,4-dithione (18i)

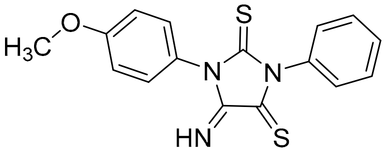

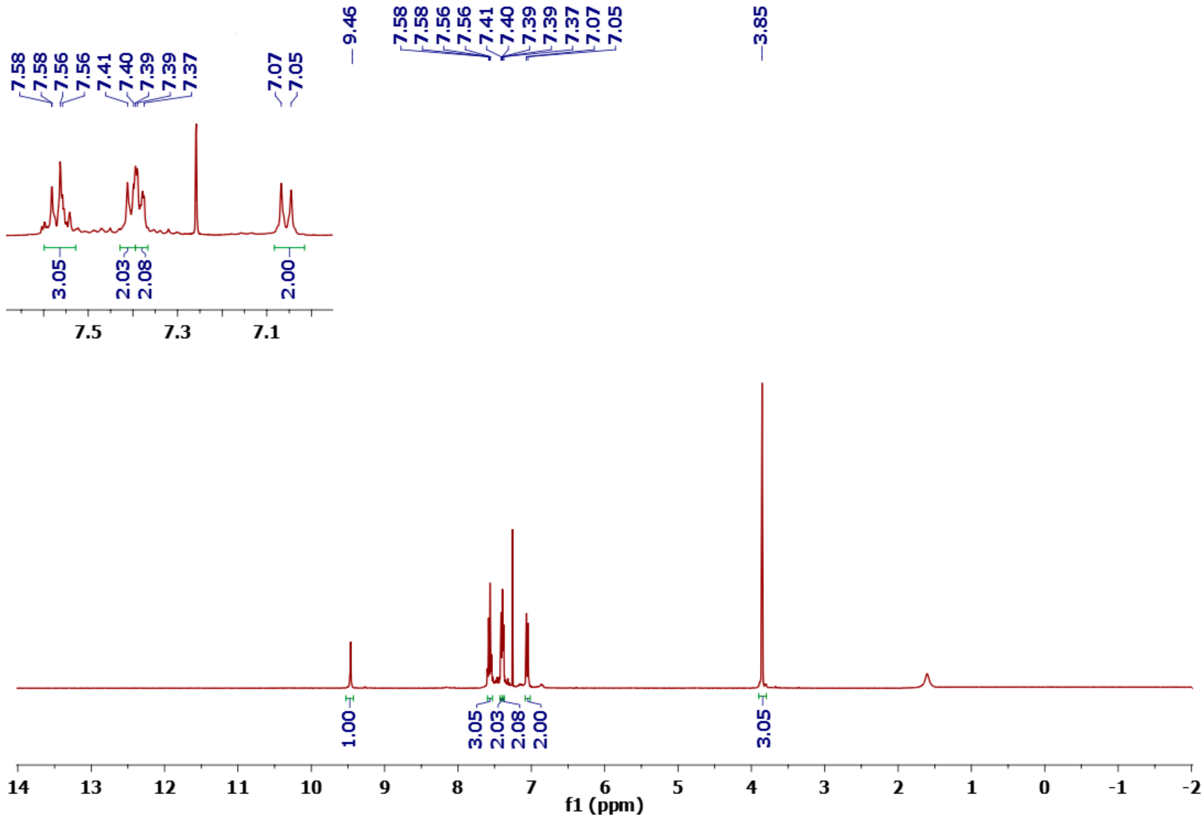

^{13}C NMR ($CDCl_3$) spectrum of 5-imino-1-(4-methoxyphenyl)-3-phenylimidazolidine-2,4-dithione

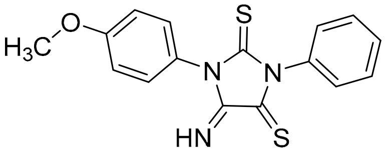

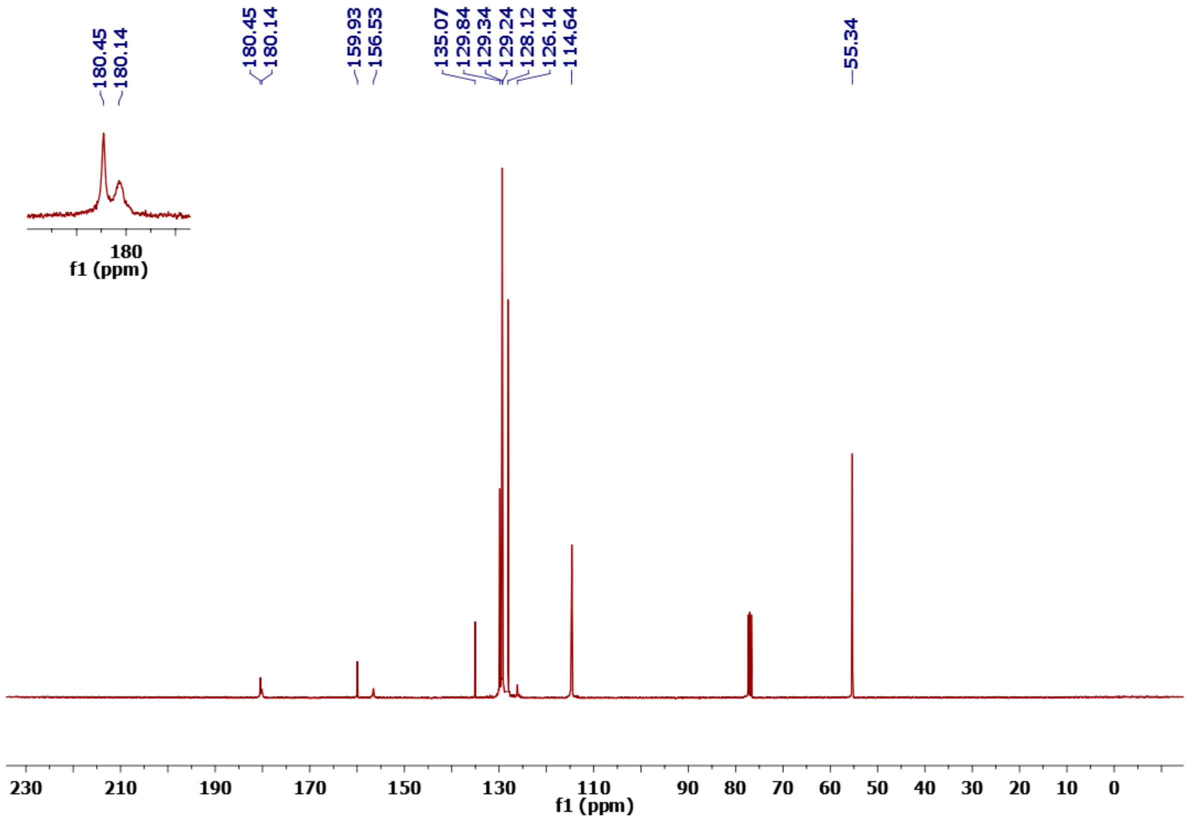

^{13}C-CRAPT NMR ($CDCl_3$) spectrum of 5-imino-1-(4-methoxyphenyl)-3-phenylimidazolidine-2,4-dithione

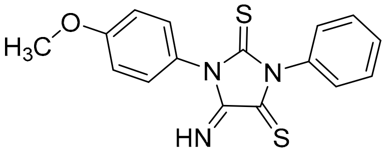

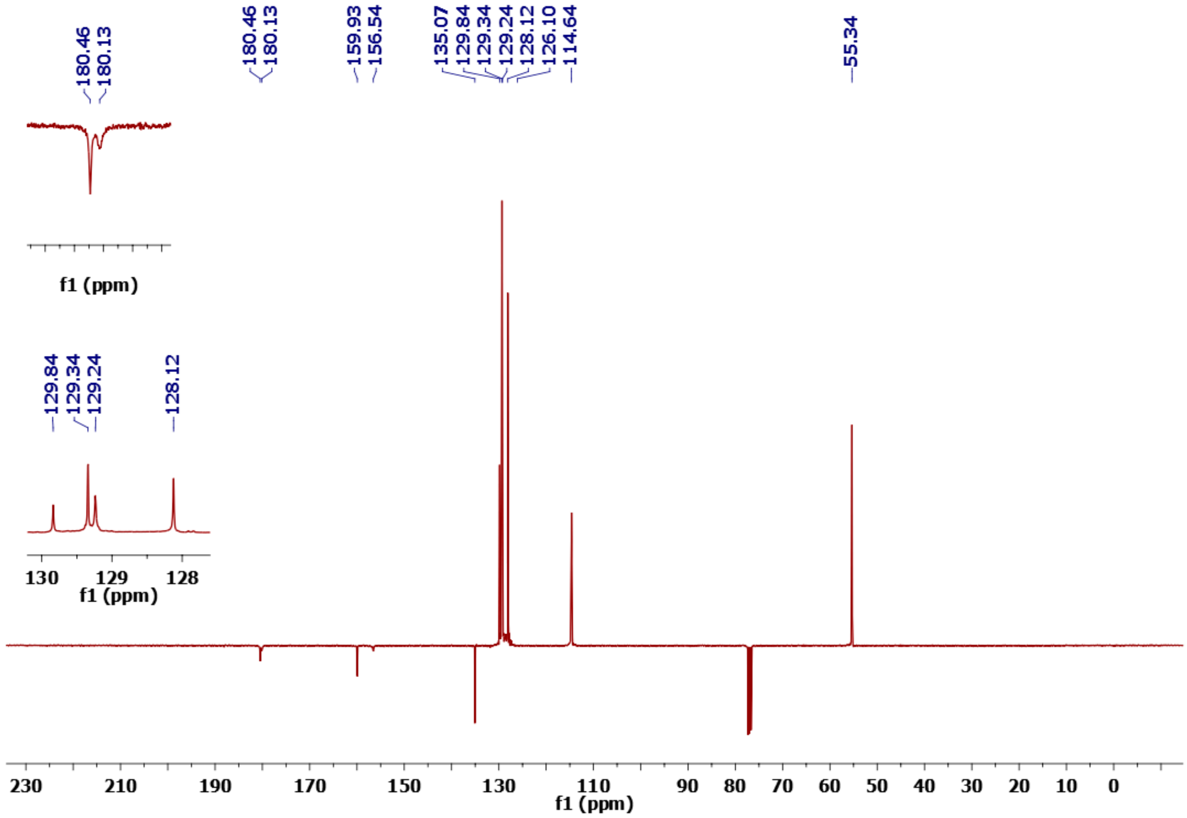

^{1}H-^{1}H-gCOSYAD NMR ($CDCl_3$) spectrum of 5-imino-1-(4-methoxyphenyl)-3-phenylimidazolidine-2,4-dithione

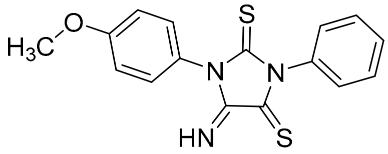

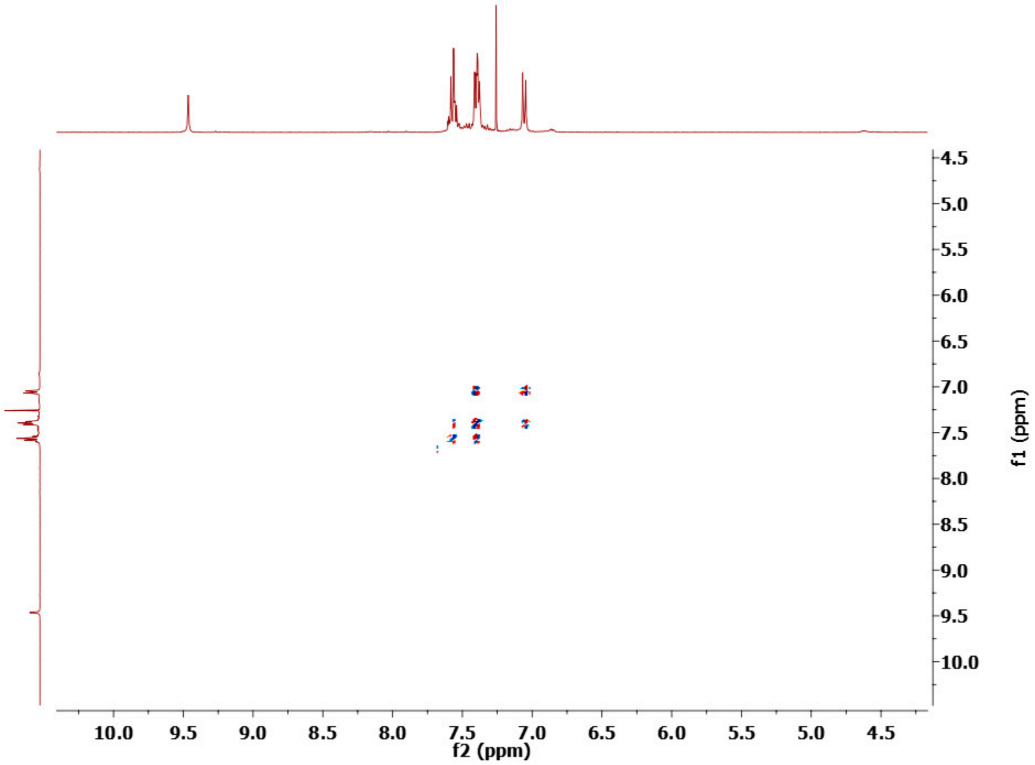

^{1}H-^{13}C-gHSQCAD NMR ($CDCl_3$) spectrum of 5-imino-1-(4-methoxyphenyl)-3-phenylimidazolidine-2,4-dithione

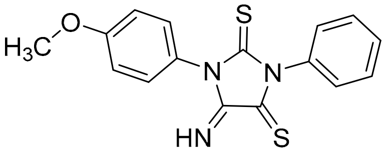

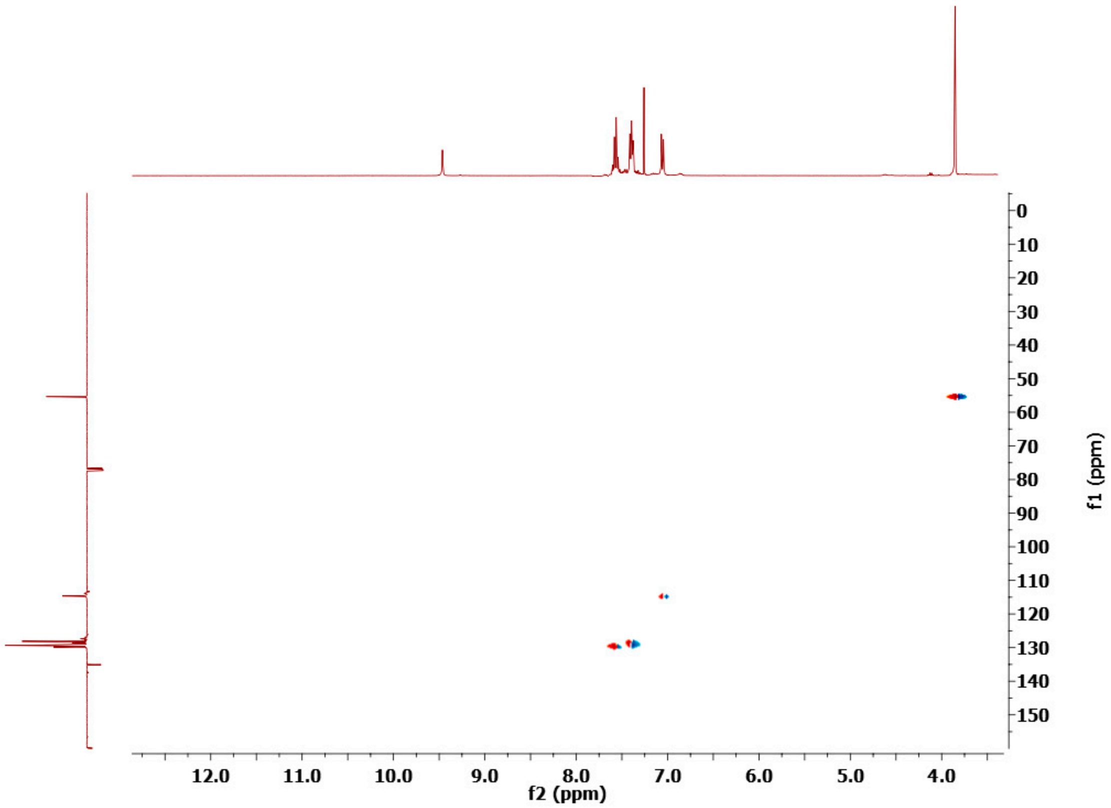

^{1}H-^{13}C-gHMBC NMR ($CDCl_3$) spectrum of 5-imino-1-(4-methoxyphenyl)-3-phenylimidazolidine-2,4-dithione

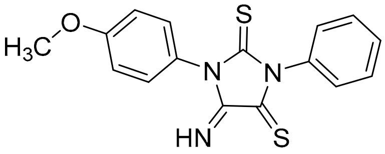

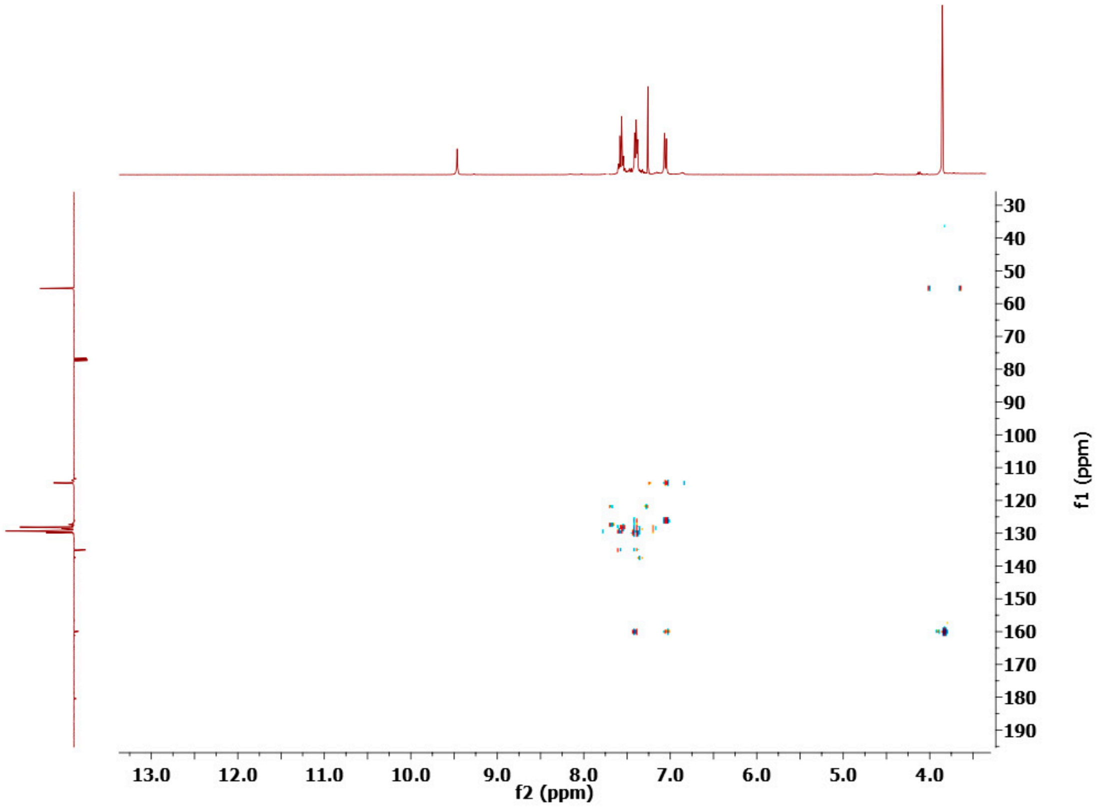

^{1}H NMR ($CDCl_3$) spectrum of (3-(4-chlorophenyl)-5-imino-2,4-dithioxoimidazolidin-1-yl)(phenyl)methanone (18j)

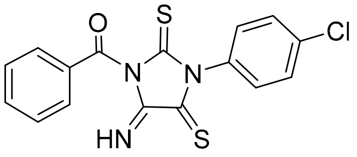

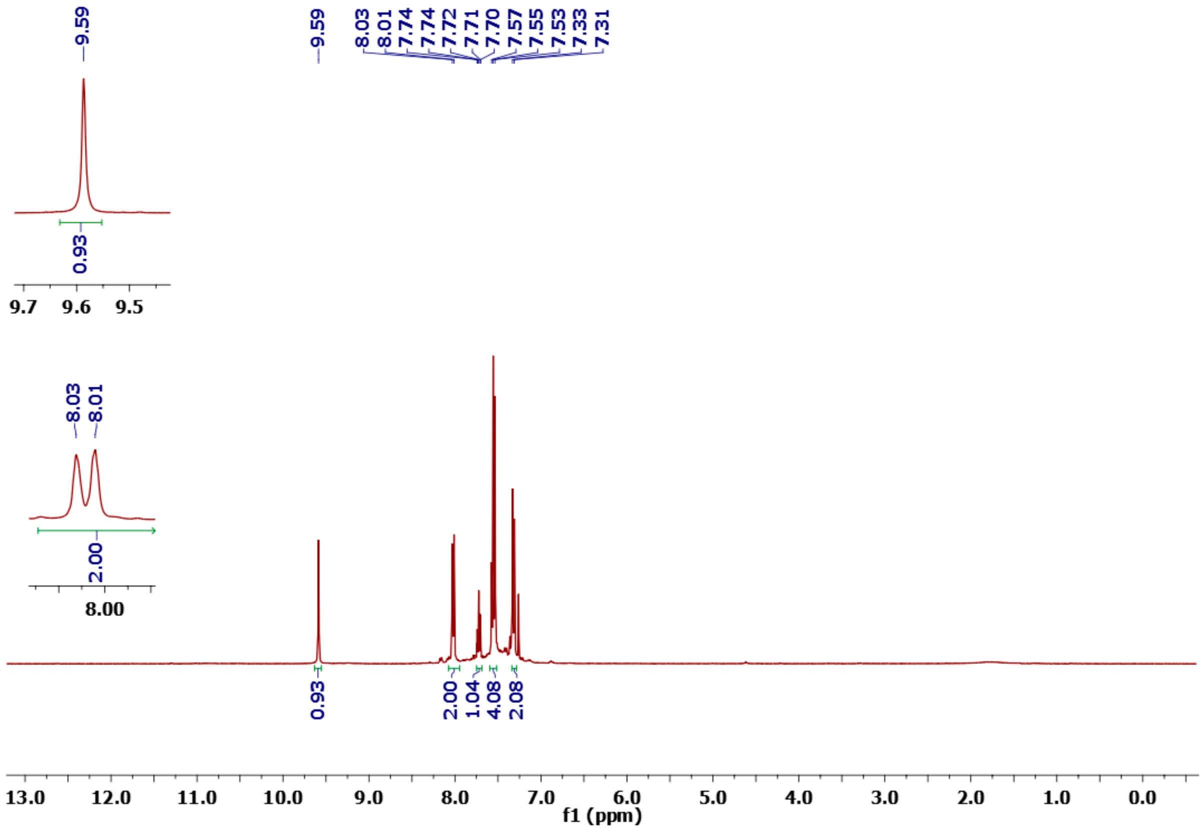

^{13}C NMR ($CDCl_3$) spectrum of (3-(4-chlorophenyl)-5-imino-2,4-dithioxoimidazolidin-1-yl)(phenyl)methanone

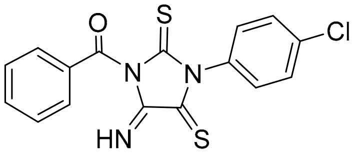

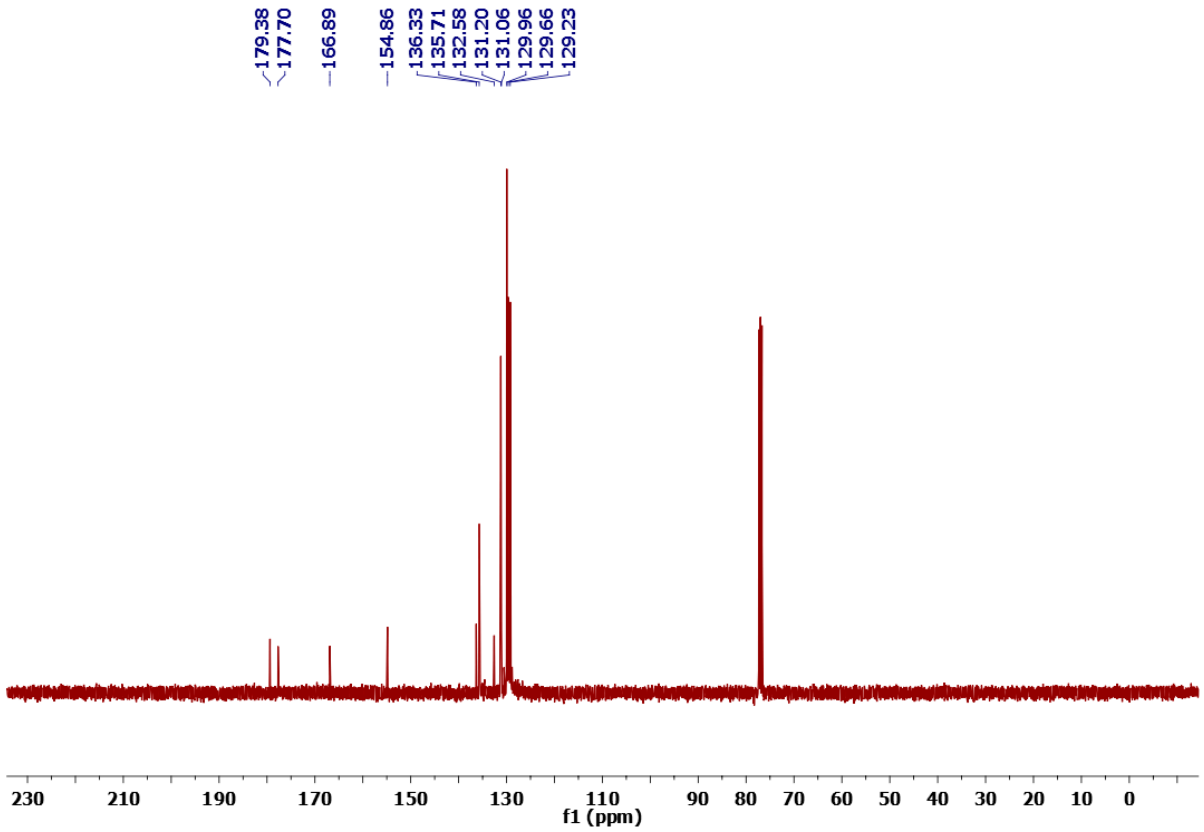

^{13}C-CRAPT NMR ($CDCl_3$) spectrum of (3-(4-chlorophenyl)-5-imino-2,4-dithioxoimidazolidin-1-yl)(phenyl)methanone

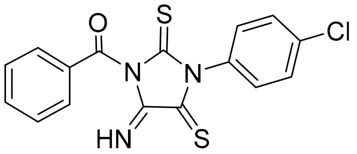

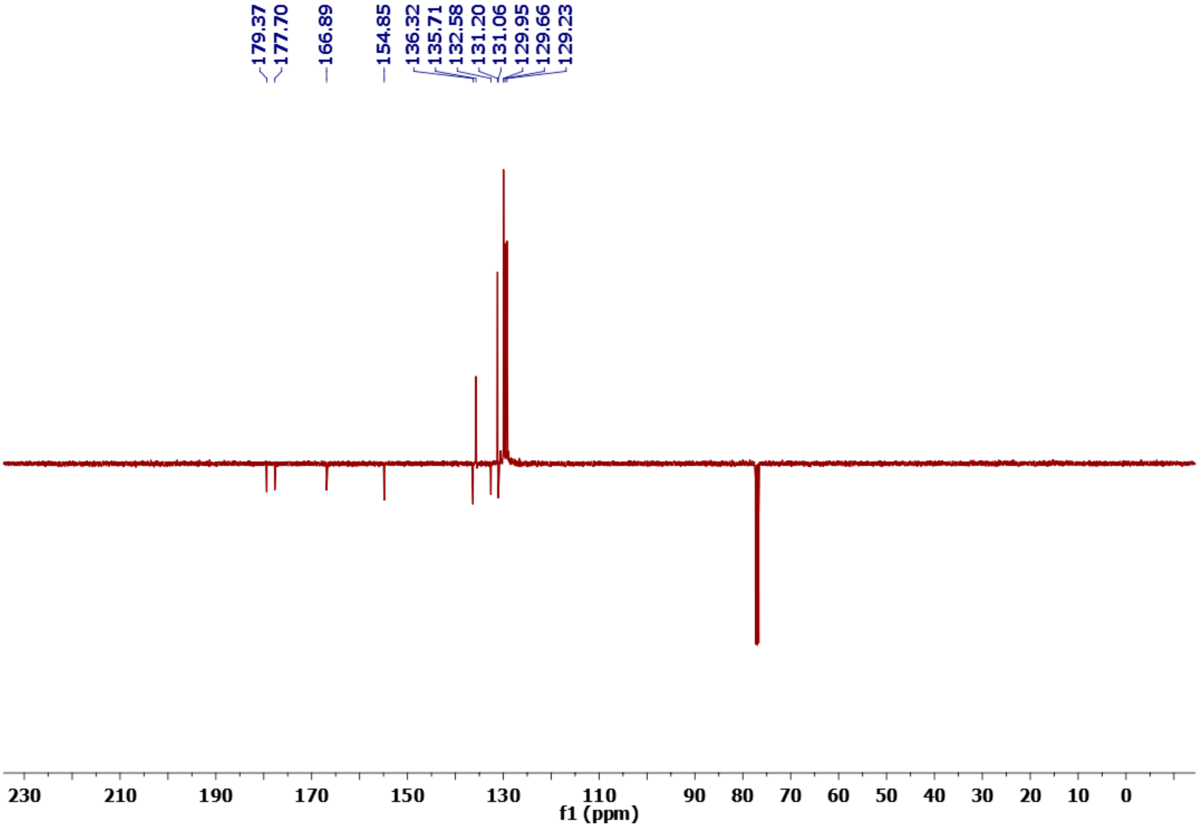

^{1}H-^{1}H-gCOSYAD NMR ($CDCl_3$) spectrum of (3-(4-chlorophenyl)-5-imino-2,4-dithioxoimidazolidin-1-yl)(phenyl)methanone

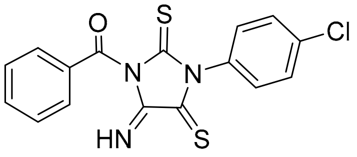

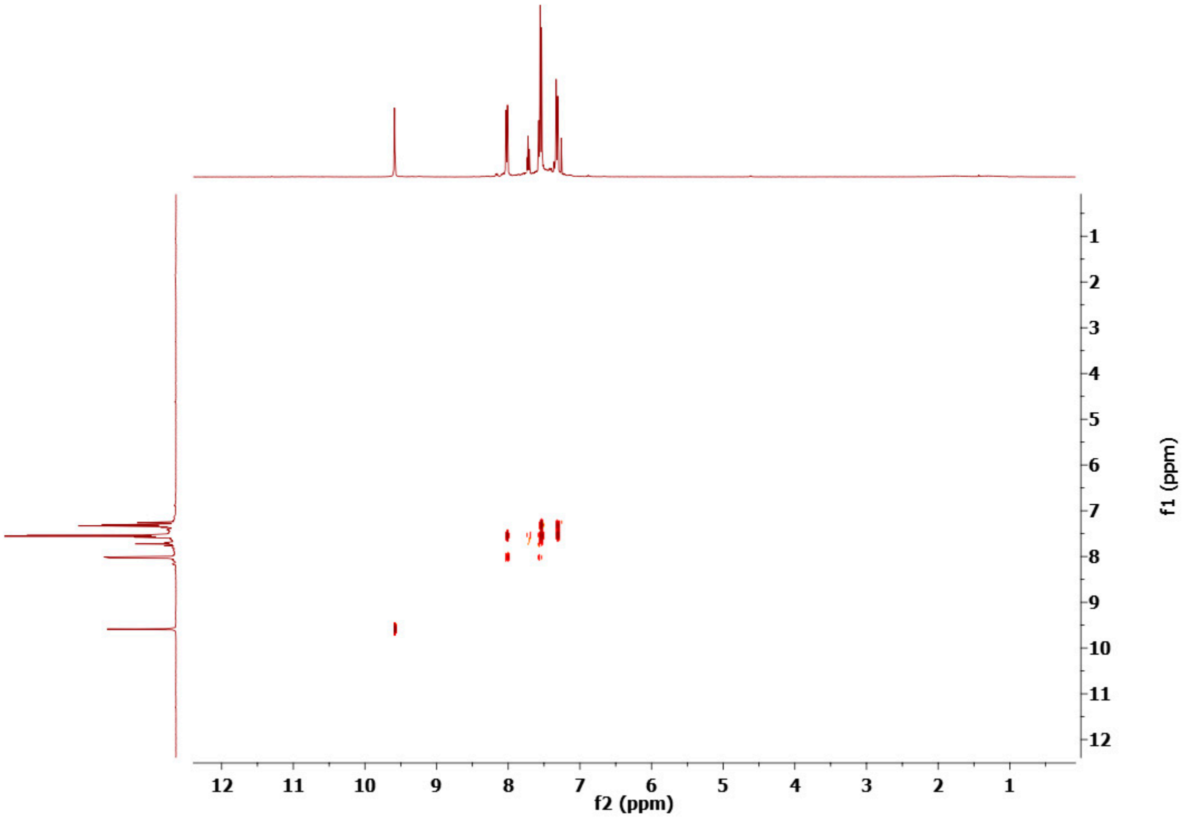

^{1}H-^{13}C-gHSQCAD NMR ($CDCl_3$) spectrum of (3-(4-chlorophenyl)-5-imino-2,4-dithioxoimidazolidin-1-yl)(phenyl)methanone

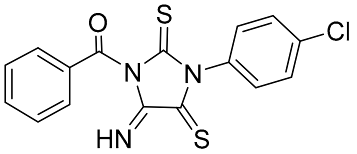

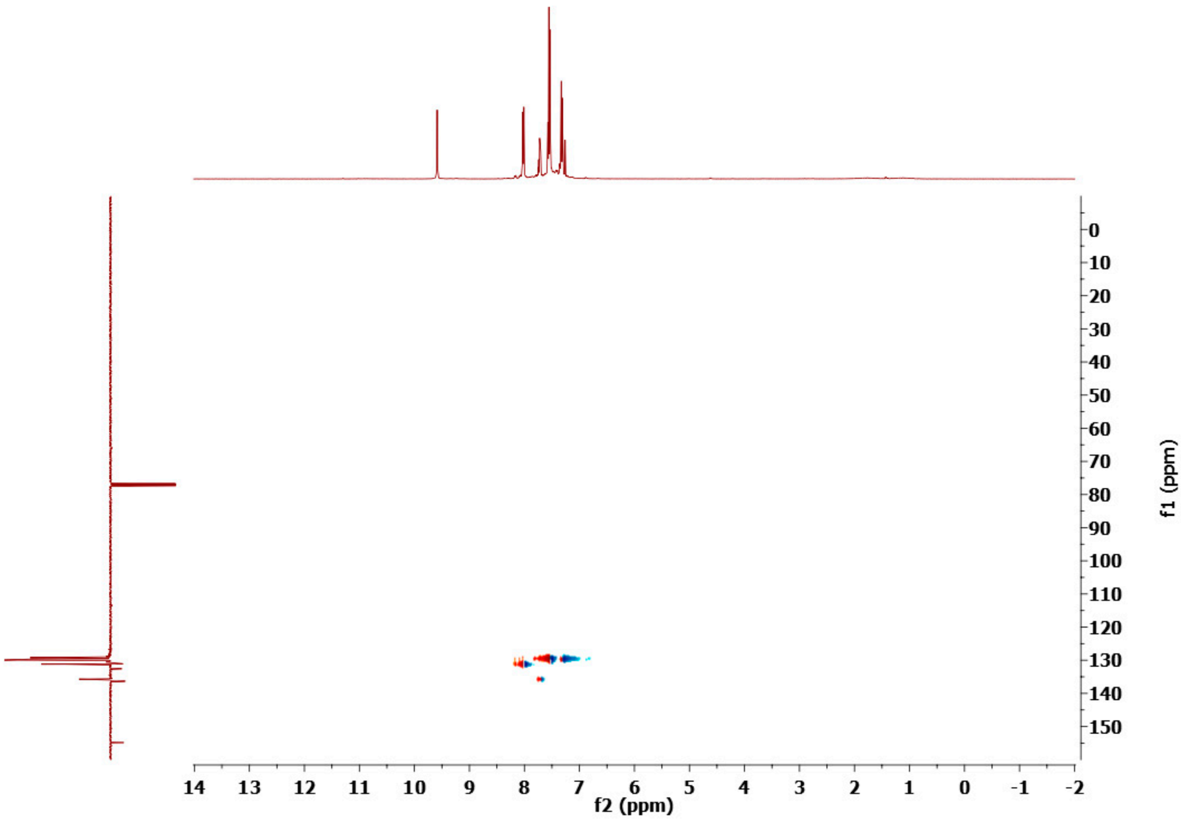

^{1}H-^{13}C-gHMBC NMR ($CDCl_3$) spectrum of (3-(4-chlorophenyl)-5-imino-2,4-dithioxoimidazolidin-1-yl)(phenyl)methanone

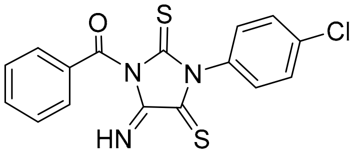

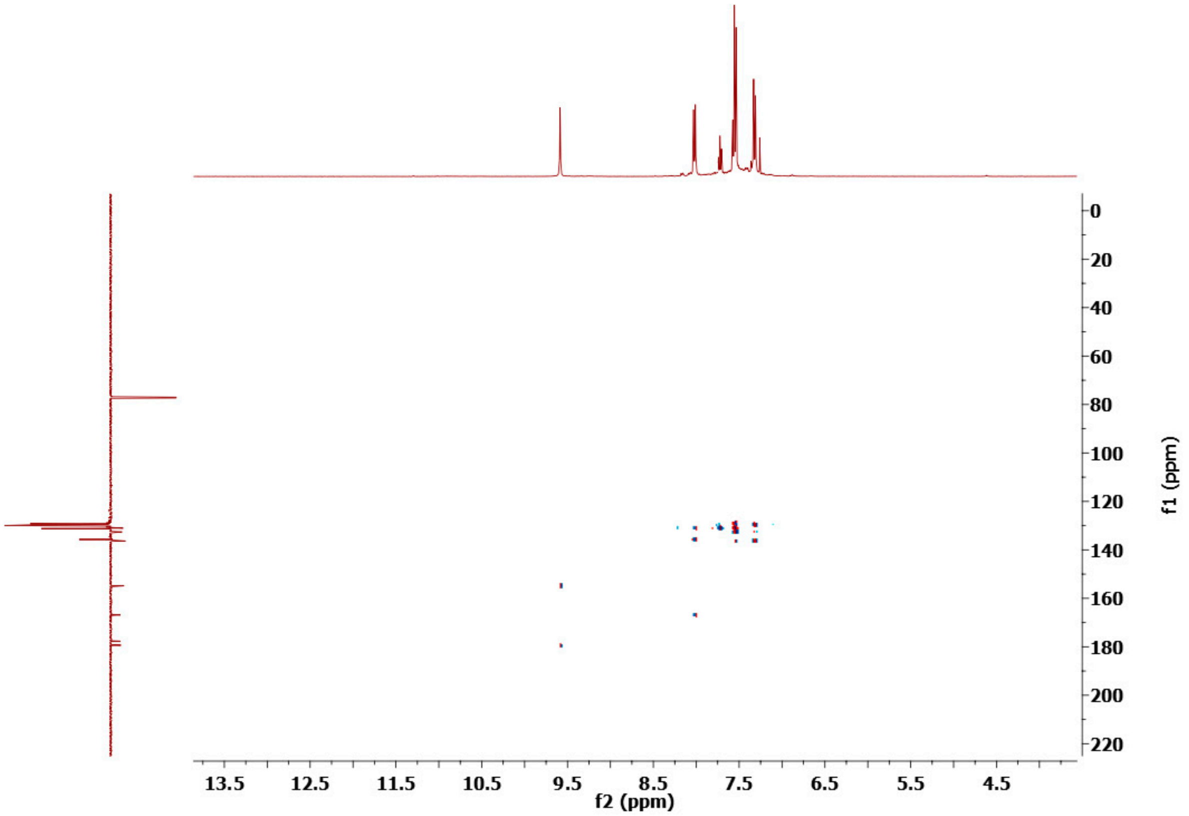

^{1}H NMR ($CDCl_3$) spectrum of 3-(4-chlorophenyl)-5-imino-1-phenylimidazolidine-2,4-dithione (18k)

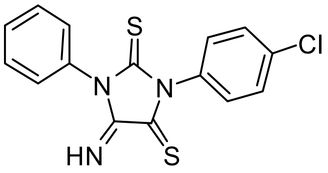

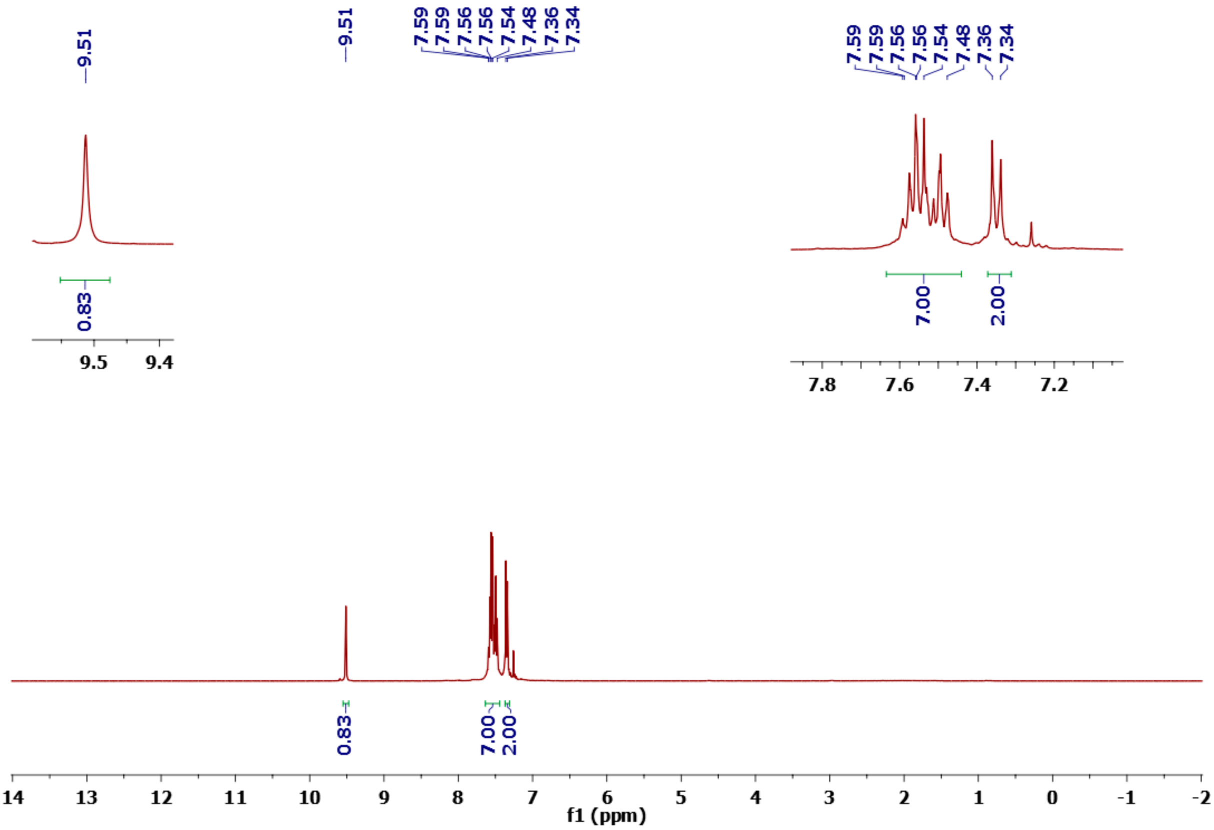

^{13}C NMR ($CDCl_3$) spectrum of 3-(4-chlorophenyl)-5-imino-1-phenylimidazolidine-2,4-dithione

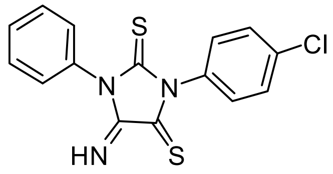

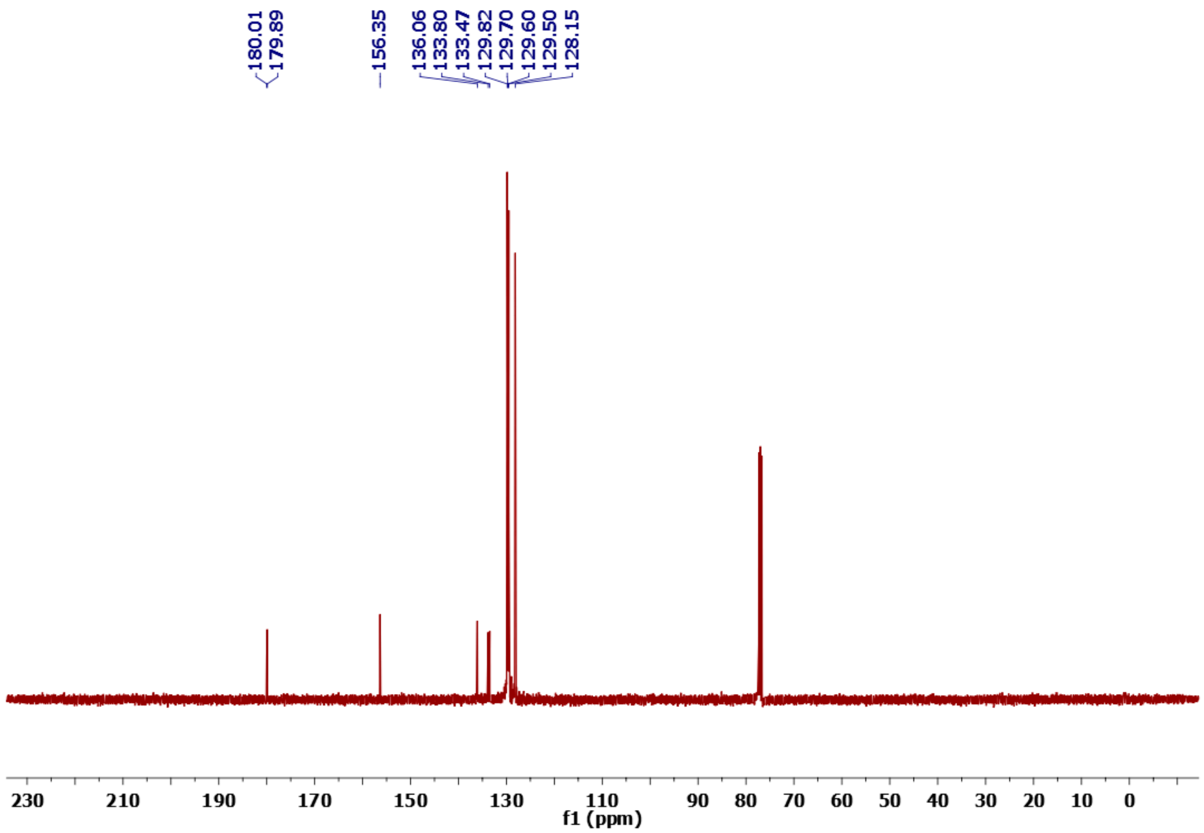

^{13}C-CRAPT NMR ($CDCl_3$) spectrum of 3-(4-chlorophenyl)-5-imino-1-phenylimidazolidine-2,4-dithione

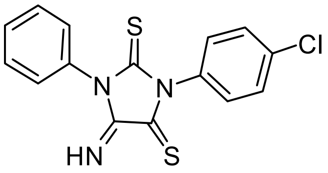

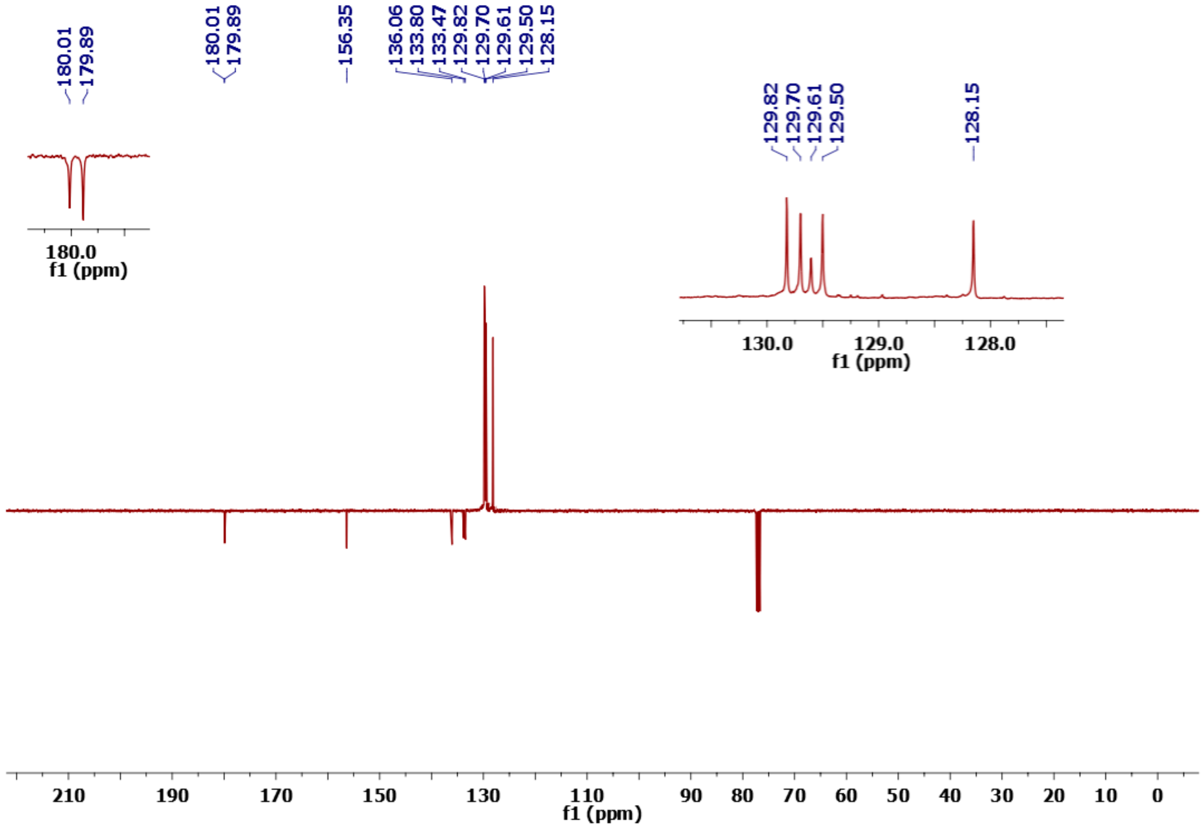

^{1}H-^{1}H-gCOSYAD NMR ($CDCl_3$) spectrum of 3-(4-chlorophenyl)-5-imino-1-phenylimidazolidine-2,4-dithione

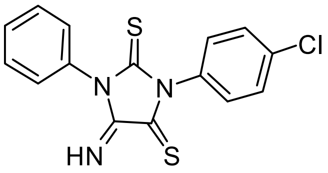

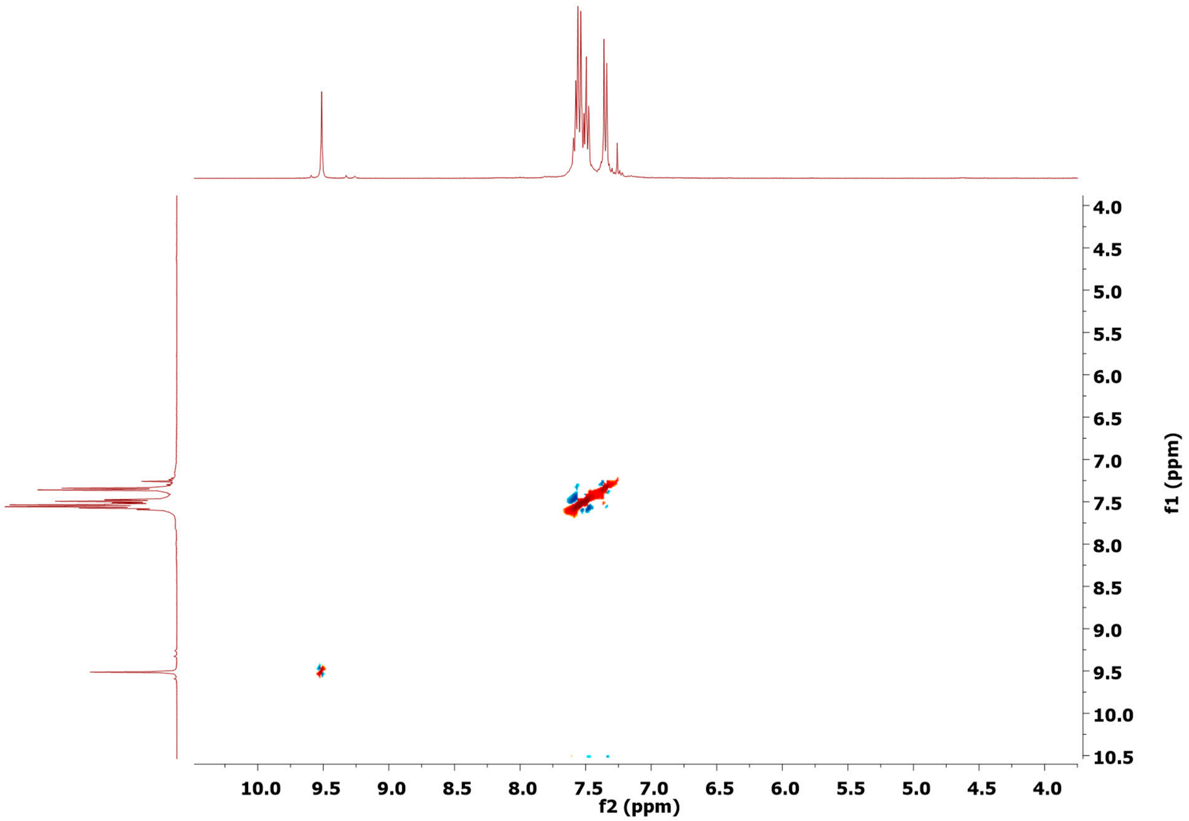

^{1}H-^{13}C-gHSQCAD NMR ($CDCl_3$) spectrum of 3-(4-chlorophenyl)-5-imino-1-phenylimidazolidine-2,4-dithione

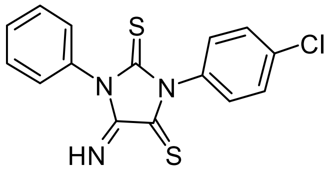

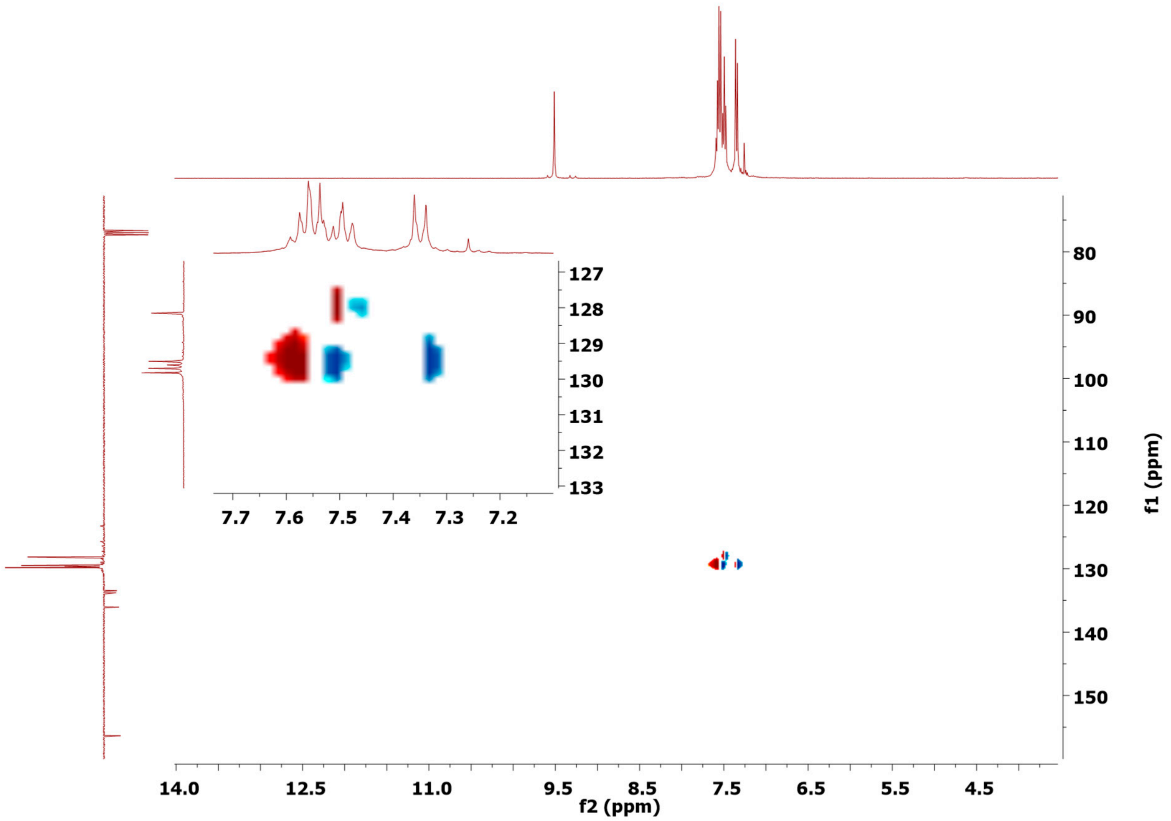

^{1}H-^{13}C-gHMBC NMR ($CDCl_3$) spectrum of 3-(4-chlorophenyl)-5-imino-1-phenylimidazolidine-2,4-dithione

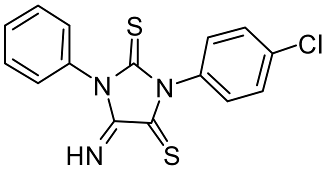

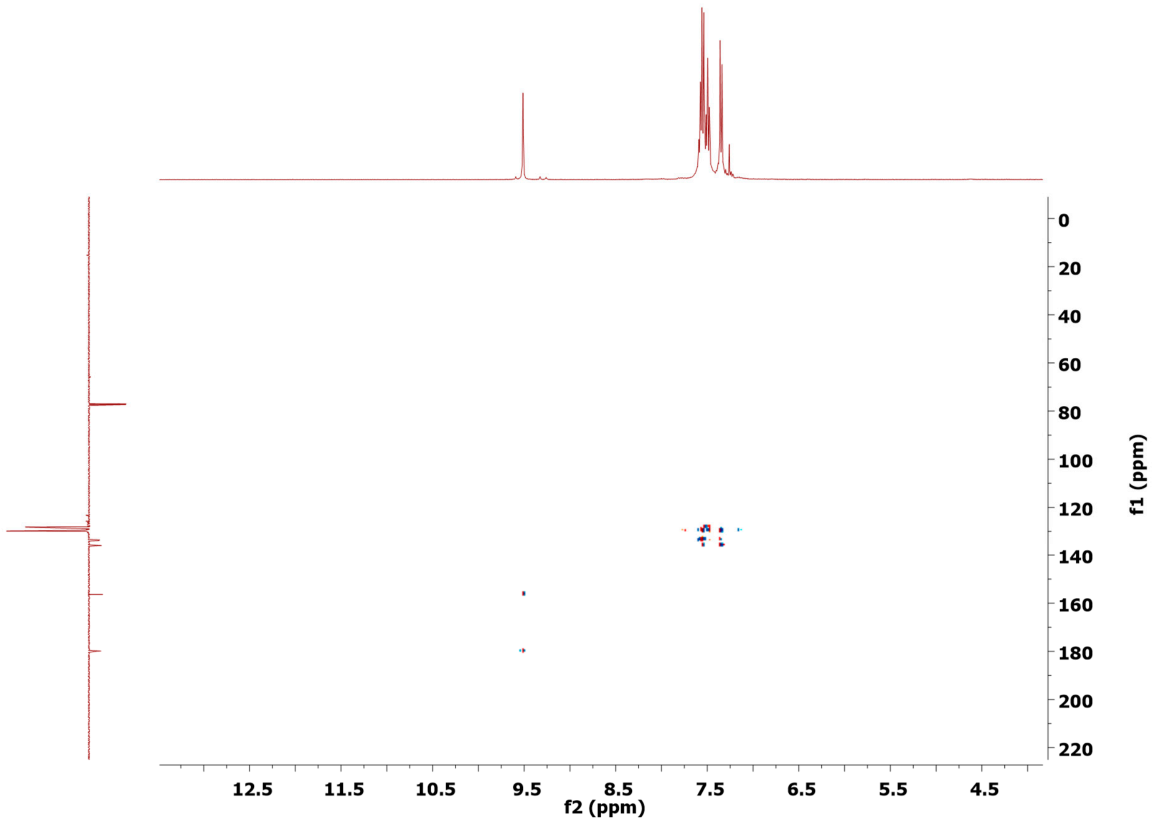

^{1}H NMR ($CDCl_3$) spectrum of 3-(4-chlorophenyl)-5-imino-1-(4-(trifluoromethyl)phenyl)imidazolidine-2,4-dithione (18l)

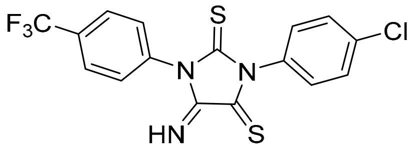

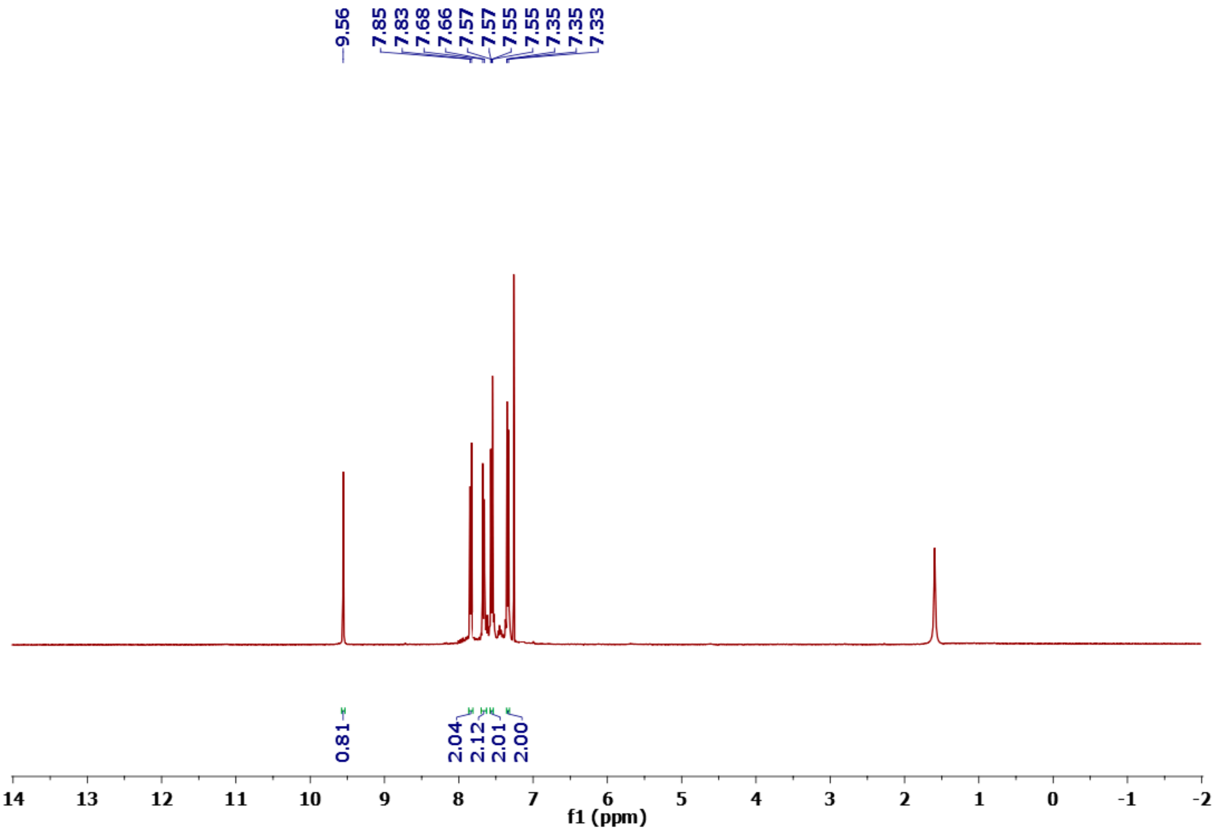

^{13}C NMR ($CDCl_3$) spectrum of 3-(4-chlorophenyl)-5-imino-1-(4-(trifluoromethyl)phenyl)imidazolidine-2,4-dithione

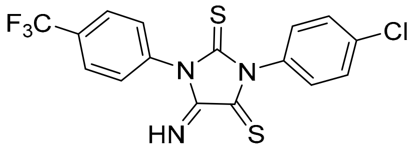

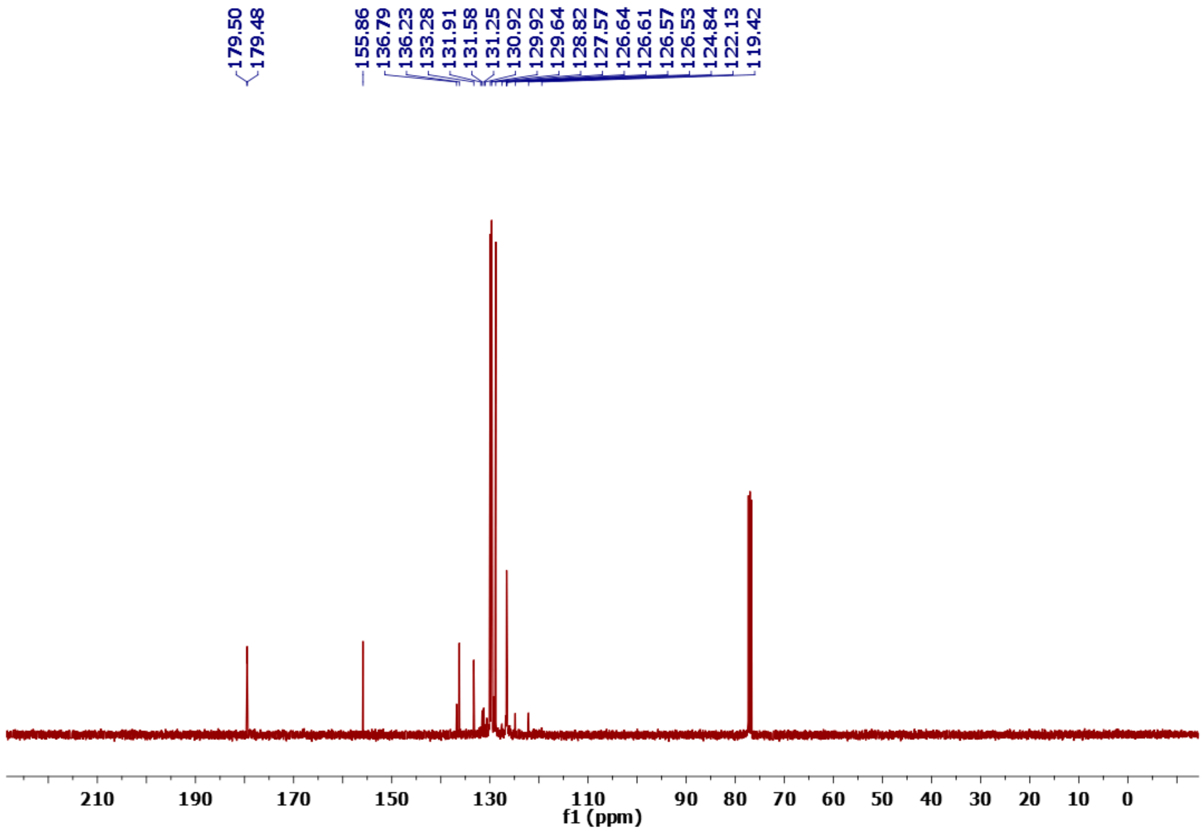

^{13}C-CRAPT NMR ($CDCl_3$) spectrum of 3-(4-chlorophenyl)-5-imino-1-(4-(trifluoromethyl)phenyl)imidazolidine-2,4-dithione

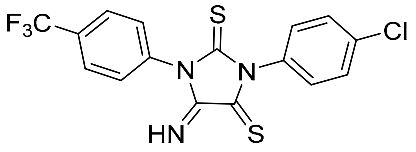

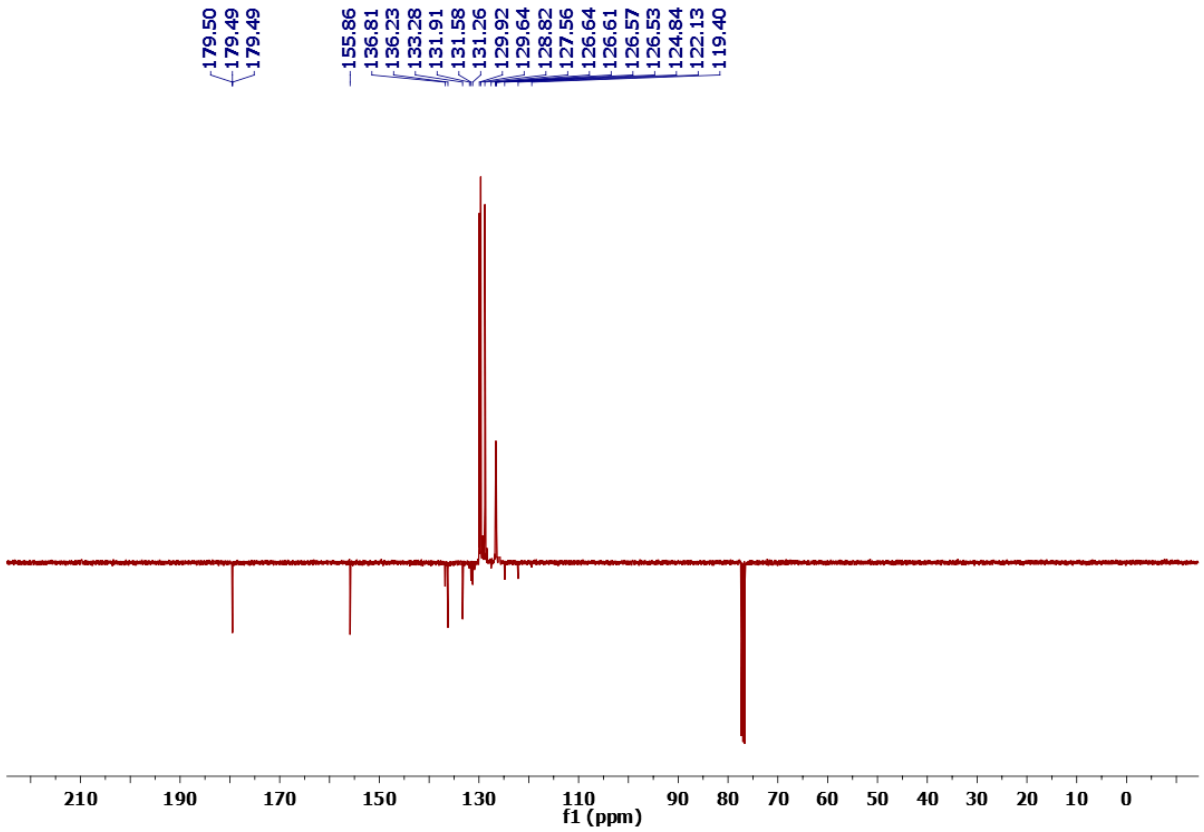

^{1}H-^{1}H-gCOSYAD NMR ($CDCl_3$) spectrum of 3-(4-chlorophenyl)-5-imino-1-(4-(trifluoromethyl)phenyl)imidazolidine-2,4-dithione

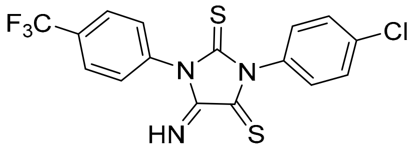

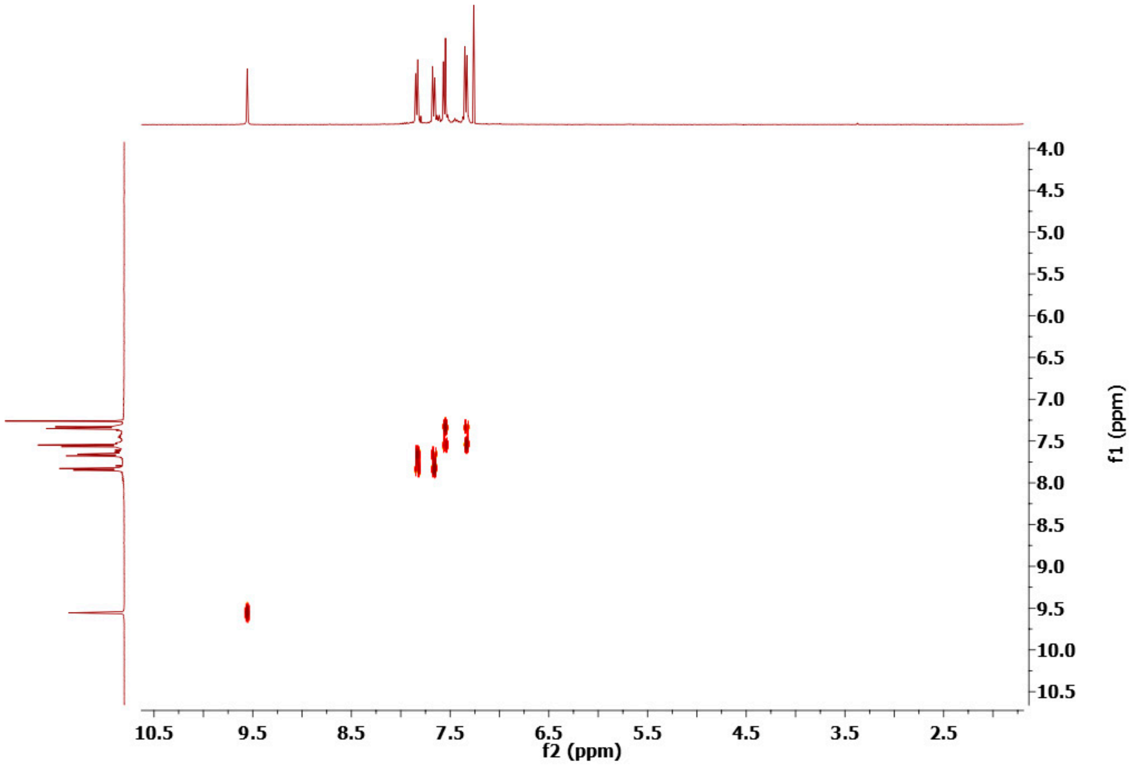

^{1}H-^{13}C-gHSQCAD NMR ($CDCl_3$) spectrum of 3-(4-chlorophenyl)-5-imino-1-(4-(trifluoromethyl)phenyl)imidazolidine-2,4-dithione

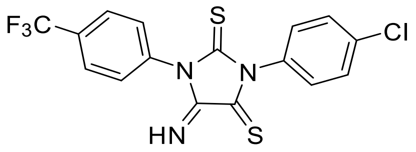

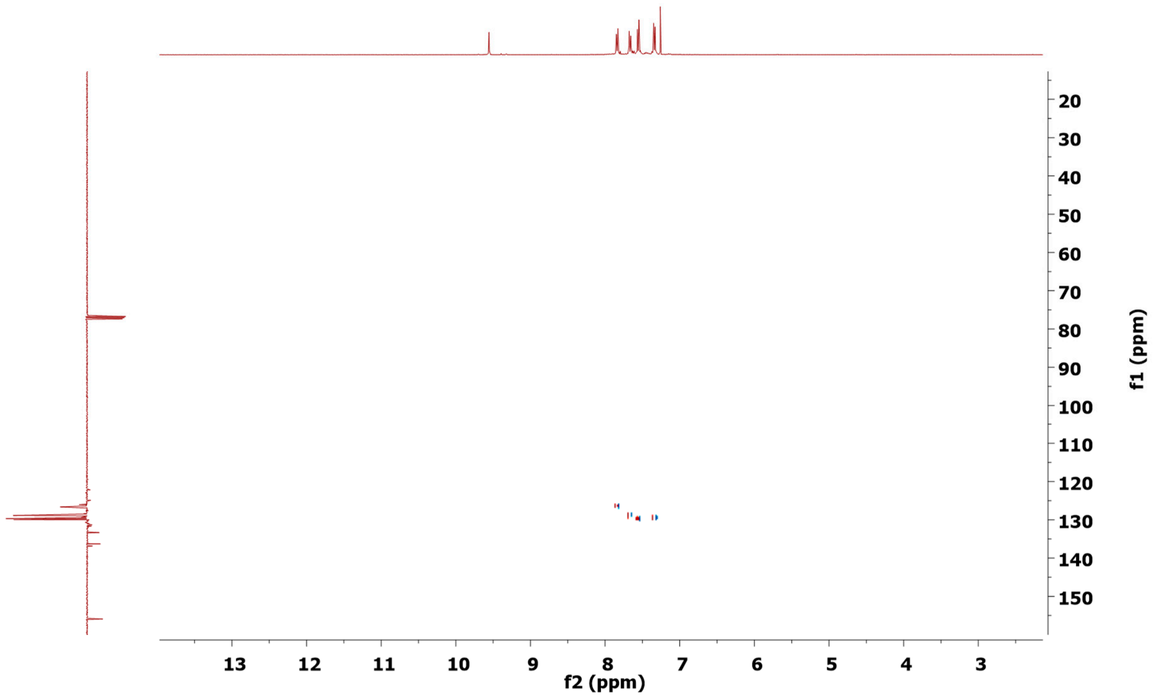

^{1}H-^{13}C-gHMBC NMR ($CDCl_3$) spectrum of 3-(4-chlorophenyl)-5-imino-1-(4-(trifluoromethyl)phenyl)imidazolidine-2,4-dithione

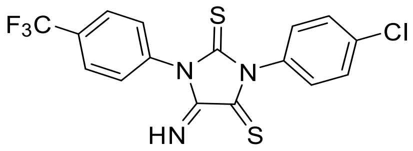

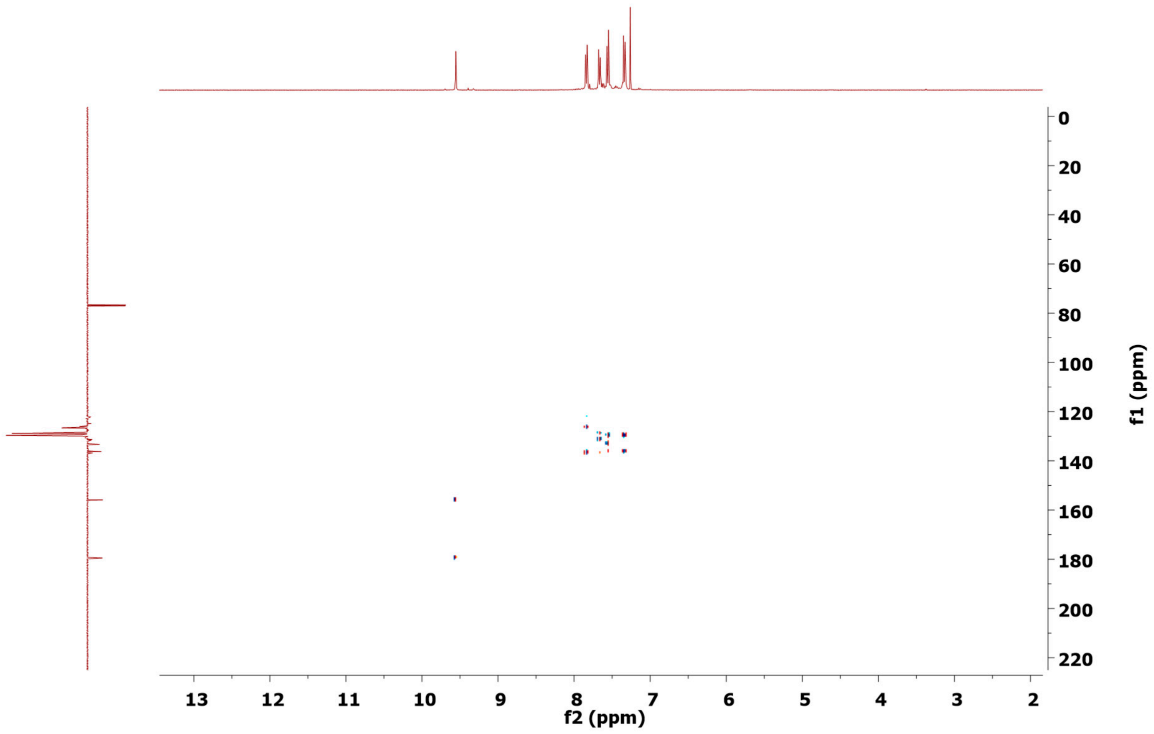

^{1}H NMR ($CDCl_3$) spectrum of 3-(4-chlorophenyl)-5-imino-1-(p-tolyl)imidazolidine-2,4-dithione (18m)

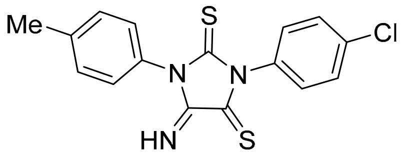

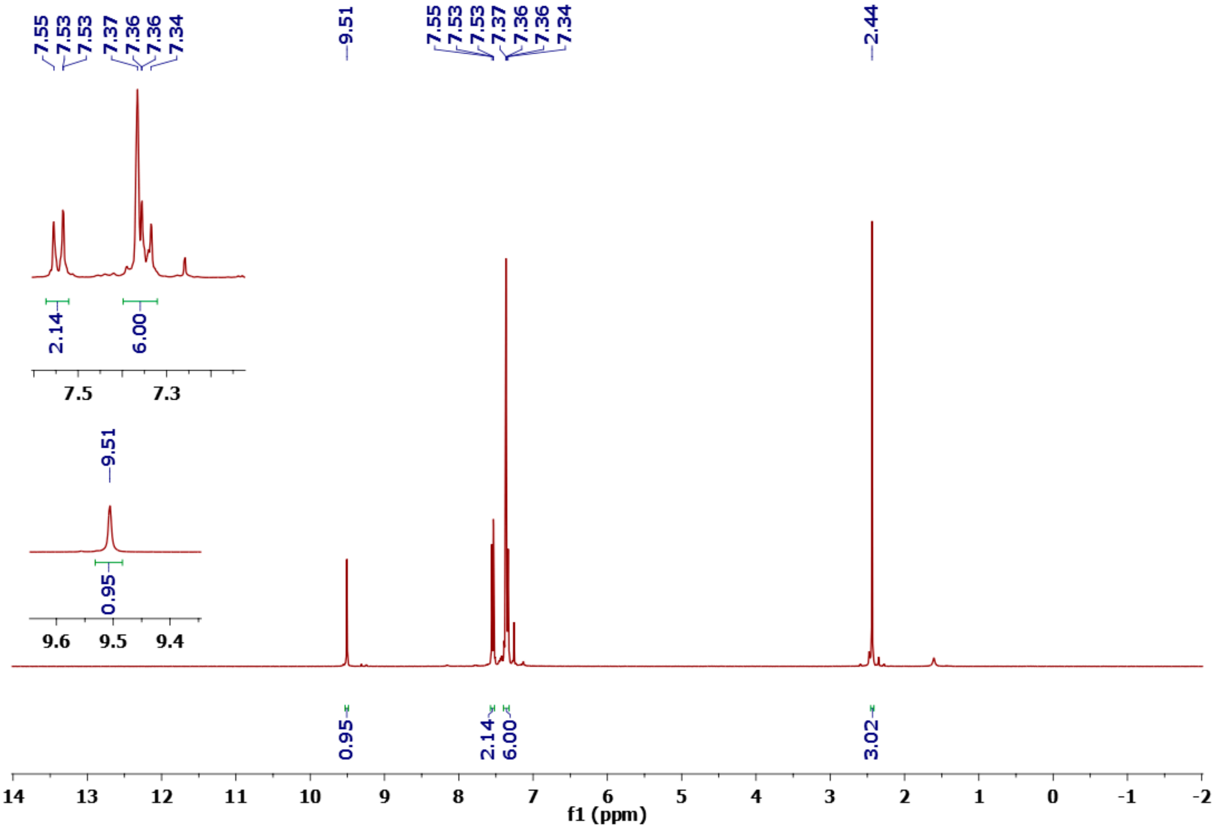

^{13}C NMR ($CDCl_3$) spectrum of 3-(4-chlorophenyl)-5-imino-1-(p-tolyl)imidazolidine-2,4-dithione

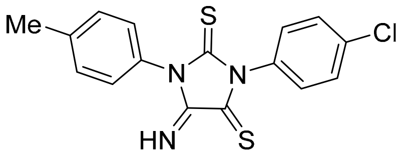

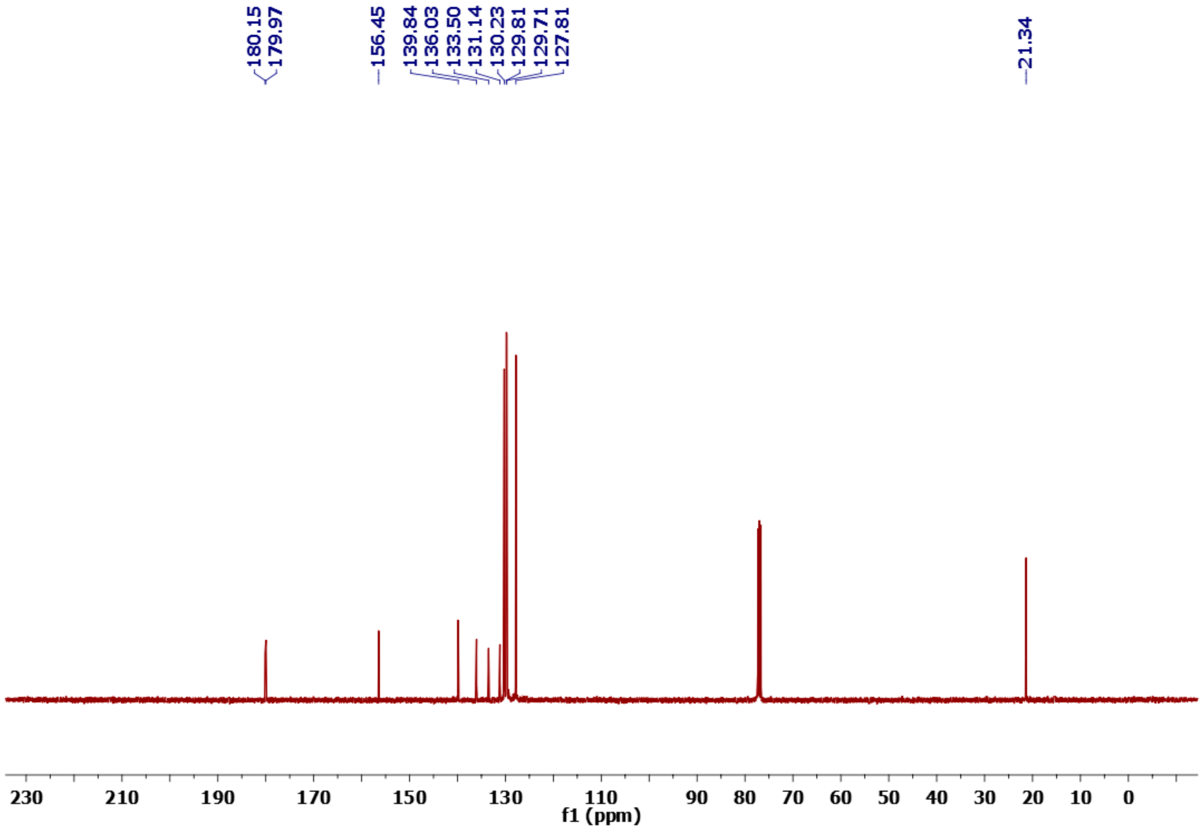

^{13}C-CRAPT NMR ($CDCl_3$) spectrum of 3-(4-chlorophenyl)-5-imino-1-(p-tolyl)imidazolidine-2,4-dithione

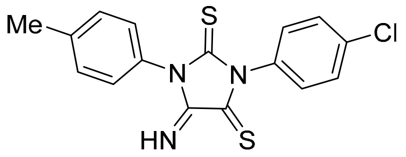

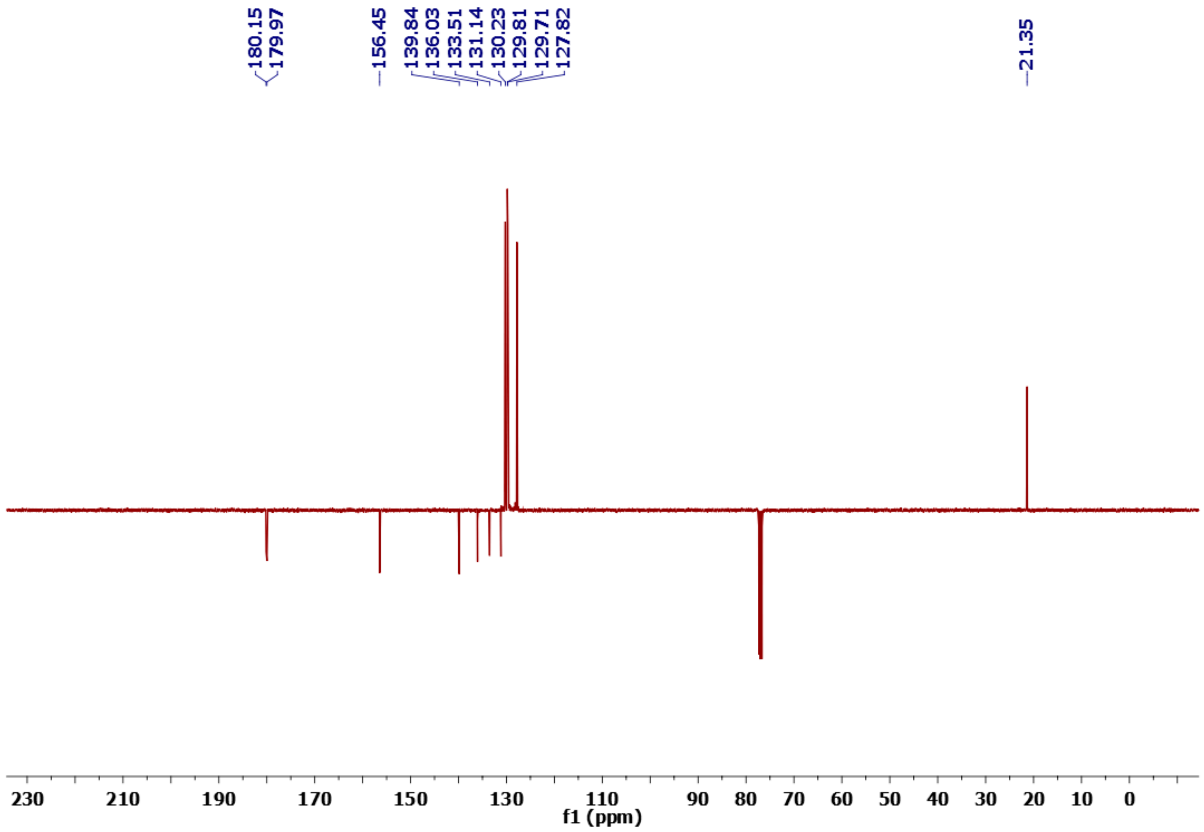

^{1}H-^{1}H-gCOSYAD NMR ($CDCl_3$) spectrum of 3-(4-chlorophenyl)-5-imino-1-(p-tolyl)imidazolidine-2,4-dithione

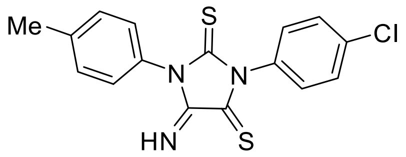

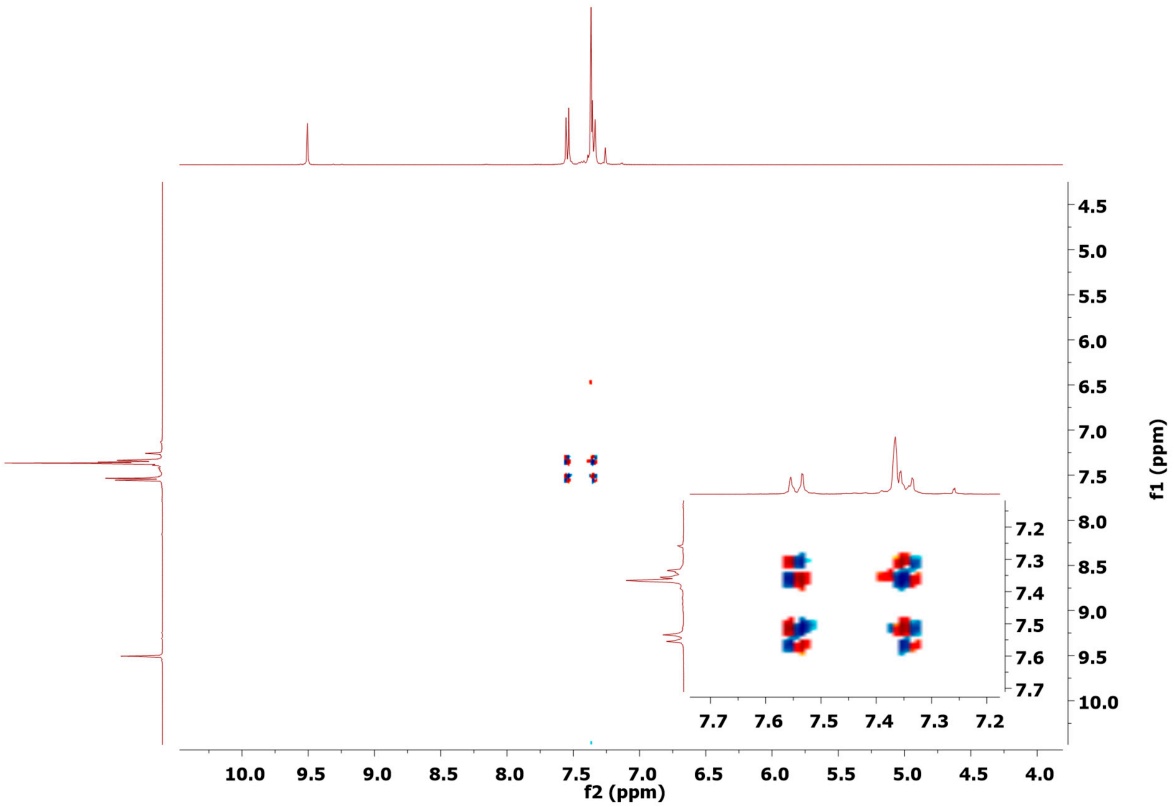

^{1}H-^{13}C-gHSQCAD NMR ($CDCl_3$) spectrum of 3-(4-chlorophenyl)-5-imino-1-(p-tolyl)imidazolidine-2,4-dithione

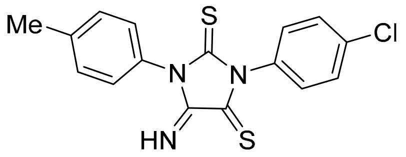

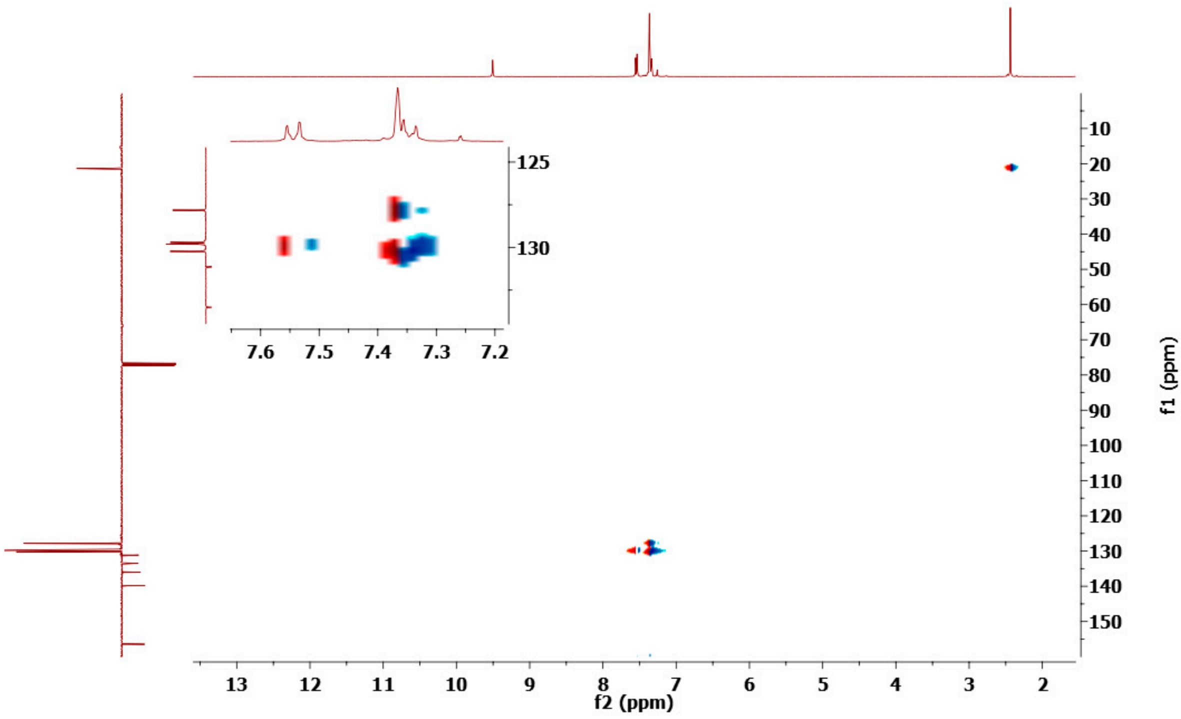

^1H-^{13}C-gHMBC NMR ($CDCl_3$) spectrum of 3-(4-chlorophenyl)-5-imino-1-(p-tolyl)imidazolidine-2,4-dithione

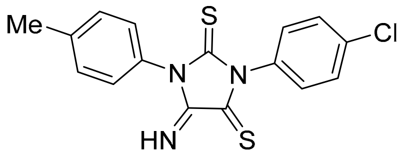

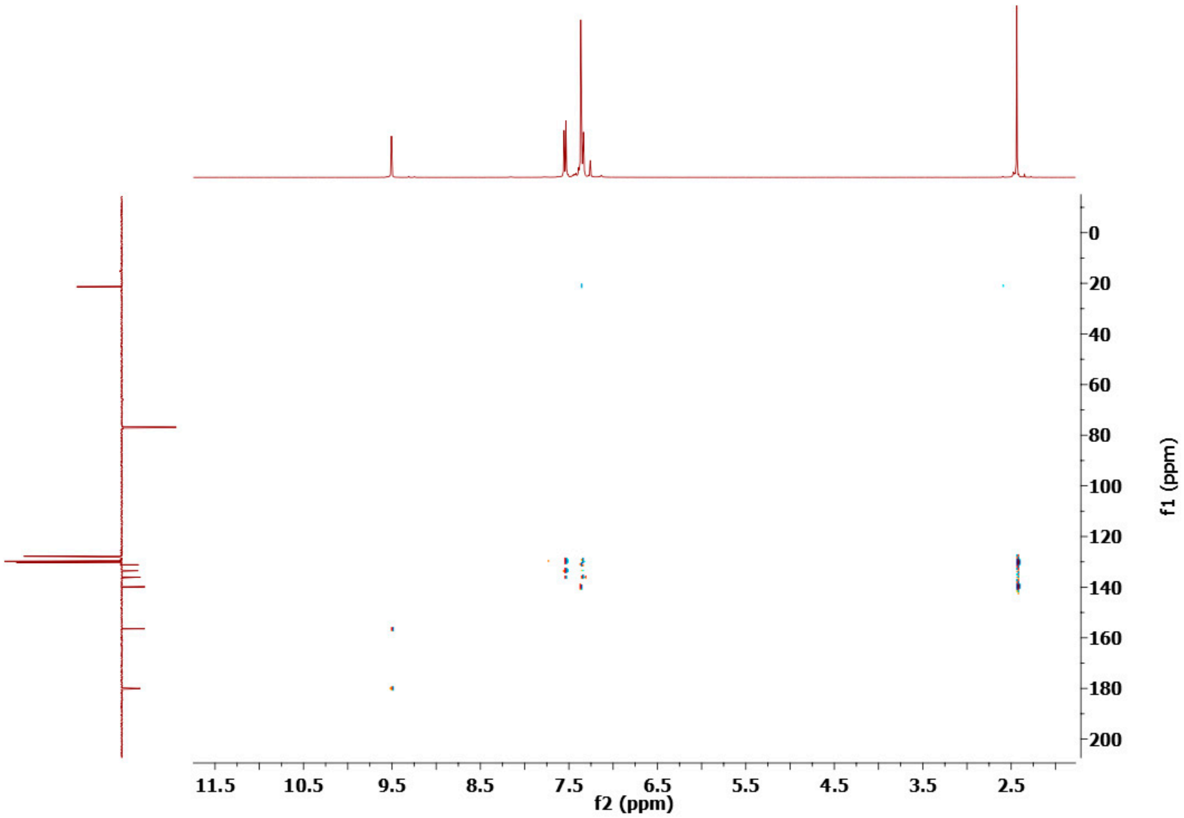

^{1}H NMR ($CDCl_3$) spectrum of 3-(4-chlorophenyl)-1-(2-fluorophenyl)-5-iminoimidazolidine-2,4-dithione (18n)

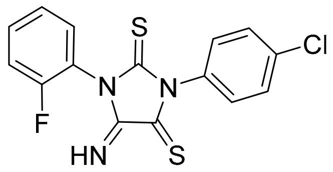

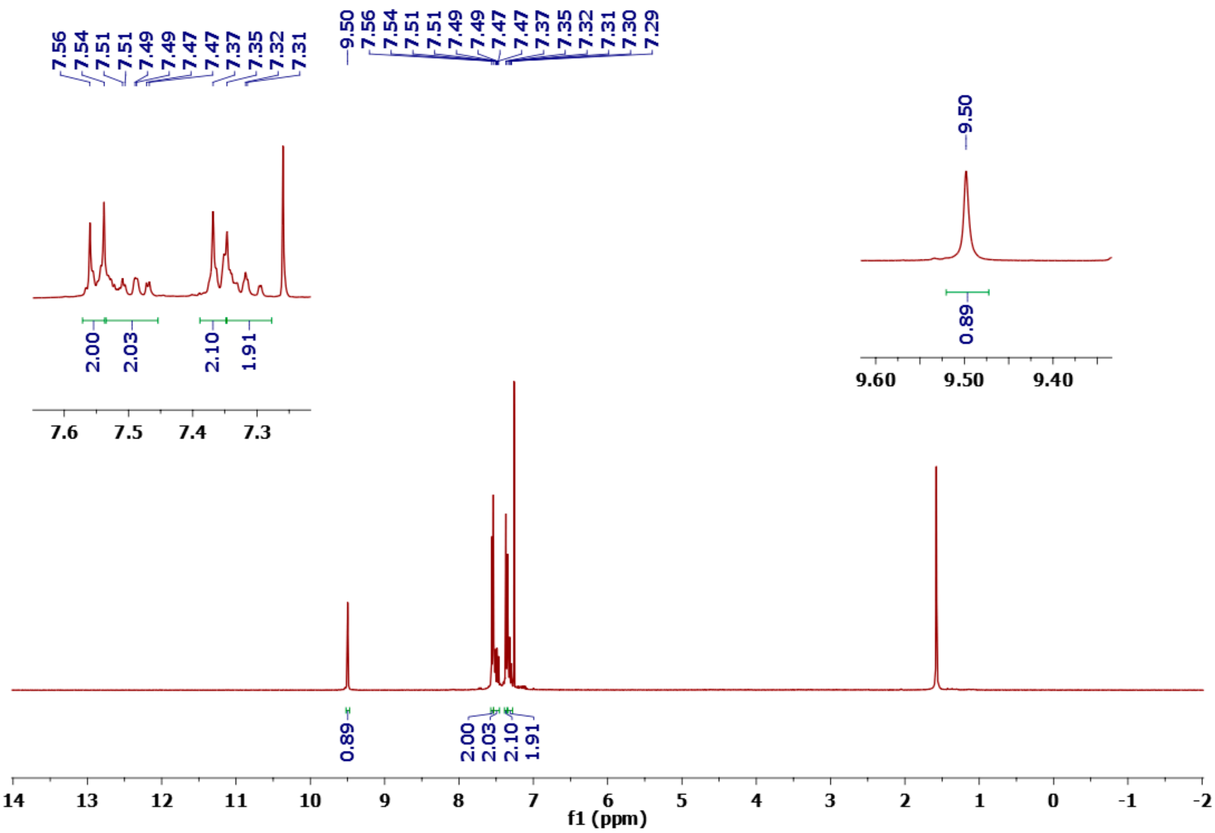

^{13}C NMR ($CDCl_3$) spectrum of 3-(4-chlorophenyl)-1-(2-fluorophenyl)-5-iminoimidazolidine-2,4-dithione

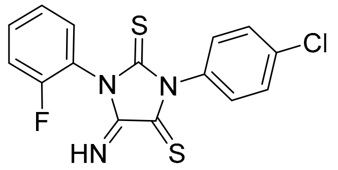

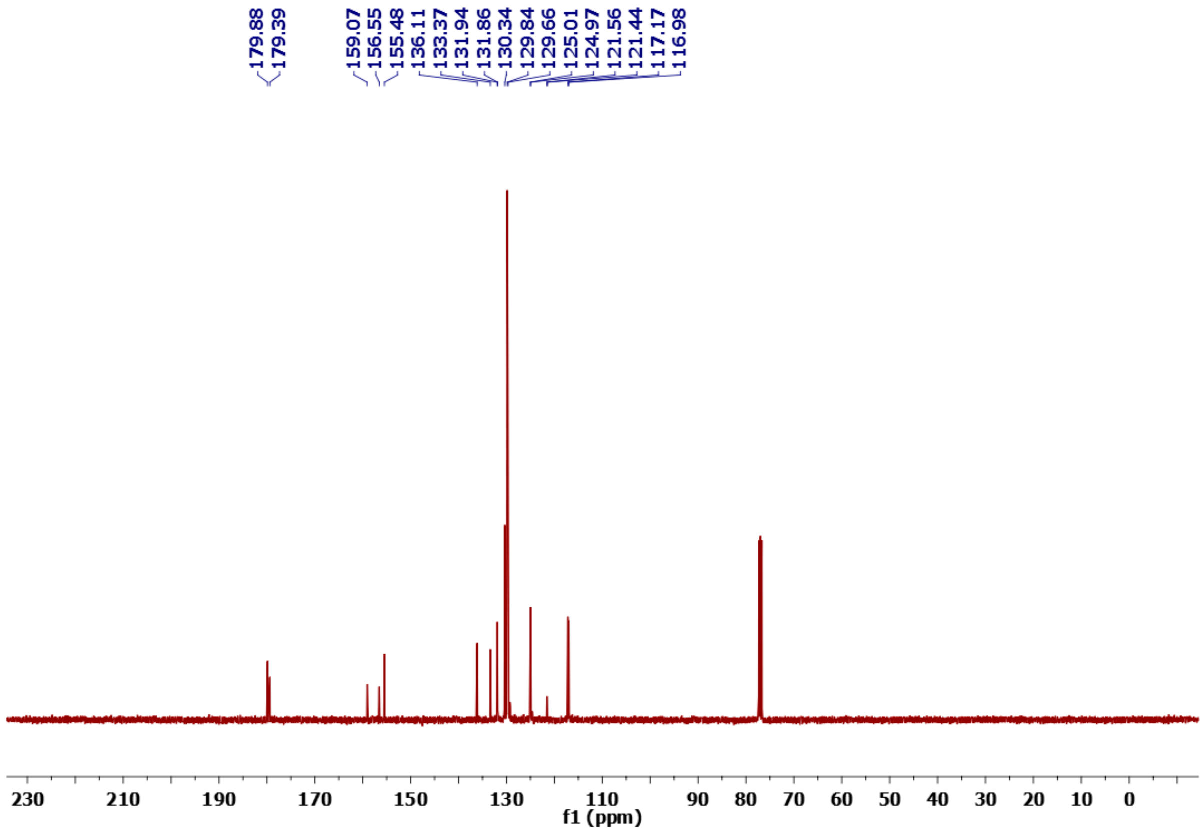

^{13}C-CRAPT NMR ($CDCl_3$) spectrum of 3-(4-chlorophenyl)-1-(2-fluorophenyl)-5-iminoimidazolidine-2,4-dithione

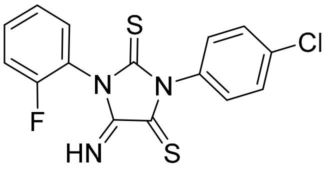

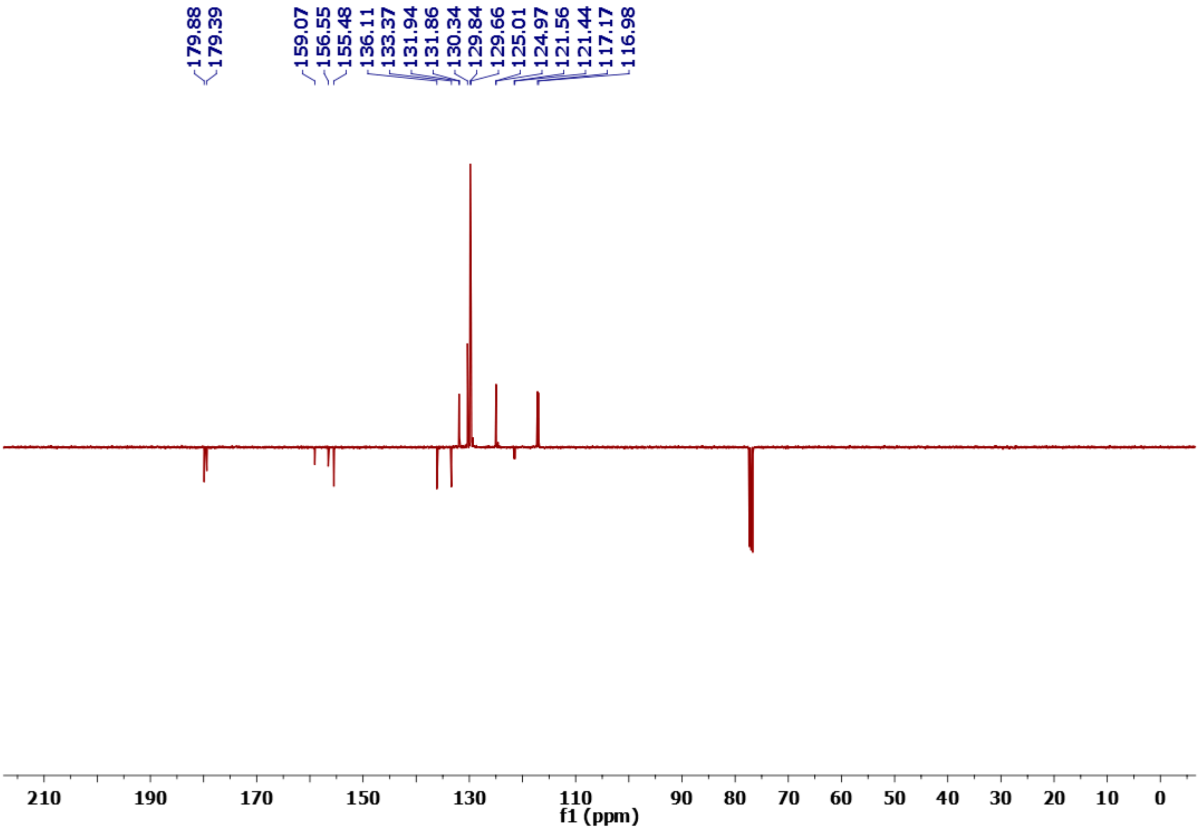

^{1}H-^{1}H-gCOSYAD NMR ($CDCl_3$) spectrum of 3-(4-chlorophenyl)-1-(2-fluorophenyl)-5-iminoimidazolidine-2,4-dithione

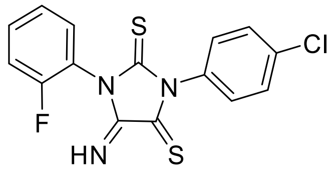

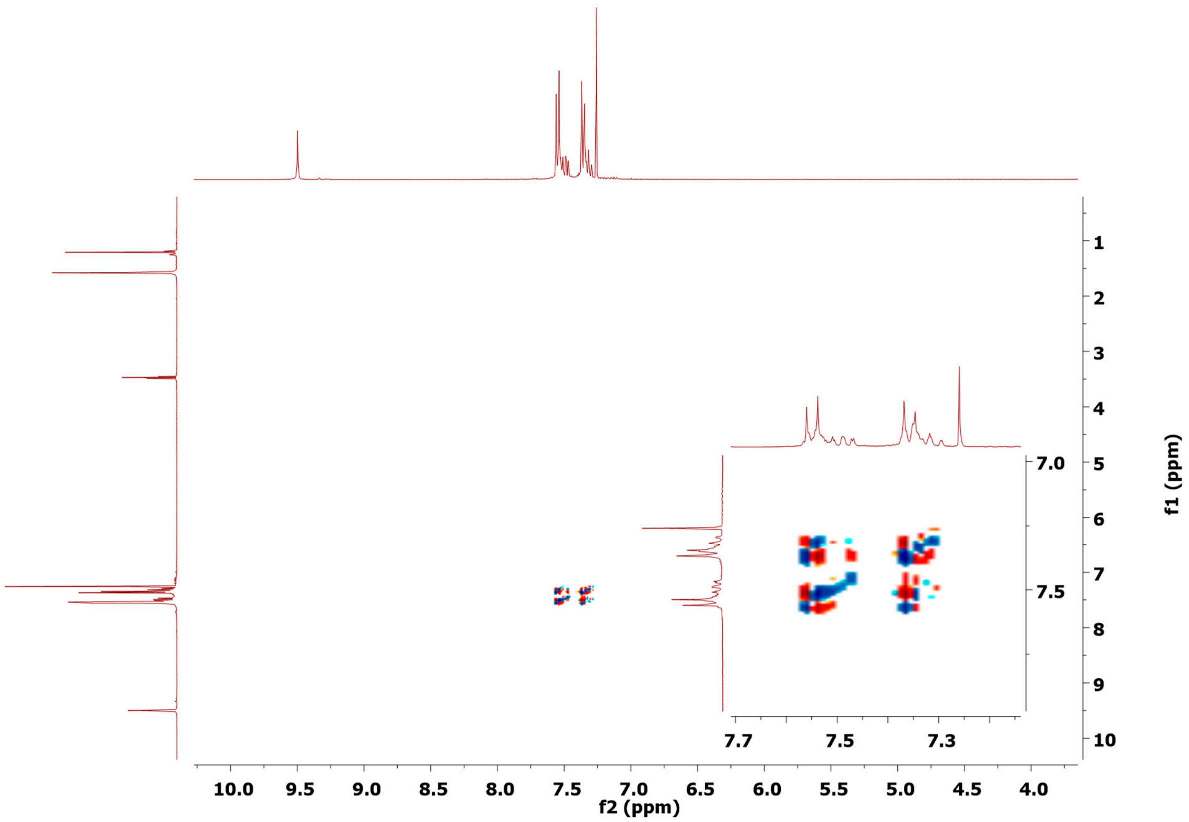

^{1}H-^{13}C-gHSQCAD NMR ($CDCl_3$) spectrum of 3-(4-chlorophenyl)-1-(2-fluorophenyl)-5-iminoimidazolidine-2,4-dithione

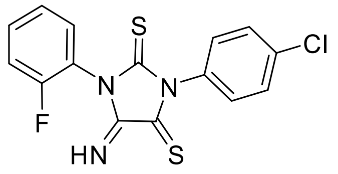

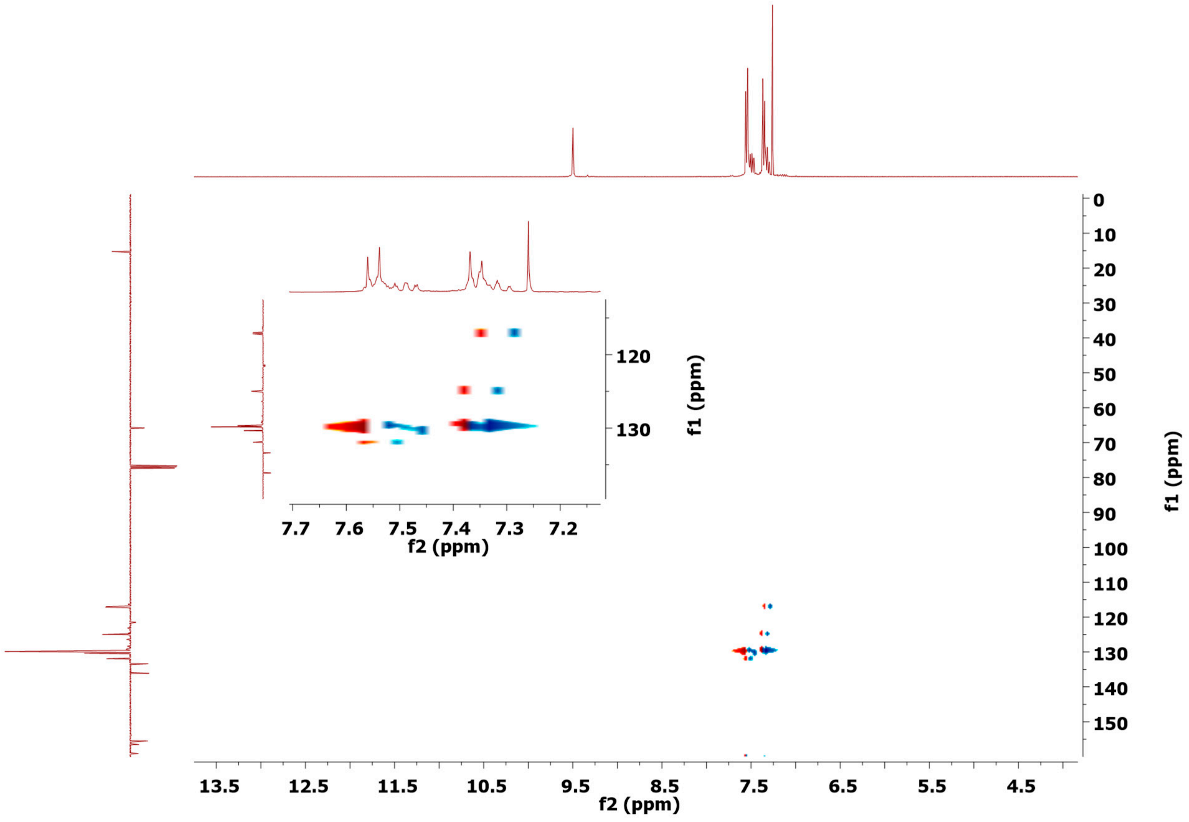

^{1}H-^{13}C-gHMBC NMR ($CDCl_3$) spectrum of 3-(4-chlorophenyl)-1-(2-fluorophenyl)-5-iminoimidazolidine-2,4-dithione

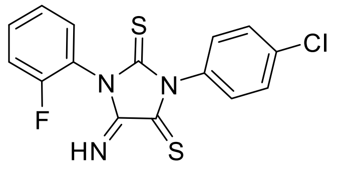

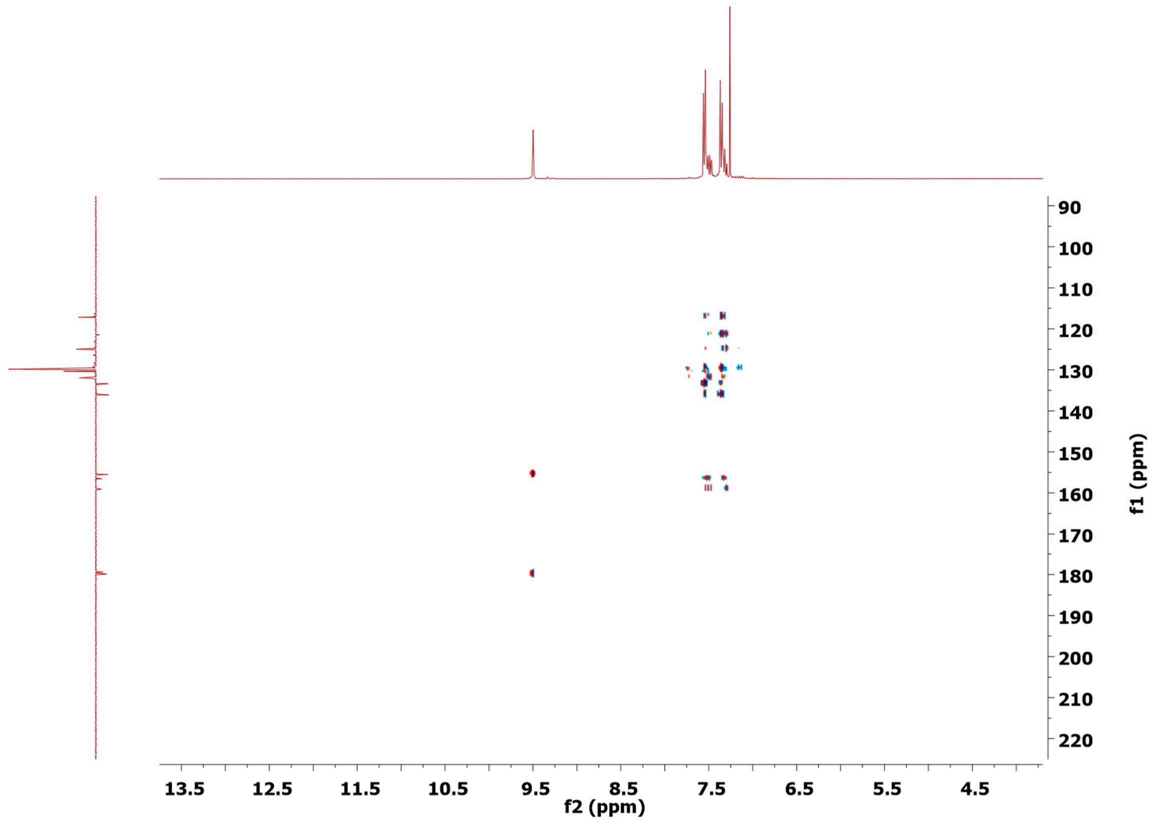

^{1}H NMR ($CDCl_3$) spectrum of 1,3-bis(4-chlorophenyl)-5-iminoimidazolidine-2,4-dithione (18o)

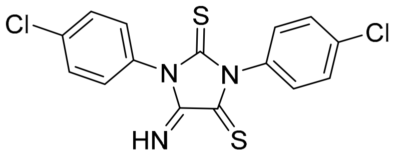

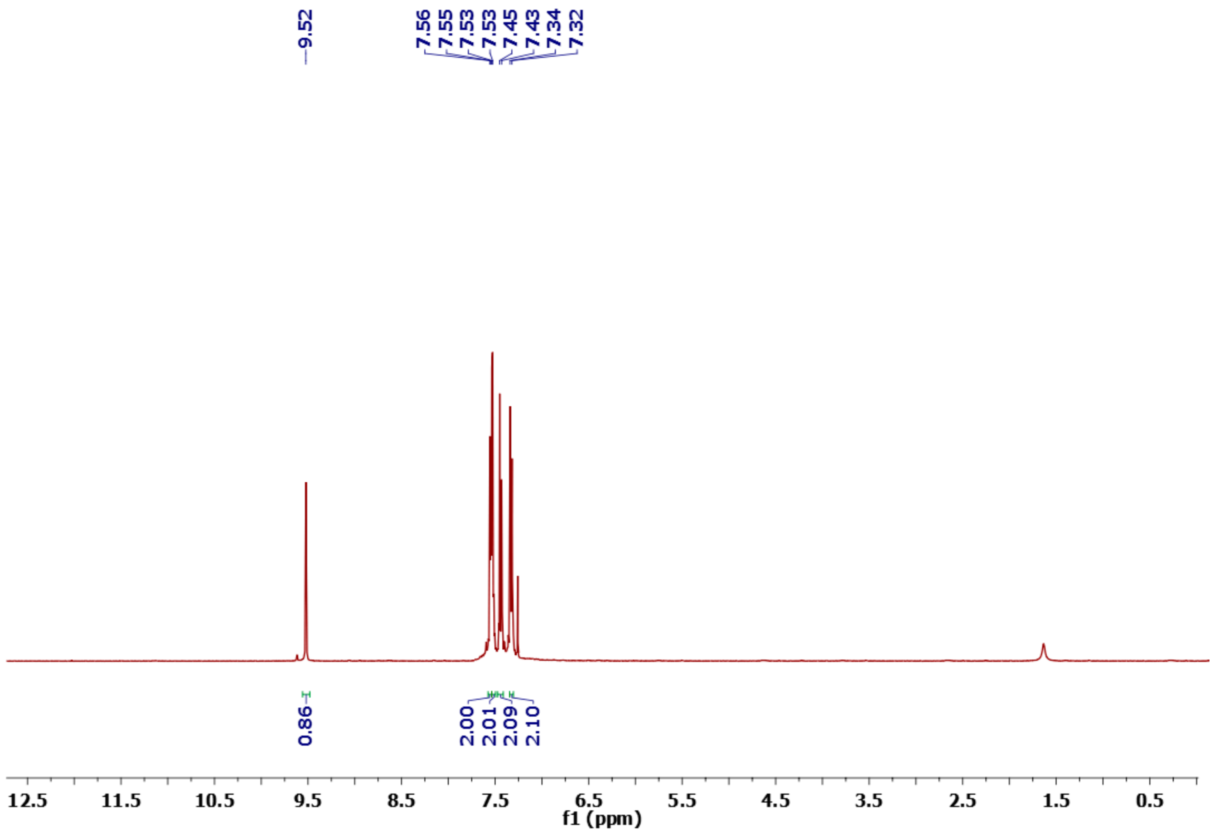

^{13}C NMR ($CDCl_3$) spectrum of 1,3-bis(4-chlorophenyl)-5-iminoimidazolidine-2,4-dithione

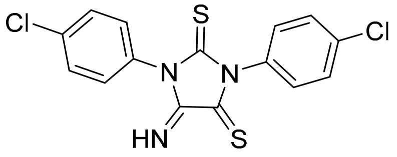

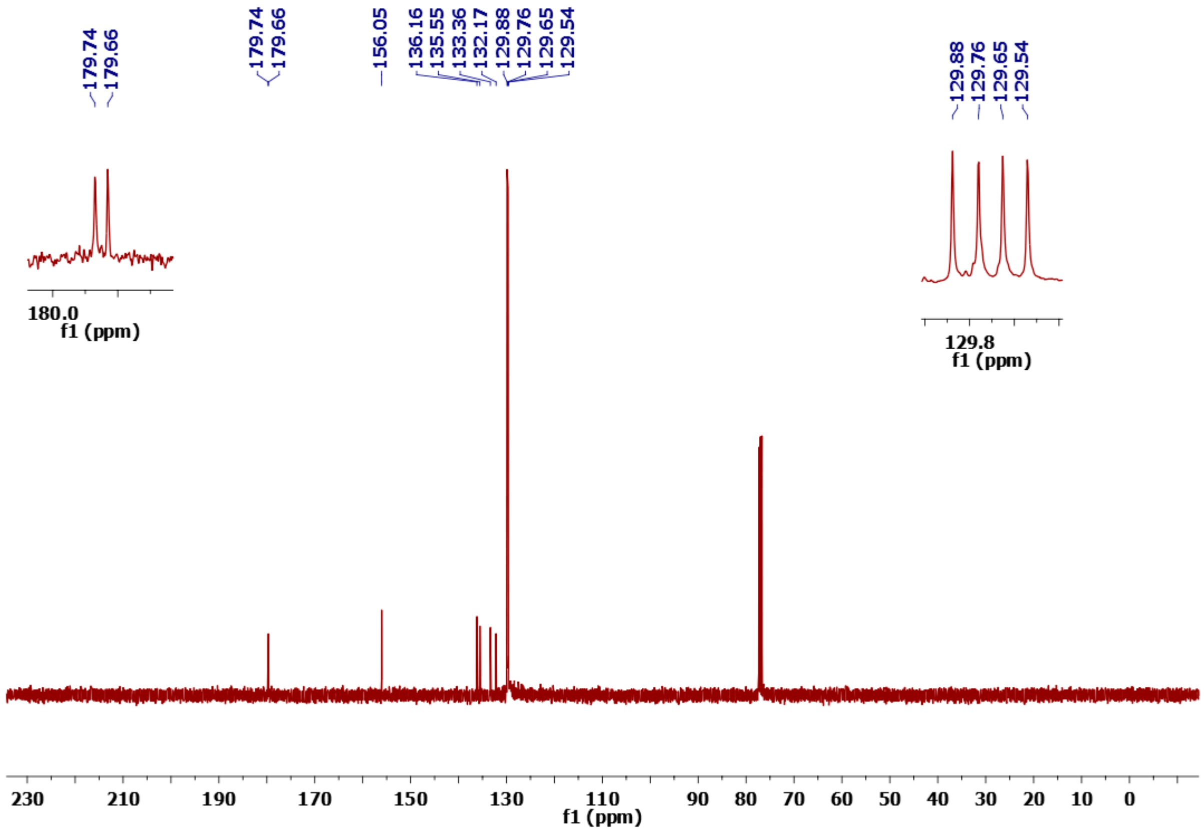

^{13}C-CRAPT NMR ($CDCl_3$) spectrum of 1,3-bis(4-chlorophenyl)-5-iminoimidazolidine-2,4-dithione

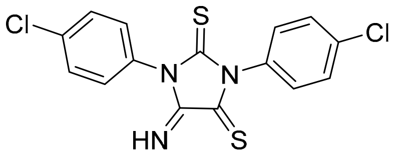

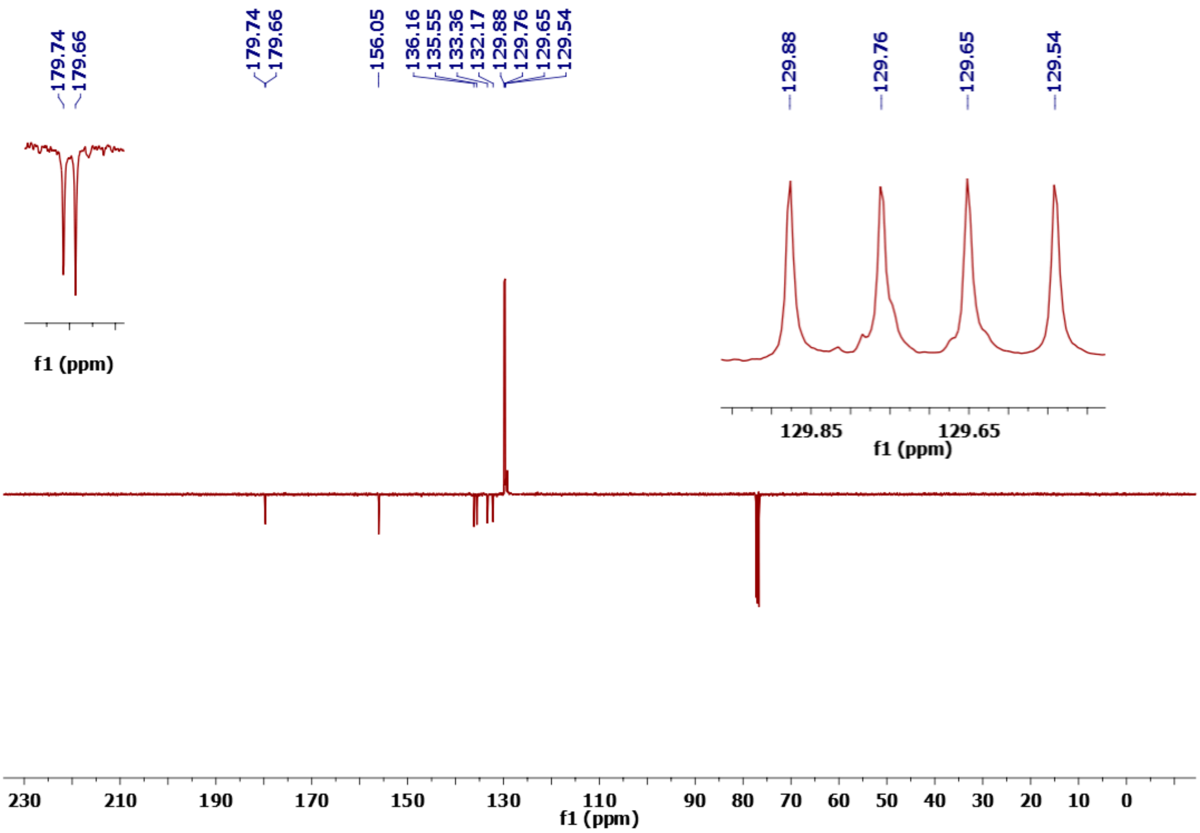

^{1}H-^{1}H-gCOSYAD NMR ($CDCl_3$) spectrum of 1,3-bis(4-chlorophenyl)-5-iminoimidazolidine-2,4-dithione

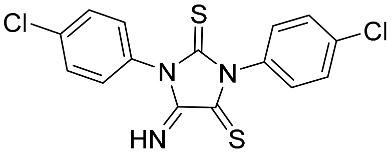

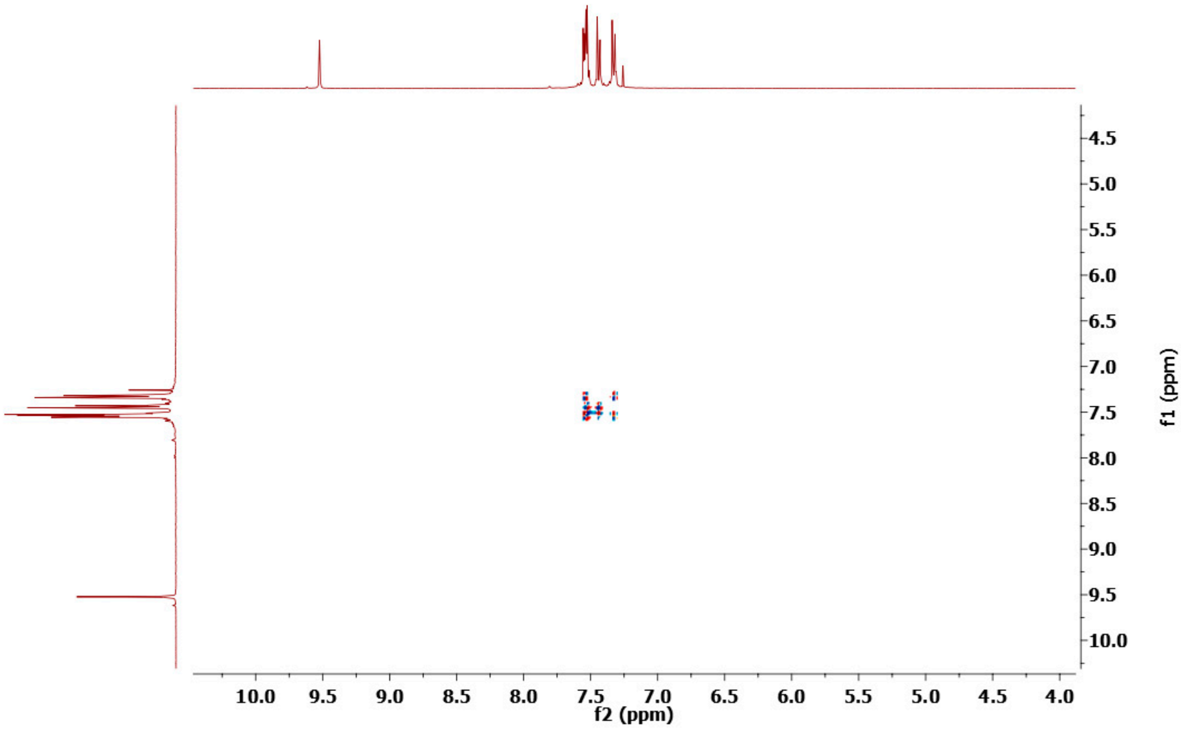

^{1}H-^{13}C-gHSQCAD NMR ($CDCl_3$) spectrum of 1,3-bis(4-chlorophenyl)-5-iminoimidazolidine-2,4-dithione

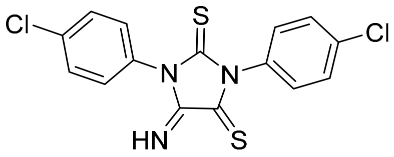

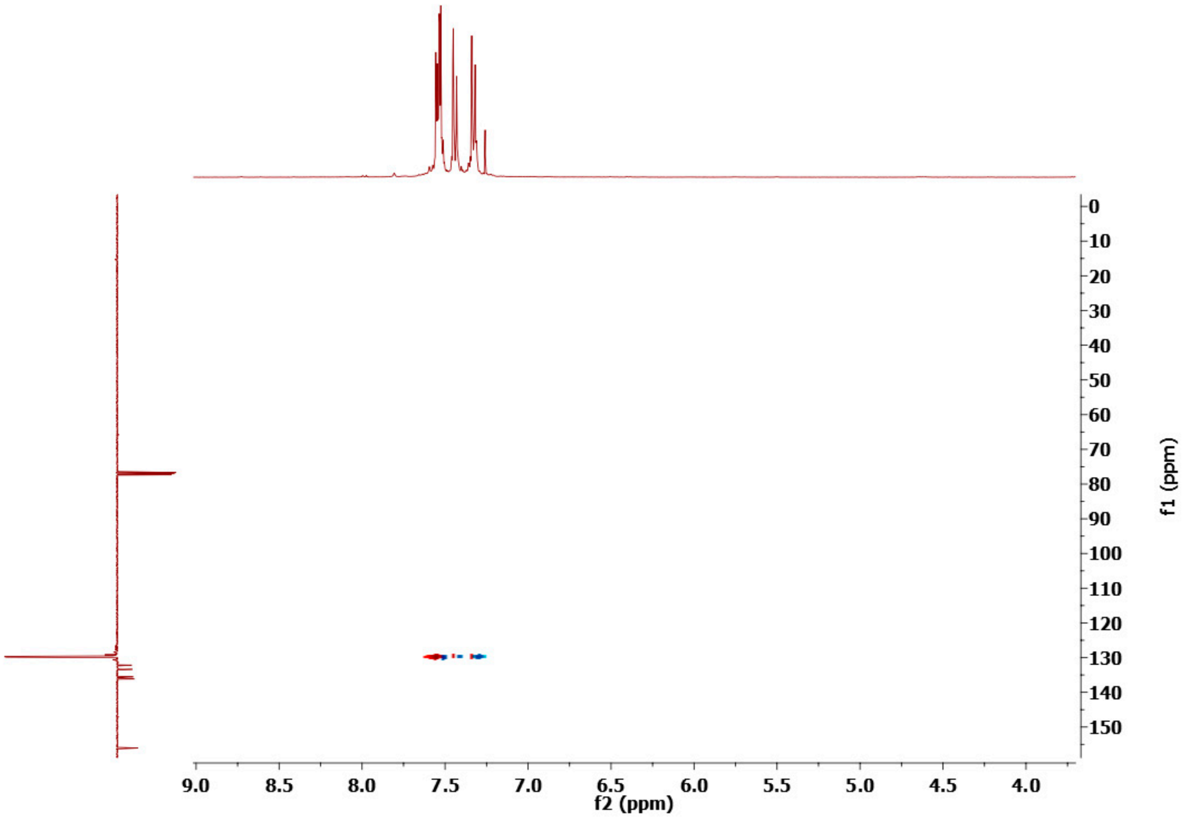

^{1}H-^{13}C-gHMBC NMR ($CDCl_3$) spectrum of 1,3-bis(4-chlorophenyl)-5-iminoimidazolidine-2,4-dithione

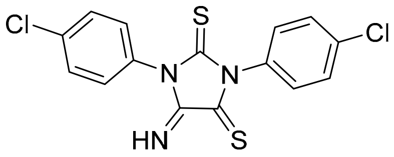

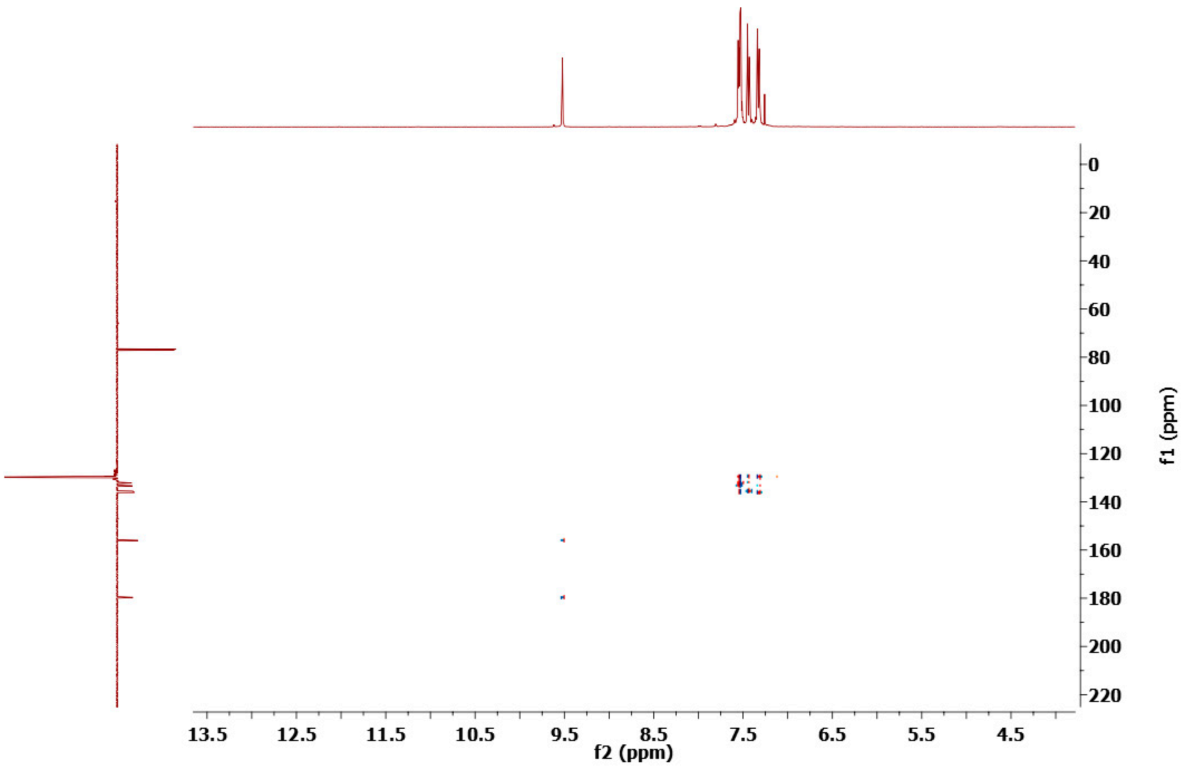

^{1}H-NMR (DMSO-d_6) spectrum of 3-(4-chlorophenyl)-5-imino-1-(4-nitrophenyl)imidazolidine-2,4-dithione (18p)

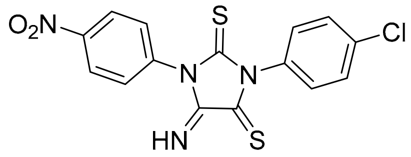

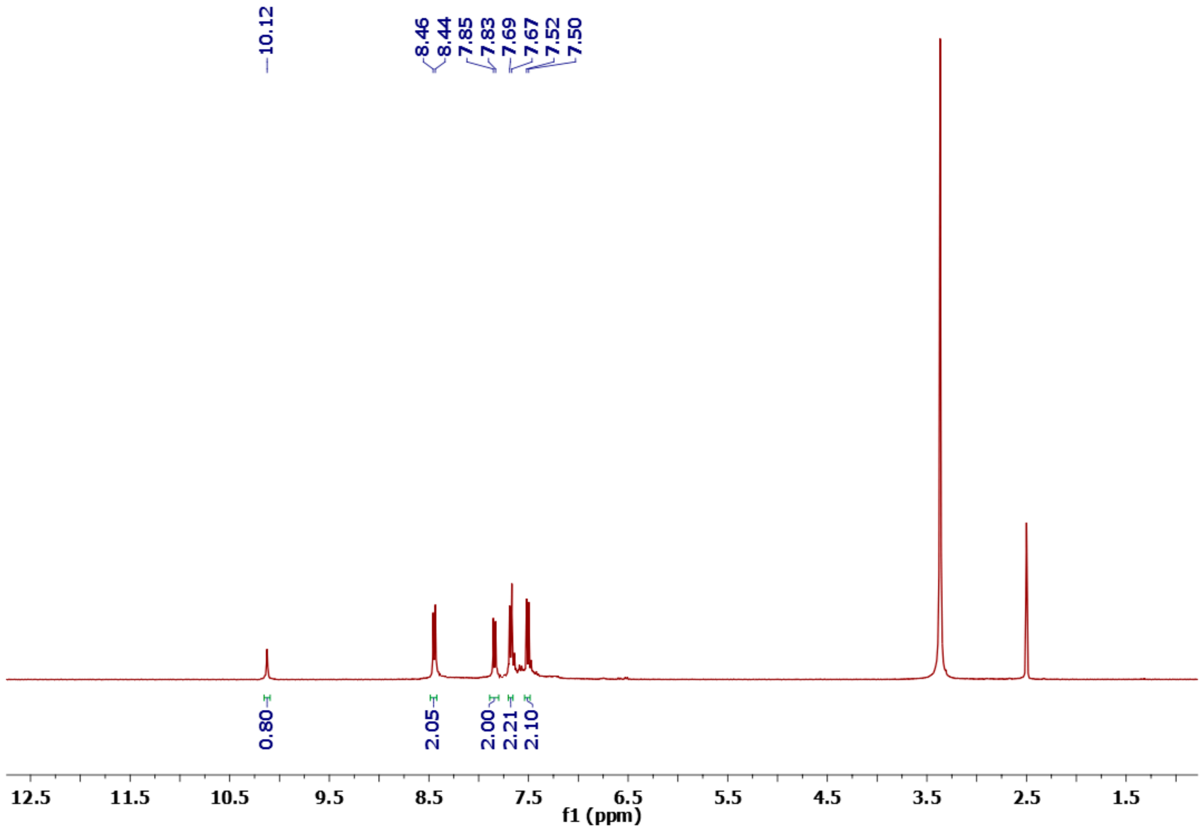

^{13}C-CRAPT NMR (DMSO-d_6) spectrum of 3-(4-chlorophenyl)-5-imino-1-(4-nitrophenyl)imidazolidine-2,4-dithione

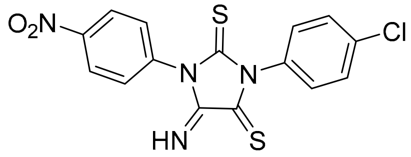

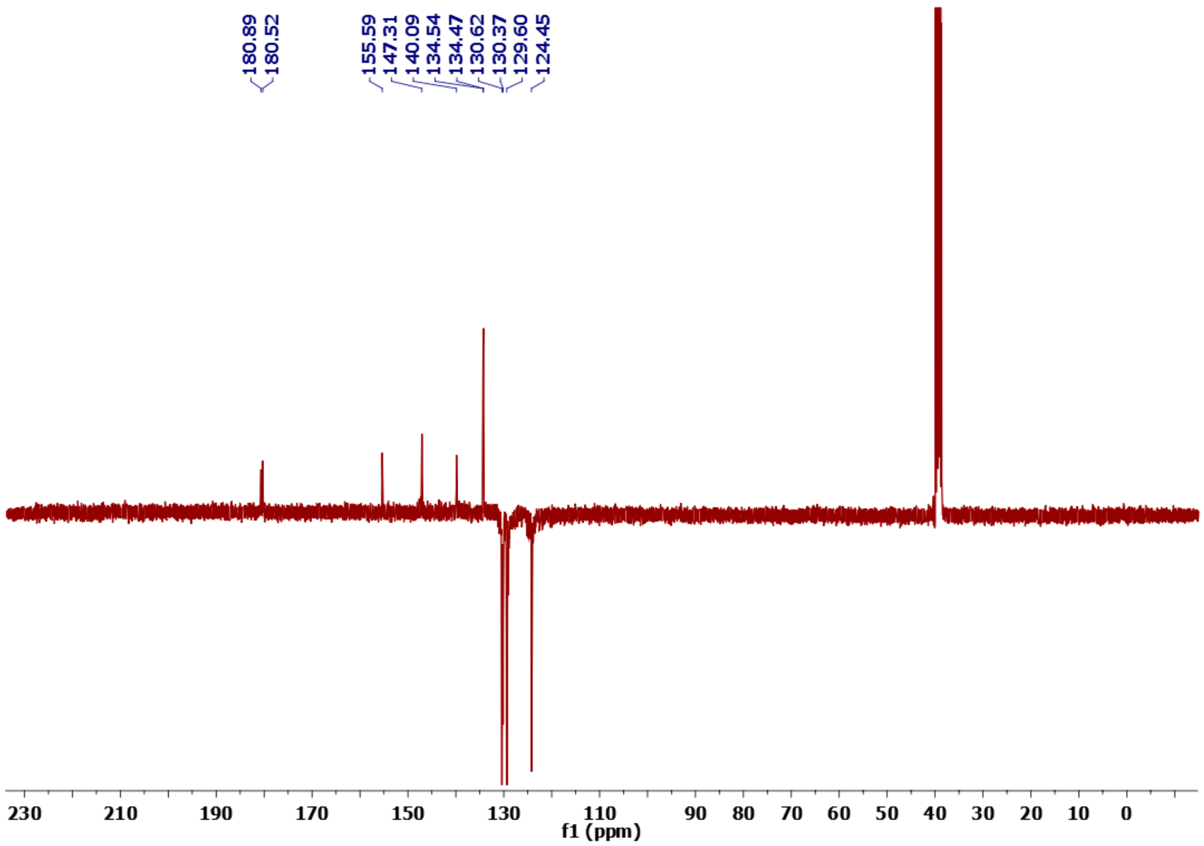

^{1}H-^{1}H-gCOSYAD NMR (DMSO-d_6) spectrum of 3-(4-chlorophenyl)-5-imino-1-(4-nitrophenyl)imidazolidine-2,4-dithione

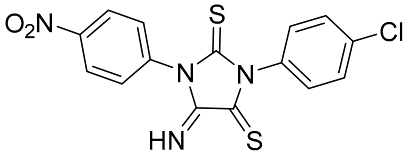

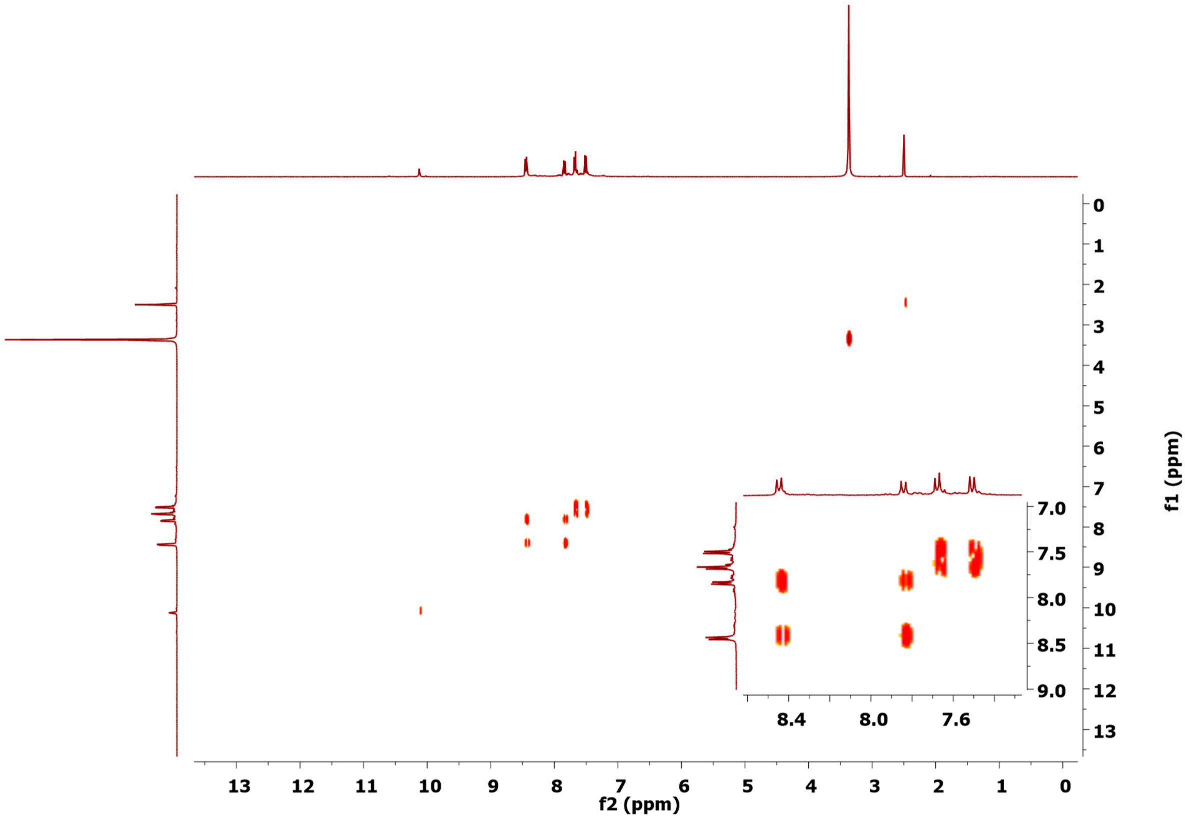

^{1}H-^{13}C-gHSQCAD NMR (DMSO-d_6) spectrum of 3-(4-chlorophenyl)-5-imino-1-(4-nitrophenyl)imidazolidine-2,4-dithione

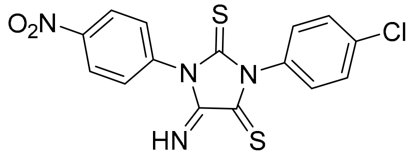

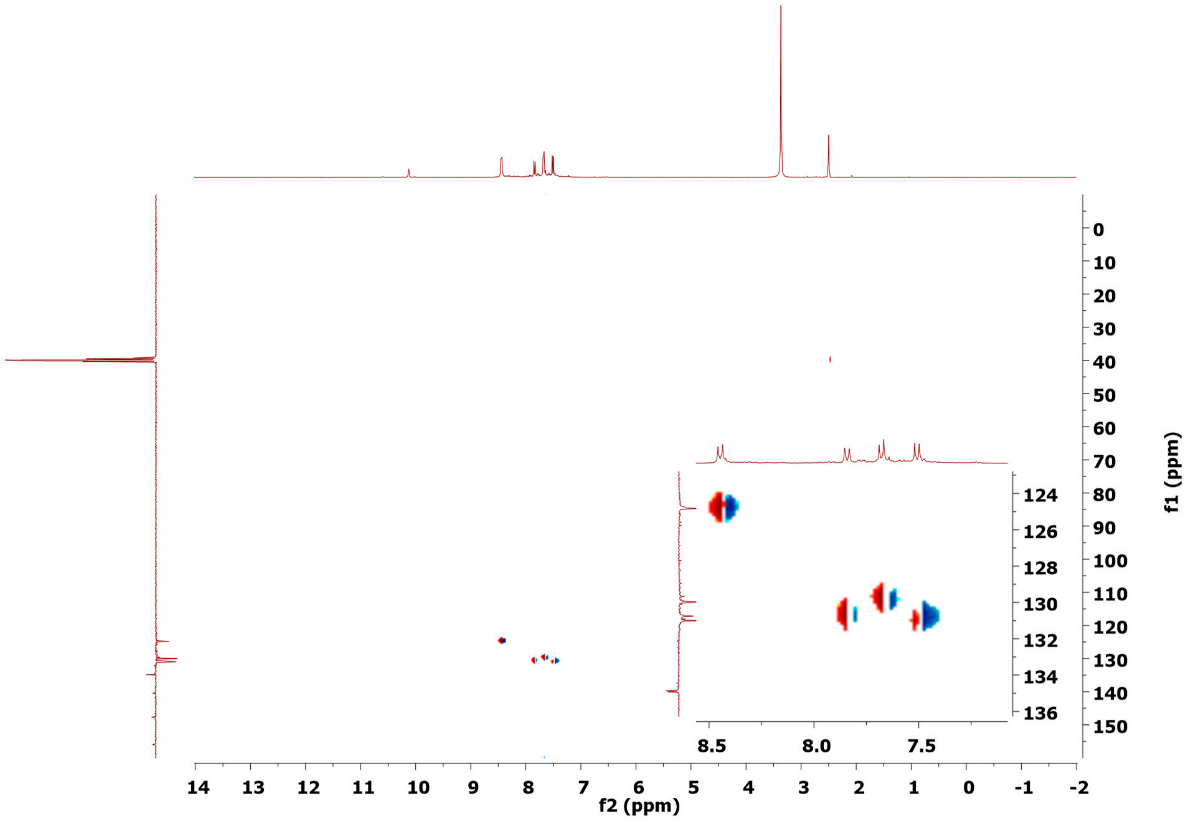

^{1}H NMR ($CDCl_3$) spectrum of 3-(4-chlorophenyl)-5-imino-1-(4-methoxyphenyl)imidazolidine-2,4-dithione (18q)

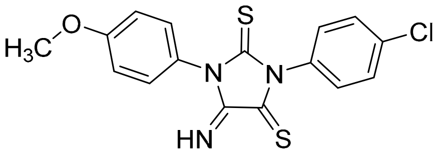

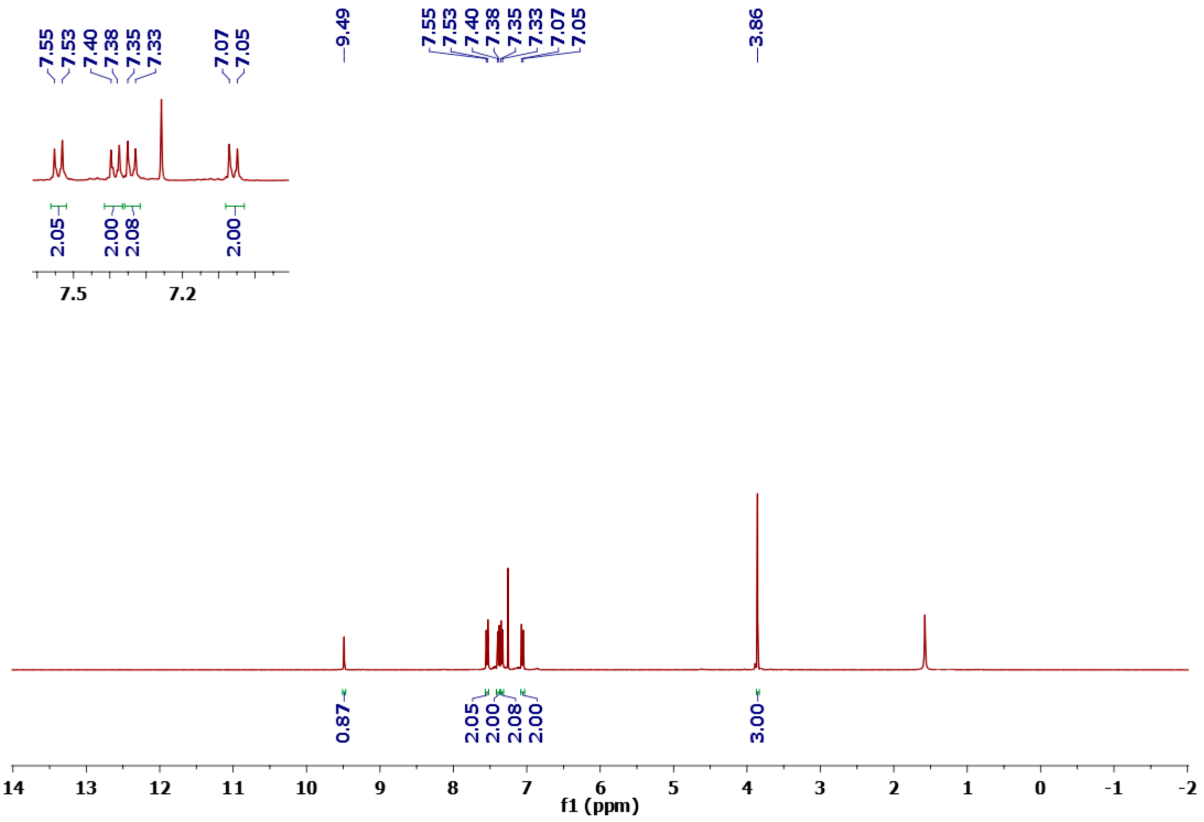

^{13}C NMR ($CDCl_3$) spectrum of 3-(4-chlorophenyl)-5-imino-1-(4-methoxyphenyl)imidazolidine-2,4-dithione

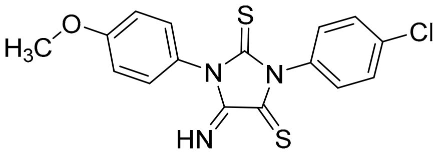

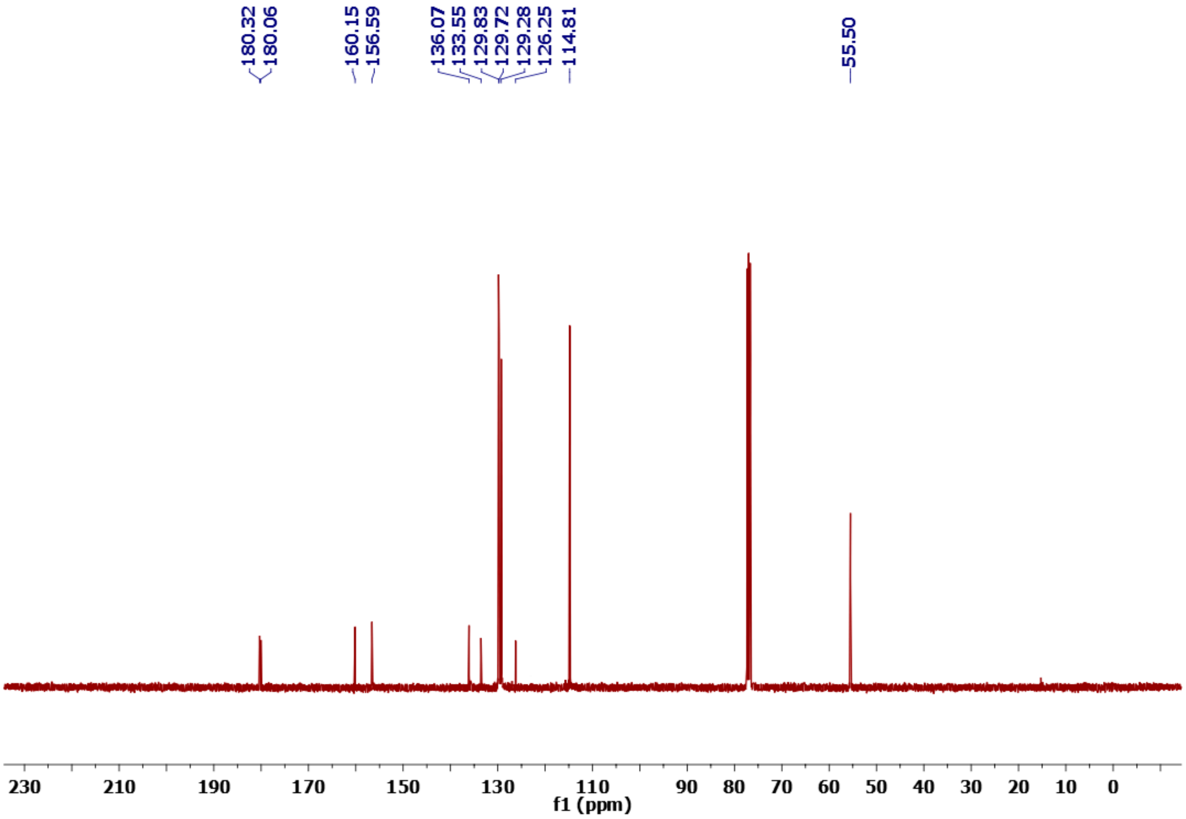

^{13}C-CRAPT NMR ($CDCl_3$) spectrum of 3-(4-chlorophenyl)-5-imino-1-(4-methoxyphenyl)imidazolidine-2,4-dithione

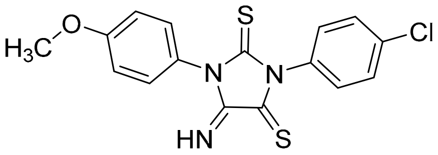

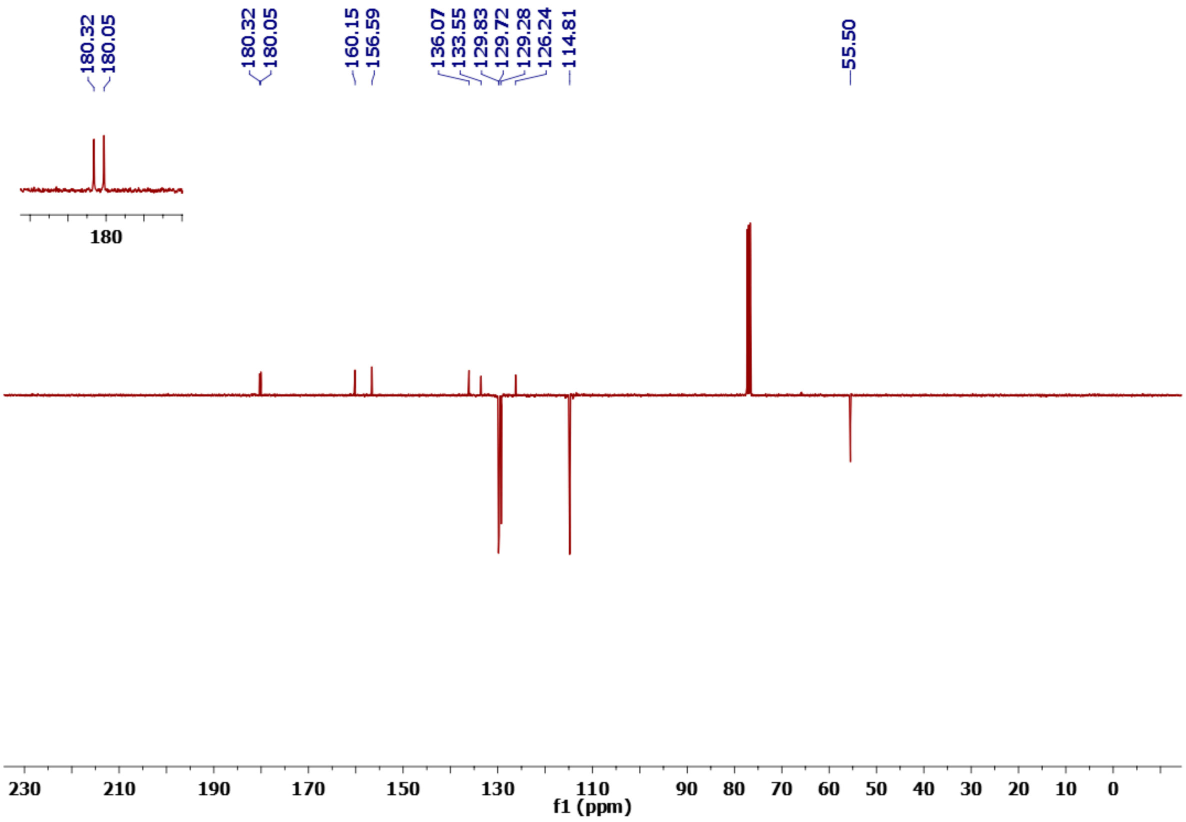

^{1}H-^{1}H-gCOSYAD NMR ($CDCl_3$) spectrum of 3-(4-chlorophenyl)-5-imino-1-(4-methoxyphenyl)imidazolidine-2,4-dithione

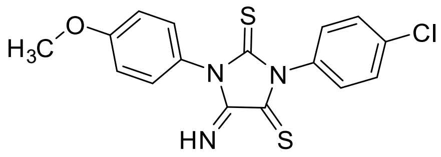

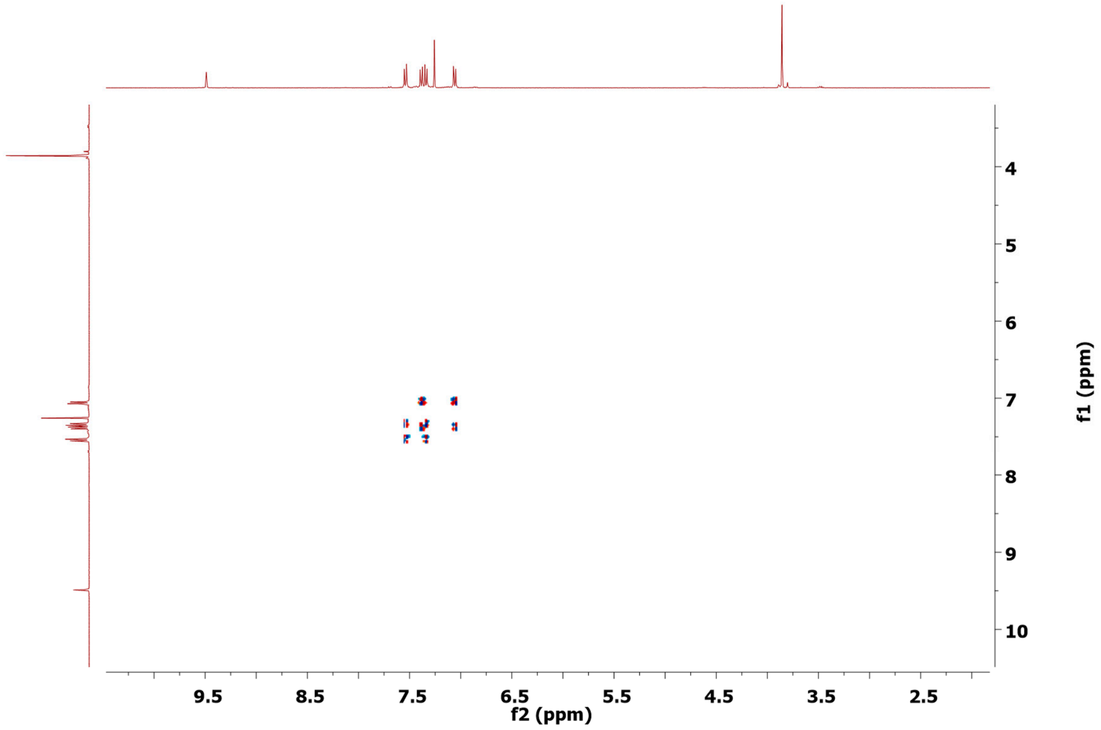

^{1}H-^{13}C-gHSQCAD NMR ($CDCl_3$) spectrum of 3-(4-chlorophenyl)-5-imino-1-(4-methoxyphenyl)imidazolidine-2,4-dithione

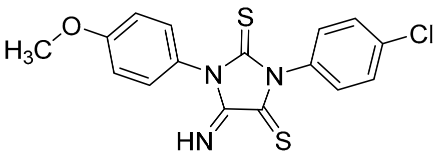

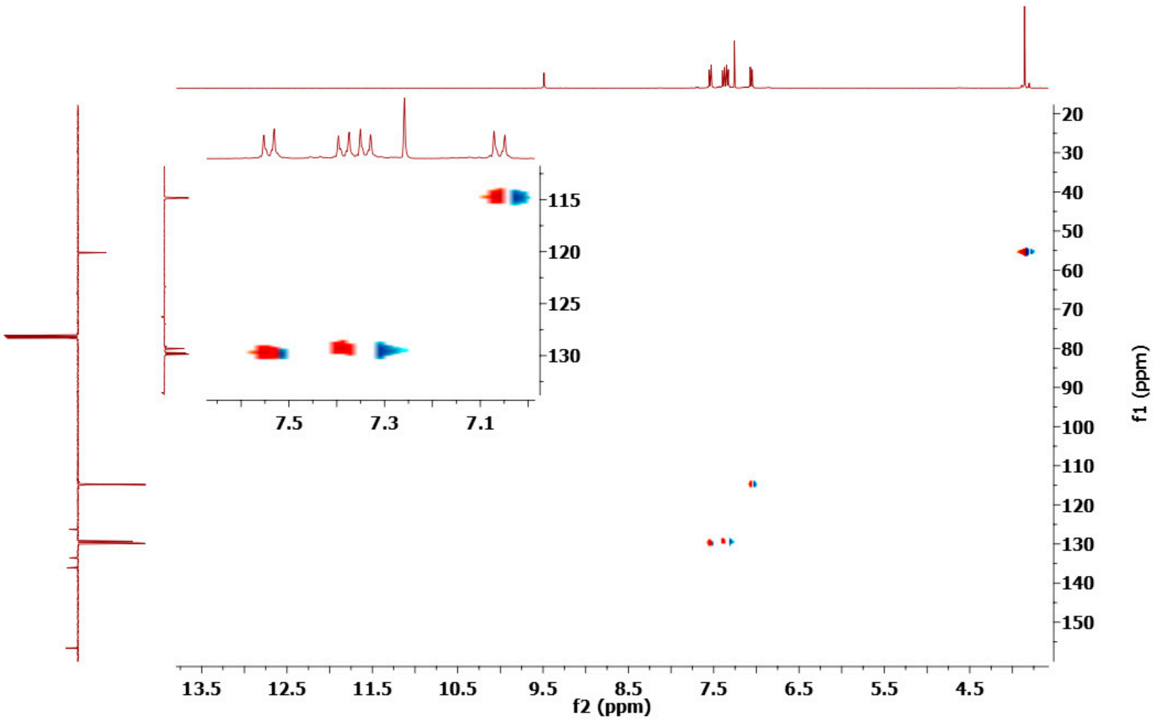

^{1}H-^{13}C-gHMBC NMR ($CDCl_3$) spectrum of 3-(4-chlorophenyl)-5-imino-1-(4-methoxyphenyl)imidazolidine-2,4-dithione

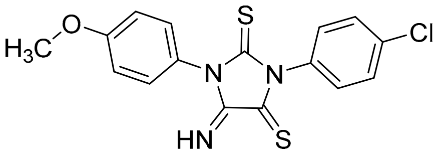

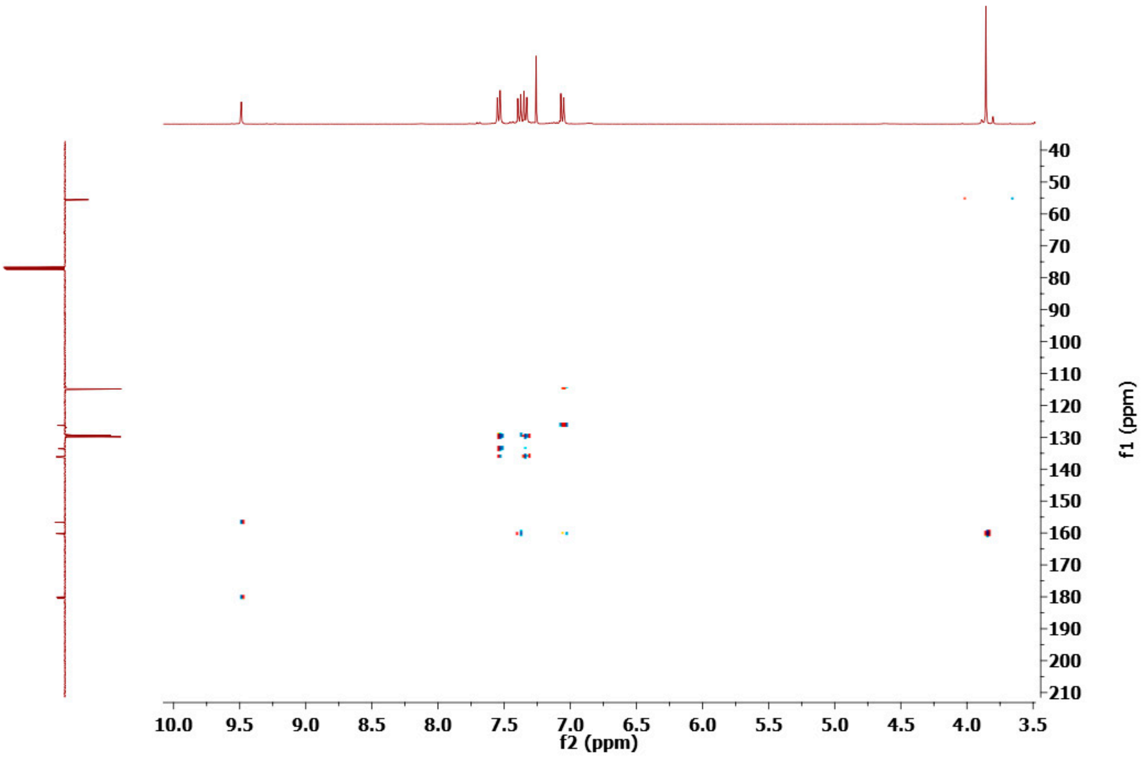

^{1}H NMR ($CDCl_3$) spectrum of (3-(2-fluorophenyl)-5-imino-2,4-dithioxoimidazolidin-1-yl)(phenyl)methanone (18r)

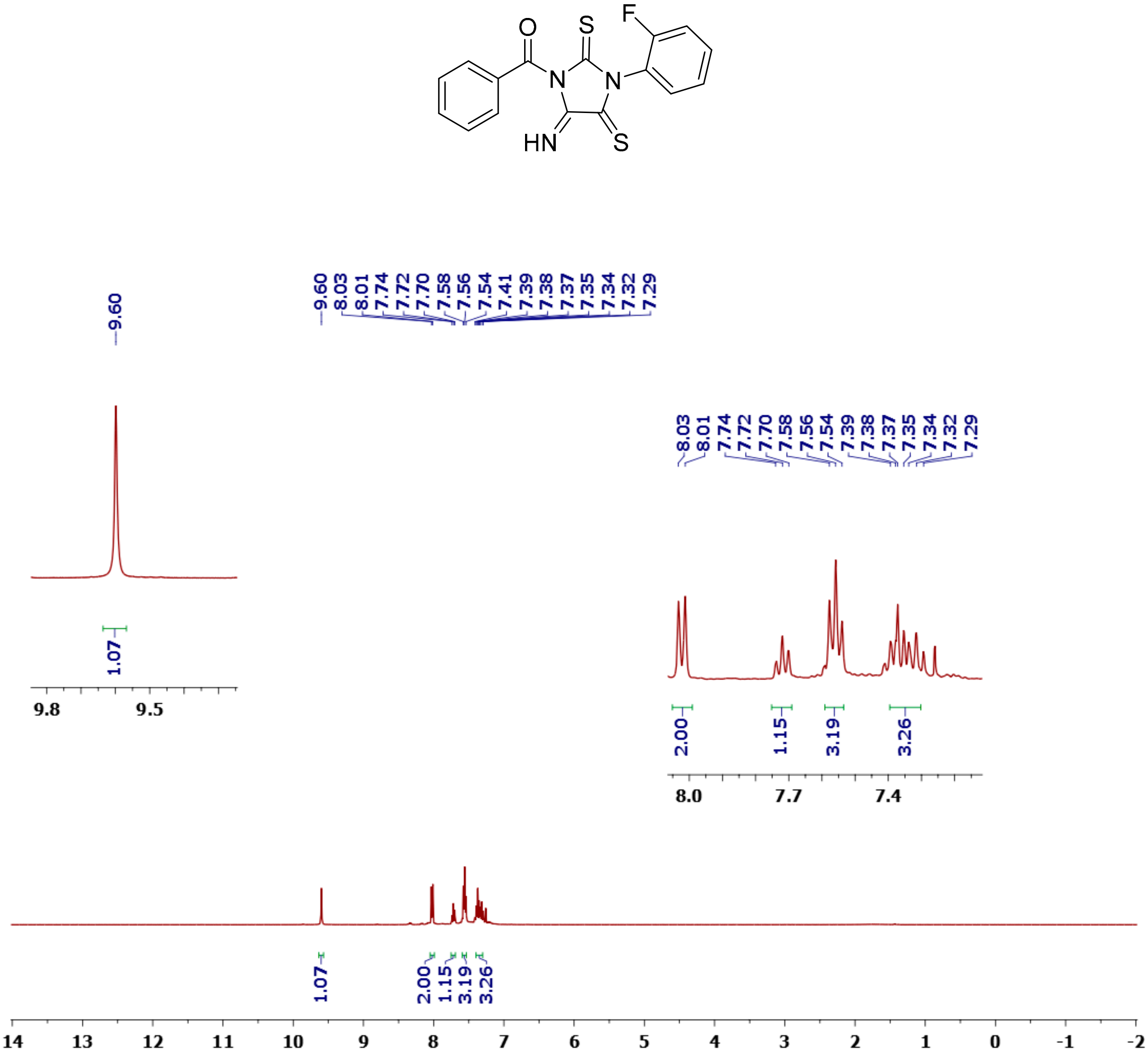

^{13}C NMR (CDCl$_3$) spectrum of (3-(2-fluorophenyl)-5-imino-2,4-dithioxoimidazolidin-1-yl)(phenyl)methanone

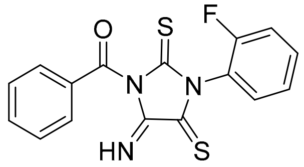

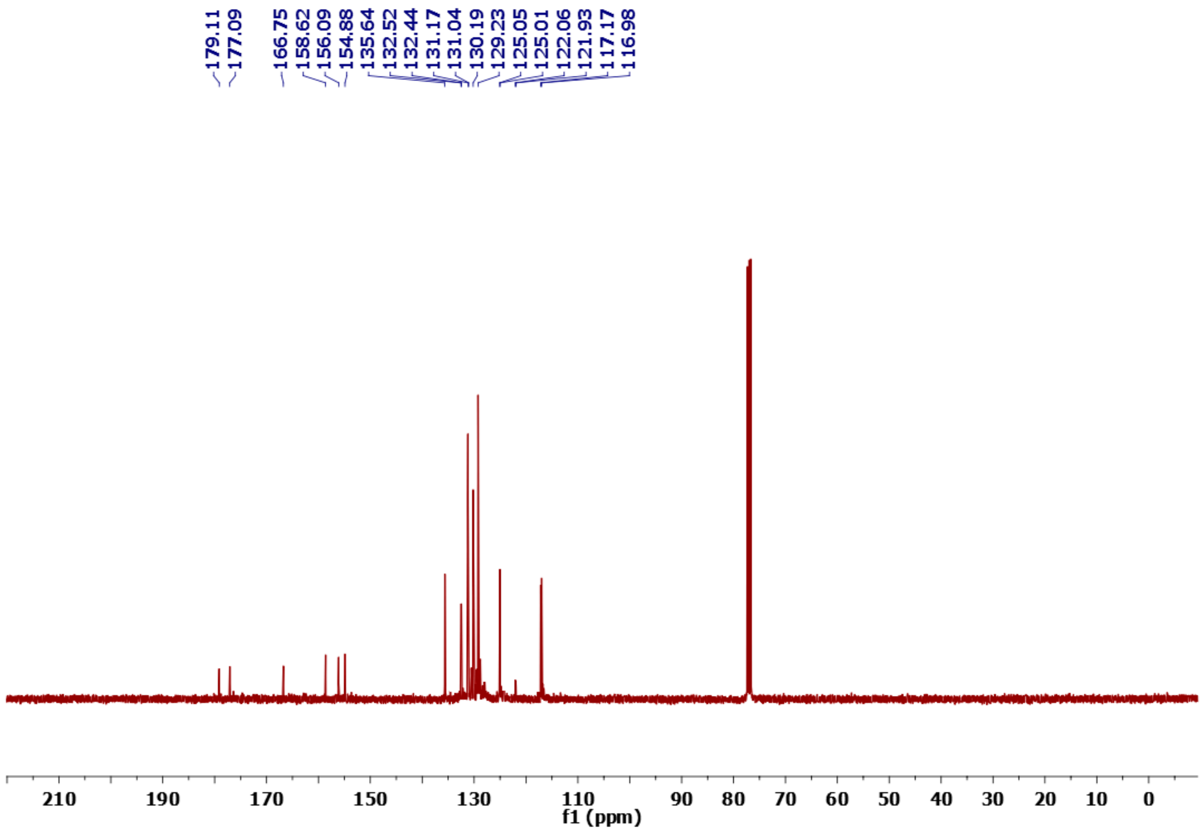

^{13}C-CRAPT NMR ($CDCl_3$) spectrum of (3-(2-fluorophenyl)-5-imino-2,4-dithioxoimidazolidin-1-yl)(phenyl)methanone

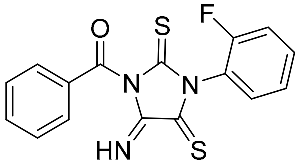

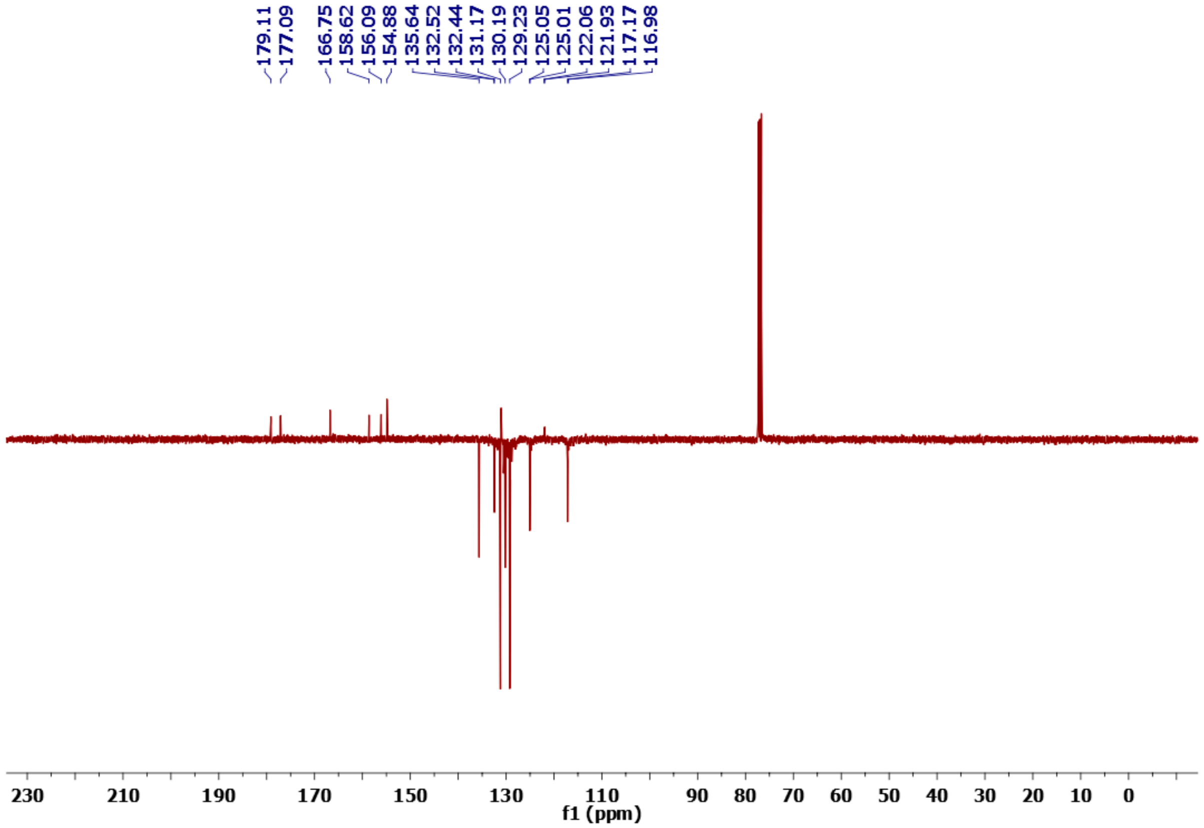

^{1}H-^{1}H-gDQCOSY NMR ($CDCl_3$) spectrum of (3-(2-fluorophenyl)-5-imino-2,4-dithioxoimidazolidin-1-yl)(phenyl)methanone

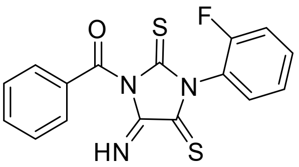

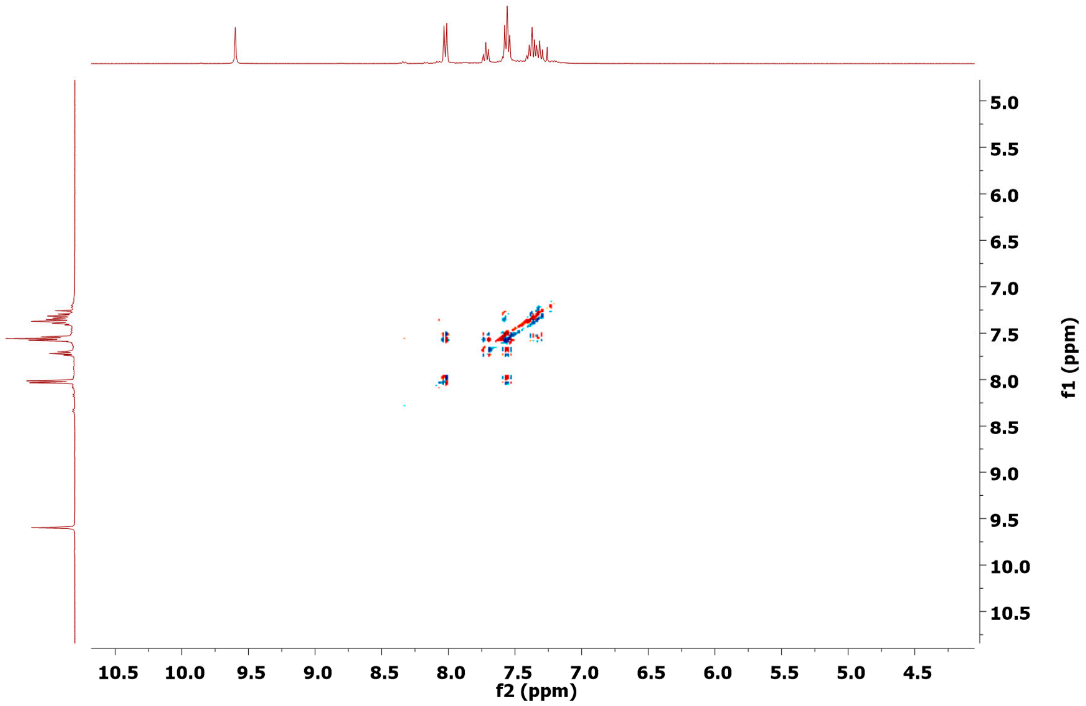

^{1}H-^{13}C-gHSQCAD NMR ($CDCl_3$) spectrum of (3-(2-fluorophenyl)-5-imino-2,4-dithioxoimidazolidin-1-yl)(phenyl)methanone

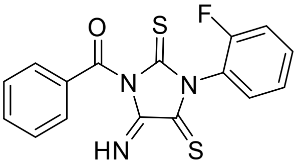

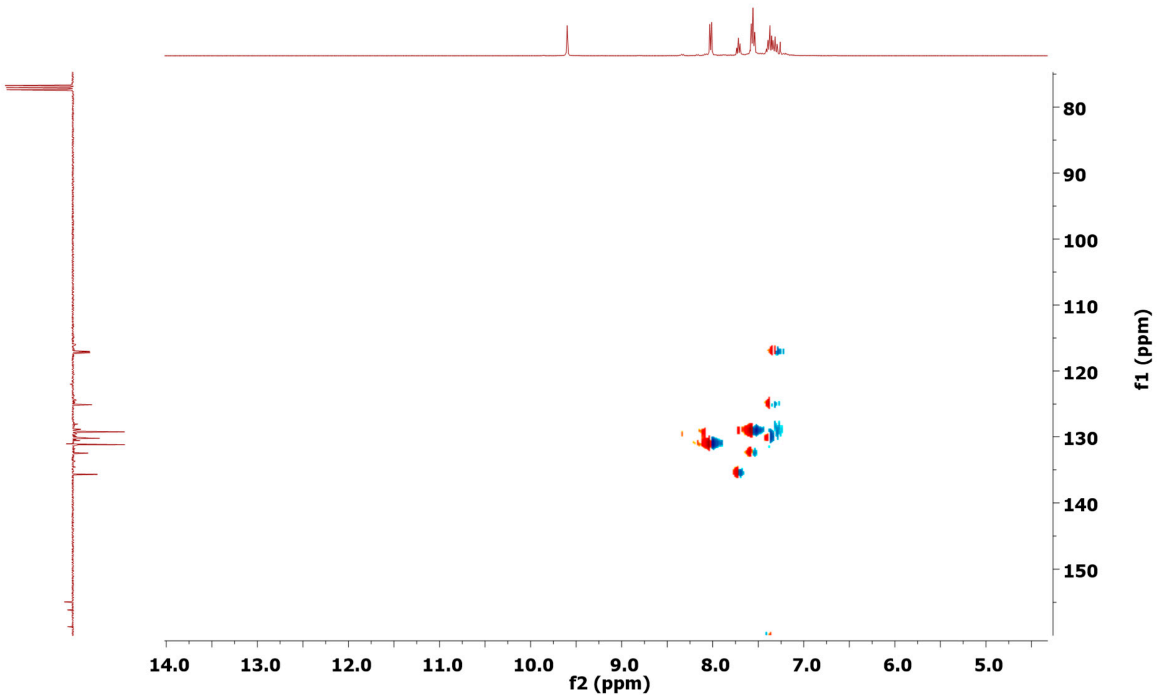

^{1}H-^{13}C-gHMBC NMR ($CDCl_3$) spectrum of (3-(2-fluorophenyl)-5-imino-2,4-dithioxoimidazolidin-1-yl)(phenyl)methanone

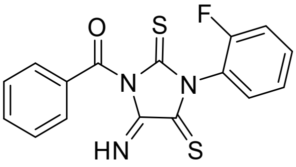

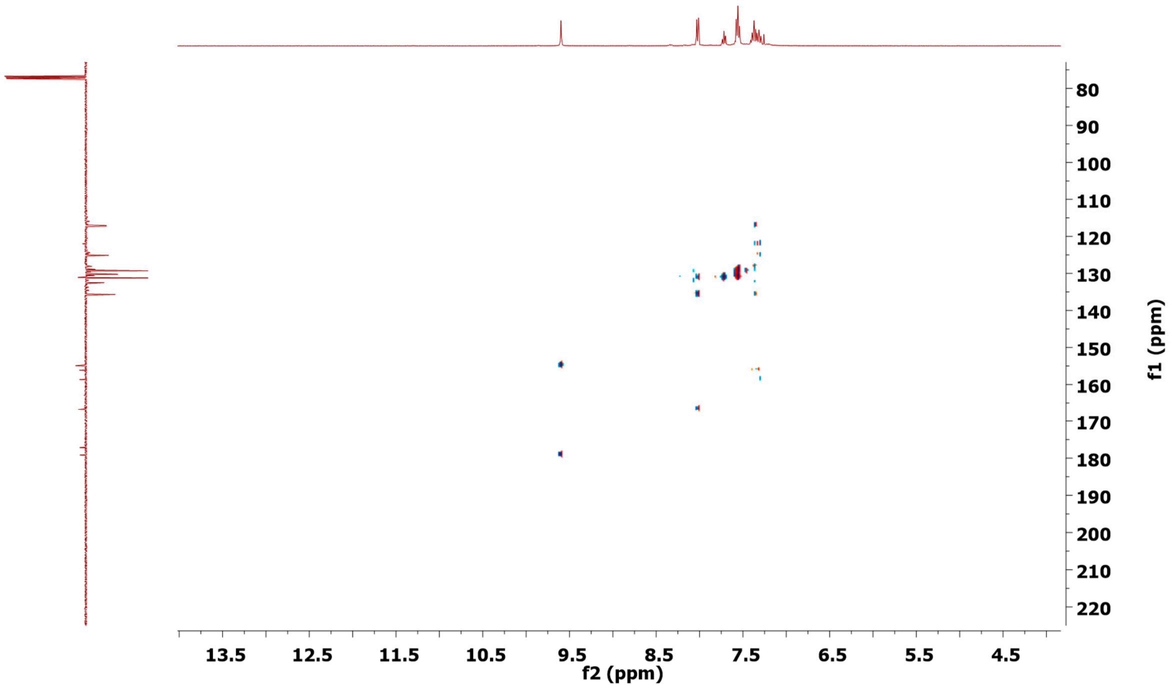

^{1}H NMR ($CDCl_3$) spectrum of 3-(2-fluorophenyl)-5-imino-1-phenylimidazolidine-2,4-dithione (18s)

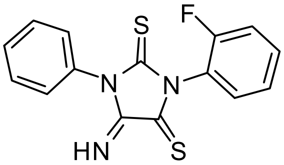

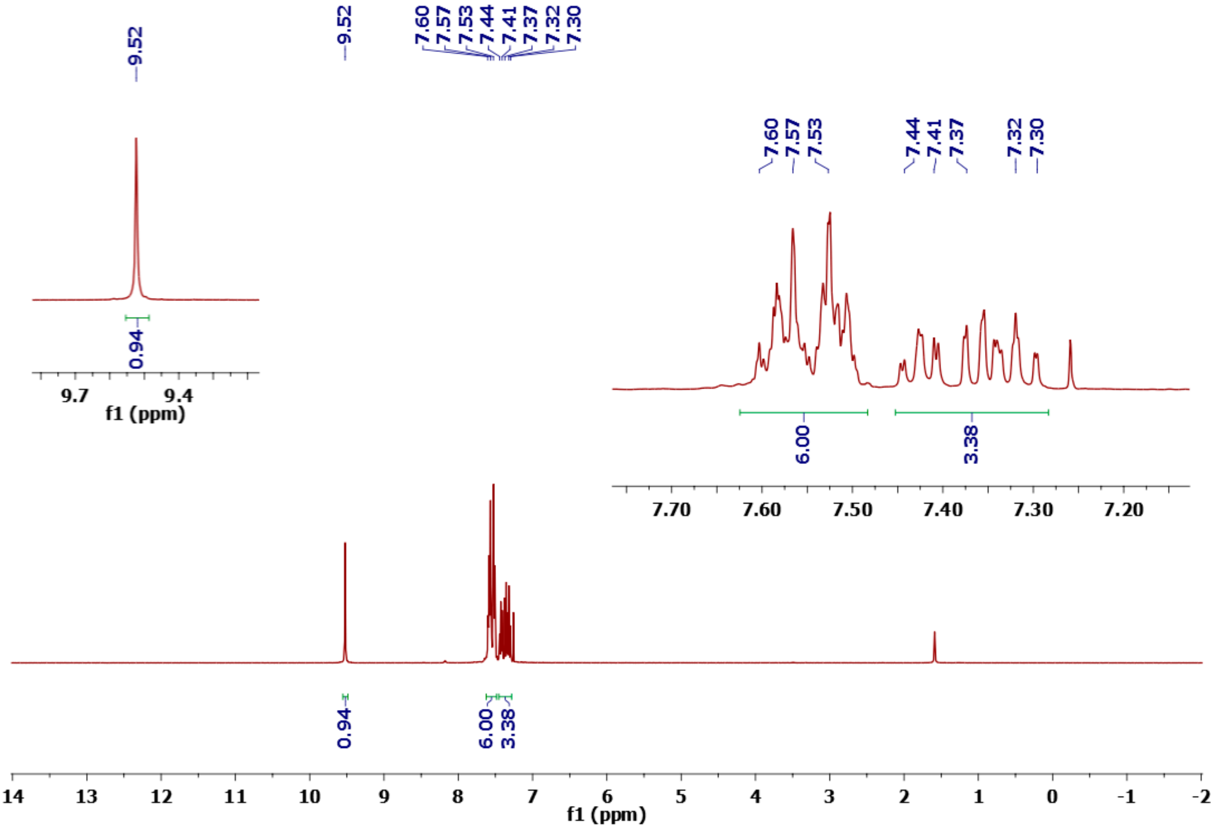

^{13}C NMR ($CDCl_3$) spectrum of 3-(2-fluorophenyl)-5-imino-1-phenylimidazolidine-2,4-dithione

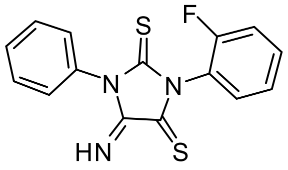

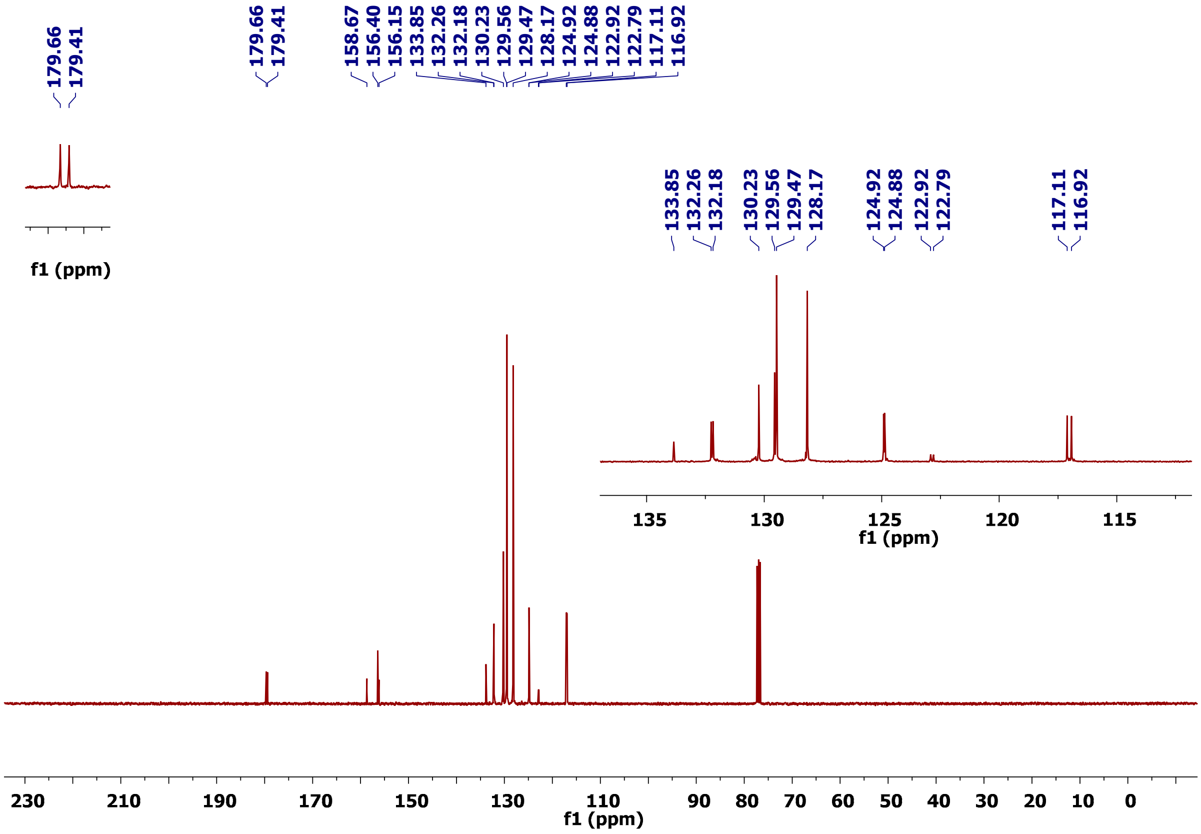

^{13}C-CRAPT NMR ($CDCl_3$) spectrum of 3-(2-fluorophenyl)-5-imino-1-phenylimidazolidine-2,4-dithione

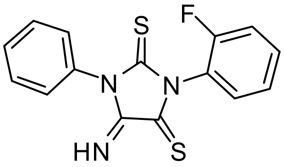

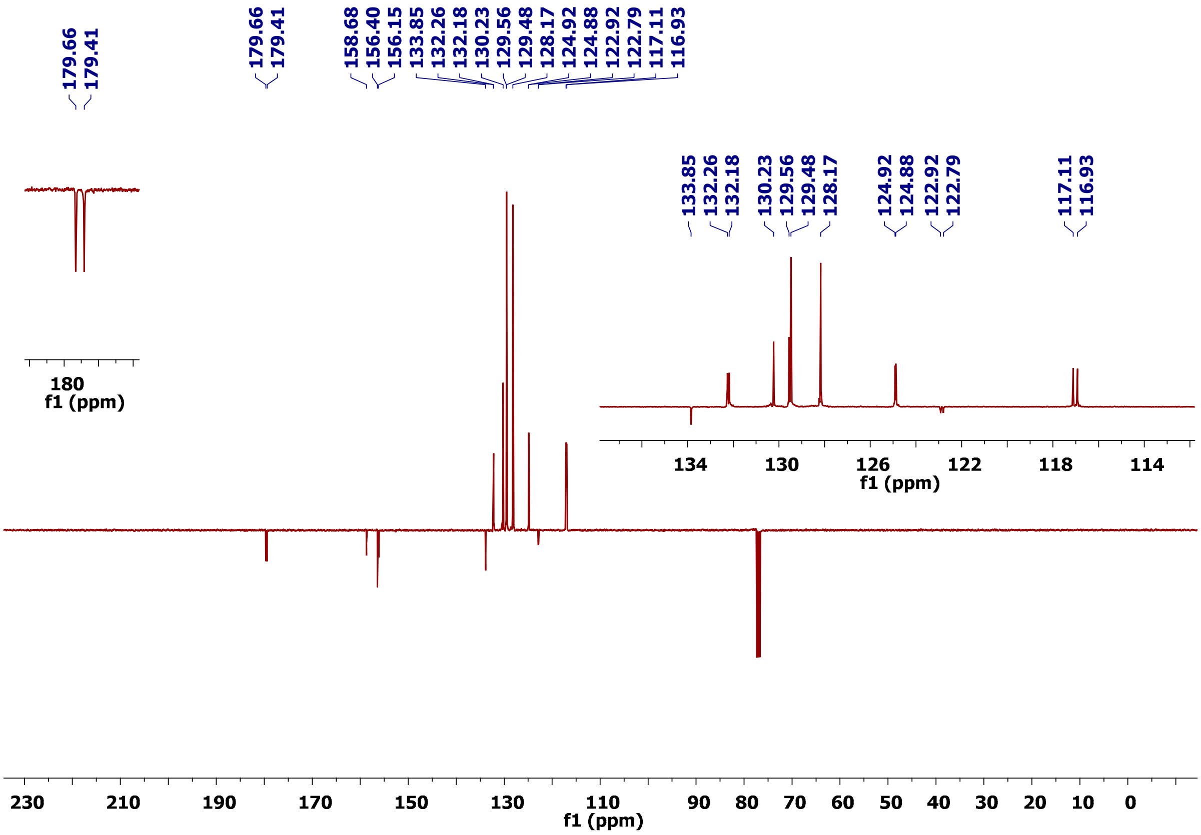

^{1}H-^{1}H-gCOSYAD NMR ($CDCl_3$) spectrum of 3-(2-fluorophenyl)-5-imino-1-phenylimidazolidine-2,4-dithione

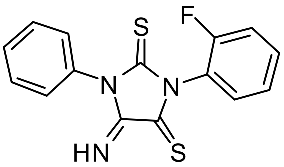

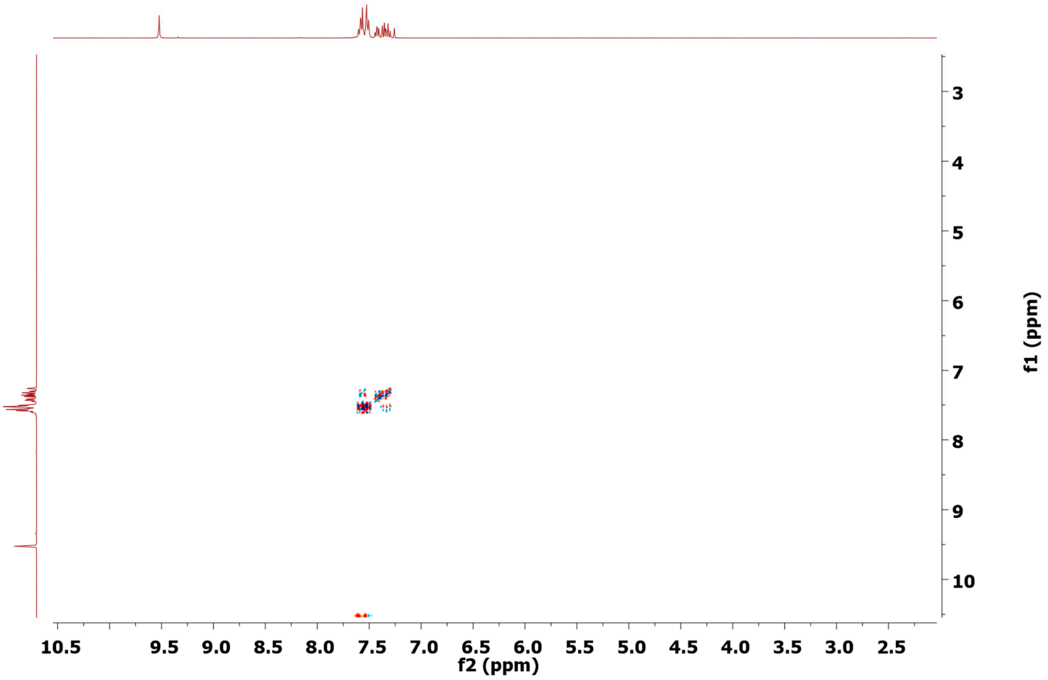

^{1}H-^{13}C-gHSQCAD ($CDCl_3$) spectrum of 3-(2-fluorophenyl)-5-imino-1-phenylimidazolidine-2,4-dithione

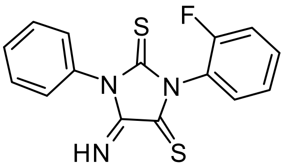

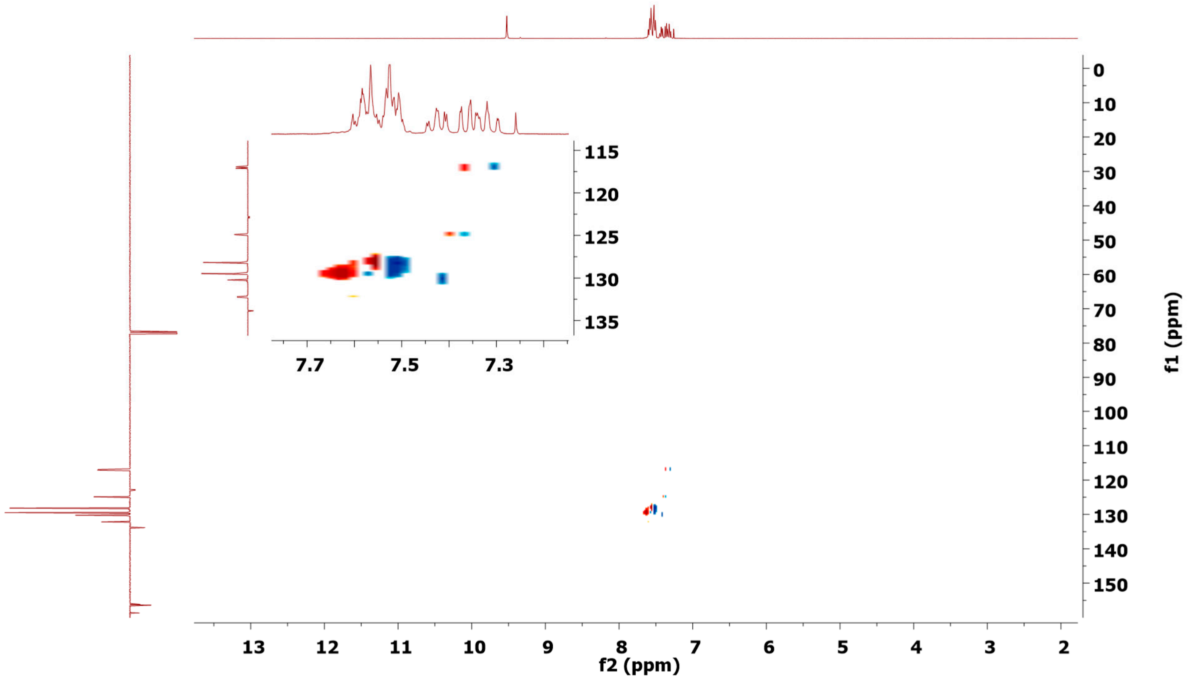

^{1}H-^{13}C-gHMBC ($CDCl_3$) spectrum of 3-(2-fluorophenyl)-5-imino-1-phenylimidazolidine-2,4-dithione

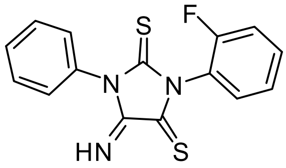

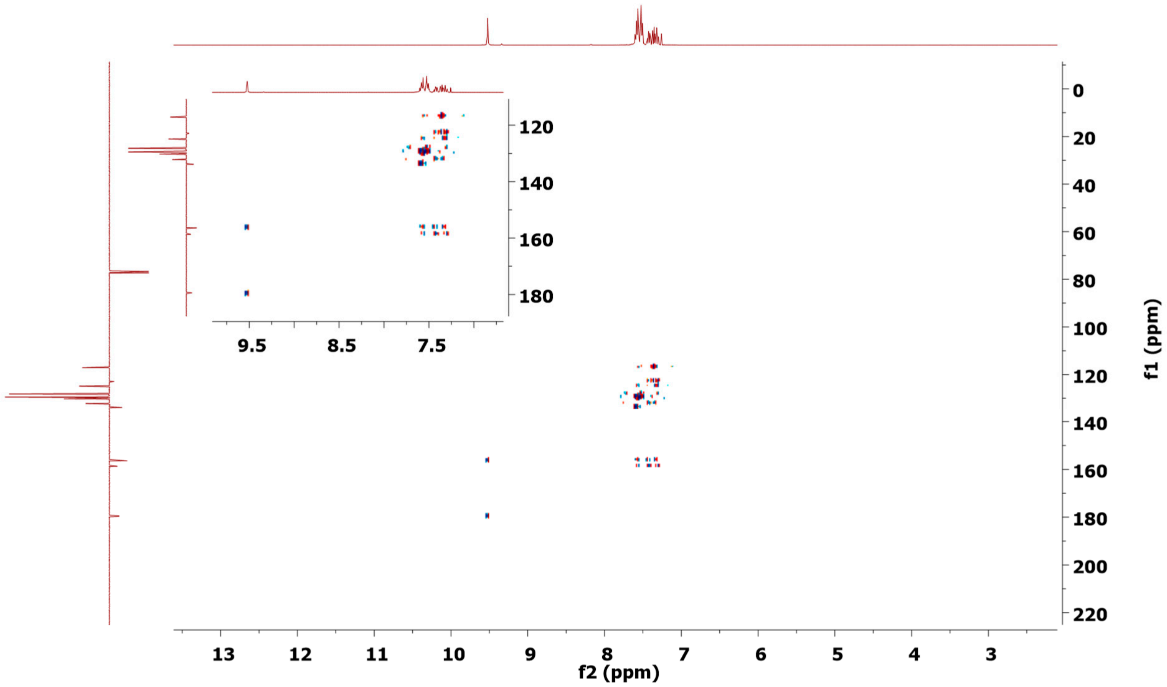

^{1}H NMR ($CDCl_3$) spectrum of 3-(2-fluorophenyl)-5-imino-1-(4-(trifluoromethyl)phenyl)imidazolidine-2,4-dithione (18t)

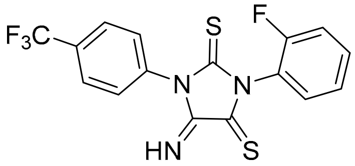

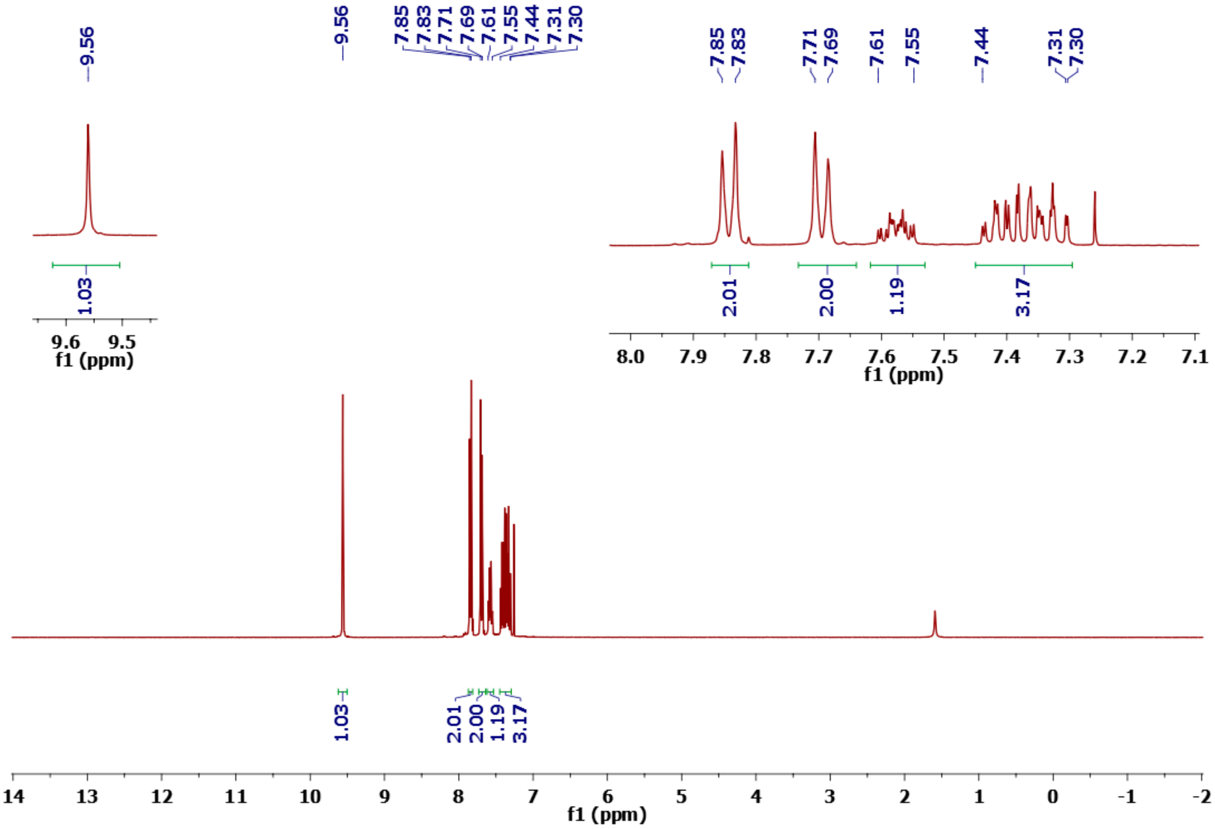

^{13}C NMR ($CDCl_3$) spectrum of 3-(2-fluorophenyl)-5-imino-1-(4-(trifluoromethyl)phenyl)imidazolidine-2,4-dithione

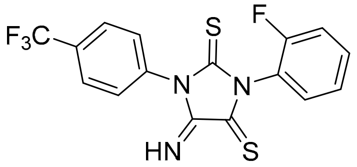

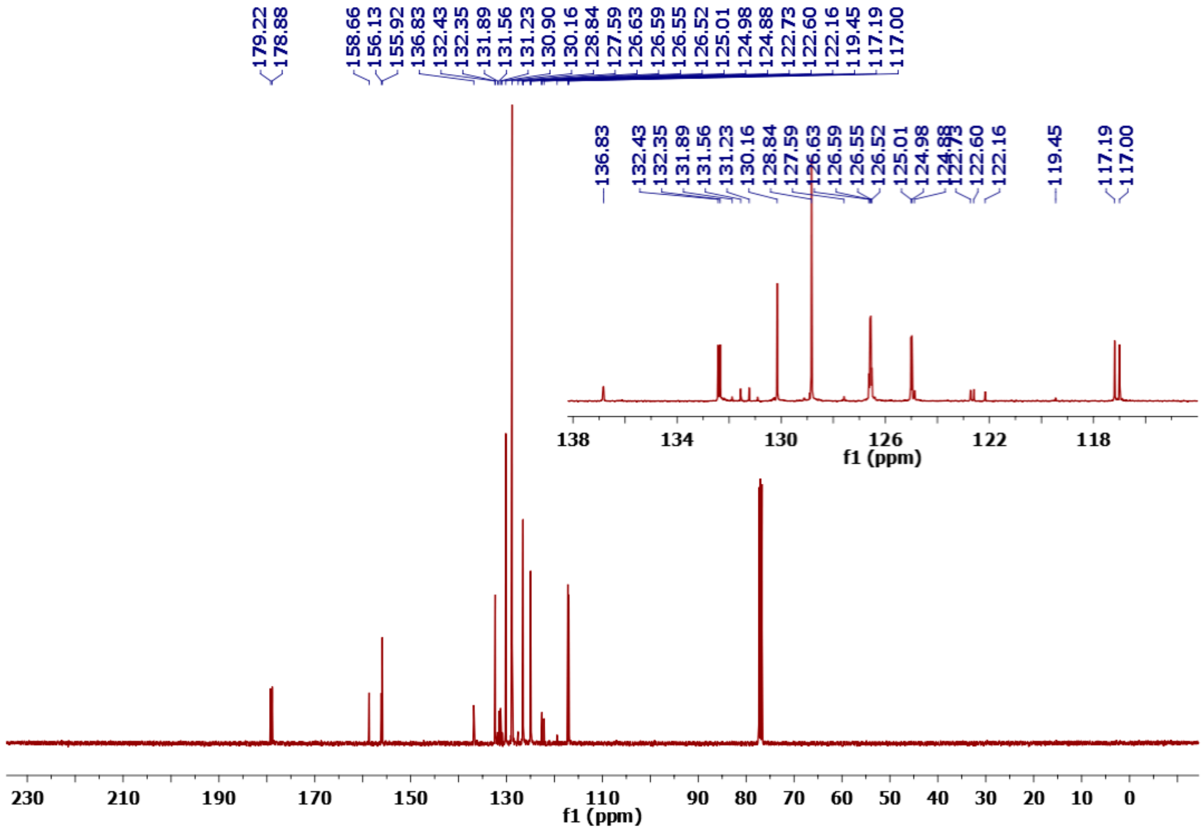

^{13}C-CRAPT NMR ($CDCl_3$) spectrum of 3-(2-fluorophenyl)-5-imino-1-(4-(trifluoromethyl)phenyl)imidazolidine-2,4-dithione

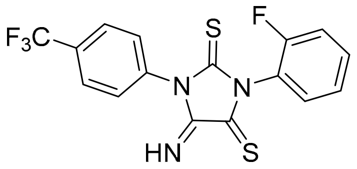

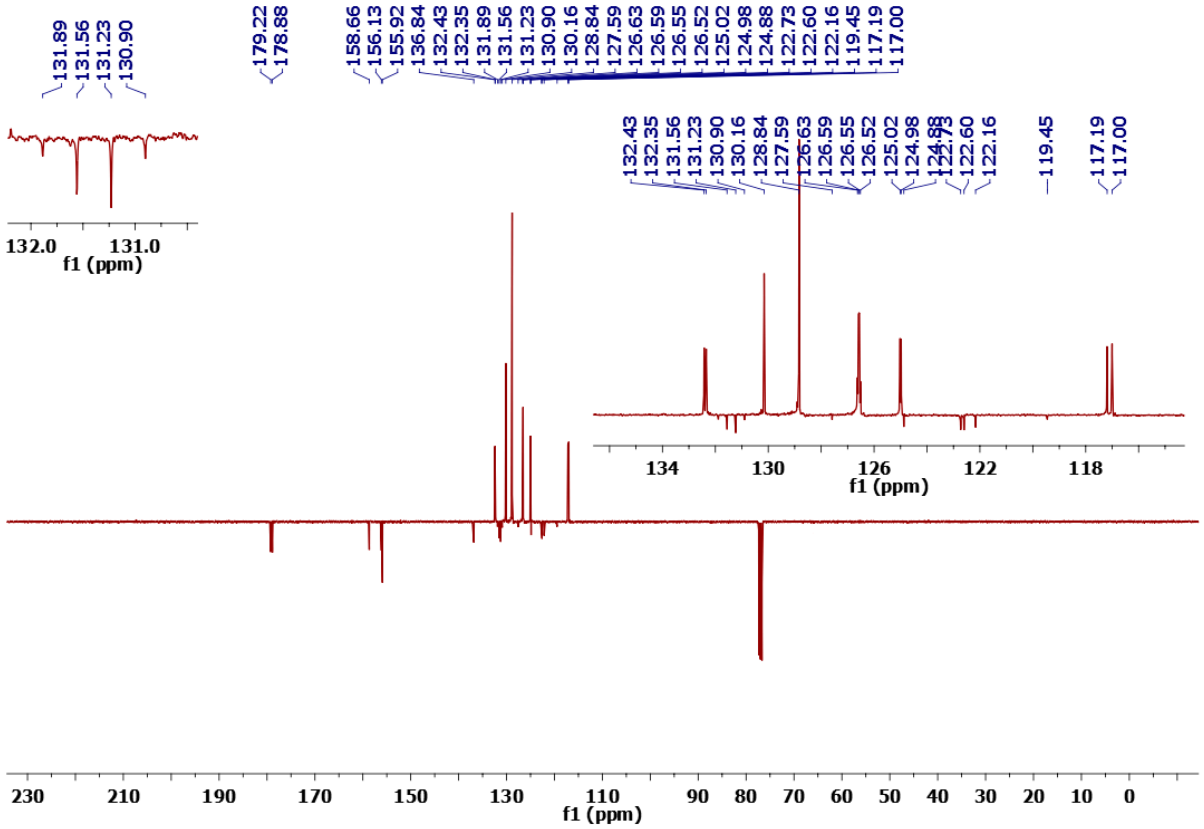

^{1}H-^{1}H-gCOSYAD NMR ($CDCl_3$) spectrum of 3-(2-fluorophenyl)-5-imino-1-(4-(trifluoromethyl)phenyl)imidazolidine-2,4-dithione

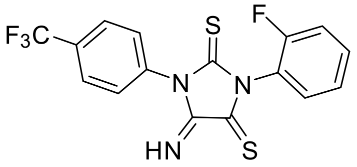

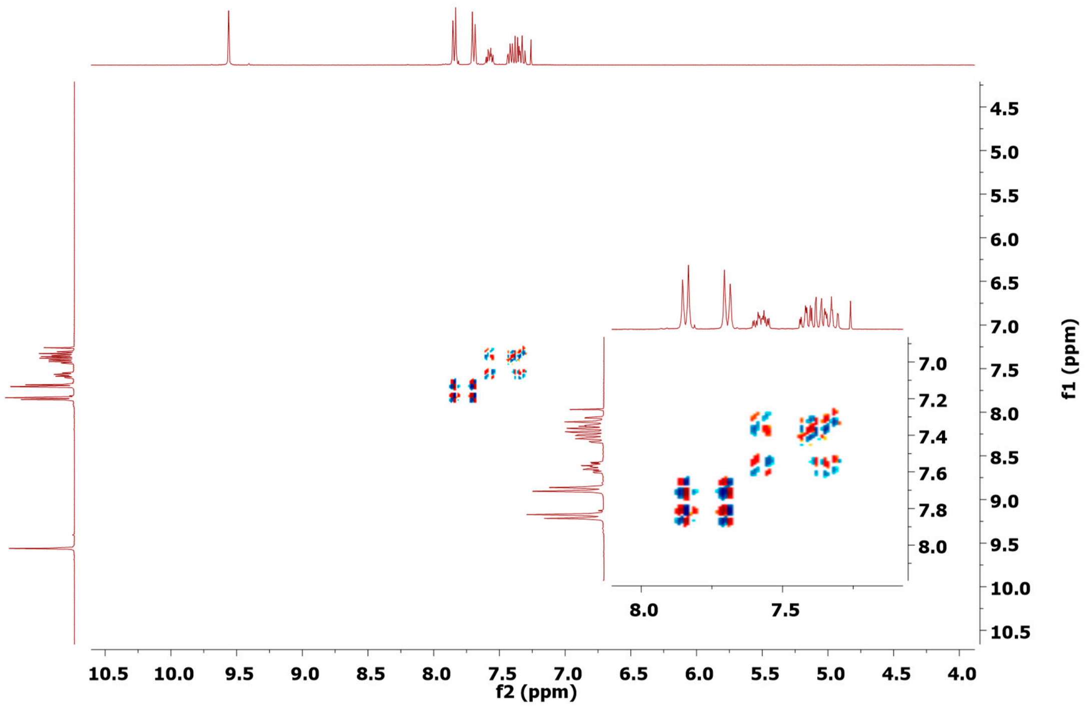

^{1}H-^{13}C-gHSQCAD NMR ($CDCl_3$) spectrum of 3-(2-fluorophenyl)-5-imino-1-(4-(trifluoromethyl)phenyl)imidazolidine-2,4-dithione

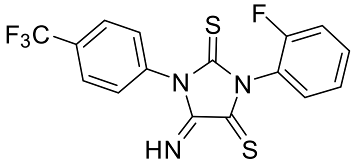

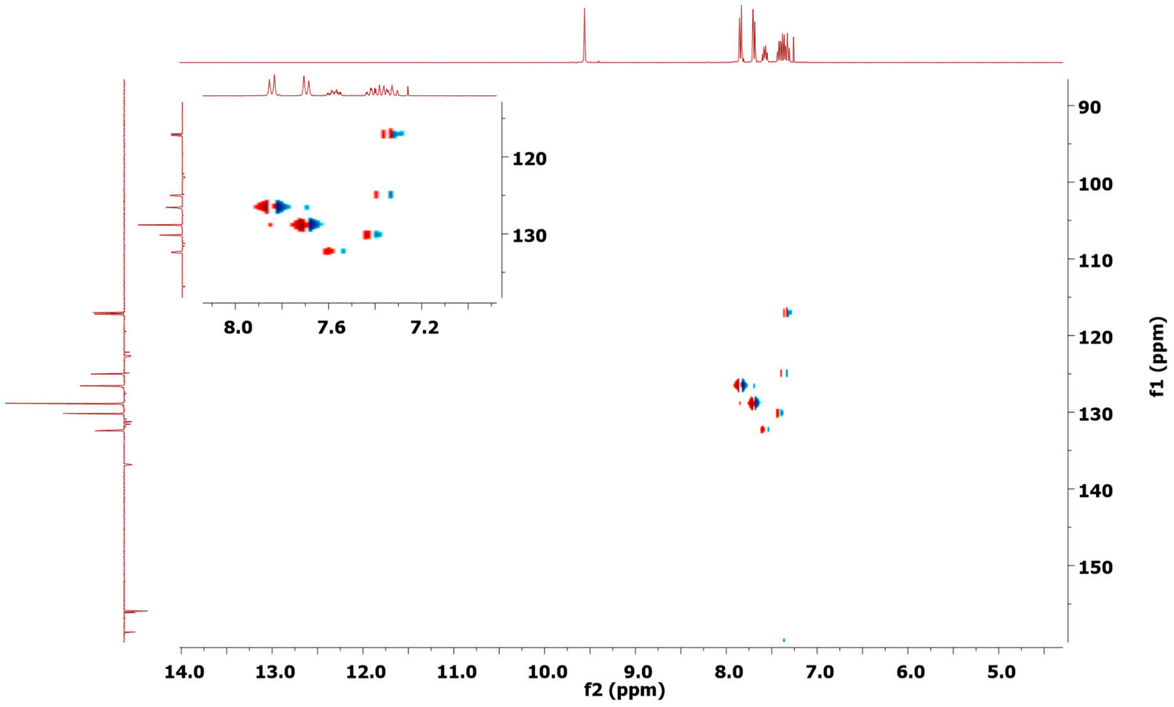

^{1}H-^{13}C-gHMBC NMR ($CDCl_3$) spectrum of 3-(2-fluorophenyl)-5-imino-1-(4-(trifluoromethyl)phenyl)imidazolidine-2,4-dithione

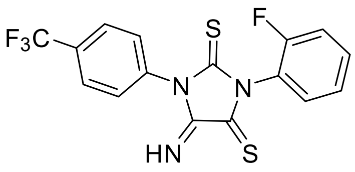

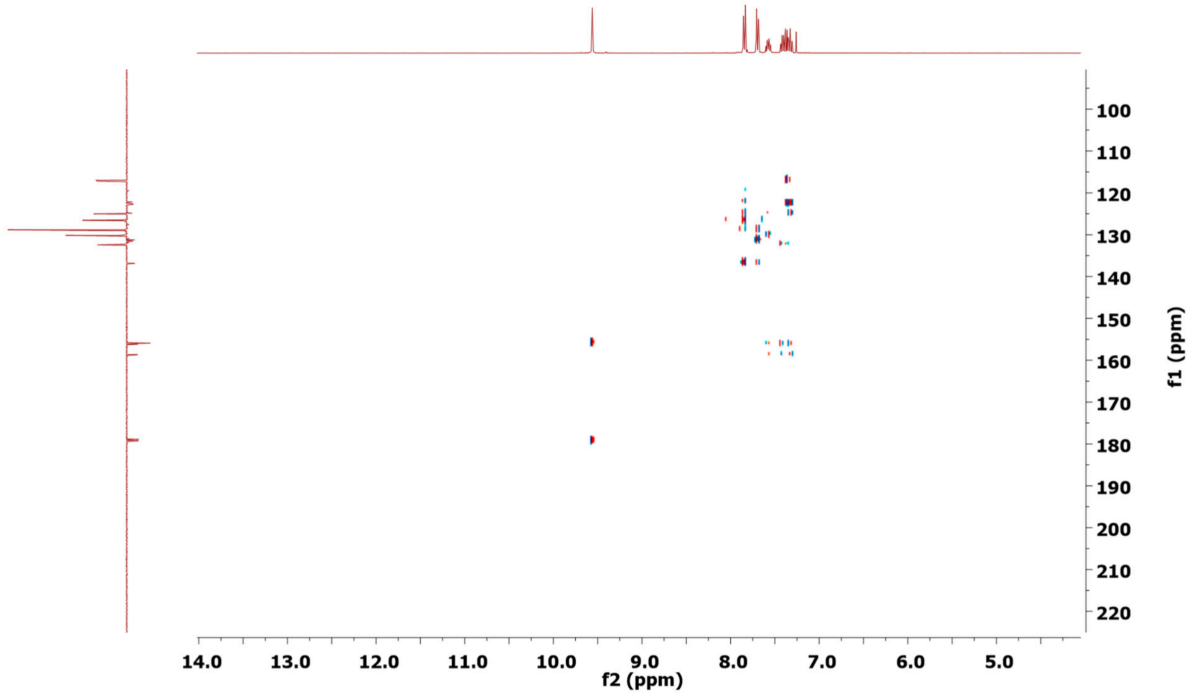

^{1}H NMR ($CDCl_3$) spectrum of 3-(2-fluorophenyl)-5-imino-1-(p-tolyl)imidazolidine-2,4-dithione (18u)

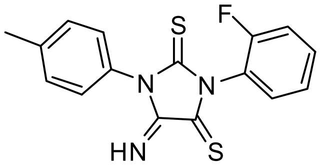

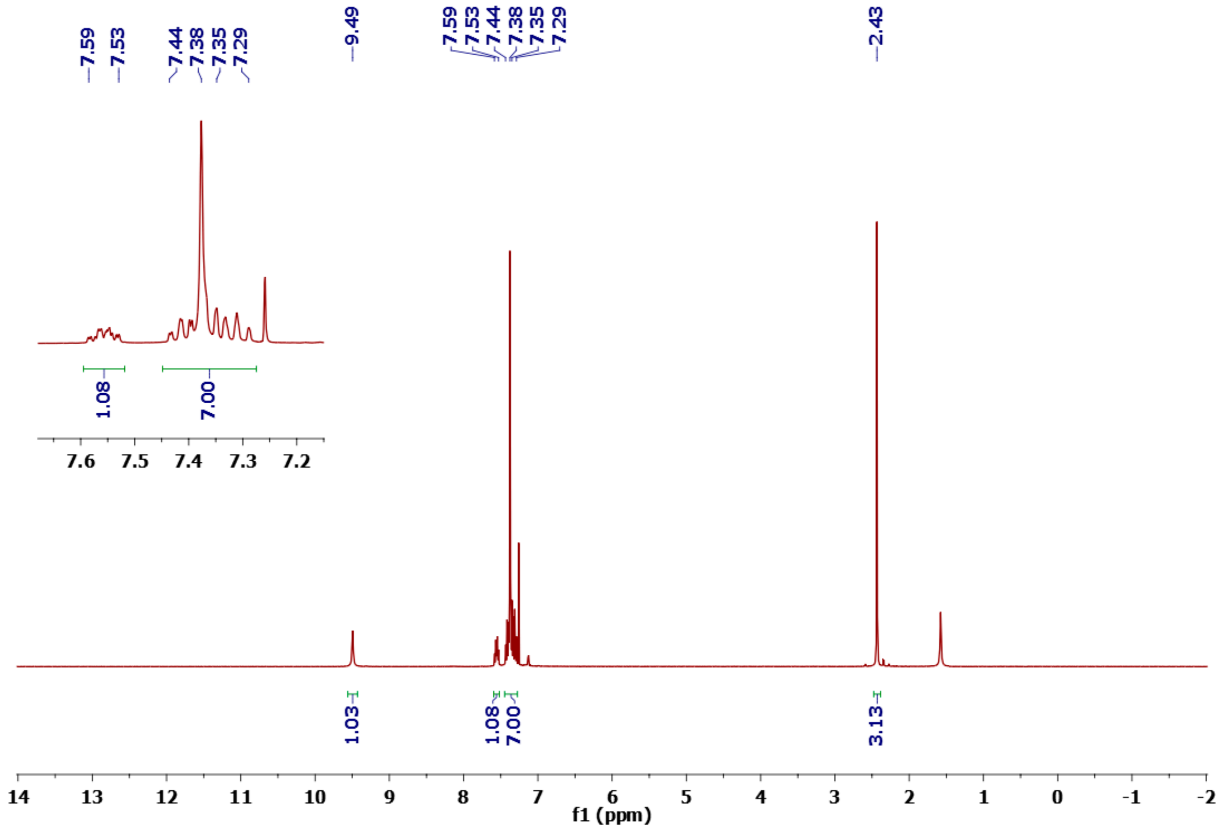

^{13}C NMR ($CDCl_3$) spectrum of 3-(2-fluorophenyl)-5-imino-1-(p-tolyl)imidazolidine-2,4-dithione

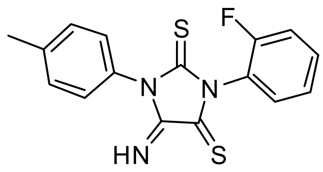

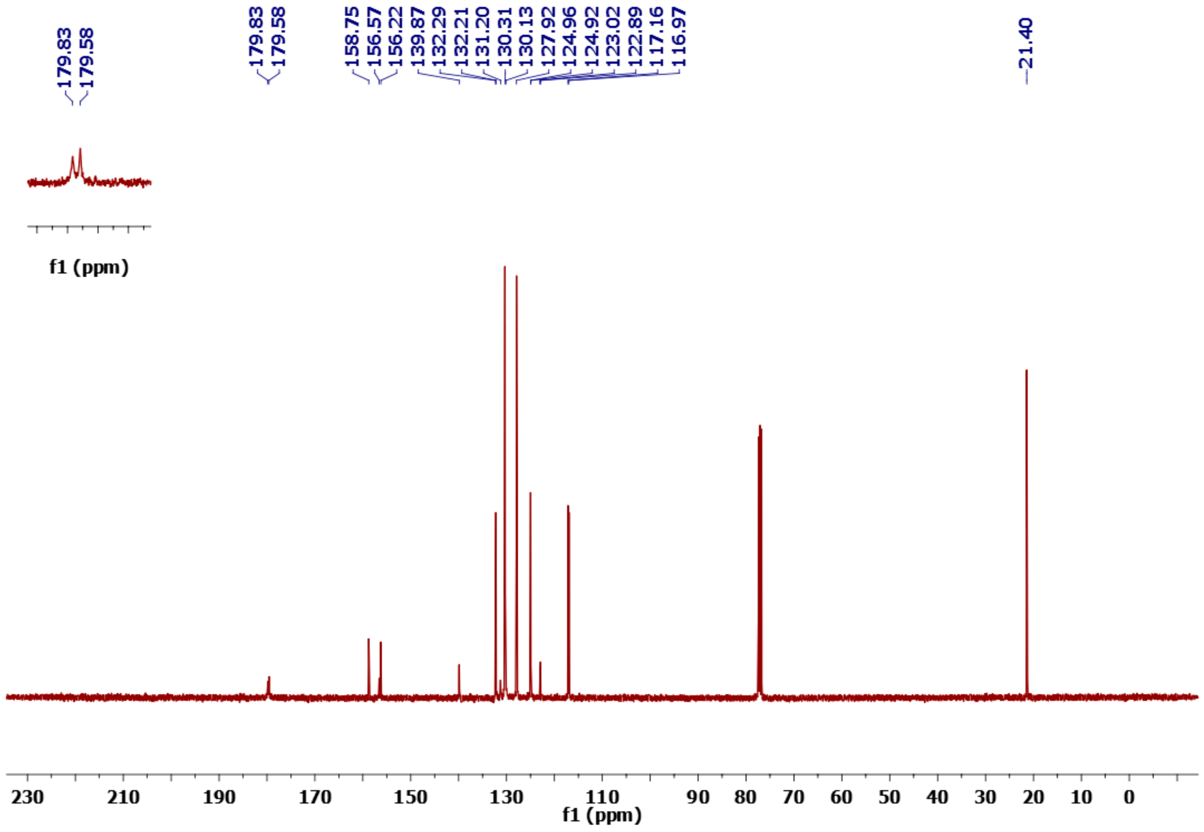

^{13}C-CRAPT NMR ($CDCl_3$) spectrum of 3-(2-fluorophenyl)-5-imino-1-(p-tolyl)imidazolidine-2,4-dithione

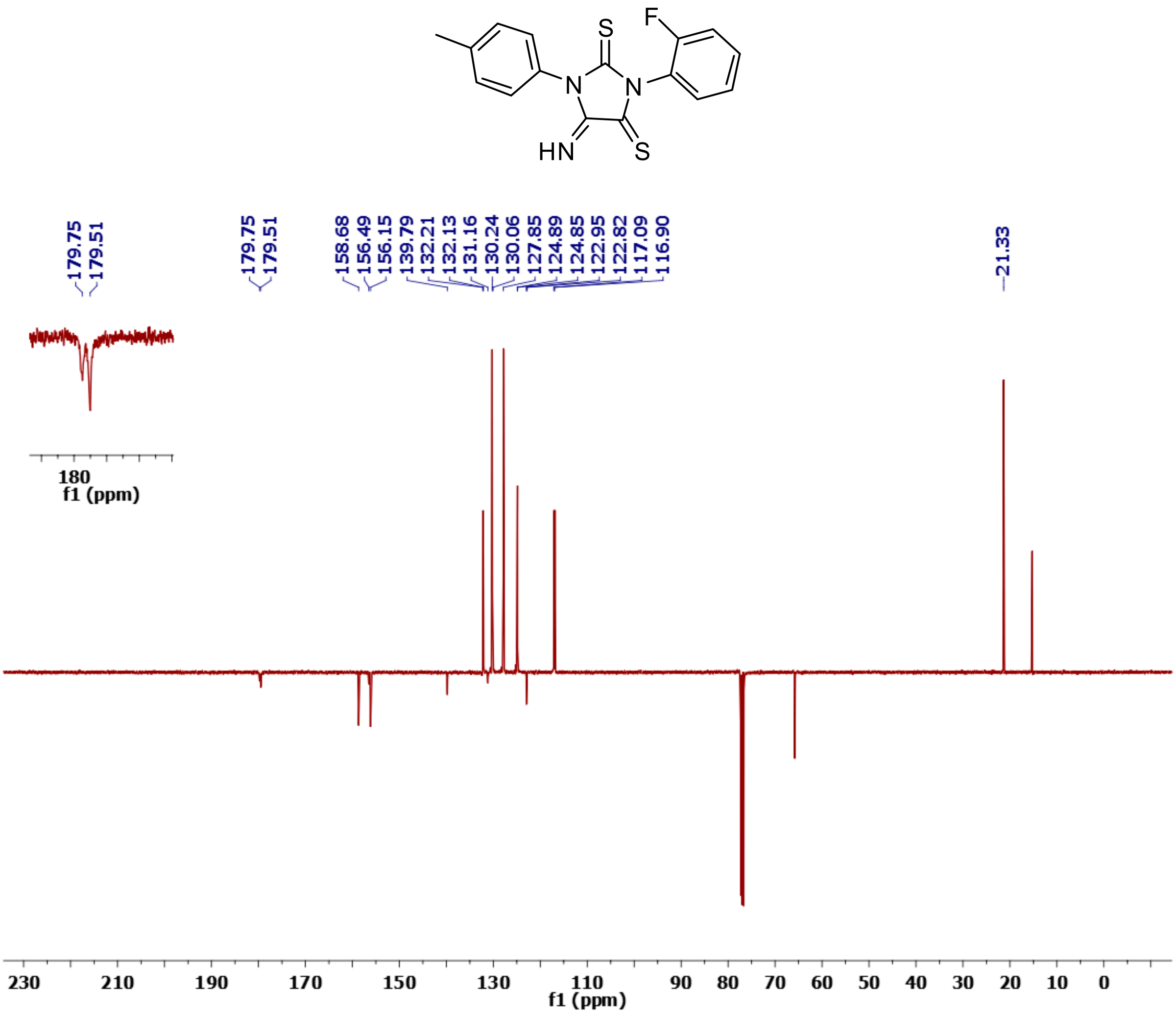

^{1}H-^{1}H-gCOSYAD NMR ($CDCl_3$) spectrum of 3-(2-fluorophenyl)-5-imino-1-(p-tolyl)imidazolidine-2,4-dithione

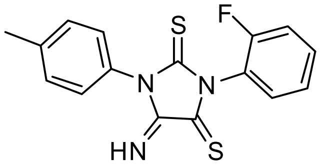

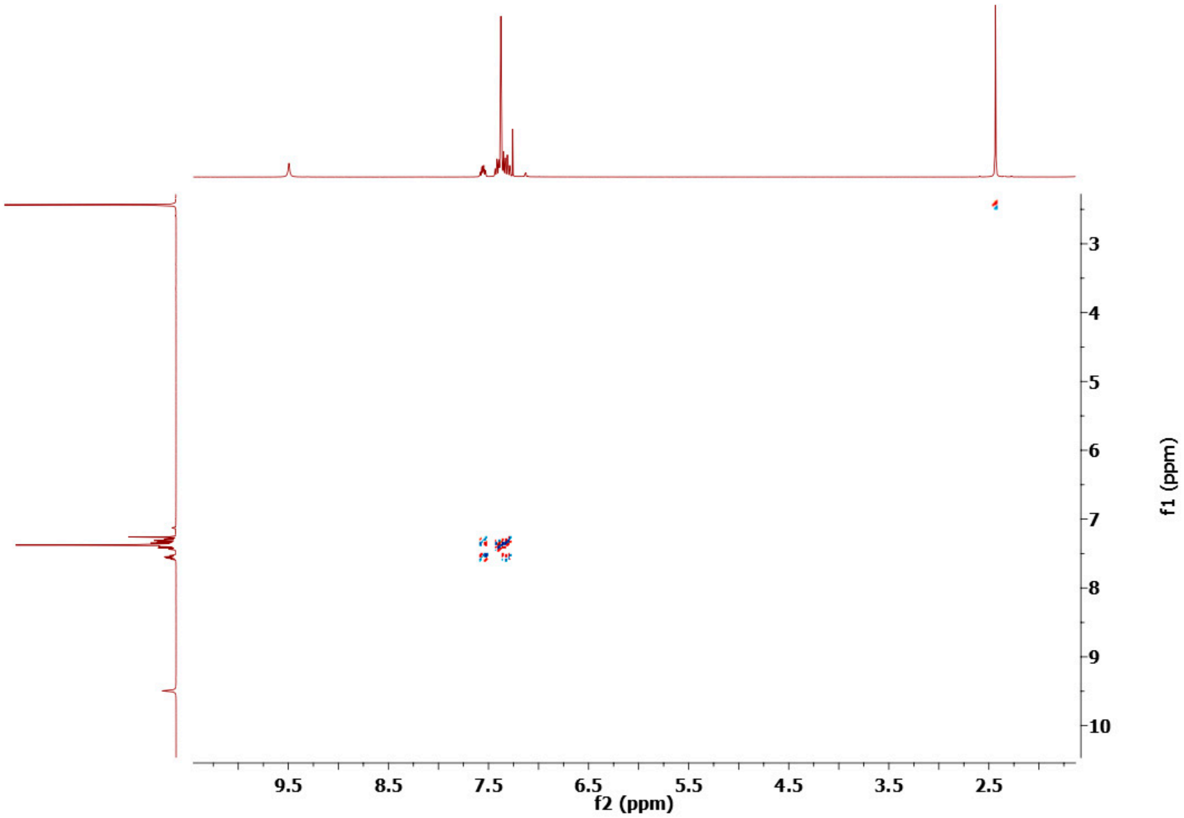

^{1}H-^{13}C-gHSQCAD ($CDCl_3$) spectrum of 3-(2-fluorophenyl)-5-imino-1-(p-tolyl)imidazolidine-2,4-dithione

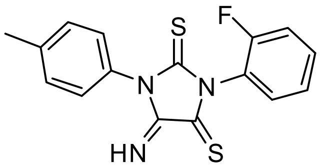

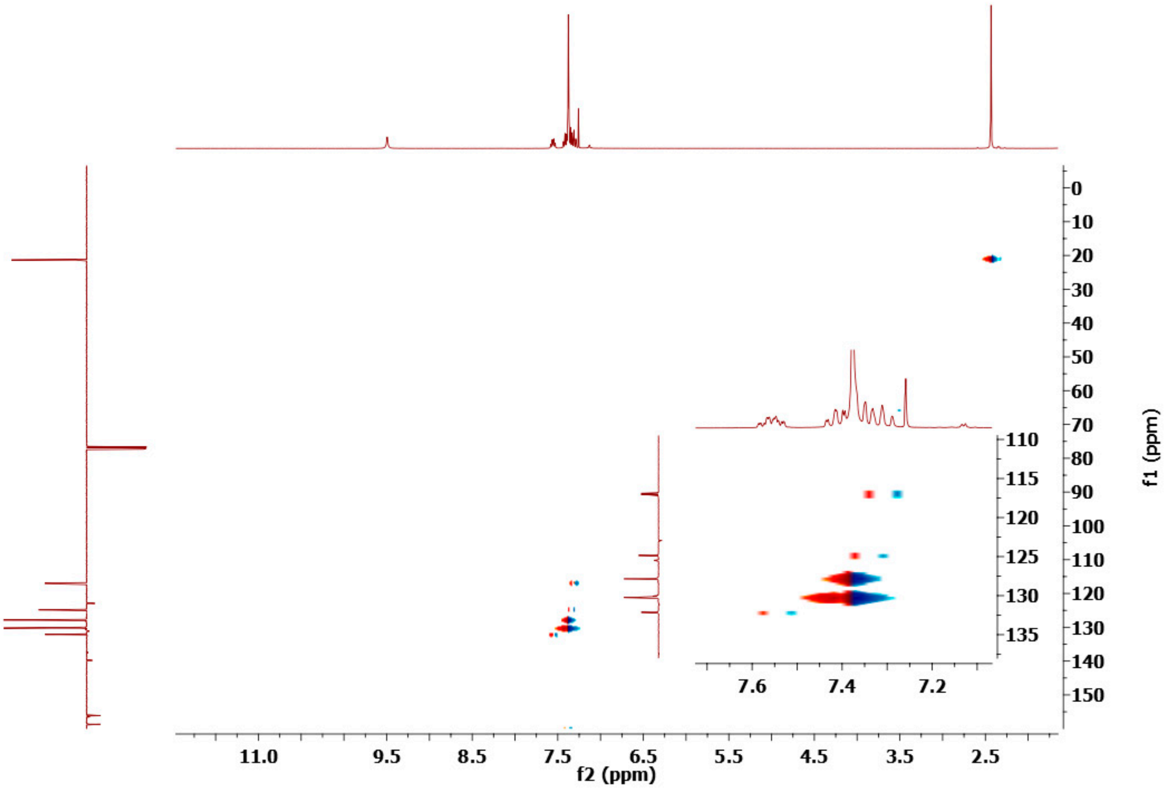

^{1}H-^{13}C-gHMBC ($CDCl_3$) spectrum of 3-(2-fluorophenyl)-5-imino-1-(p-tolyl)imidazolidine-2,4-dithione

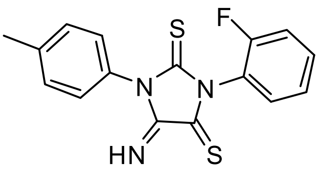

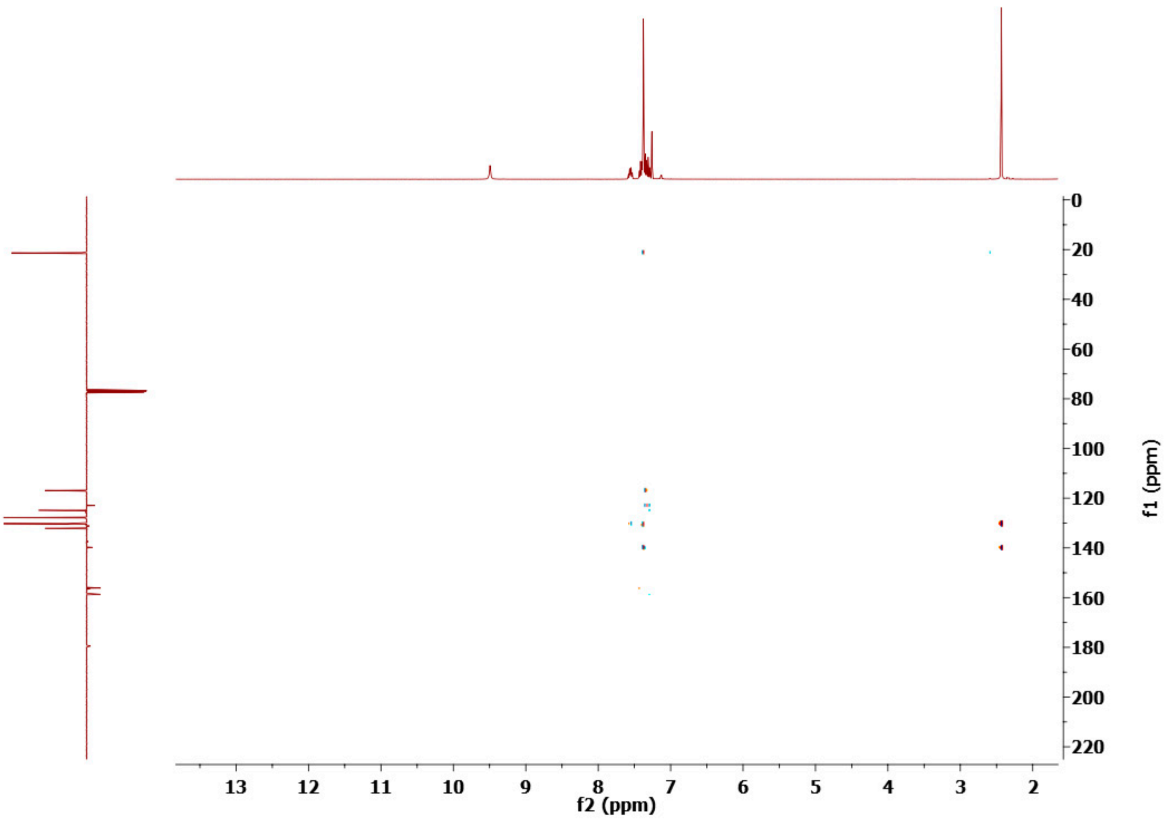

^{1}H NMR ($CDCl_3$) spectrum of 1-(4-chlorophenyl)-3-(2-fluorophenyl)-5-iminoimidazolidine-2,4-dithione (18v)

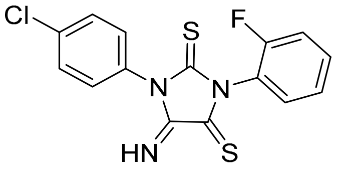

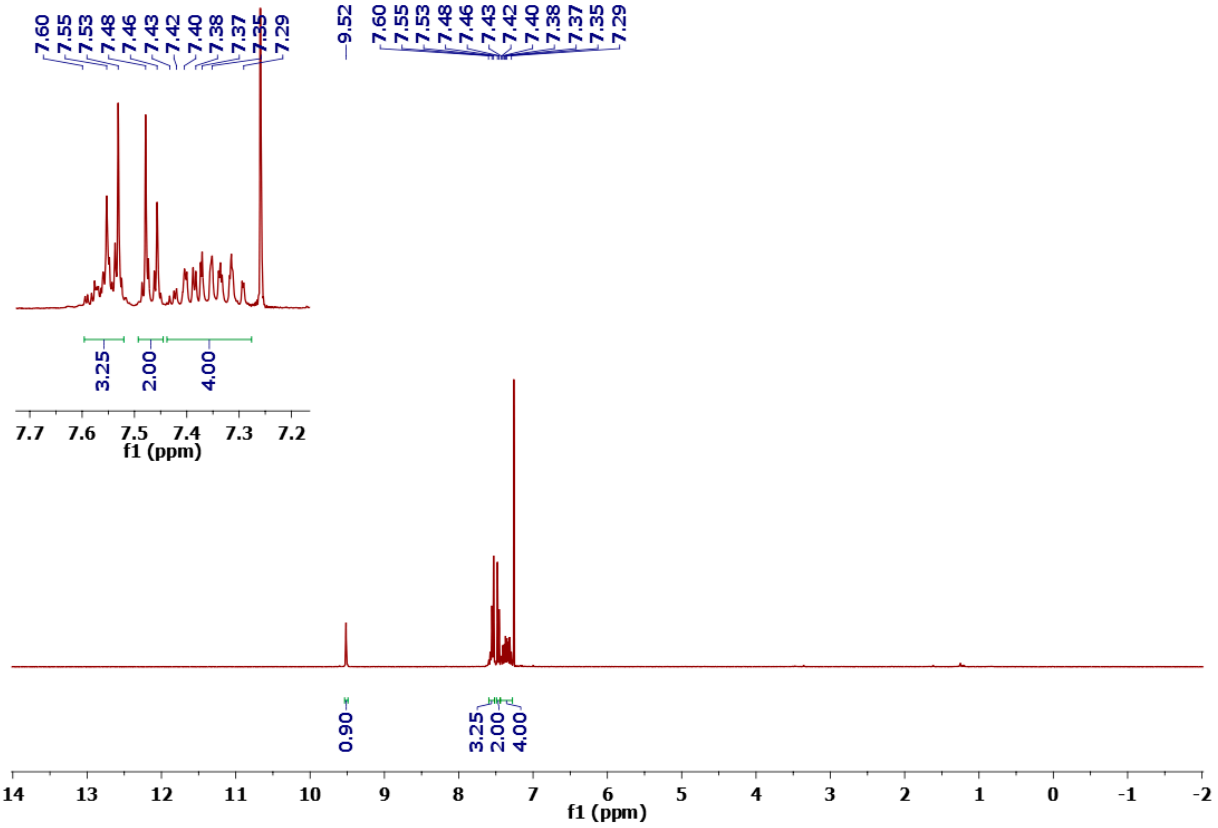

^{13}C NMR (CDCl₃) spectrum of 1-(4-chlorophenyl)-3-(2-fluorophenyl)-5-iminoimidazolidine-2,4-dithione

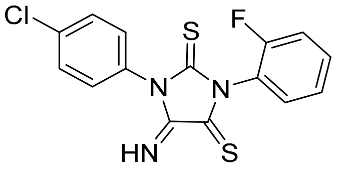

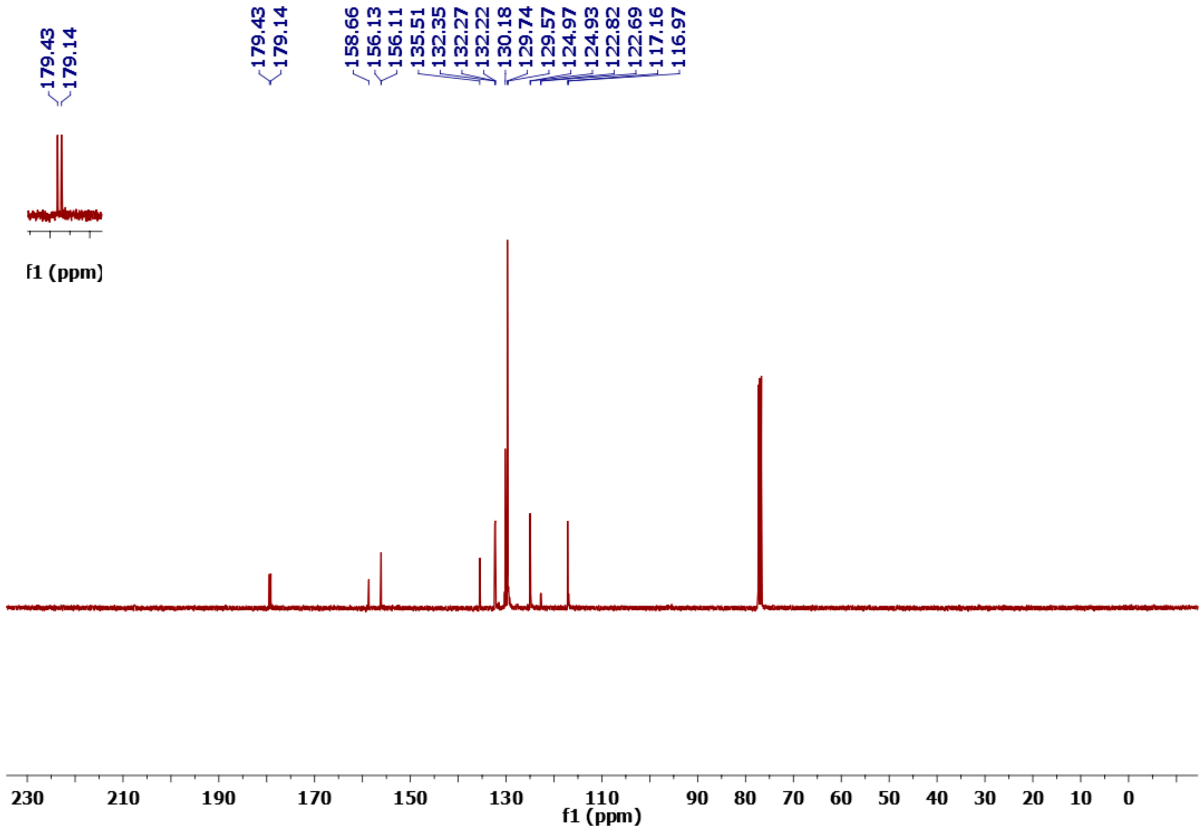

^{13}C-CRAPT NMR ($CDCl_3$) spectrum of 1-(4-chlorophenyl)-3-(2-fluorophenyl)-5-iminoimidazolidine-2,4-dithione

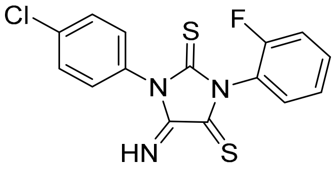

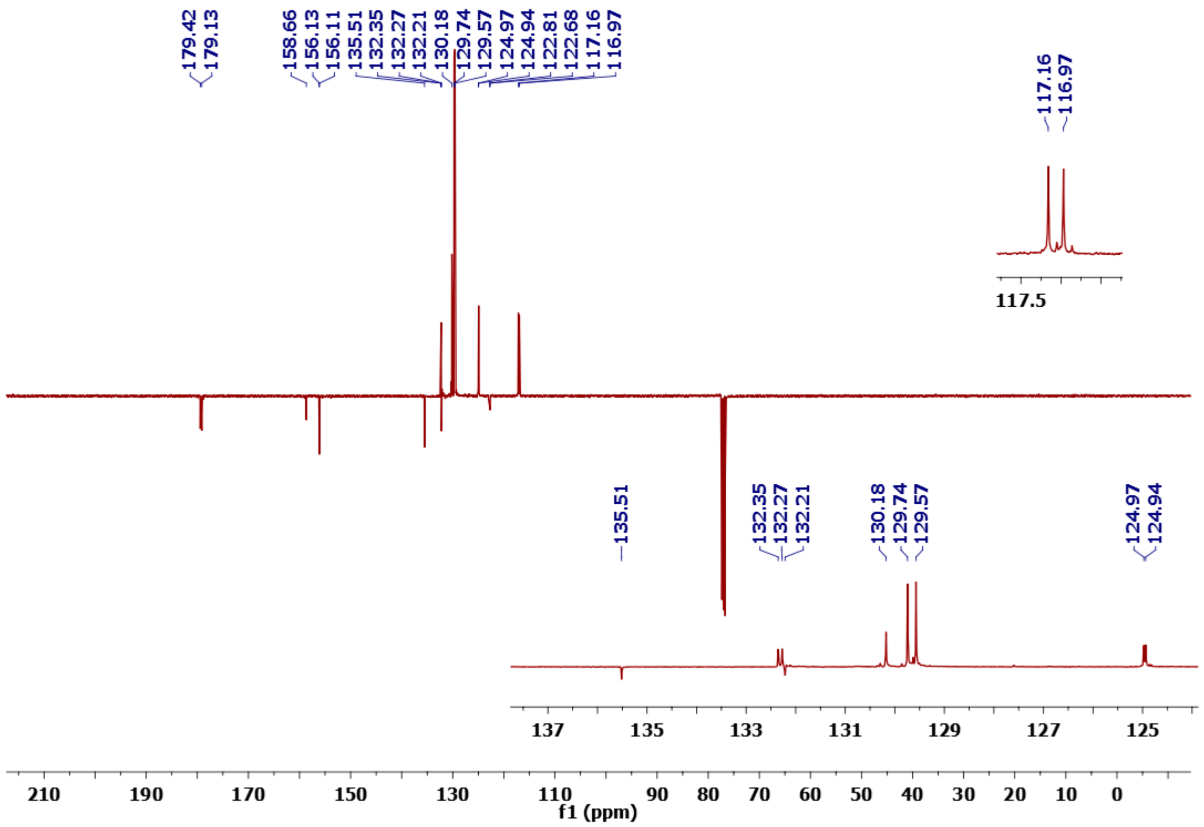

^{1}H-^{1}H-gCOSYAD NMR ($CDCl_3$) spectrum of 1-(4-chlorophenyl)-3-(2-fluorophenyl)-5-iminoimidazolidine-2,4-dithione

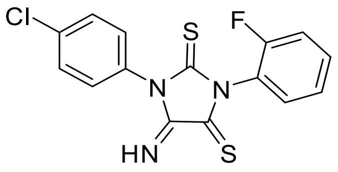

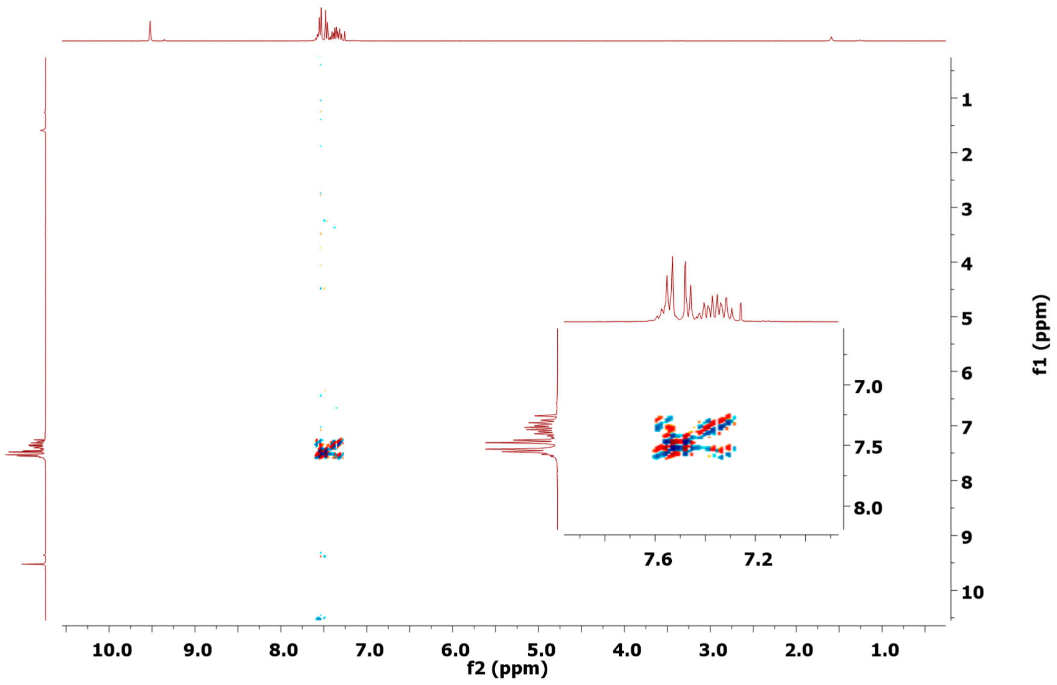

^{1}H-^{13}C-gHSQCAD NMR ($CDCl_3$) spectrum of 1-(4-chlorophenyl)-3-(2-fluorophenyl)-5-iminoimidazolidine-2,4-dithione

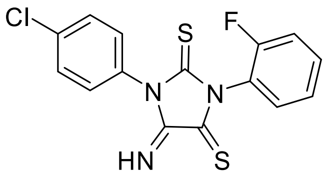

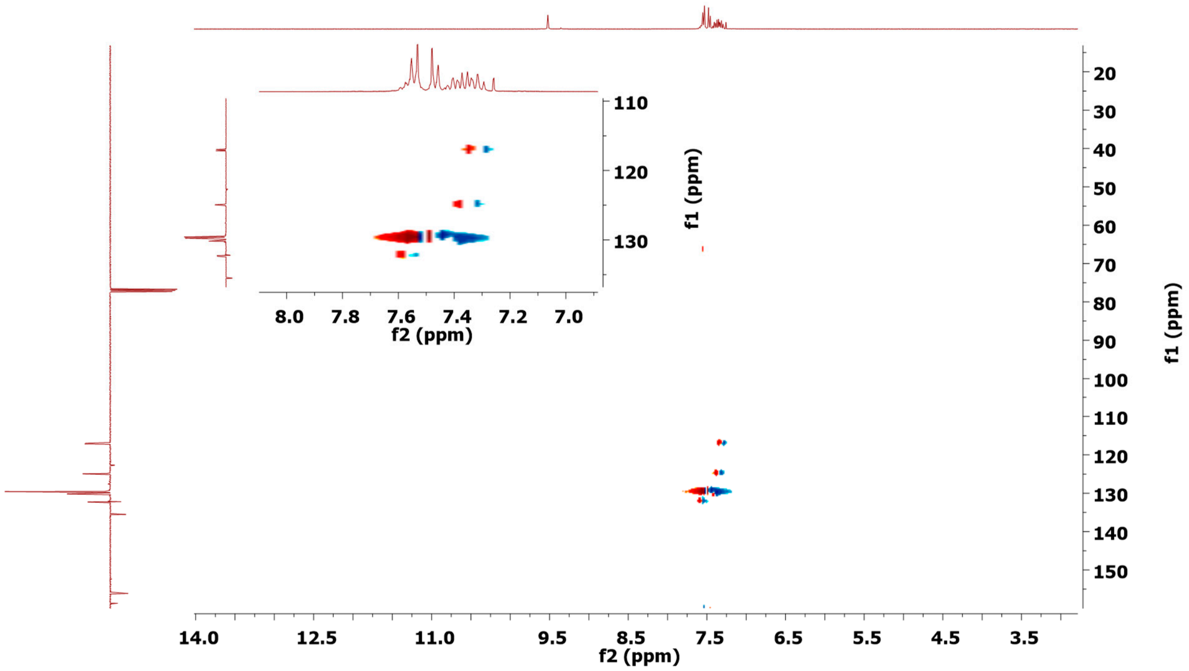

^{1}H-^{13}C-gHMBC NMR ($CDCl_3$) spectrum of 1-(4-chlorophenyl)-3-(2-fluorophenyl)-5-iminoimidazolidine-2,4-dithione

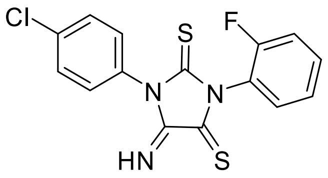

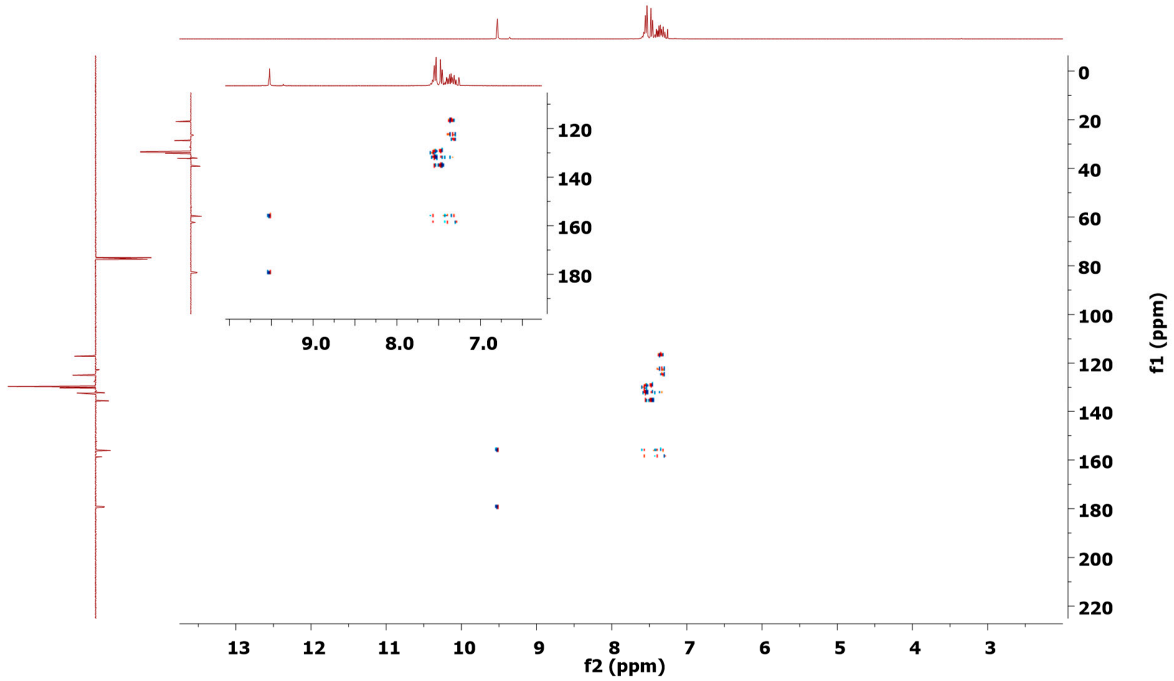

^{1}H NMR ($CDCl_3$) spectrum of 3-(2-fluorophenyl)-1-(4-fluorophenyl)-5-iminoimidazolidine-2,4-dithione (18w)

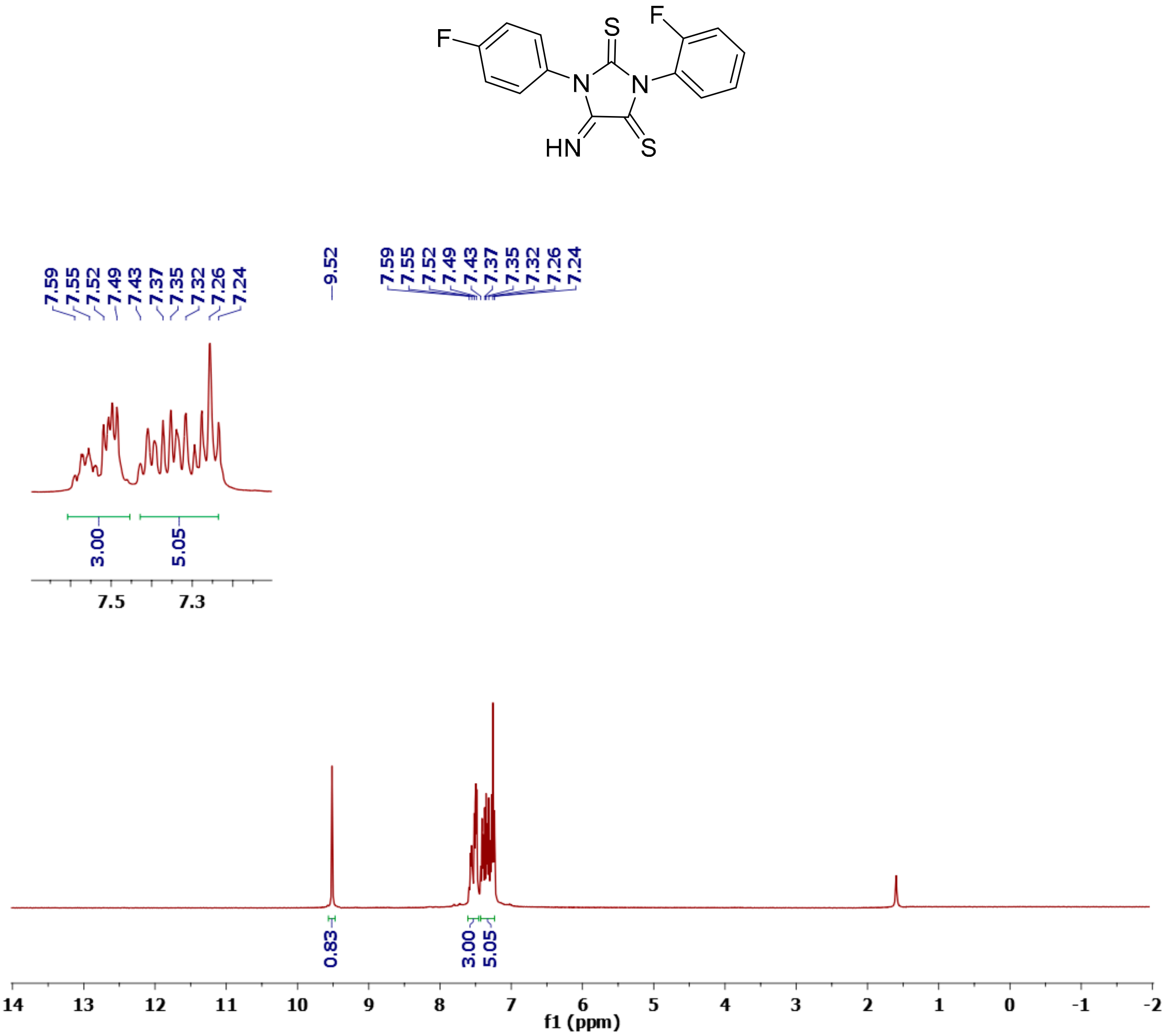

^{13}C NMR (CDCl$_3$) spectrum of 3-(2-fluorophenyl)-1-(4-fluorophenyl)-5-iminoimidazolidine-2,4-dithione

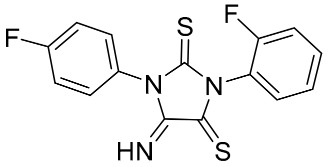

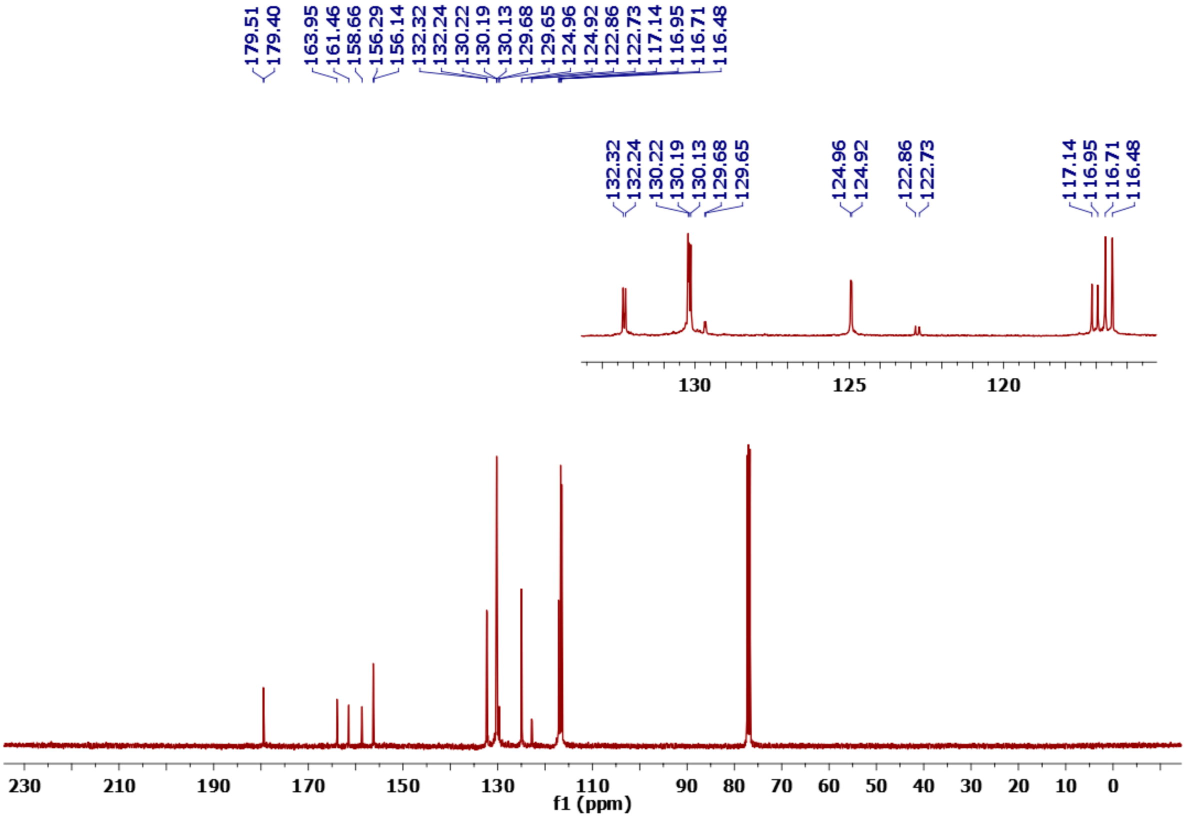

^{13}C CRAPT NMR ($CDCl_3$) spectrum of 3-(2-fluorophenyl)-1-(4-fluorophenyl)-5-iminoimidazolidine-2,4-dithione

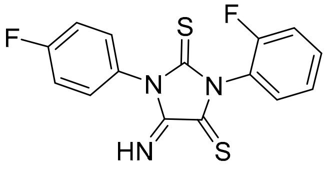

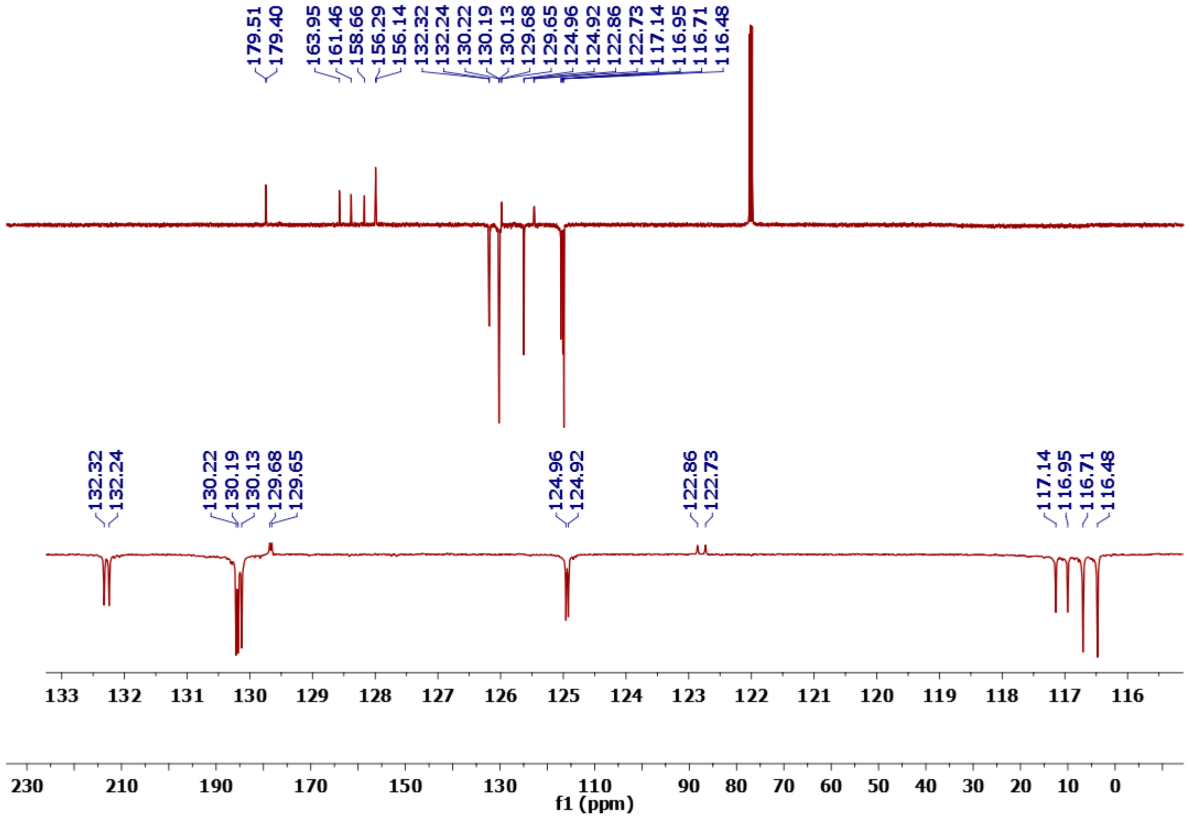

^{1}H-^{1}H-gDQCOSY NMR ($CDCl_3$) spectrum of 3-(2-fluorophenyl)-1-(4-fluorophenyl)-5-iminoimidazolidine-2,4-dithione

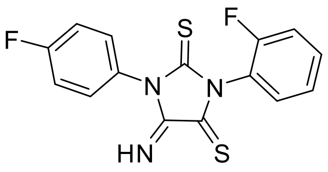

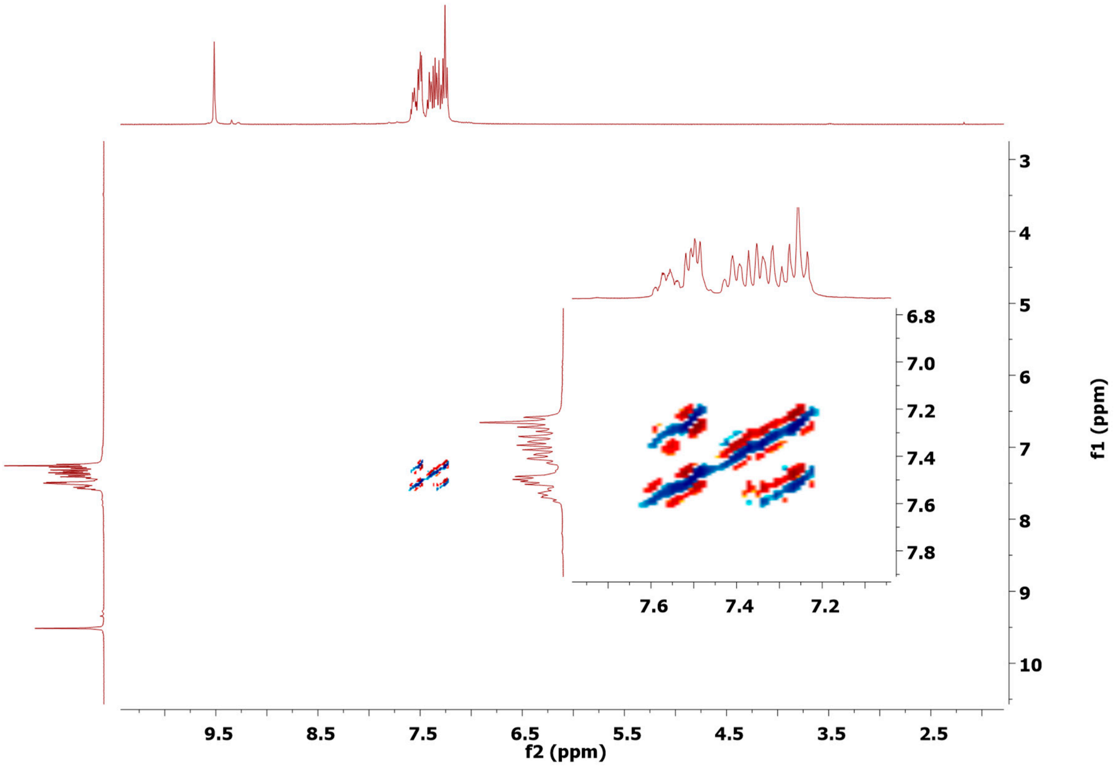

^{1}H-^{13}C-gHSQCAD NMR ($CDCl_3$) spectrum of 3-(2-fluorophenyl)-1-(4-fluorophenyl)-5-iminoimidazolidine-2,4-dithione

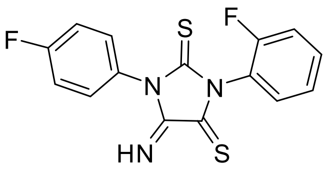

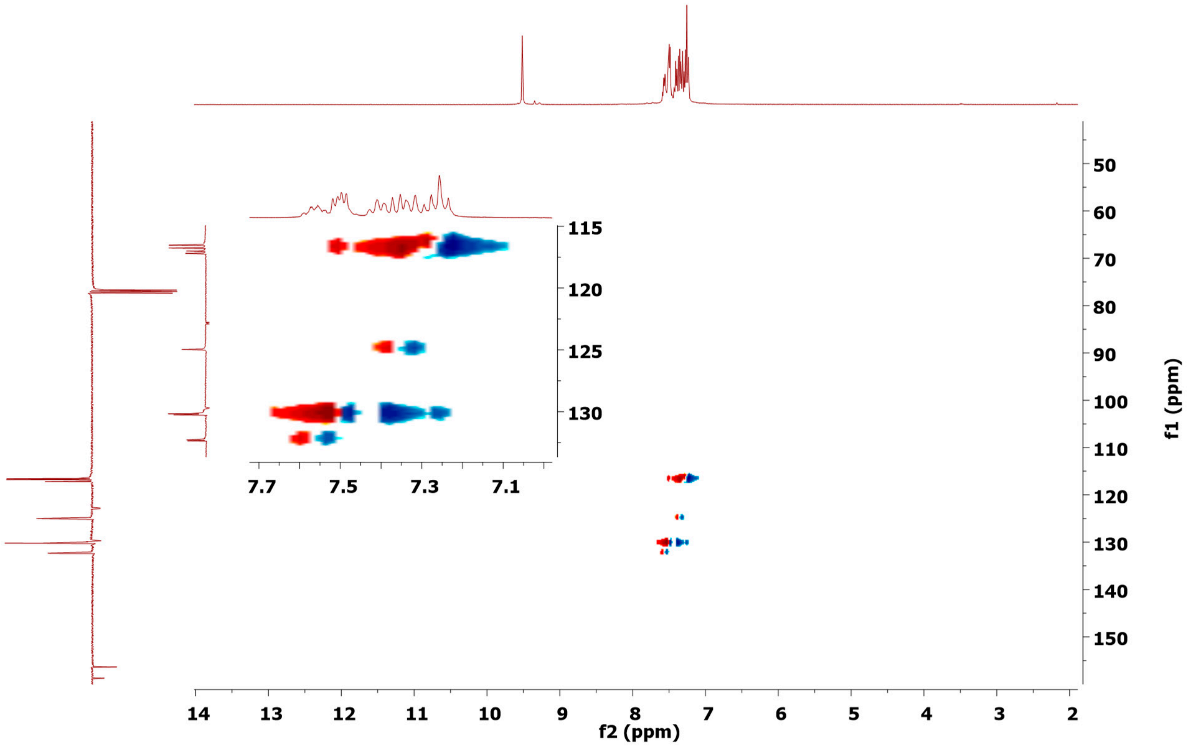

^{1}H-NMR ($CDCl_3$) spectrum of 3-(2-fluorophenyl)-5-imino-1-(4-nitrophenyl)imidazolidine-2,4-dithione (18x)

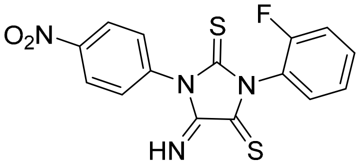

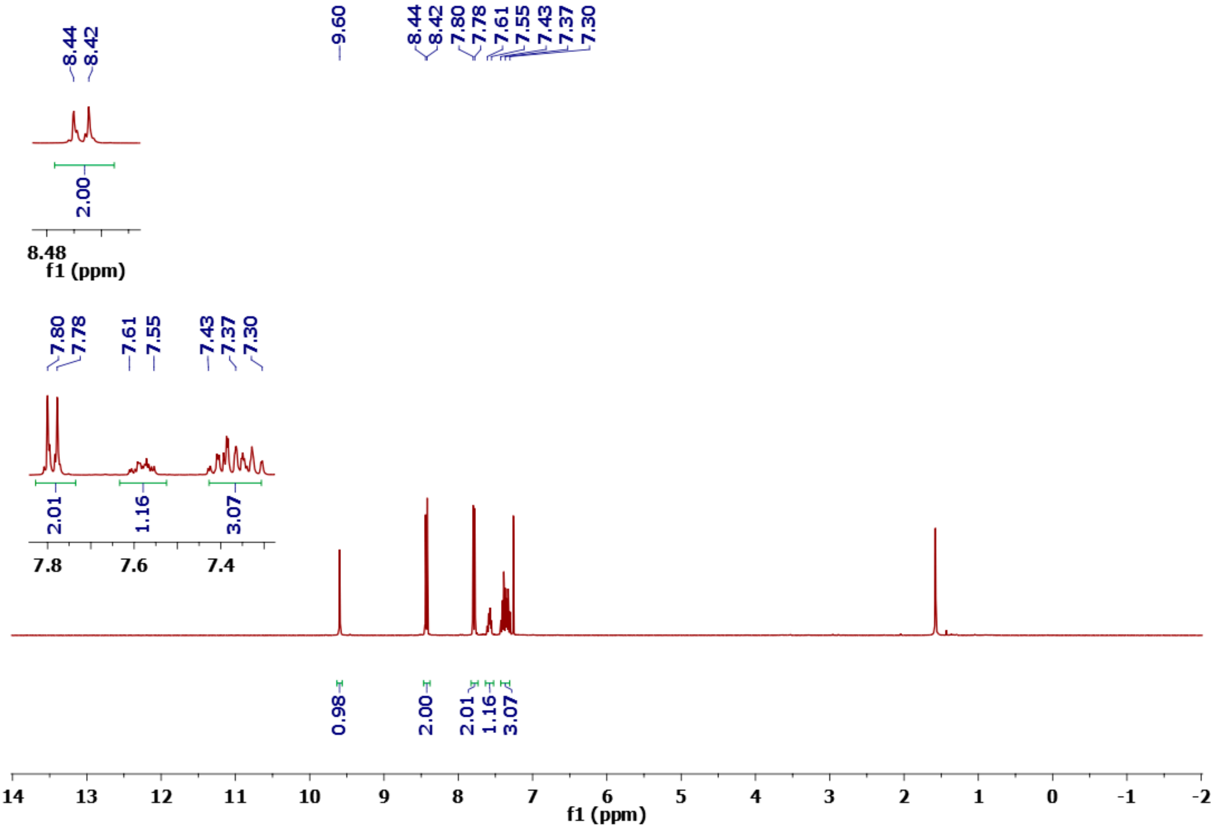

^{13}C-CRAPT NMR ($CDCl_3$) spectrum of 3-(2-fluorophenyl)-5-imino-1-(4-nitrophenyl)imidazolidine-2,4-dithione

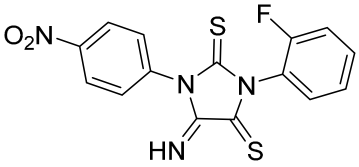

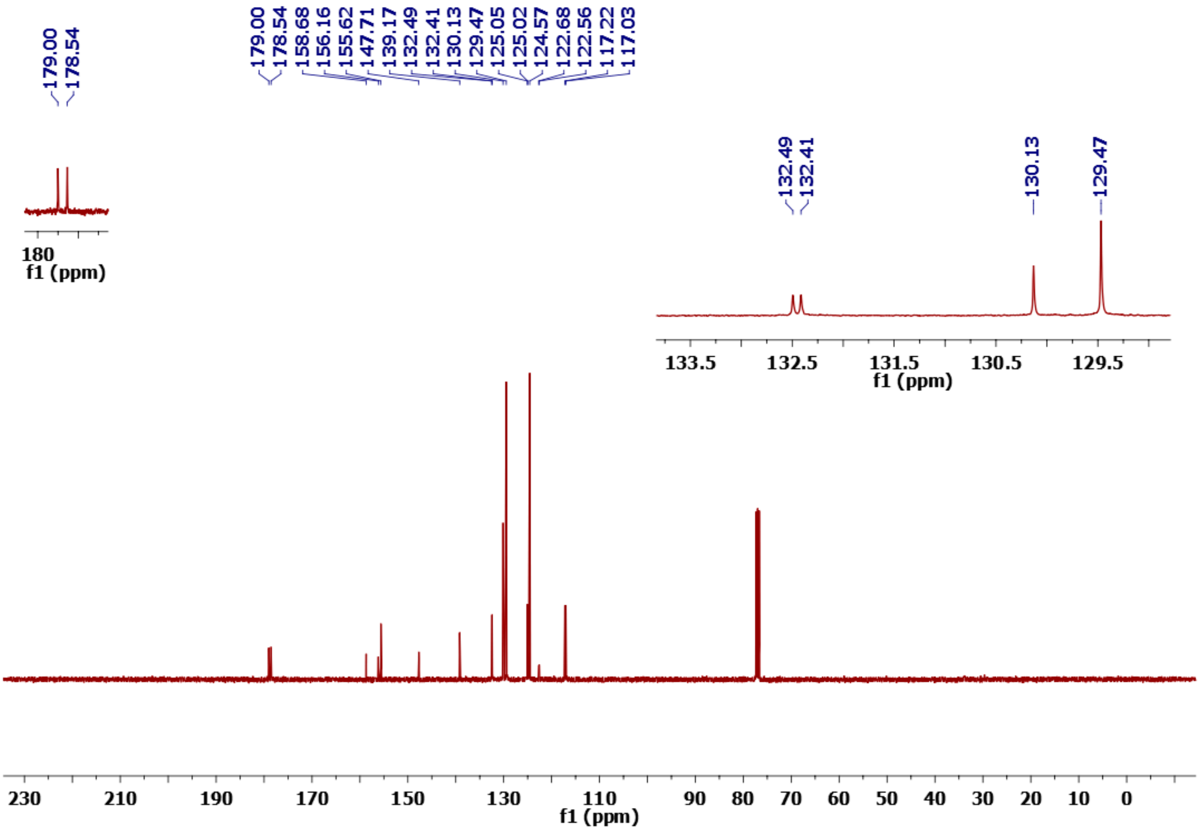

^{13}C-CRAPT NMR ($CDCl_3$) spectrum of 3-(2-fluorophenyl)-5-imino-1-(4-nitrophenyl)imidazolidine-2,4-dithione

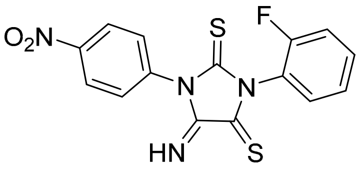

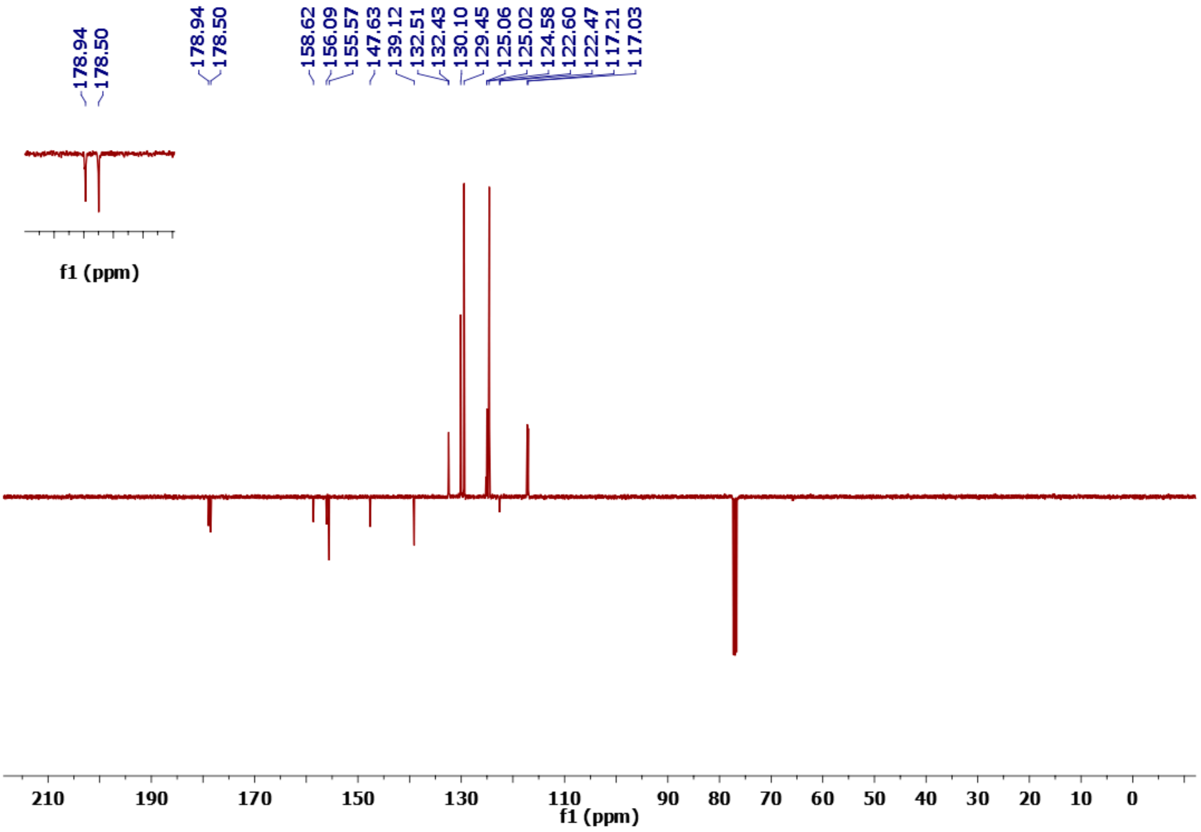

^{1}H-^{1}H-gCOSYAD NMR ($CDCl_3$) spectrum of 3-(2-fluorophenyl)-5-imino-1-(4-nitrophenyl)imidazolidine-2,4-dithione

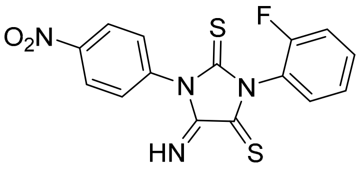

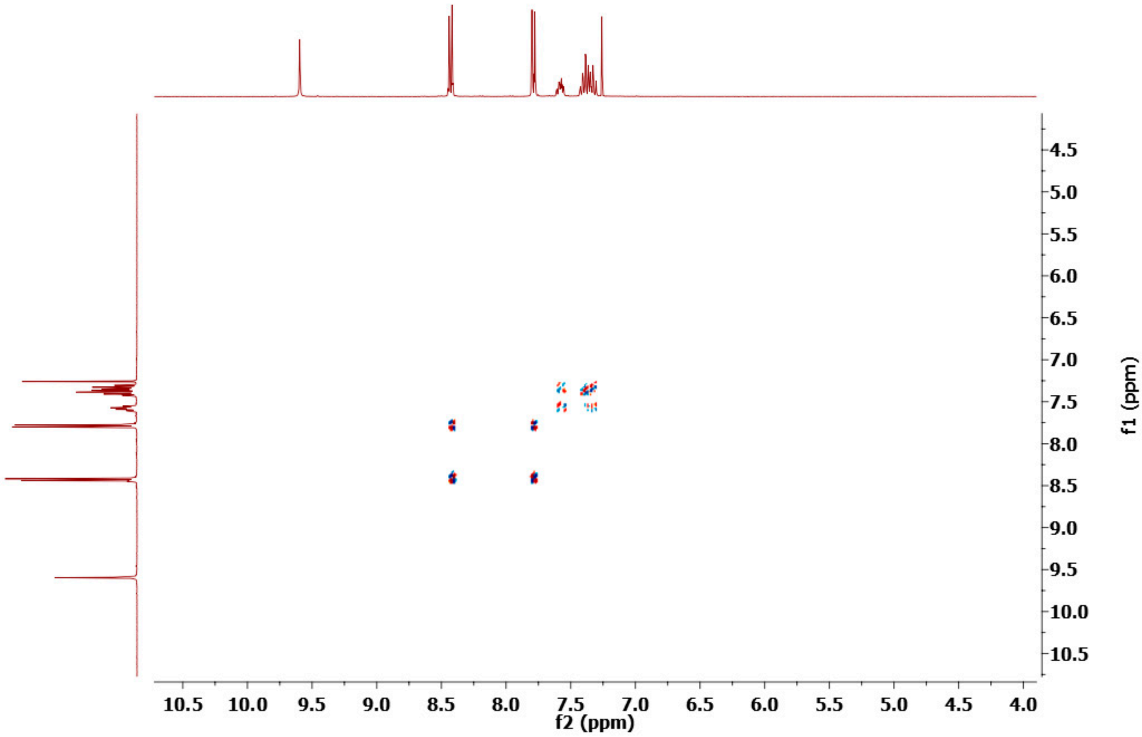

^{1}H-^{13}C-gHSQCAD NMR ($CDCl_3$) spectrum of 3-(2-fluorophenyl)-5-imino-1-(4-nitrophenyl)imidazolidine-2,4-dithione

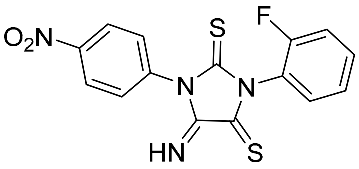

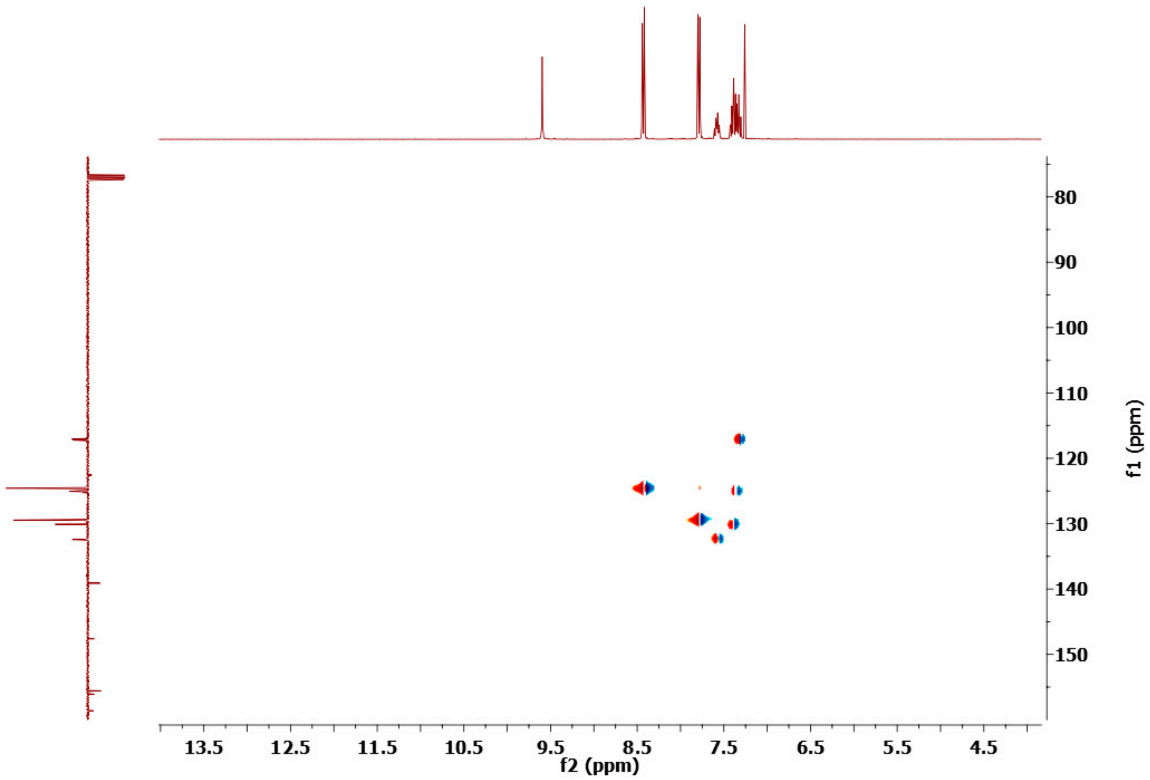

^{1}H-^{13}C-HMBC NMR ($CDCl_3$) spectrum of 3-(2-fluorophenyl)-5-imino-1-(4-nitrophenyl)imidazolidine-2,4-dithione

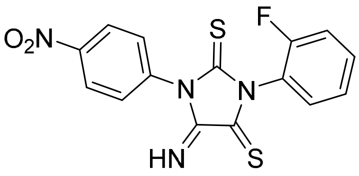

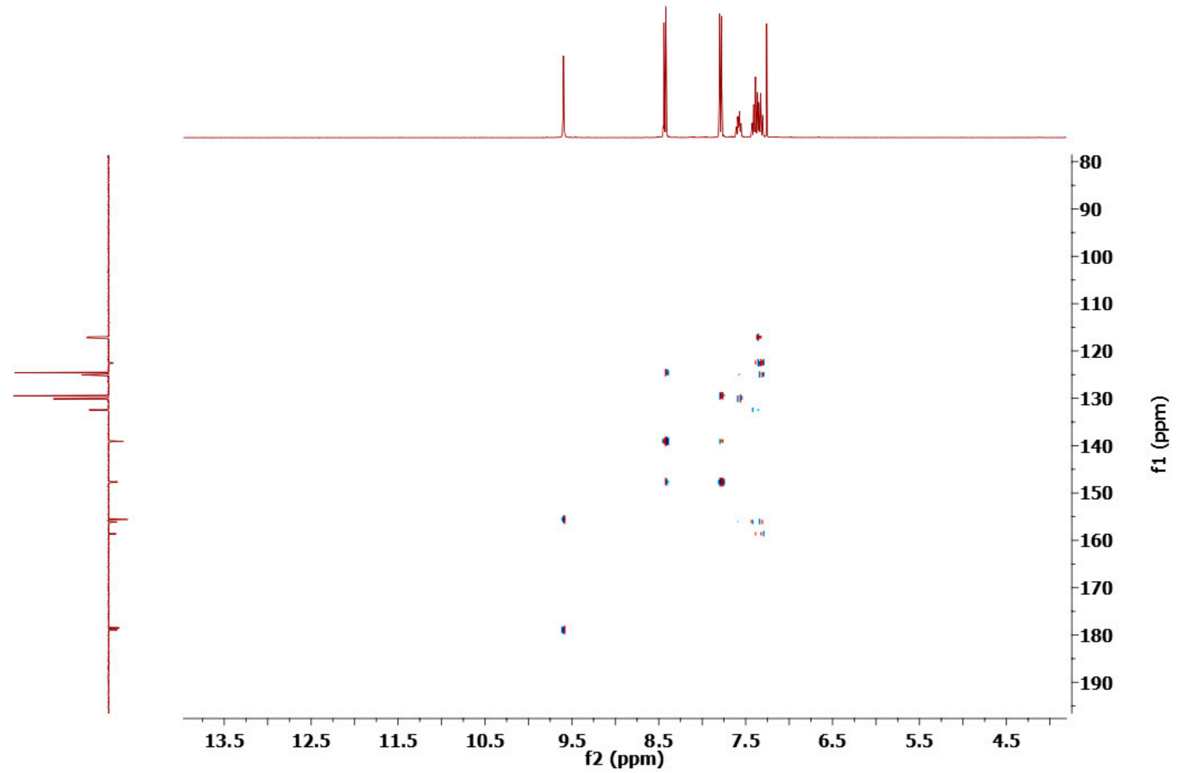

^{1}H NMR ($CDCl_3$) spectrum of 3-(2-fluorophenyl)-5-imino-1-(4-methoxyphenyl)imidazolidine-2,4-dithione (18y)

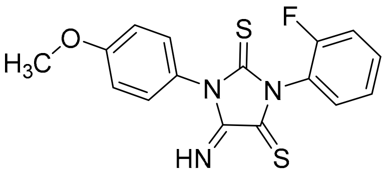

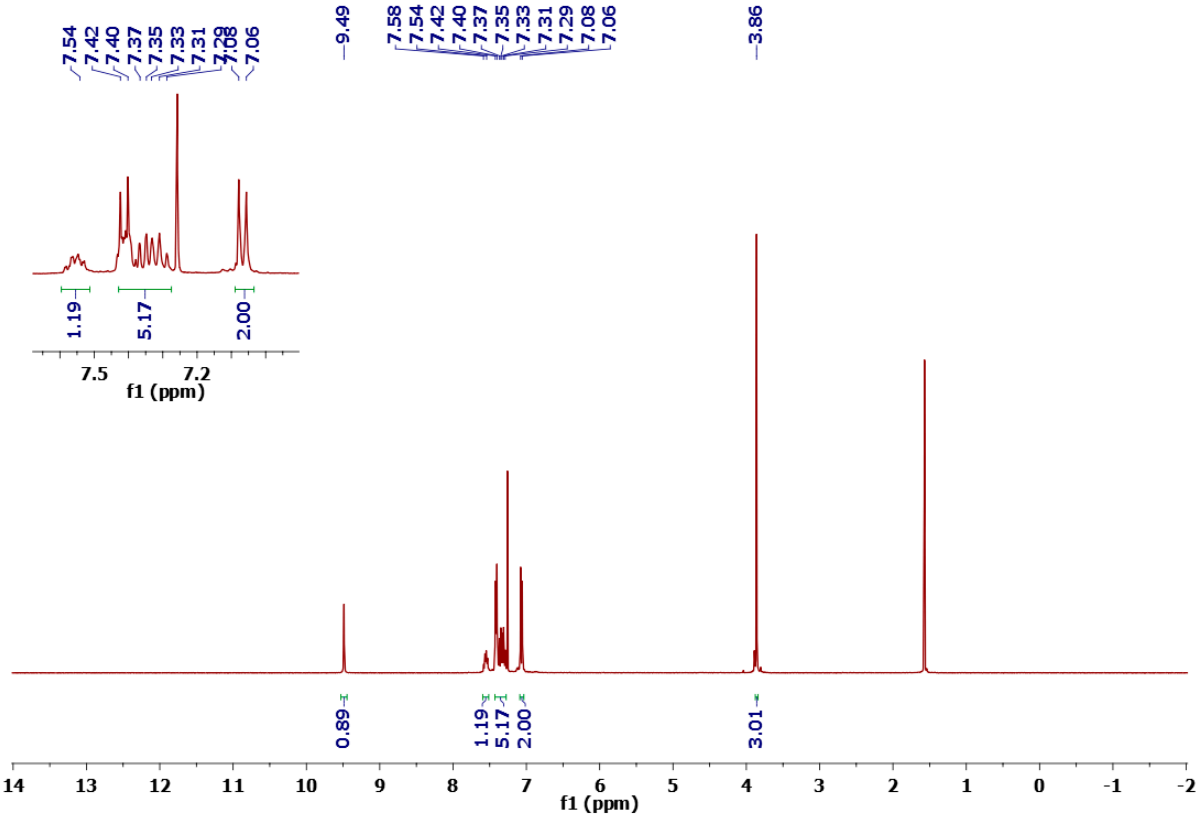

^{13}C NMR ($CDCl_3$) spectrum of 3-(2-fluorophenyl)-5-imino-1-(4-methoxyphenyl)imidazolidine-2,4-dithione

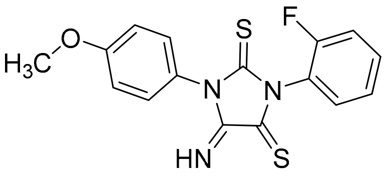

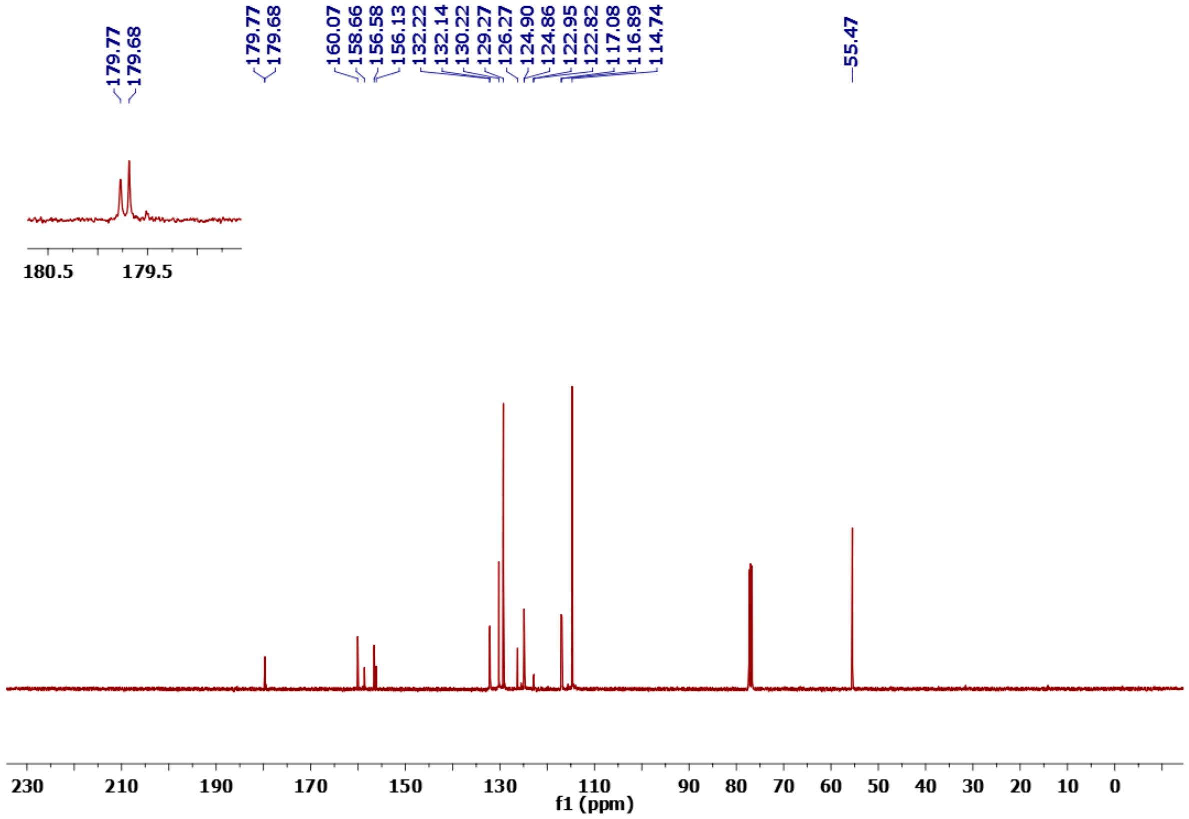

^{13}C-CRAPT NMR (CDCl₃) spectrum of 3-(2-fluorophenyl)-5-imino-1-(4-methoxyphenyl)imidazolidine-2,4-dithione

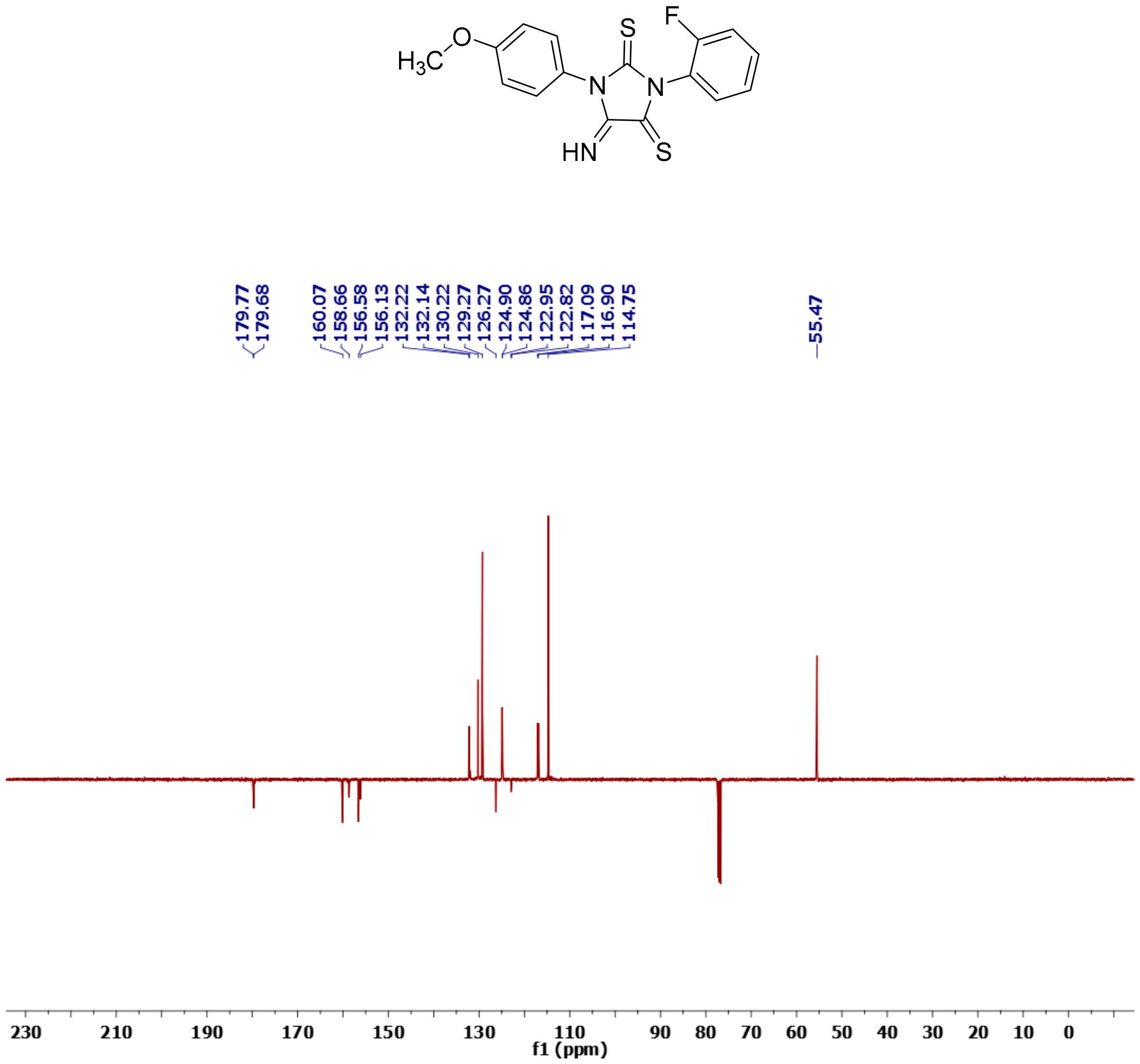

^{1}H-^{1}H-gCOSYAD NMR ($CDCl_3$) spectrum of 3-(2-fluorophenyl)-5-imino-1-(4-methoxyphenyl)imidazolidine-2,4-dithione

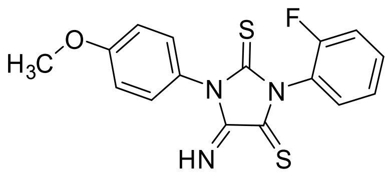

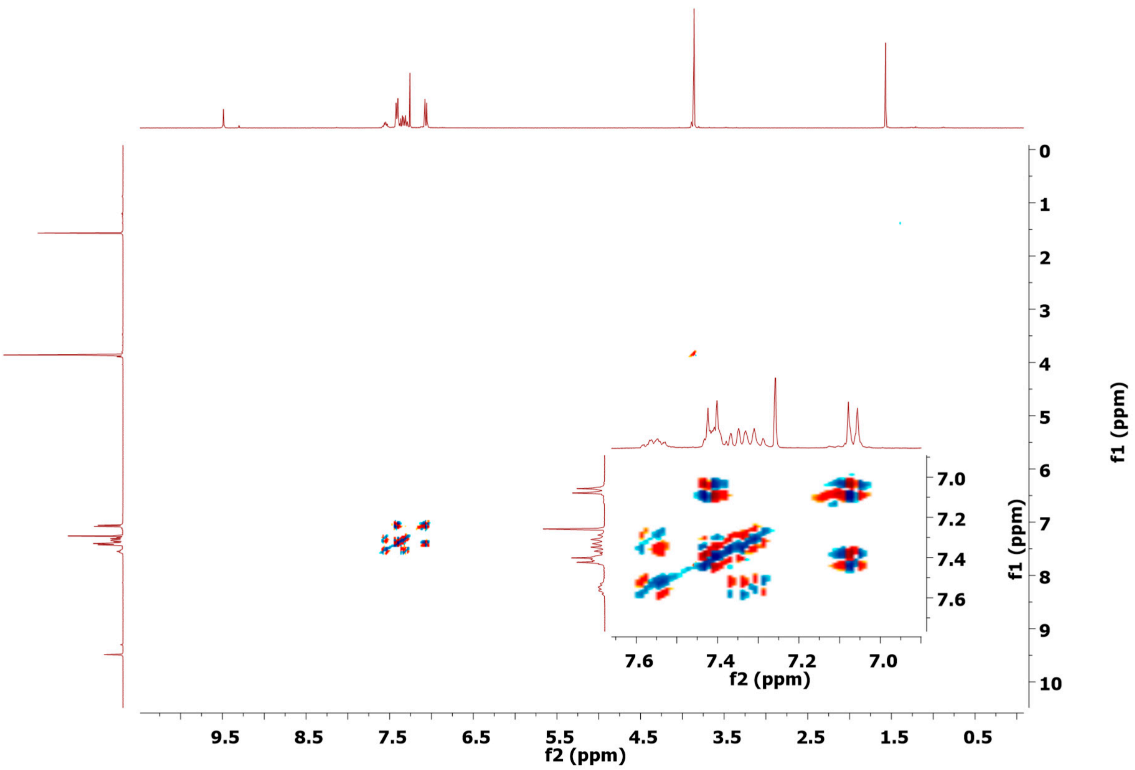

^{1}H-^{13}C-gHSQCAD NMR ($CDCl_3$) spectrum of 3-(2-fluorophenyl)-5-imino-1-(4-methoxyphenyl)imidazolidine-2,4-dithione

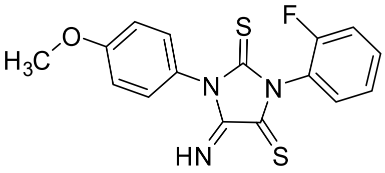

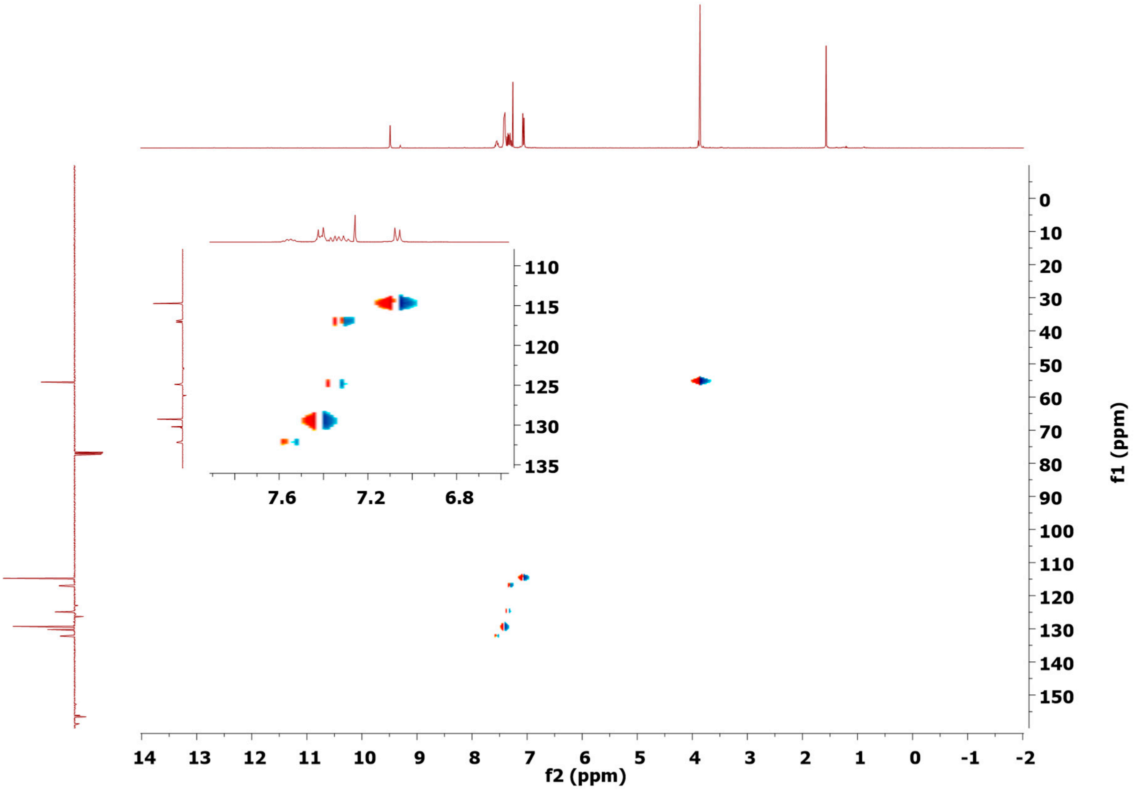

^{1}H-^{13}C-gHMBC NMR ($CDCl_3$) spectrum of 3-(2-fluorophenyl)-5-imino-1-(4-methoxyphenyl)imidazolidine-2,4-dithione

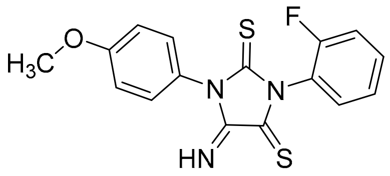

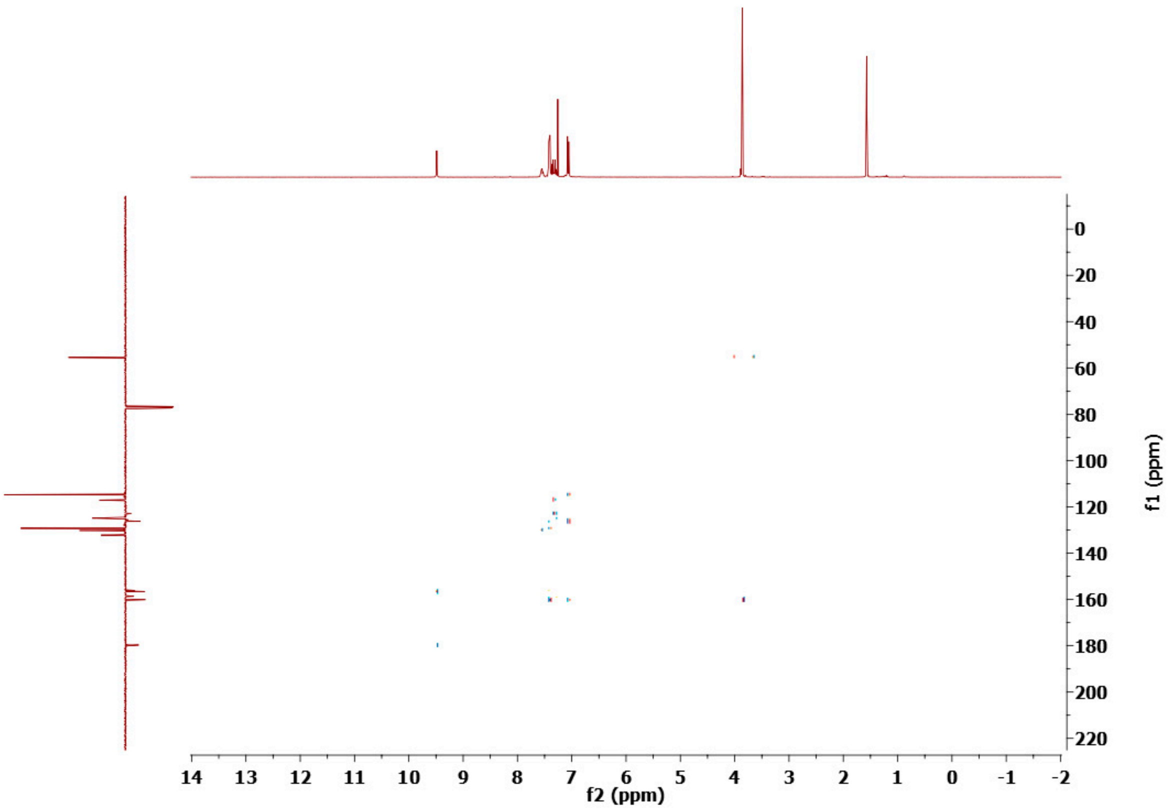

^{1}H NMR ($CDCl_3$) spectrum of (5-imino-2,4-dithioxo-3-(p-tolyl)imidazolidin-1-yl)(phenyl)methanone (18z)

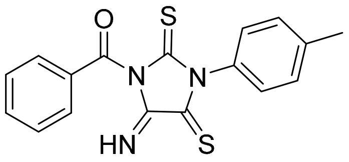

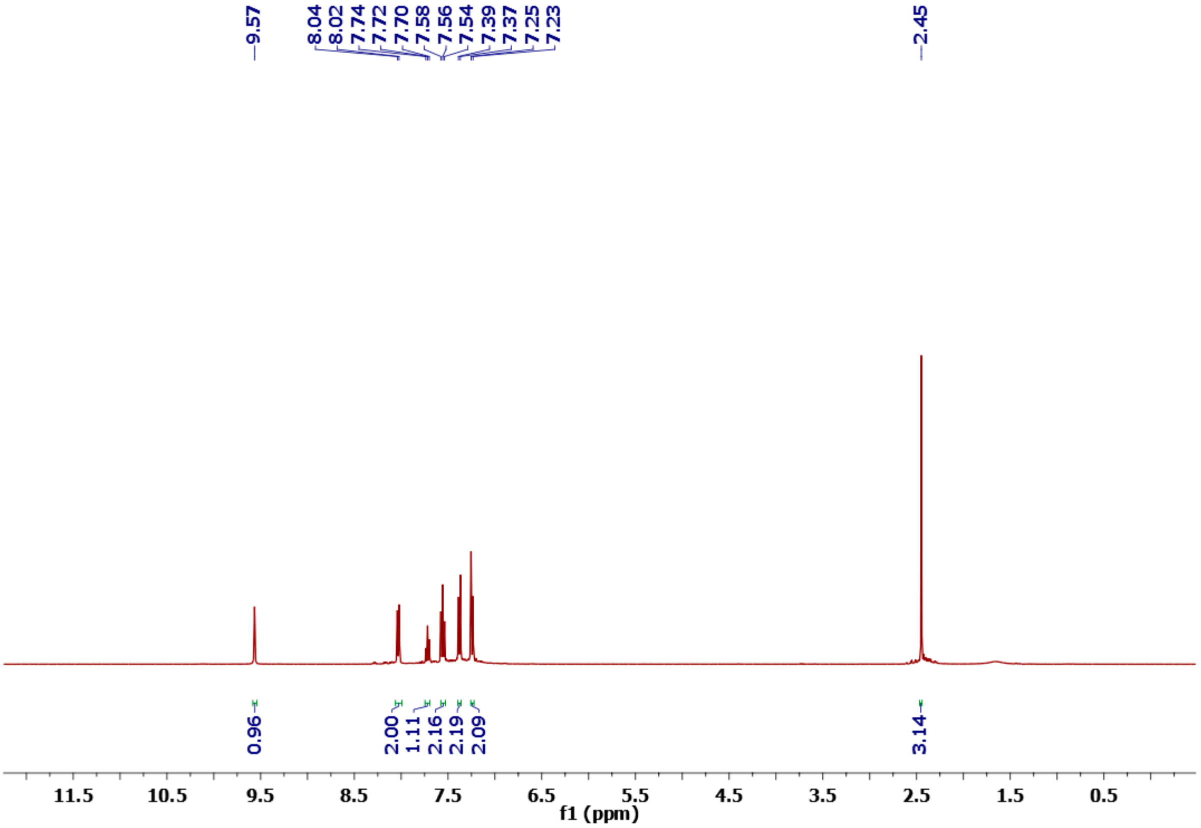

^{13}C NMR ($CDCl_3$) spectrum of (5-imino-2,4-dithioxo-3-(p-tolyl)imidazolidin-1-yl)(phenyl)methanone

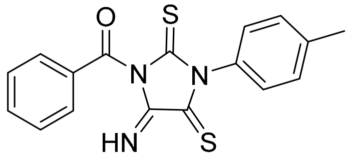

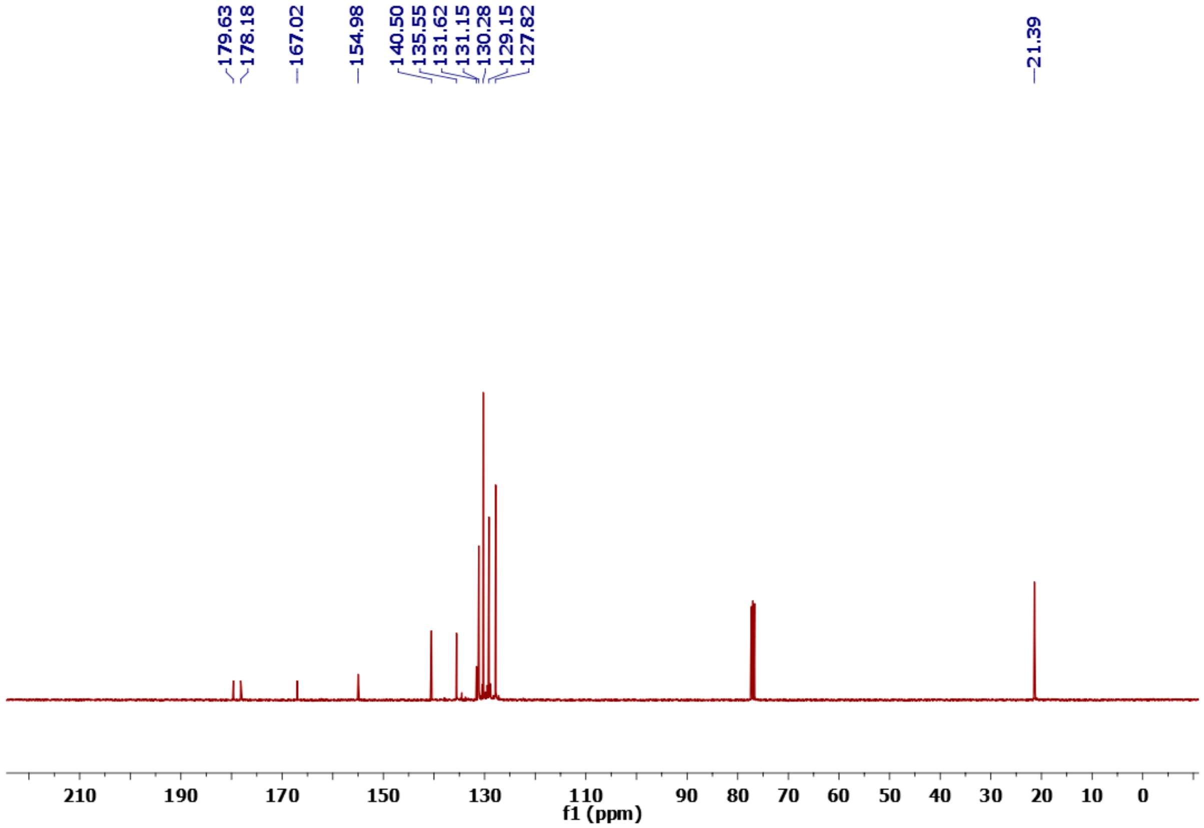

^{13}C-CRAPT NMR ($CDCl_3$) spectrum of (5-imino-2,4-dithioxo-3-(p-tolyl)imidazolidin-1-yl)(phenyl)methanone

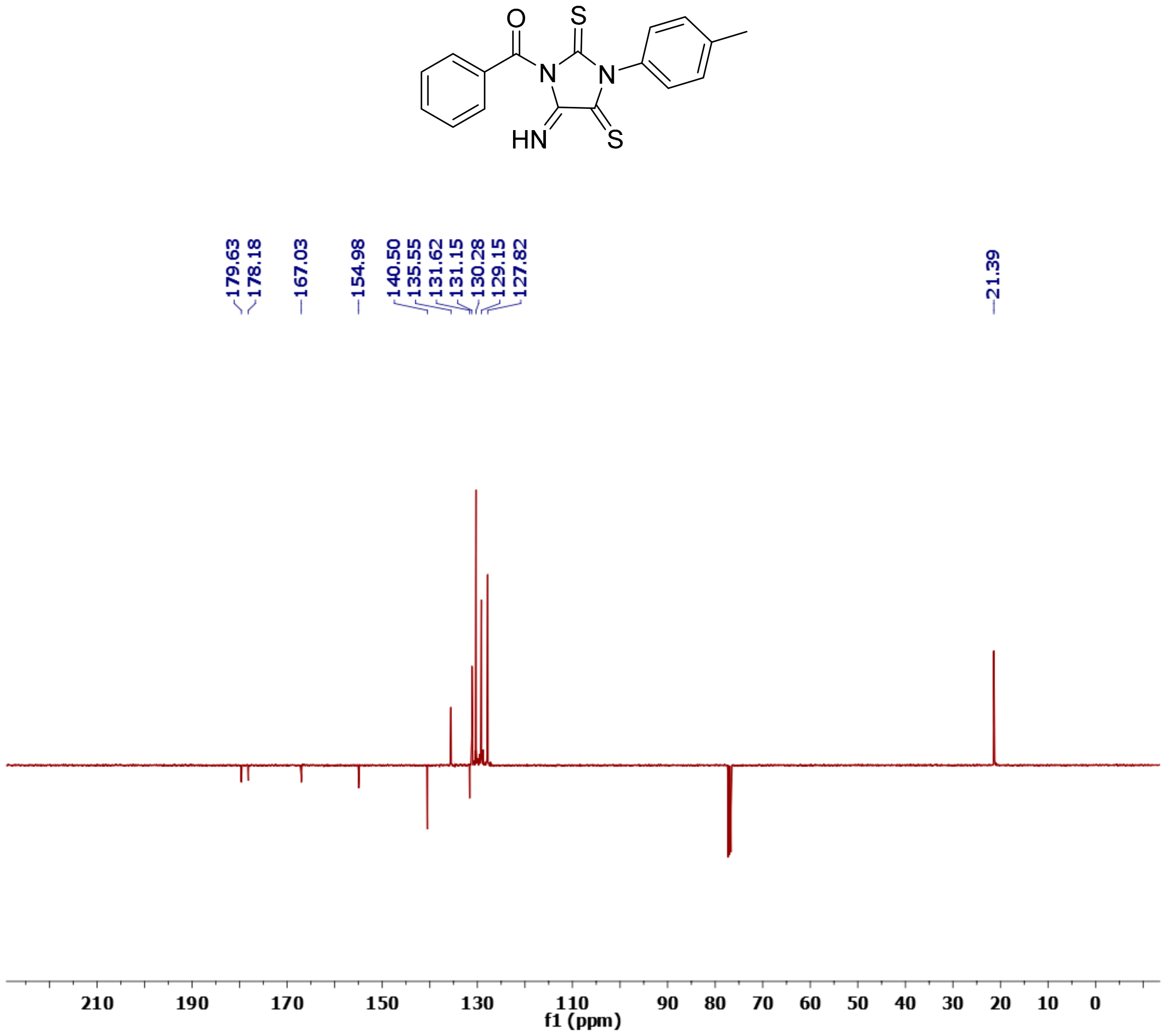

^{1}H-^{1}H-gCOSYAD NMR ($CDCl_3$) spectrum of (5-imino-2,4-dithioxo-3-(p-tolyl)imidazolidin-1-yl)(phenyl)methanone

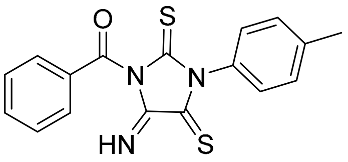

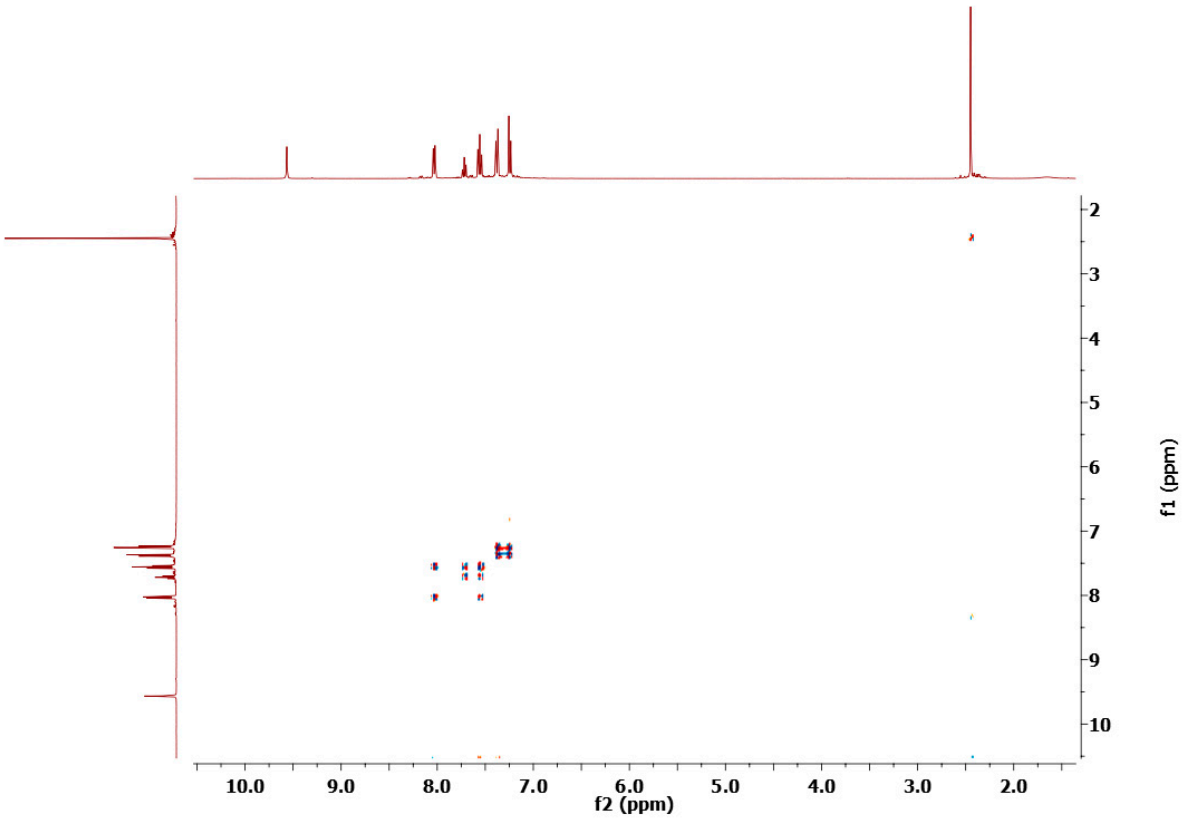

^{1}H-^{13}C-gHSQCAD NMR ($CDCl_3$) spectrum of (5-imino-2,4-dithioxo-3-(p-tolyl)imidazolidin-1-yl)(phenyl)methanone

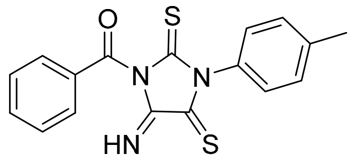

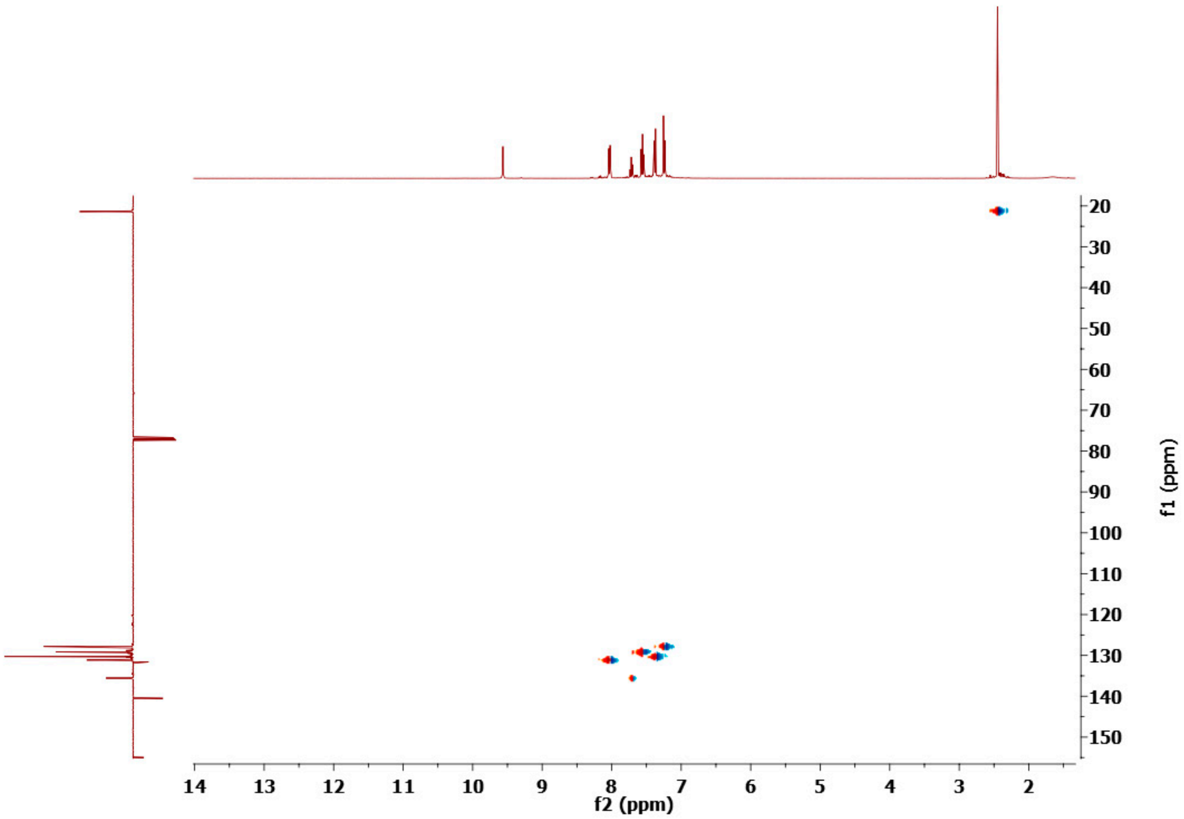

^{1}H-^{13}C-gHMBC NMR ($CDCl_3$) spectrum of (5-imino-2,4-dithioxo-3-(p-tolyl)imidazolidin-1-yl)(phenyl)methanone

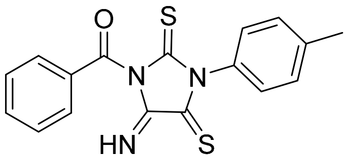

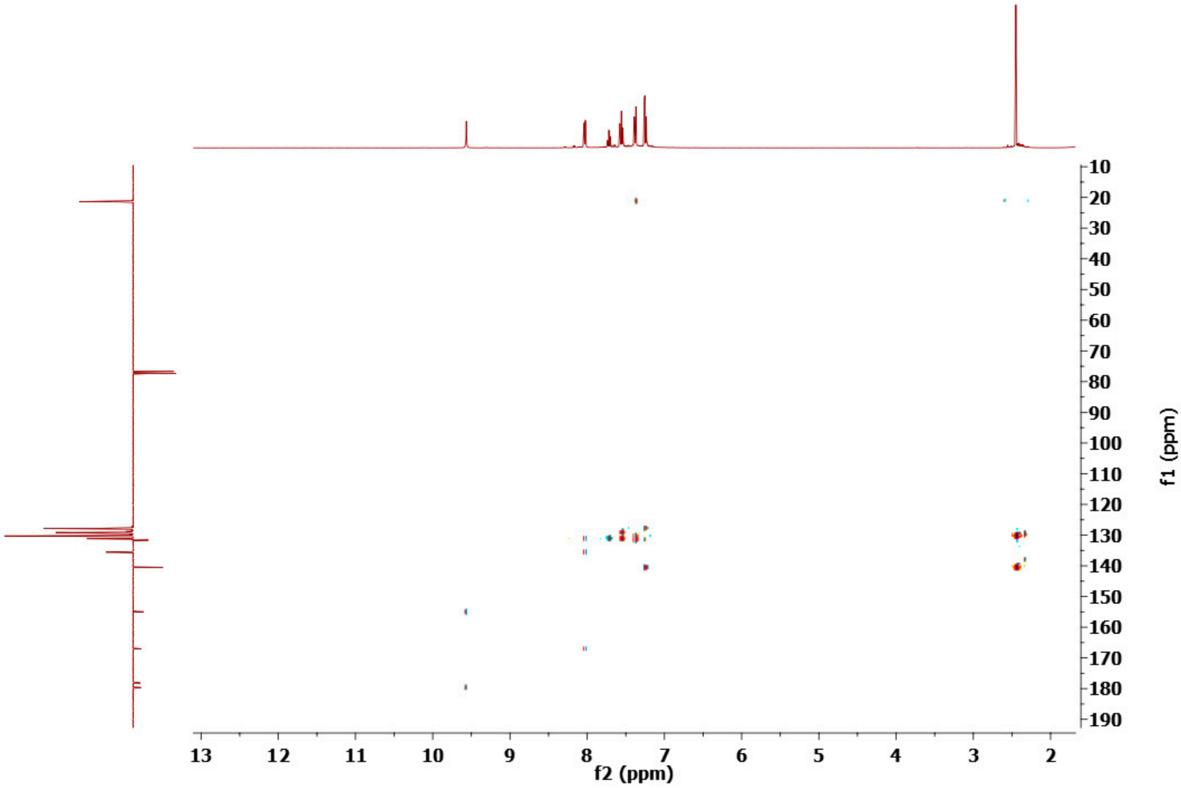

^{1}H NMR ($CDCl_3$) spectrum of 5-imino-1-phenyl-3-(p-tolyl)imidazolidine-2,4-dithione (18a')

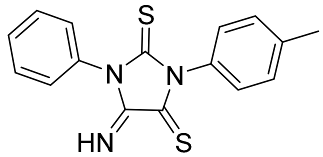

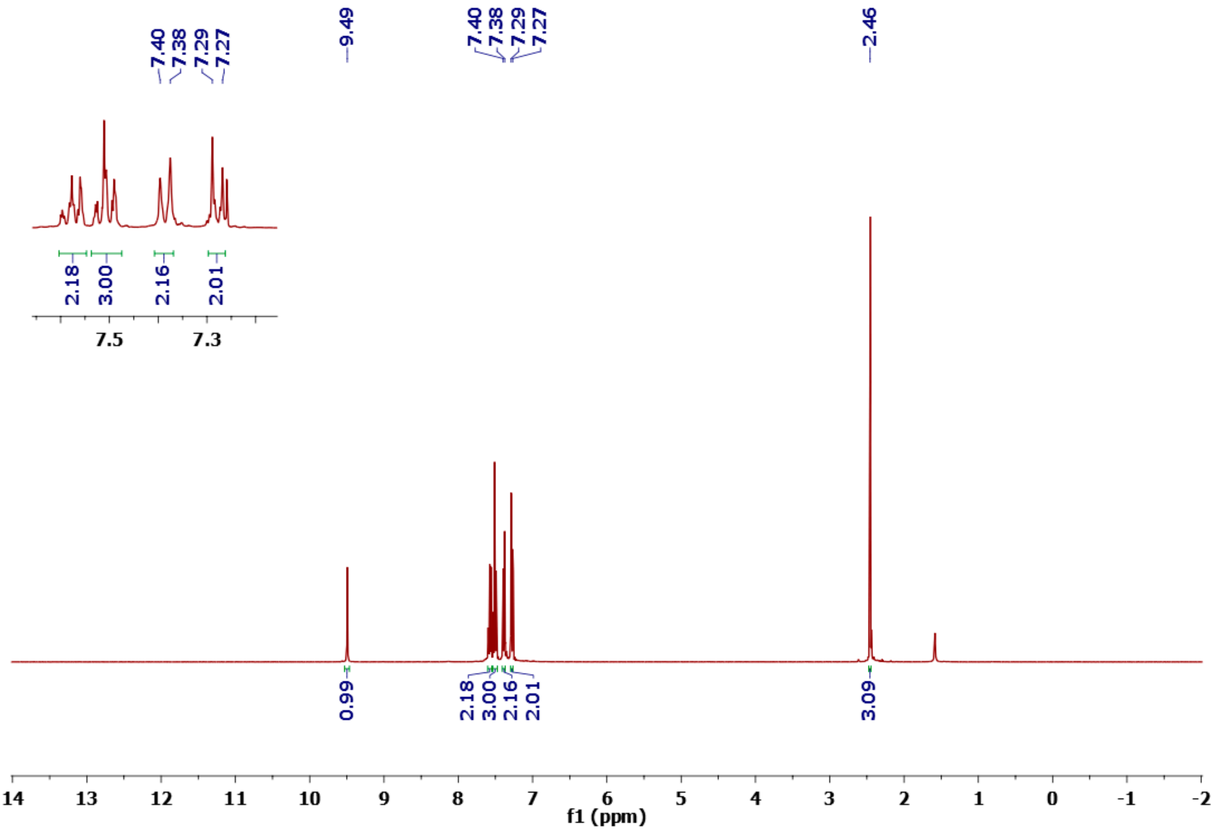

^{13}C NMR ($CDCl_3$) spectrum of 5-imino-1-phenyl-3-(p-tolyl)imidazolidine-2,4-dithione

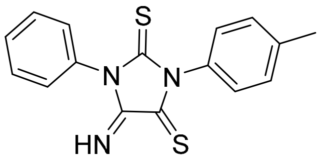

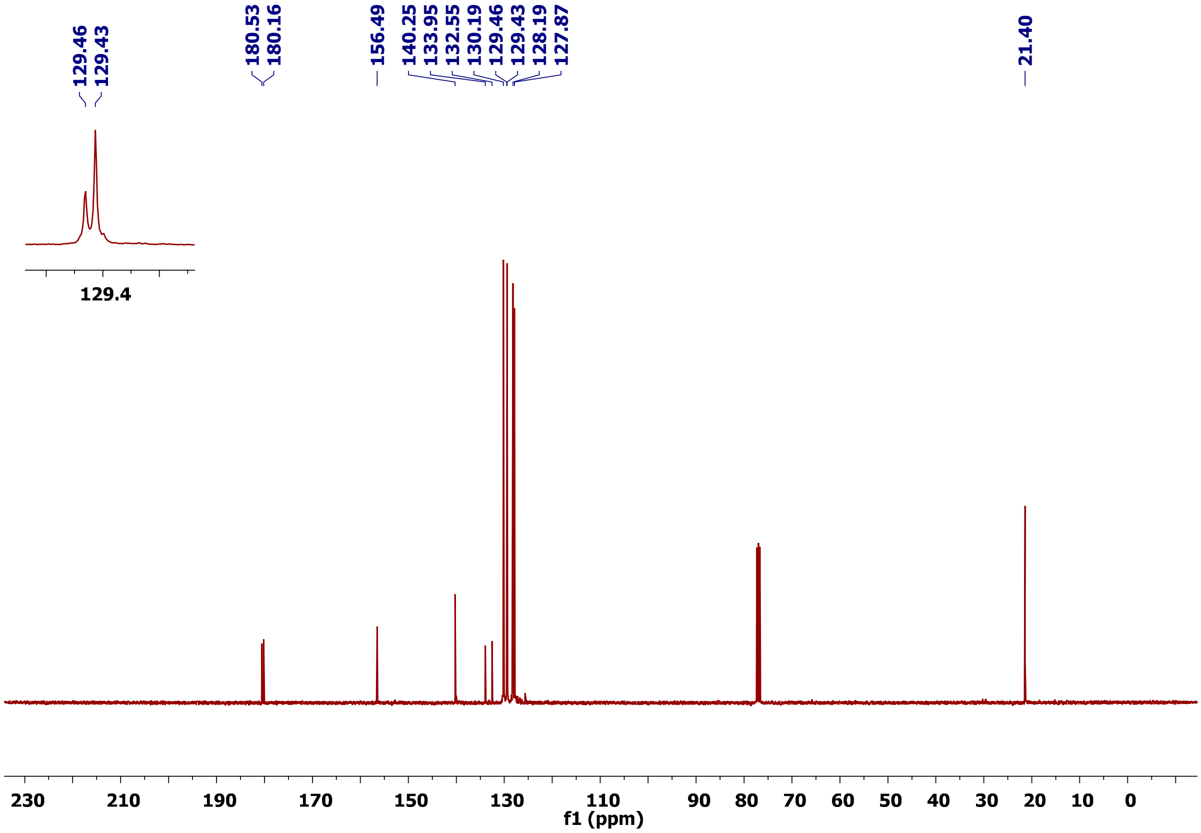

^{13}C-CRAPT NMR ($CDCl_3$) spectrum of 5-imino-1-phenyl-3-(p-tolyl)imidazolidine-2,4-dithione

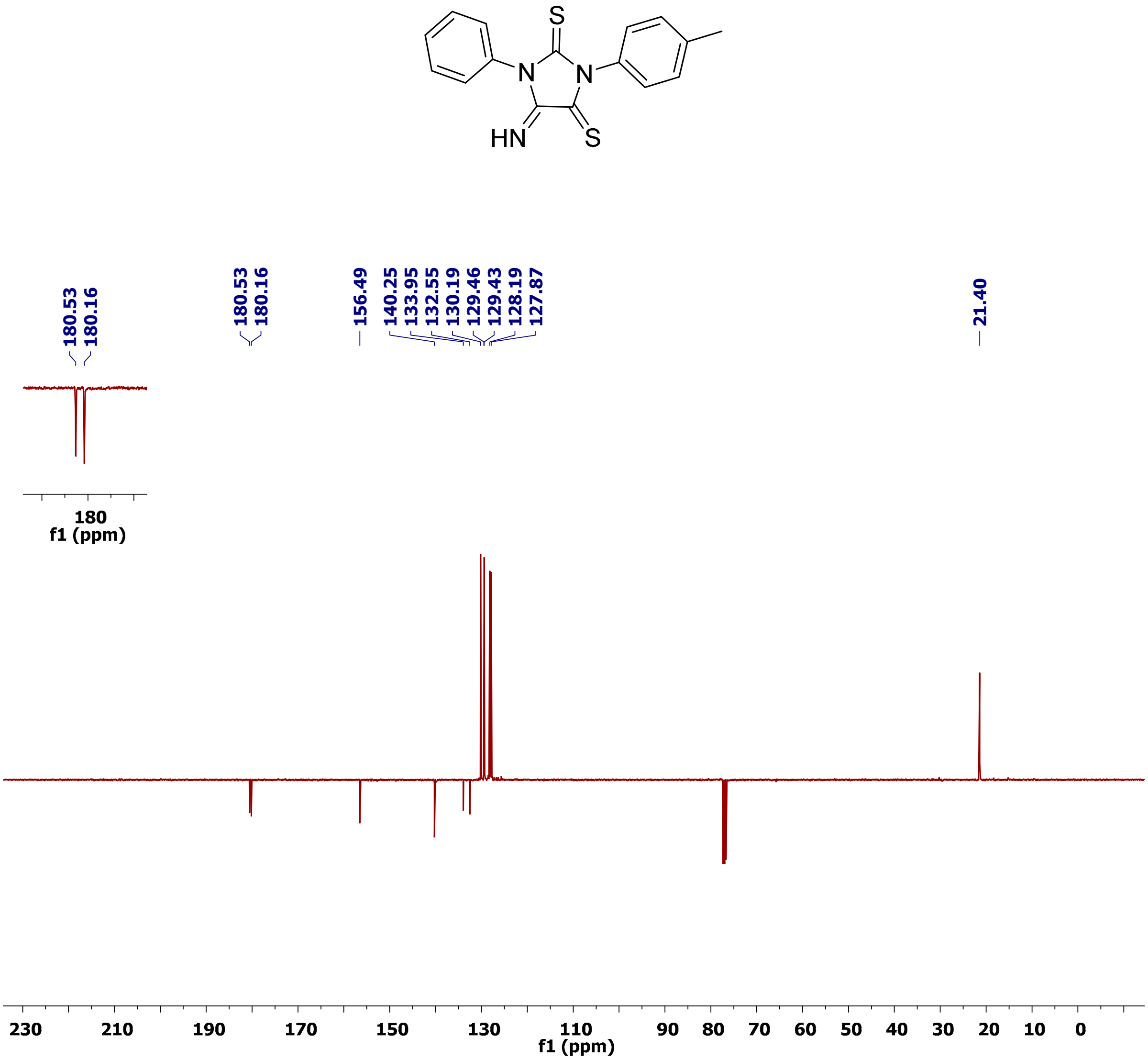

^{1}H-^{1}H-gCOSYAD NMR ($CDCl_3$) spectrum of 5-imino-1-phenyl-3-(p-tolyl)imidazolidine-2,4-dithione

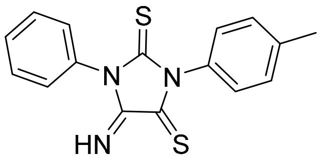

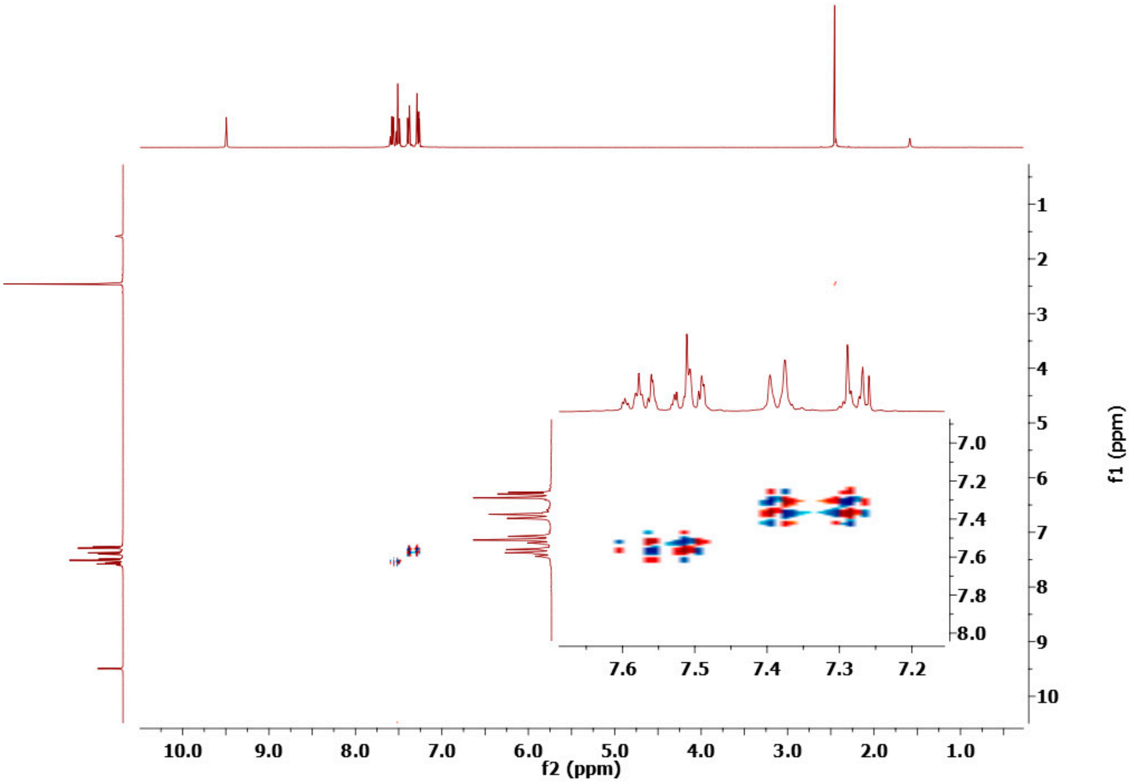

^{1}H-^{13}C-gHSQCAD ($CDCl_3$) spectrum of 5-imino-1-phenyl-3-(p-tolyl)imidazolidine-2,4-dithione

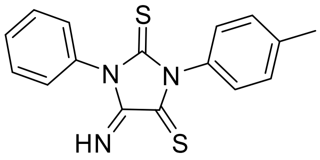

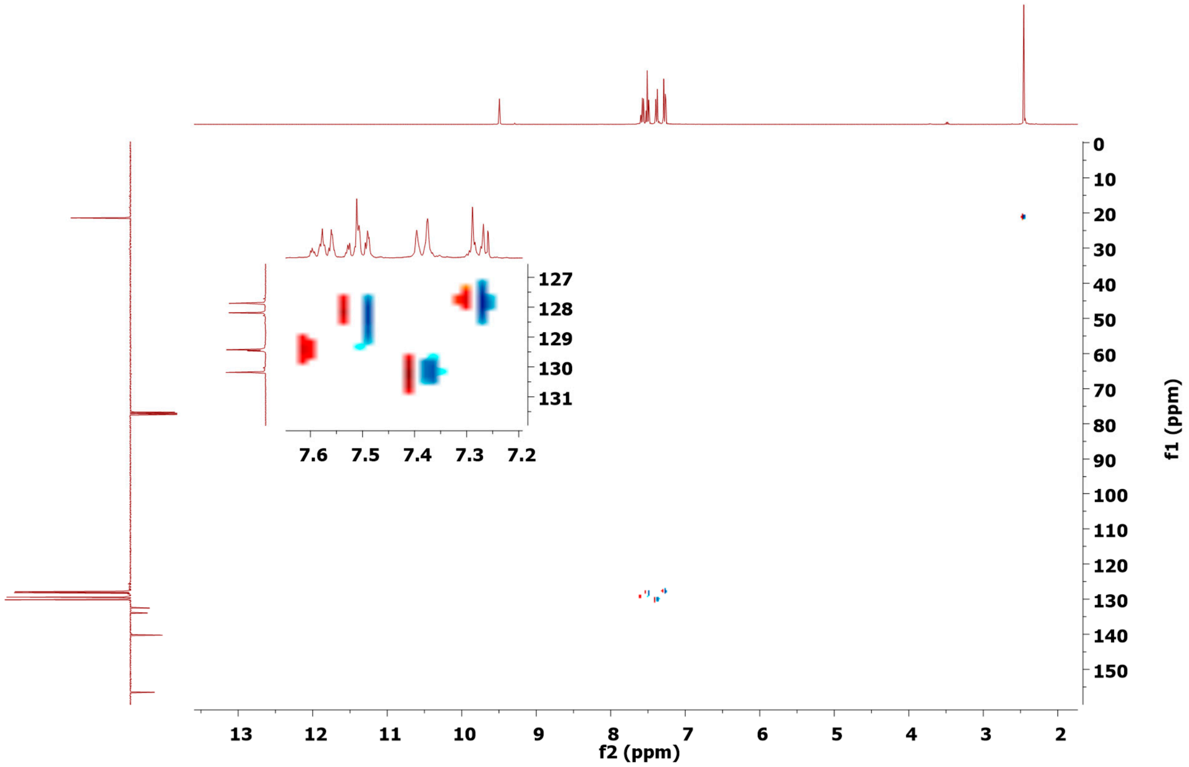

^{1}H-^{13}C-gHMBC ($CDCl_3$) spectrum of 5-imino-1-phenyl-3-(p-tolyl)imidazolidine-2,4-dithione

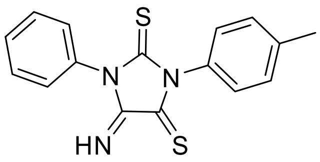

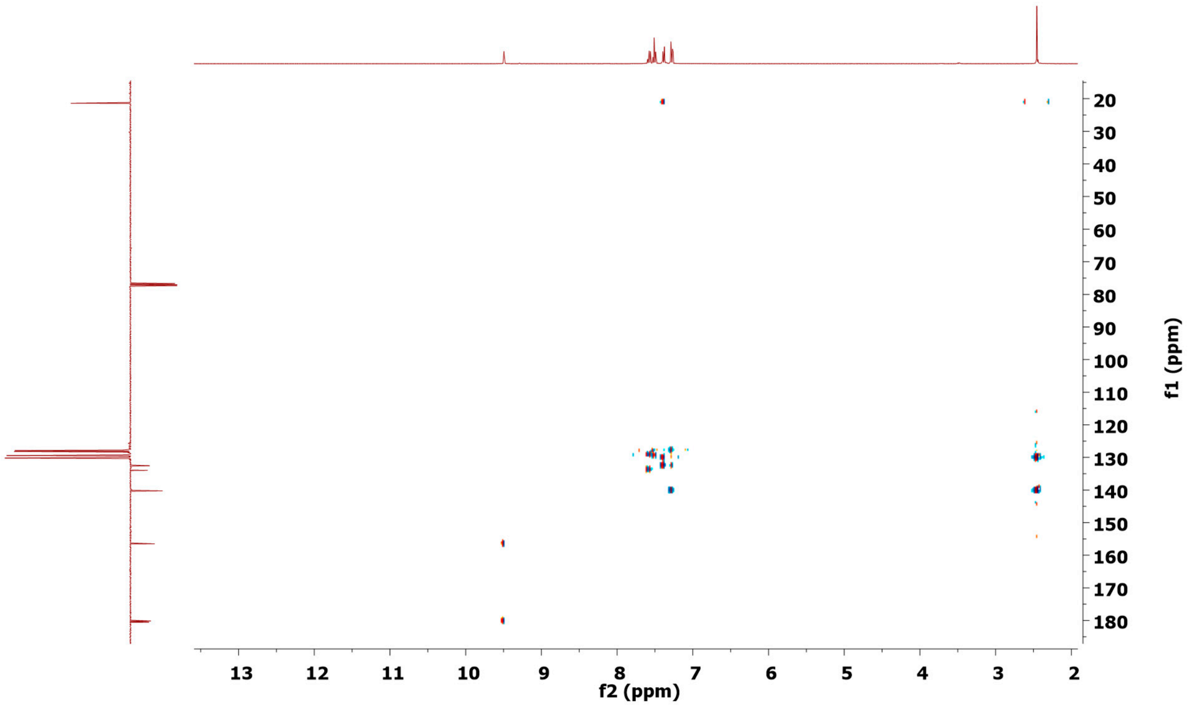

^{1}H NMR ($CDCl_3$) spectrum of 5-imino-3-(p-tolyl)-1-(4-(trifluoromethyl)phenyl)imidazolidine-2,4-dithione (18b')

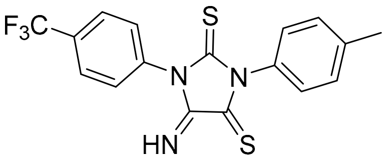

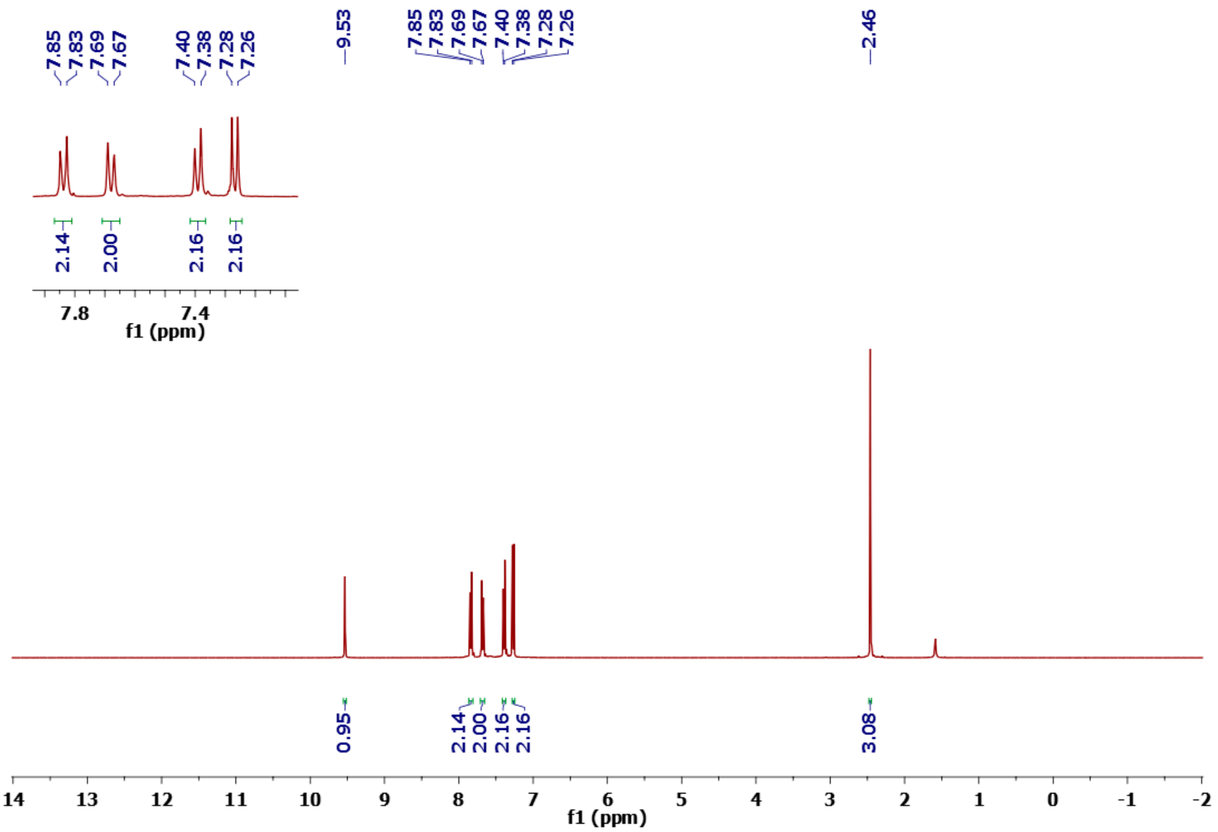

^{13}C NMR ($CDCl_3$) spectrum of 5-imino-3-(p-tolyl)-1-(4-(trifluoromethyl)phenyl)imidazolidine-2,4-dithione

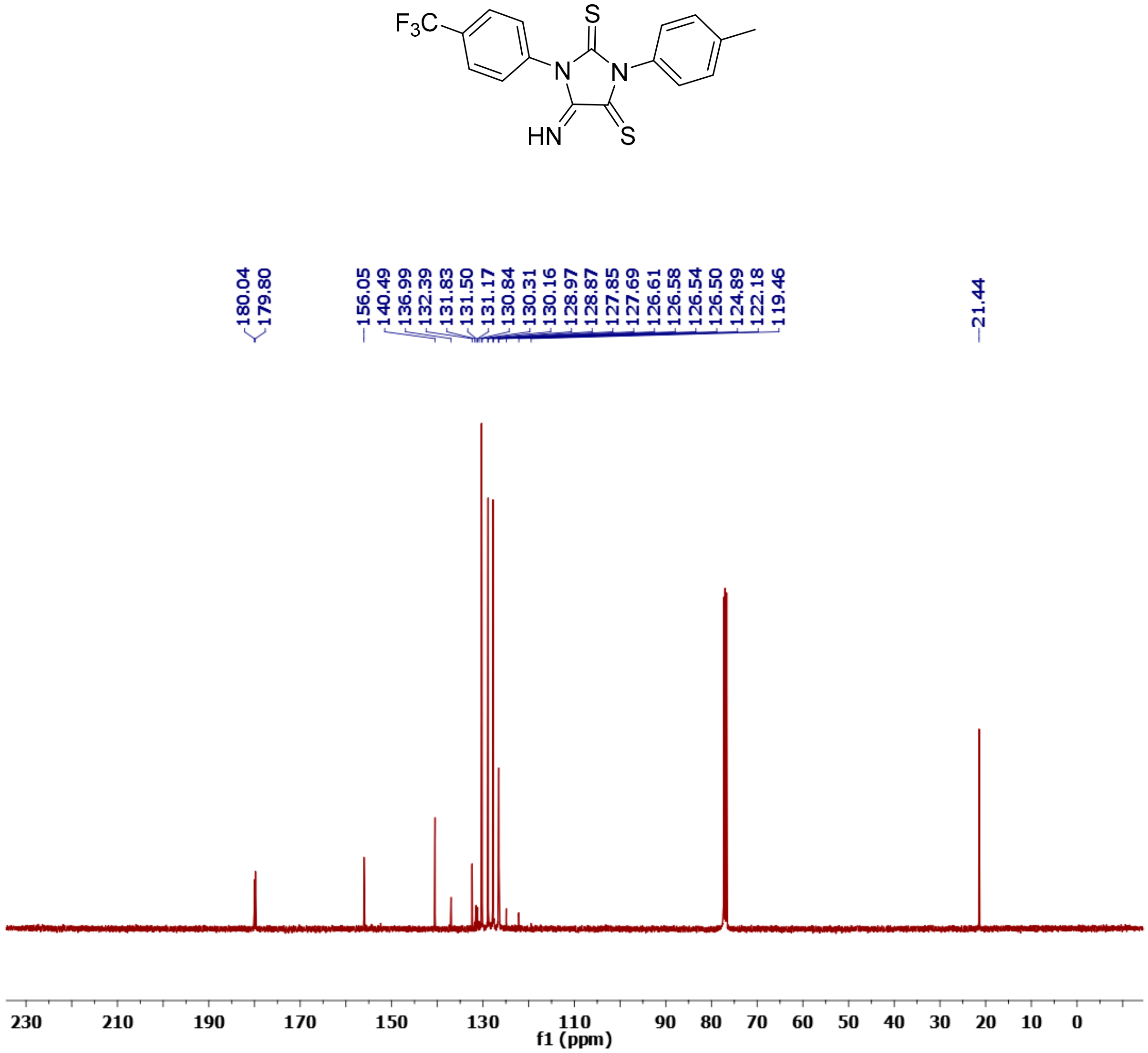

^{13}C-CRAPT NMR ($CDCl_3$) spectrum of 5-imino-3-(p-tolyl)-1-(4-(trifluoromethyl)phenyl)imidazolidine-2,4-dithione

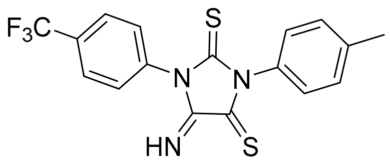

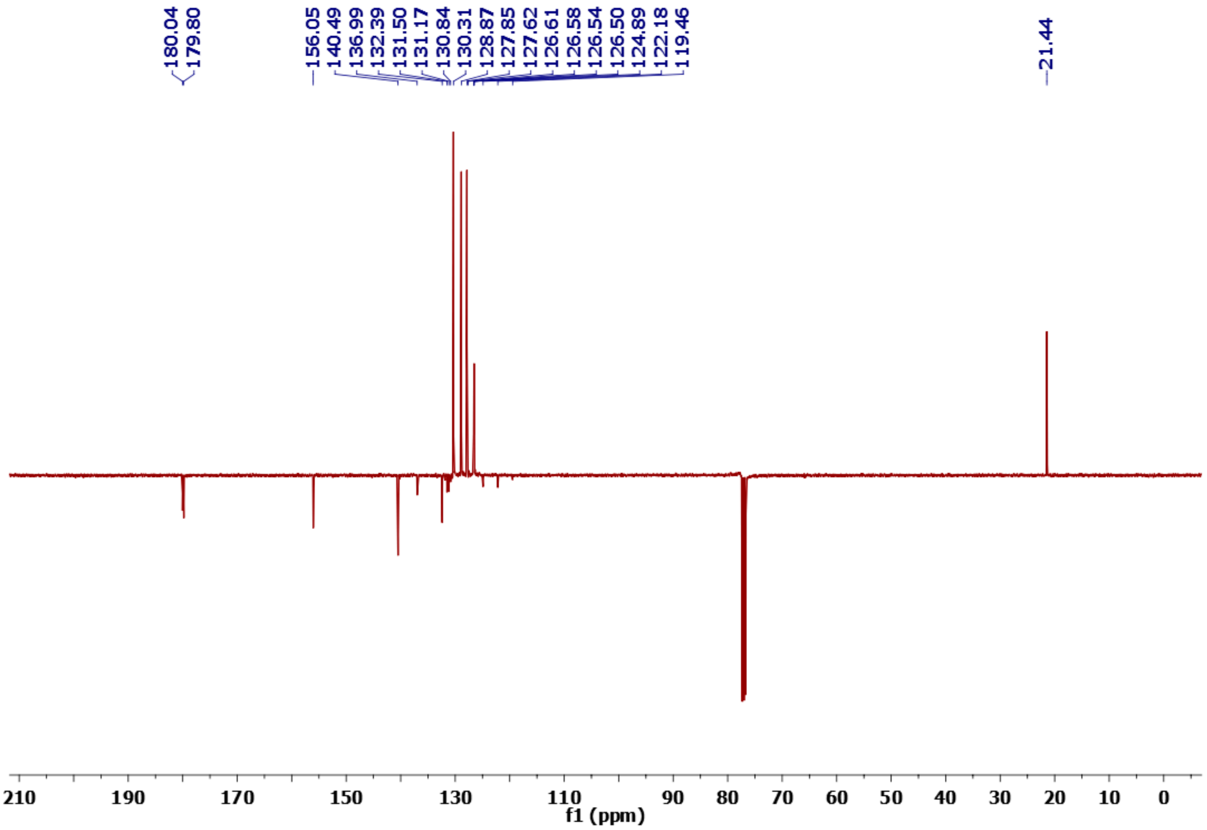

^{1}H-^{1}H-gCOSYAD NMR ($CDCl_3$) spectrum of 5-imino-3-(p-tolyl)-1-(4-(trifluoromethyl)phenyl)imidazolidine-2,4-dithione

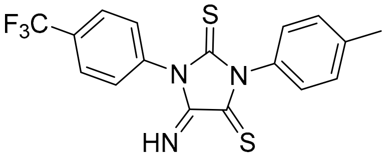

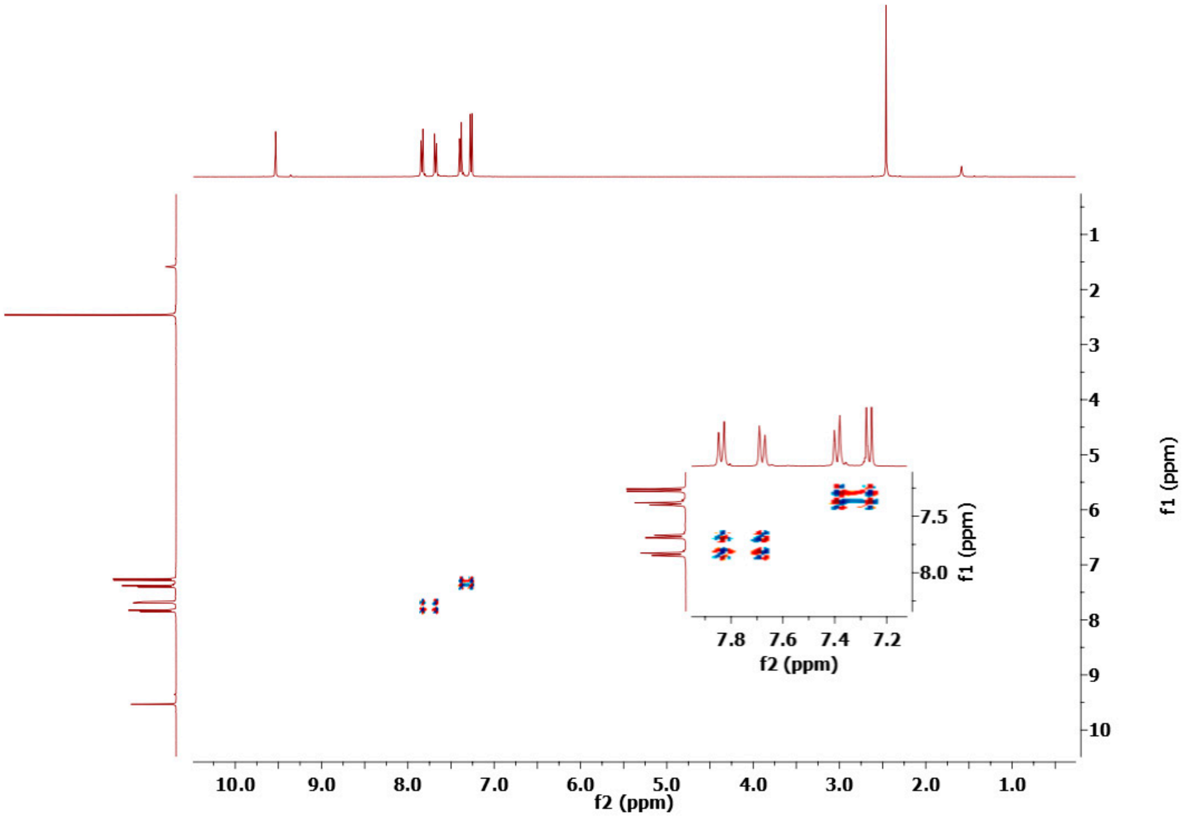

^{1}H-^{13}C-gHSQCAD NMR ($CDCl_3$) spectrum of 5-imino-3-(p-tolyl)-1-(4-(trifluoromethyl)phenyl)imidazolidine-2,4-dithione

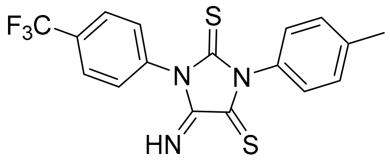

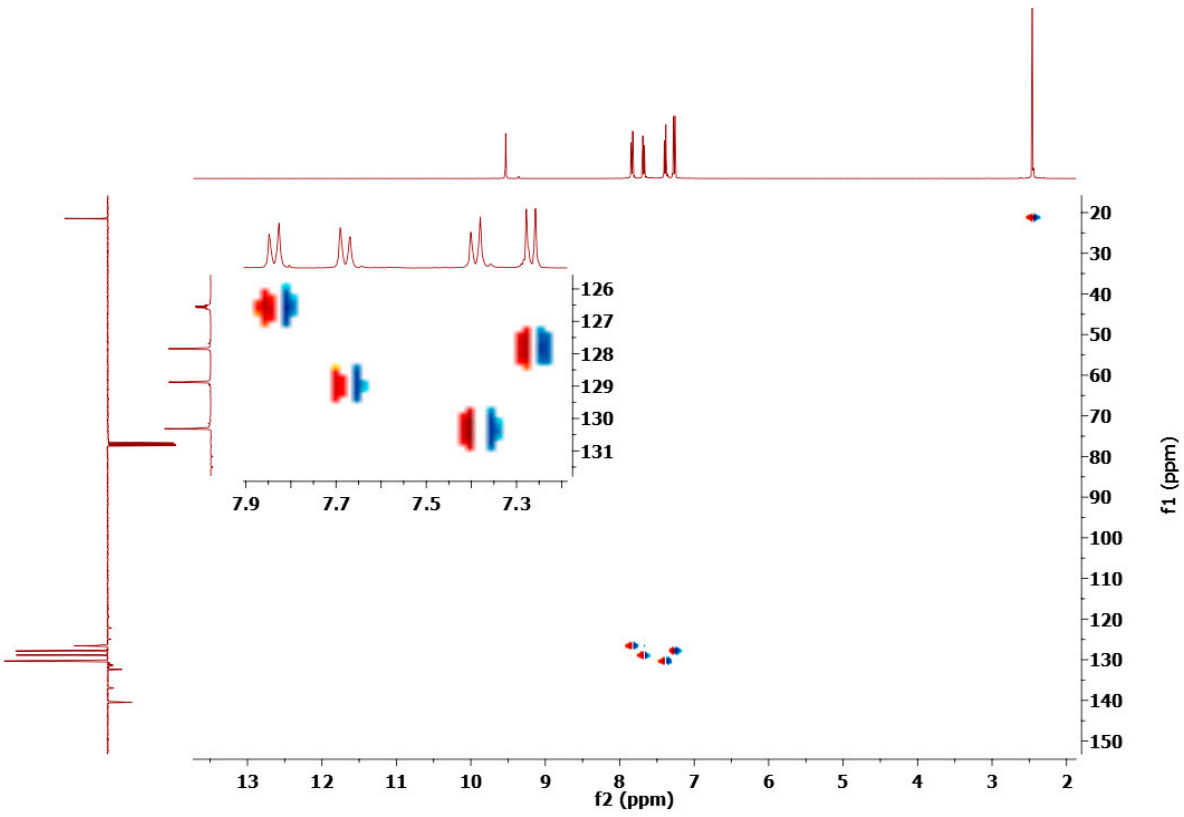

^{1}H-^{13}C-gHMBC NMR ($CDCl_3$) spectrum of 5-imino-3-(p-tolyl)-1-(4-(trifluoromethyl)phenyl)imidazolidine-2,4-dithione

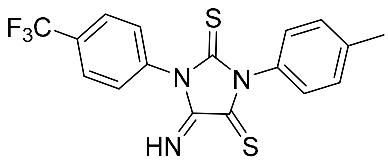

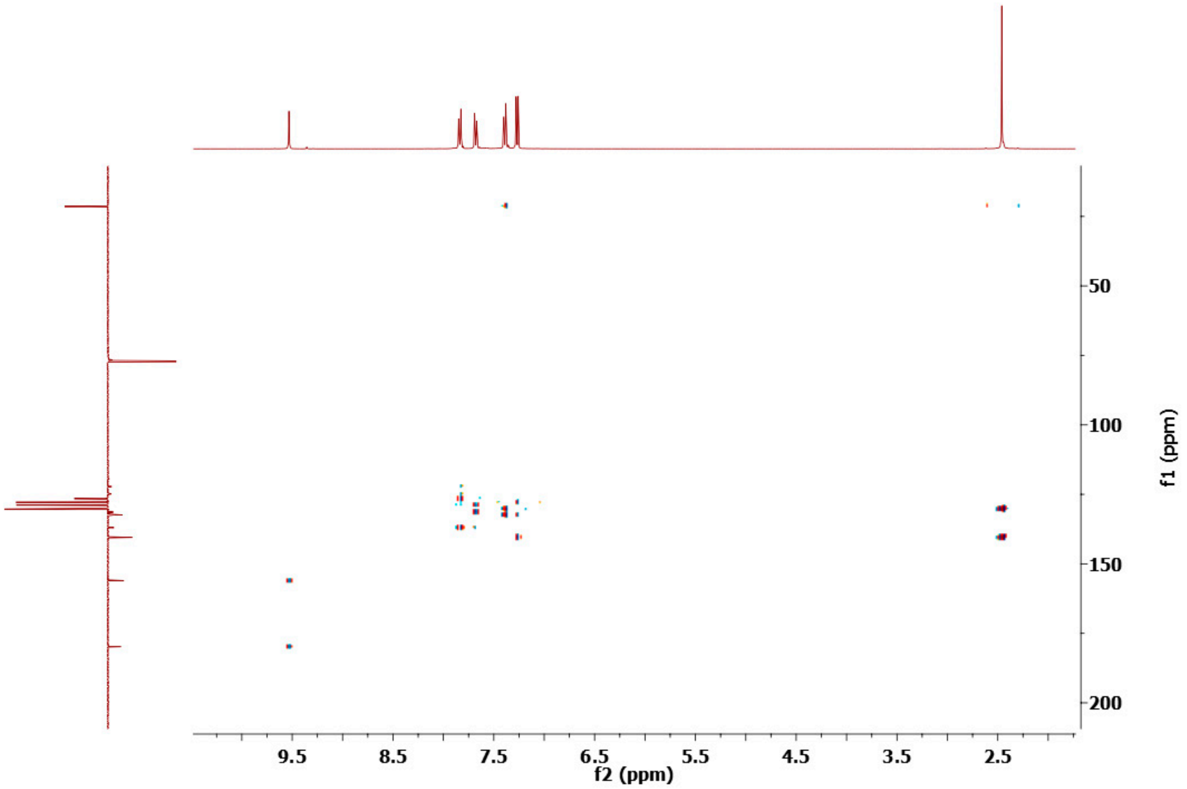

^{1}H NMR ($CDCl_3$) spectrum of 5-imino-1,3-di-p-tolylimidazolidine-2,4-dithione (18c')

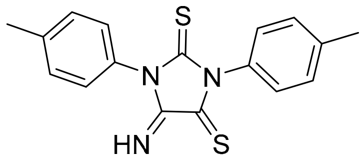

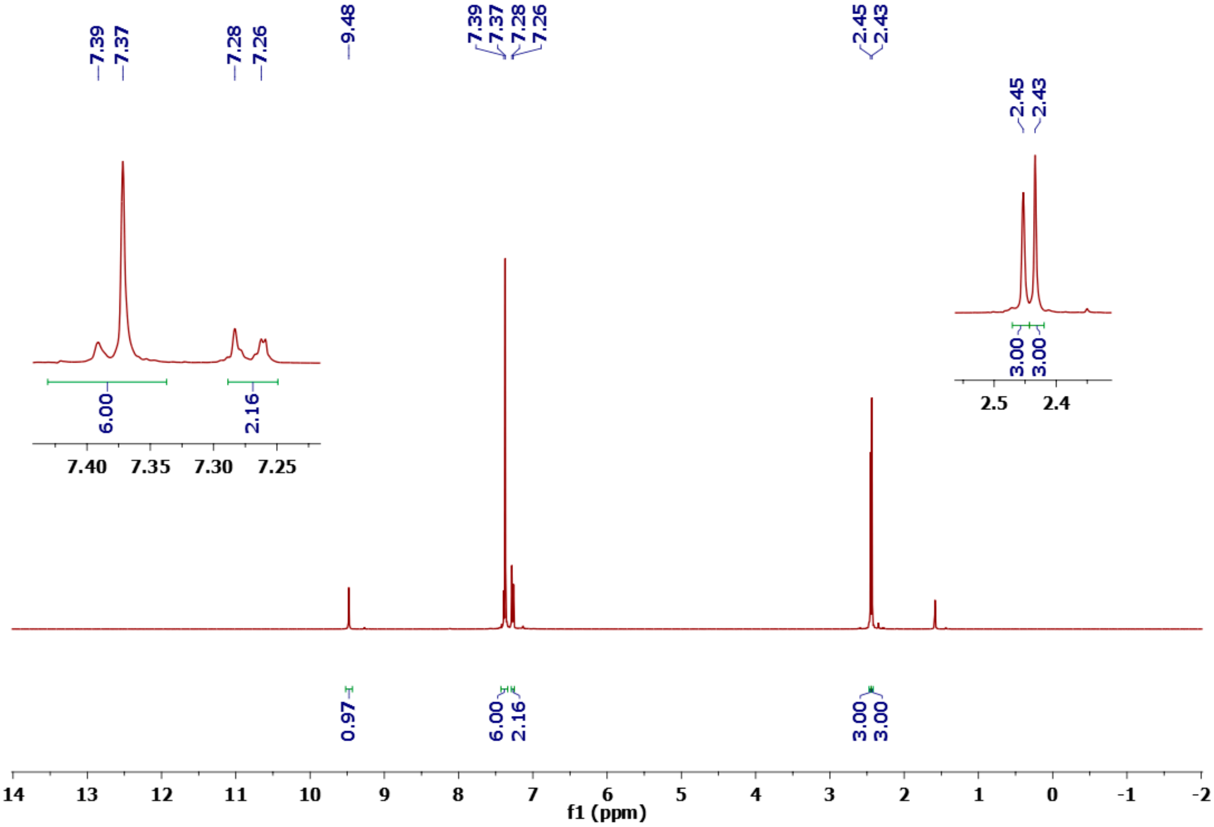

^{13}C NMR ($CDCl_3$) spectrum of 5-imino-1,3-di-p-tolylimidazolidine-2,4-dithione

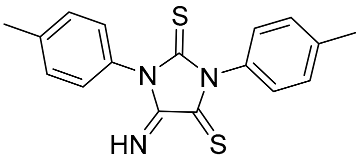

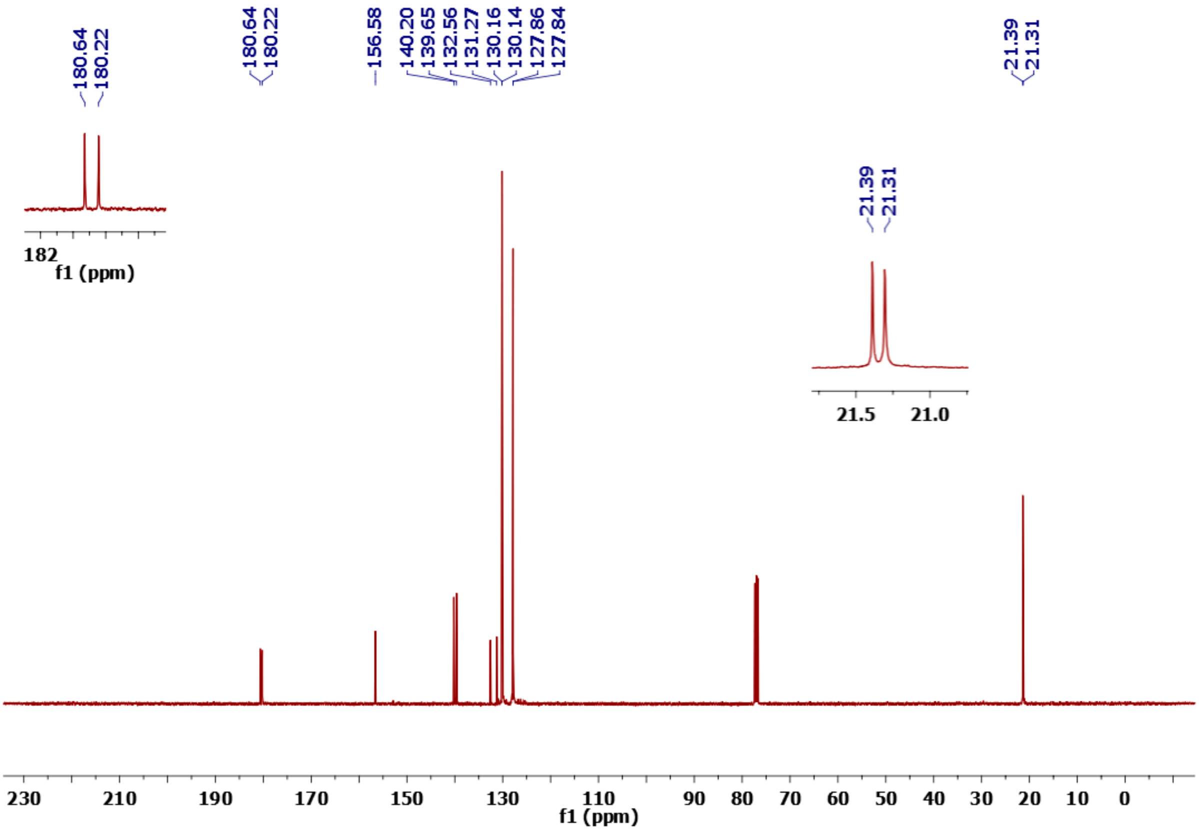

^{13}C-CRAPT NMR ($CDCl_3$) spectrum of 5-imino-1,3-di-p-tolylimidazolidine-2,4-dithione

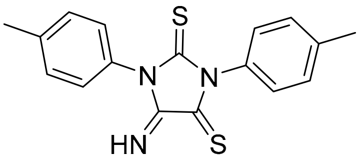

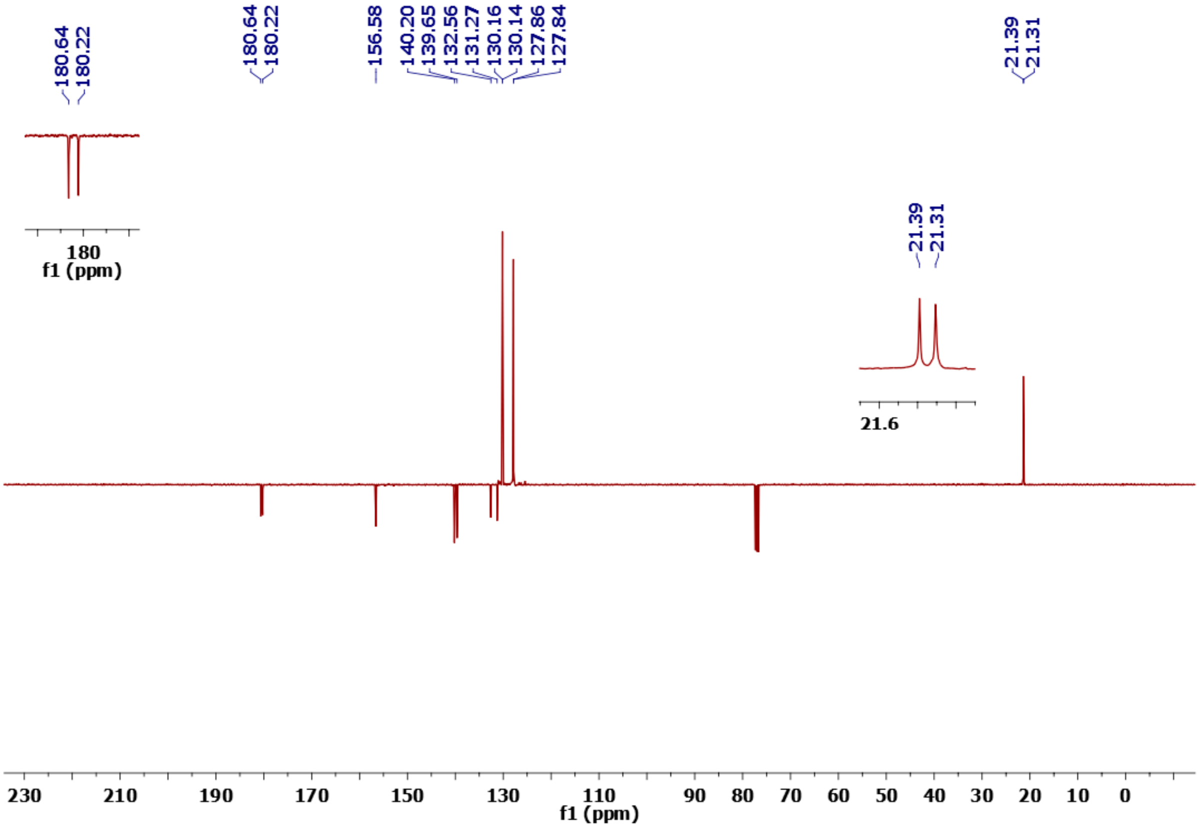

^{1}H-^{1}H-gCOSYAD NMR ($CDCl_3$) spectrum of 5-imino-1,3-di-p-tolylimidazolidine-2,4-dithione

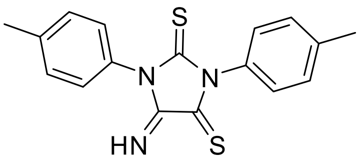

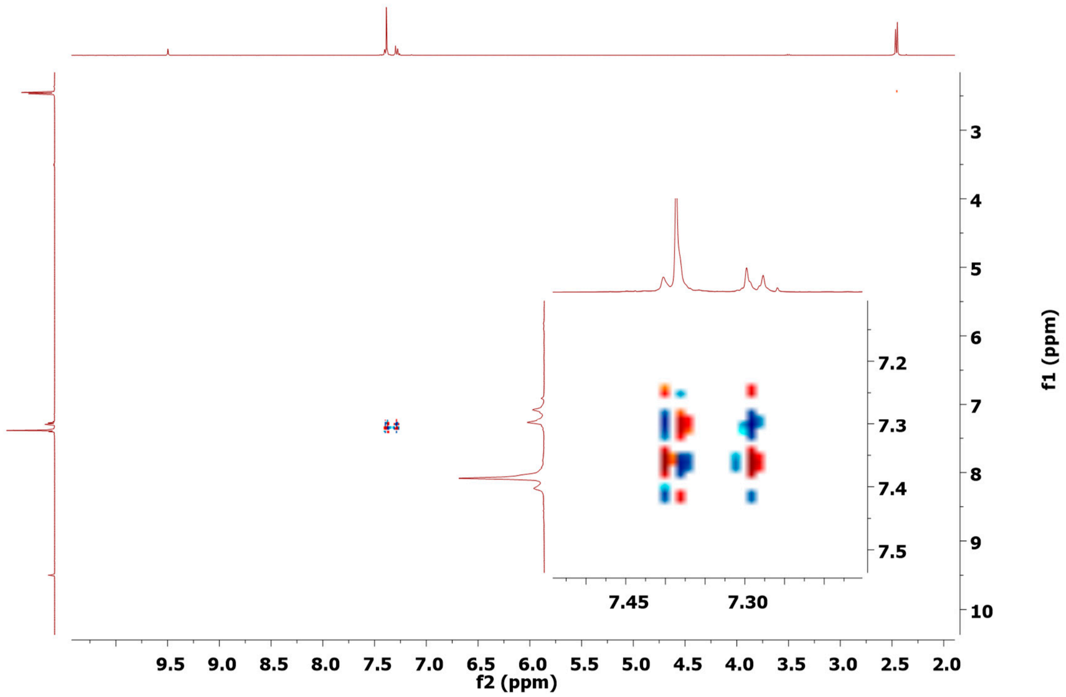

^{1}H-^{13}C-gHSQCAD ($CDCl_3$) spectrum of 5-imino-1,3-di-p-tolylimidazolidine-2,4-dithione

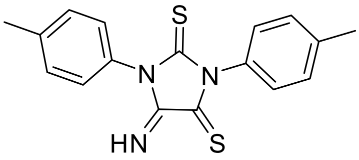

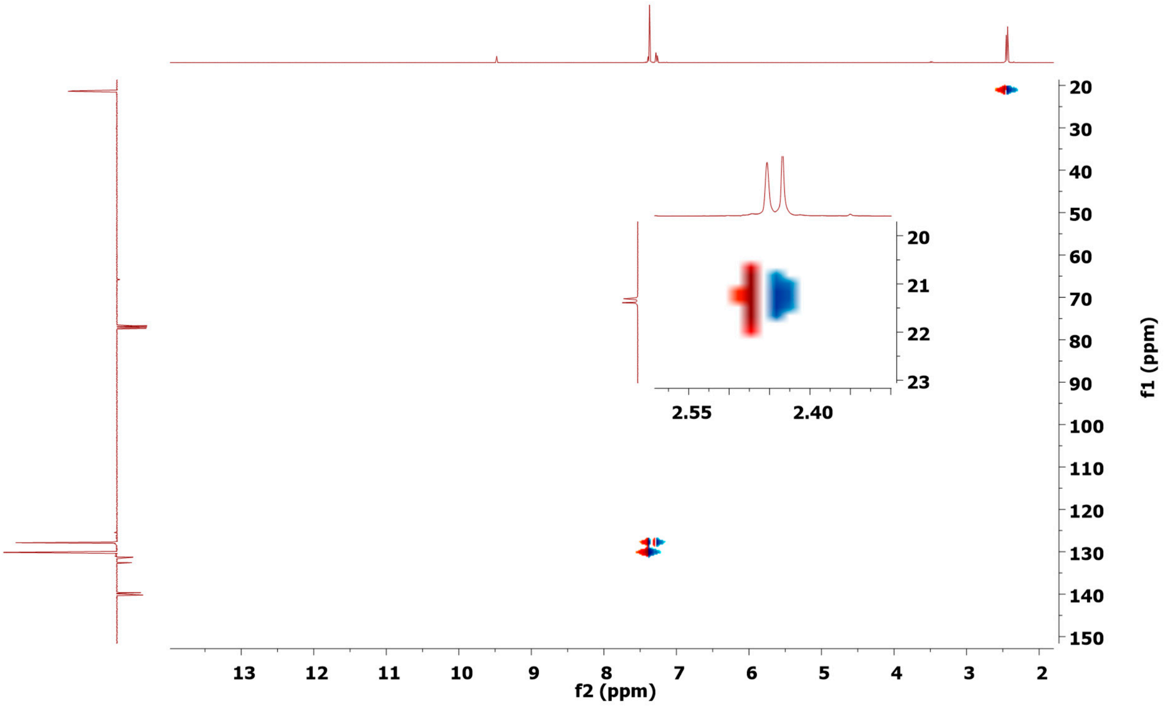

^1H-^{13}C-gHMBC ($CDCl_3$) spectrum of 5-imino-1,3-di-p-tolylimidazolidine-2,4-dithione

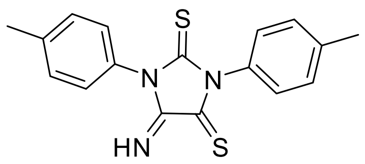

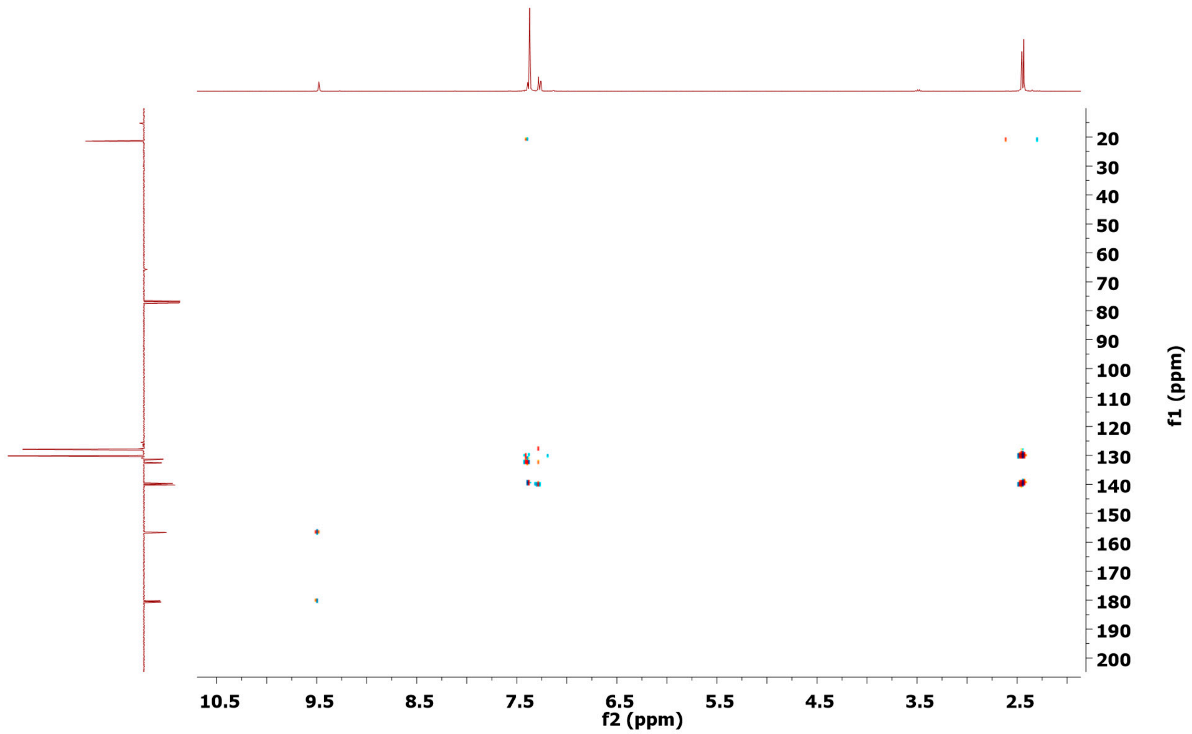

^{1}H NMR ($CDCl_3$) spectrum of 1-(2-fluorophenyl)-5-imino-3-(p-tolyl)imidazolidine-2,4-dithione (18d')

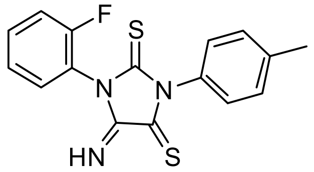

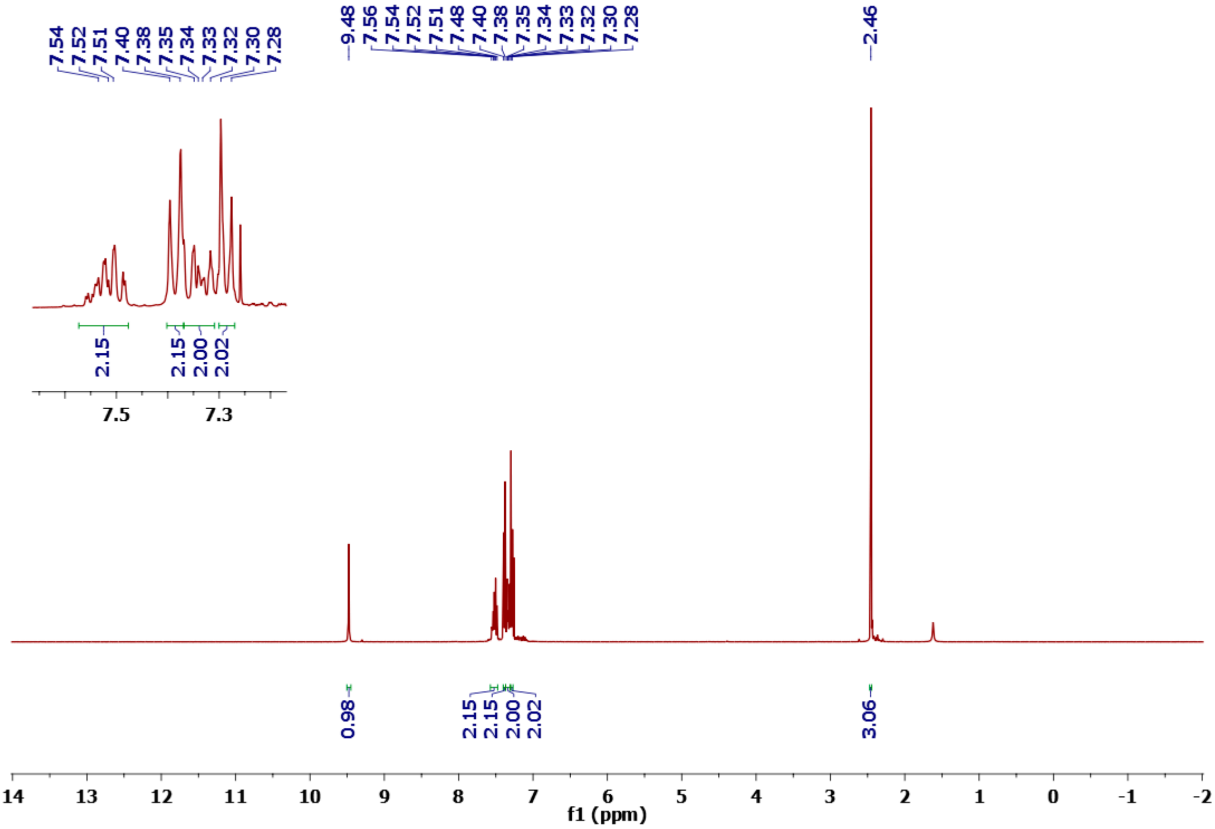

^{13}C NMR ($CDCl_3$) spectrum of 1-(2-fluorophenyl)-5-imino-3-(p-tolyl)imidazolidine-2,4-dithione

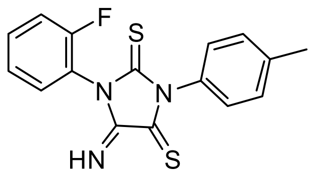

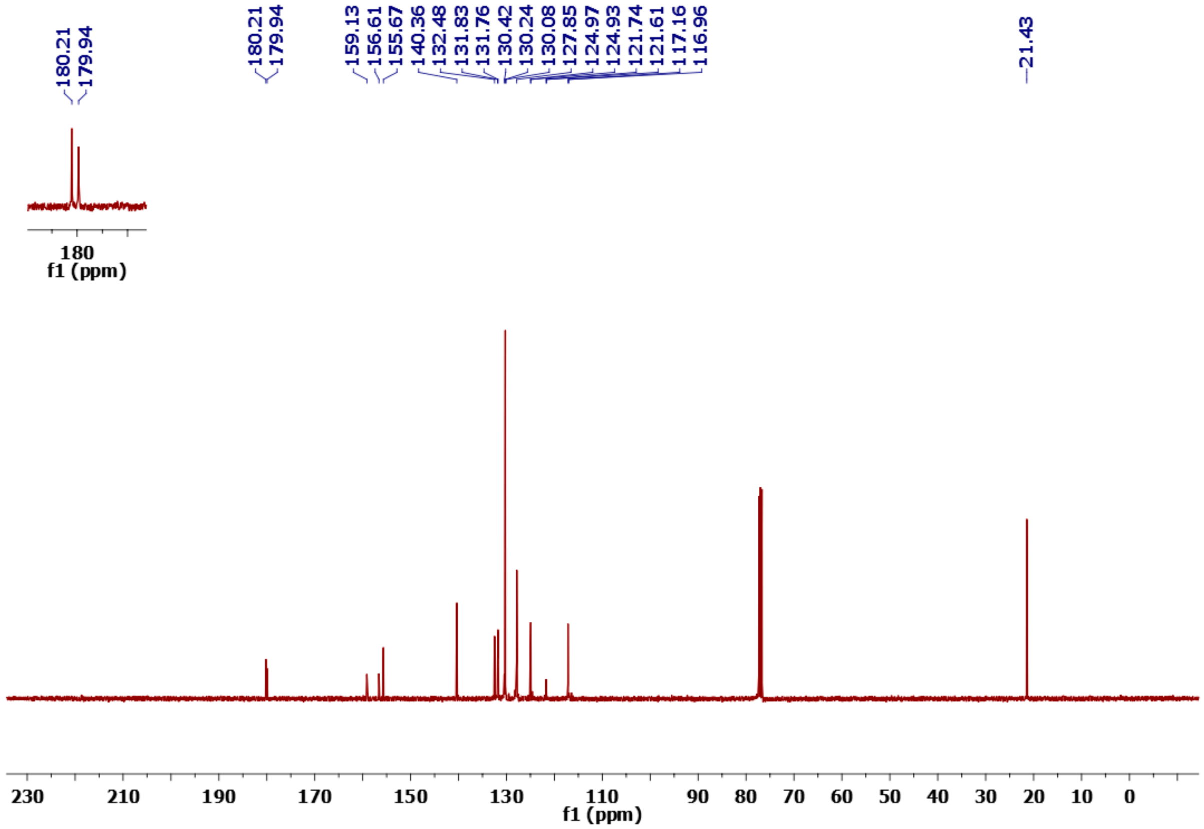

^{13}C-CRAPT NMR ($CDCl_3$) spectrum of 1-(2-fluorophenyl)-5-imino-3-(p-tolyl)imidazolidine-2,4-dithione

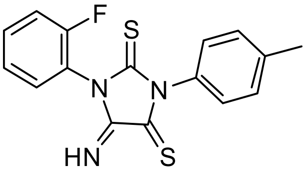

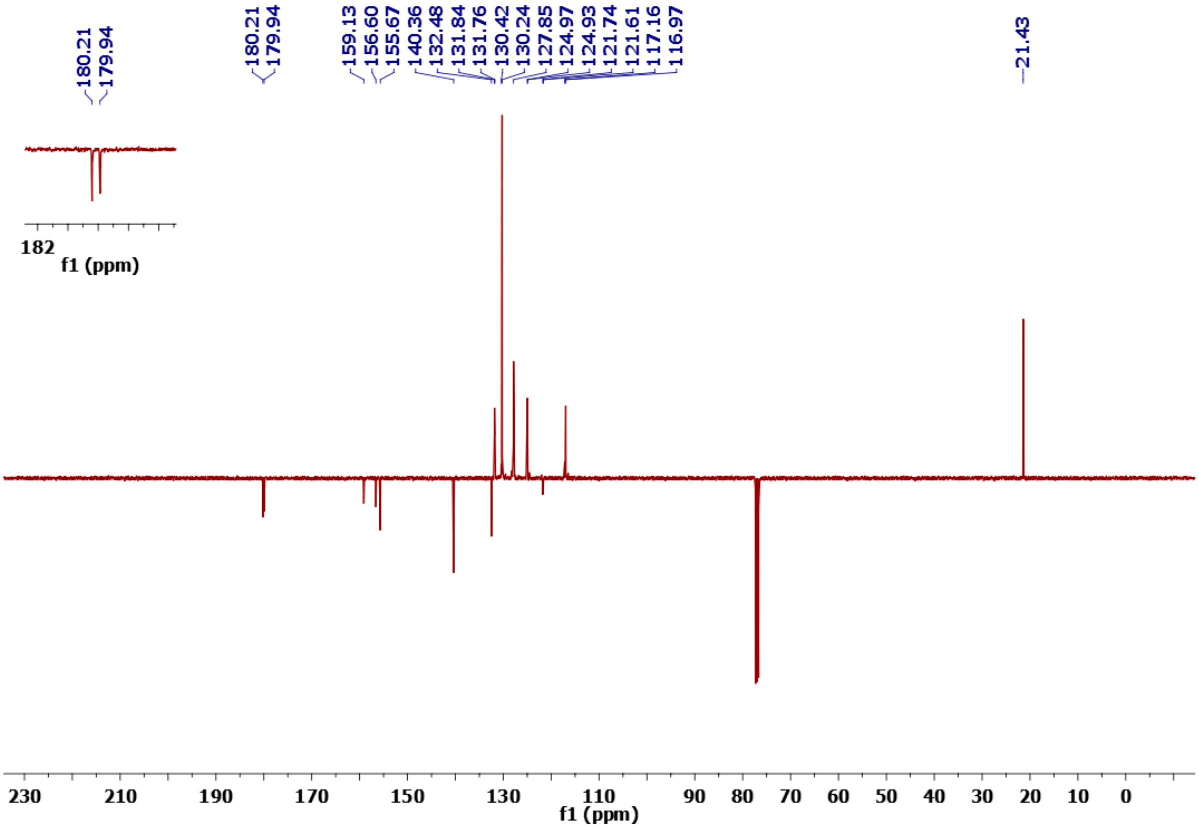

^{1}H-^{1}H-gCOSYAD NMR ($CDCl_3$) spectrum of 1-(2-fluorophenyl)-5-imino-3-(p-tolyl)imidazolidine-2,4-dithione

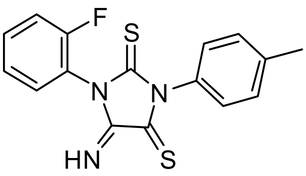

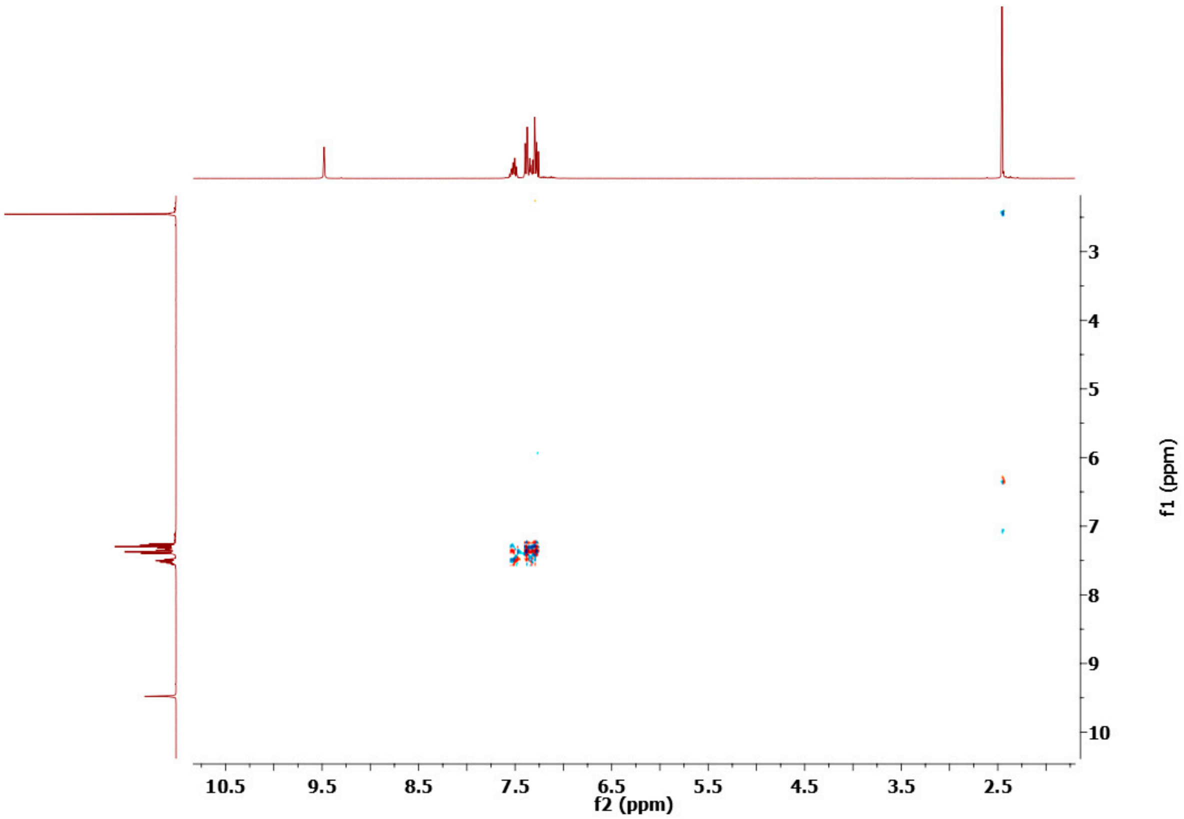

^{1}H-^{13}C-gHSQCAD NMR ($CDCl_3$) spectrum of 1-(2-fluorophenyl)-5-imino-3-(p-tolyl)imidazolidine-2,4-dithione

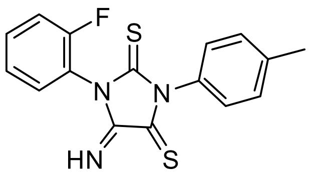

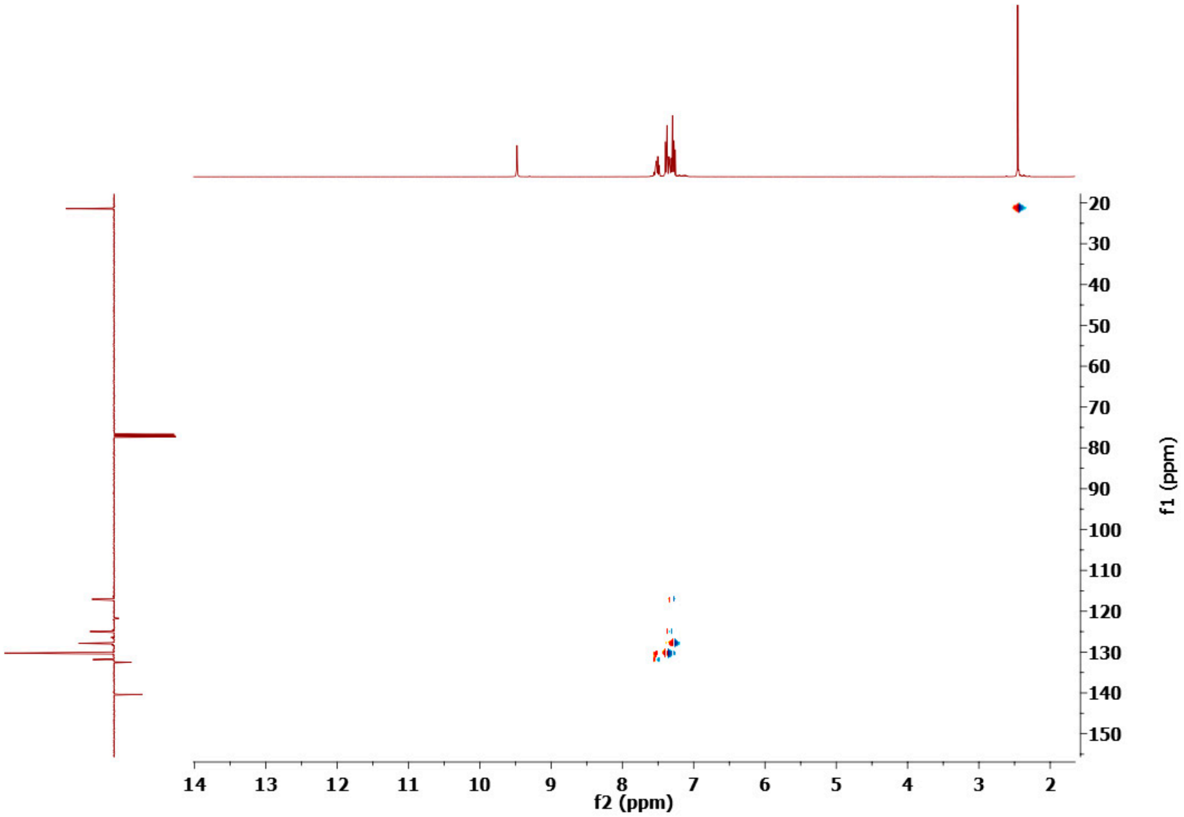

^{1}H-^{13}C-gHMBC NMR ($CDCl_3$) spectrum of 1-(2-fluorophenyl)-5-imino-3-(p-tolyl)imidazolidine-2,4-dithione

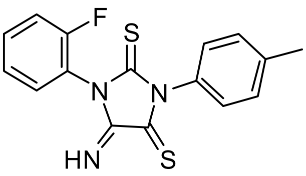

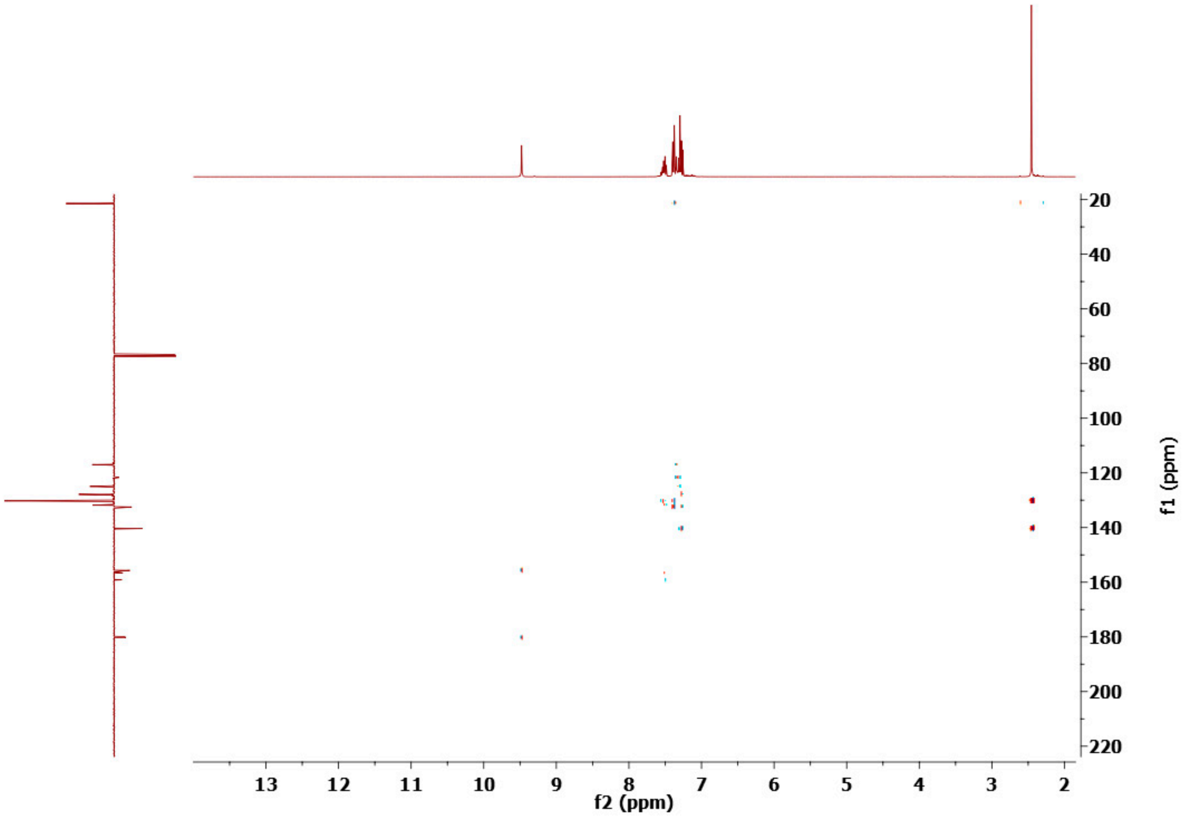

^{1}H NMR ($CDCl_3$) spectrum of 1-(4-chlorophenyl)-5-imino-3-(p-tolyl)imidazolidine-2,4-dithione (18e')

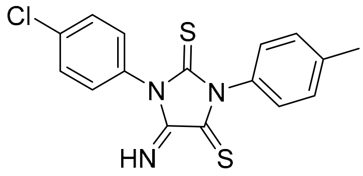

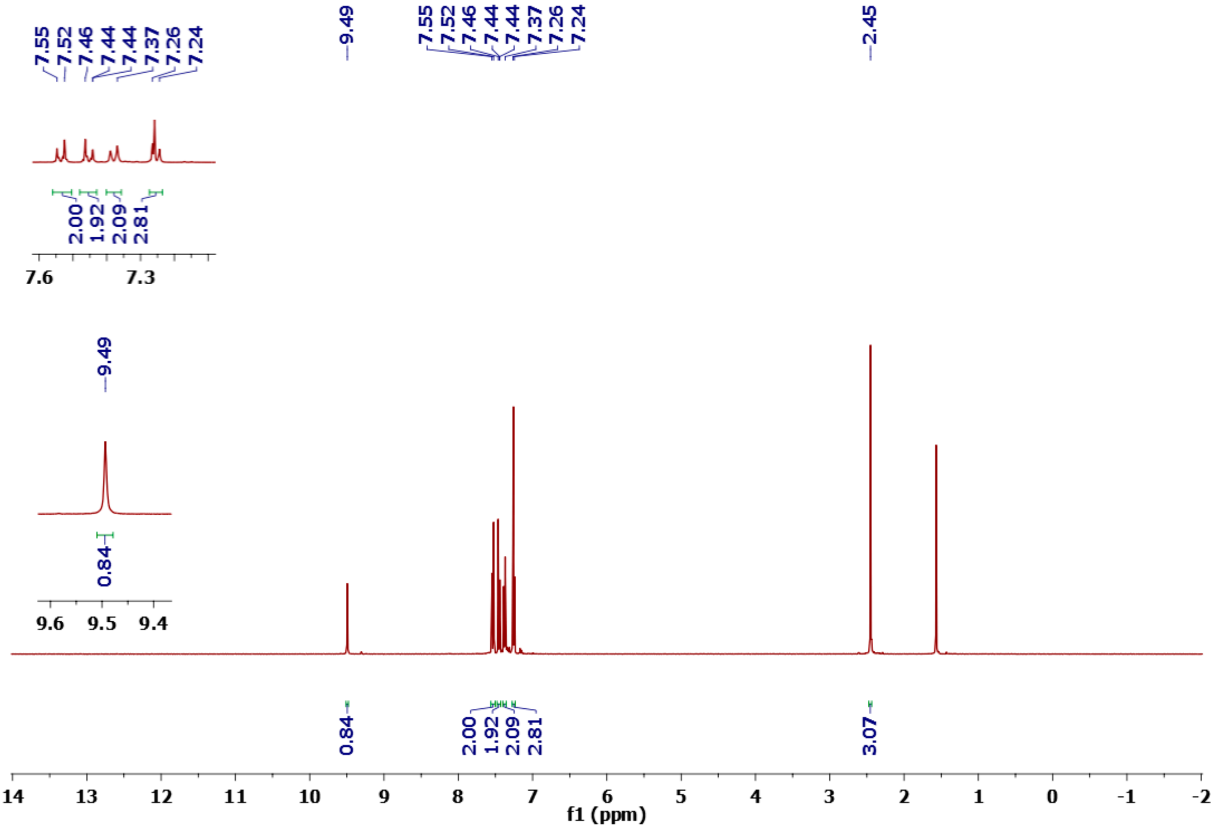

^{13}C NMR ($CDCl_3$) spectrum of 1-(4-chlorophenyl)-5-imino-3-(p-tolyl)imidazolidine-2,4-dithione

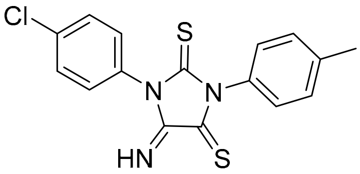

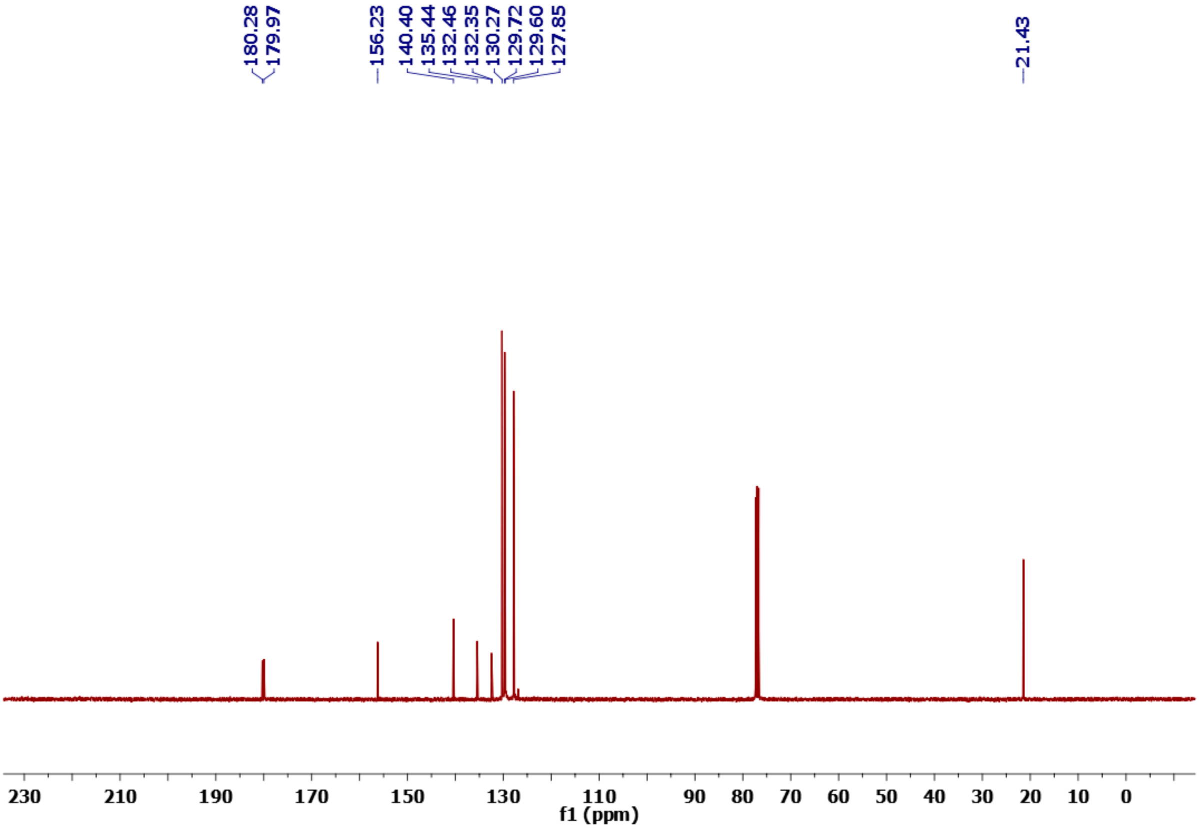

^{13}C-CRAPT NMR ($CDCl_3$) spectrum of 1-(4-chlorophenyl)-5-imino-3-(p-tolyl)imidazolidine-2,4-dithione

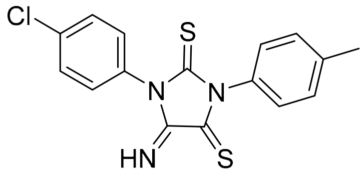

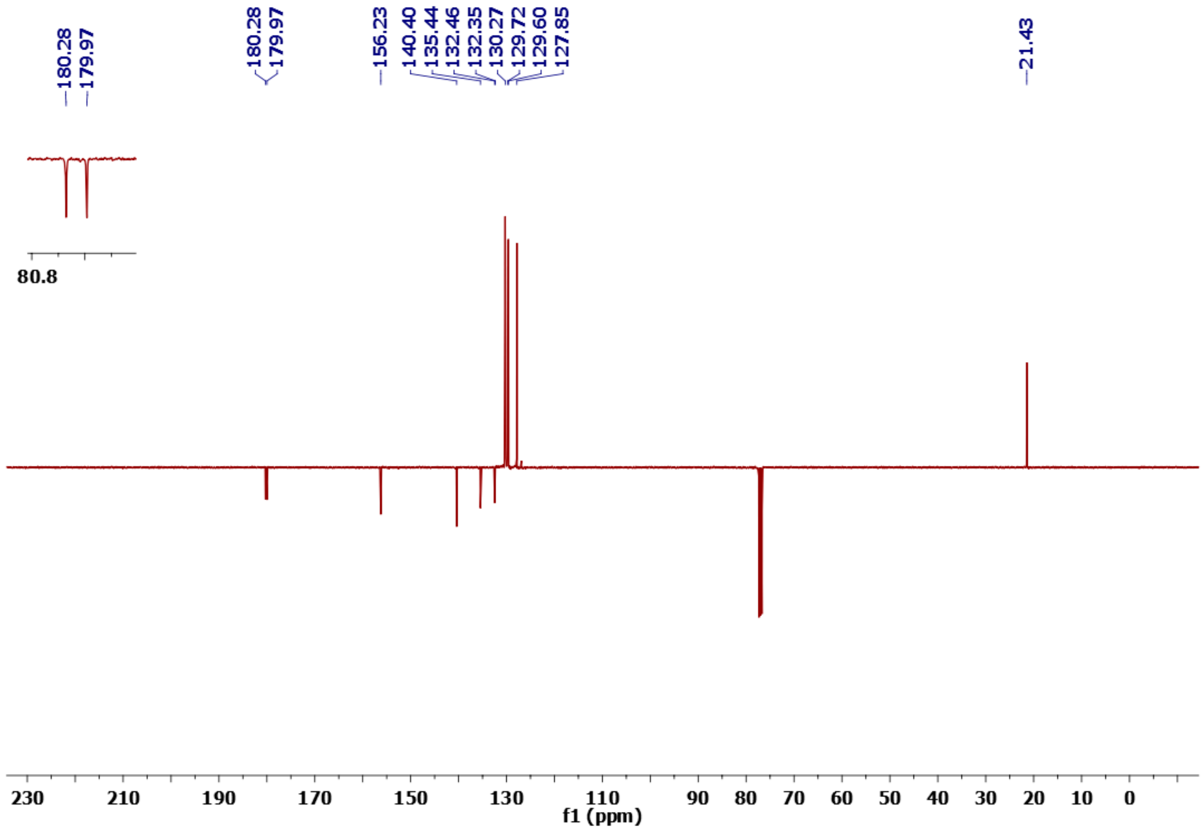

^{1}H-^{1}H-gCOSYAD NMR ($CDCl_3$) spectrum of 1-(4-chlorophenyl)-5-imino-3-(p-tolyl)imidazolidine-2,4-dithione

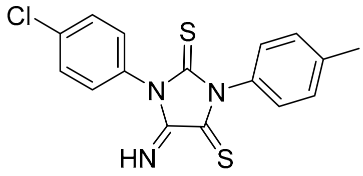

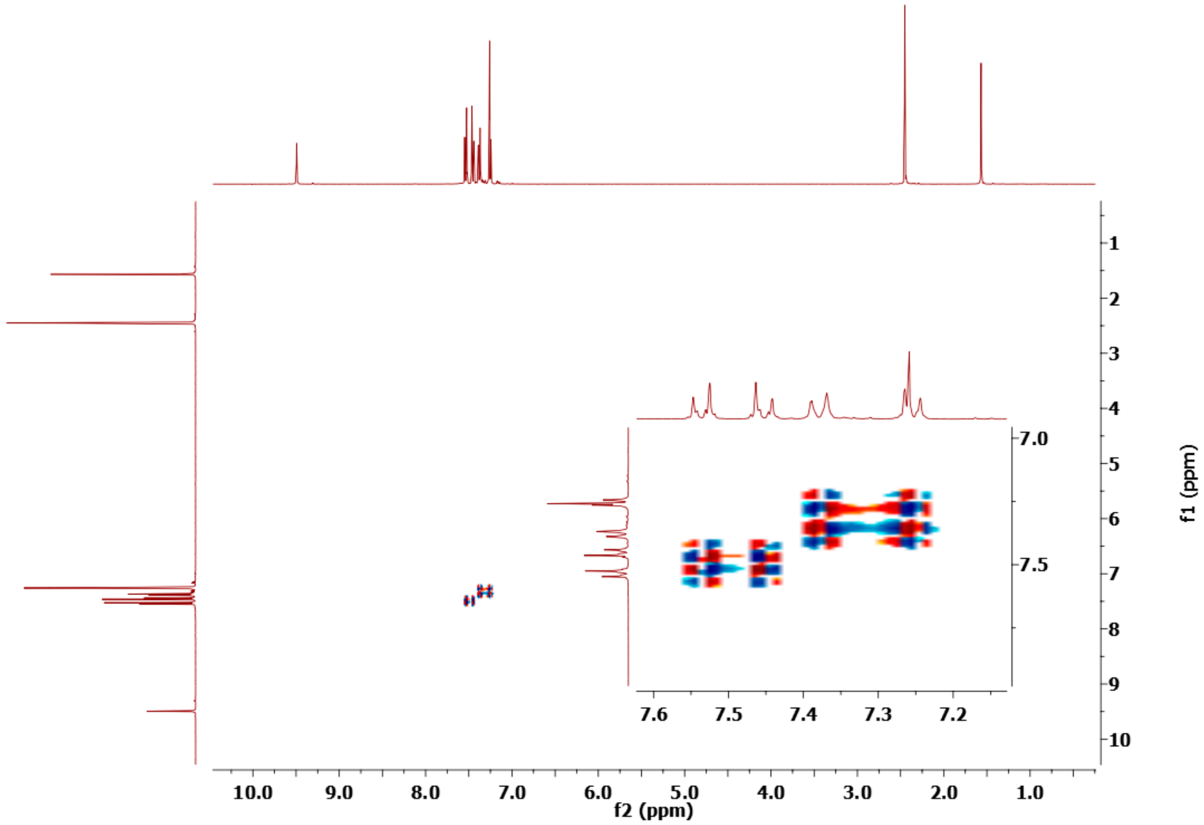

^{1}H-^{13}C-gHSQCAD NMR ($CDCl_3$) spectrum of 1-(4-chlorophenyl)-5-imino-3-(p-tolyl)imidazolidine-2,4-dithione

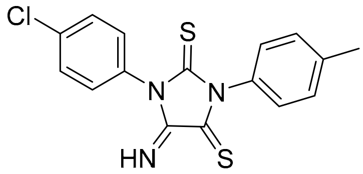

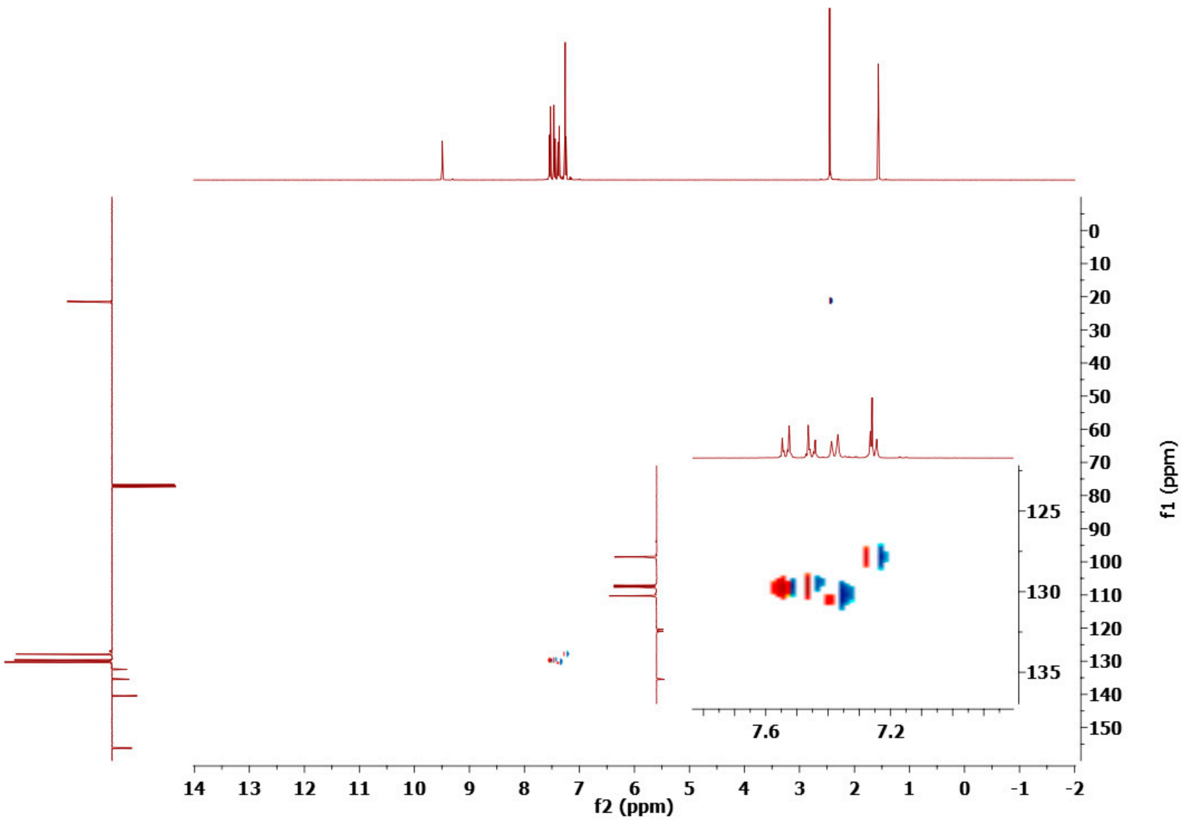

^{1}H-^{13}C-gHMBC NMR ($CDCl_3$) spectrum of 1-(4-chlorophenyl)-5-imino-3-(p-tolyl)imidazolidine-2,4-dithione

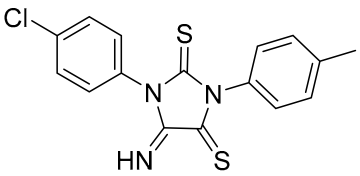

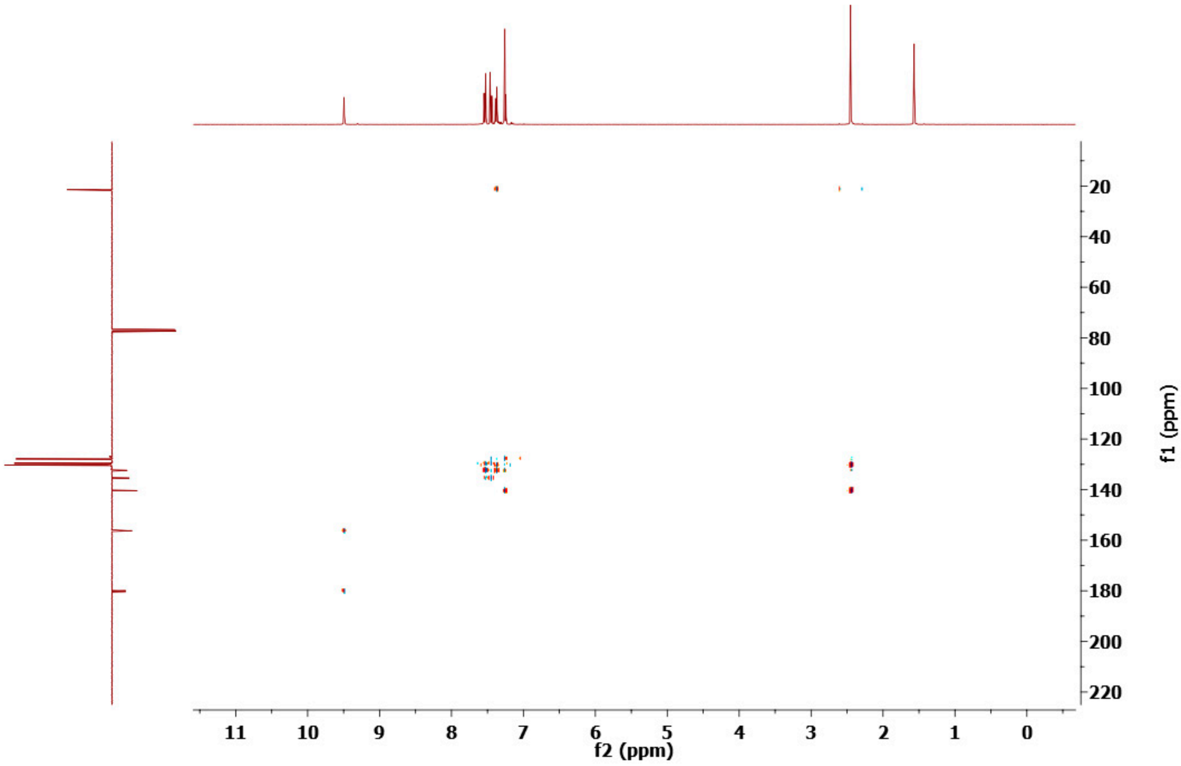

^{1}H NMR ($CDCl_3$) spectrum of 1-(4-fluorophenyl)-5-imino-3-(p-tolyl)imidazolidine-2,4-dithione (18f')

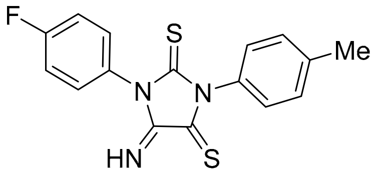

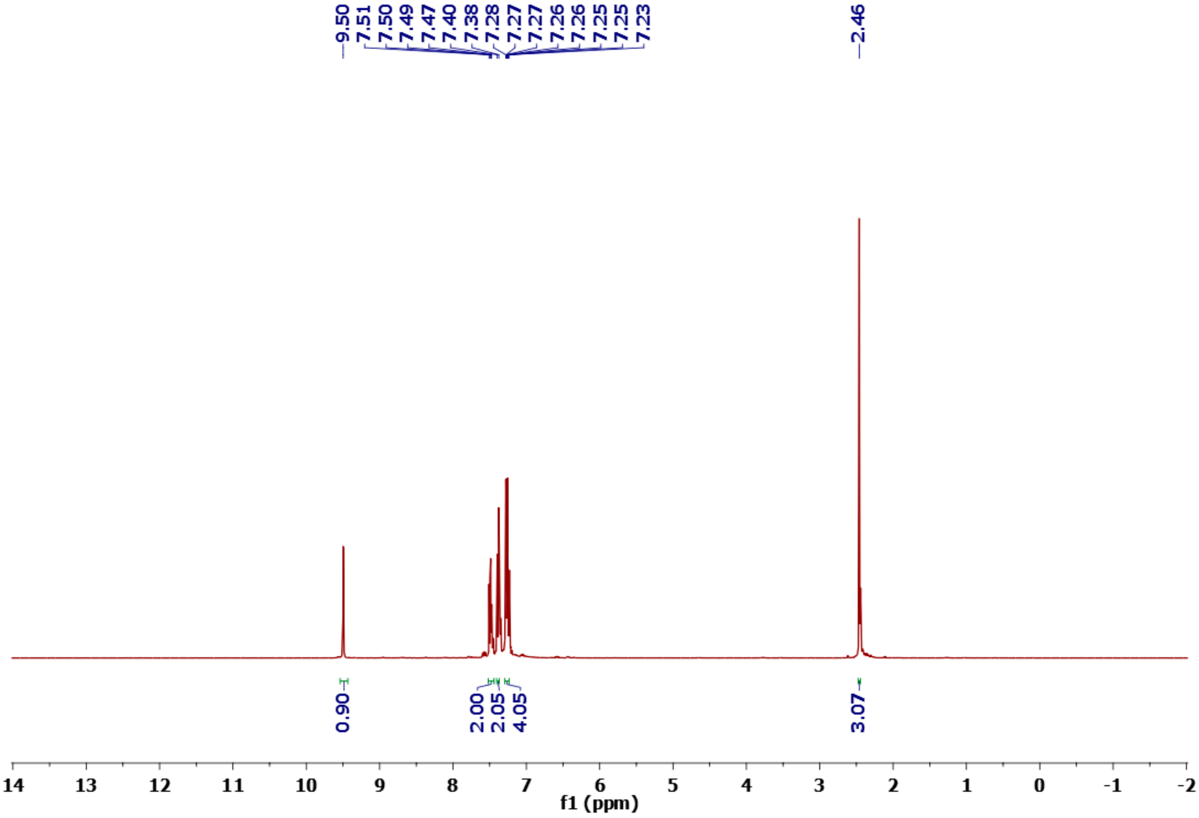

^{13}C NMR ($CDCl_3$) spectrum of 1-(4-fluorophenyl)-5-imino-3-(p-tolyl)imidazolidine-2,4-dithione

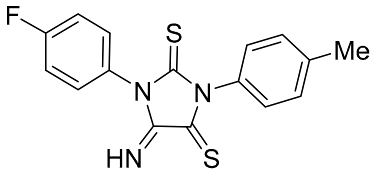

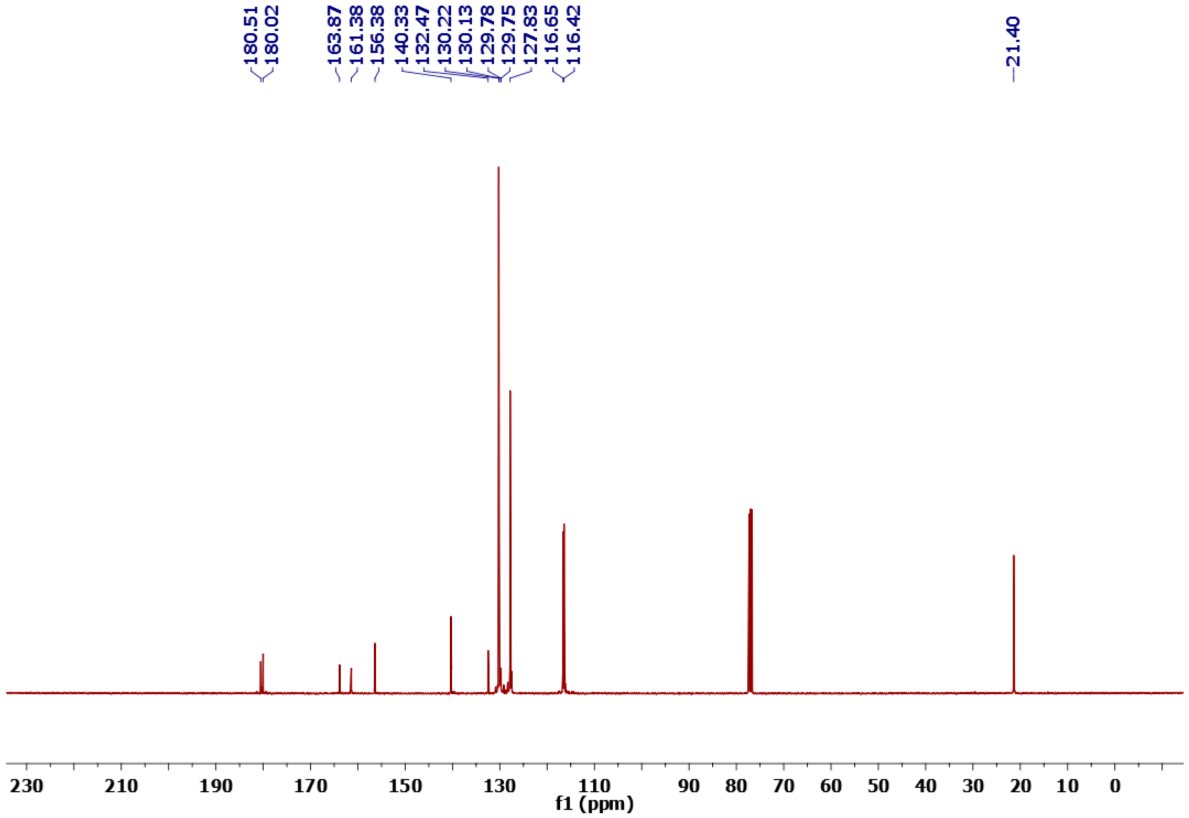

^{13}C-CRAPT NMR ($CDCl_3$) spectrum of 1-(4-fluorophenyl)-5-imino-3-(p-tolyl)imidazolidine-2,4-dithione

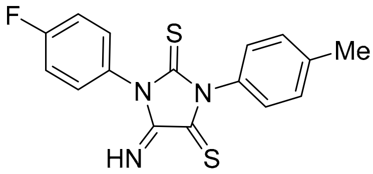

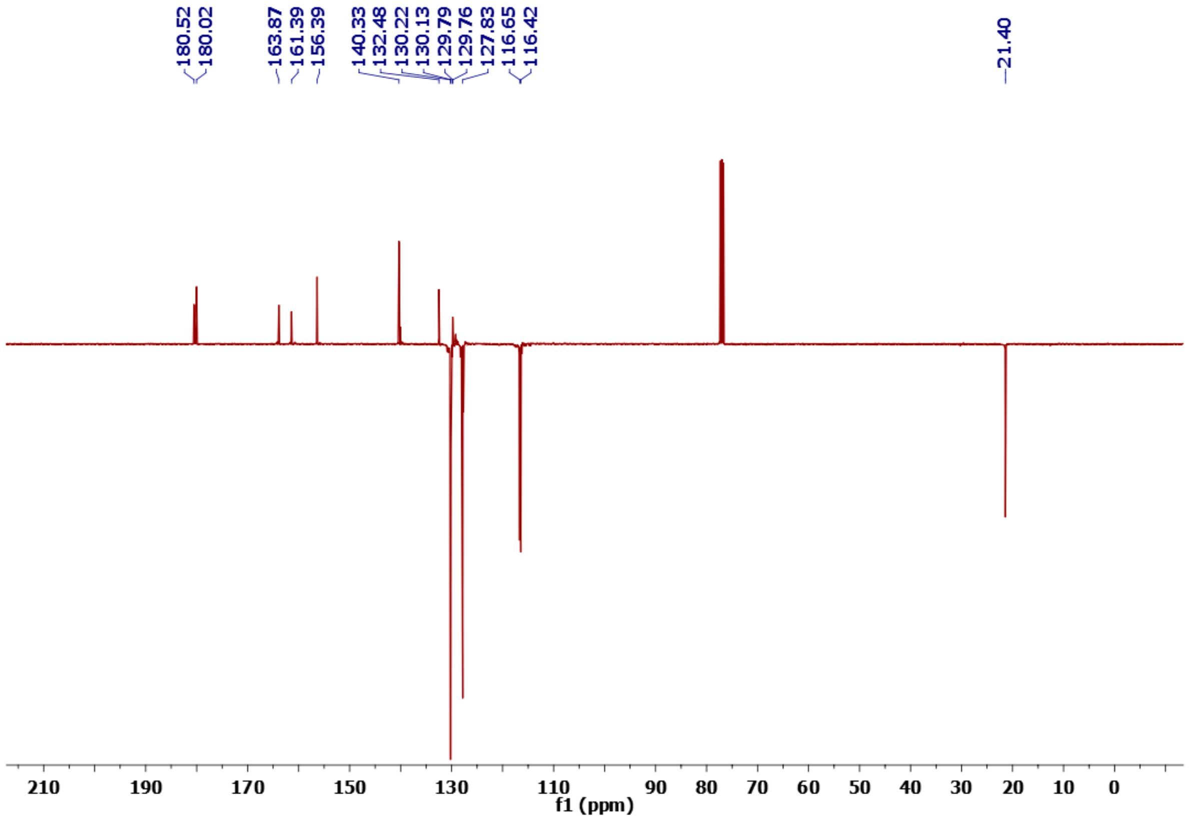

^{1}H-^{1}H-gCOSYAD NMR ($CDCl_3$) spectrum of 1-(4-fluorophenyl)-5-imino-3-(p-tolyl)imidazolidine-2,4-dithione

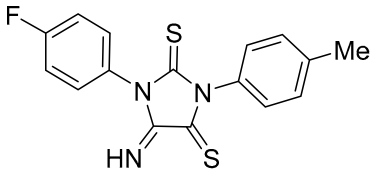

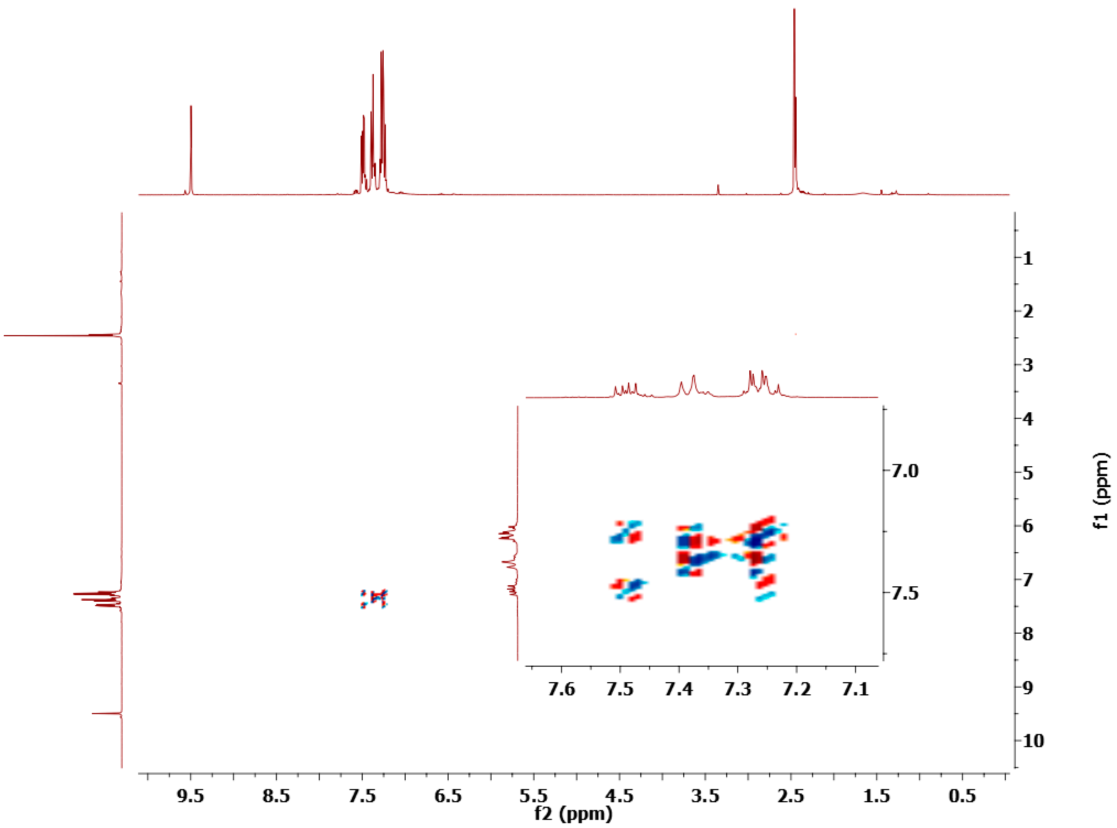

^{1}H-^{13}C-gHSQCAD NMR ($CDCl_3$) spectrum of 1-(4-fluorophenyl)-5-imino-3-(p-tolyl)imidazolidine-2,4-dithione

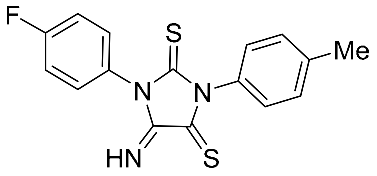

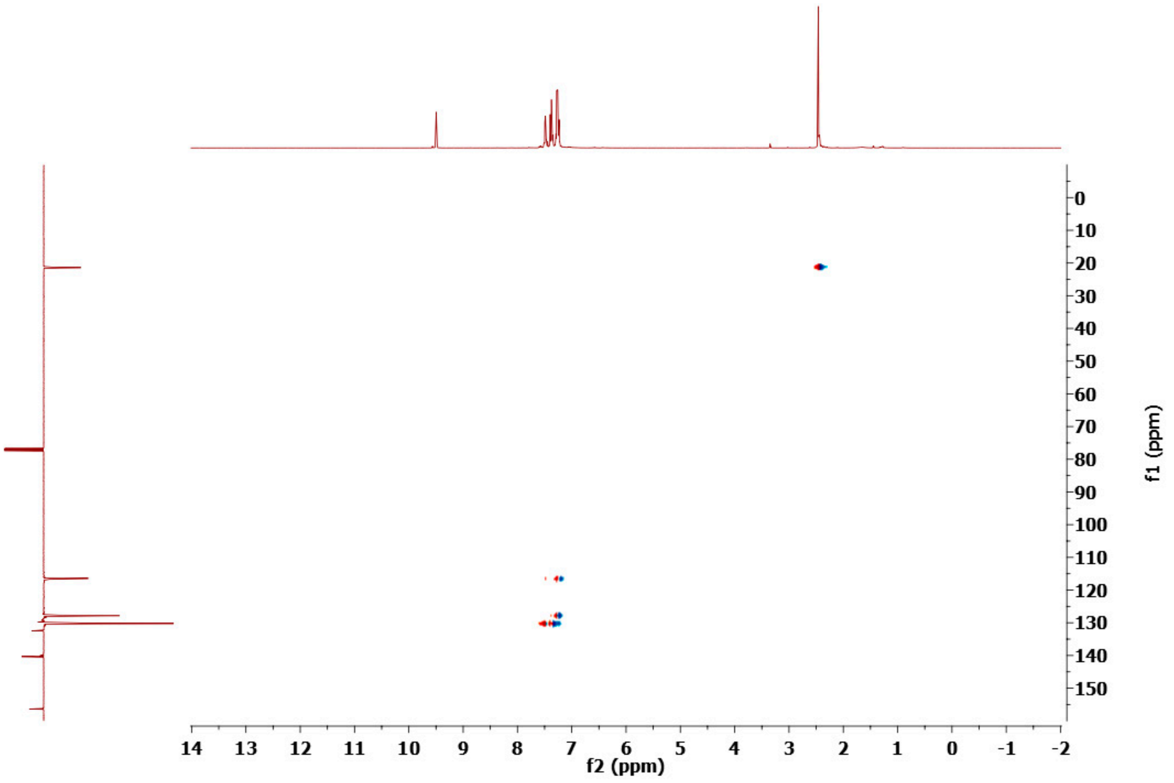

^{1}H-^{13}C-gHMBC NMR ($CDCl_3$) spectrum of 1-(4-fluorophenyl)-5-imino-3-(p-tolyl)imidazolidine-2,4-dithione

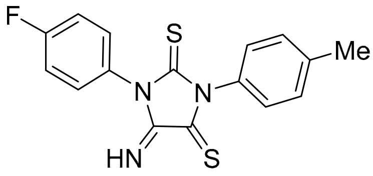

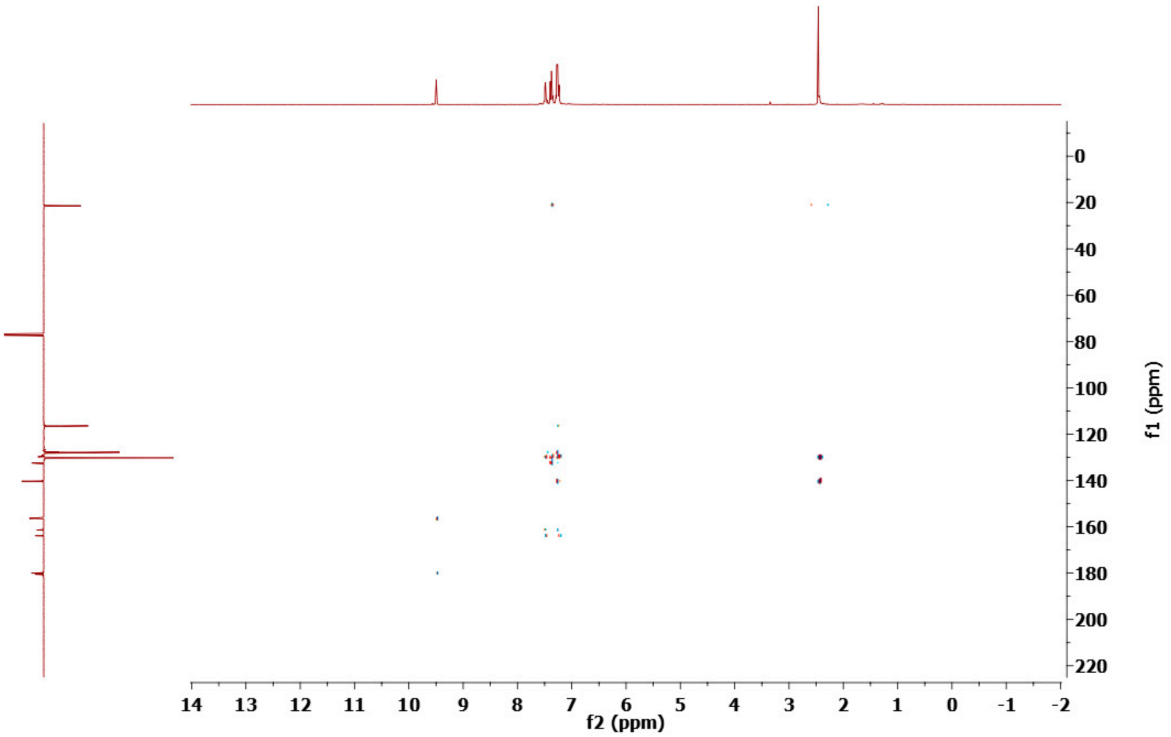

^{1}H-NMR ($CDCl_3$) spectrum of 5-imino-1-(4-nitrophenyl)-3-(p-tolyl)imidazolidine-2,4-dithione (18g')

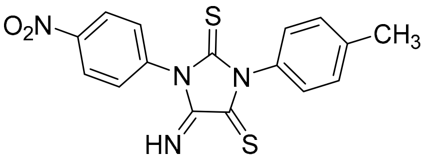

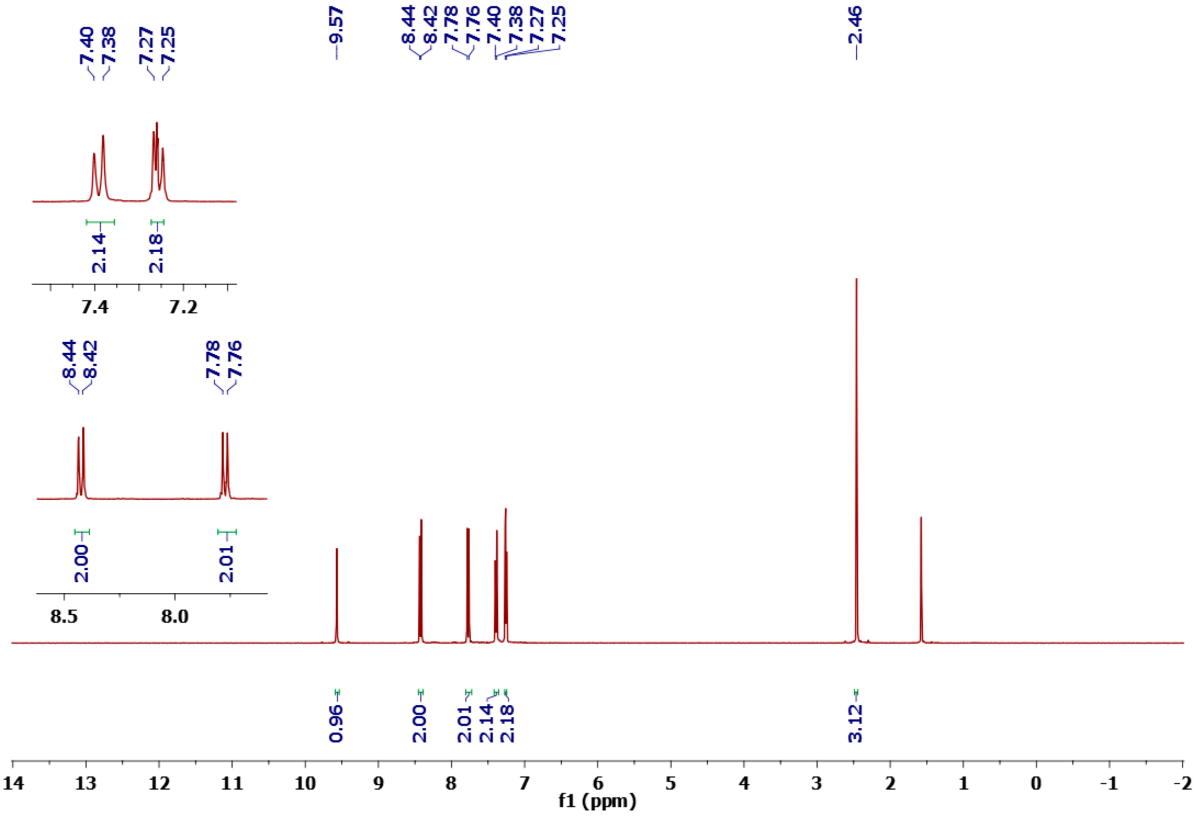

^{13}C NMR ($CDCl_3$) spectrum of 5-imino-1-(4-nitrophenyl)-3-(p-tolyl)imidazolidine-2,4-dithione

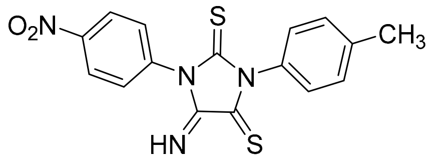

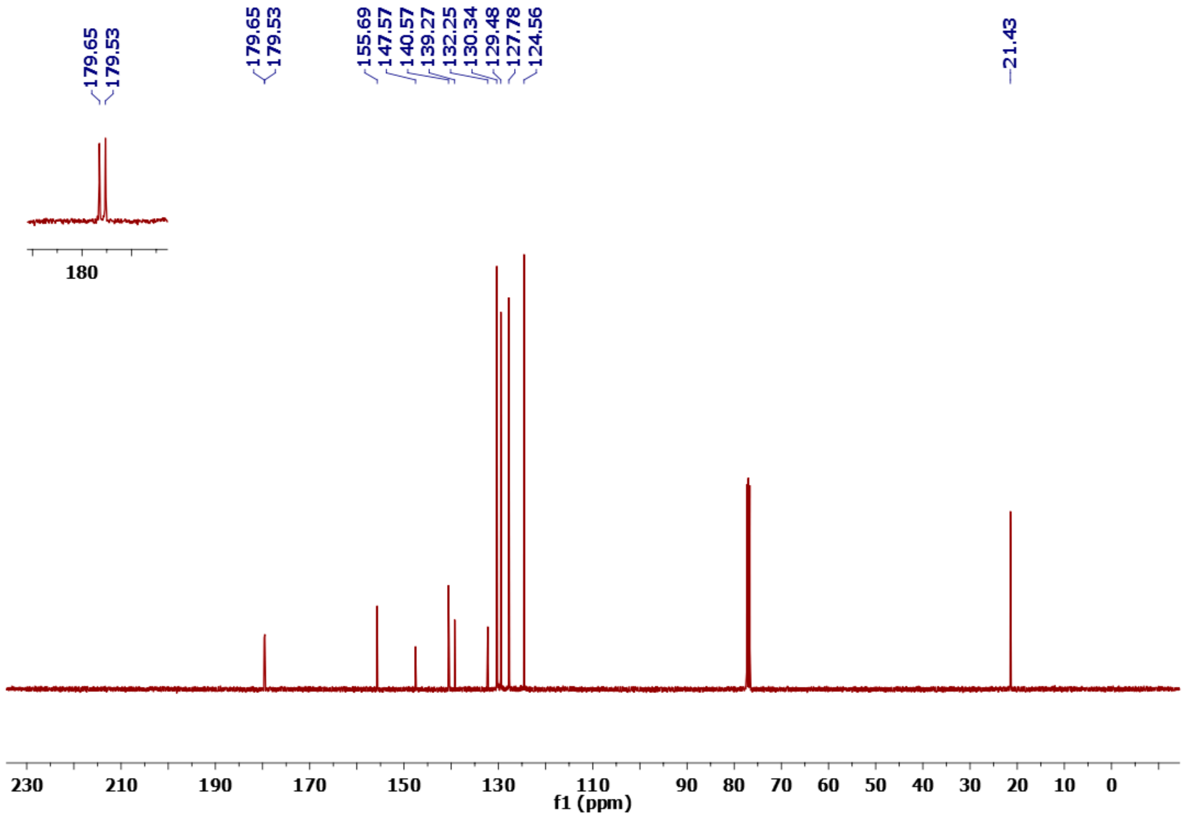

^{13}C-CRAPT NMR ($CDCl_3$) spectrum of 5-imino-1-(4-nitrophenyl)-3-(p-tolyl)imidazolidine-2,4-dithione

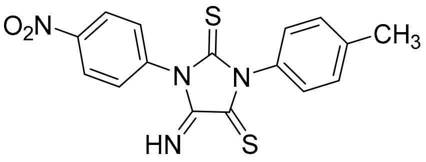

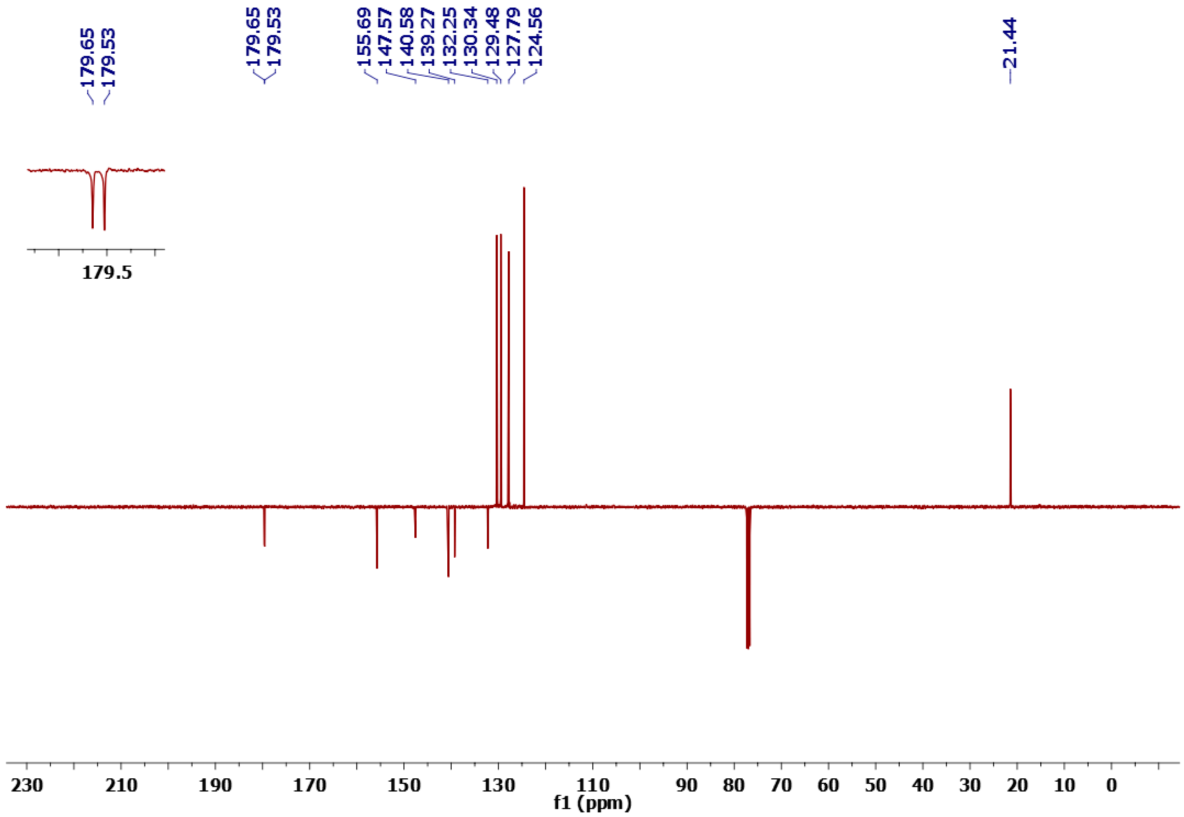

^{1}H-^{1}H-gCOSYAD NMR ($CDCl_3$) spectrum of 5-imino-1-(4-nitrophenyl)-3-(p-tolyl)imidazolidine-2,4-dithione

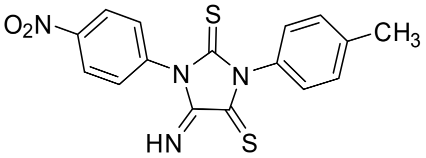

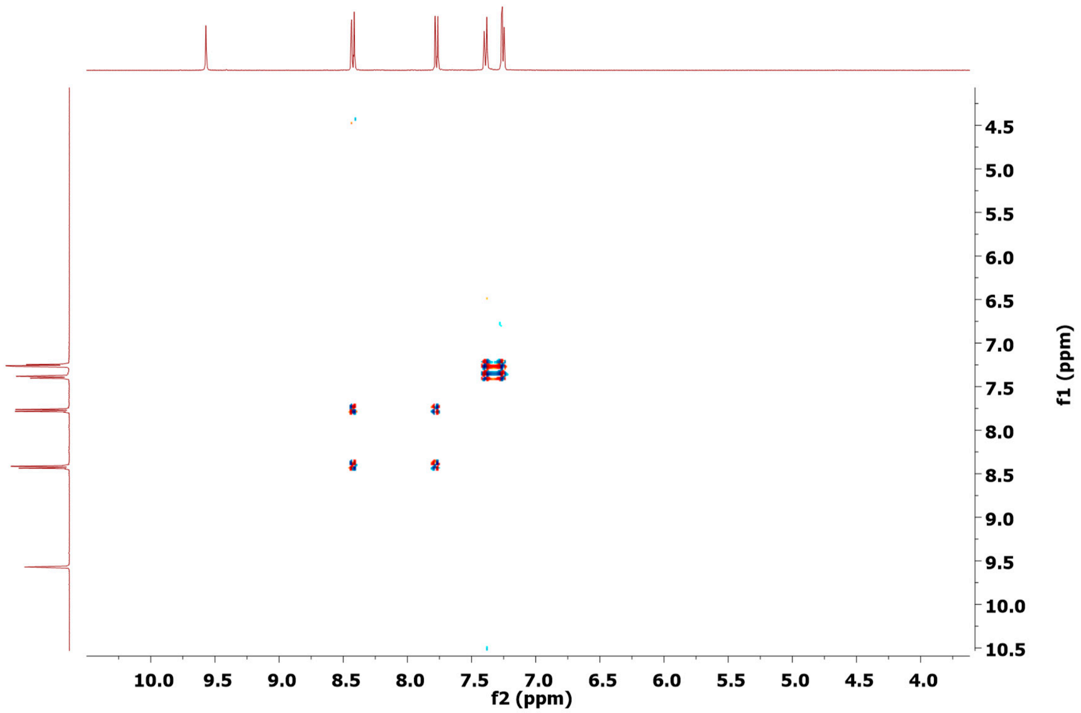

^{1}H-^{13}C-gHSQCAD NMR ($CDCl_3$) spectrum of 5-imino-1-(4-nitrophenyl)-3-(p-tolyl)imidazolidine-2,4-dithione

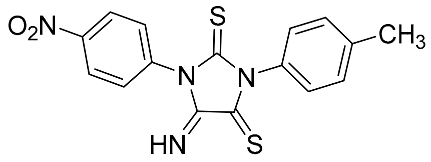

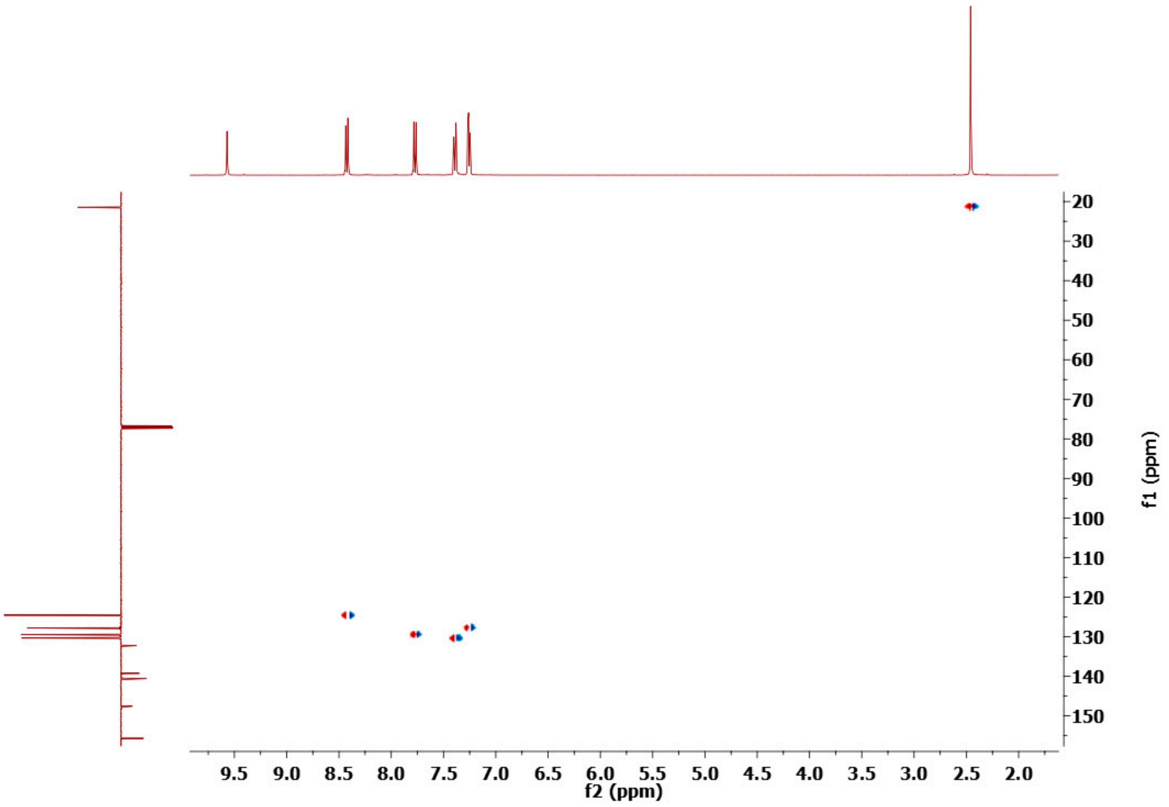

^{1}H-^{13}C-HMBC NMR ($CDCl_3$) spectrum of 5-imino-1-(4-nitrophenyl)-3-(p-tolyl)imidazolidine-2,4-dithione

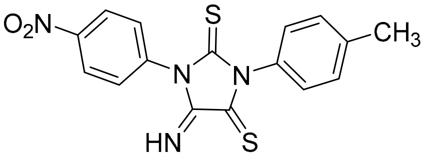

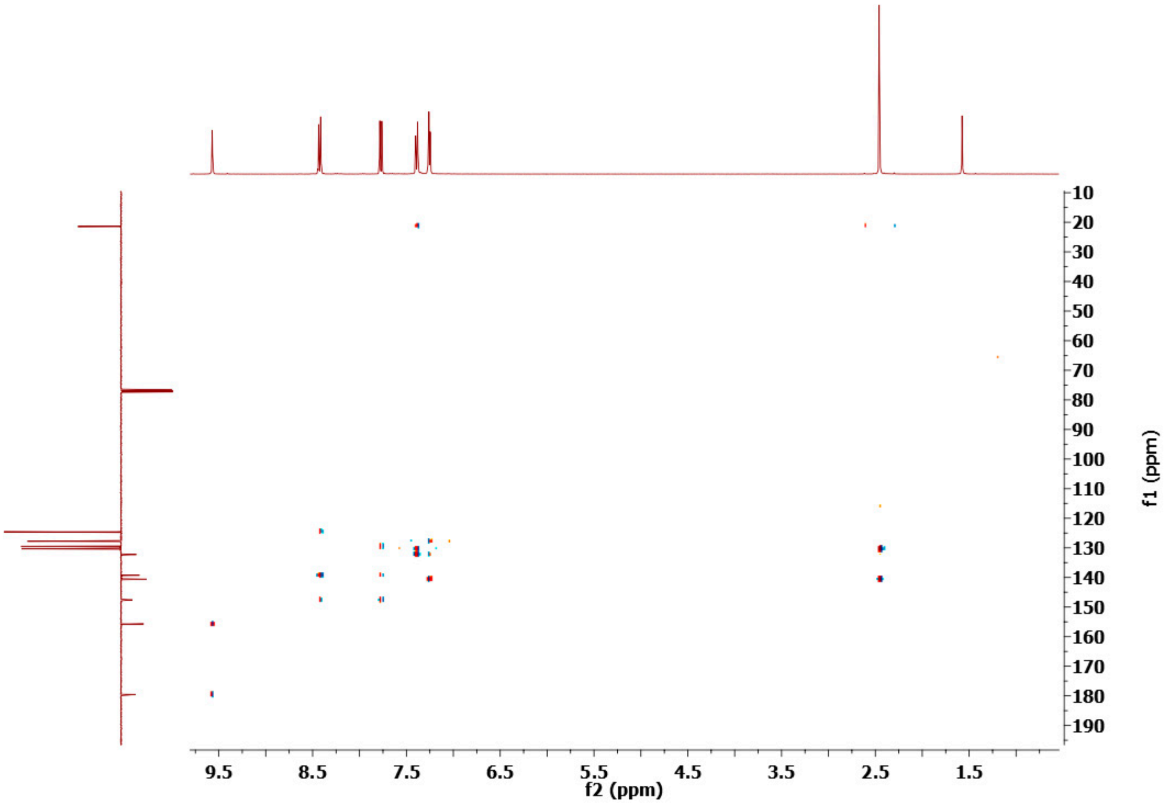

^{1}H NMR ($CDCl_3$) spectrum of 5-imino-1-(4-methoxyphenyl)-3-(p-tolyl)imidazolidine-2,4-dithione (18h')

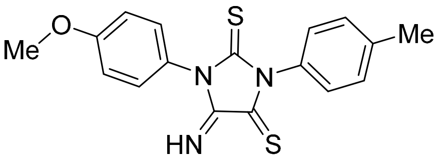

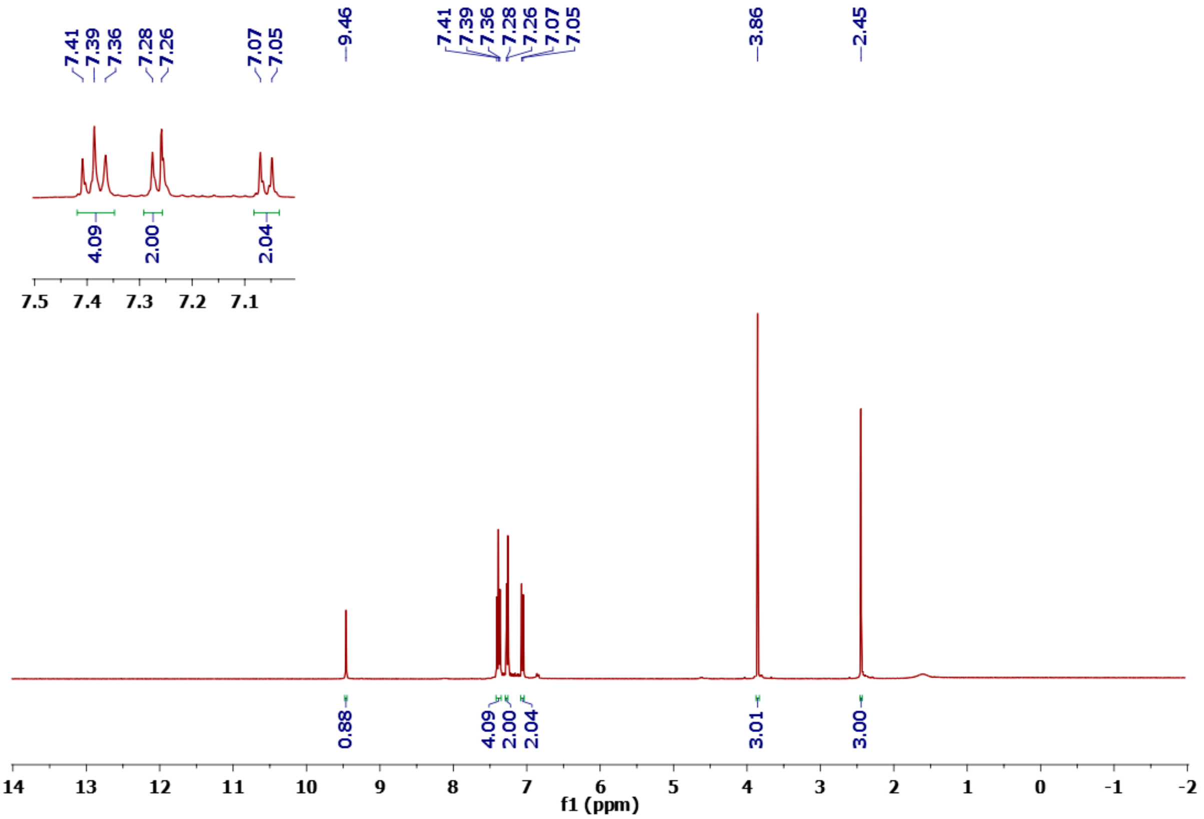

^{13}C NMR ($CDCl_3$) spectrum of 5-imino-1-(4-methoxyphenyl)-3-(p-tolyl)imidazolidine-2,4-dithione

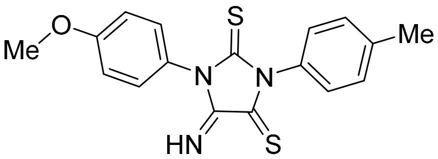

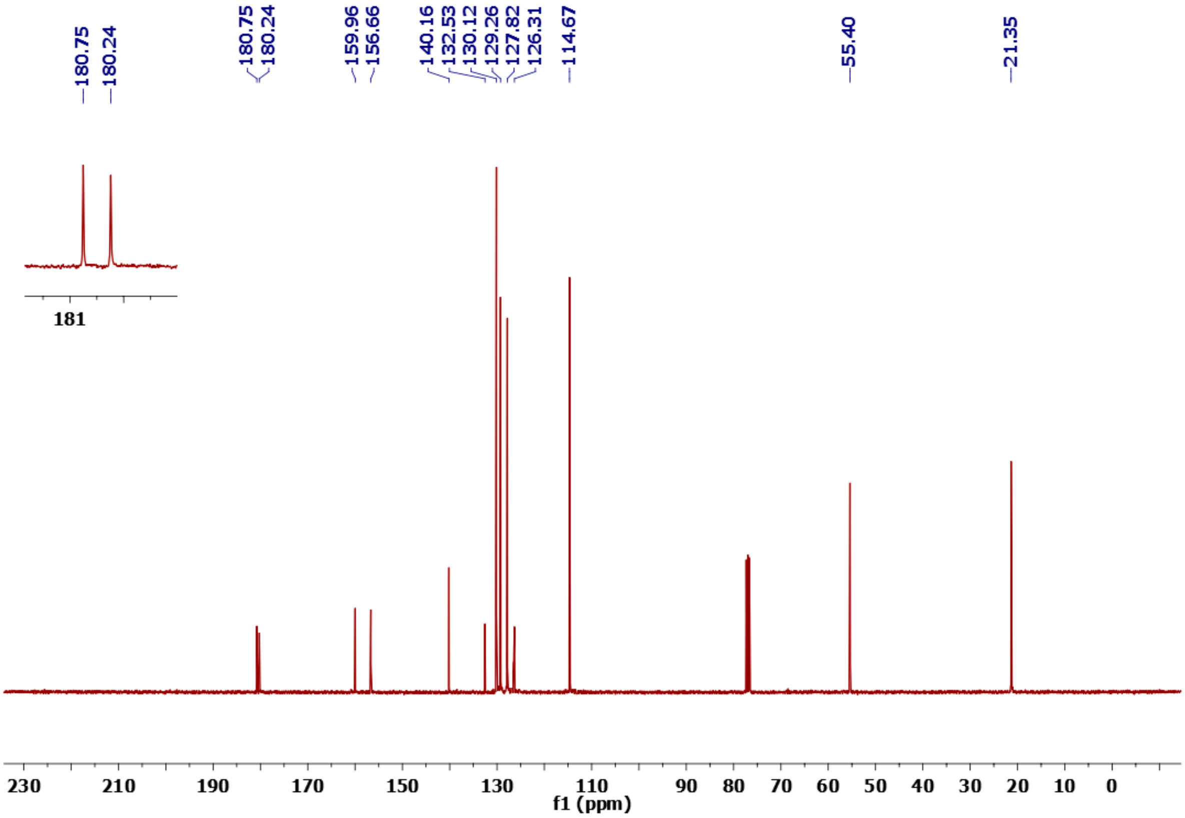

^{13}C-CRAPT NMR ($CDCl_3$) spectrum of 5-imino-1-(4-methoxyphenyl)-3-(p-tolyl)imidazolidine-2,4-dithione

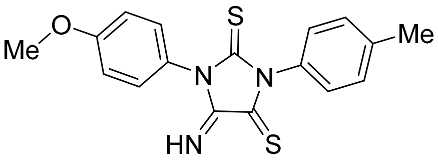

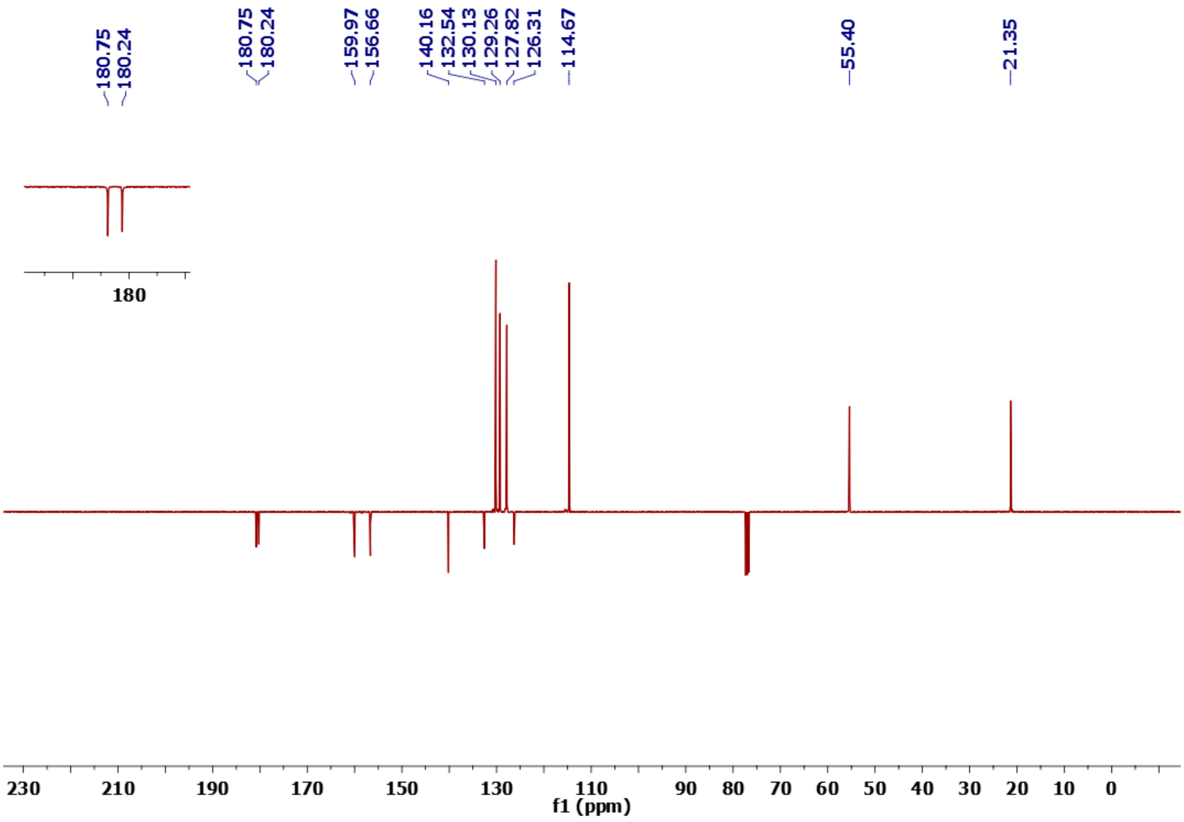

^{1}H-^{1}H-gCOSYAD NMR ($CDCl_3$) spectrum of 5-imino-1-(4-methoxyphenyl)-3-(p-tolyl)imidazolidine-2,4-dithione

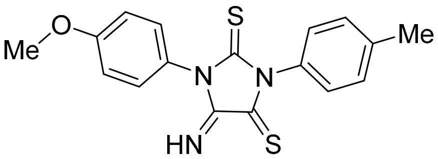

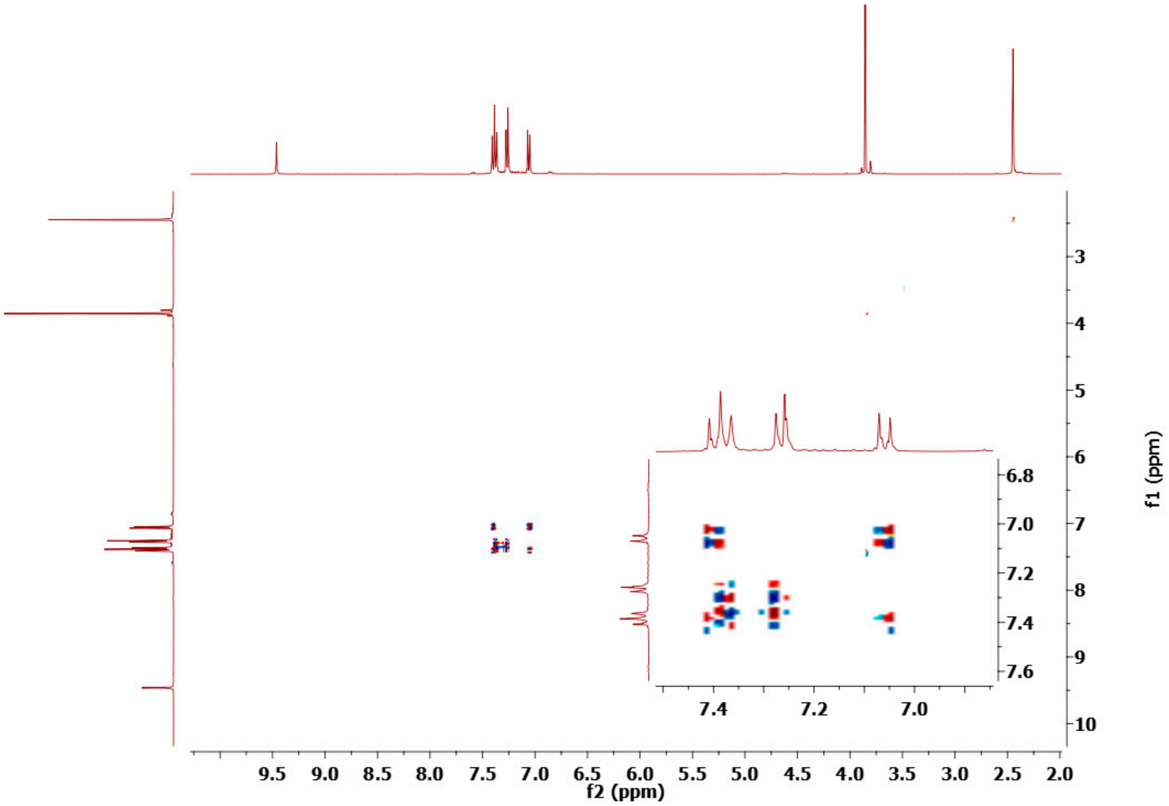

^{1}H-^{13}C-gHSQCAD NMR ($CDCl_3$) spectrum of 5-imino-1-(4-methoxyphenyl)-3-(p-tolyl)imidazolidine-2,4-dithione

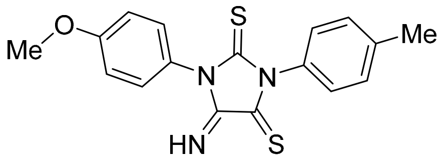

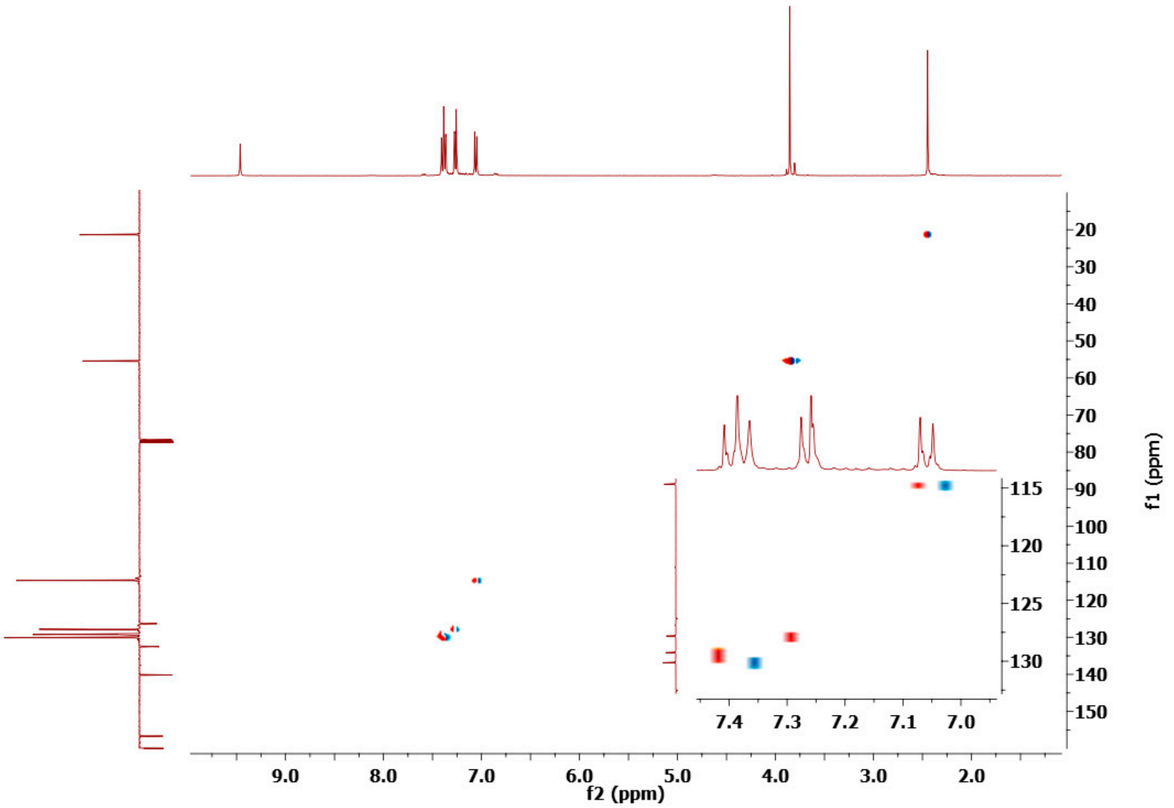

^{1}H-^{13}C-gHMBC NMR ($CDCl_3$) spectrum of 5-imino-1-(4-methoxyphenyl)-3-(p-tolyl)imidazolidine-2,4-dithione

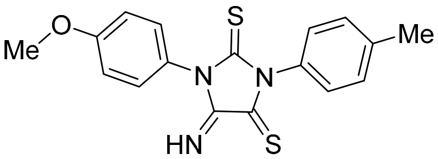

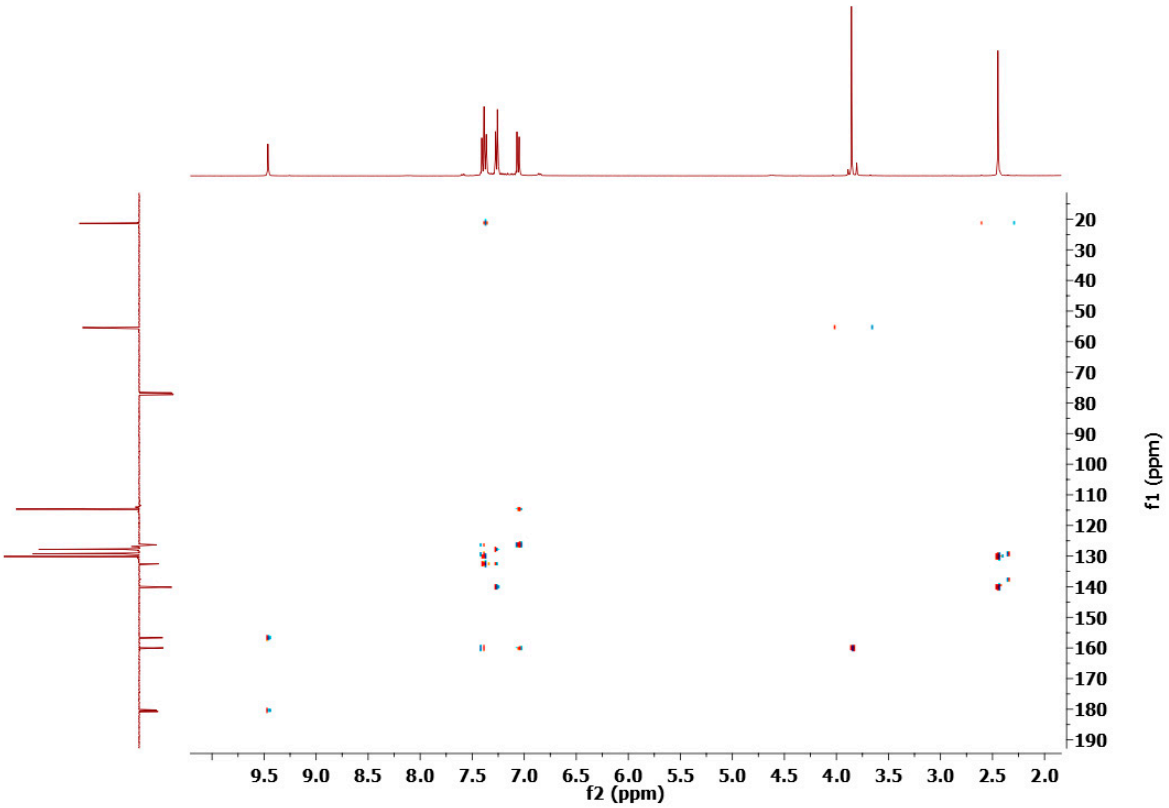

^{1}H NMR ($CDCl_3$) spectrum of (5-imino-3-(4-methoxyphenyl)-2,4-dithioxoimidazolidin-1-yl)(phenyl)methanone (18i')

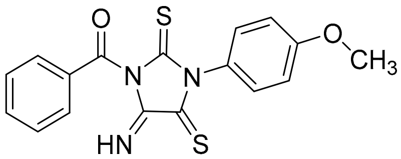

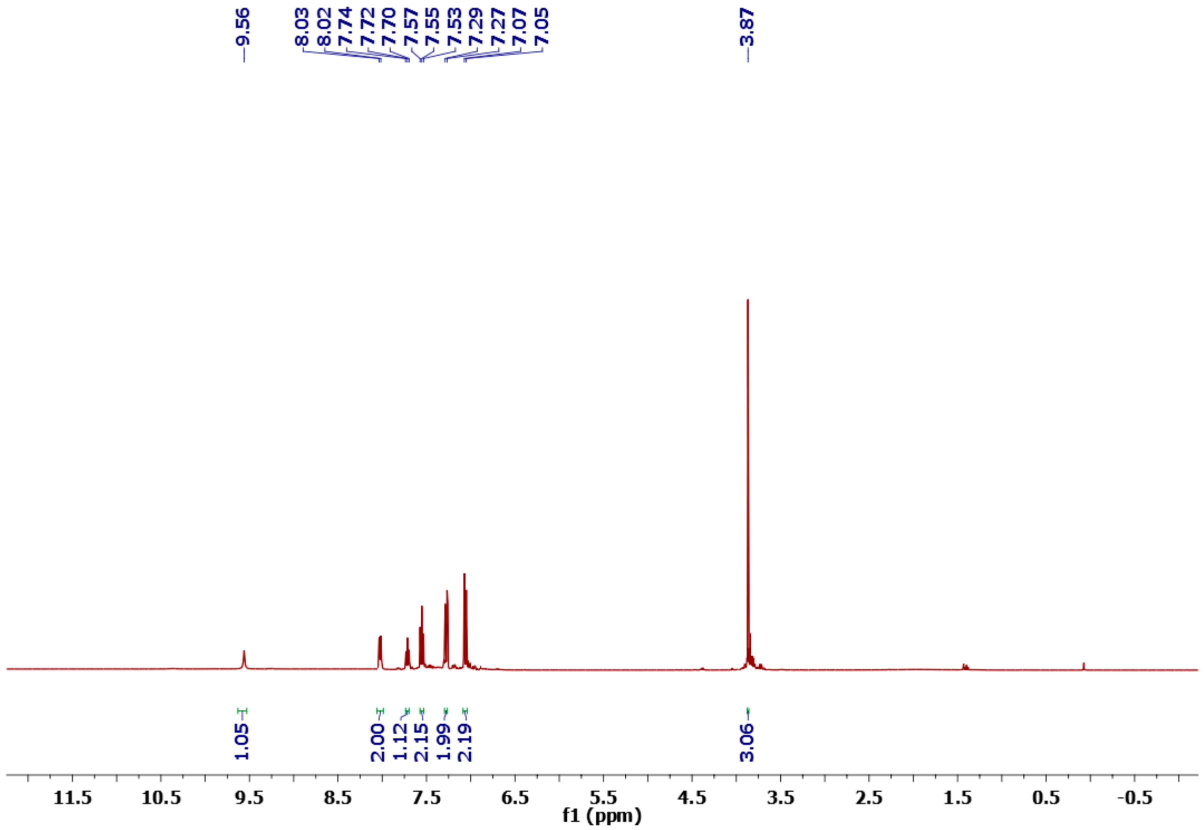

^{13}C-CRAPT NMR ($CDCl_3$) spectrum of of (5-imino-3-(4-methoxyphenyl)-2,4-dithioxoimidazolidin-1-yl)(phenyl)methanone

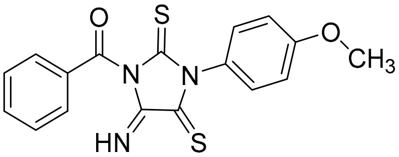

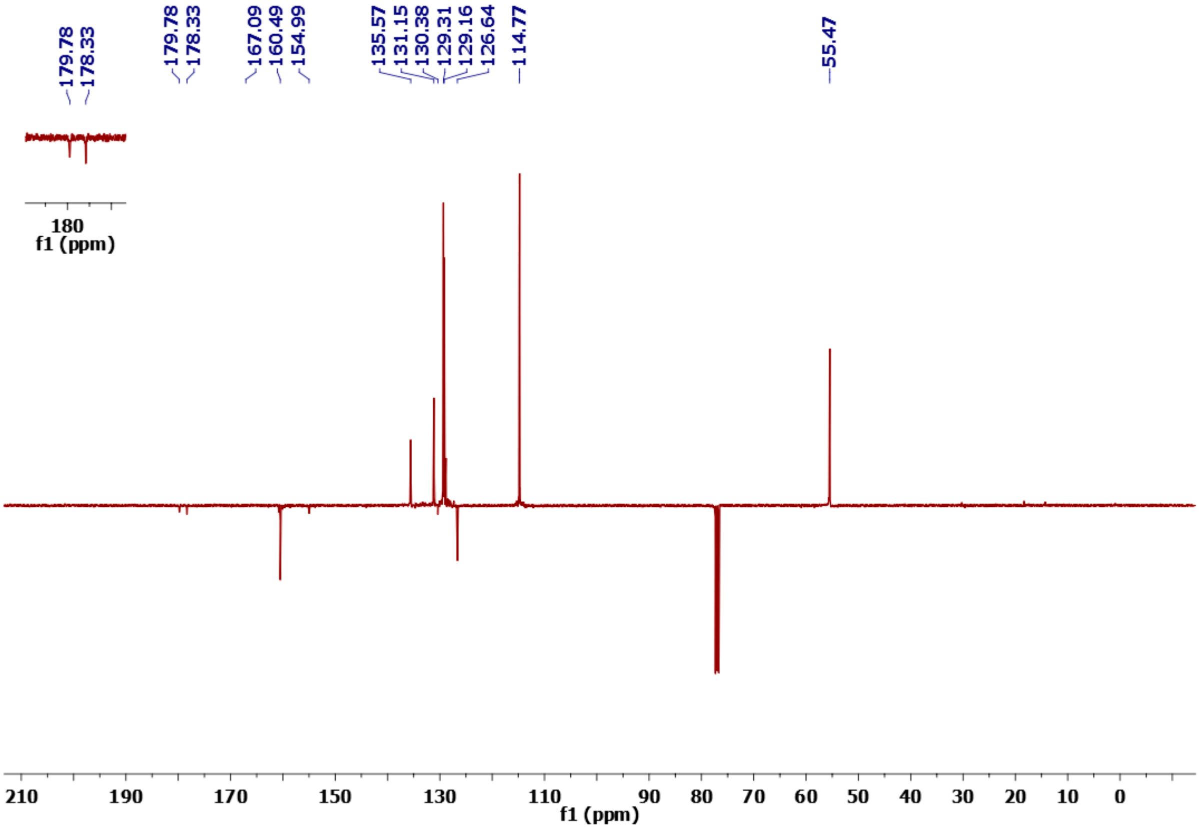

^{1}H NMR ($CDCl_3$) spectrum of 5-imino-3-(4-methoxyphenyl)-1-phenylimidazolidine-2,4-dithione (18j')

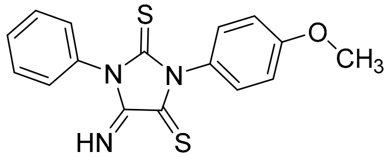

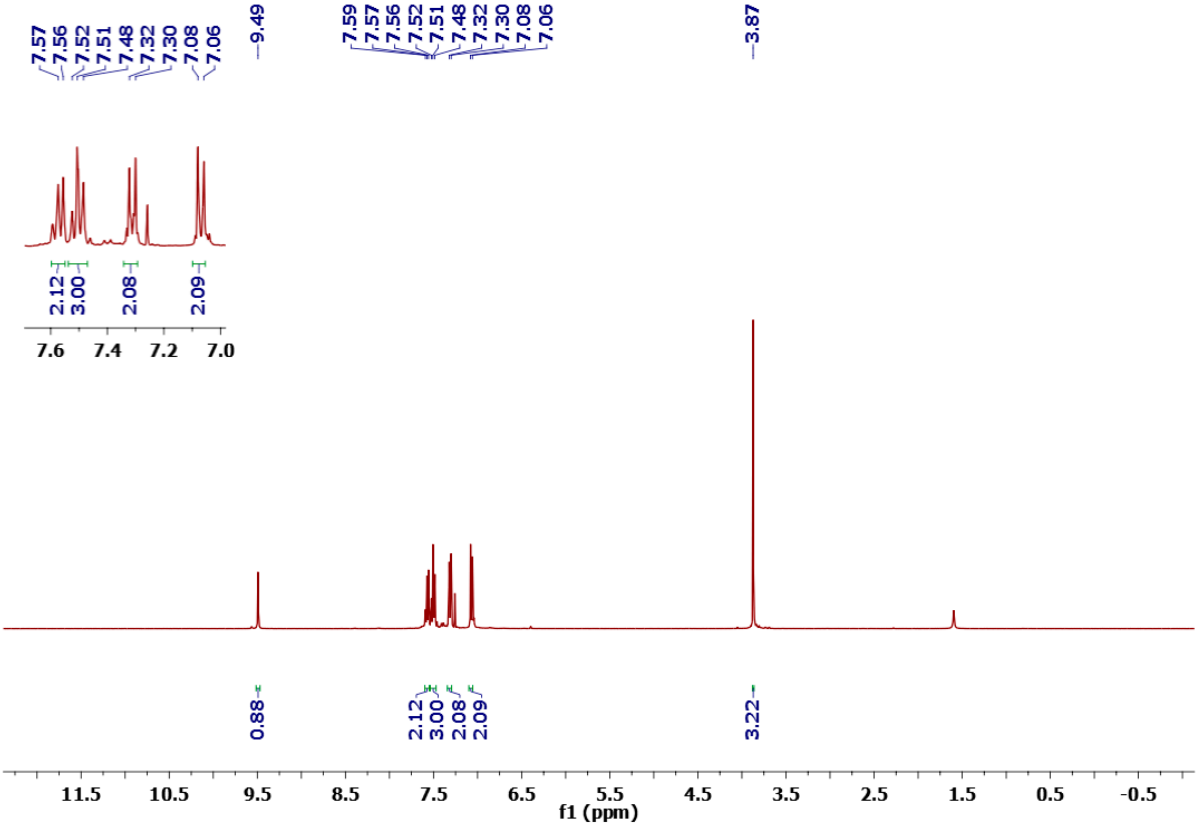

^{13}C NMR ($CDCl_3$) spectrum of 5-imino-3-(4-methoxyphenyl)-1-phenylimidazolidine-2,4-dithione

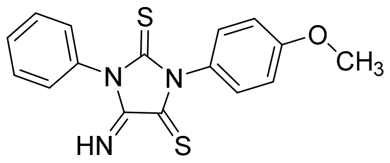

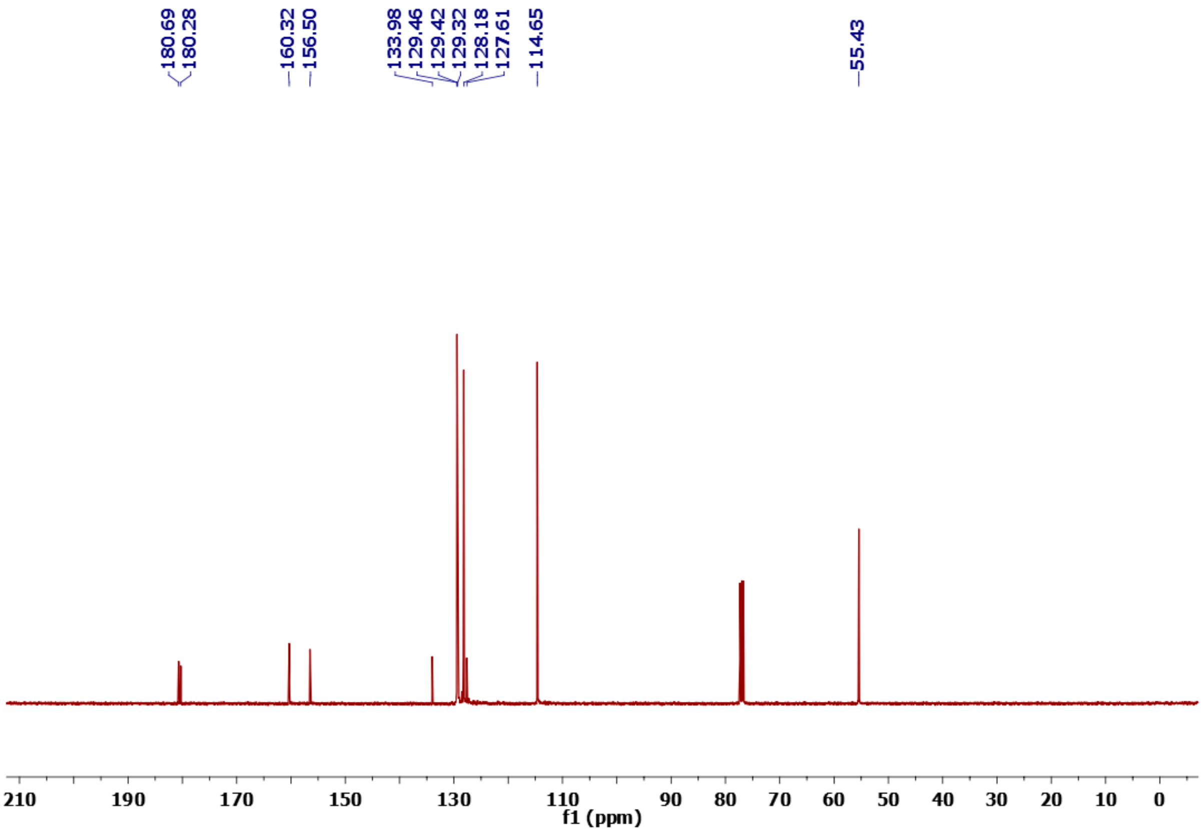

^{13}C-CRAPT NMR ($CDCl_3$) spectrum of 5-imino-3-(4-methoxyphenyl)-1-phenylimidazolidine-2,4-dithione

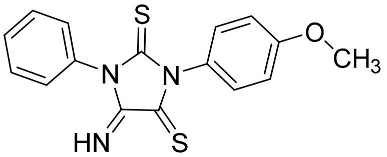

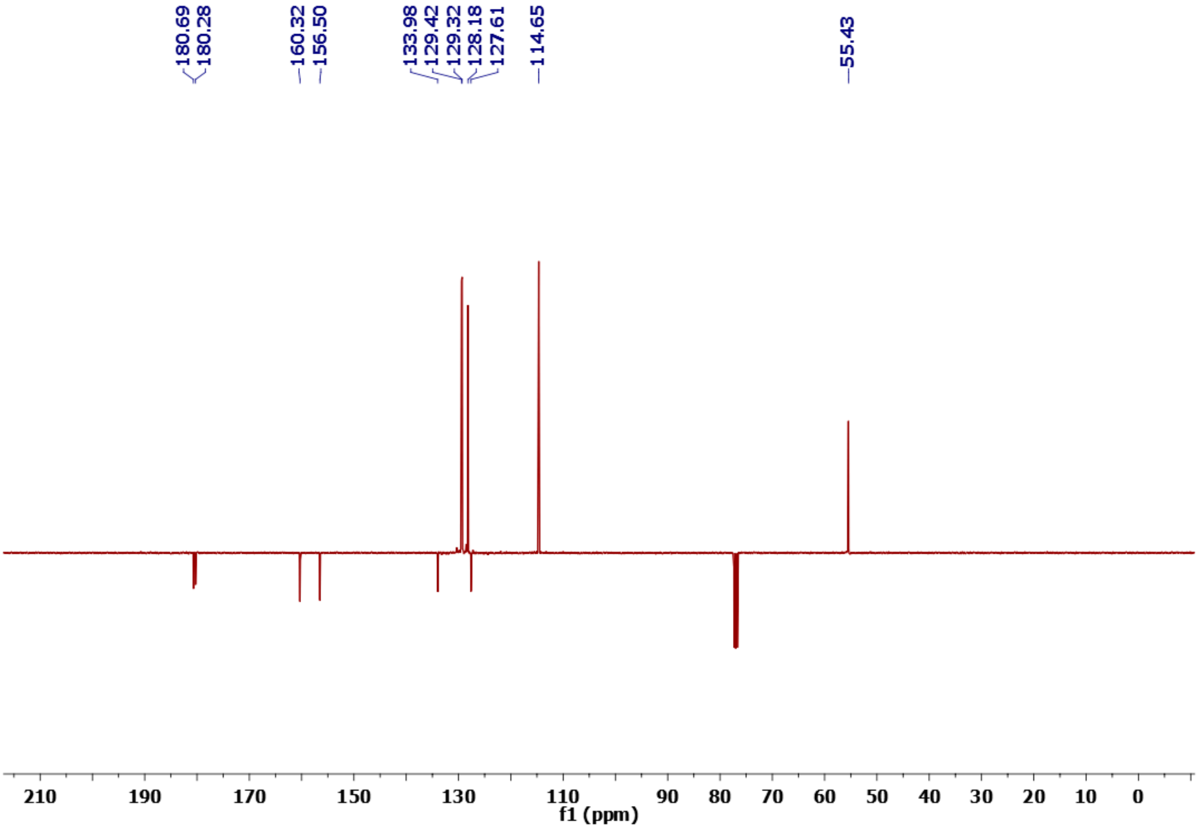

^{1}H-^{1}H-gCOSYAD NMR ($CDCl_3$) spectrum of 5-imino-3-(4-methoxyphenyl)-1-phenylimidazolidine-2,4-dithione

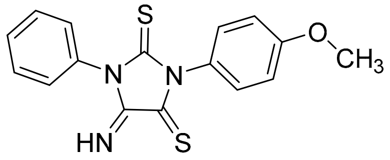

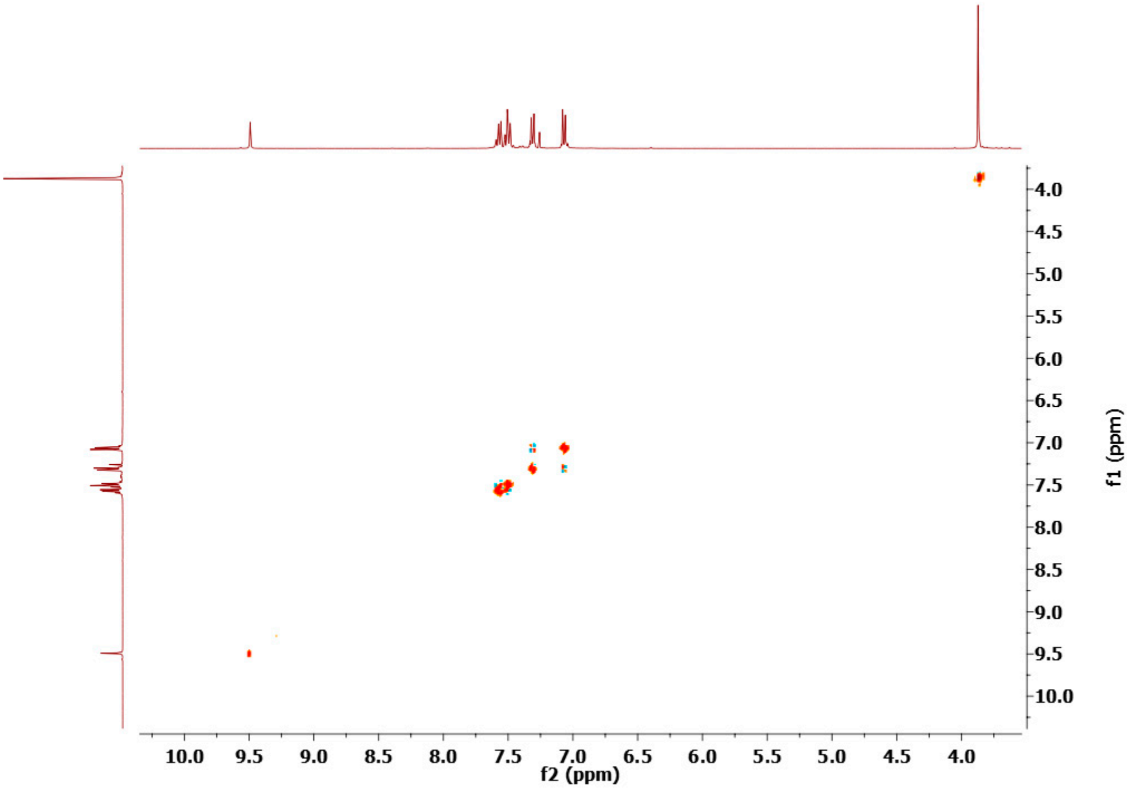

^{1}H-^{13}C-gHSQCAD NMR ($CDCl_3$) spectrum of 5-imino-3-(4-methoxyphenyl)-1-phenylimidazolidine-2,4-dithione

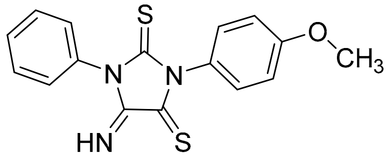

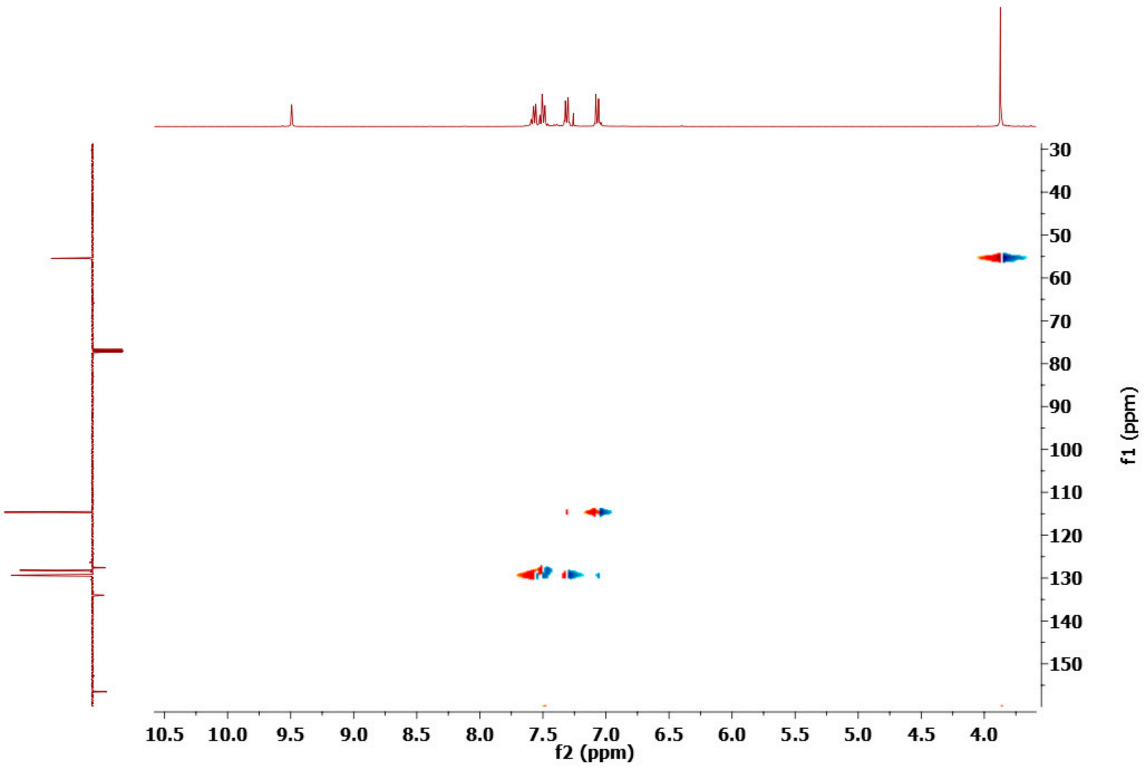

^{1}H-^{13}C-gHMBC NMR ($CDCl_3$) spectrum of 5-imino-3-(4-methoxyphenyl)-1-phenylimidazolidine-2,4-dithione

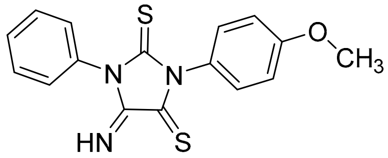

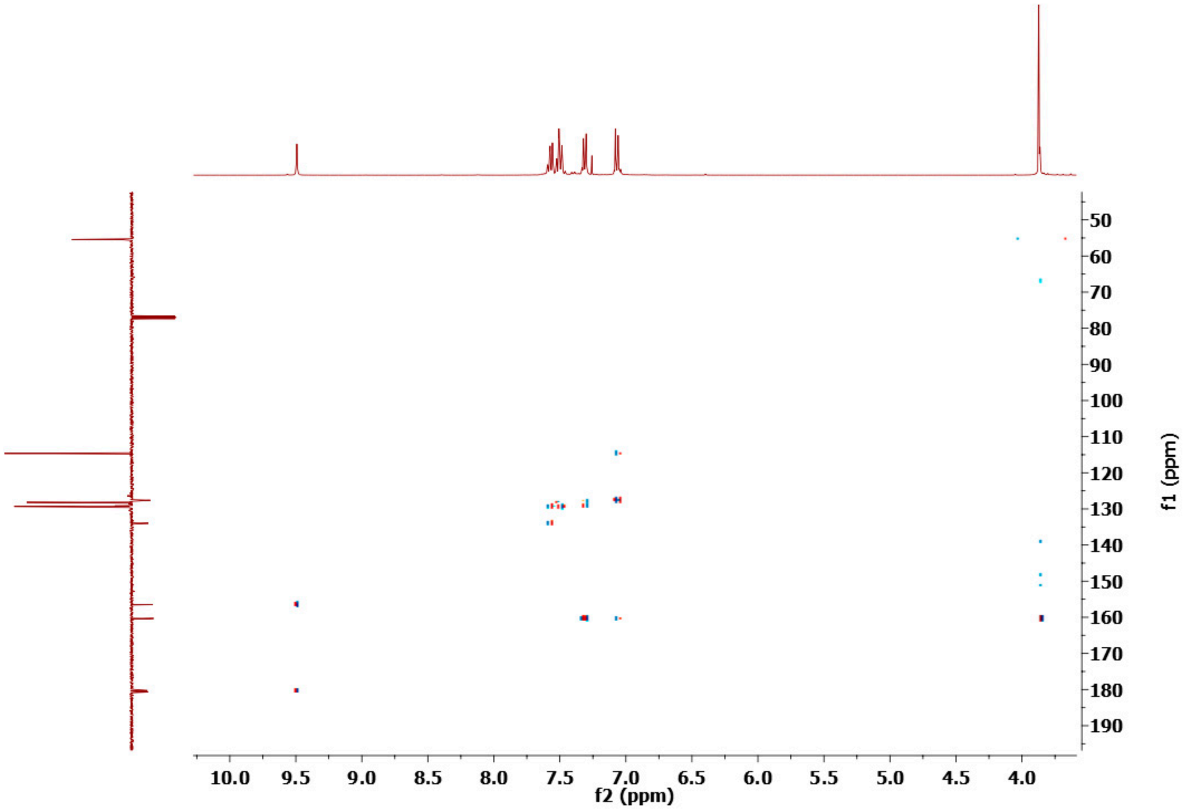

^{1}H NMR ($CDCl_3$) spectrum of 5-imino-3-(4-methoxyphenyl)-1-(4-(trifluoromethyl)phenyl)imidazolidine-2,4-dithione (18k')

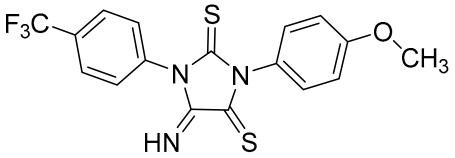

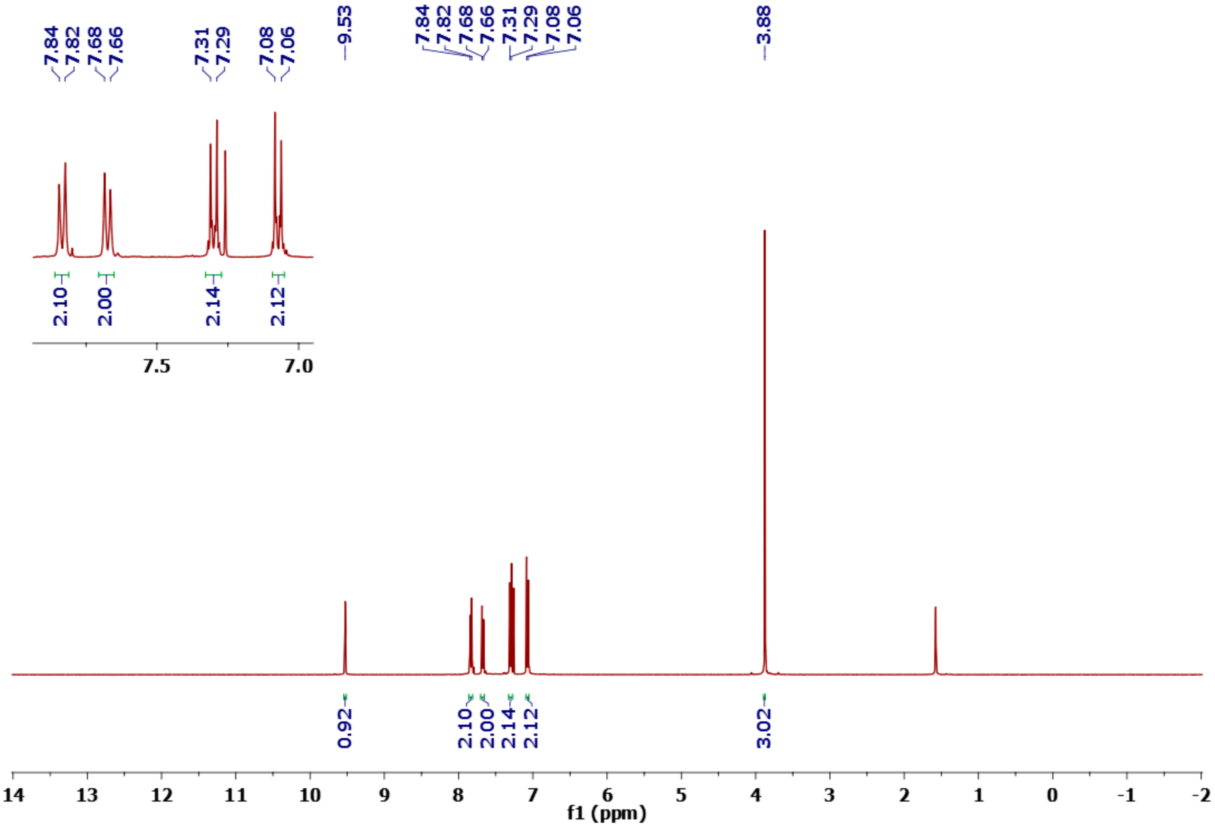

^{13}C NMR ($CDCl_3$) spectrum of 5-imino-3-(4-methoxyphenyl)-1-(4-(trifluoromethyl)phenyl)imidazolidine-2,4-dithione

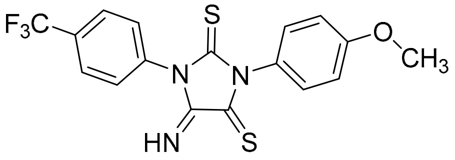

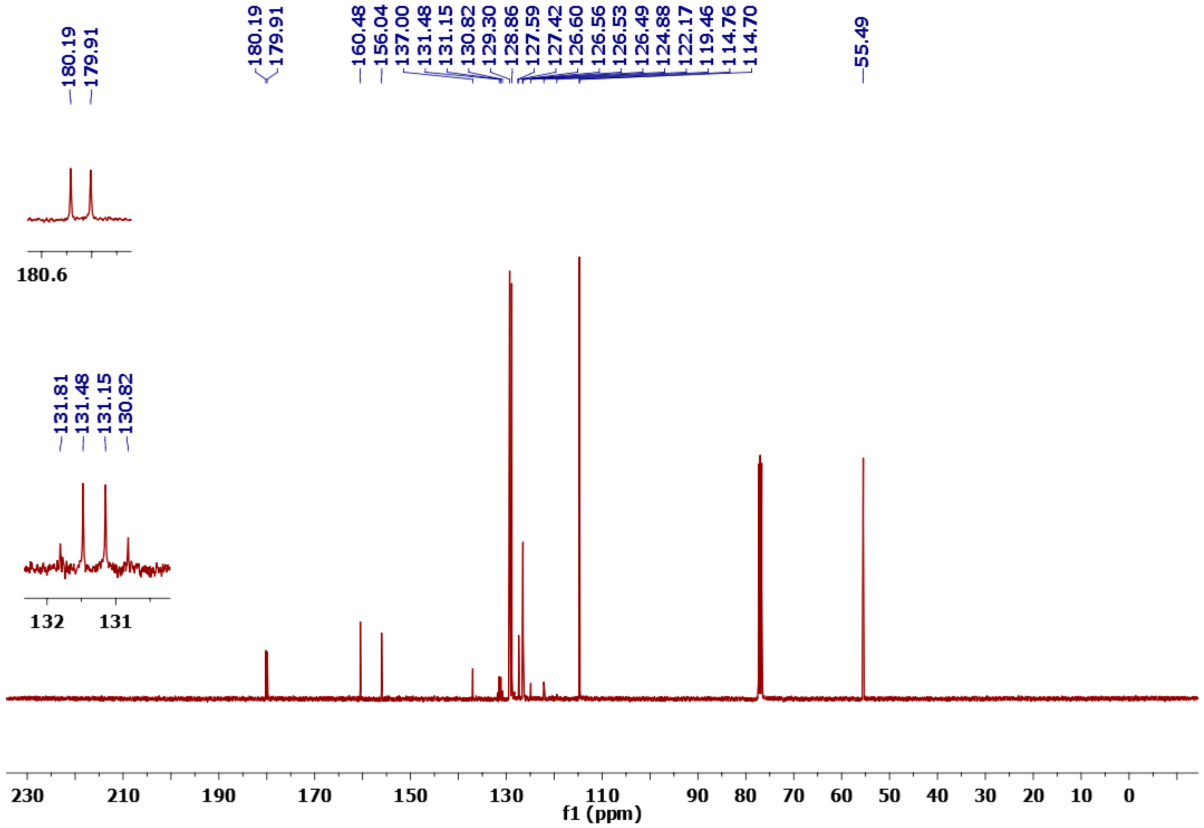

^{13}C-CRAPT NMR ($CDCl_3$) spectrum of 5-imino-3-(4-methoxyphenyl)-1-(4-(trifluoromethyl)phenyl)imidazolidine-2,4-dithione

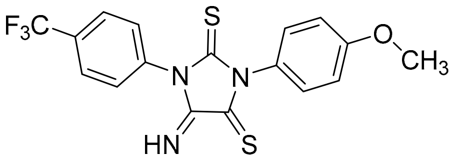

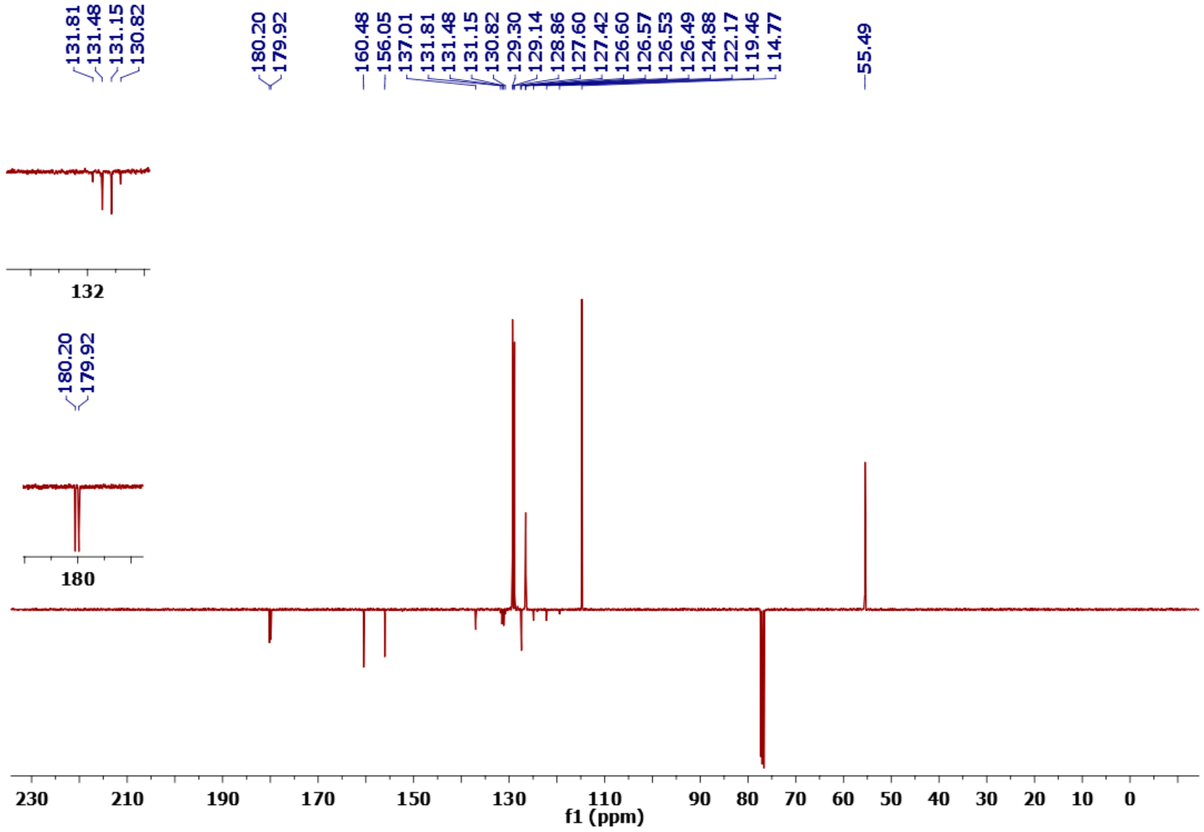

^{1}H-^{1}H-gCOSYAD NMR ($CDCl_3$) spectrum of 5-imino-3-(4-methoxyphenyl)-1-(4-(trifluoromethyl)phenyl)imidazolidine-2,4-dithione

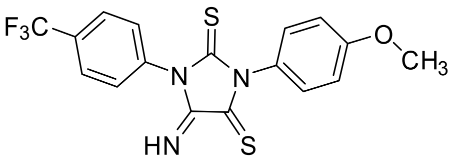

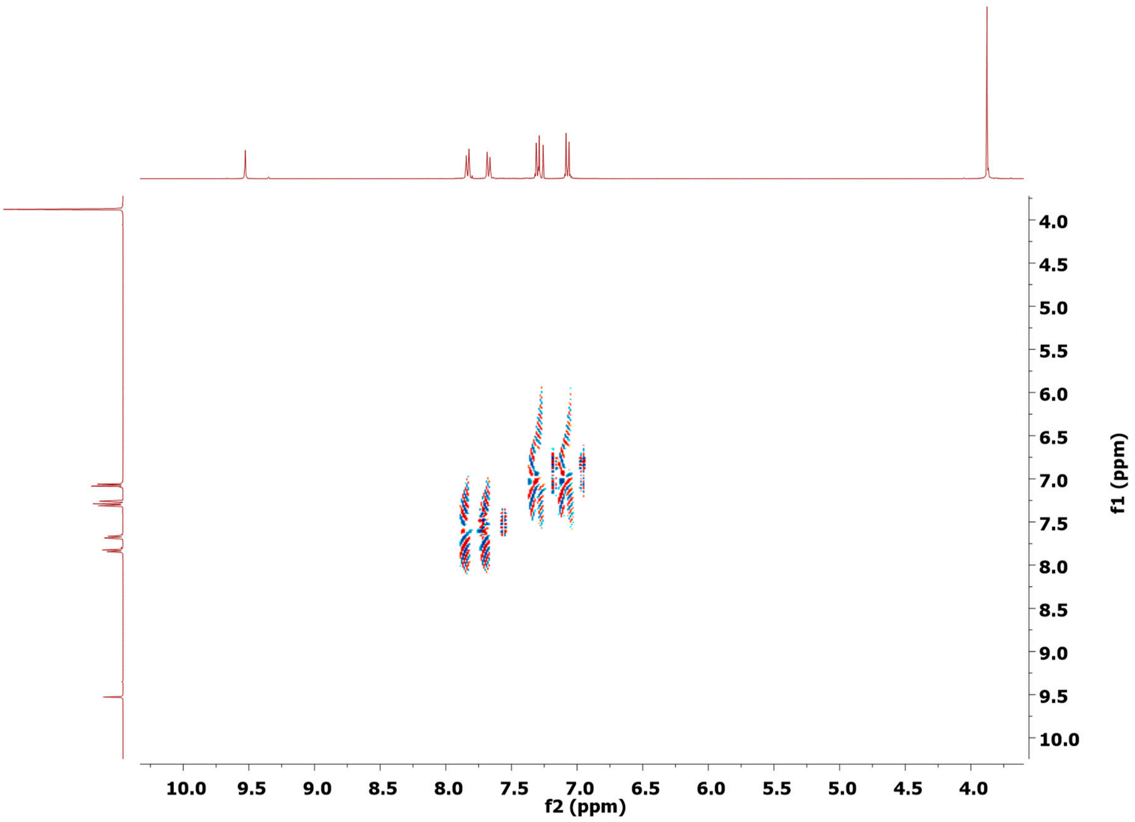

^{1}H-^{13}C-gHSQCAD NMR ($CDCl_3$) spectrum of 5-imino-3-(4-methoxyphenyl)-1-(4-(trifluoromethyl)phenyl)imidazolidine-2,4-dithione

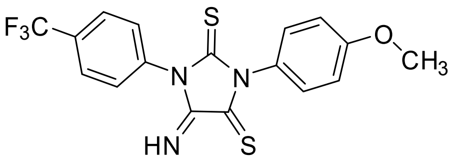

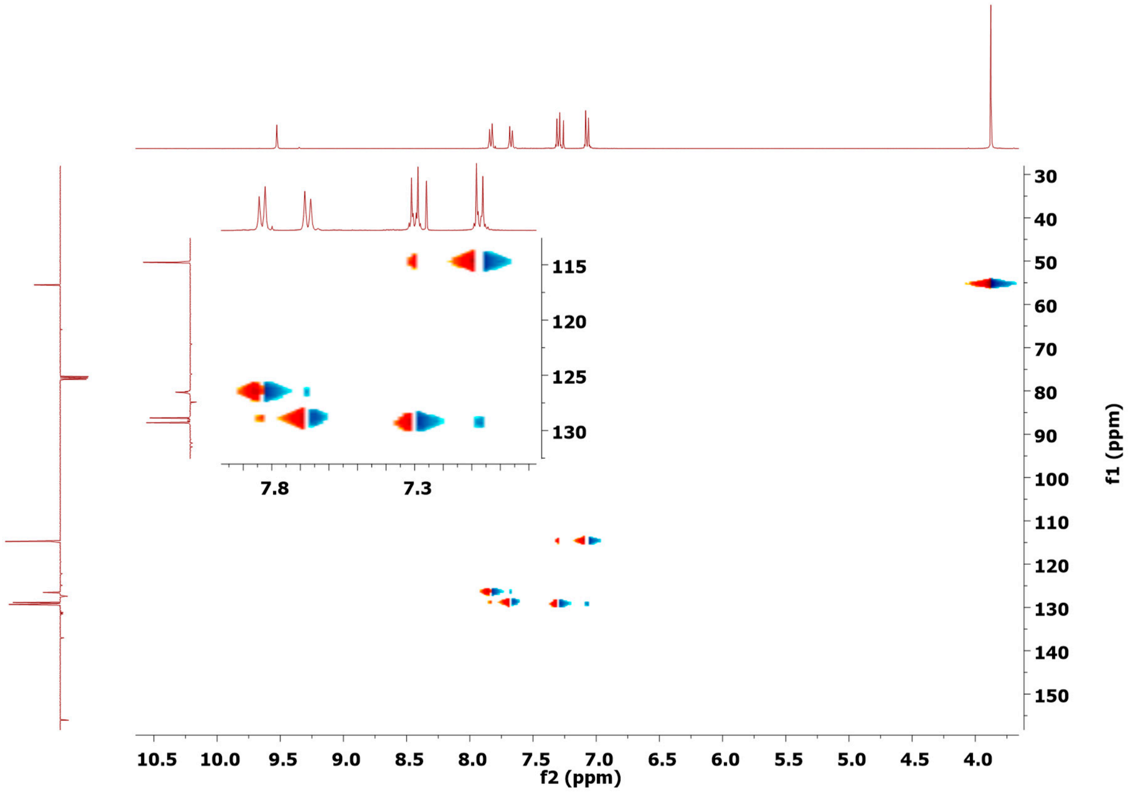

^{1}H-^{13}C-gHMBC NMR ($CDCl_3$) spectrum of 5-imino-3-(4-methoxyphenyl)-1-(4-(trifluoromethyl)phenyl)imidazolidine-2,4-dithione

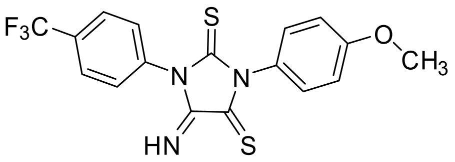

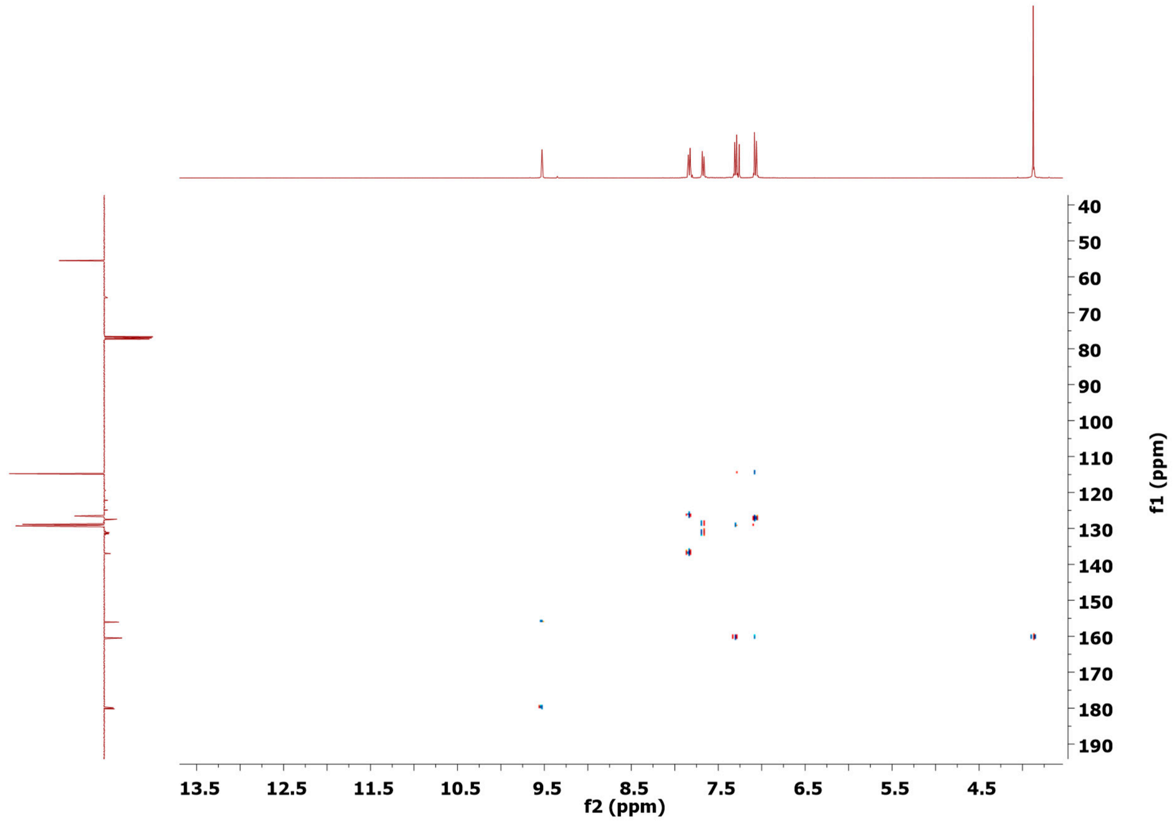

^{1}H NMR ($CDCl_3$) spectrum of 5-imino-3-(4-methoxyphenyl)-1-(p-tolyl)imidazolidine-2,4-dithione (18l')

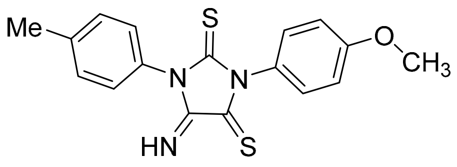

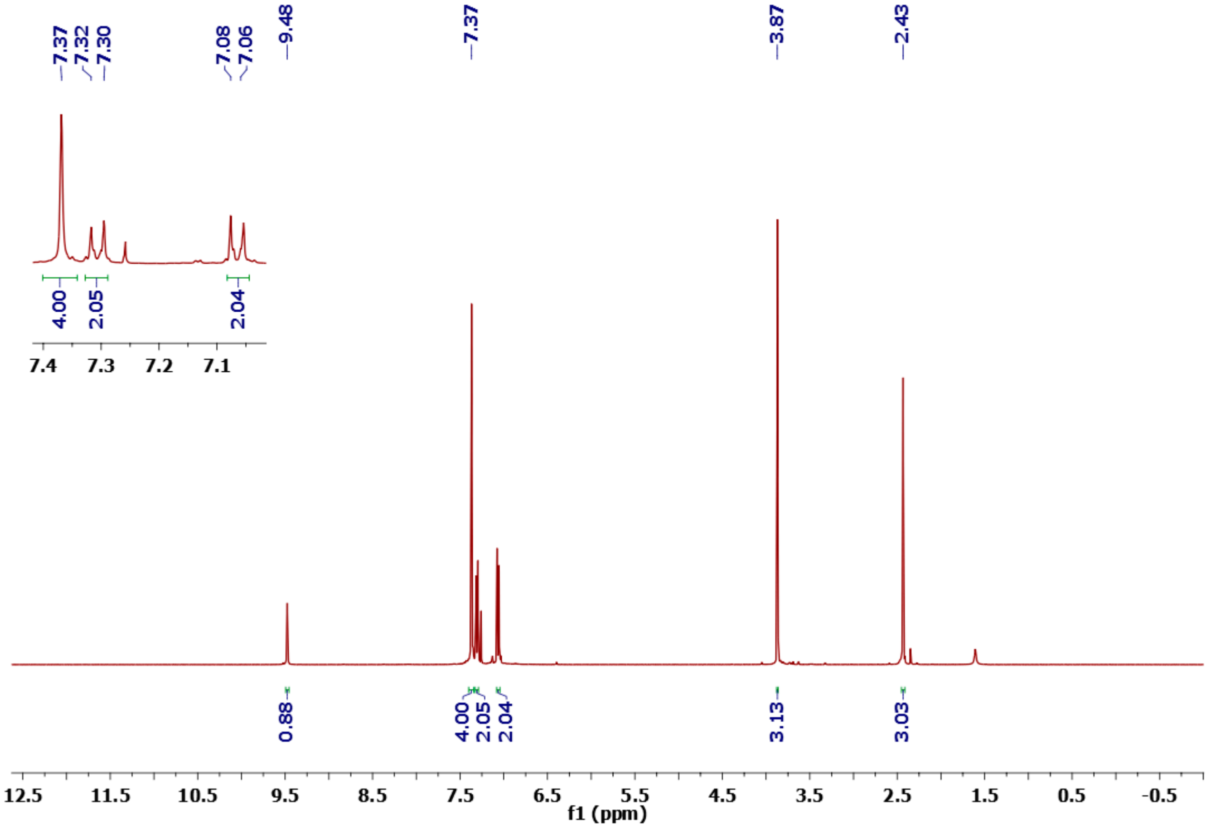

^{13}C NMR ($CDCl_3$) spectrum of 5-imino-3-(4-methoxyphenyl)-1-(p-tolyl)imidazolidine-2,4-dithione

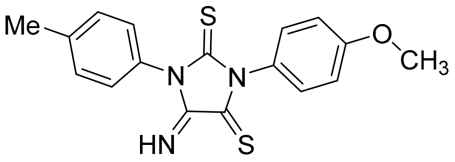

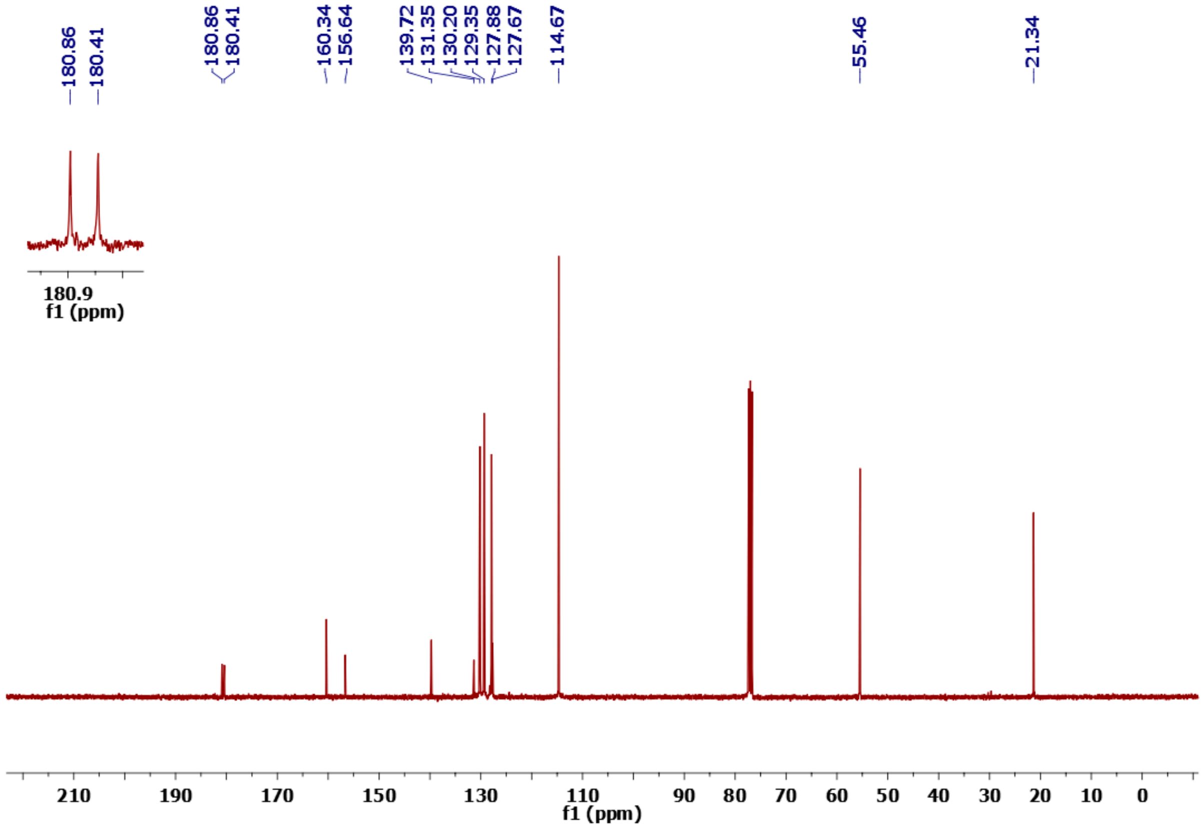

^{13}C-CRAPT NMR ($CDCl_3$) spectrum of 5-imino-3-(4-methoxyphenyl)-1-(p-tolyl)imidazolidine-2,4-dithione

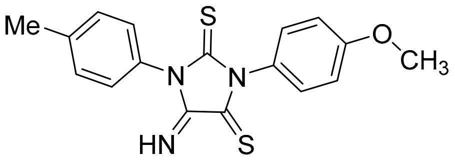

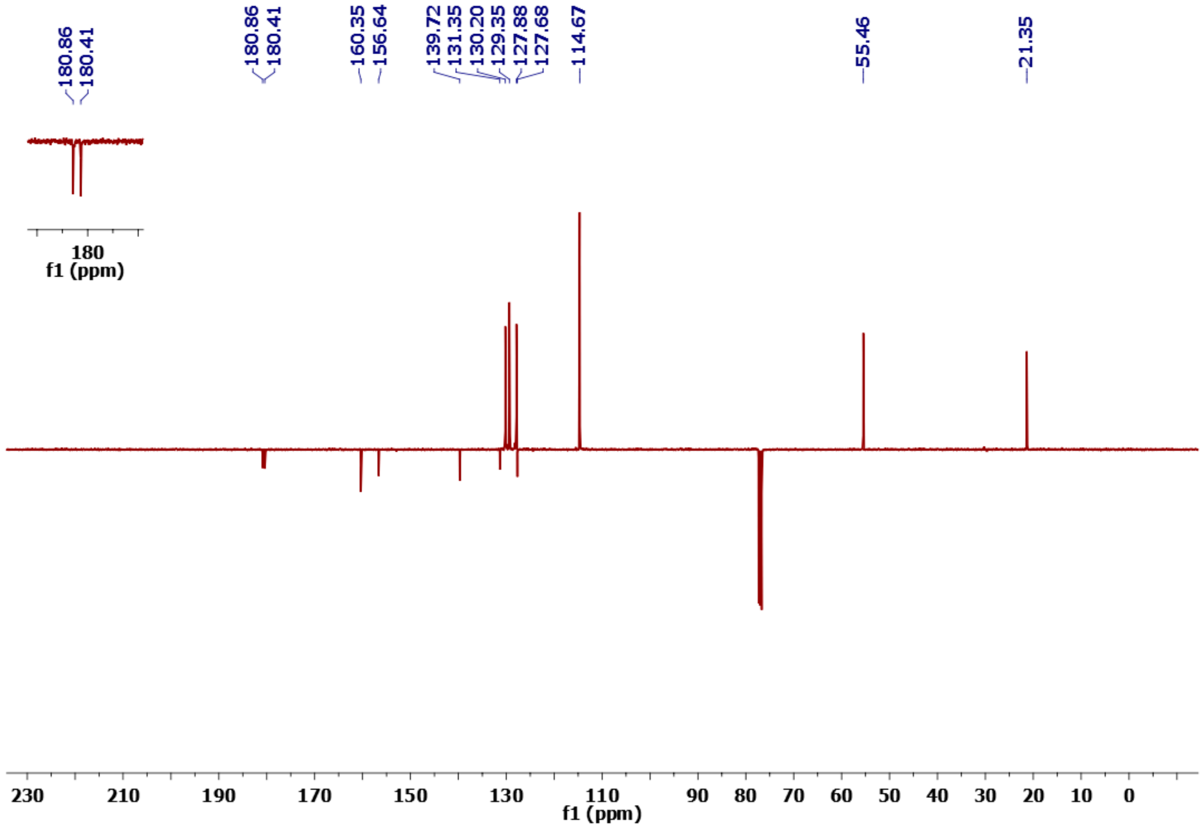

^{1}H-^{1}H-gCOSYAD NMR ($CDCl_3$) spectrum of 5-imino-3-(4-methoxyphenyl)-1-(p-tolyl)imidazolidine-2,4-dithione

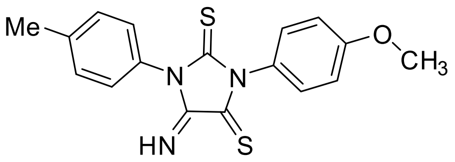

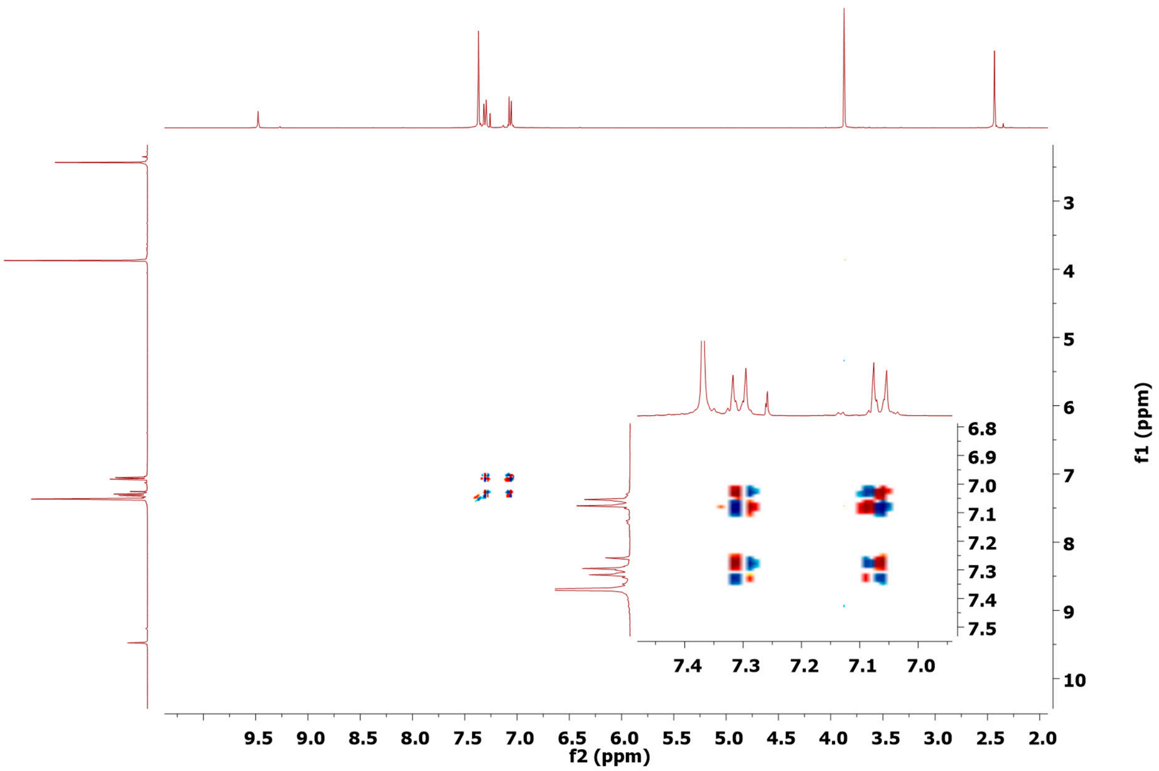

^{1}H-^{13}C-gHSQCAD NMR ($CDCl_3$) spectrum of 5-imino-3-(4-methoxyphenyl)-1-(p-tolyl)imidazolidine-2,4-dithione

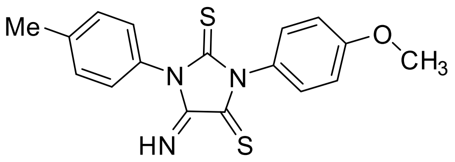

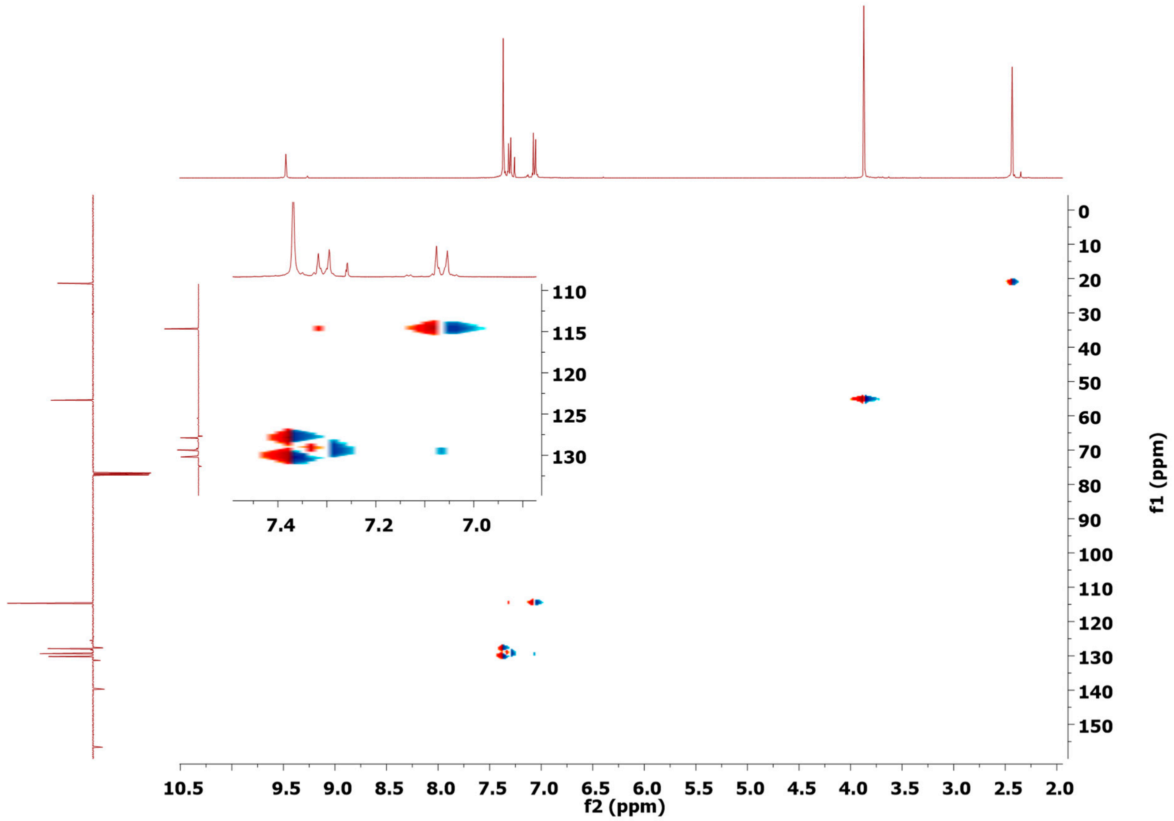

^{1}H-^{13}C-gHMBC NMR ($CDCl_3$) spectrum of 5-imino-3-(4-methoxyphenyl)-1-(p-tolyl)imidazolidine-2,4-dithione

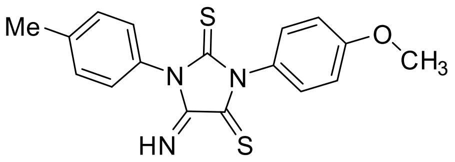

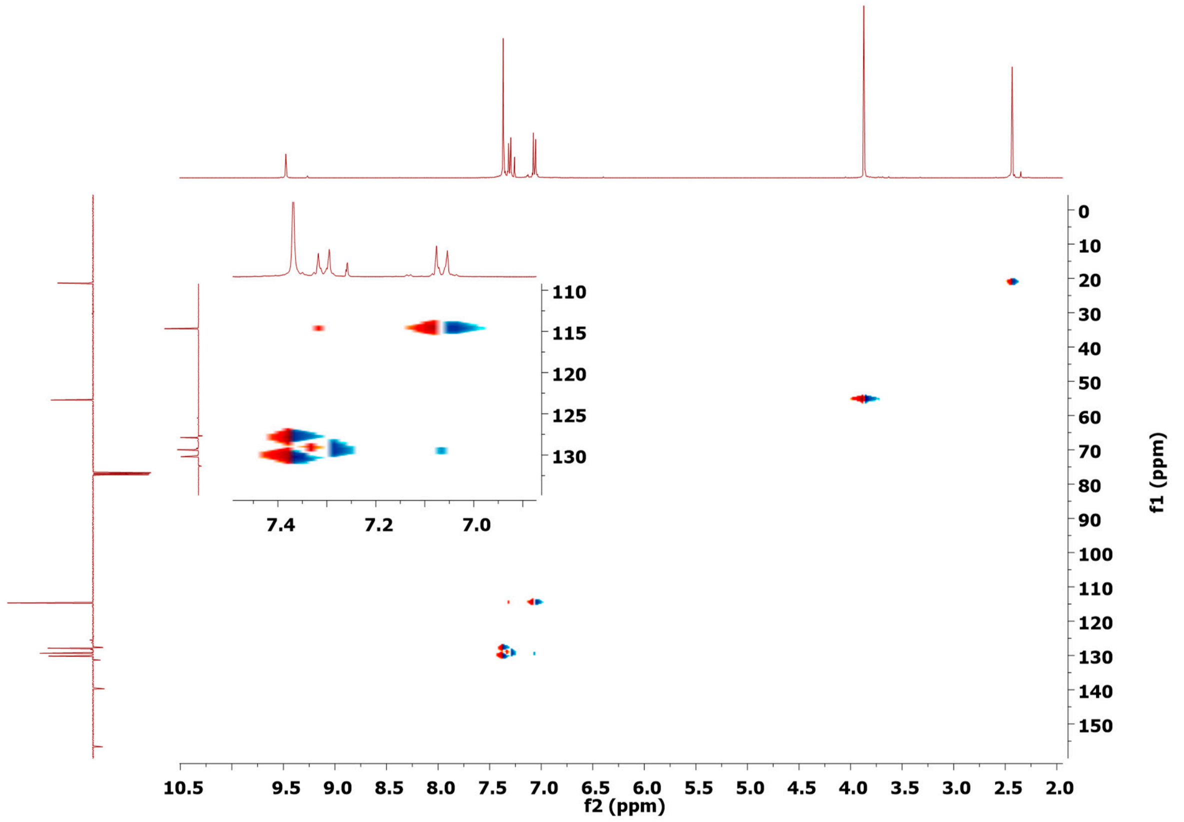

^{1}H NMR ($CDCl_3$) spectrum of 1-(2-fluorophenyl)-5-imino-3-(4-methoxyphenyl)imidazolidine-2,4-dithione (18m')

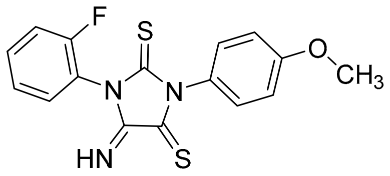

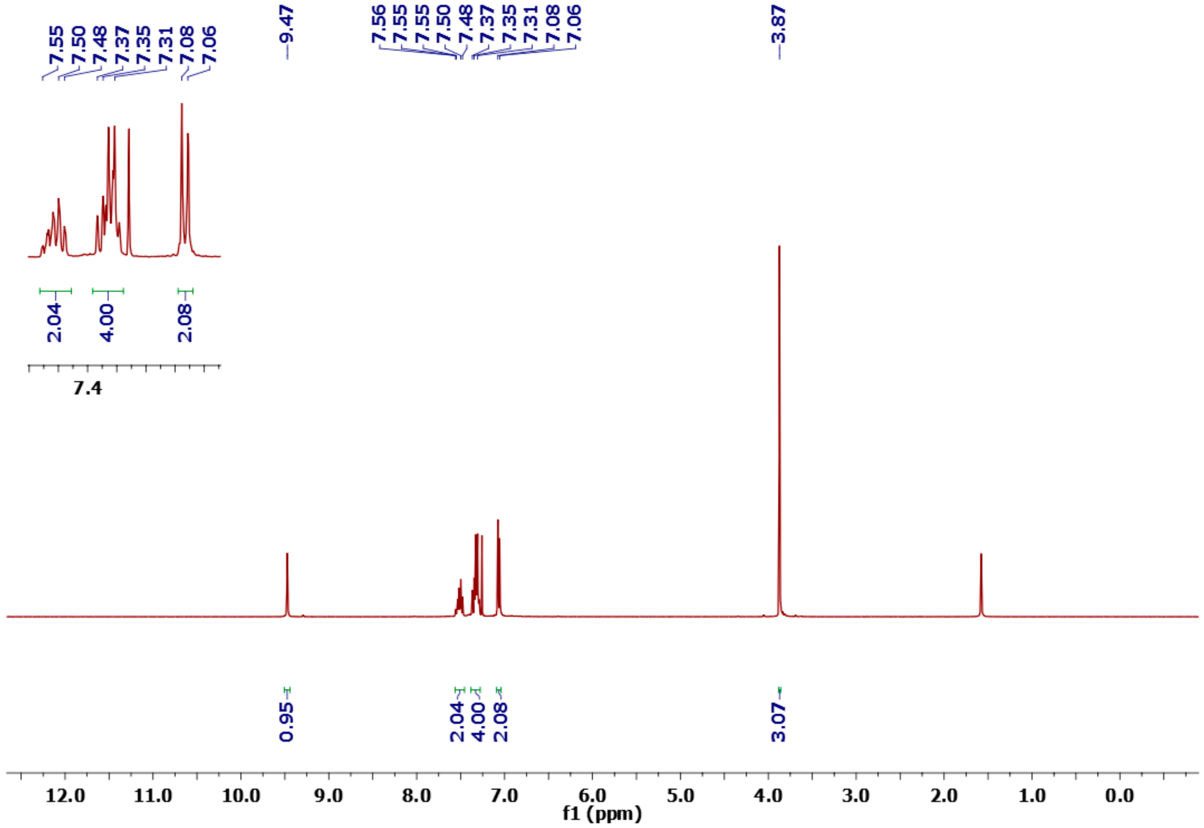

^{13}C NMR ($CDCl_3$) spectrum of 1-(2-fluorophenyl)-5-imino-3-(4-methoxyphenyl)imidazolidine-2,4-dithione

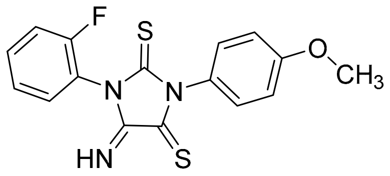

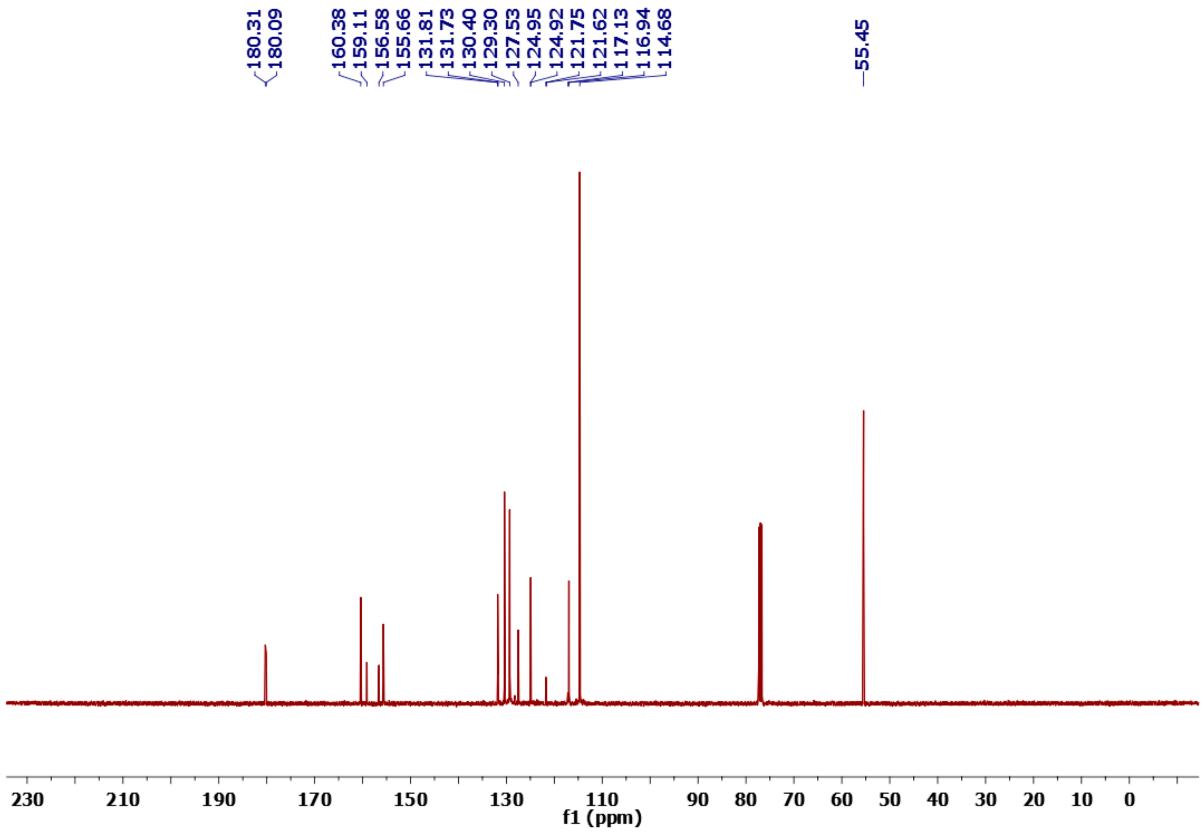

^{13}C-CRAPT NMR ($CDCl_3$) spectrum of 1-(2-fluorophenyl)-5-imino-3-(4-methoxyphenyl)imidazolidine-2,4-dithione

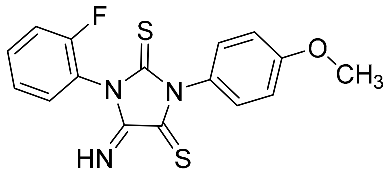

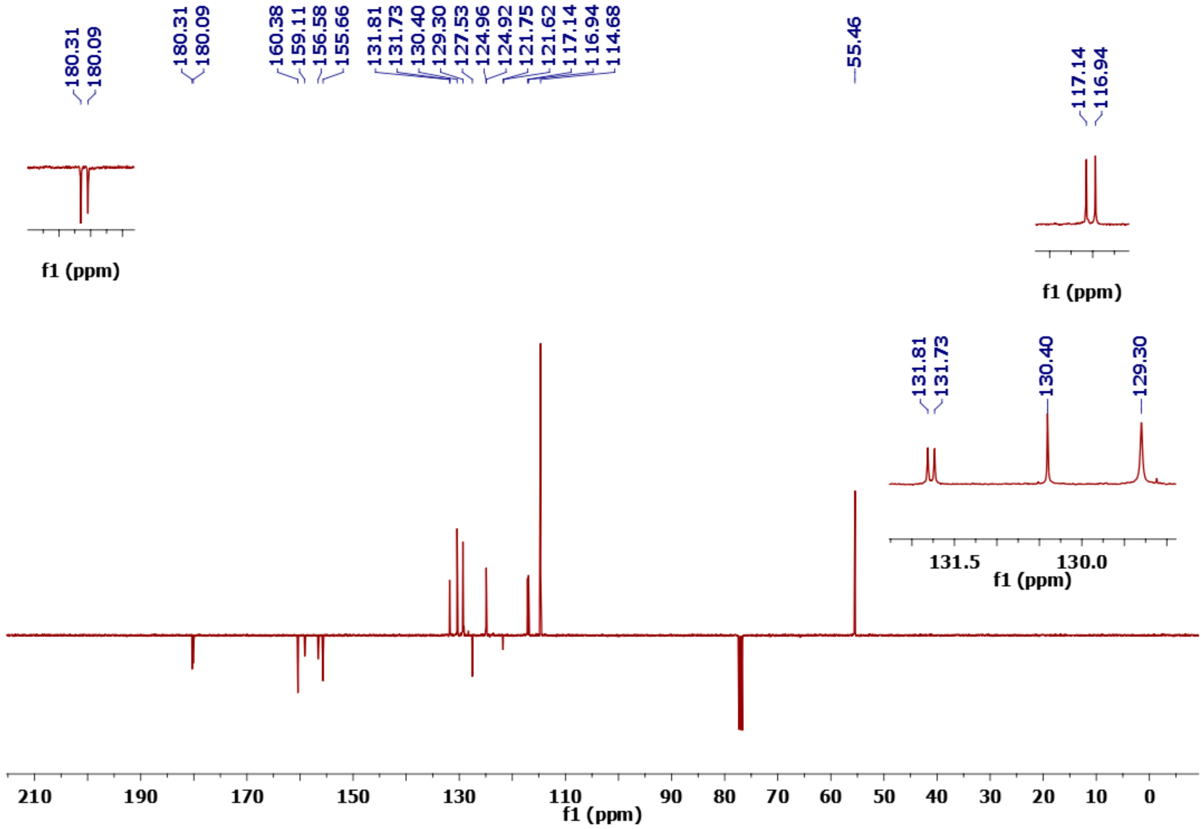

^{1}H-^{1}H-gCOSYAD NMR ($CDCl_3$) spectrum of 1-(2-fluorophenyl)-5-imino-3-(4-methoxyphenyl)imidazolidine-2,4-dithione

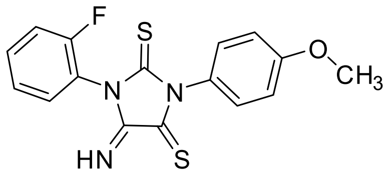

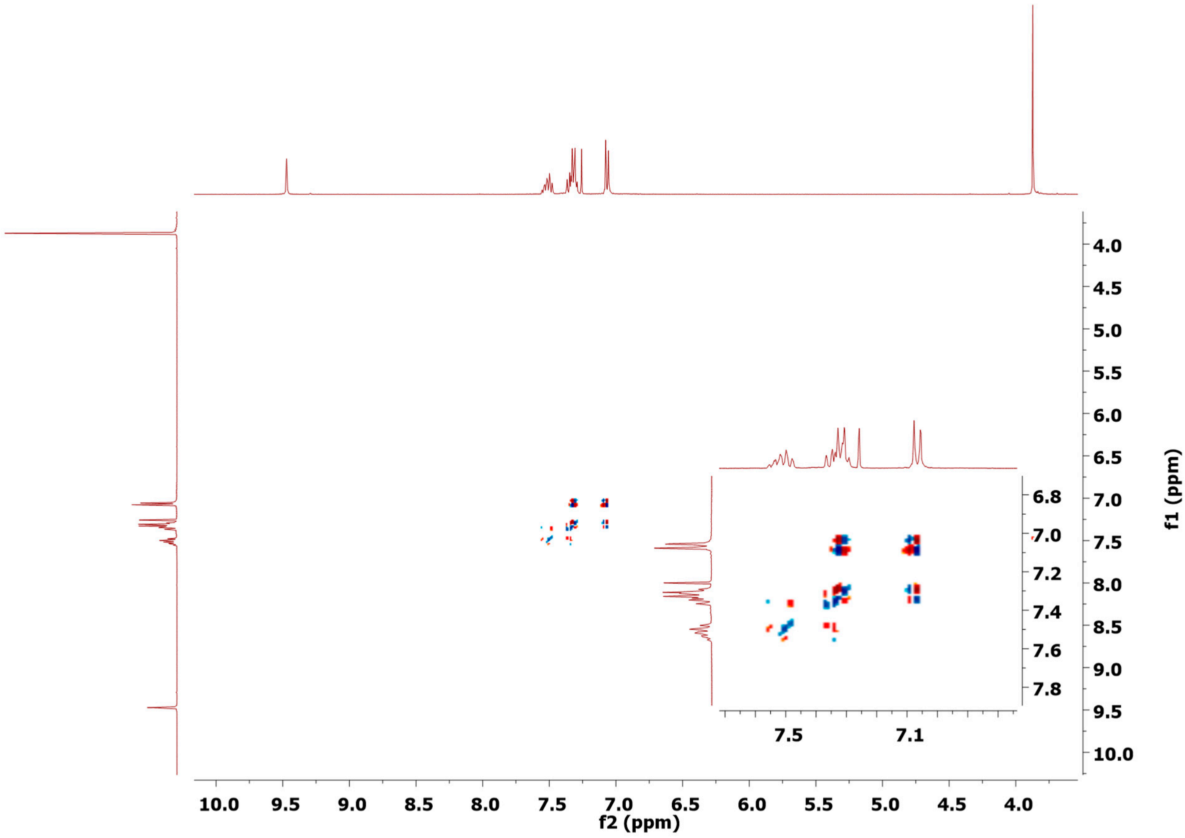

^{1}H-^{13}C-gHSQCAD NMR ($CDCl_3$) spectrum of 1-(2-fluorophenyl)-5-imino-3-(4-methoxyphenyl)imidazolidine-2,4-dithione

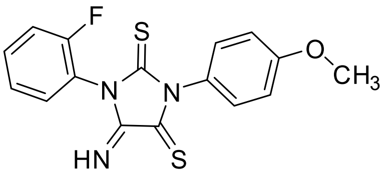

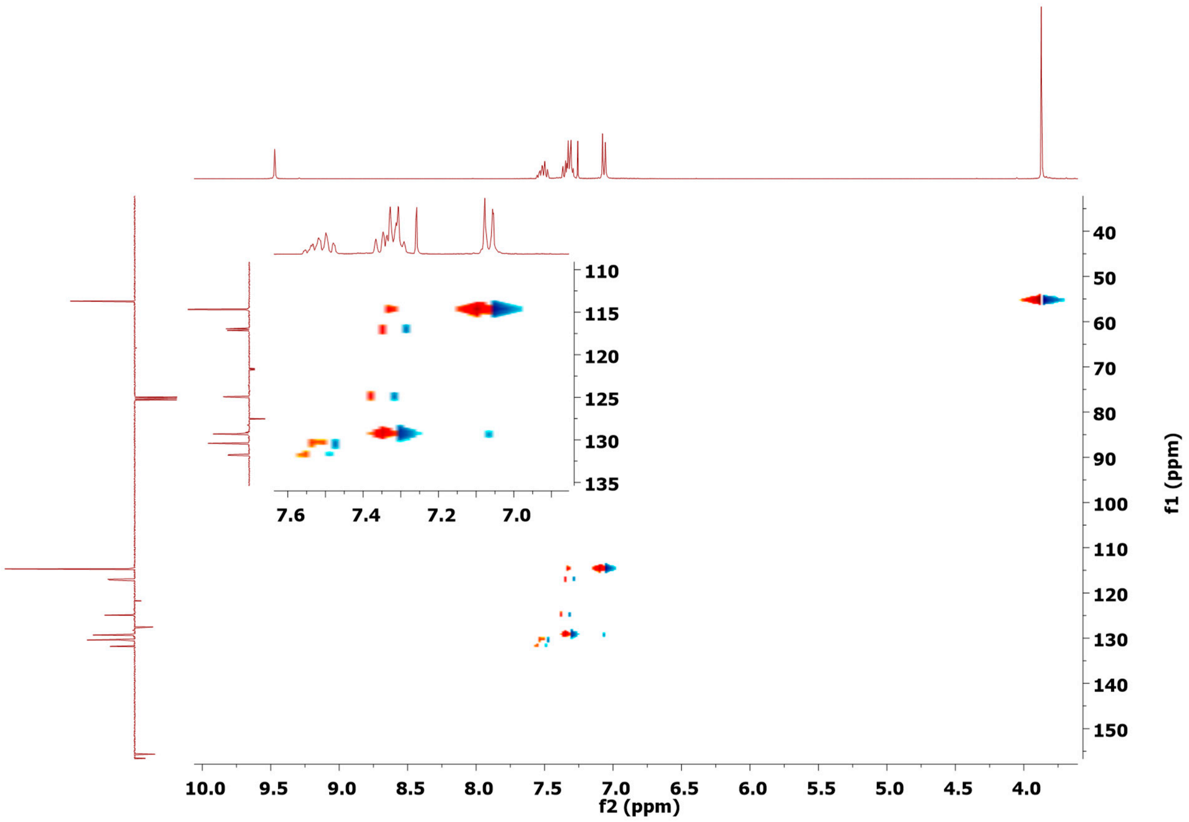

^{1}H-^{13}C-gHMBC NMR ($CDCl_3$) spectrum of 1-(2-fluorophenyl)-5-imino-3-(4-methoxyphenyl)imidazolidine-2,4-dithione

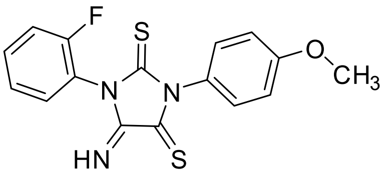

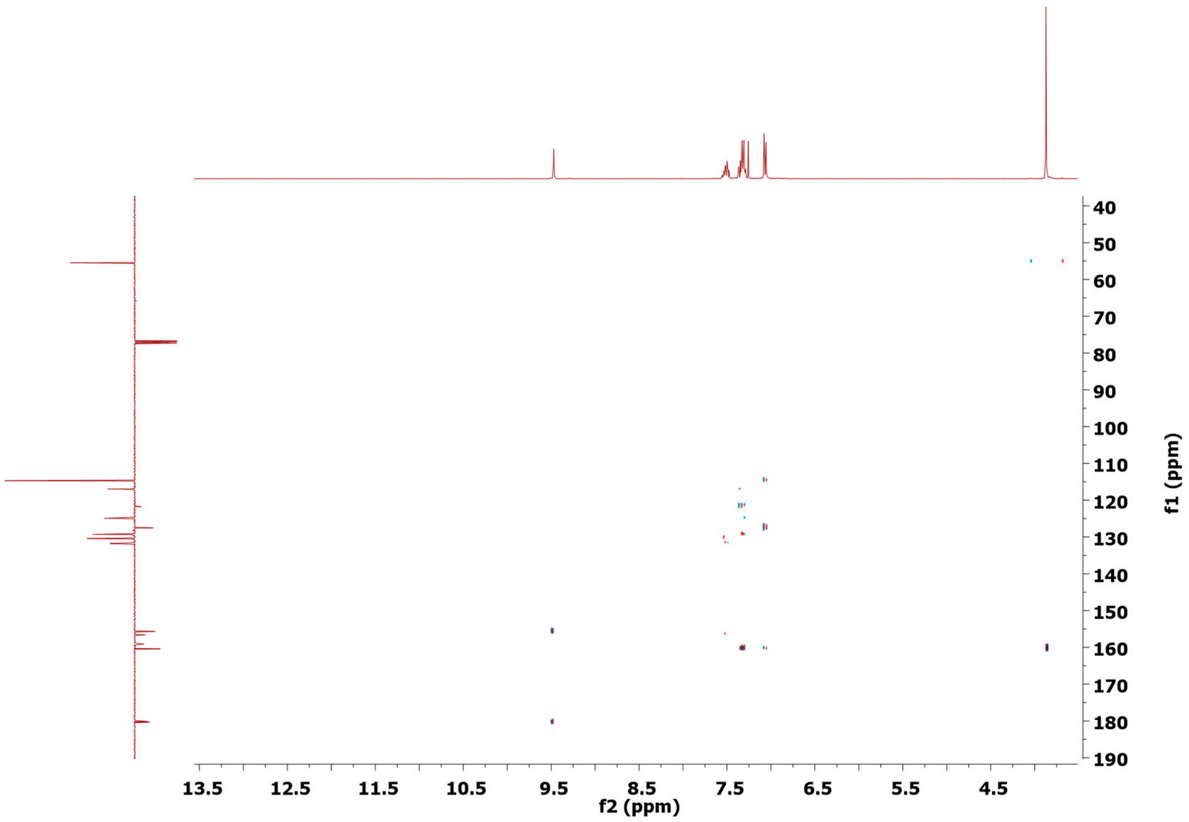

^{1}H NMR ($CDCl_3$) spectrum of 1-(4-chlorophenyl)-5-imino-3-(4-methoxyphenyl)imidazolidine-2,4-dithione (18n')

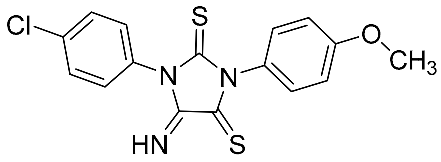

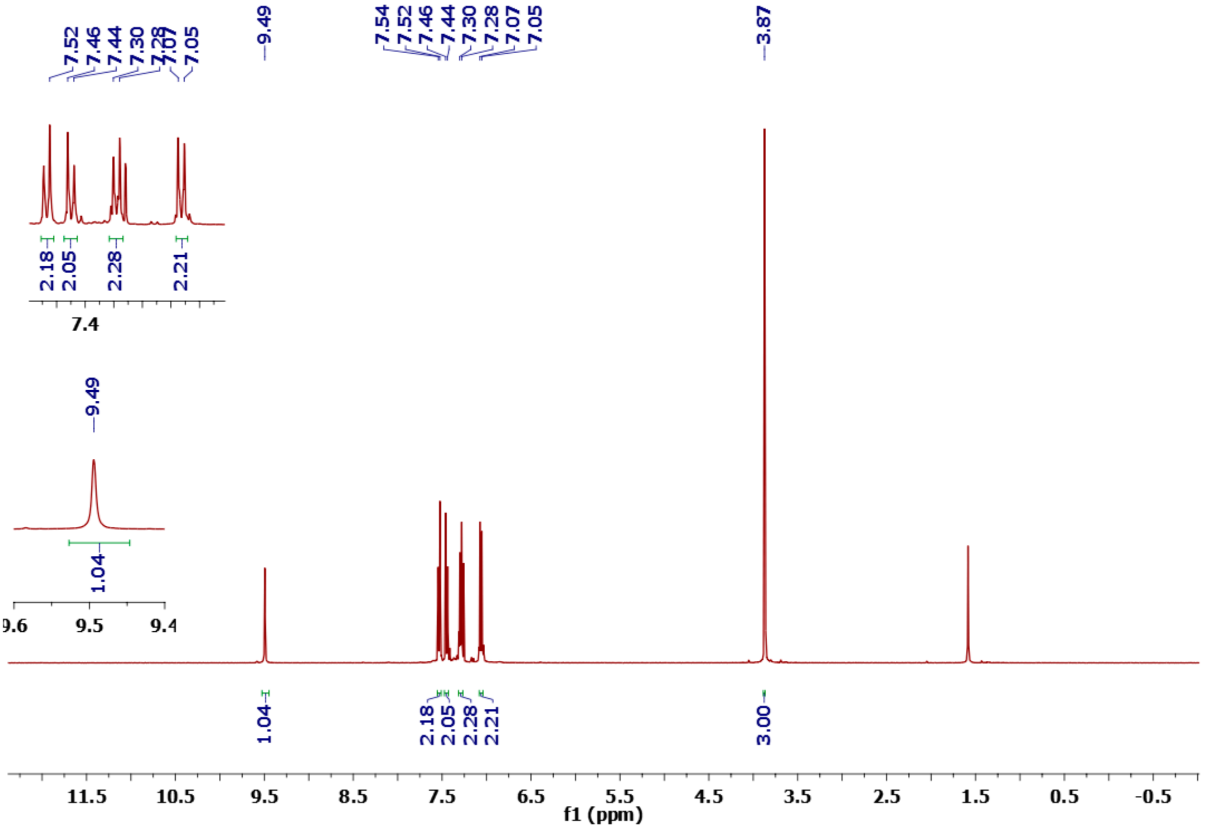

^{13}C NMR ($CDCl_3$) spectrum of 1-(4-chlorophenyl)-5-imino-3-(4-methoxyphenyl)imidazolidine-2,4-dithione

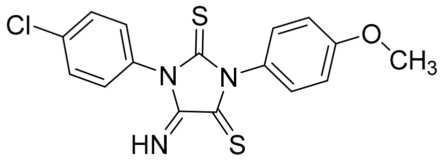

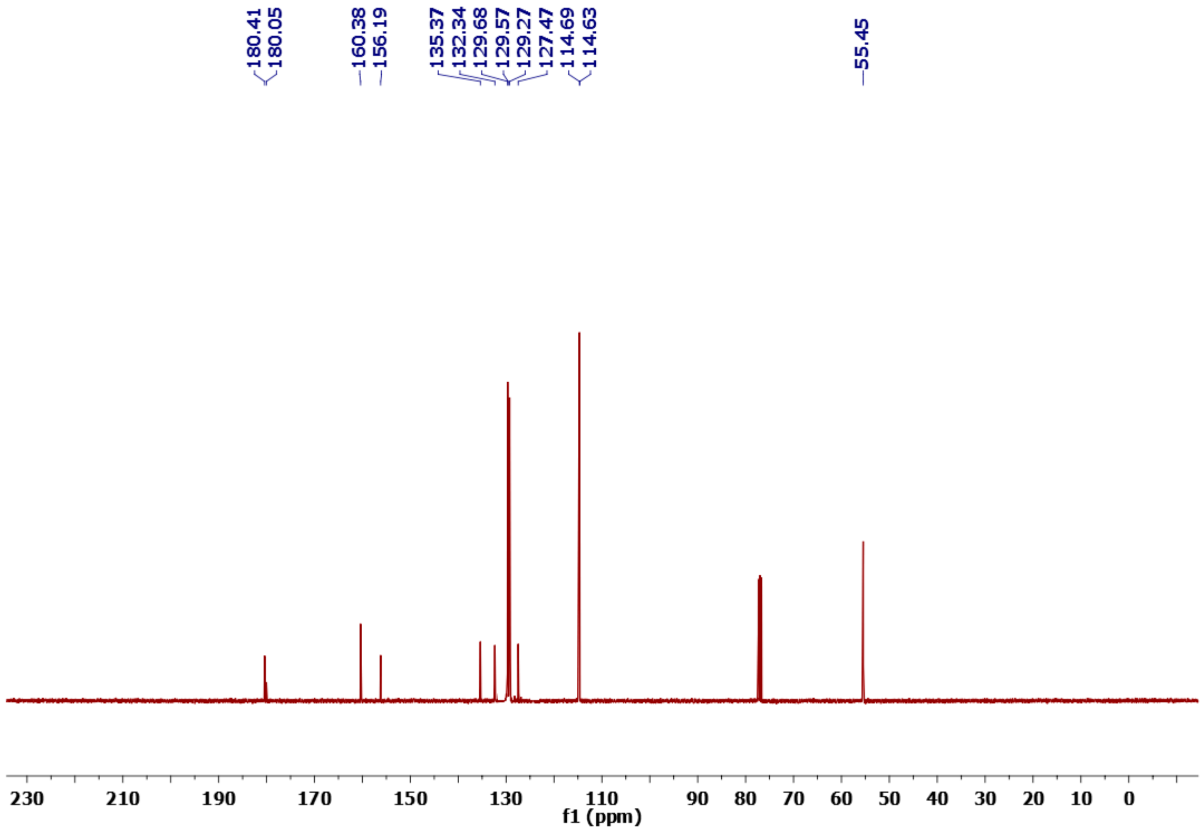

^{13}C-CRAPT NMR ($CDCl_3$) spectrum of 1-(4-chlorophenyl)-5-imino-3-(4-methoxyphenyl)imidazolidine-2,4-dithione

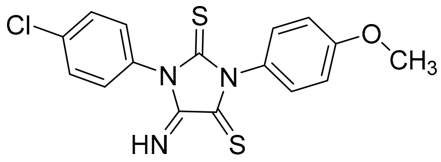

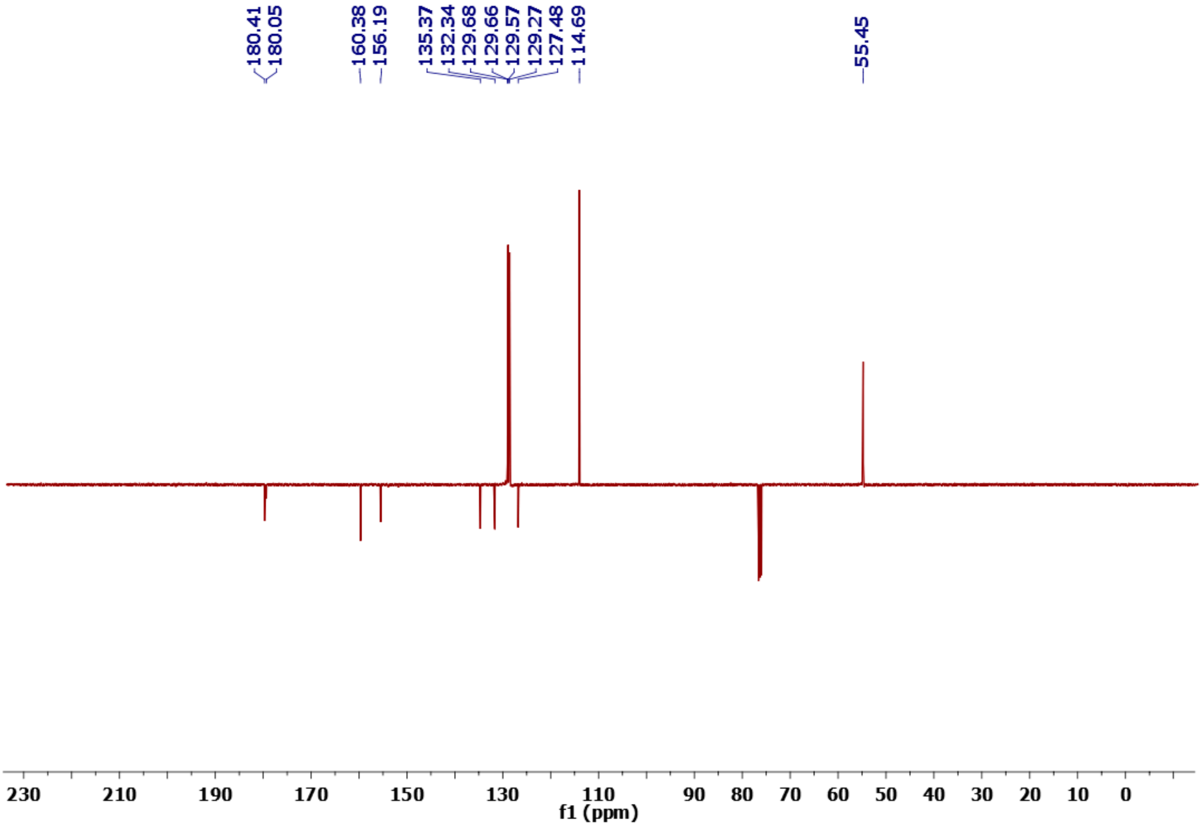

^{1}H-^{1}H-gCOSYAD NMR ($CDCl_3$) spectrum of 1-(4-chlorophenyl)-5-imino-3-(4-methoxyphenyl)imidazolidine-2,4-dithione

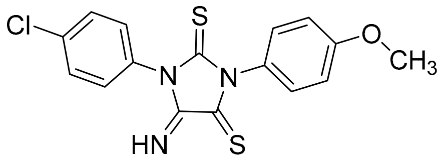

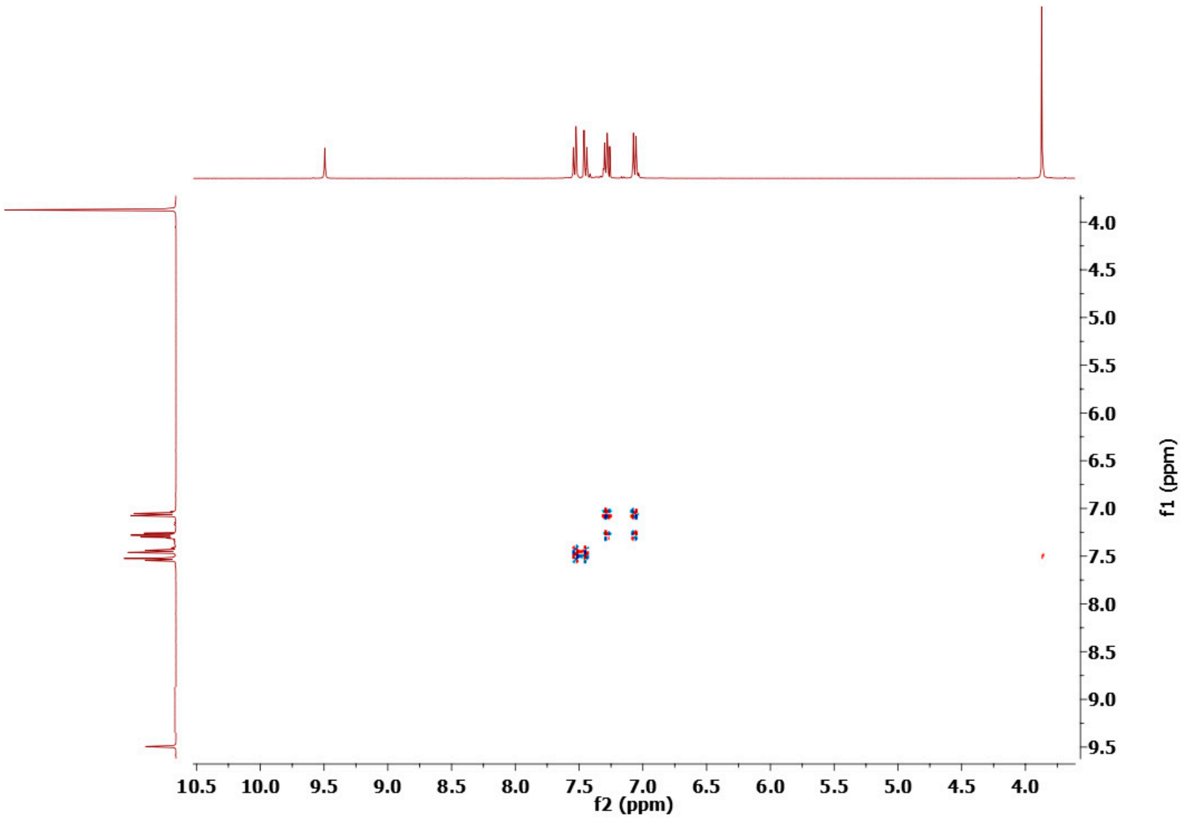

^{1}H-^{13}C-gHSQCAD NMR ($CDCl_3$) spectrum of 1-(4-chlorophenyl)-5-imino-3-(4-methoxyphenyl)imidazolidine-2,4-dithione

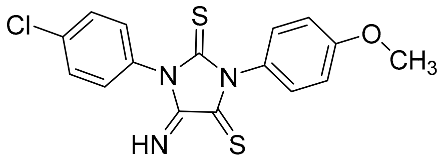

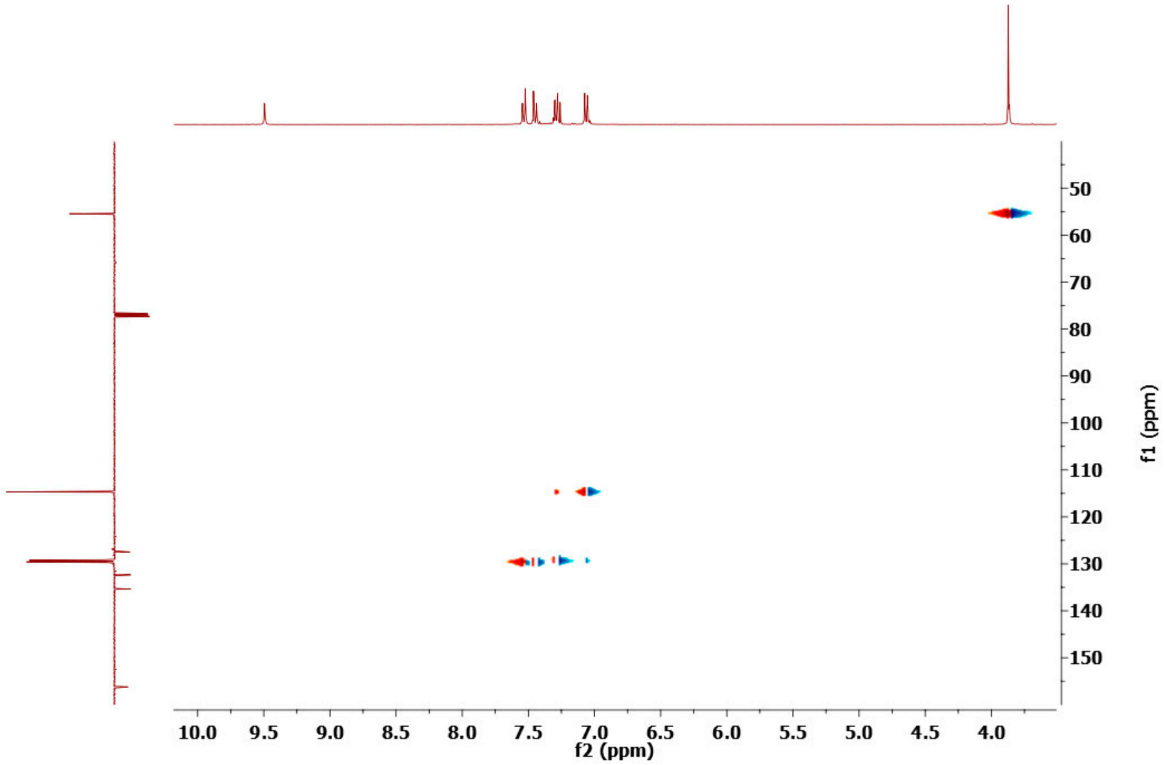

^{1}H-^{13}C-gHMBC NMR ($CDCl_3$) spectrum of 1-(4-chlorophenyl)-5-imino-3-(4-methoxyphenyl)imidazolidine-2,4-dithione

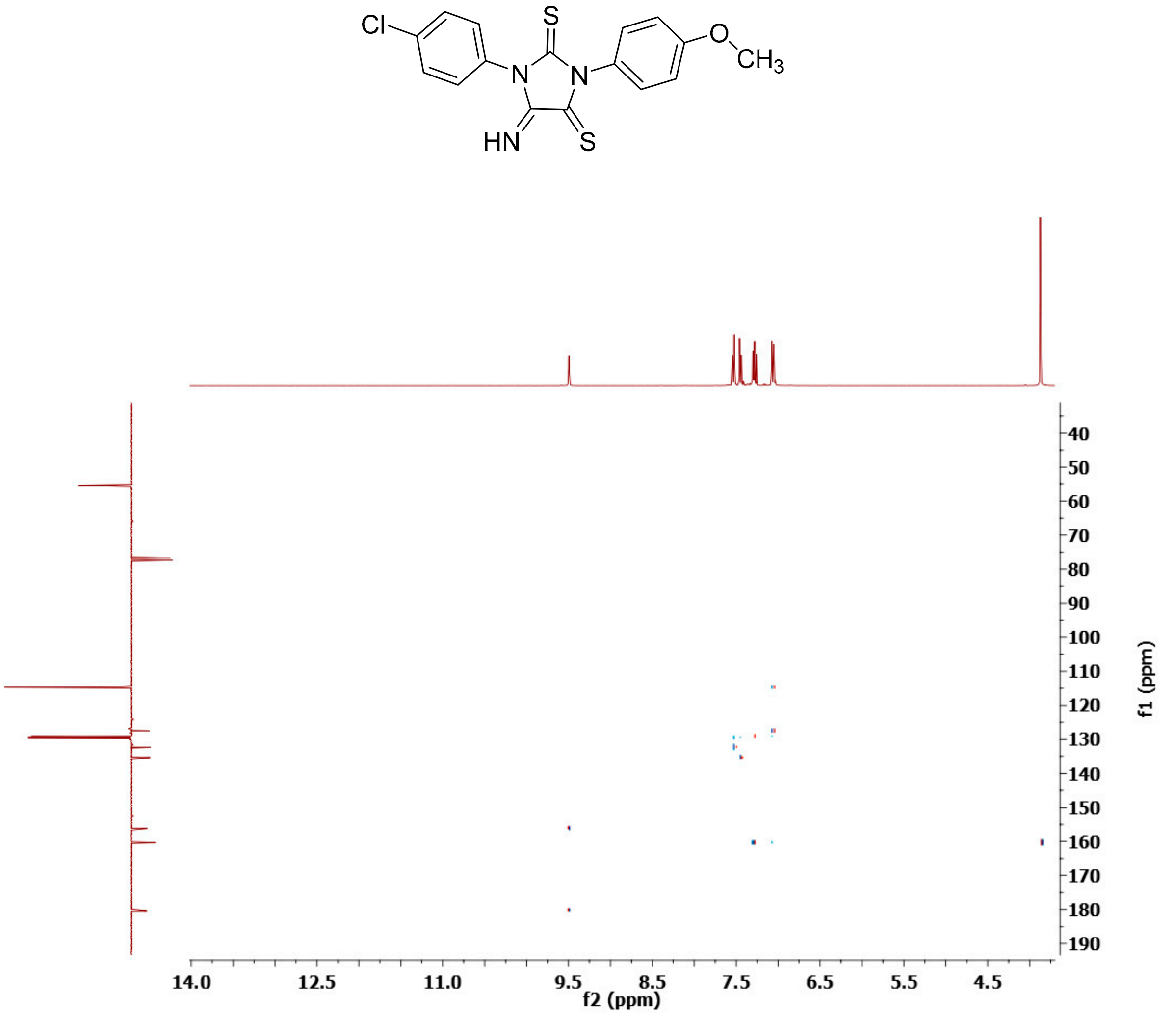

^{1}H NMR ($CDCl_3$) spectrum of 1-(4-fluorophenyl)-5-imino-3-(4-methoxyphenyl)imidazolidine-2,4-dithione (18o')

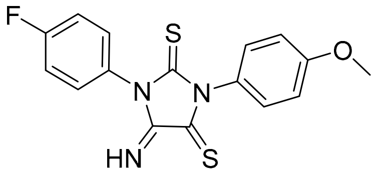

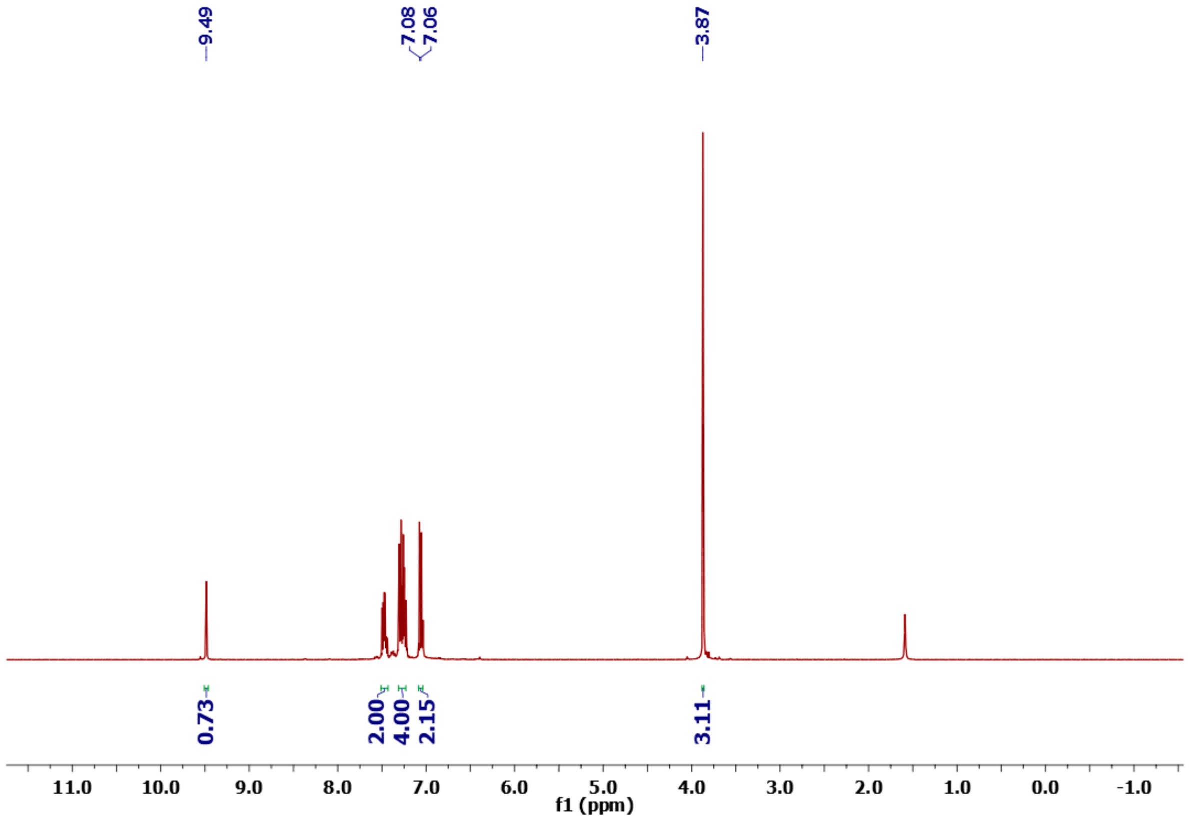

^{13}C NMR ($CDCl_3$) spectrum of 1-(4-fluorophenyl)-5-imino-3-(4-methoxyphenyl)imidazolidine-2,4-dithione

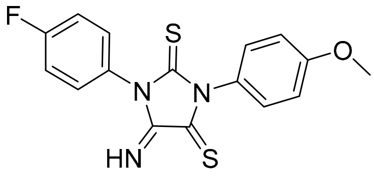

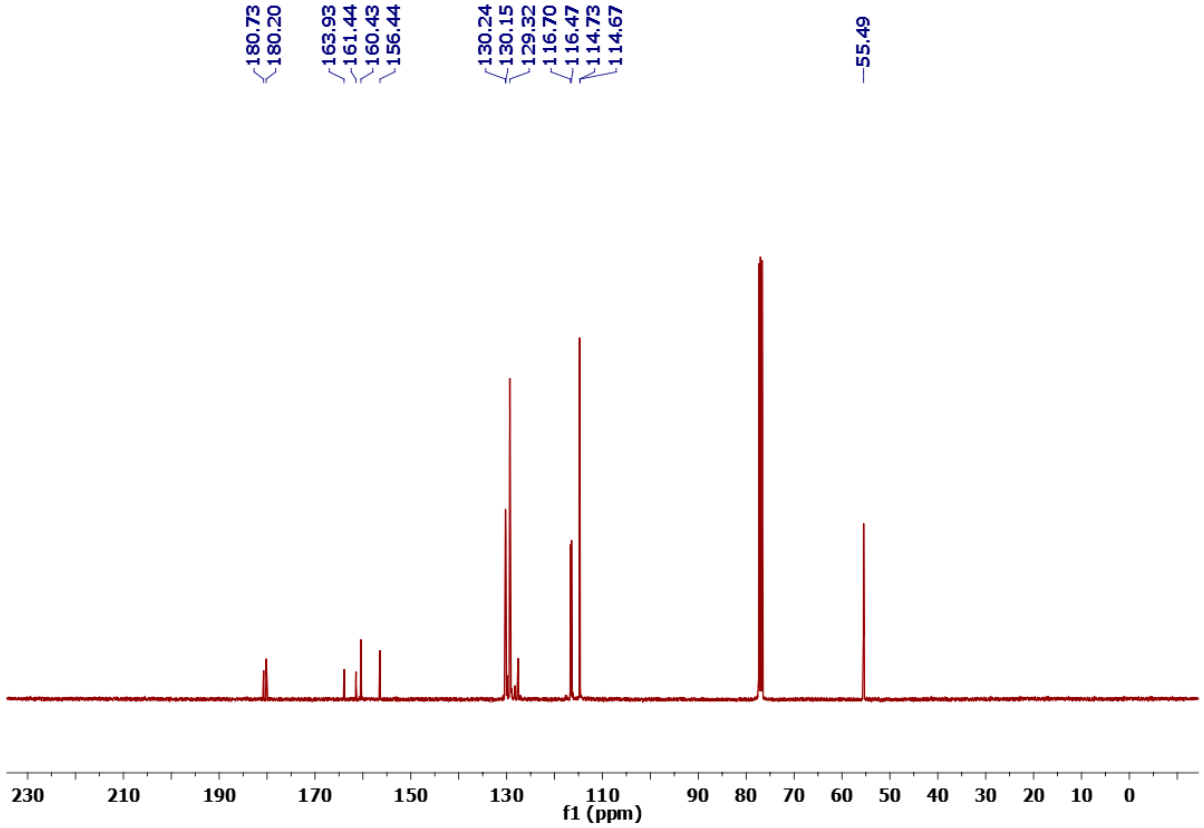

^{13}C-CRAPT NMR ($CDCl_3$) spectrum of 1-(4-fluorophenyl)-5-imino-3-(4-methoxyphenyl)imidazolidine-2,4-dithione

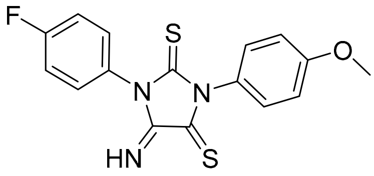

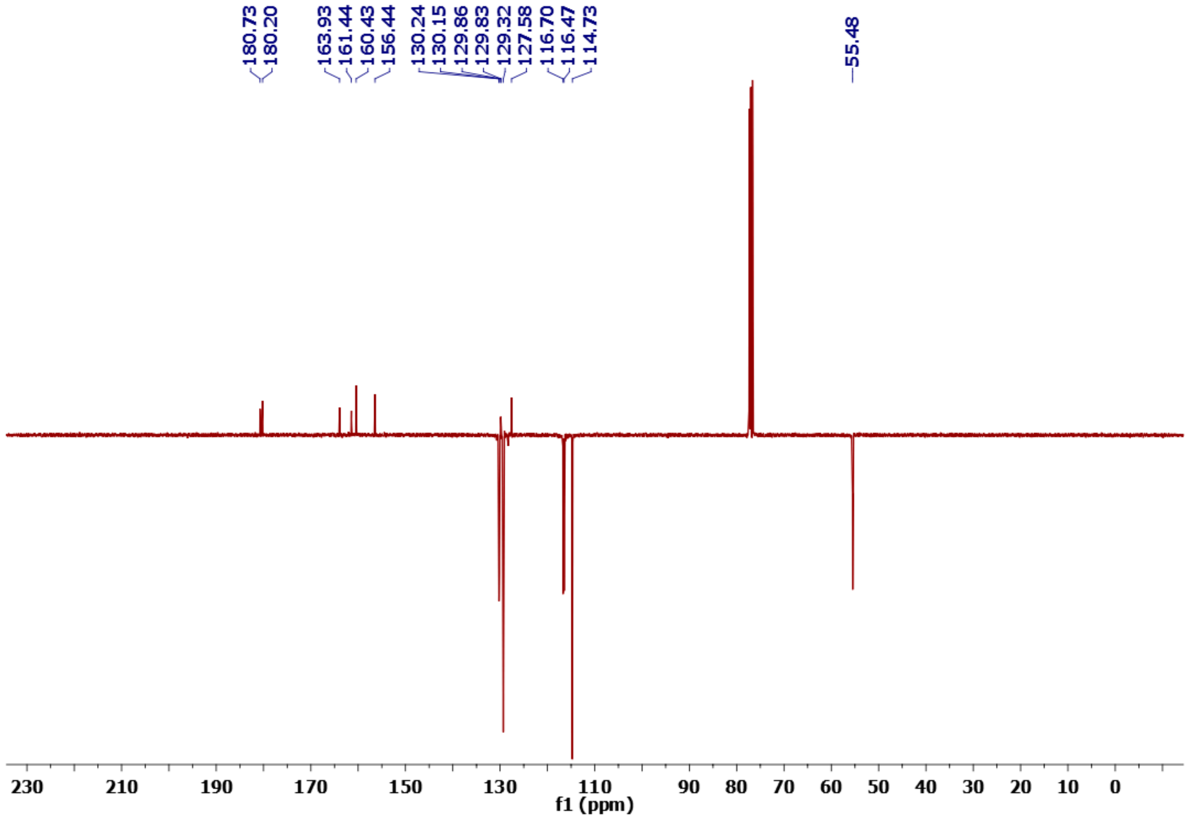

^{1}H-^{1}H-gCOSYAD NMR ($CDCl_3$) spectrum of 1-(4-fluorophenyl)-5-imino-3-(4-methoxyphenyl)imidazolidine-2,4-dithione

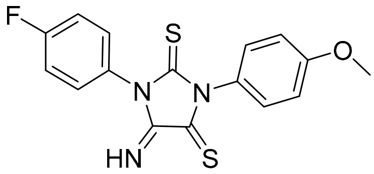

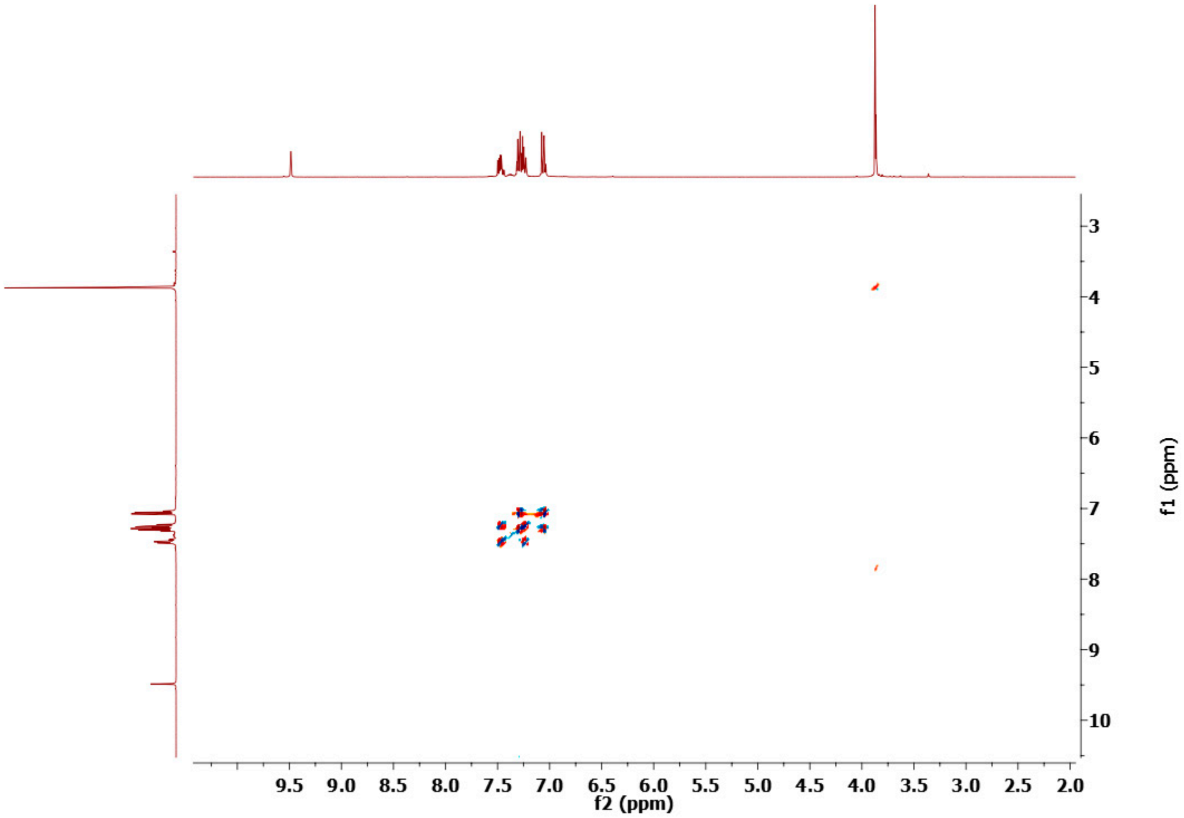

^{1}H-^{13}C-gHSQCAD NMR ($CDCl_3$) spectrum of 1-(4-fluorophenyl)-5-imino-3-(4-methoxyphenyl)imidazolidine-2,4-dithione

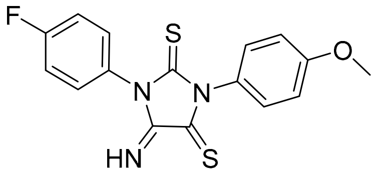

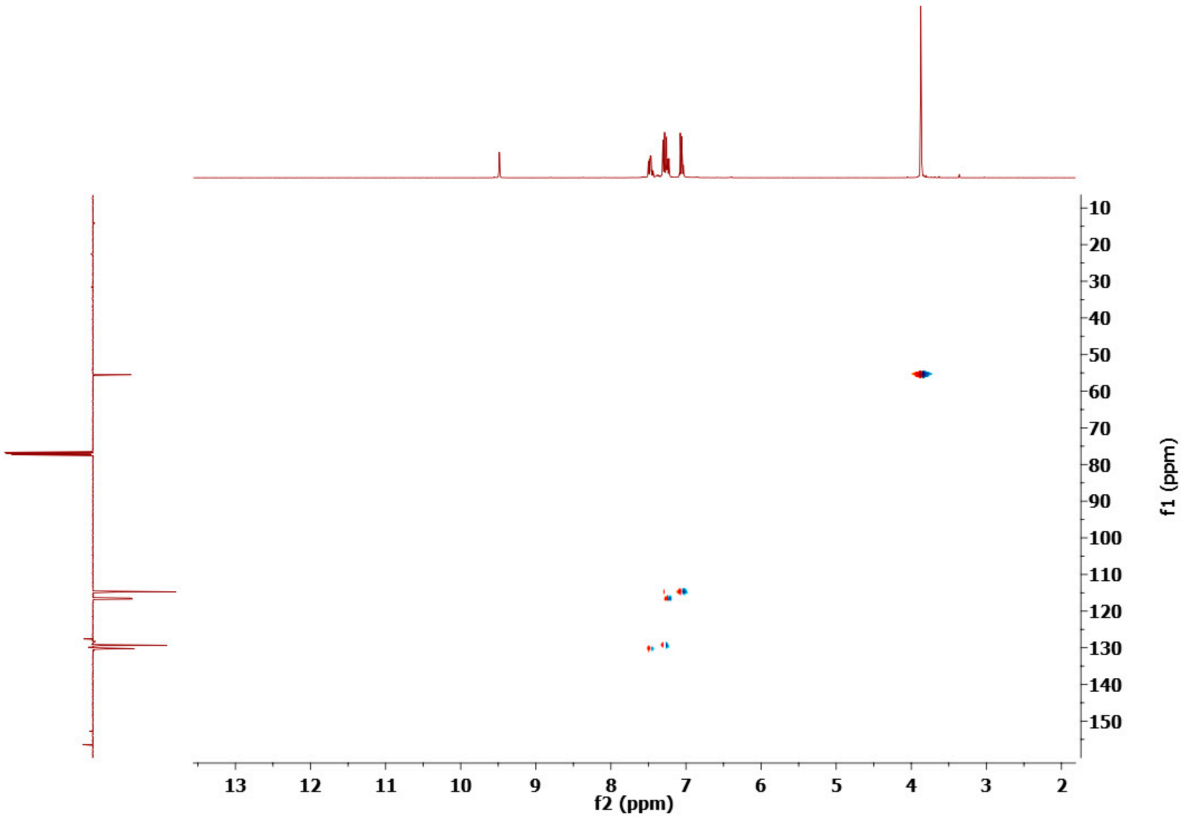

^{1}H-^{13}C-gHMBC NMR ($CDCl_3$) spectrum of 1-(4-fluorophenyl)-5-imino-3-(4-methoxyphenyl)imidazolidine-2,4-dithione

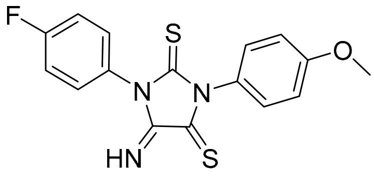

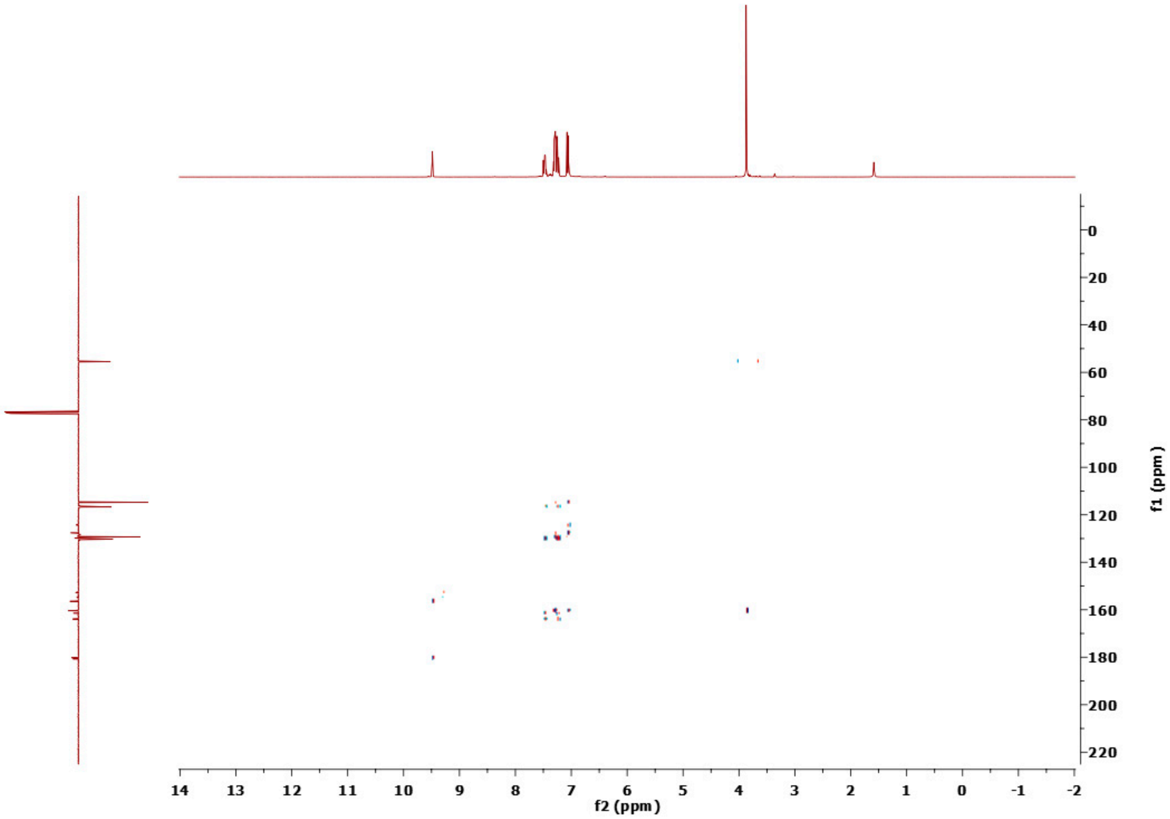

^{1}H-NMR (CDCl$_3$) spectrum of 5-imino-3-(4-methoxyphenyl)-1-(4-nitrophenyl)imidazolidine-2,4-dithione (18p')

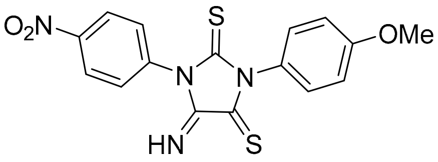

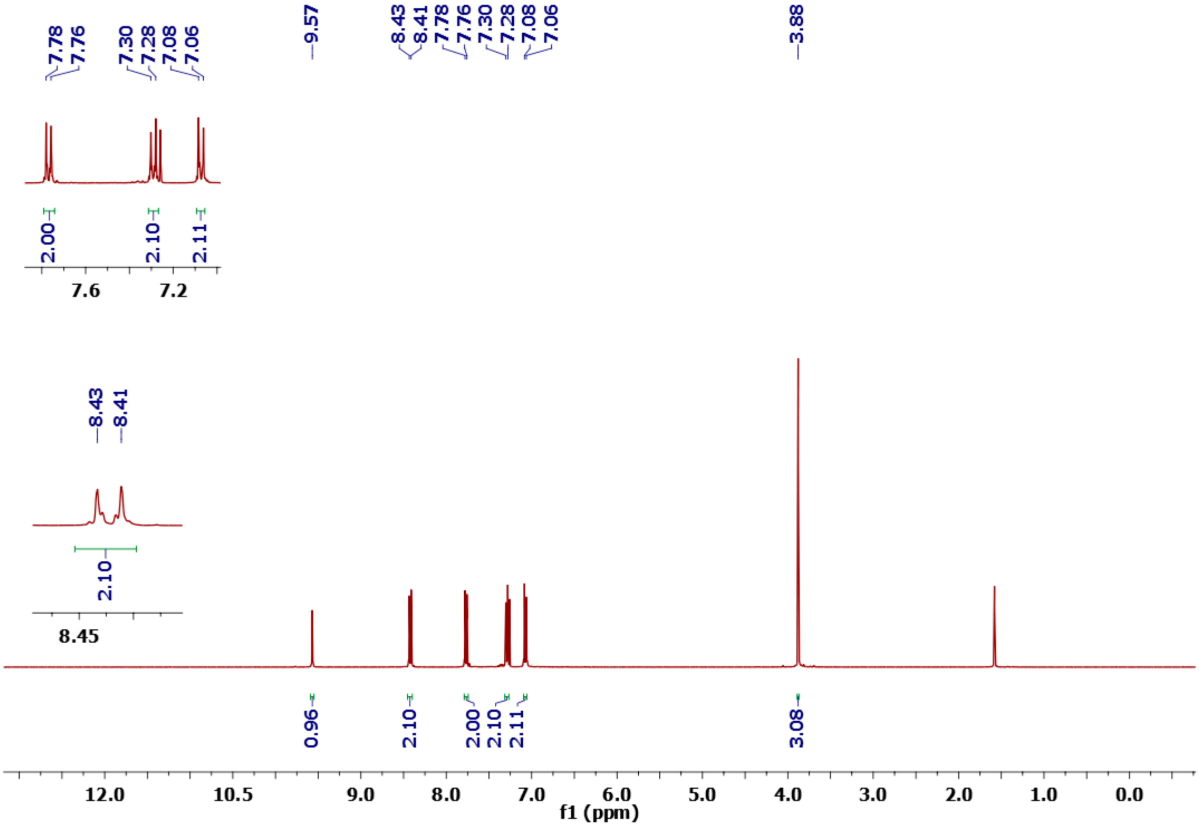

^{13}C NMR ($CDCl_3$) spectrum of 5-imino-3-(4-methoxyphenyl)-1-(4-nitrophenyl)imidazolidine-2,4-dithione

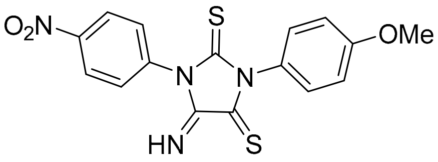

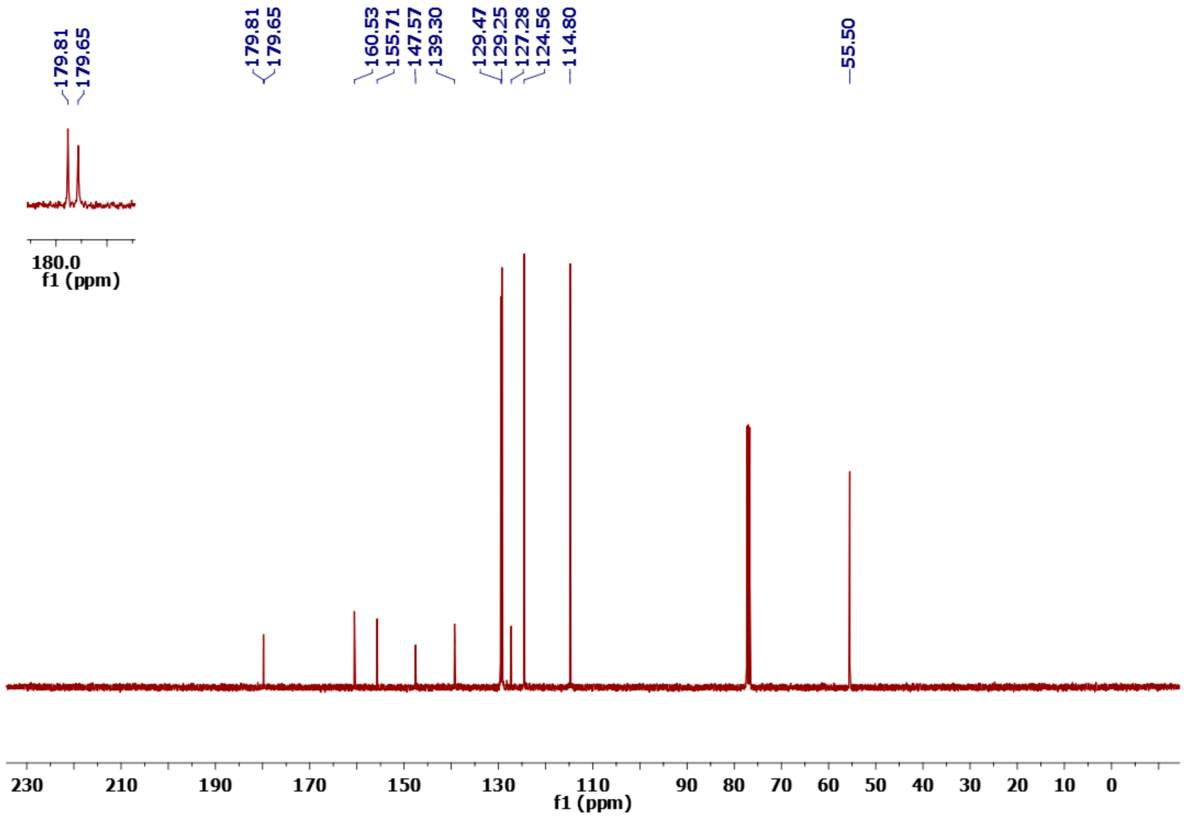

^{13}C-CRAPT NMR ($CDCl_3$) spectrum of 5-imino-3-(4-methoxyphenyl)-1-(4-nitrophenyl)imidazolidine-2,4-dithione

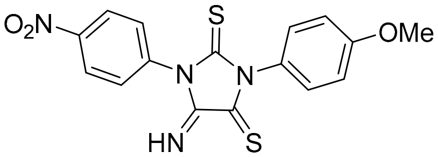

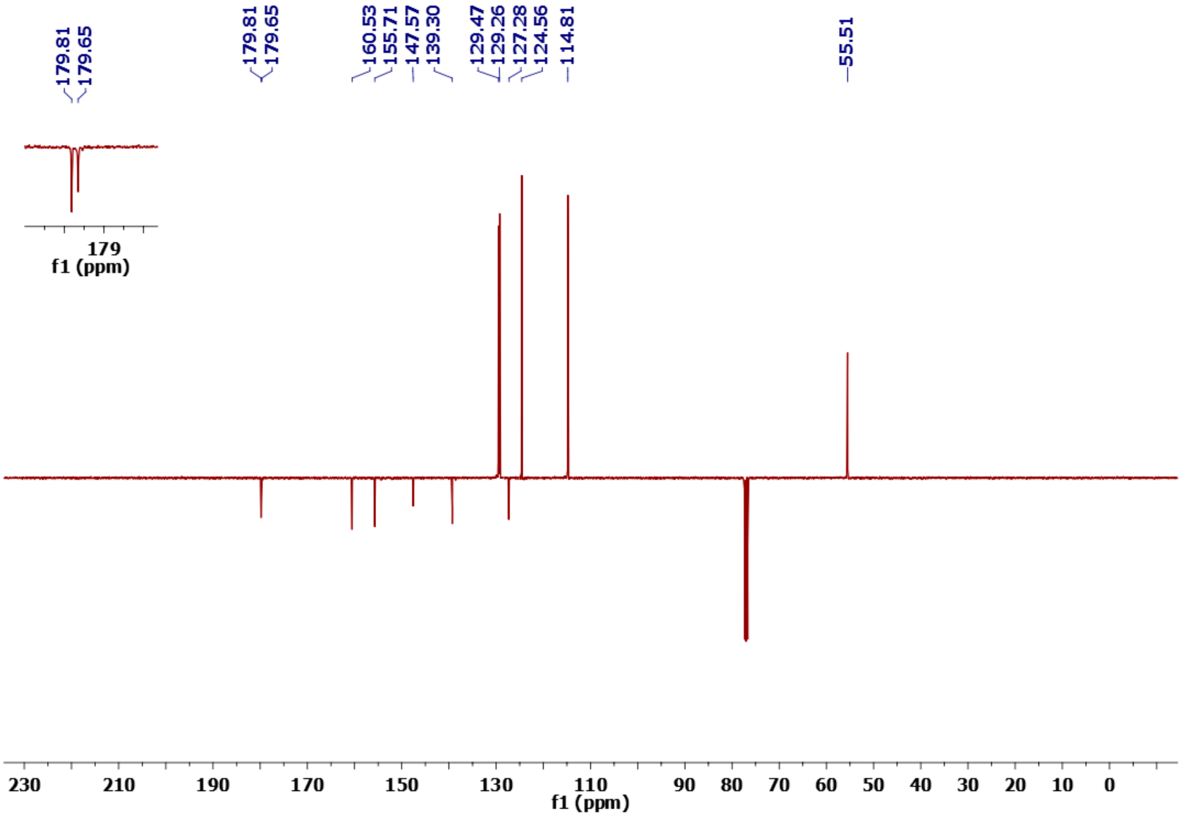

^{1}H-^{1}H-gCOSYAD NMR ($CDCl_3$) spectrum of 5-imino-3-(4-methoxyphenyl)-1-(4-nitrophenyl)imidazolidine-2,4-dithione

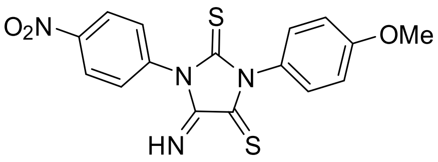

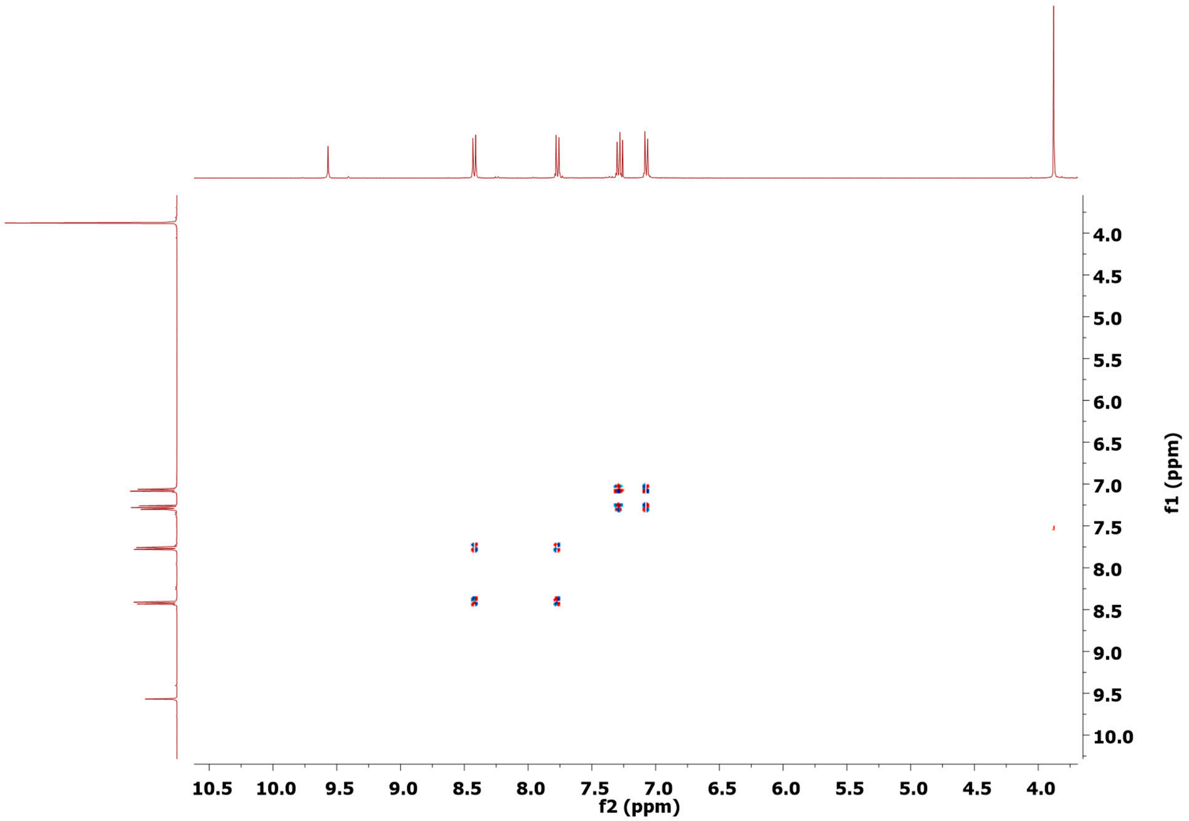

^1H-^{13}C-gHSQCAD NMR ($CDCl_3$) spectrum of 5-imino-3-(4-methoxyphenyl)-1-(4-nitrophenyl)imidazolidine-2,4-dithione

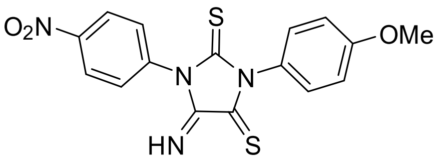

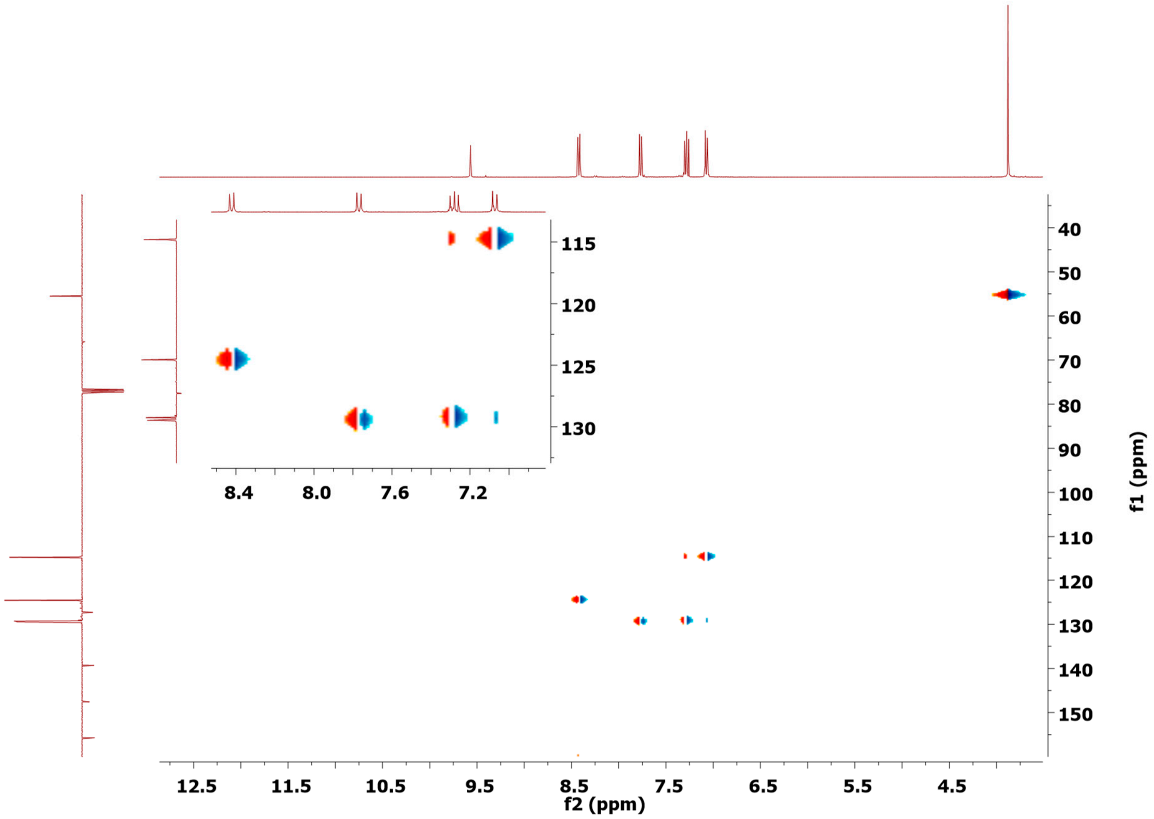

^{1}H-^{13}C-HMBC NMR ($CDCl_3$) spectrum of 5-imino-3-(4-methoxyphenyl)-1-(4-nitrophenyl)imidazolidine-2,4-dithione

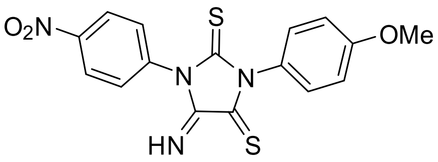

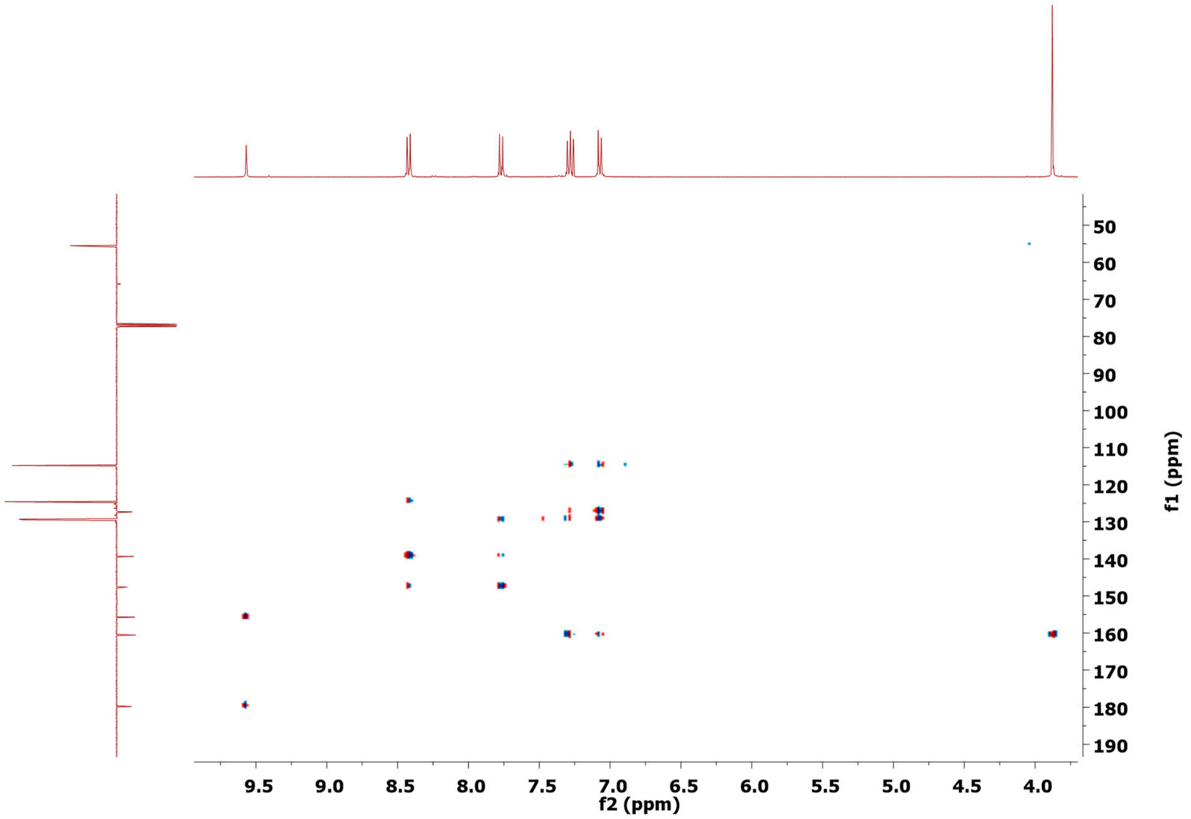

^{1}H NMR (CDCl$_3$) spectrum of 5-imino-1,3-bis(4-methoxyphenyl)imidazolidine-2,4-dithione (18q')

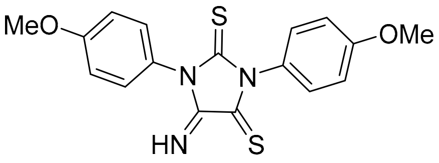

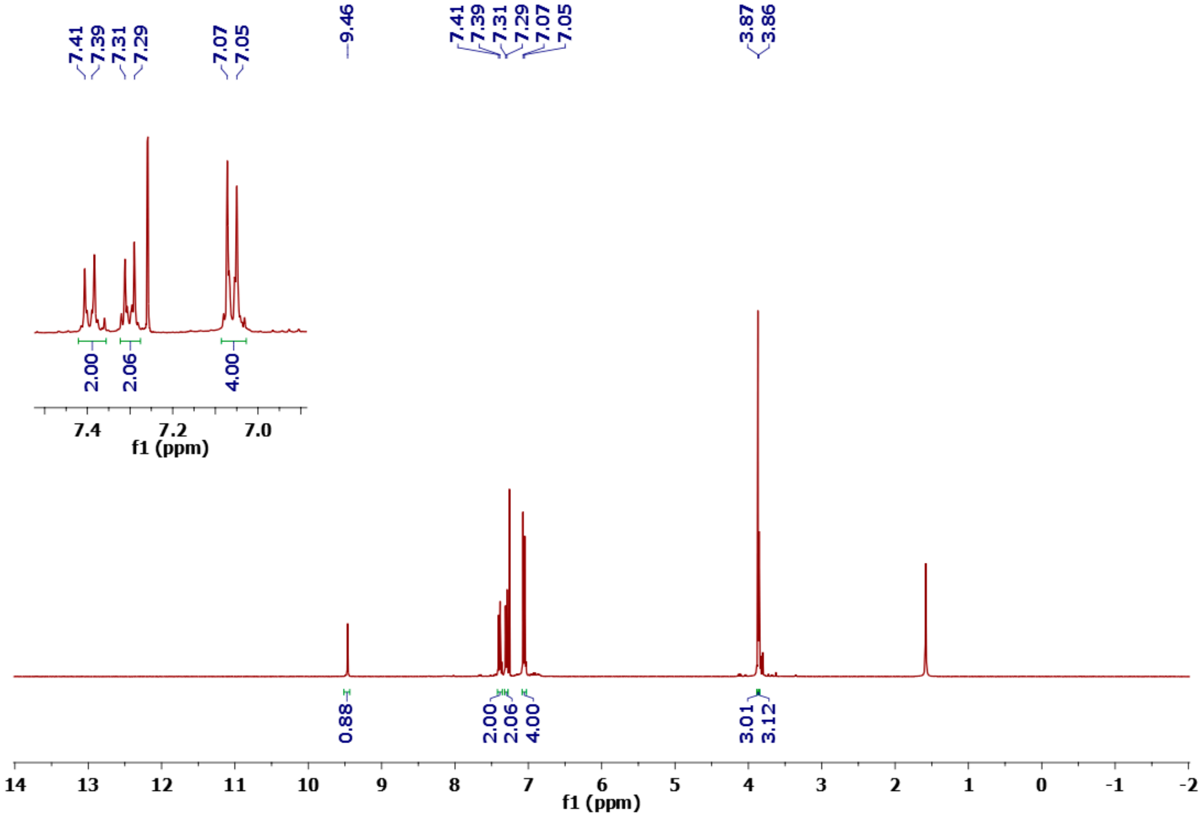

^{13}C NMR ($CDCl_3$) spectrum 5-imino-1,3-bis(4-methoxyphenyl)imidazolidine-2,4-dithione

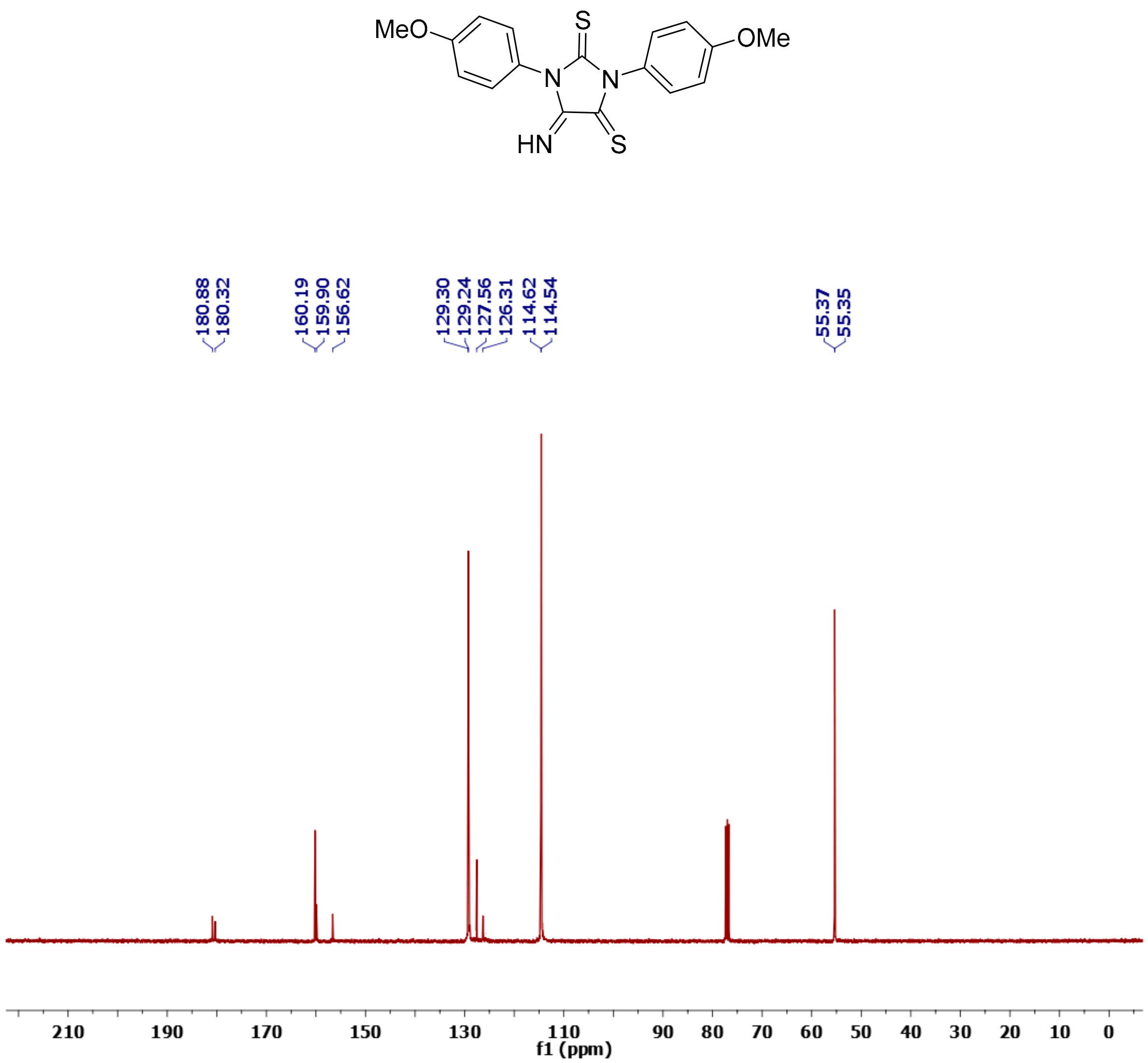

^{13}C-CRAPT NMR ($CDCl_3$) spectrum of 5-imino-1,3-bis(4-methoxyphenyl)imidazolidine-2,4-dithione

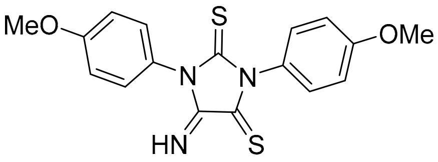

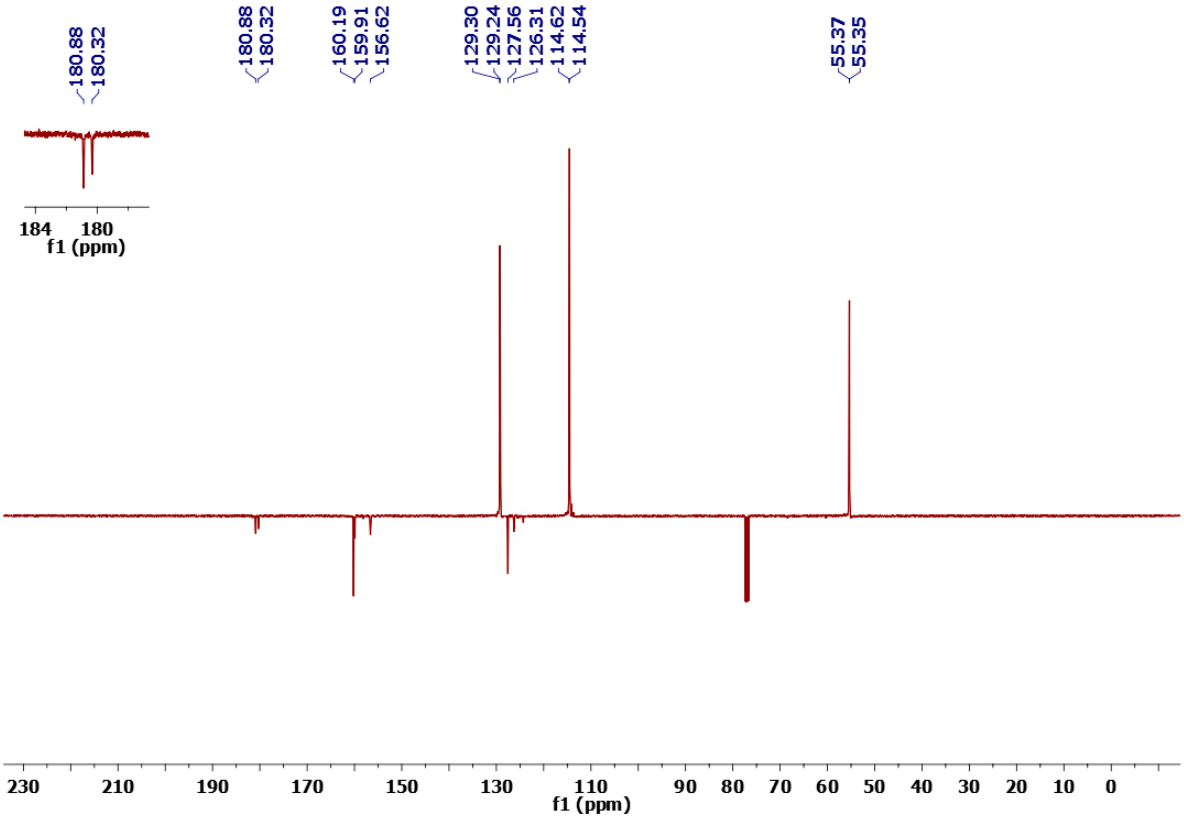

^{1}H-^{1}H-gCOSYAD NMR ($CDCl_3$) spectrum of 5-imino-1,3-bis(4-methoxyphenyl)imidazolidine-2,4-dithione

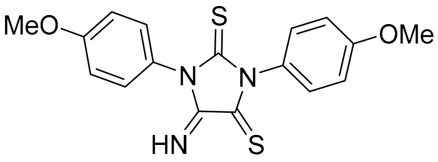

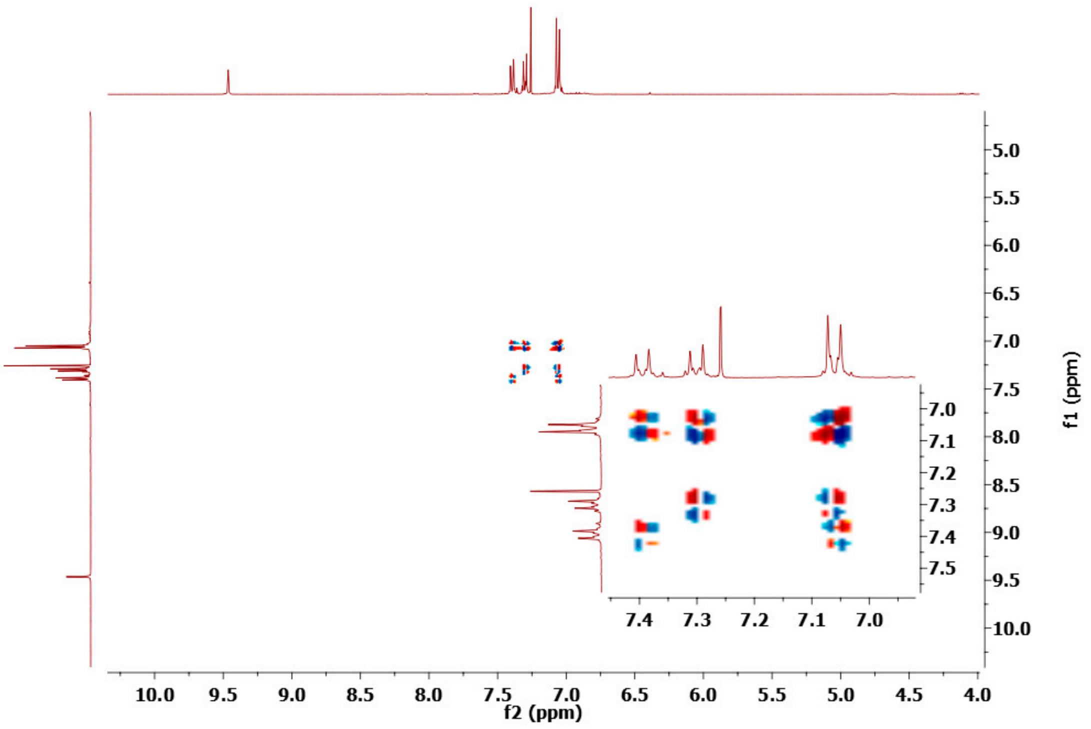

^{1}H-^{13}C-gHSQCAD NMR ($CDCl_3$) spectrum of 5-imino-1,3-bis(4-methoxyphenyl)imidazolidine-2,4-dithione

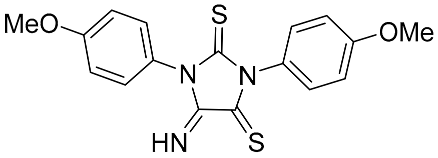

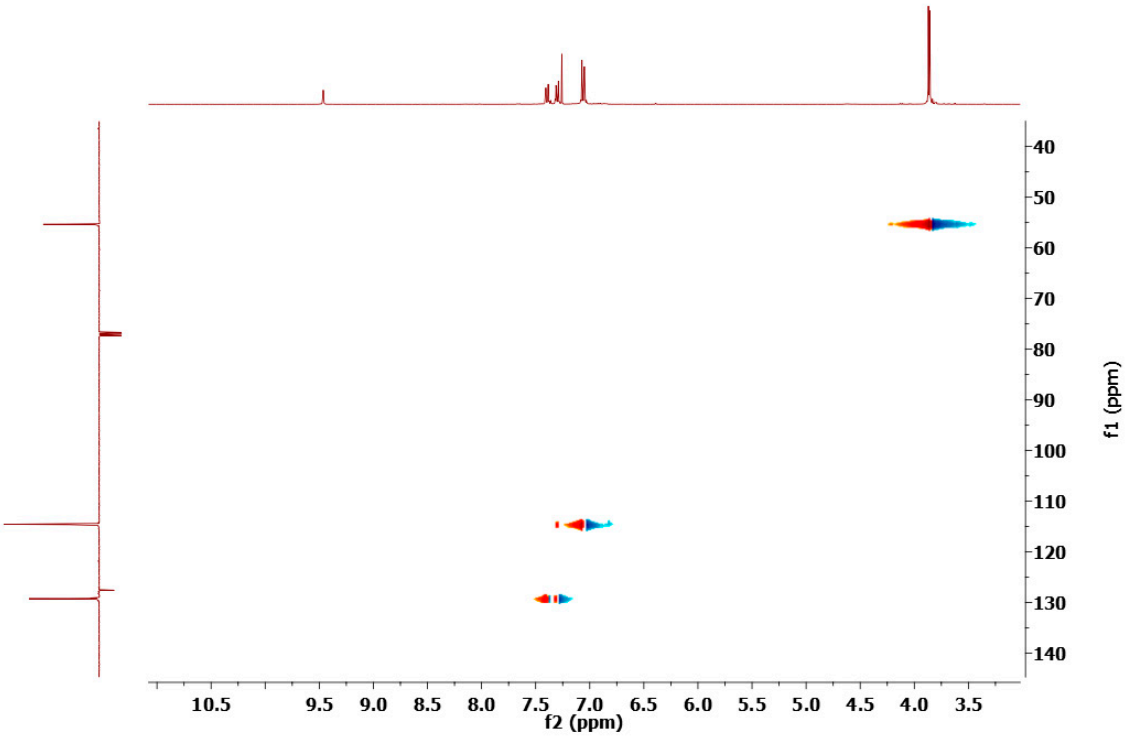

^{1}H-^{13}C-gHMBC NMR ($CDCl_3$) spectrum 5-imino-1,3-bis(4-methoxyphenyl)imidazolidine-2,4-dithione

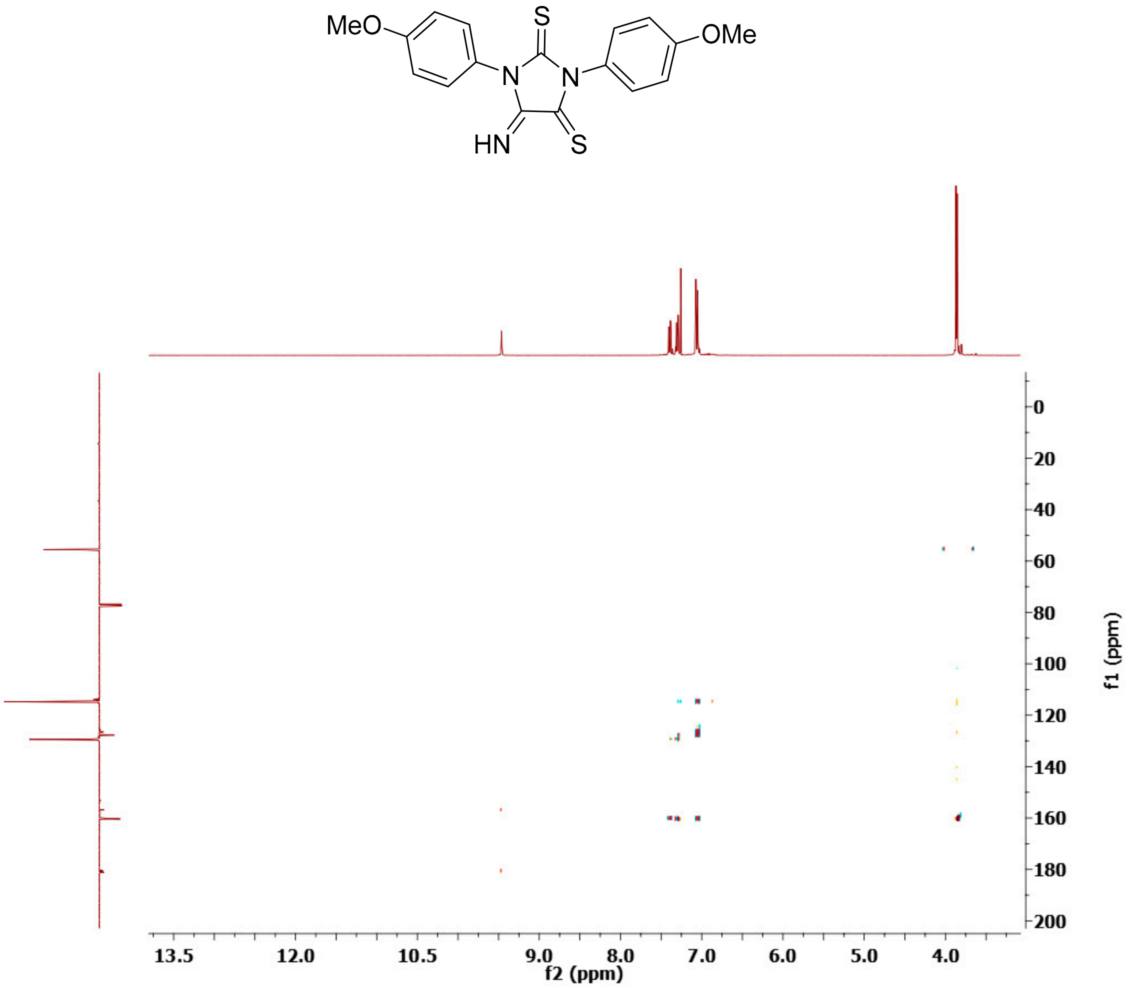

^{1}H-NMR ($CDCl_3$) spectrum of (5-imino-2,4-dithioxo-3-(4-(trifluoromethyl)phenyl)imidazolidin-1-yl)(phenyl)methanone (18r')

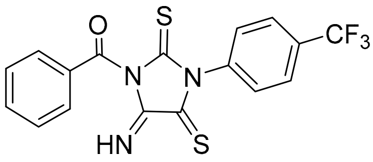

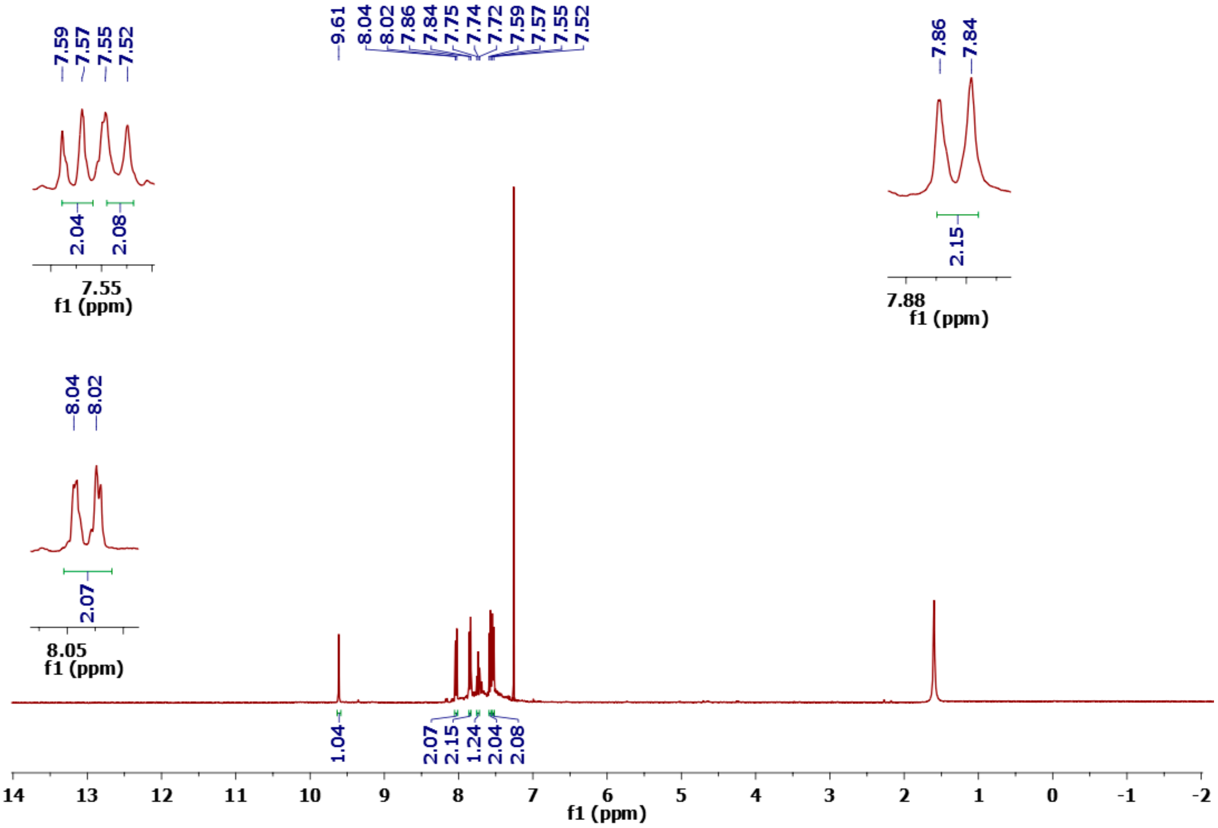

^{13}C NMR ($CDCl_3$) spectrum of (5-imino-2,4-dithioxo-3-(4-(trifluoromethyl)phenyl)imidazolidin-1-yl)(phenyl)methanone

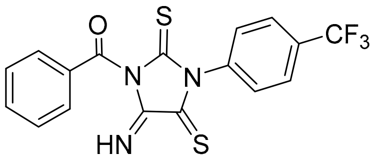

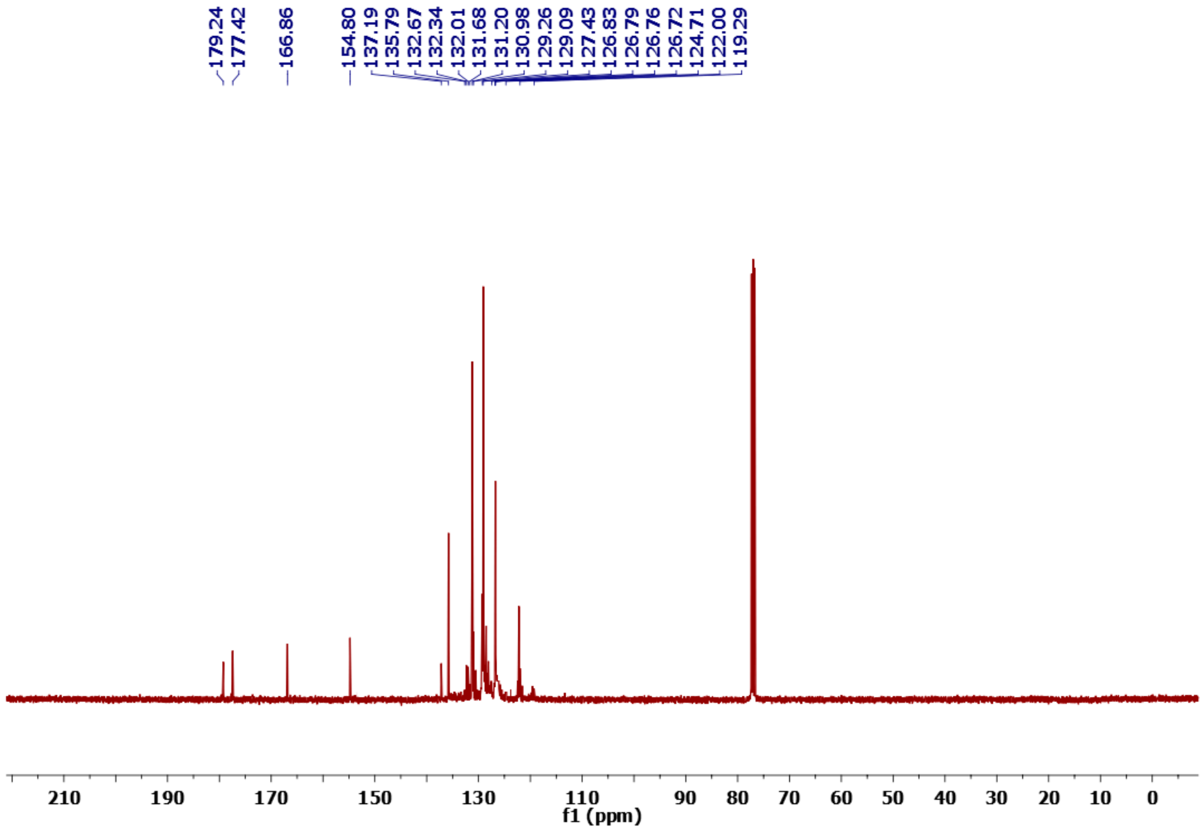

^{13}C-CRAPT NMR ($CDCl_3$) spectrum of (5-imino-2,4-dithioxo-3-(4-(trifluoromethyl)phenyl)imidazolidin-1-yl)(phenyl)methanone

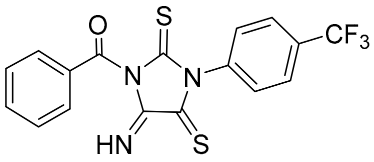

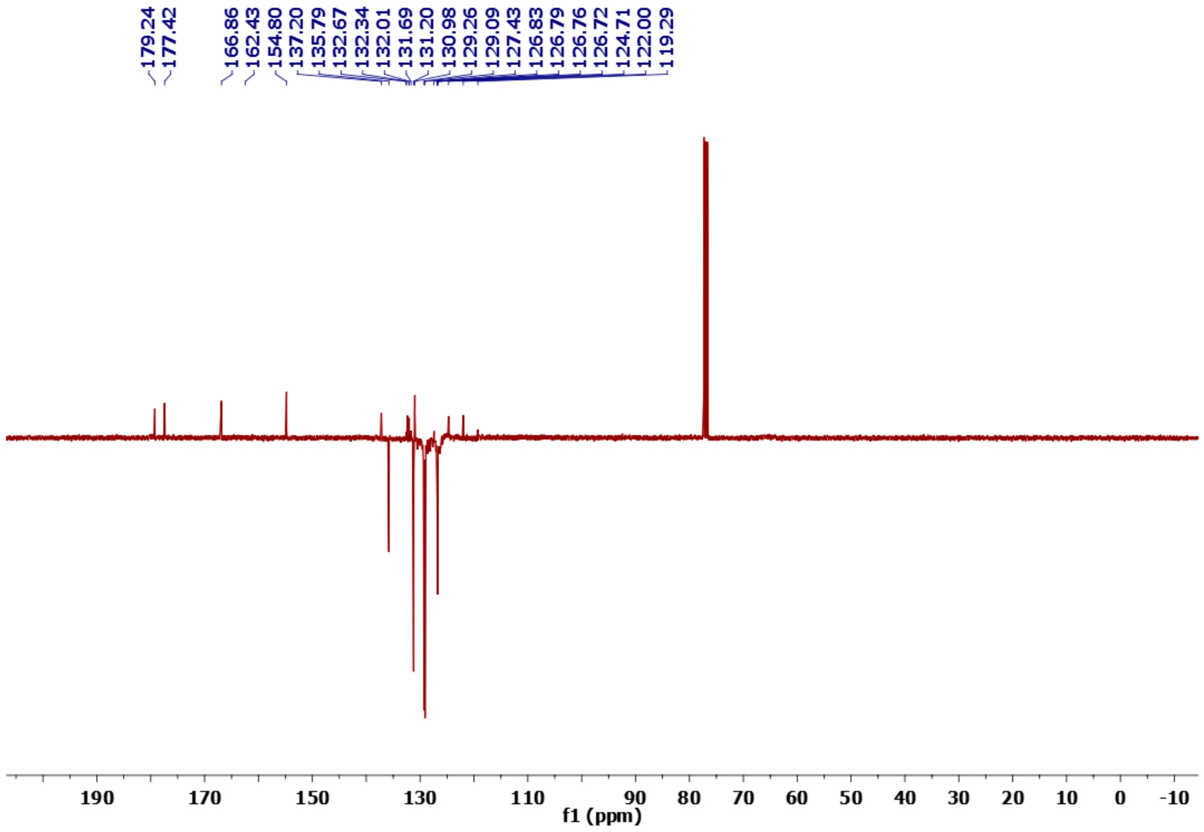

^{1}H-^{1}H-gCOSYAD NMR ($CDCl_3$) spectrum of (5-imino-2,4-dithioxo-3-(4-(trifluoromethyl)phenyl)imidazolidin-1-yl)(phenyl)methanone

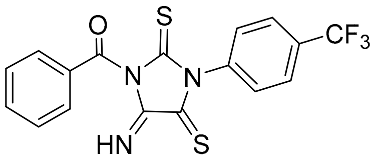

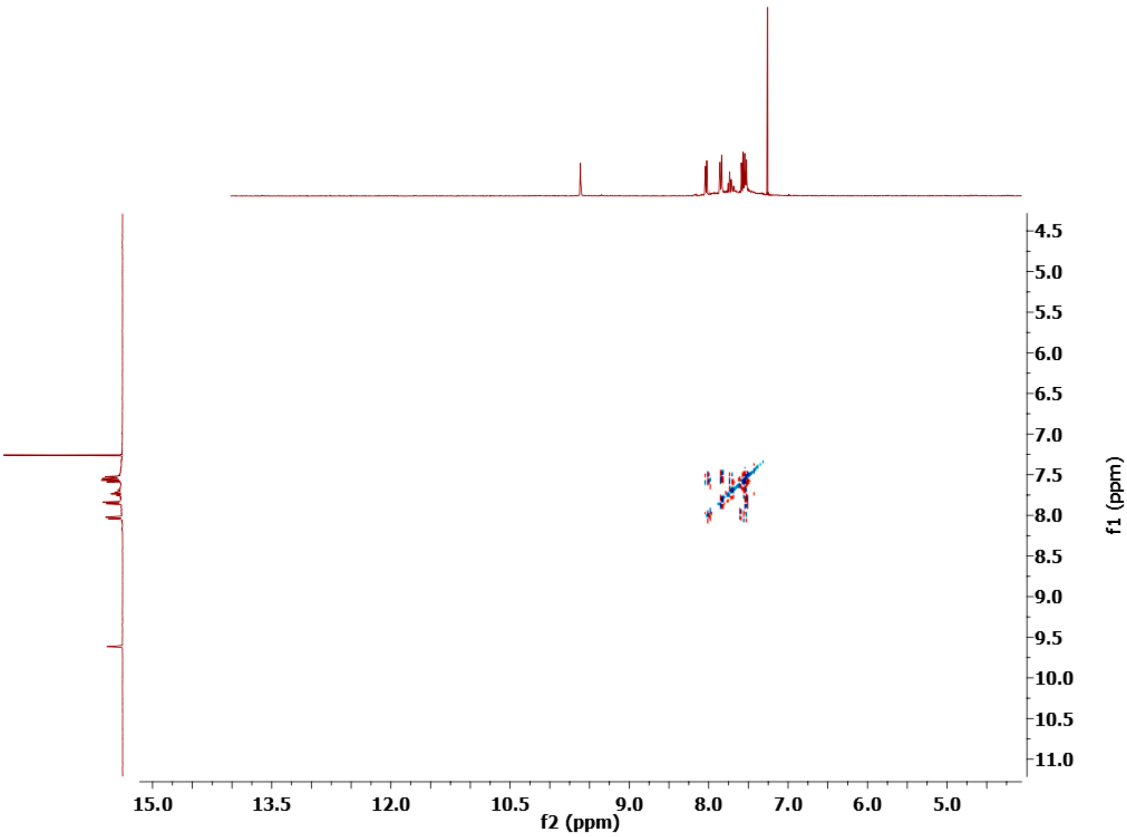

^{1}H-^{13}C-gHSQCAD NMR ($CDCl_3$) spectrum of (5-imino-2,4-dithioxo-3-(4-(trifluoromethyl)phenyl)imidazolidin-1-yl)(phenyl)methanone

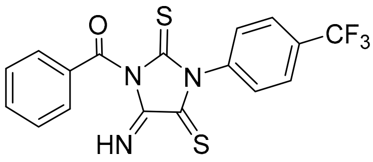

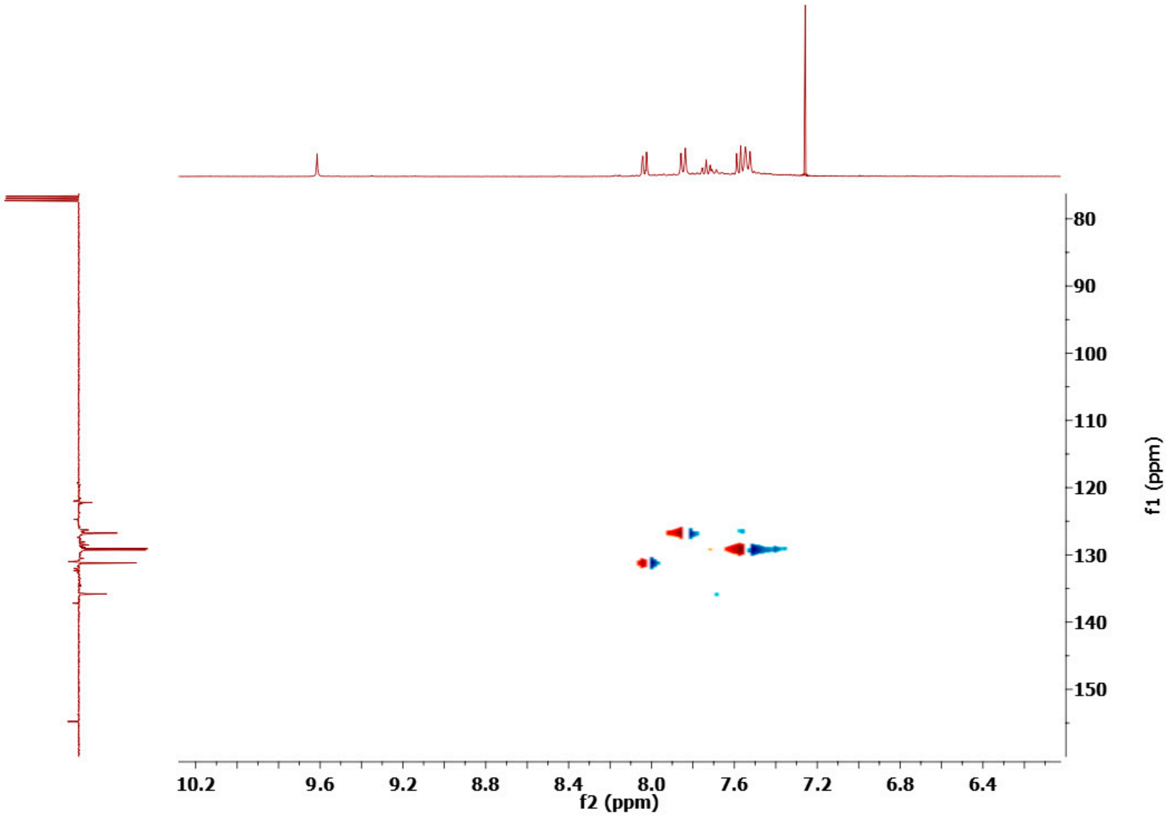

^{1}H-^{13}C-HMBC NMR ($CDCl_3$) spectrum of (5-imino-2,4-dithioxo-3-(4-(trifluoromethyl)phenyl)imidazolidin-1-yl)(phenyl)methanone

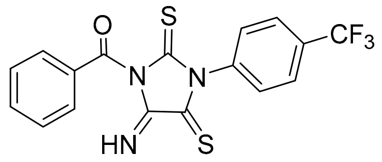

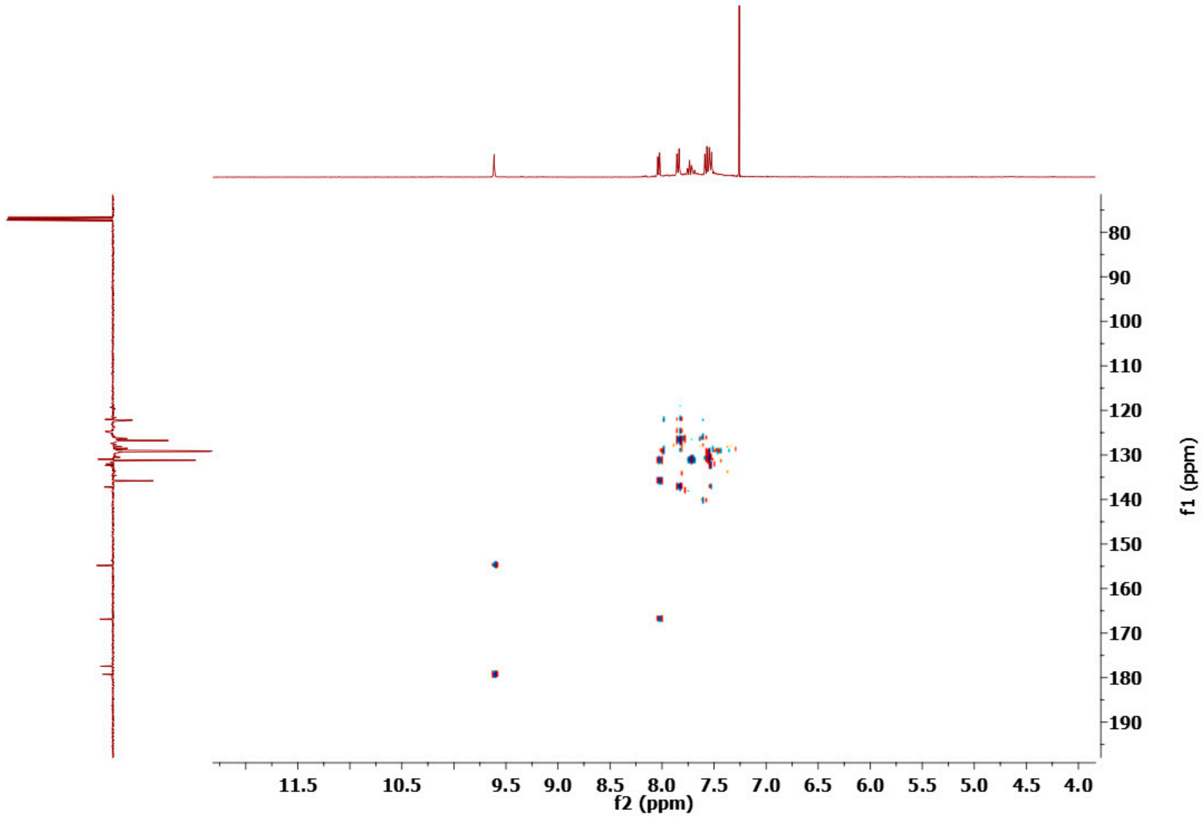

^{1}H NMR ($CDCl_3$) spectrum of 5-imino-3-phenyl-1-(4-(trifluoromethyl)phenyl)imidazolidine-2,4-dithione (18s')

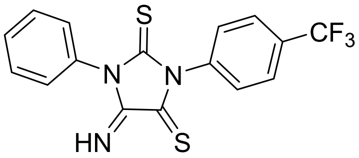

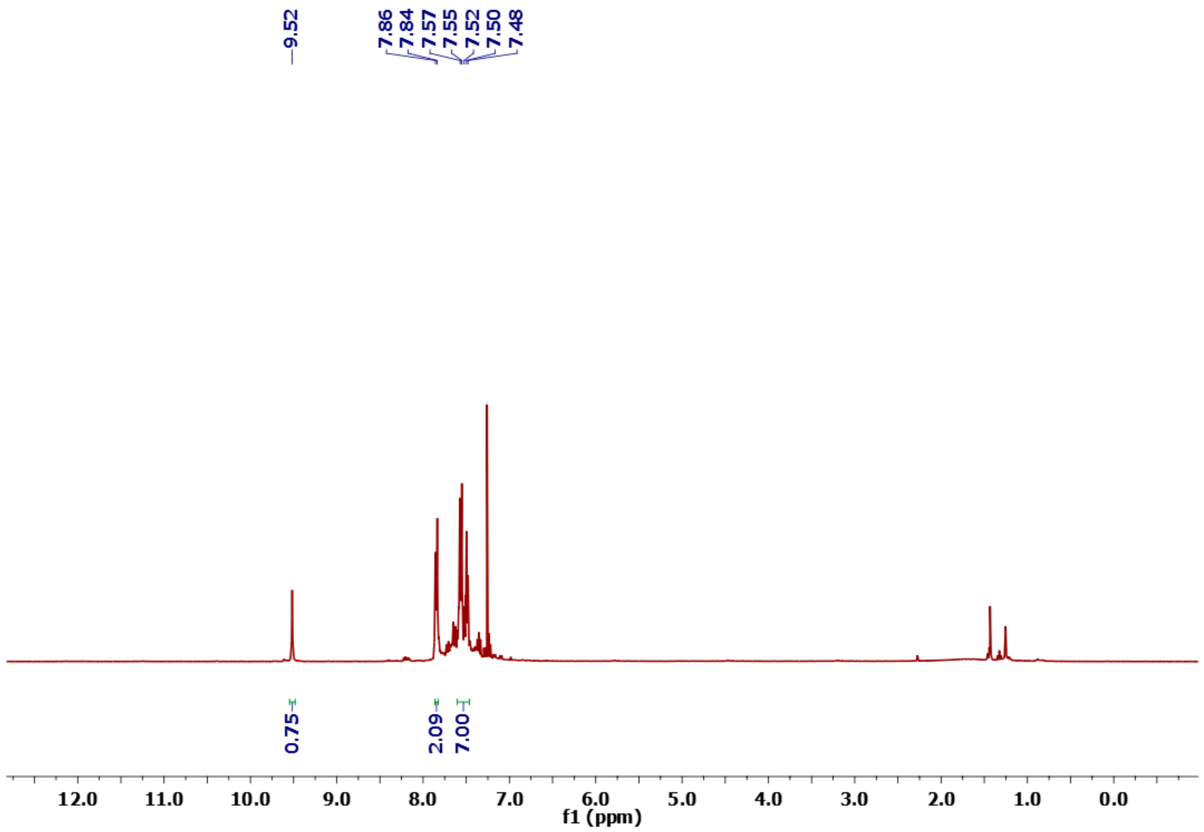

^{13}C-CRAPT NMR ($CDCl_3$) spectrum of 5-imino-3-phenyl-1-(4-(trifluoromethyl)phenyl)imidazolidine-2,4-dithione

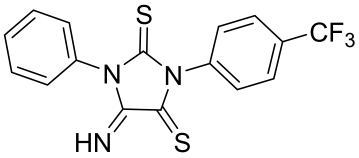

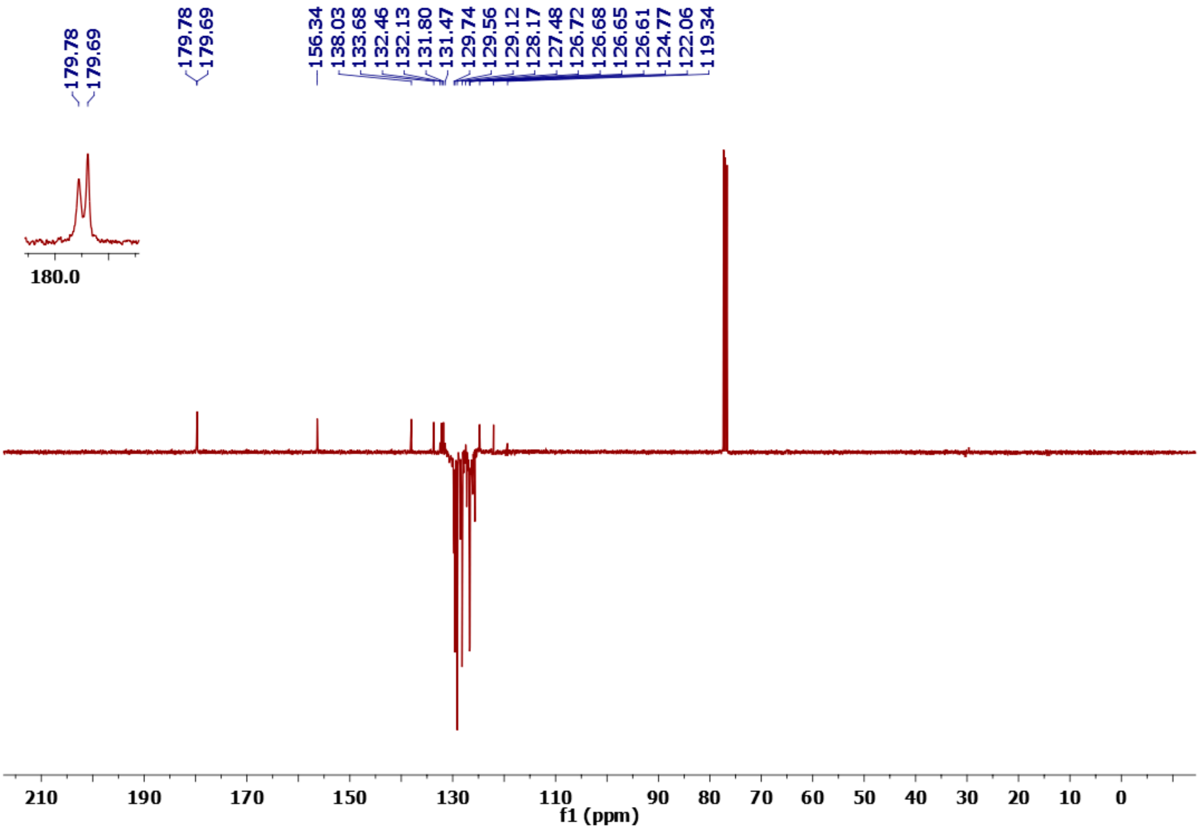

^{1}H-^{1}H-gCOSYAD NMR ($CDCl_3$) spectrum of 5-imino-3-phenyl-1-(4-(trifluoromethyl)phenyl)imidazolidine-2,4-dithione

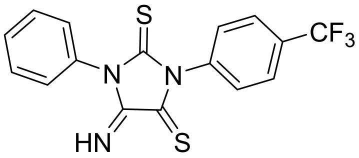

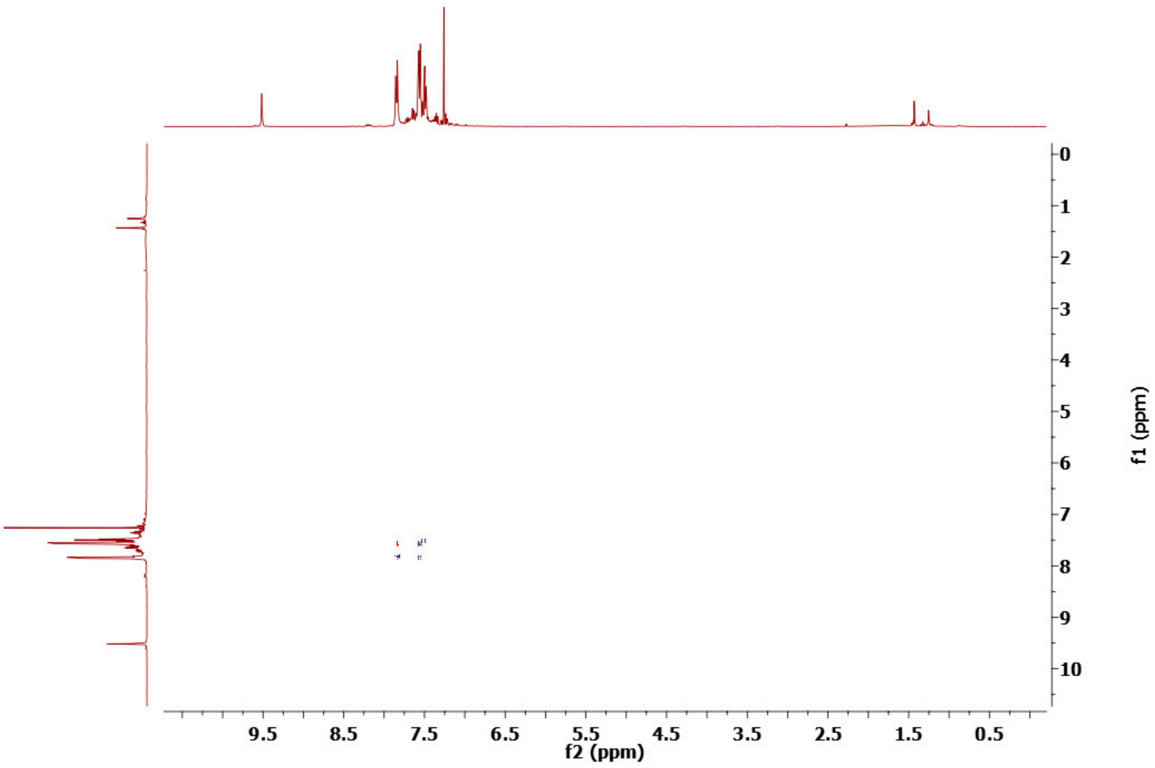

^{1}H NMR ($CDCl_3$) spectrum of 5-imino-1-(p-tolyl)-3-(4-(trifluoromethyl)phenyl)imidazolidine-2,4-dithione (18t')

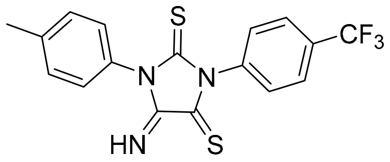

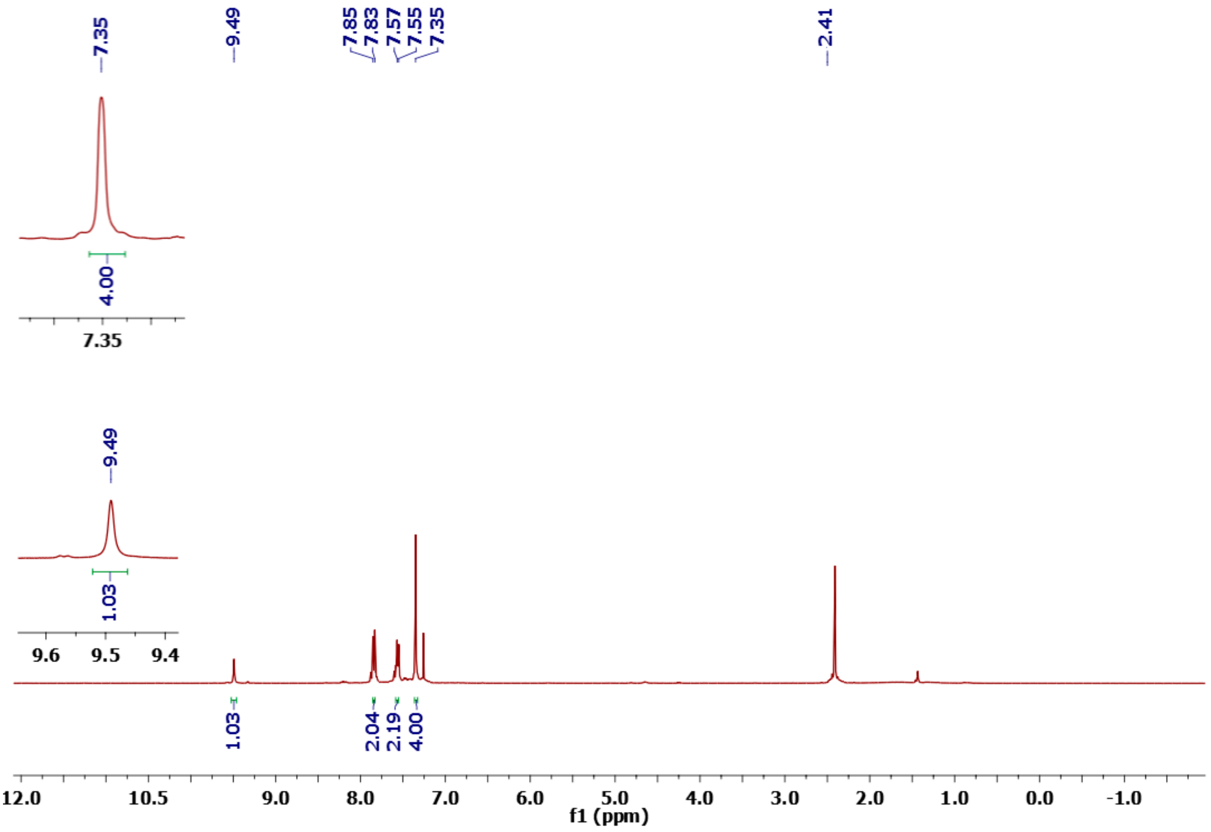

^{13}C-CRAPT NMR ($CDCl_3$) spectrum of 5-imino-1-(p-tolyl)-3-(4-(trifluoromethyl)phenyl)imidazolidine-2,4-dithione

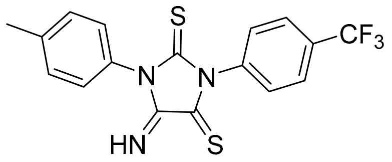

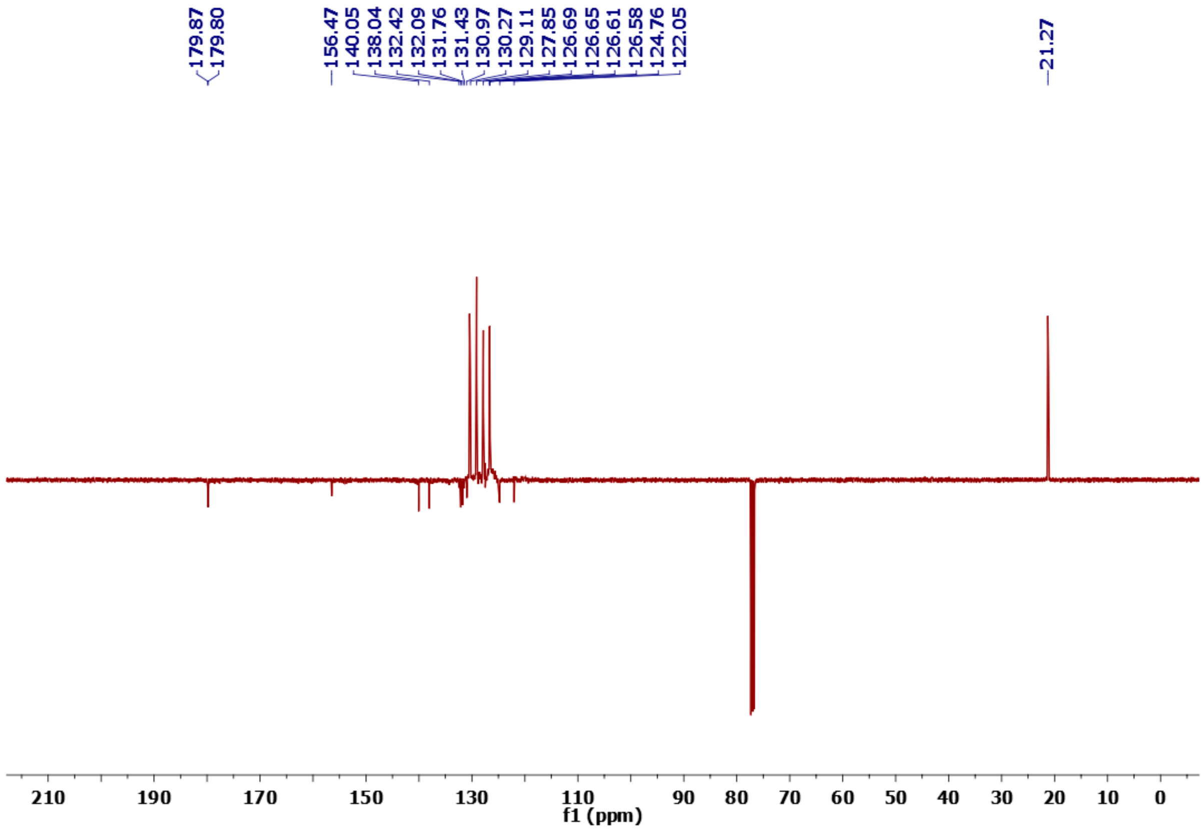

^{1}H NMR (CDCl$_3$) spectrum of 1-(2-fluorophenyl)-5-imino-3-(4-(trifluoromethyl)phenyl)imidazolidine-2,4-dithione (18u')

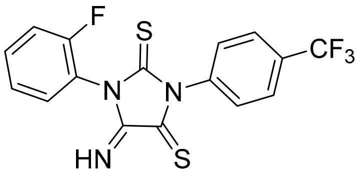

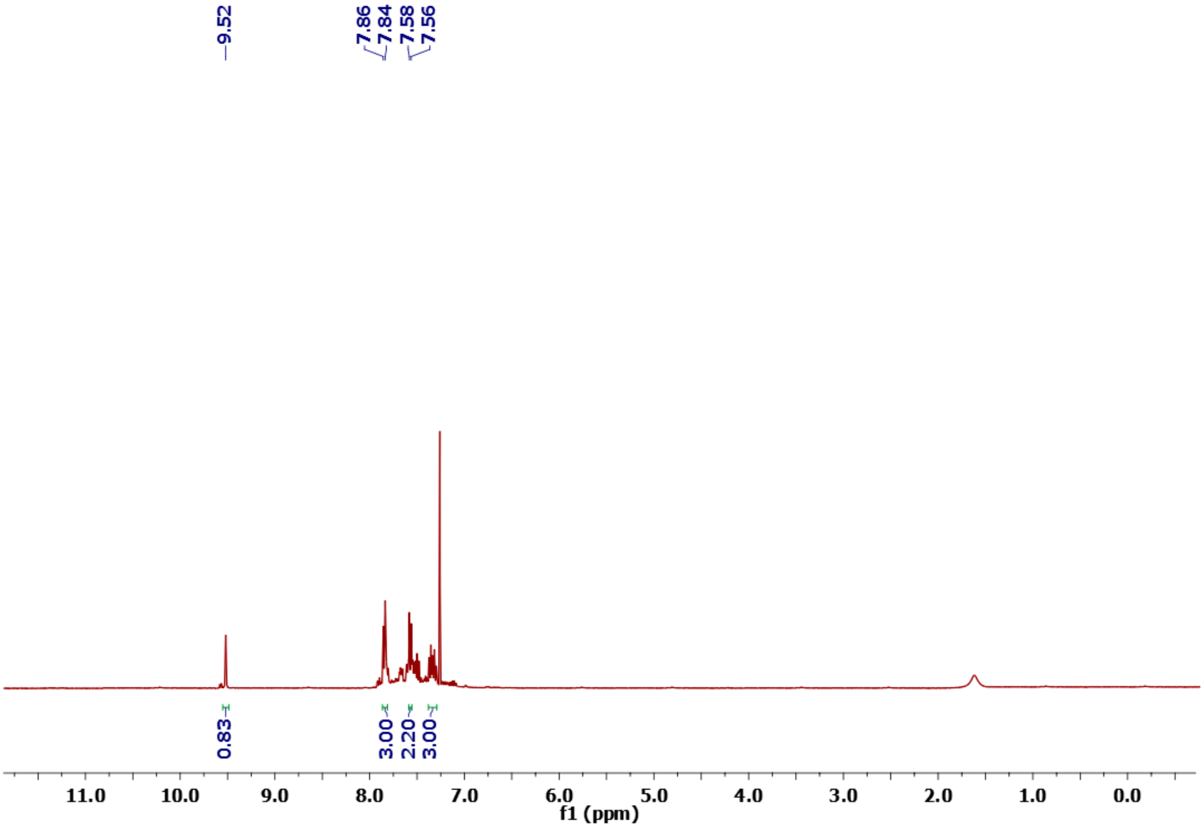

^{13}C-CRAPT NMR ($CDCl_3$) spectrum of 1-(2-fluorophenyl)-5-imino-3-(4-(trifluoromethyl)phenyl)imidazolidine-2,4-dithione

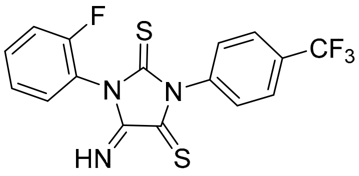

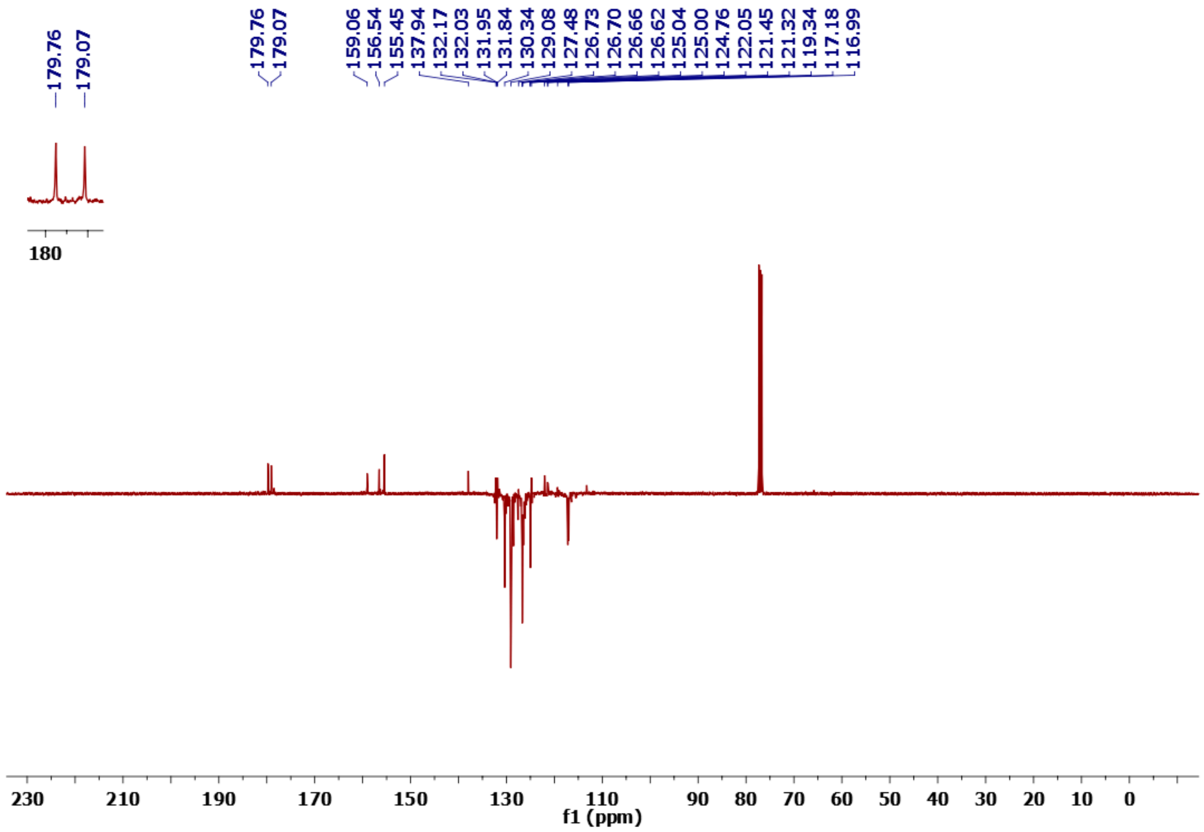

^{1}H NMR ($CDCl_3$) spectrum of 1-(4-chlorophenyl)-5-imino-3-(4-(trifluoromethyl)phenyl)imidazolidine-2,4-dithione (18v')

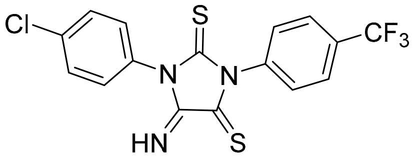

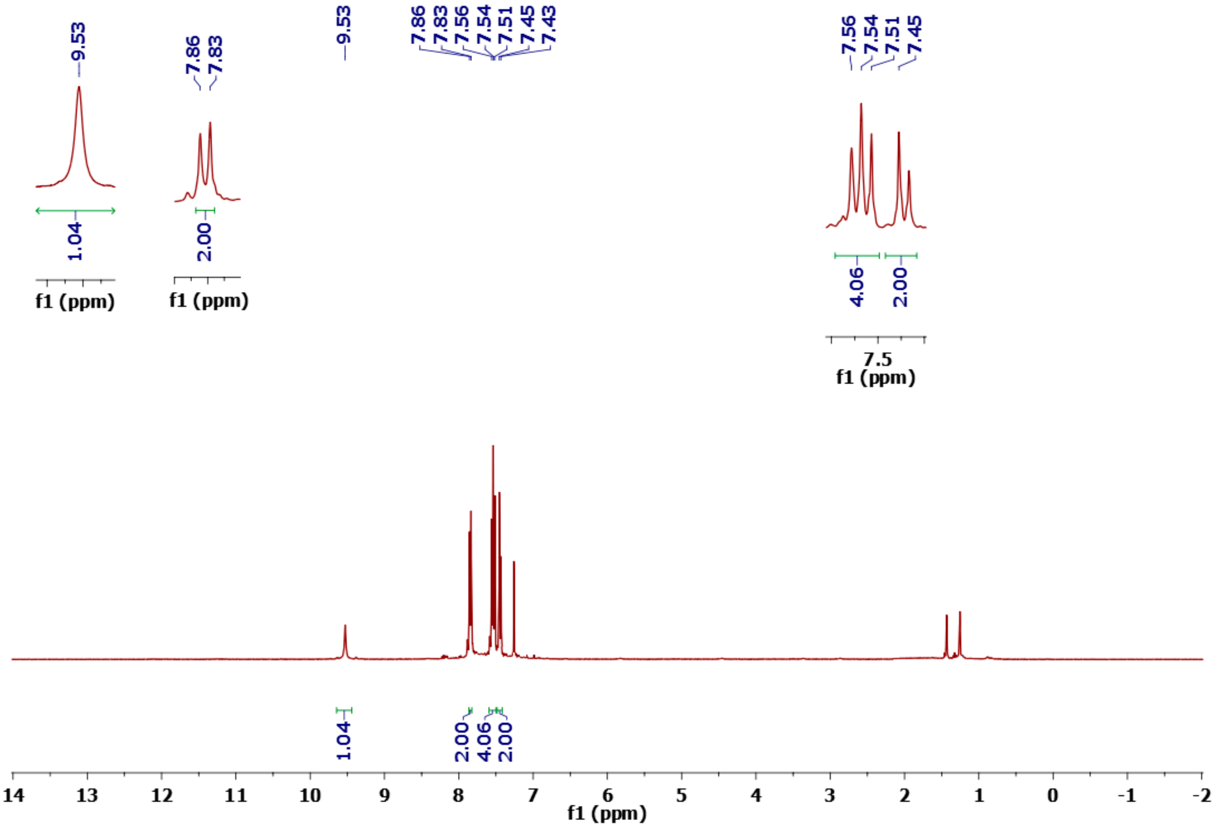

^{13}C NMR ($CDCl_3$) spectrum of 1-(4-chlorophenyl)-5-imino-3-(4-(trifluoromethyl)phenyl)imidazolidine-2,4-dithione

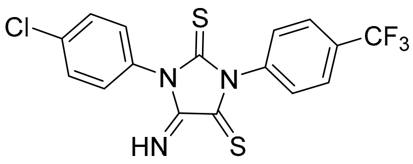

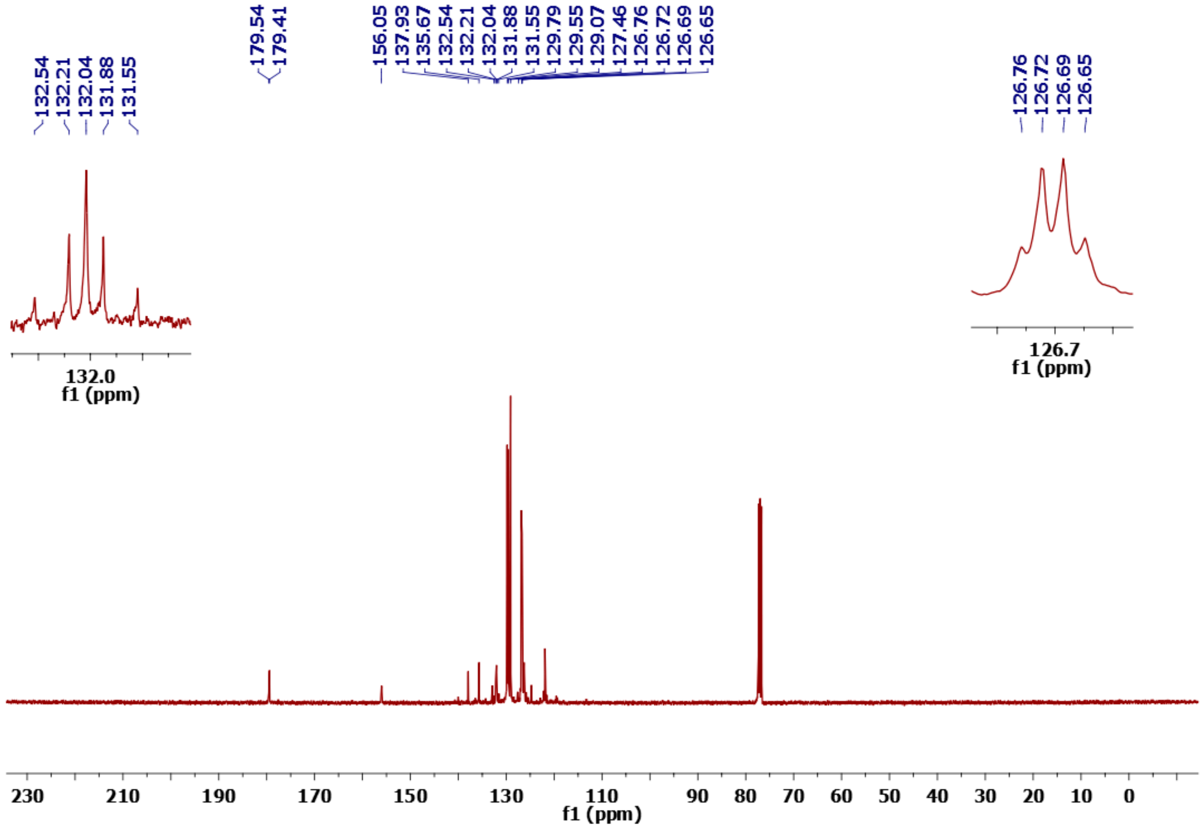

^{13}C-CRAPT NMR ($CDCl_3$) spectrum of 1-(4-chlorophenyl)-5-imino-3-(4-(trifluoromethyl)phenyl)imidazolidine-2,4-dithione

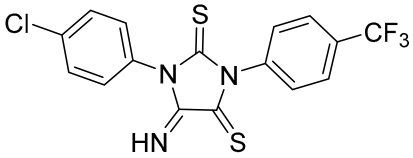

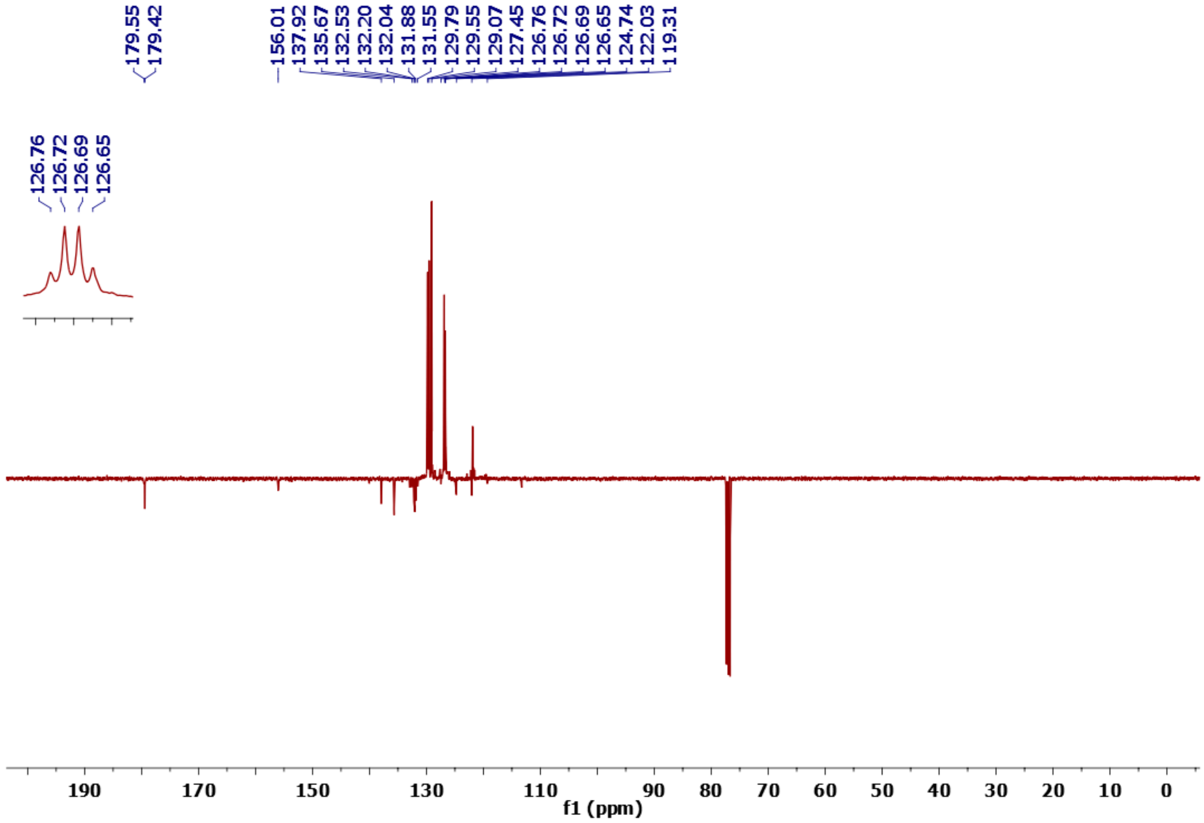

^{1}H-^{1}H-gCOSYAD NMR ($CDCl_3$) spectrum of 1-(4-chlorophenyl)-5-imino-3-(4-(trifluoromethyl)phenyl)imidazolidine-2,4-dithione

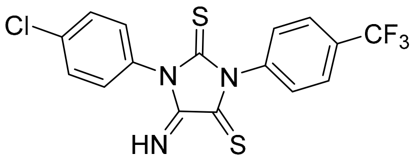

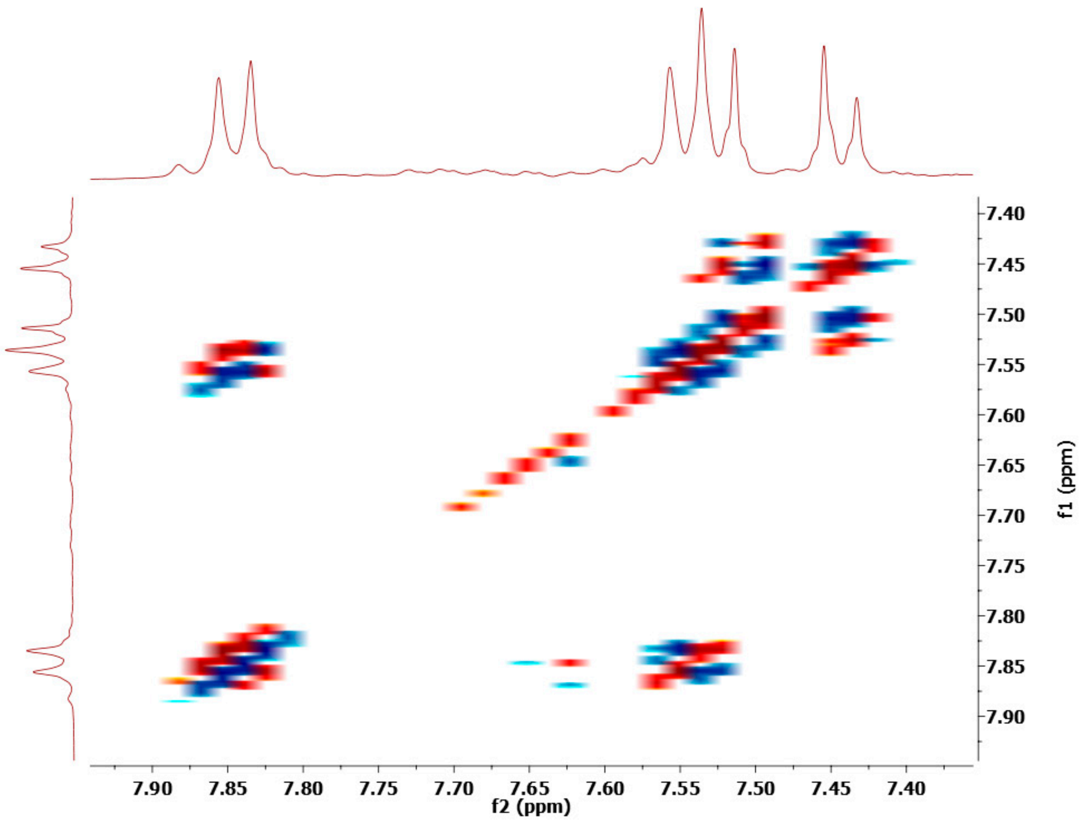

^{1}H-^{13}C-gHSQCAD NMR ($CDCl_3$) spectrum of 1-(4-chlorophenyl)-5-imino-3-(4-(trifluoromethyl)phenyl)imidazolidine-2,4-dithione

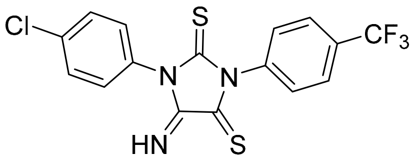

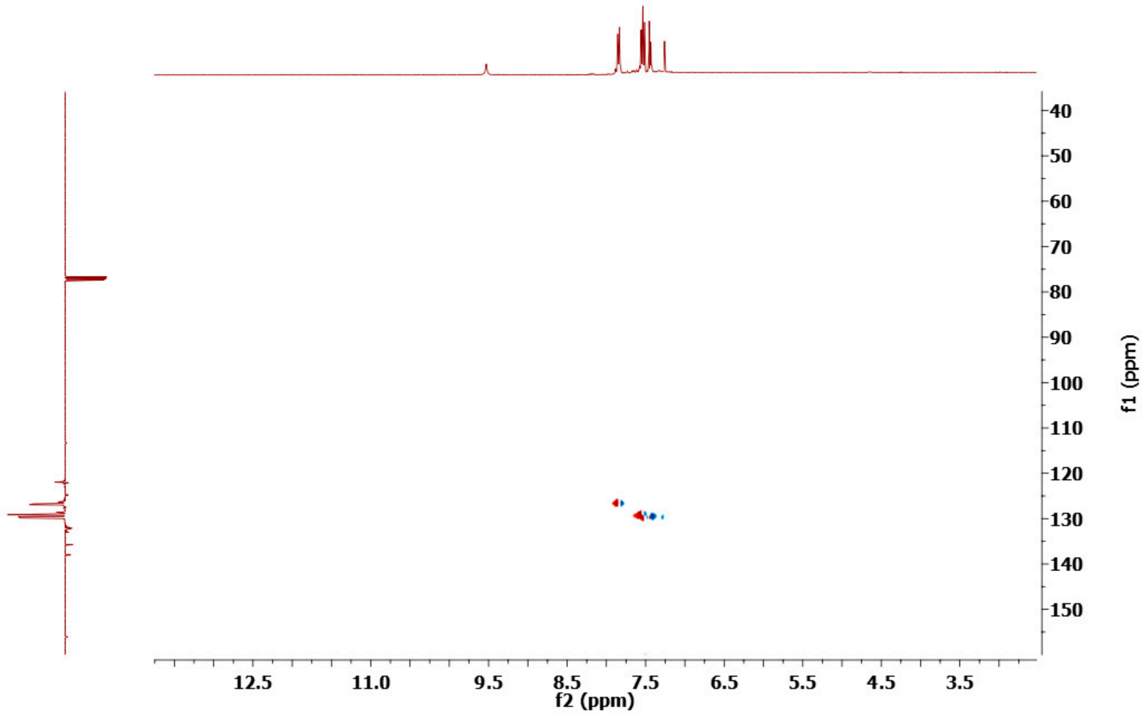

^{1}H-NMR (CDCl$_3$) spectrum of 5-imino-1-(4-nitrophenyl)-3-(4-(trifluoromethyl)phenyl)imidazolidine-2,4-dithione (18w')

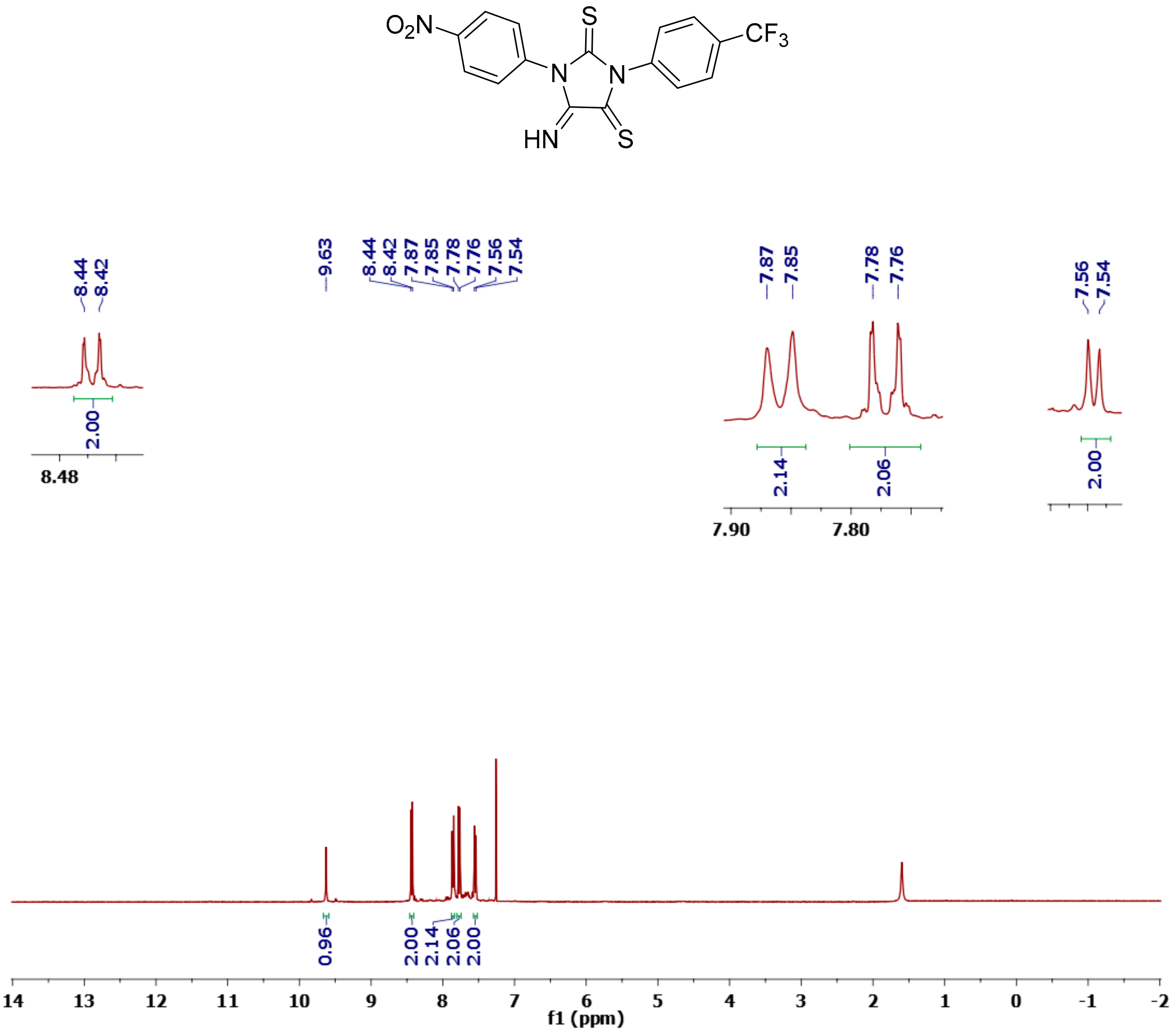

^{13}C NMR ($CDCl_3$) spectrum of 5-imino-1-(4-nitrophenyl)-3-(4-(trifluoromethyl)phenyl)imidazolidine-2,4-dithione

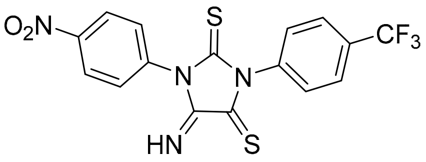

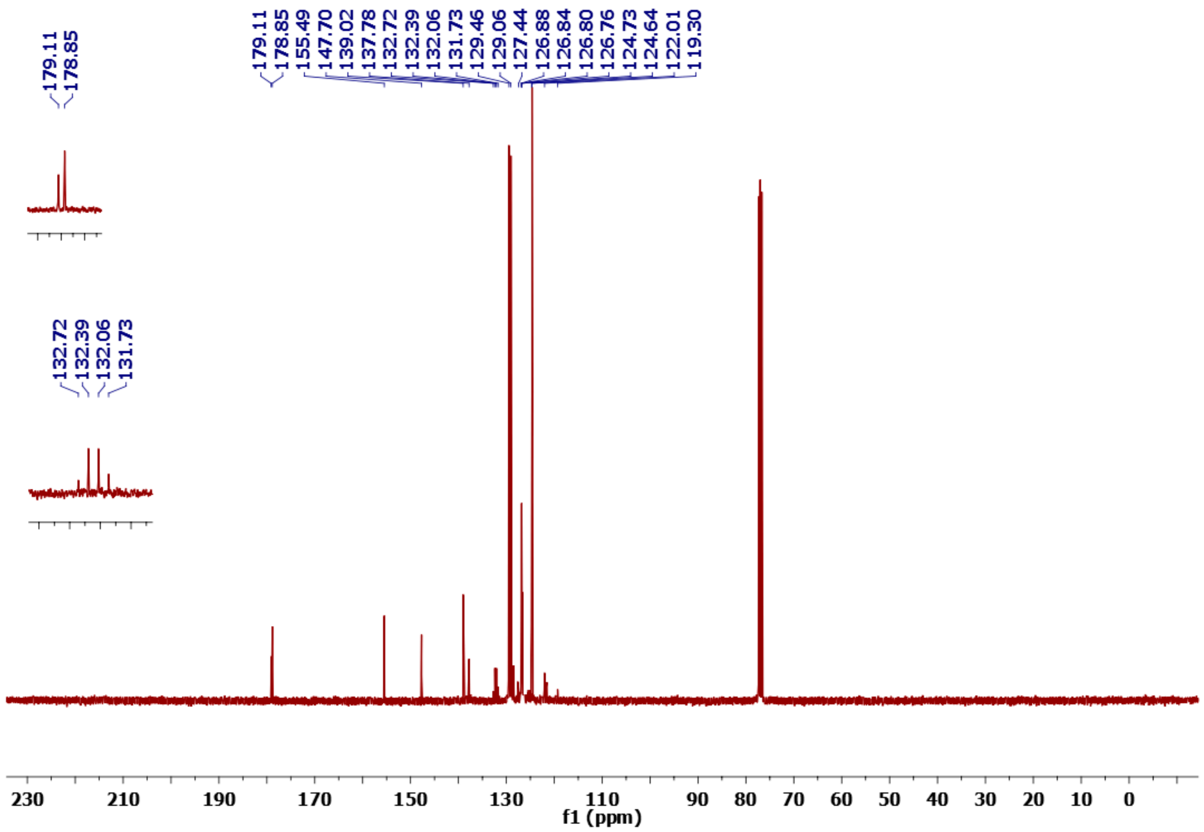

^{13}C-CRAPT NMR ($CDCl_3$) spectrum of 5-imino-1-(4-nitrophenyl)-3-(4-(trifluoromethyl)phenyl)imidazolidine-2,4-dithione

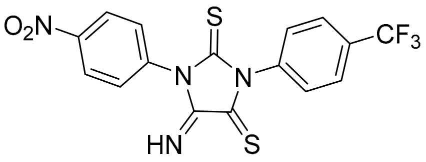

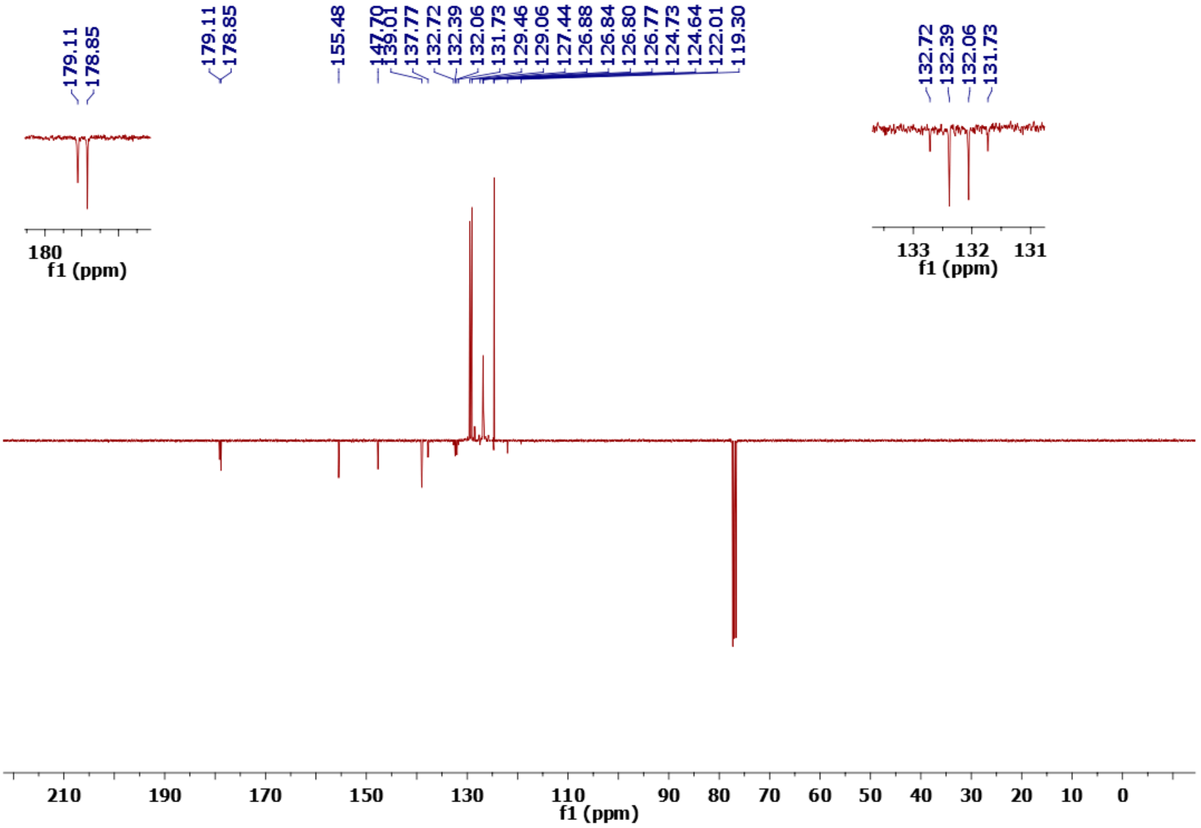

^{1}H-^{1}H-gCOSYAD NMR ($CDCl_3$) spectrum of 5-imino-1-(4-nitrophenyl)-3-(4-(trifluoromethyl)phenyl)imidazolidine-2,4-dithione

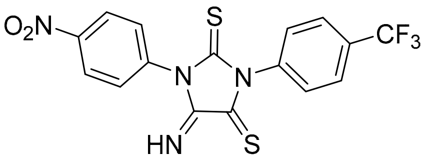

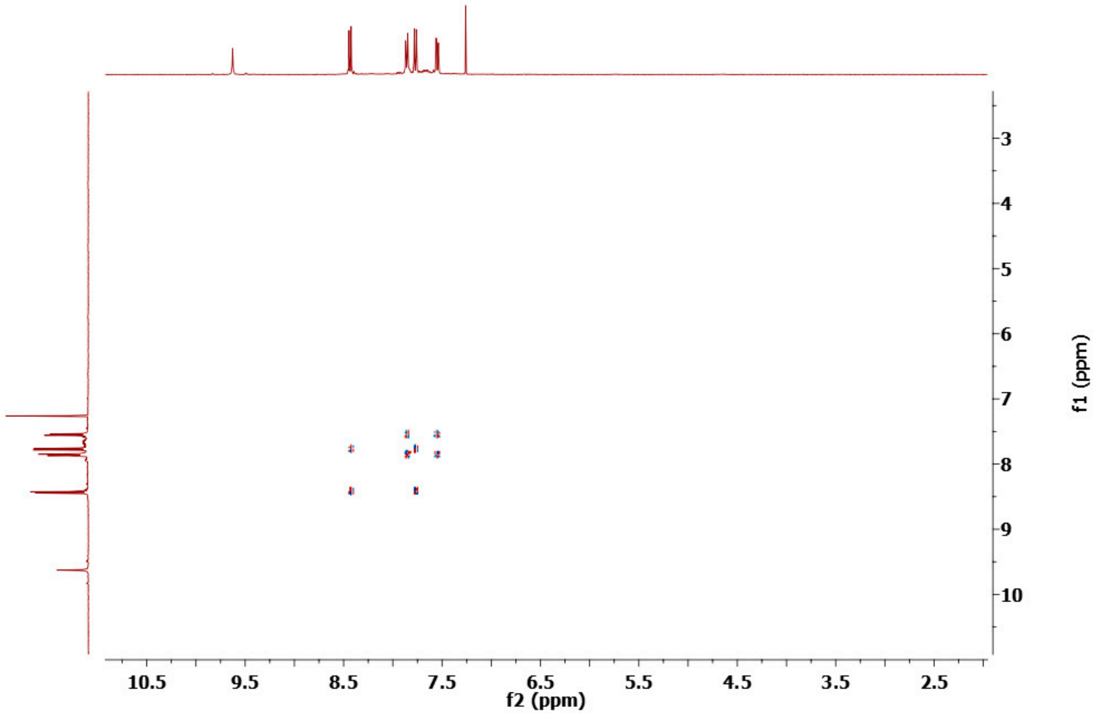

^{1}H-^{13}C-gHSQCAD NMR ($CDCl_3$) spectrum of 5-imino-1-(4-nitrophenyl)-3-(4-(trifluoromethyl)phenyl)imidazolidine-2,4-dithione

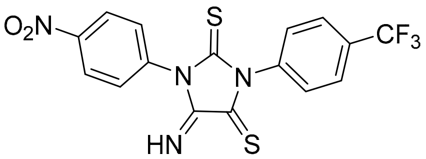

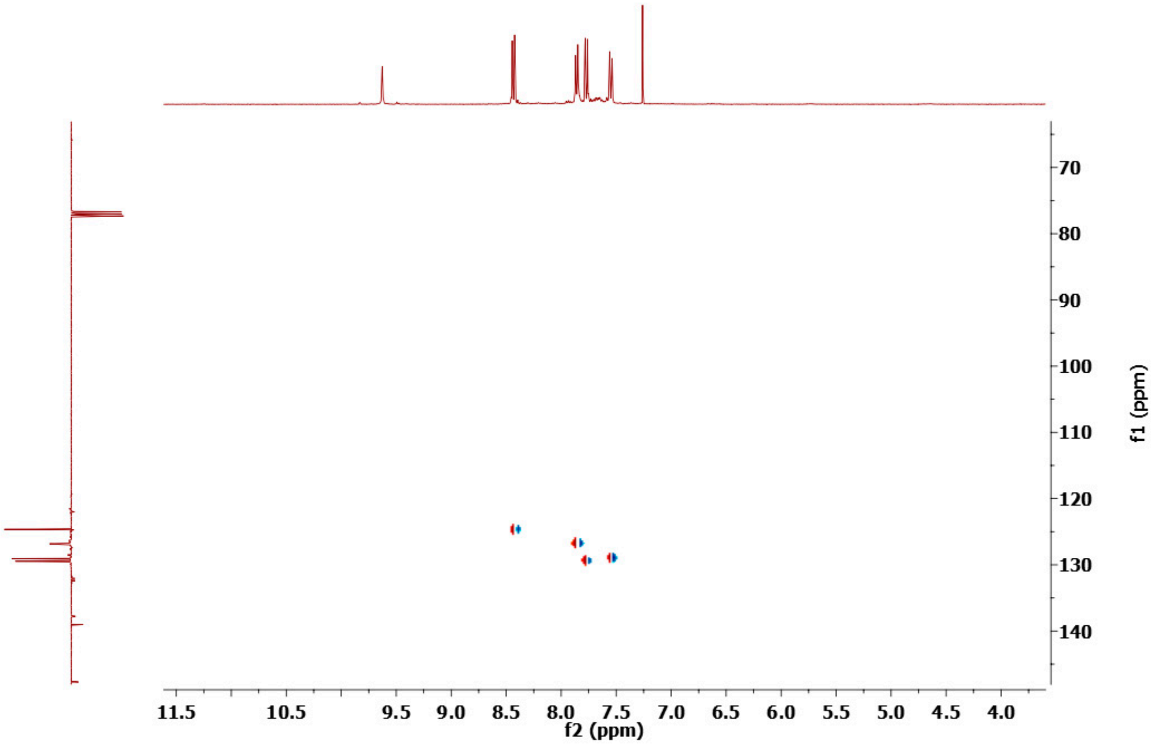

^{1}H-^{13}C-HMBC NMR ($CDCl_3$) spectrum of 5-imino-1-(4-nitrophenyl)-3-(4-(trifluoromethyl)phenyl)imidazolidine-2,4-dithione

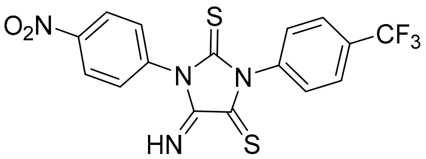

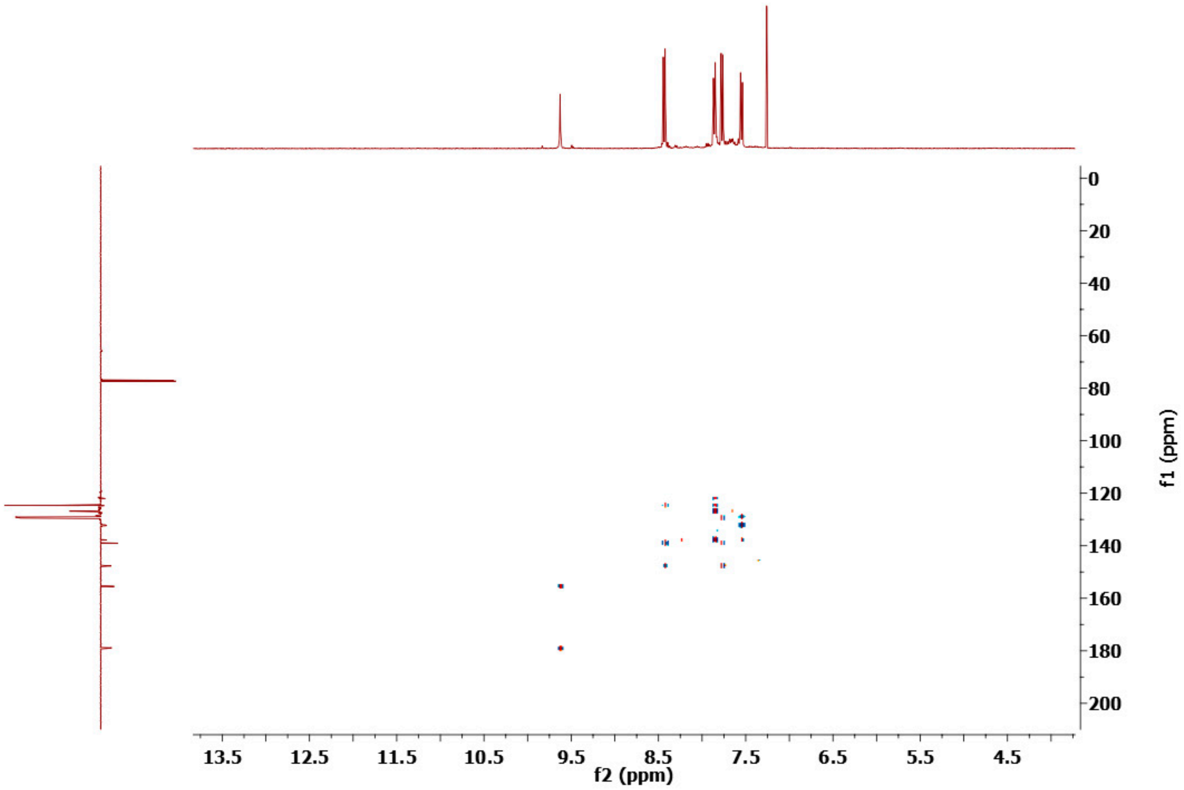

^{1}H-NMR (DMSO-d$_6$) spectrum of 5-imino-1,3-bis(4-nitrophenyl)imidazolidine-2,4-dithione (18x')

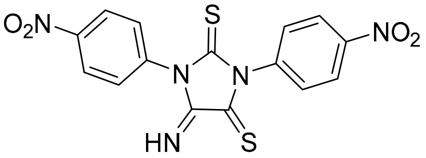

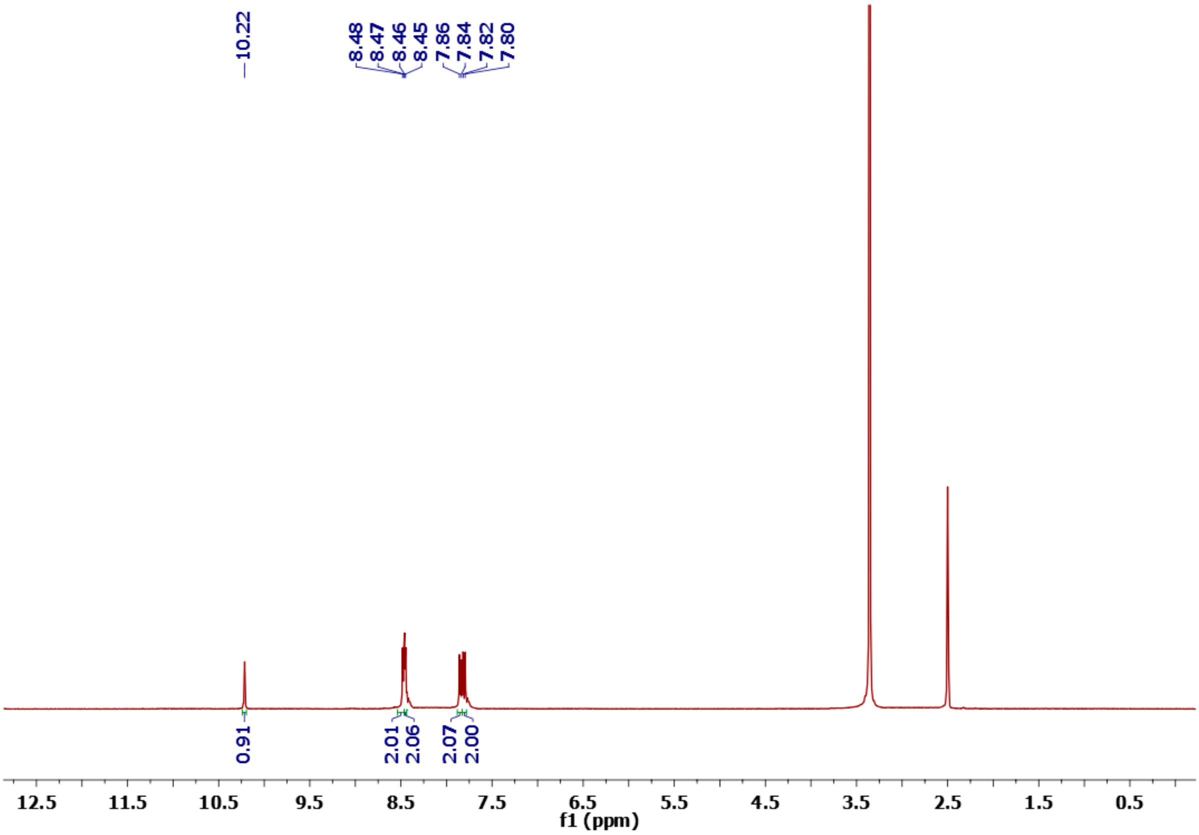

^{13}C-CRAPT NMR (DMSO-d$_6$) spectrum of 5-imino-1,3-bis(4-nitrophenyl)imidazolidine-2,4-dithione

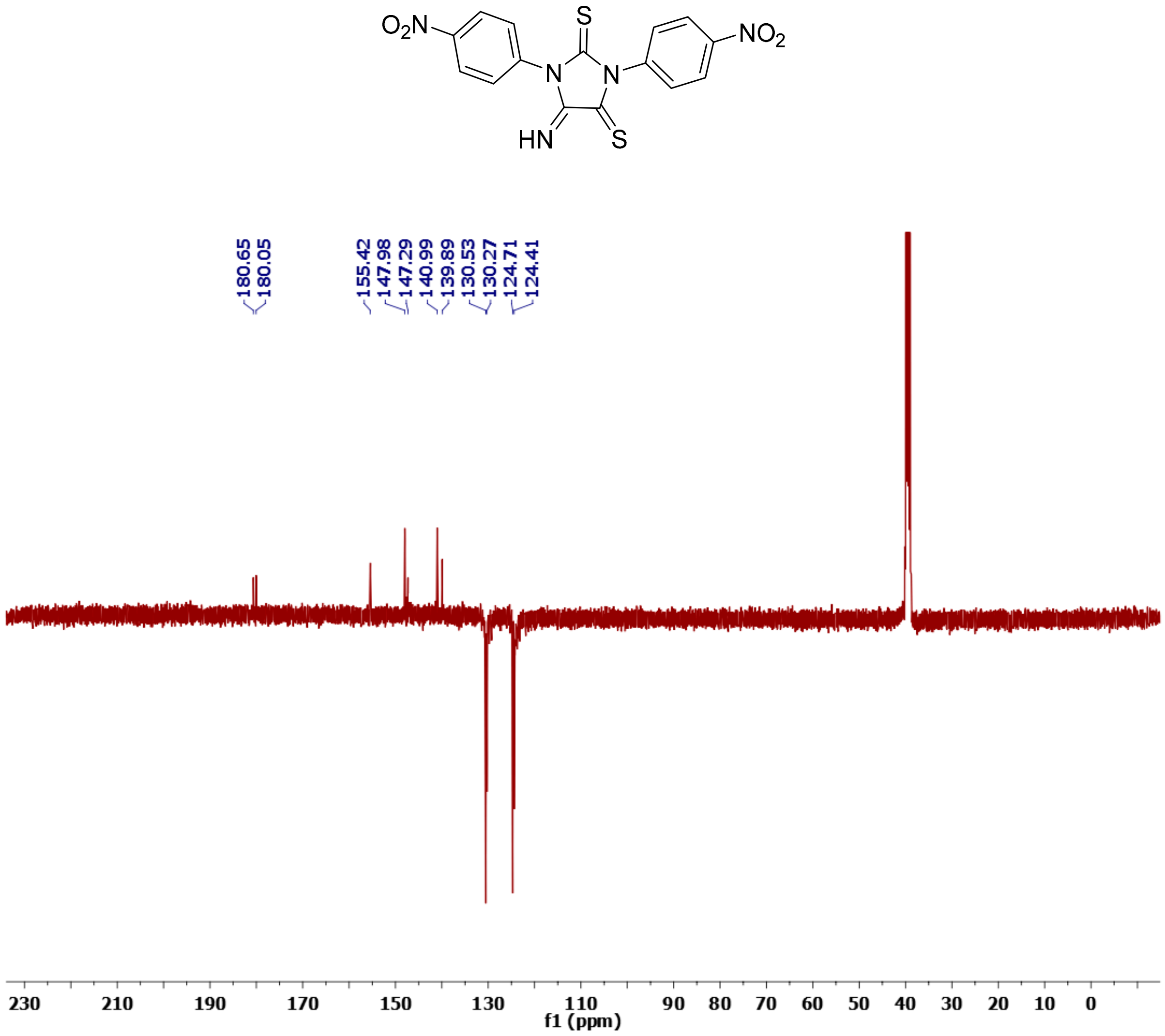

^{1}H NMR ($CDCl_3$) spectrum of (3-(4-fluorophenyl)-5-imino-2,4-dithioxoimidazolidin-1-yl)(phenyl)methanone

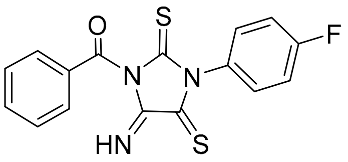

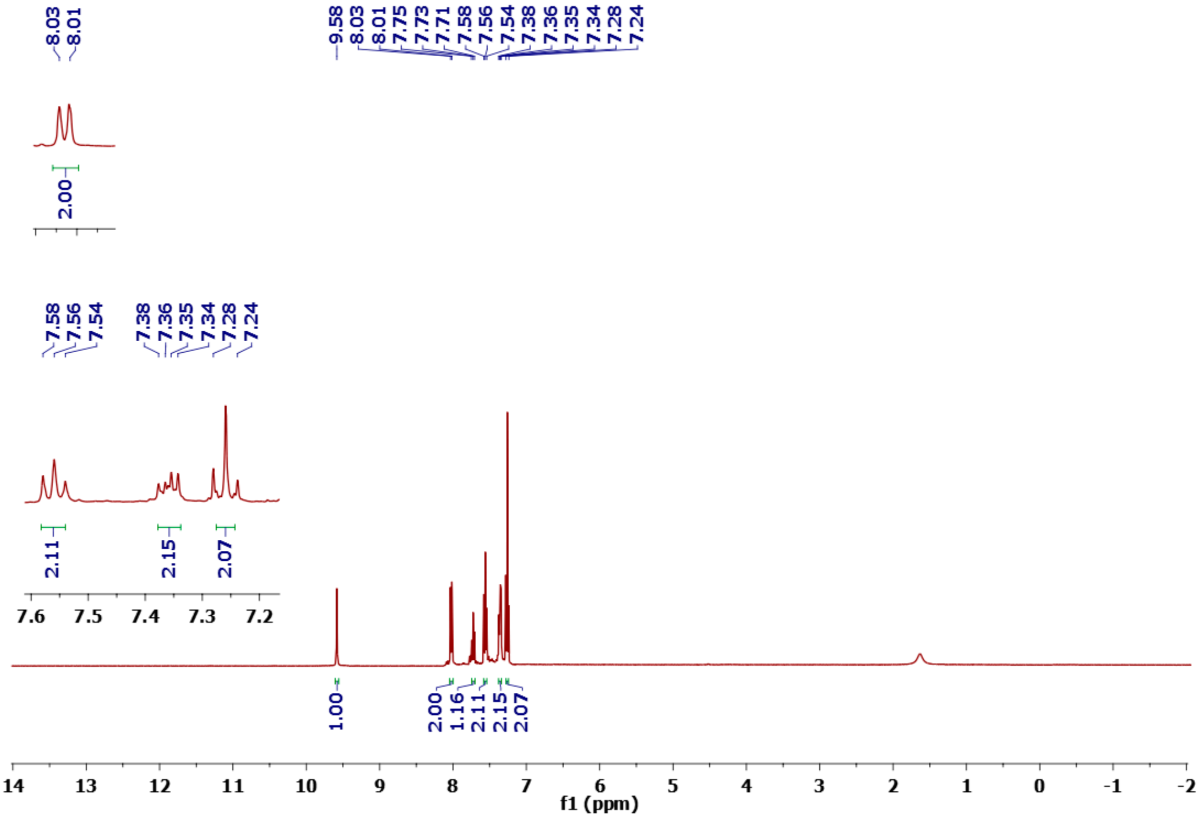

^{13}C NMR ($CDCl_3$) spectrum of (3-(4-fluorophenyl)-5-imino-2,4-dithioxoimidazolidin-1-yl)(phenyl)methanone

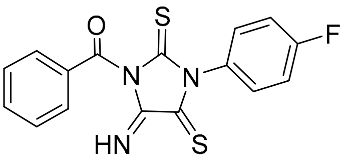

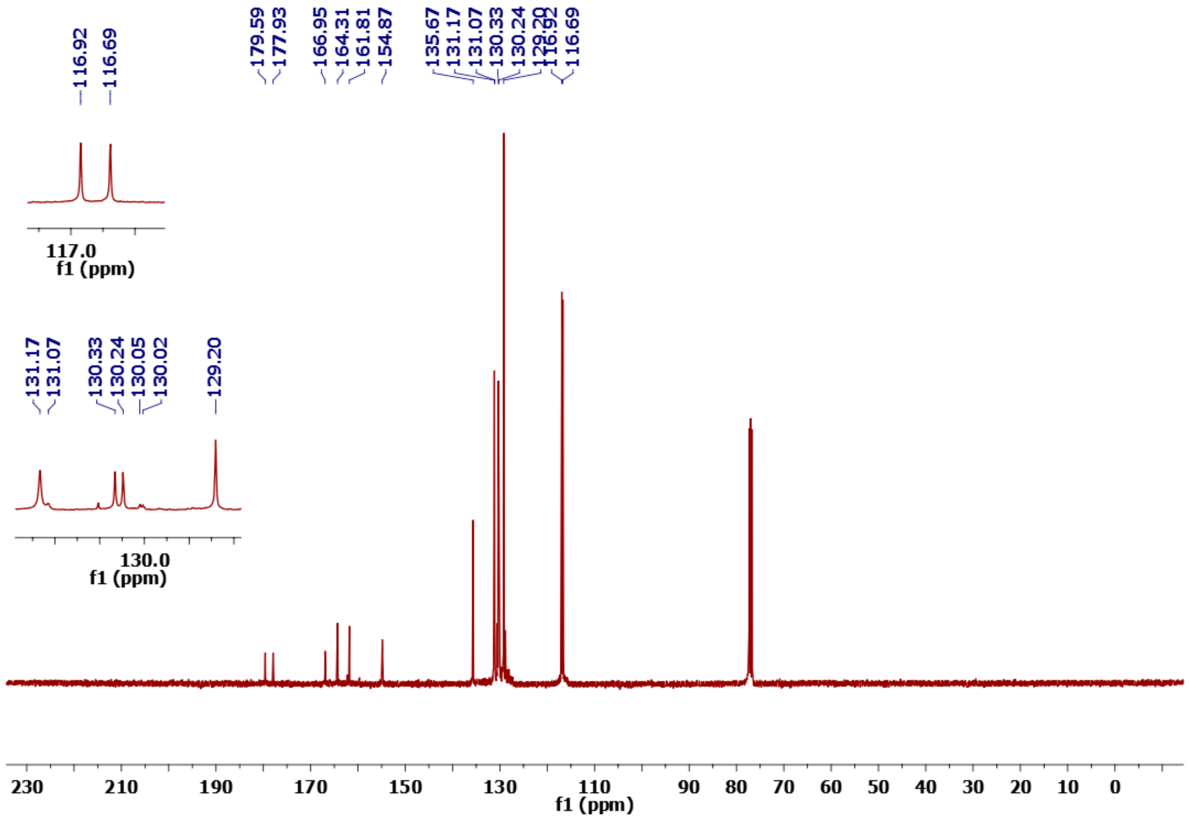

^{13}C-CRAPT NMR ($CDCl_3$) spectrum of (3-(4-fluorophenyl)-5-imino-2,4-dithioxoimidazolidin-1-yl)(phenyl)methanone

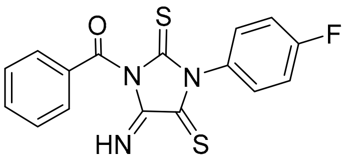

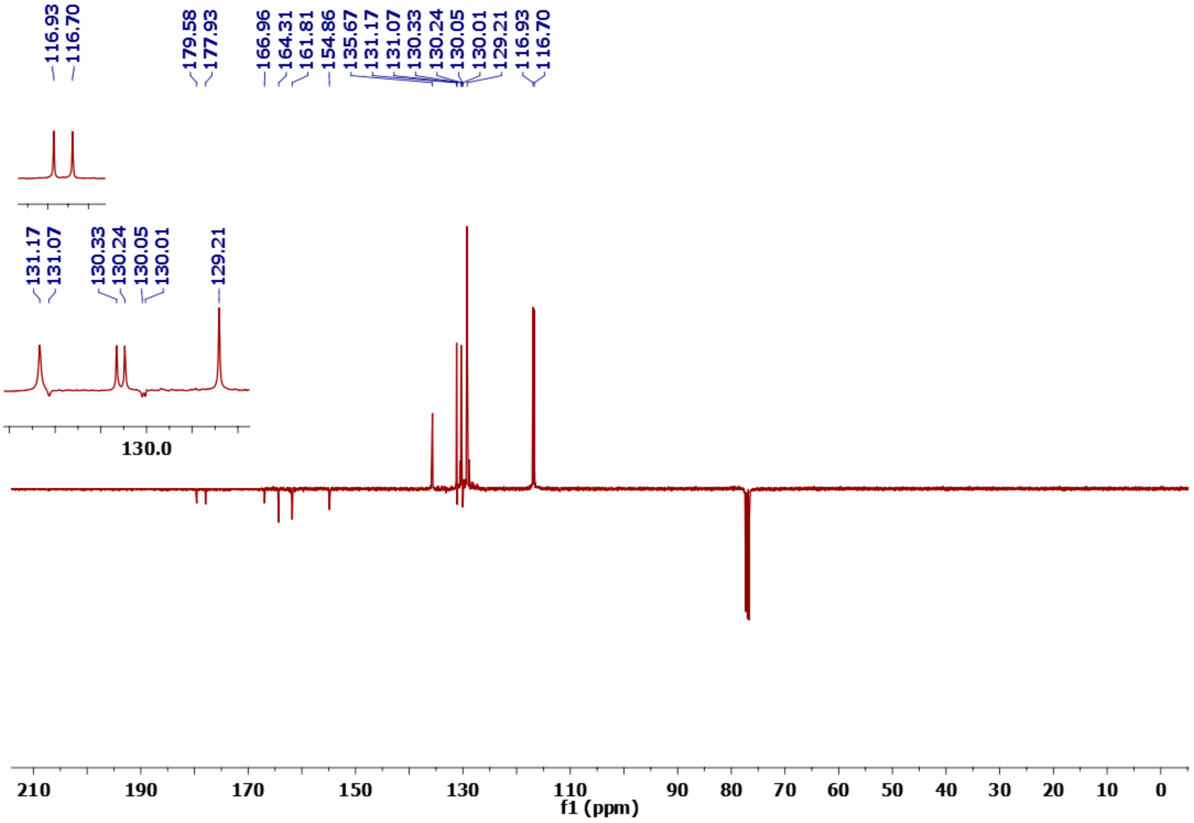

^{1}H-^{1}H-gCOSYAD NMR ($CDCl_3$) spectrum of (3-(4-fluorophenyl)-5-imino-2,4-dithioxoimidazolidin-1-yl)(phenyl)methanone

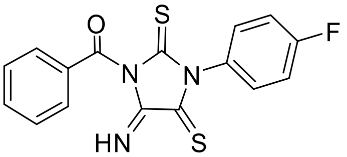

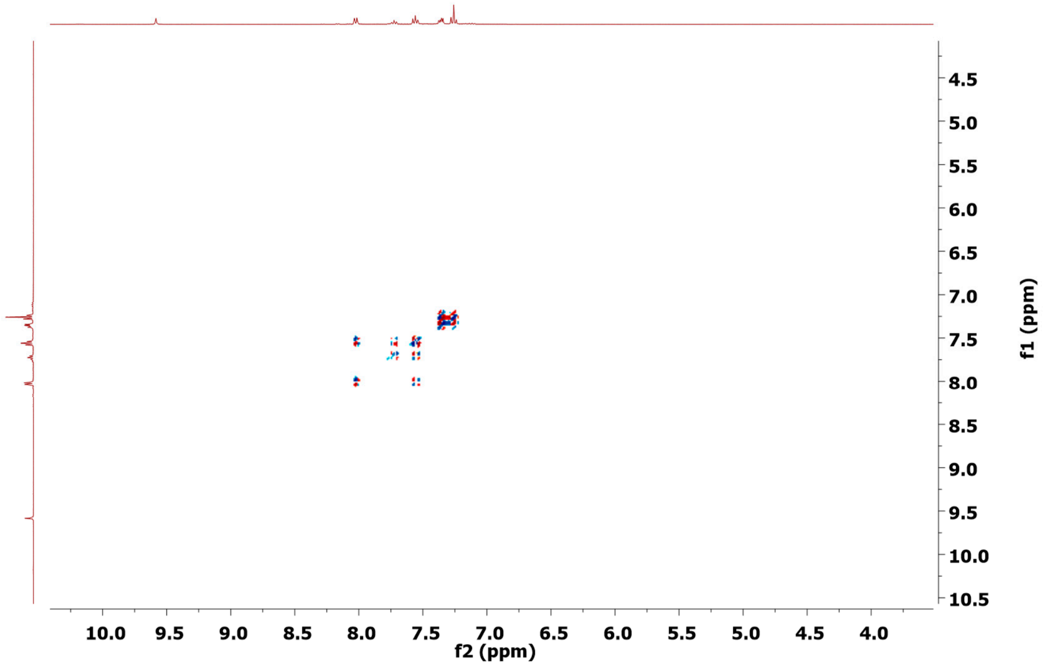

^{1}H NMR ($CDCl_3$) spectrum of 3-(4-fluorophenyl)-5-imino-1-phenylimidazolidine-2,4-dithione (18z')

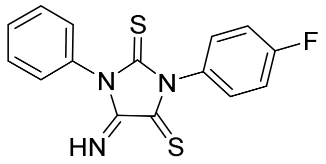

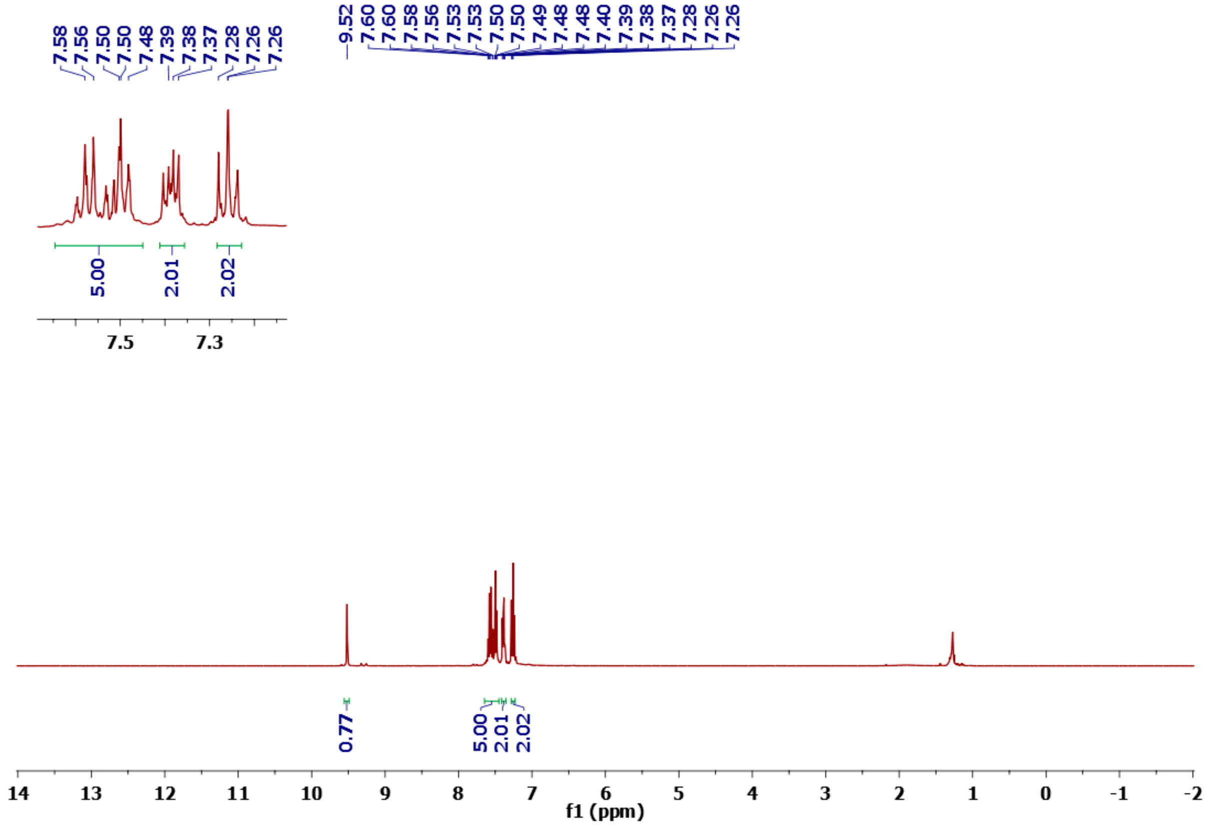

^{13}C NMR ($CDCl_3$) spectrum of 3-(4-fluorophenyl)-5-imino-1-phenylimidazolidine-2,4-dithione

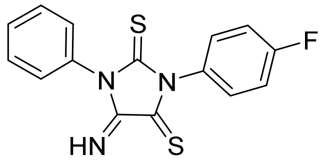

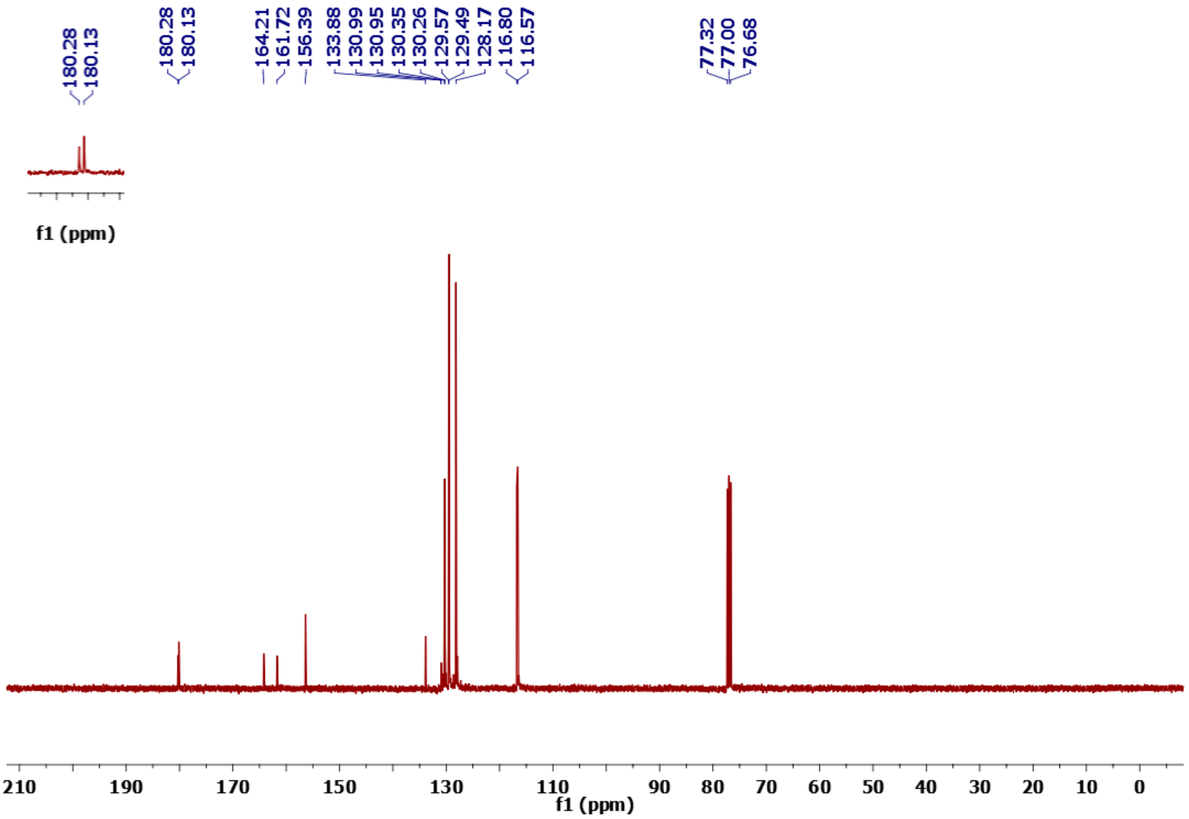

^{13}C-CRAPT NMR ($CDCl_3$) spectrum of 3-(4-fluorophenyl)-5-imino-1-phenylimidazolidine-2,4-dithione

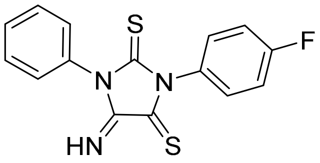

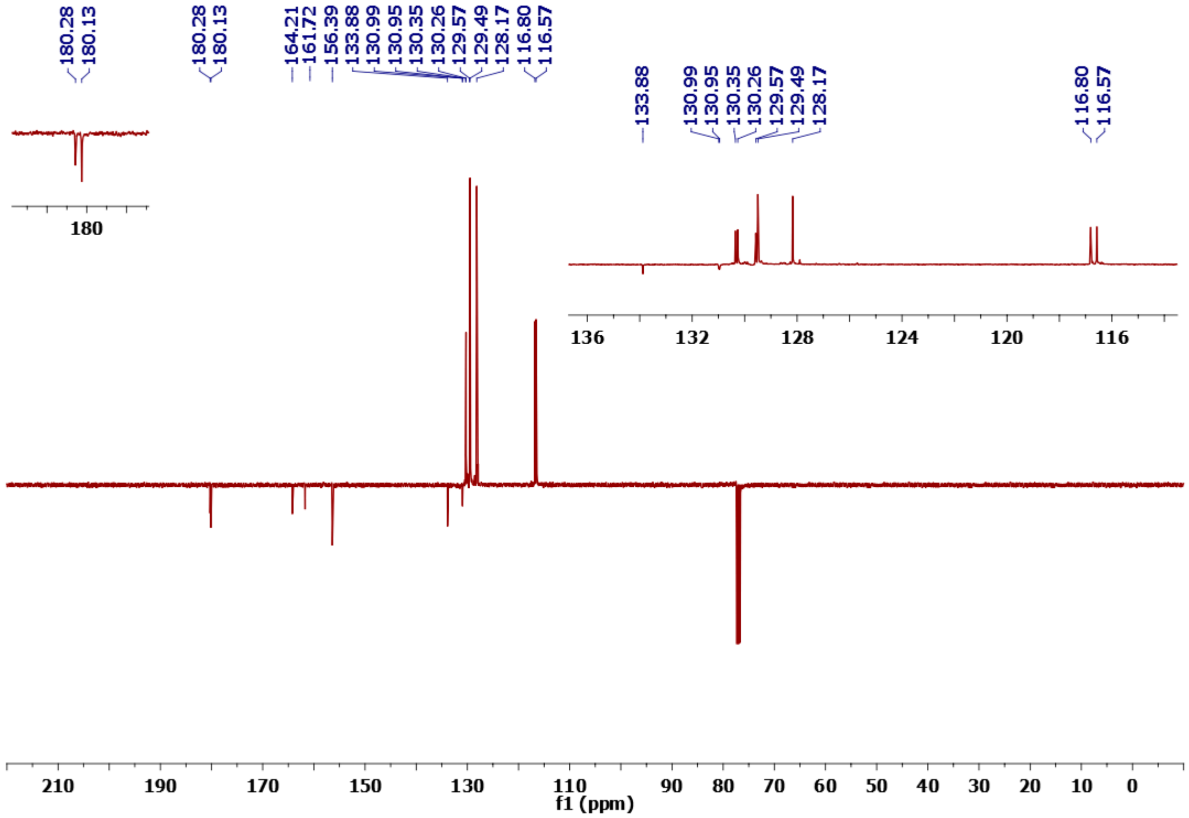

^{1}H-^{1}H-gCOSYAD NMR ($CDCl_3$) spectrum of 3-(4-fluorophenyl)-5-imino-1-phenylimidazolidine-2,4-dithione

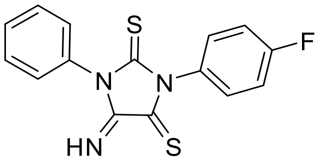

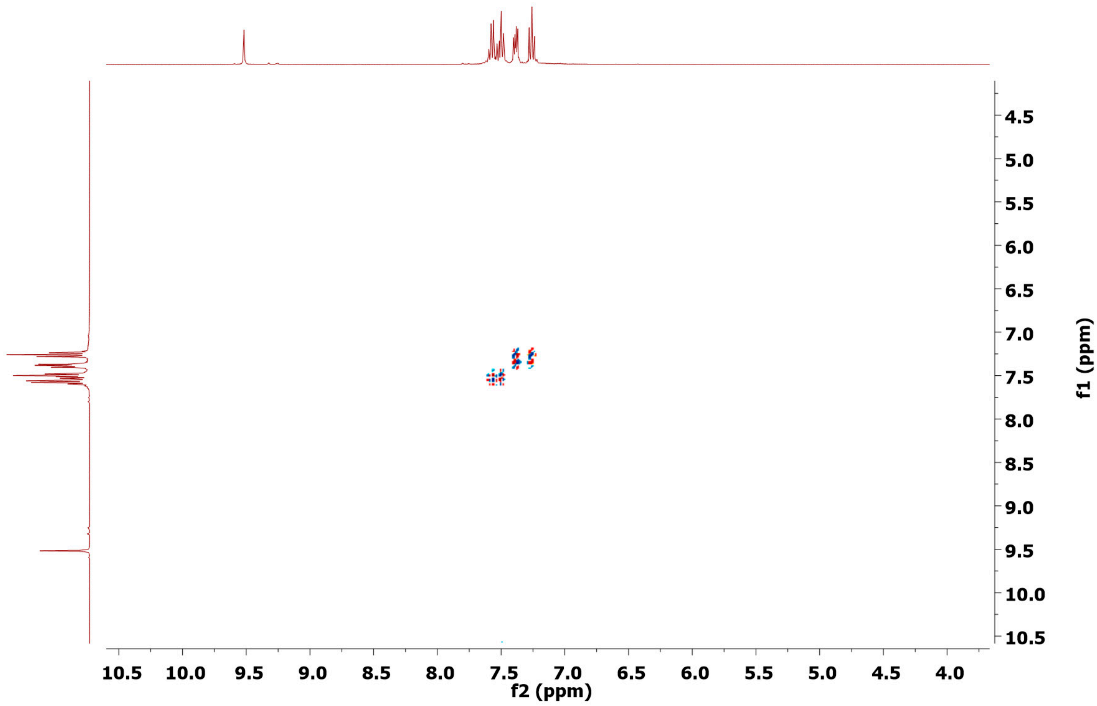

^{1}H-^{13}C-gHSQCAD NMR ($CDCl_3$) spectrum of 3-(4-fluorophenyl)-5-imino-1-phenylimidazolidine-2,4-dithione

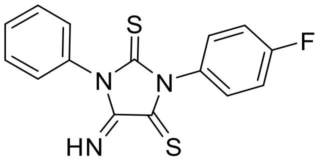

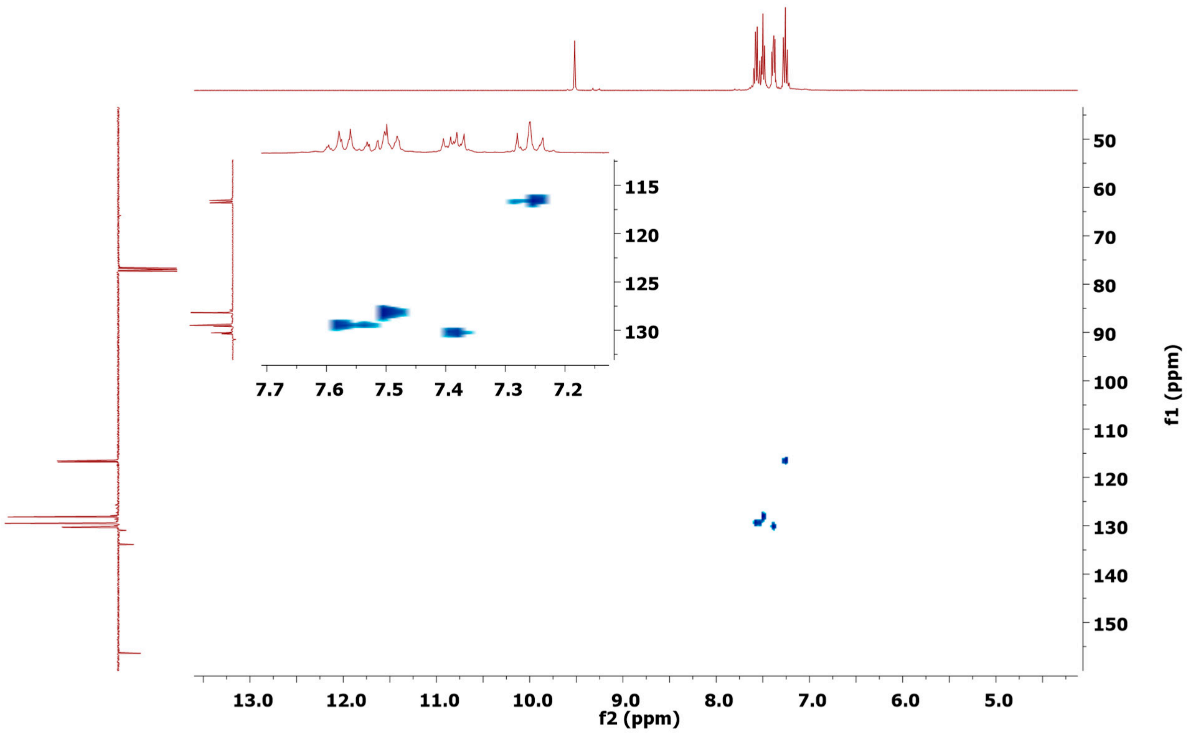

^{1}H-^{13}C-gHMBC NMR ($CDCl_3$) spectrum of 3-(4-fluorophenyl)-5-imino-1-phenylimidazolidine-2,4-dithione

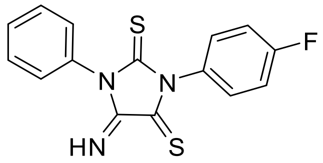

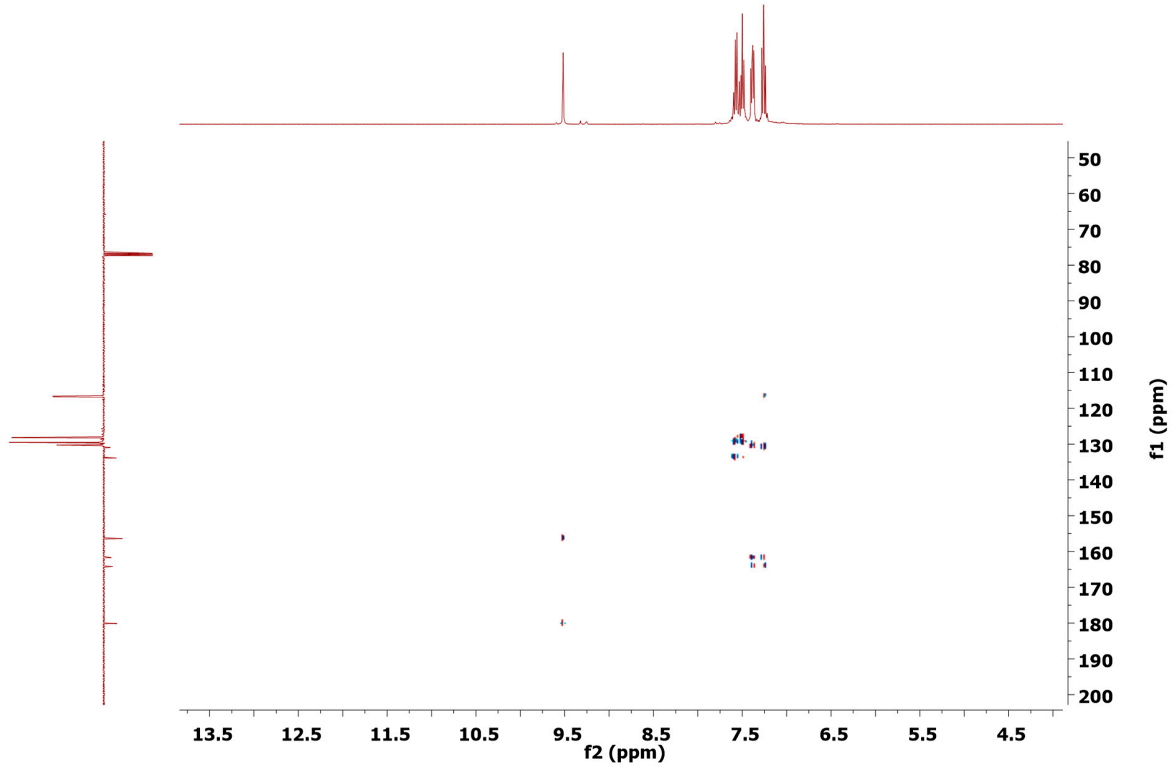

^{1}H NMR ($CDCl_3$) spectrum of 3-(4-fluorophenyl)-5-imino-1-(4-(trifluoromethyl)phenyl)imidazolidine-2,4-dithione (18a'')

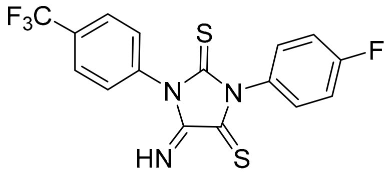

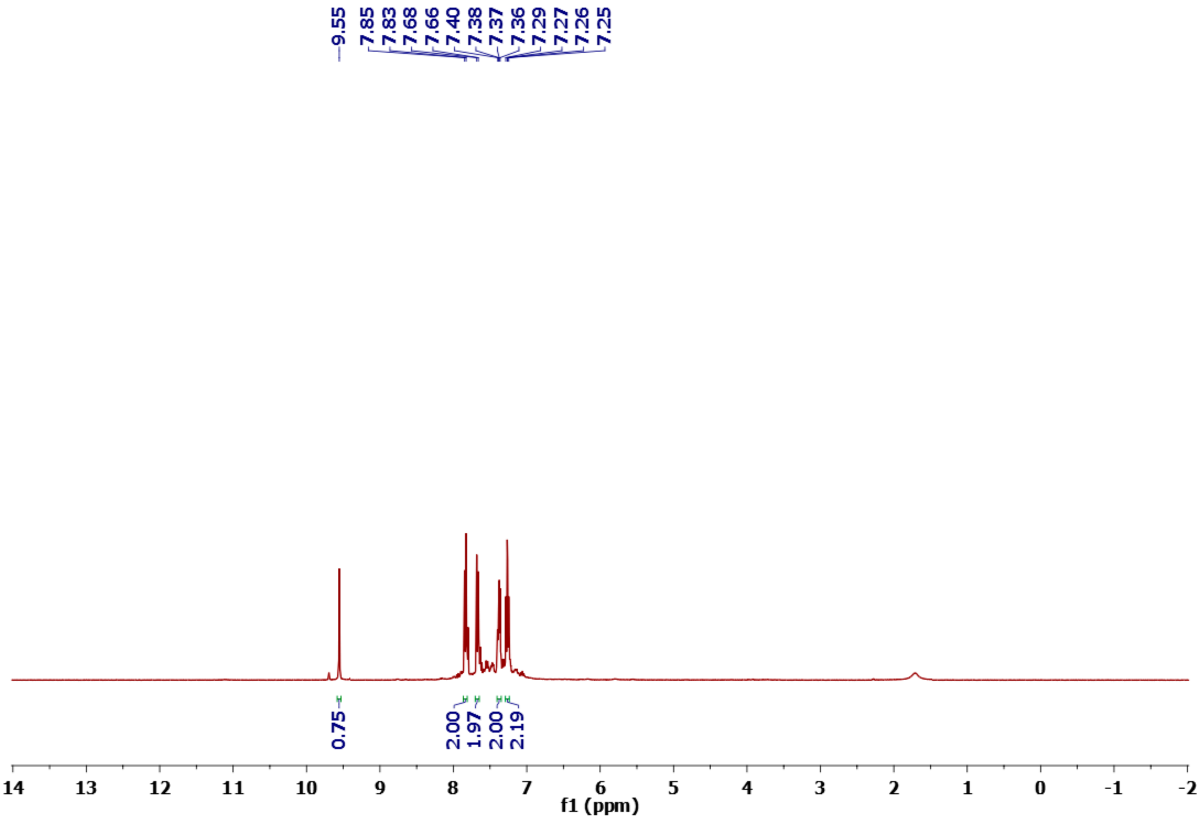

^{13}C NMR ($CDCl_3$) spectrum of 3-(4-fluorophenyl)-5-imino-1-(4-(trifluoromethyl)phenyl)imidazolidine-2,4-dithione

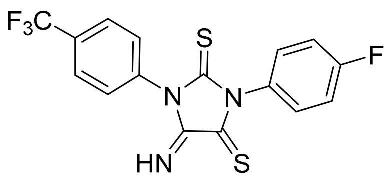

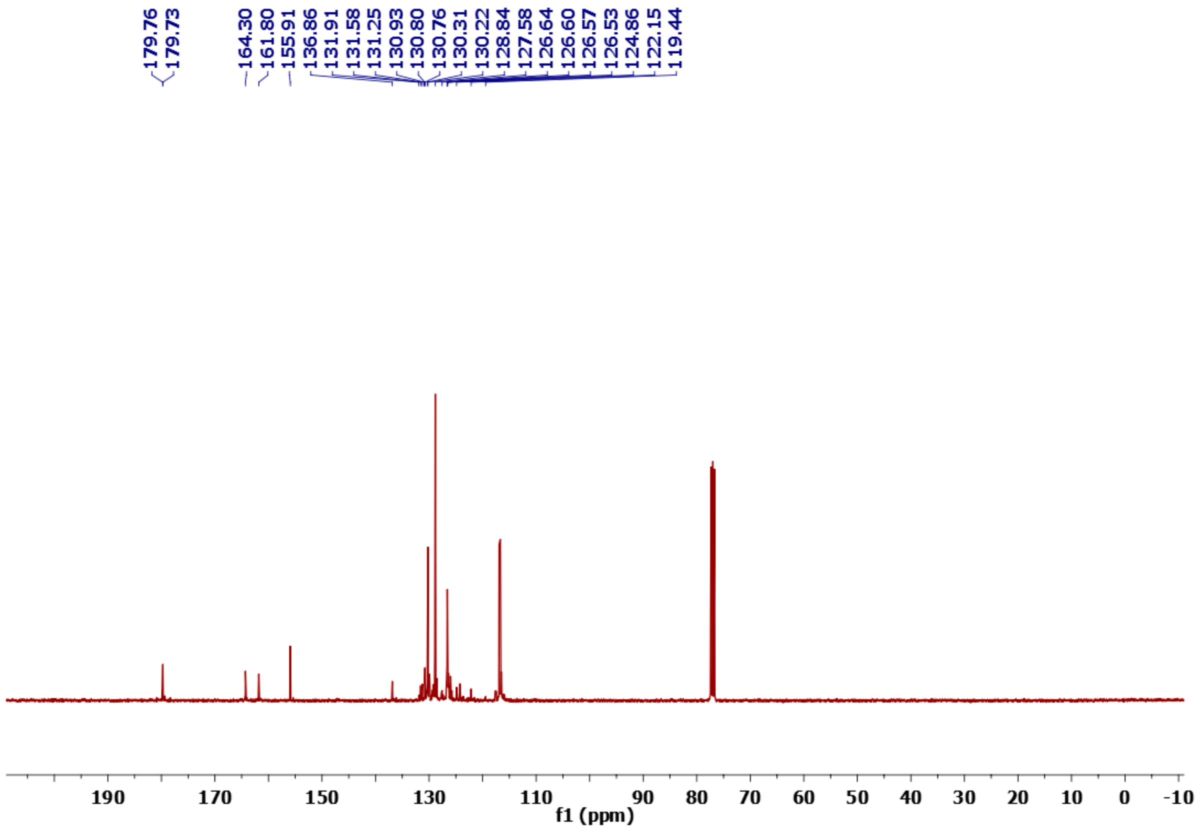

^{13}C-CRAPT NMR ($CDCl_3$) spectrum of 3-(4-fluorophenyl)-5-imino-1-(4-(trifluoromethyl)phenyl)imidazolidine-2,4-dithione

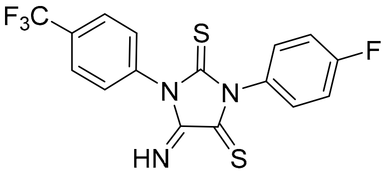

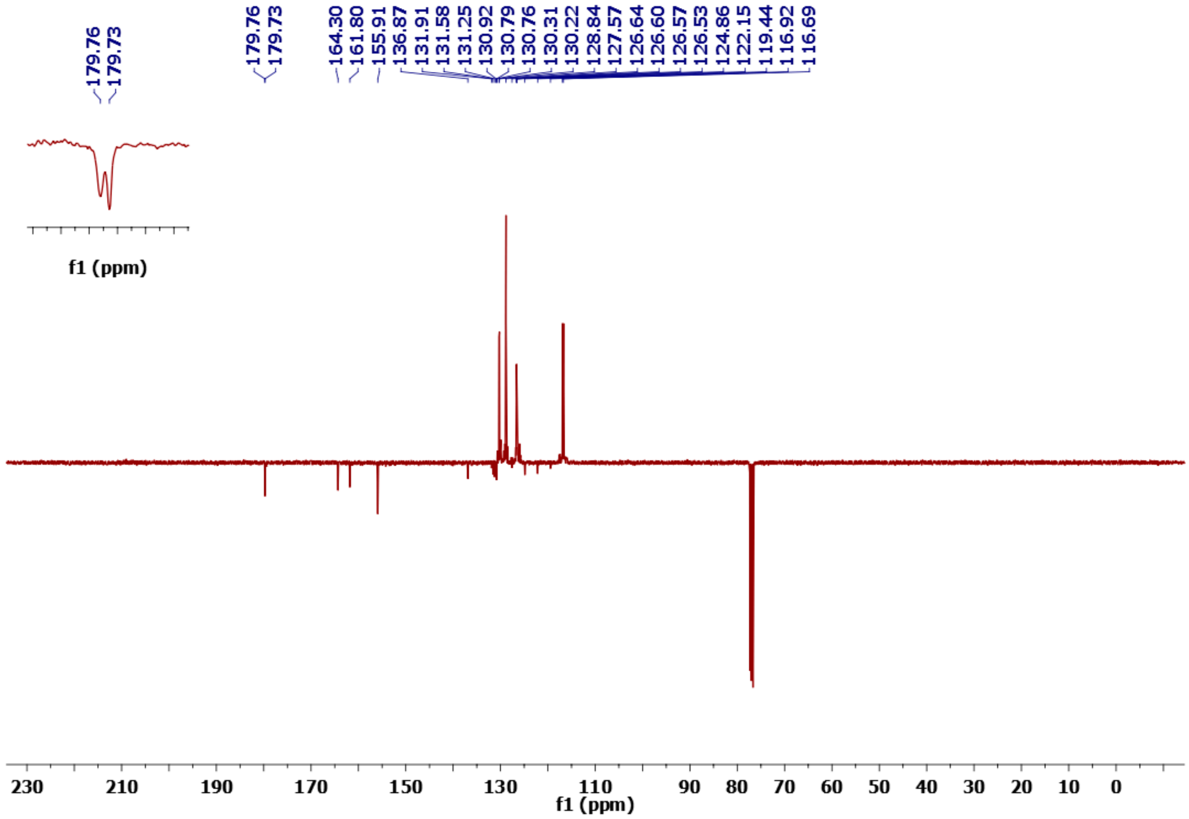

^{1}H-^{1}H-gCOSYAD NMR ($CDCl_3$) spectrum of 3-(4-fluorophenyl)-5-imino-1-(4-(trifluoromethyl)phenyl)imidazolidine-2,4-dithione

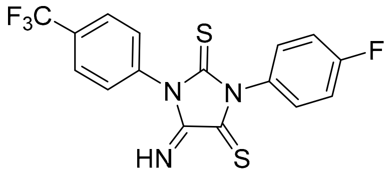

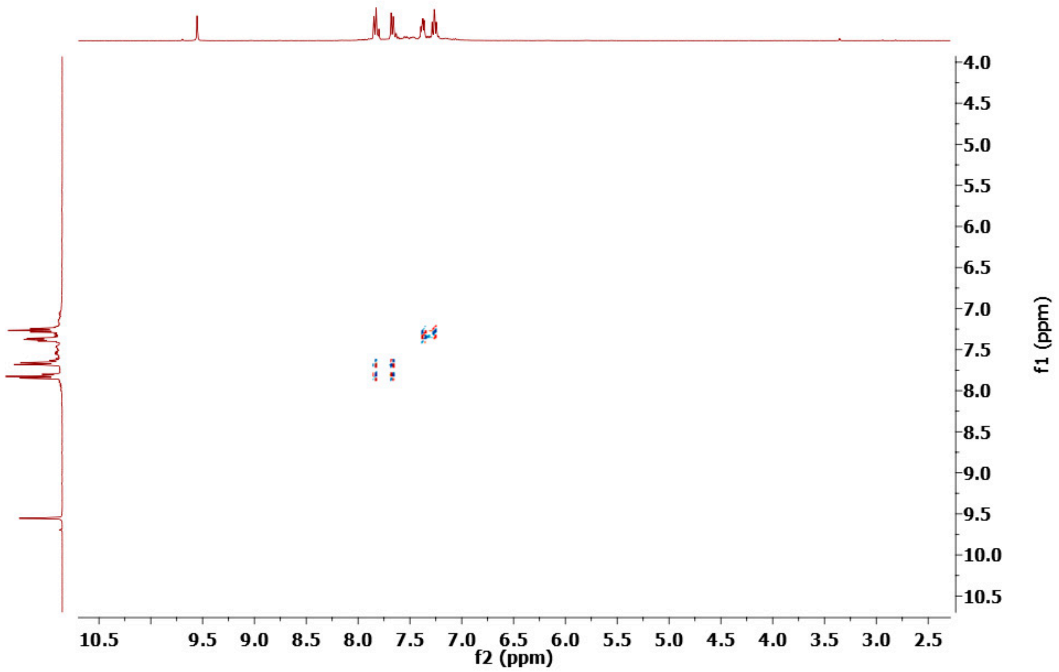

^{1}H-^{13}C-gHSQCAD NMR ($CDCl_3$) spectrum of 3-(4-fluorophenyl)-5-imino-1-(4-(trifluoromethyl)phenyl)imidazolidine-2,4-dithione

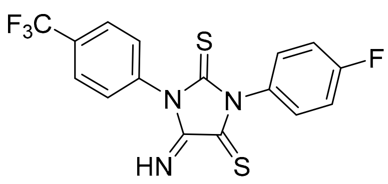

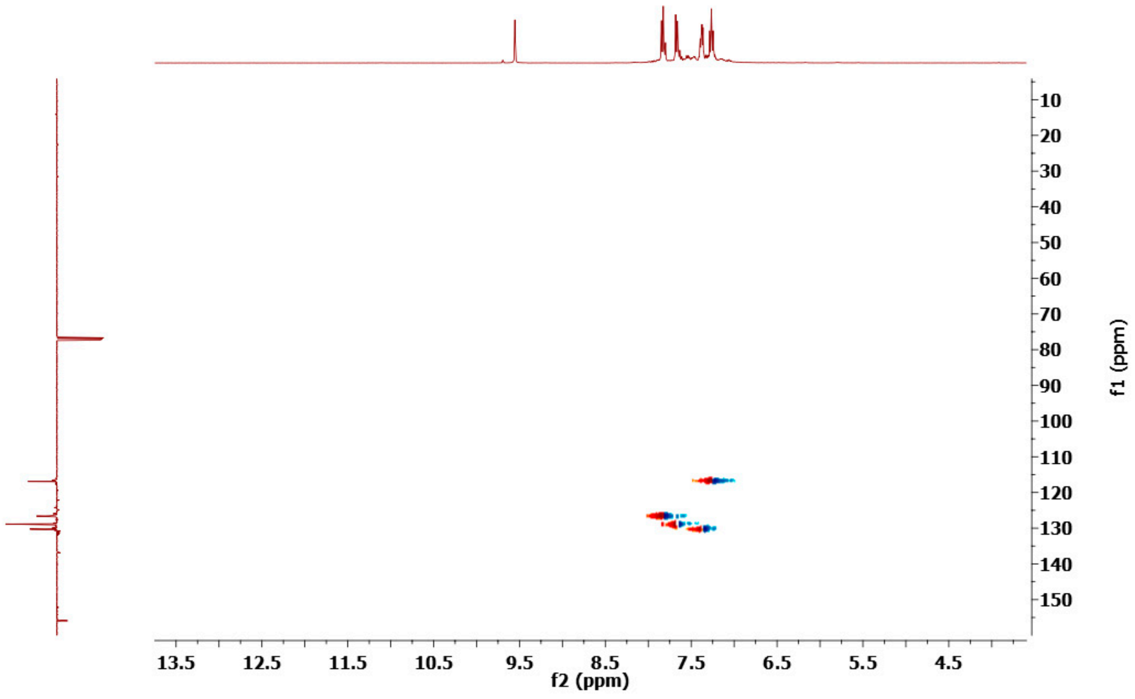

^{1}H-^{13}C-gHMBC NMR ($CDCl_3$) spectrum 3-(4-fluorophenyl)-5-imino-1-(4-(trifluoromethyl)phenyl)imidazolidine-2,4-dithione

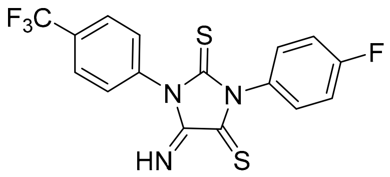

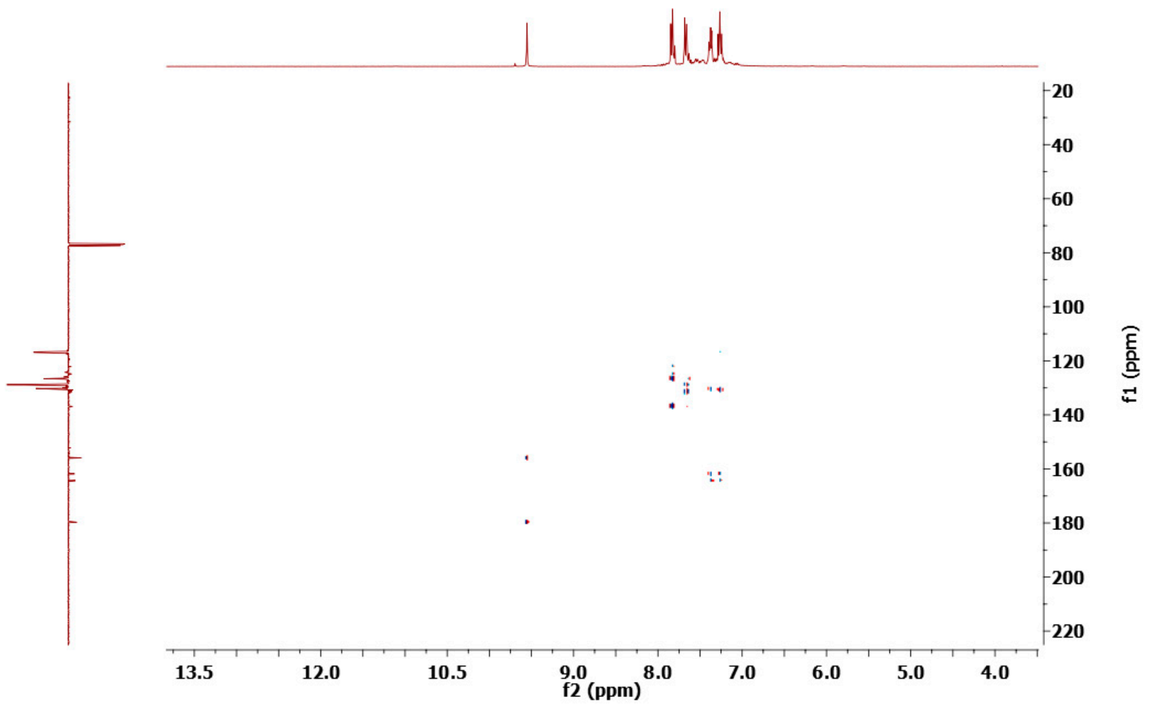

^{1}H NMR ($CDCl_3$) spectrum of 3-(4-fluorophenyl)-5-imino-1-(p-tolyl)imidazolidine-2,4-dithione (18b'')

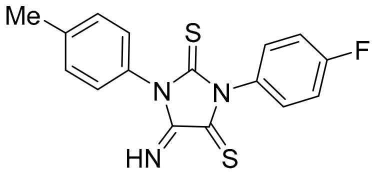

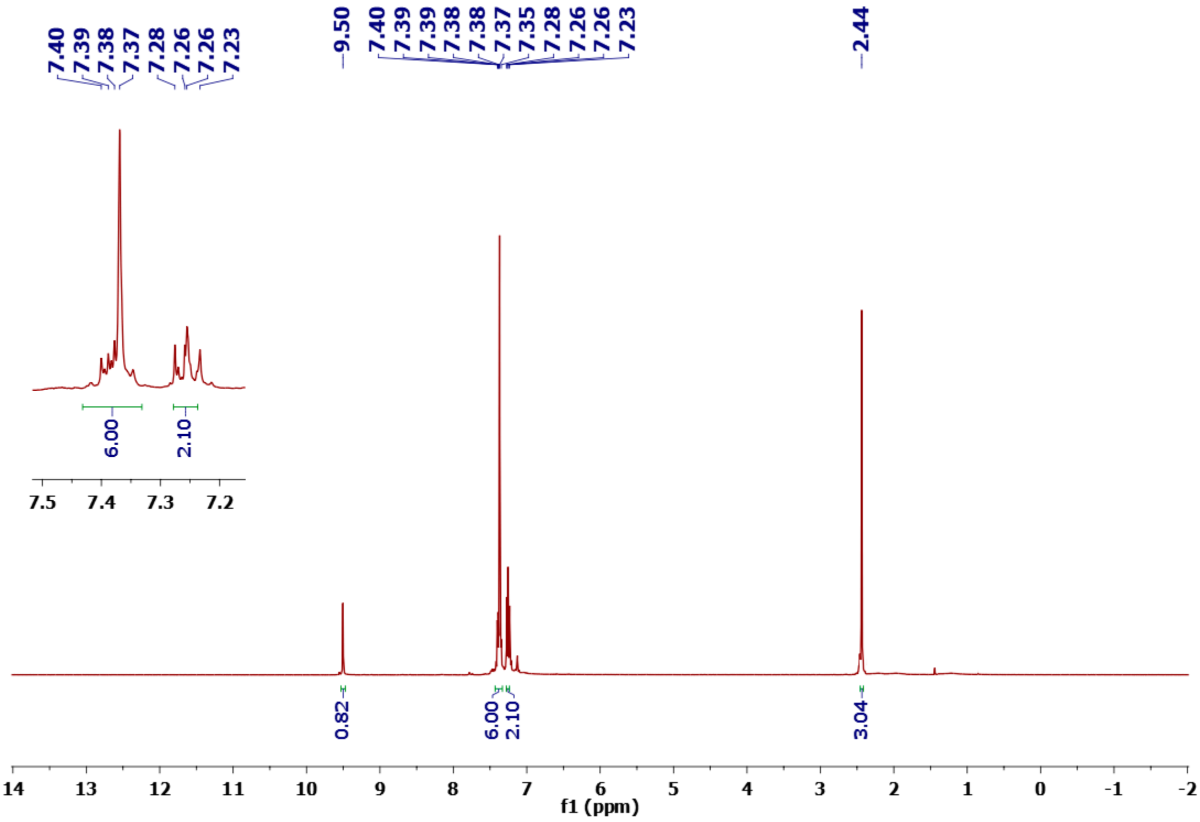

^{13}C NMR (CDCl$_3$) spectrum of 3-(4-fluorophenyl)-5-imino-1-(p-tolyl)imidazolidine-2,4-dithione

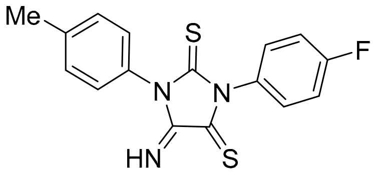

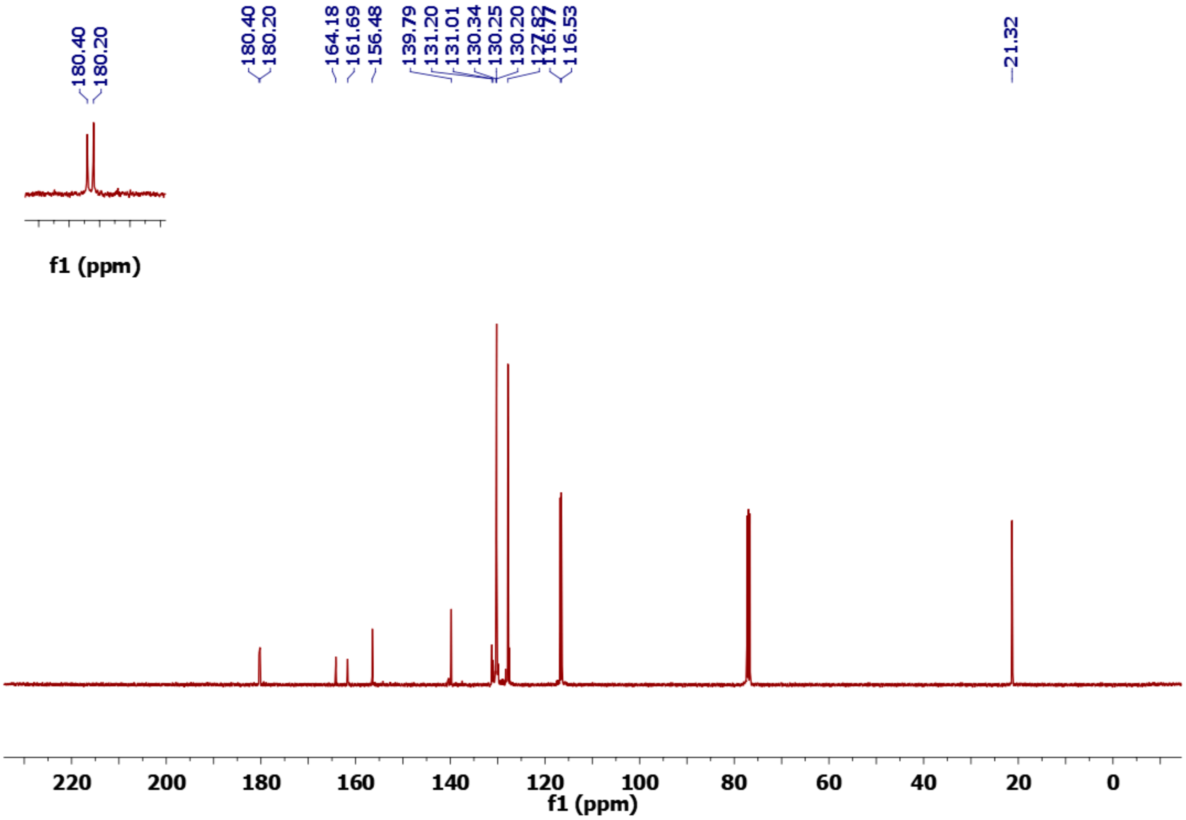

^{13}C-CRAPT NMR ($CDCl_3$) spectrum of 3-(4-fluorophenyl)-5-imino-1-(p-tolyl)imidazolidine-2,4-dithione

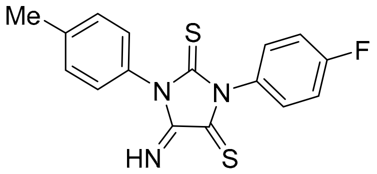

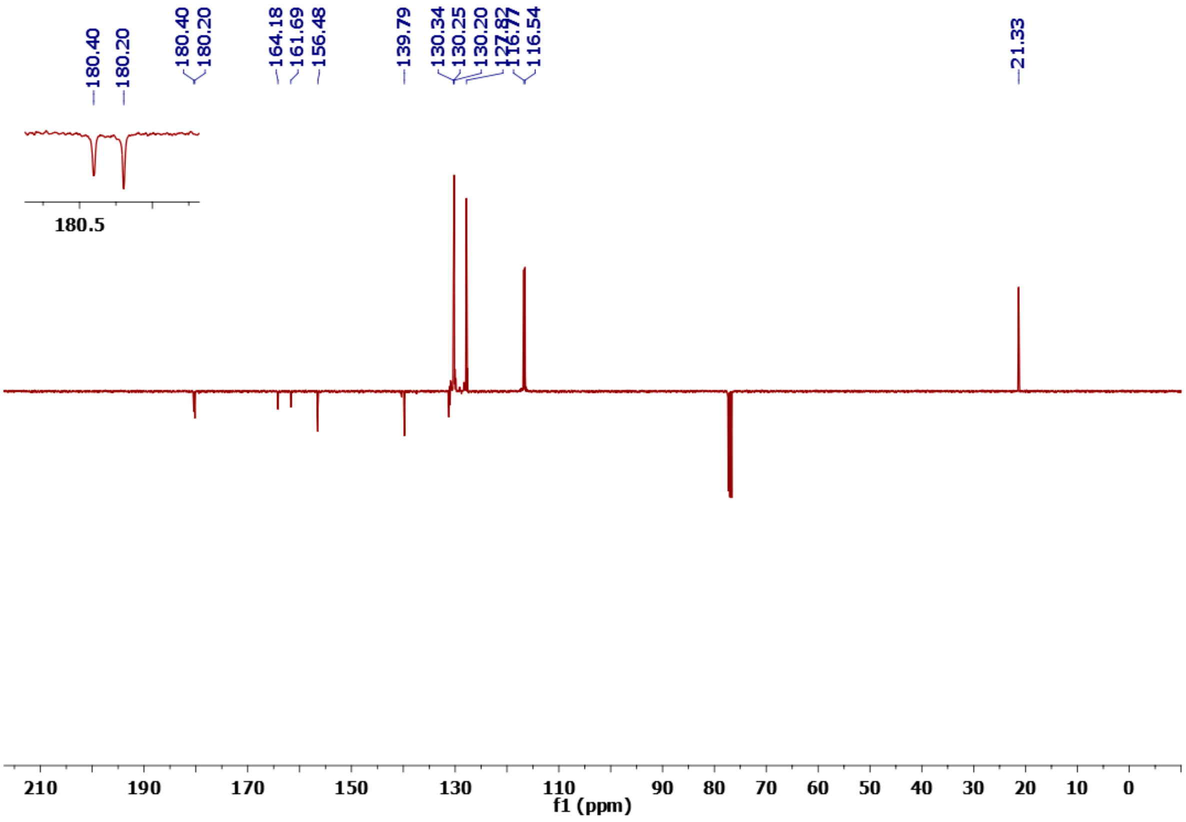

^{1}H-^{1}H-gCOSYAD NMR ($CDCl_3$) spectrum of 3-(4-fluorophenyl)-5-imino-1-(p-tolyl)imidazolidine-2,4-dithione

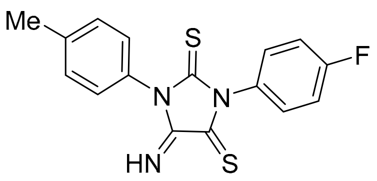

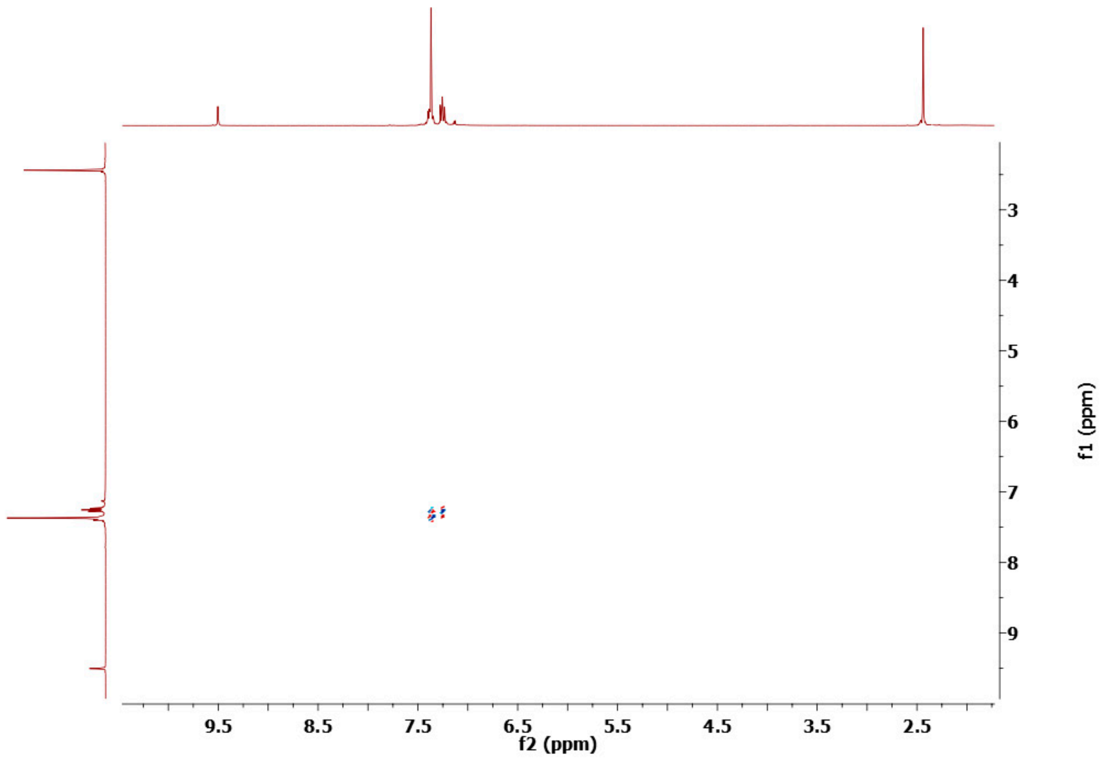

^{1}H-^{13}C-gHSQCAD NMR ($CDCl_3$) spectrum of 3-(4-fluorophenyl)-5-imino-1-(p-tolyl)imidazolidine-2,4-dithione

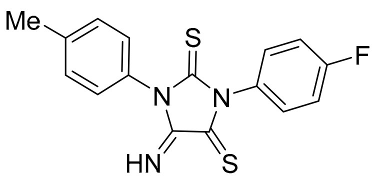

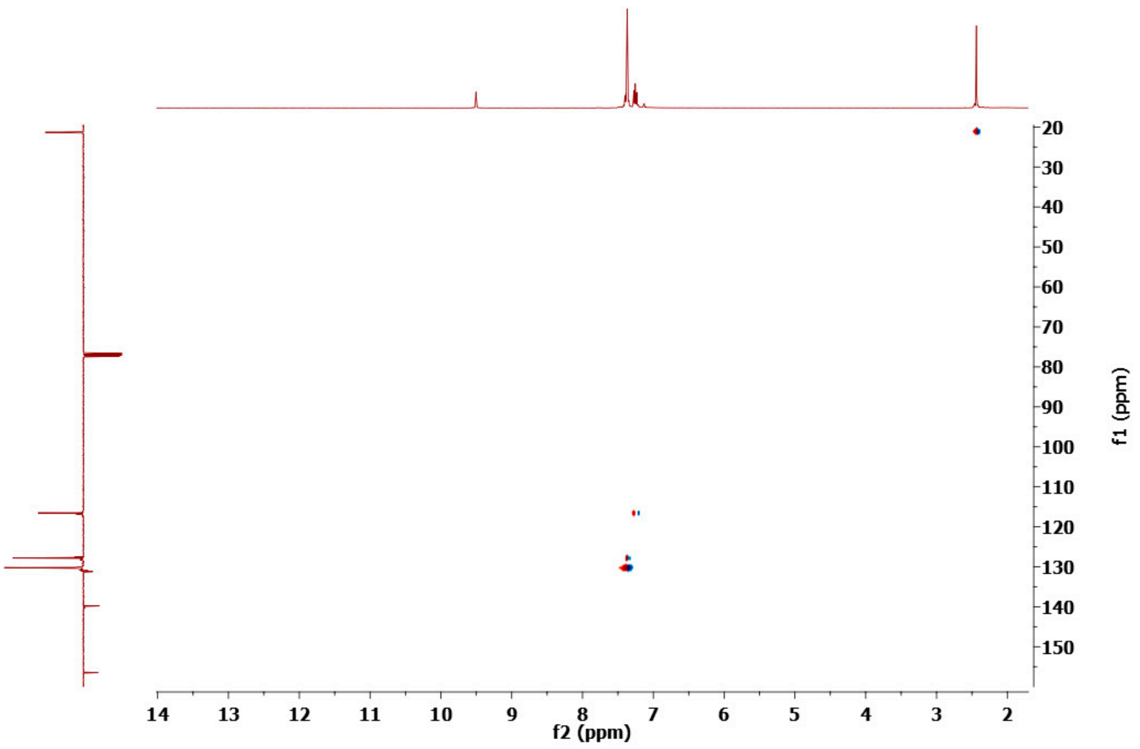

^{1}H-^{13}C-gHMBC NMR ($CDCl_3$) spectrum of 3-(4-fluorophenyl)-5-imino-1-(p-tolyl)imidazolidine-2,4-dithione

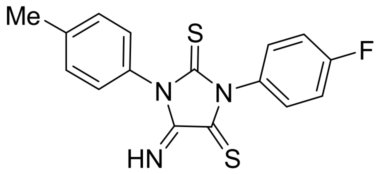

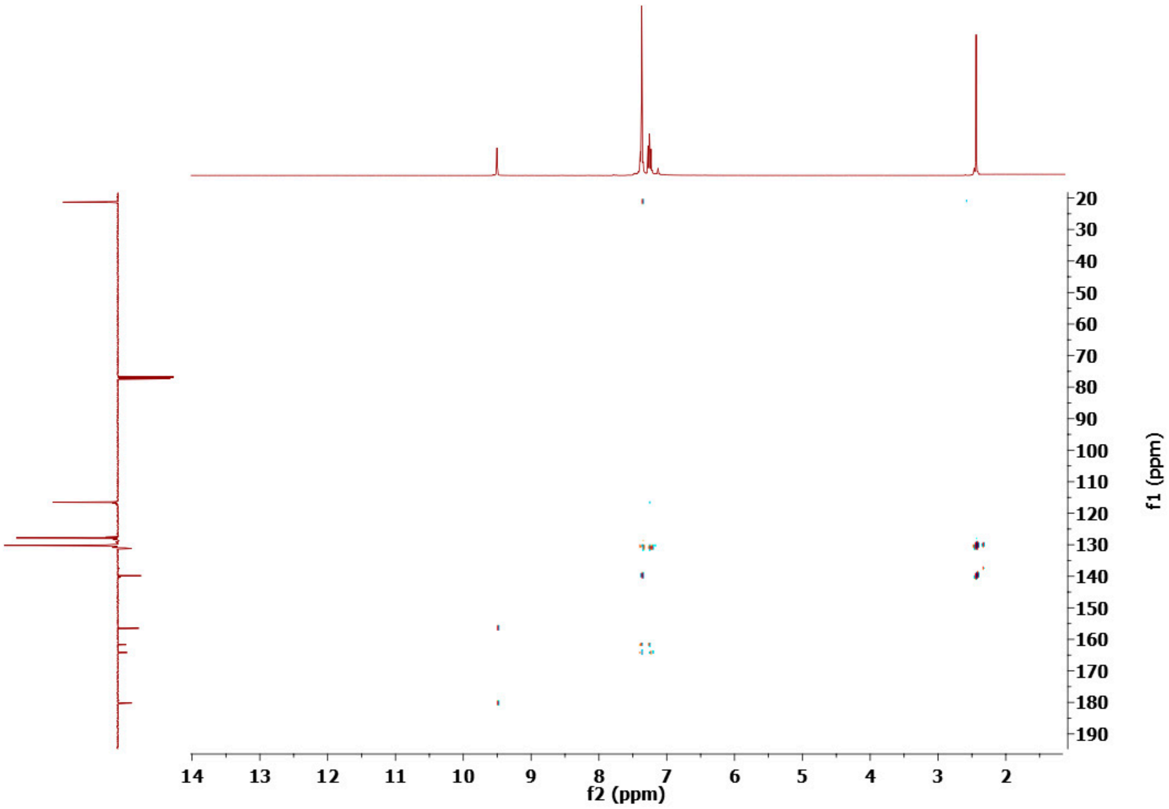

^{1}H NMR ($CDCl_3$) spectrum of 1-(4-chlorophenyl)-3-(4-fluorophenyl)-5-iminoimidazolidine-2,4-dithione (18c'')

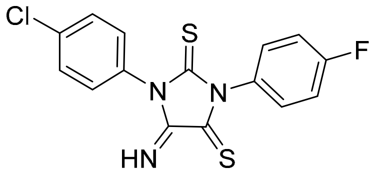

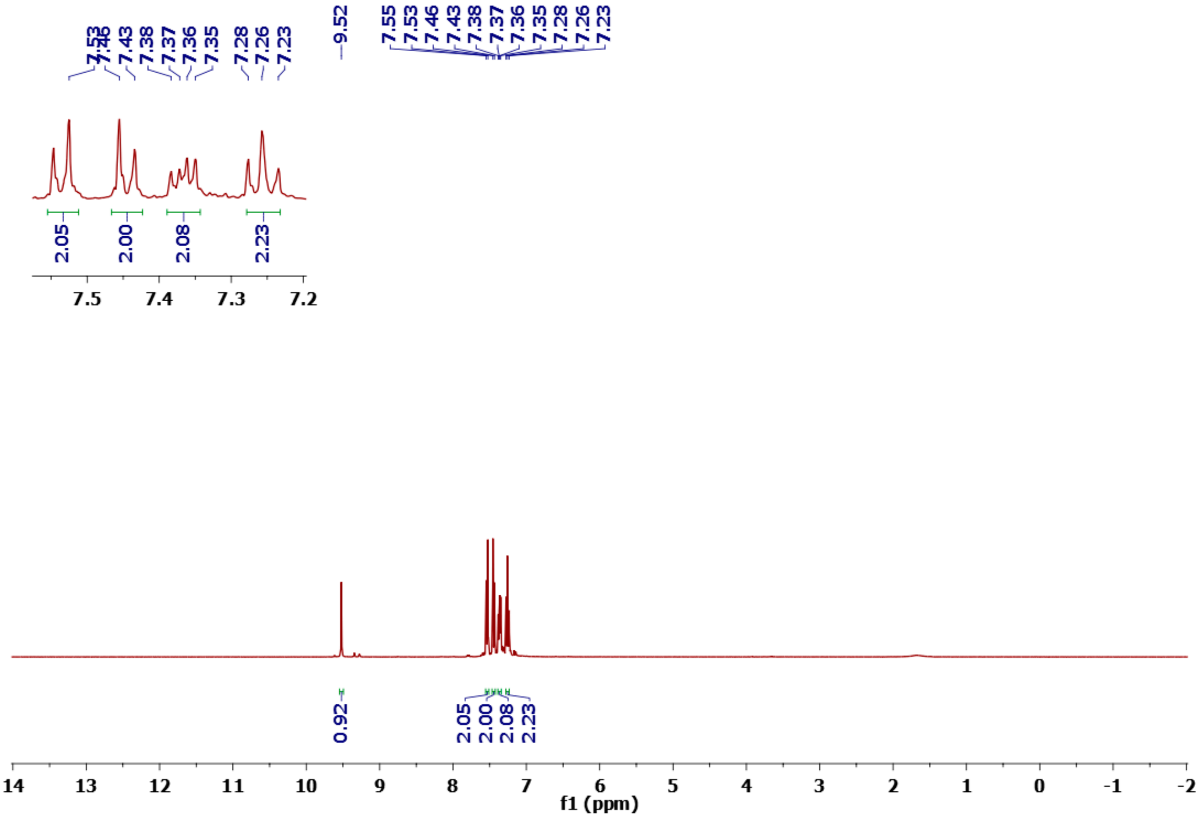

^{13}C NMR ($CDCl_3$) spectrum of 1-(4-chlorophenyl)-3-(4-fluorophenyl)-5-iminoimidazolidine-2,4-dithione

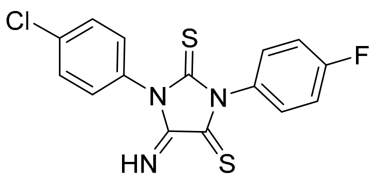

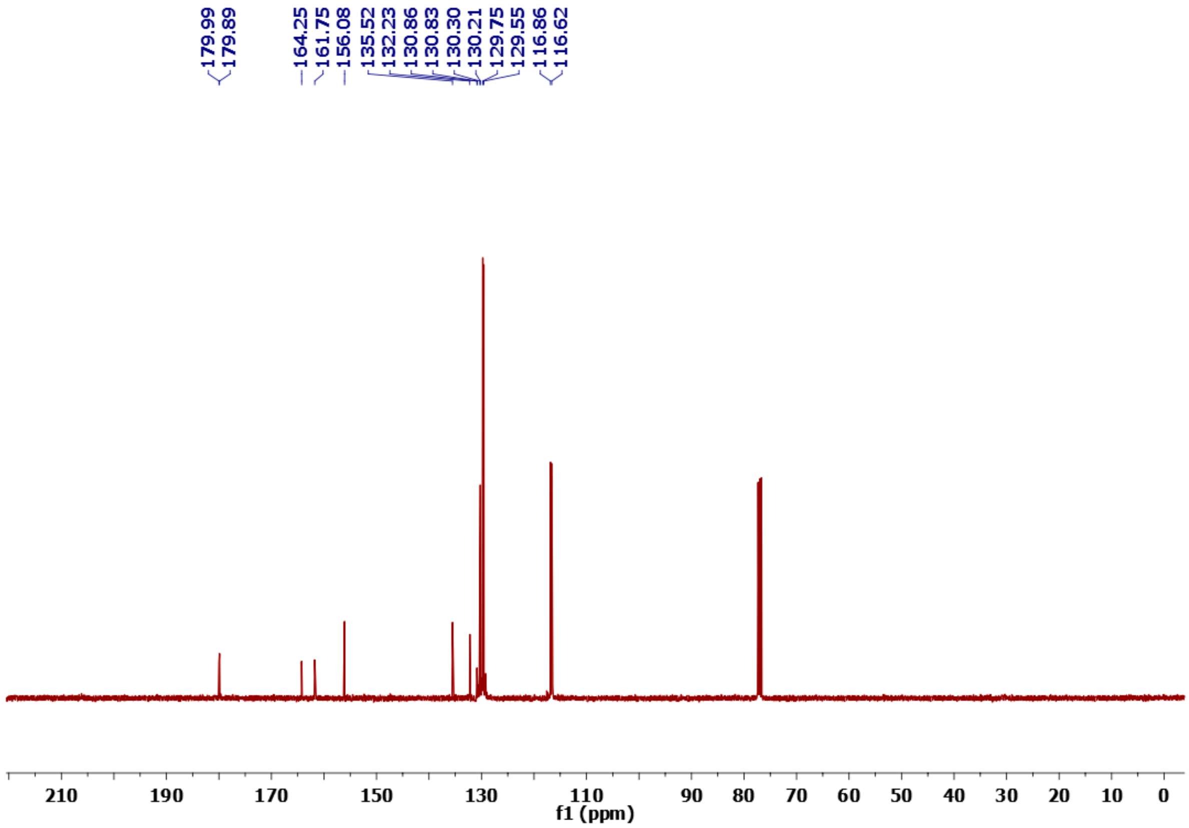

^{13}C-CRAPT NMR ($CDCl_3$) spectrum of 1-(4-chlorophenyl)-3-(4-fluorophenyl)-5-iminoimidazolidine-2,4-dithione

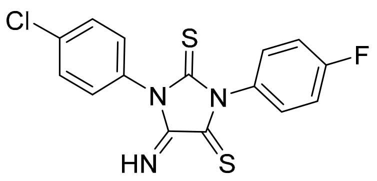

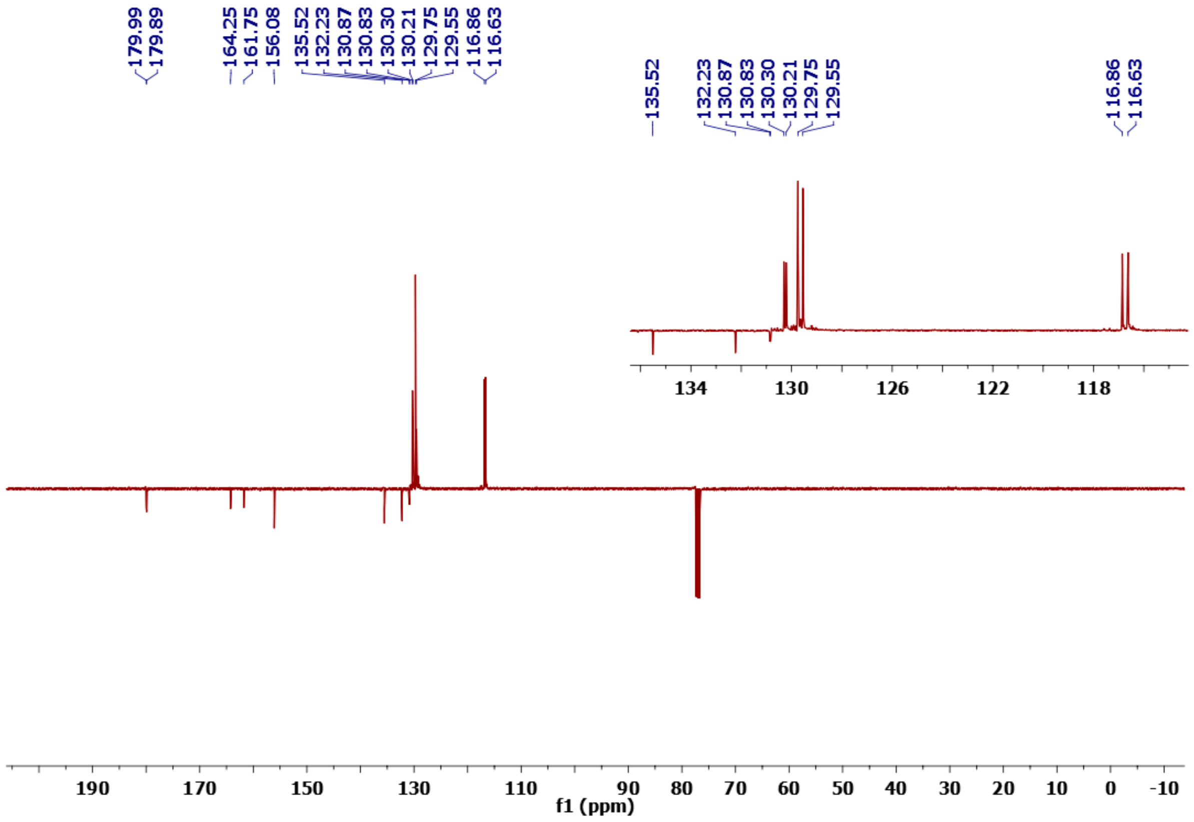

^{1}H-^{1}H-gCOSYAD NMR (CDCl$_3$) spectrum of 1-(4-chlorophenyl)-3-(4-fluorophenyl)-5-iminoimidazolidine-2,4-dithione

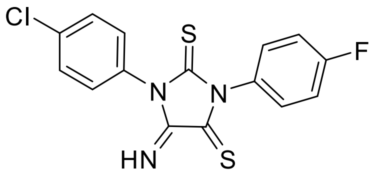

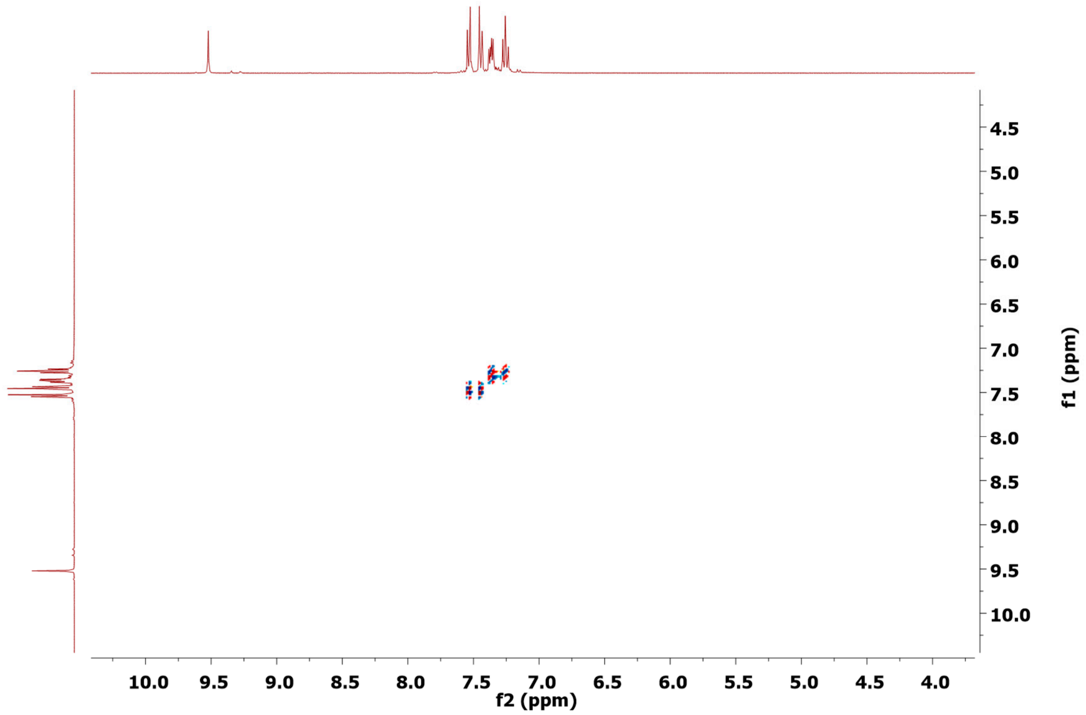

^{1}H-^{13}C-gHSQCAD NMR ($CDCl_3$) spectrum of 1-(4-chlorophenyl)-3-(4-fluorophenyl)-5-iminoimidazolidine-2,4-dithione

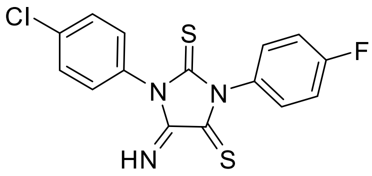

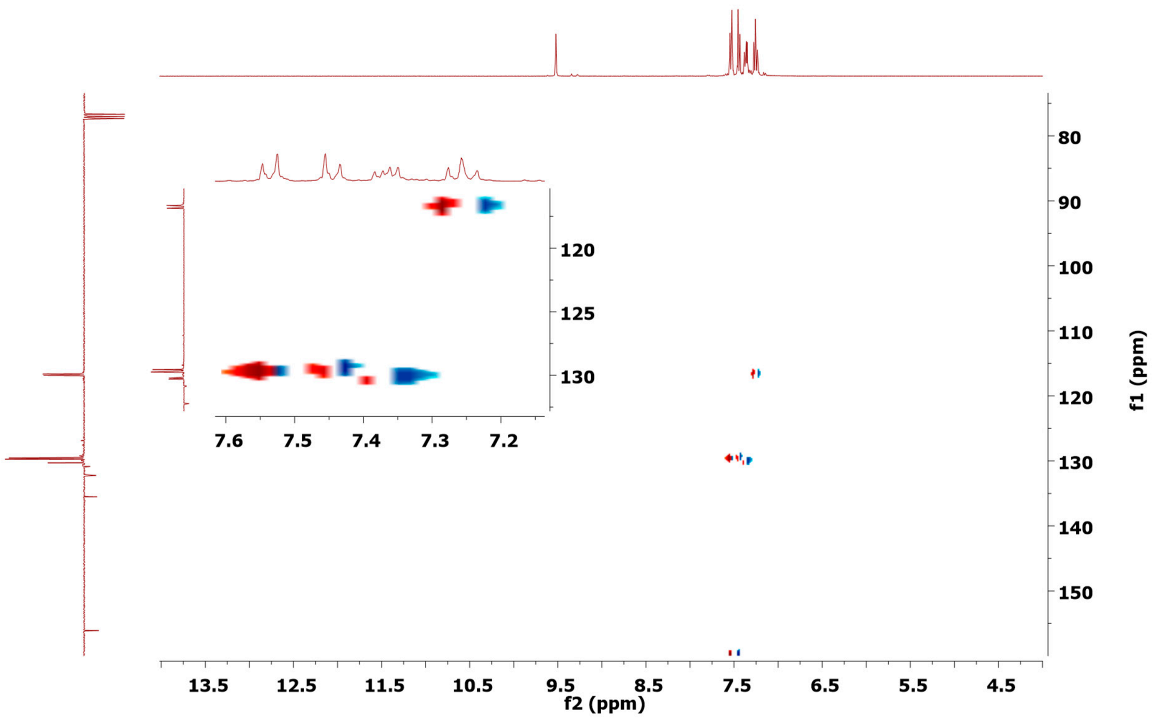

^{1}H-^{13}C-gHMBC NMR ($CDCl_3$) spectrum of 1-(4-chlorophenyl)-3-(4-fluorophenyl)-5-iminoimidazolidine-2,4-dithione

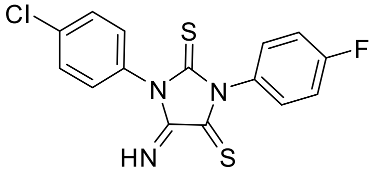

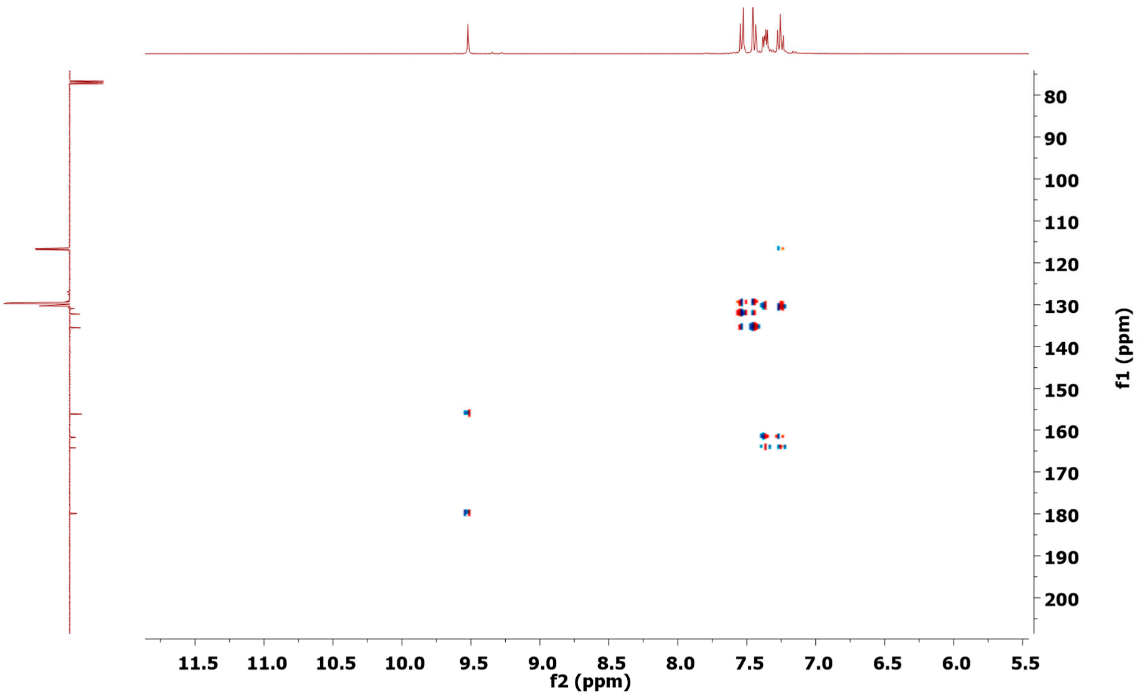

^{1}H NMR ($CDCl_3$) spectrum of 3-(4-fluorophenyl)-5-imino-1-(4-methoxyphenyl)imidazolidine-2,4-dithione (18d'')

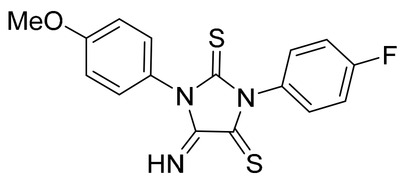

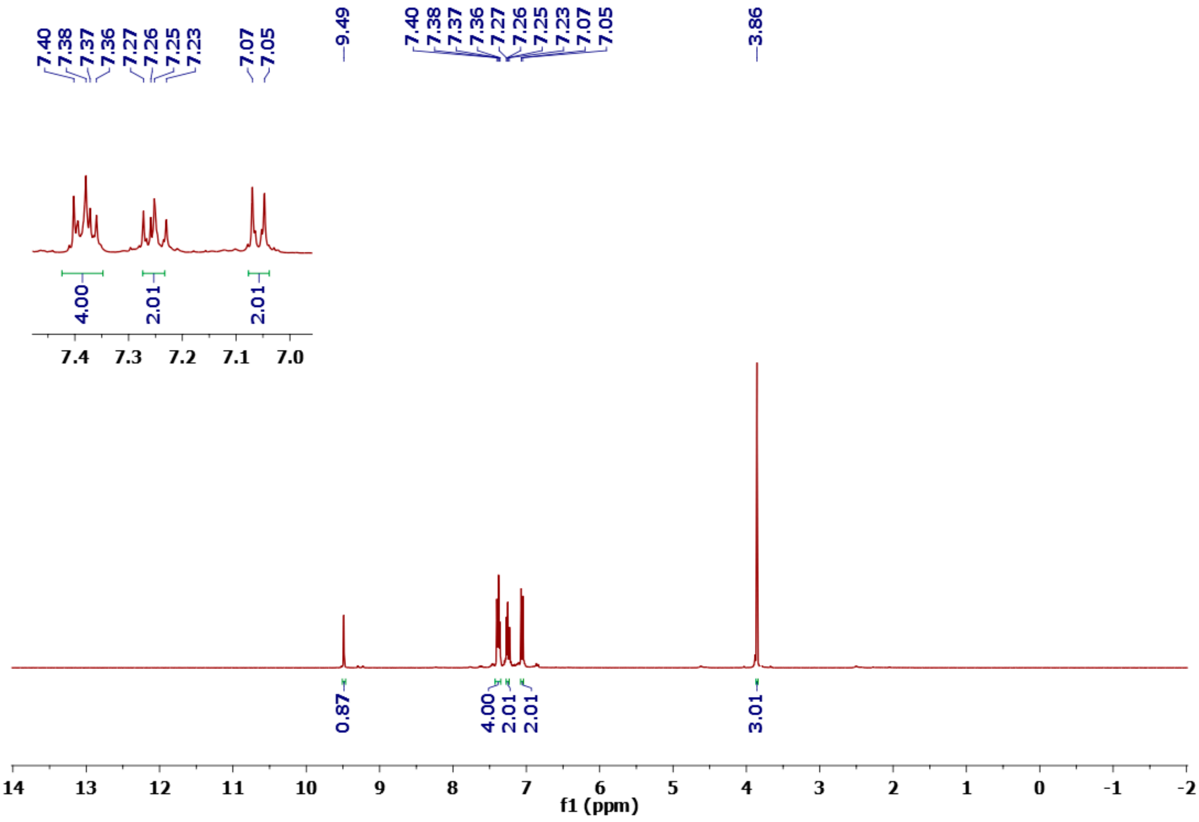

^{13}C NMR ($CDCl_3$) spectrum of 3-(4-fluorophenyl)-5-imino-1-(4-methoxyphenyl)imidazolidine-2,4-dithione

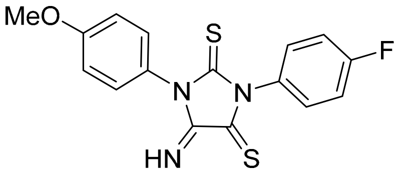

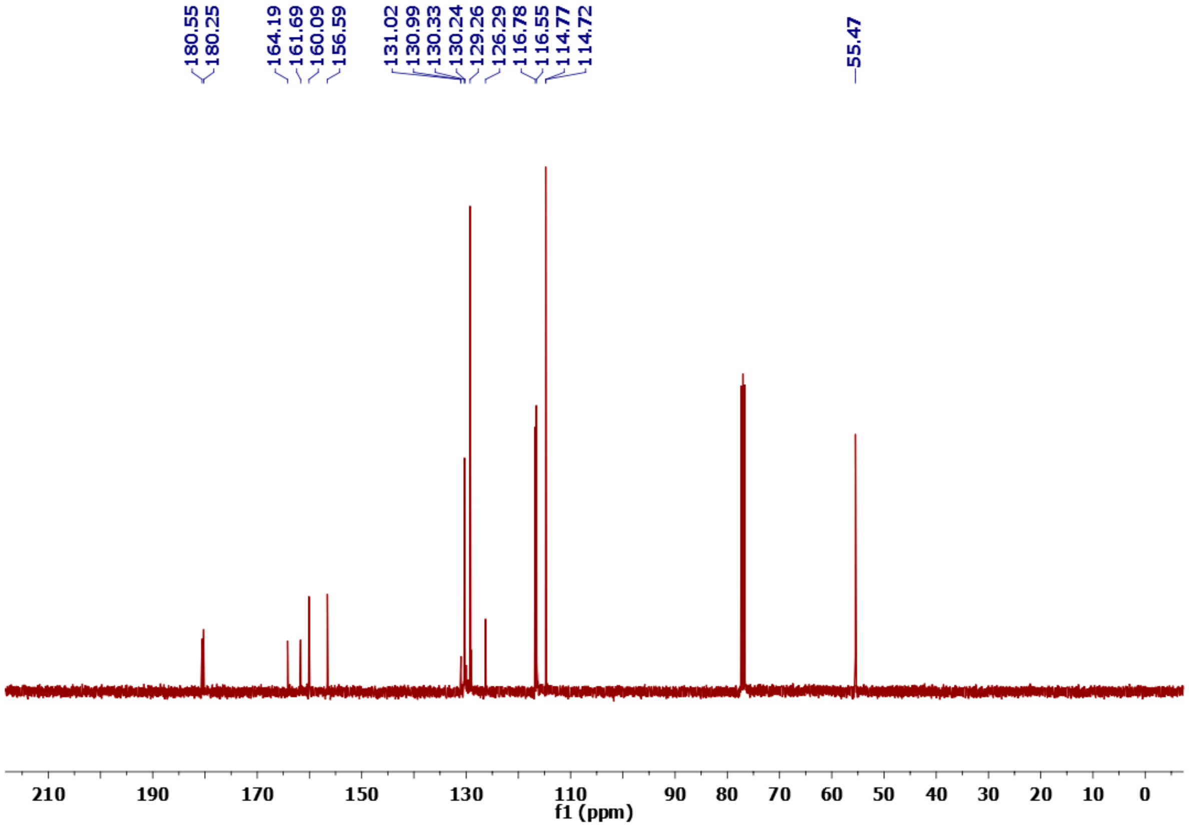

^{13}C-CRAPT NMR ($CDCl_3$) spectrum of 3-(4-fluorophenyl)-5-imino-1-(4-methoxyphenyl)imidazolidine-2,4-dithione

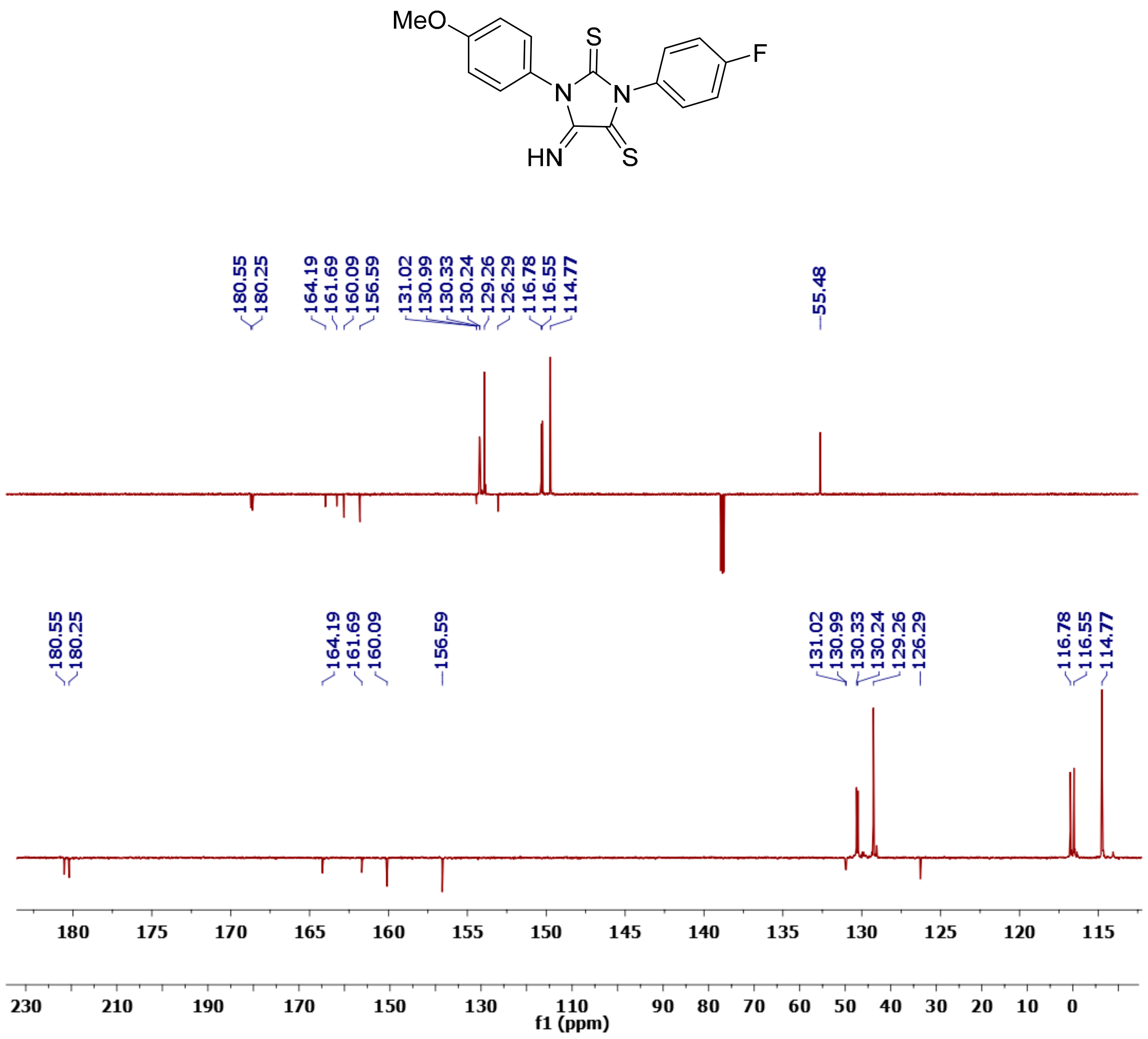

^{1}H-^{1}H-gCOSYAD NMR ($CDCl_3$) spectrum of 3-(4-fluorophenyl)-5-imino-1-(4-methoxyphenyl)imidazolidine-2,4-dithione

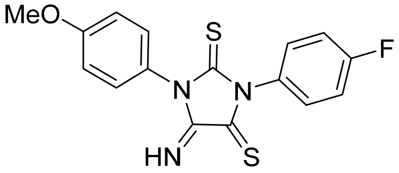

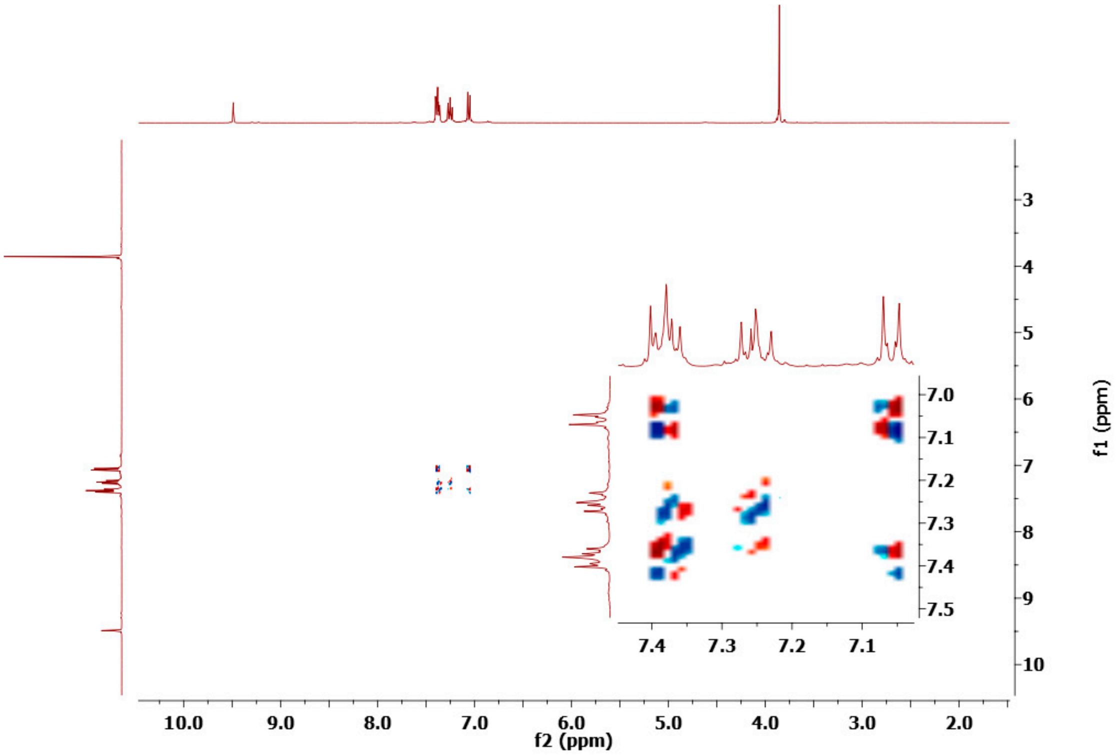

^{1}H-^{13}C-gHSQCAD NMR ($CDCl_3$) spectrum of 3-(4-fluorophenyl)-5-imino-1-(4-methoxyphenyl)imidazolidine-2,4-dithione

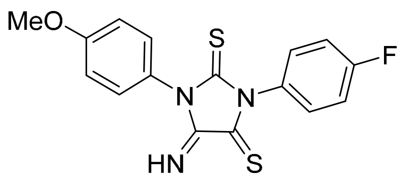

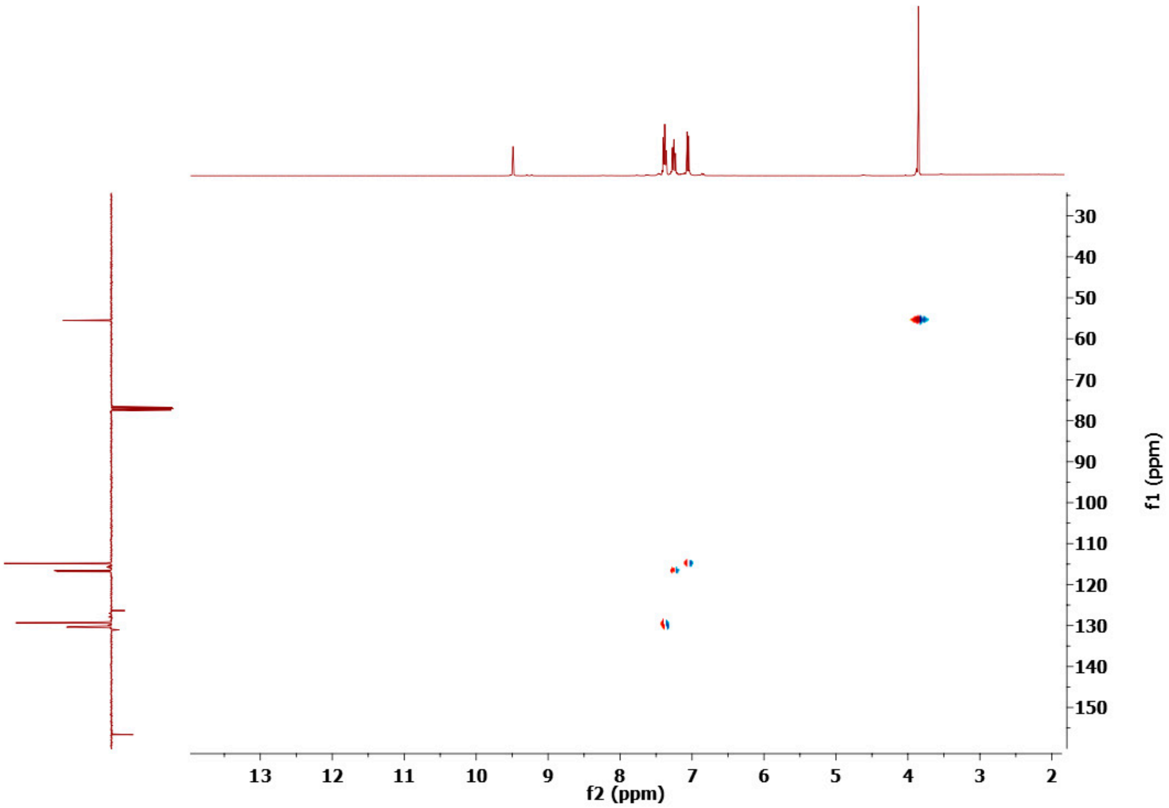

^{1}H-^{13}C-gHMBC NMR ($CDCl_3$) spectrum of 3-(4-fluorophenyl)-5-imino-1-(4-methoxyphenyl)imidazolidine-2,4-dithione

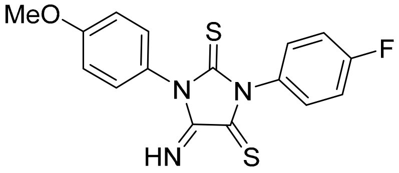

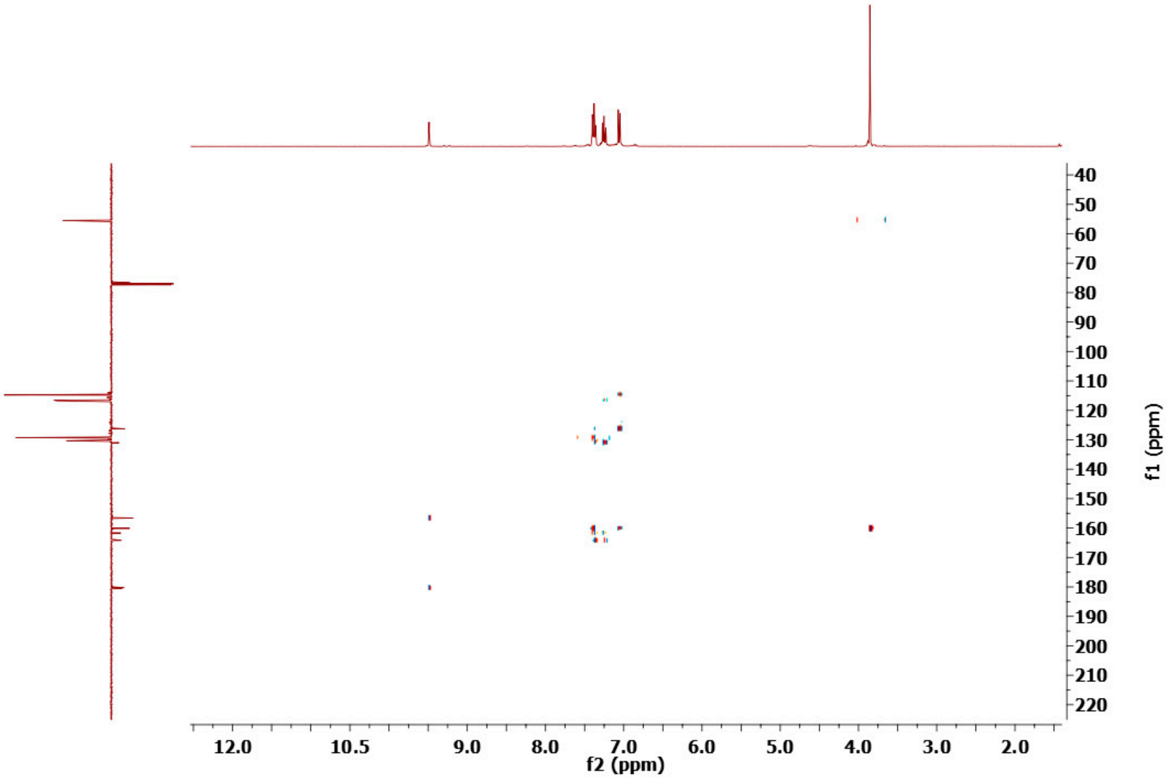

^{1}H NMR (CDCl$_3$) spectrum of 3-(4-fluorophenyl)-5-imino-1-(4-nitrophenyl)imidazolidine-2,4-dithione (18e'')

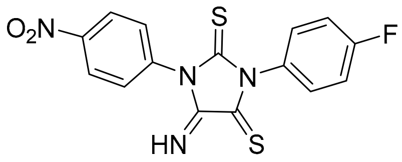

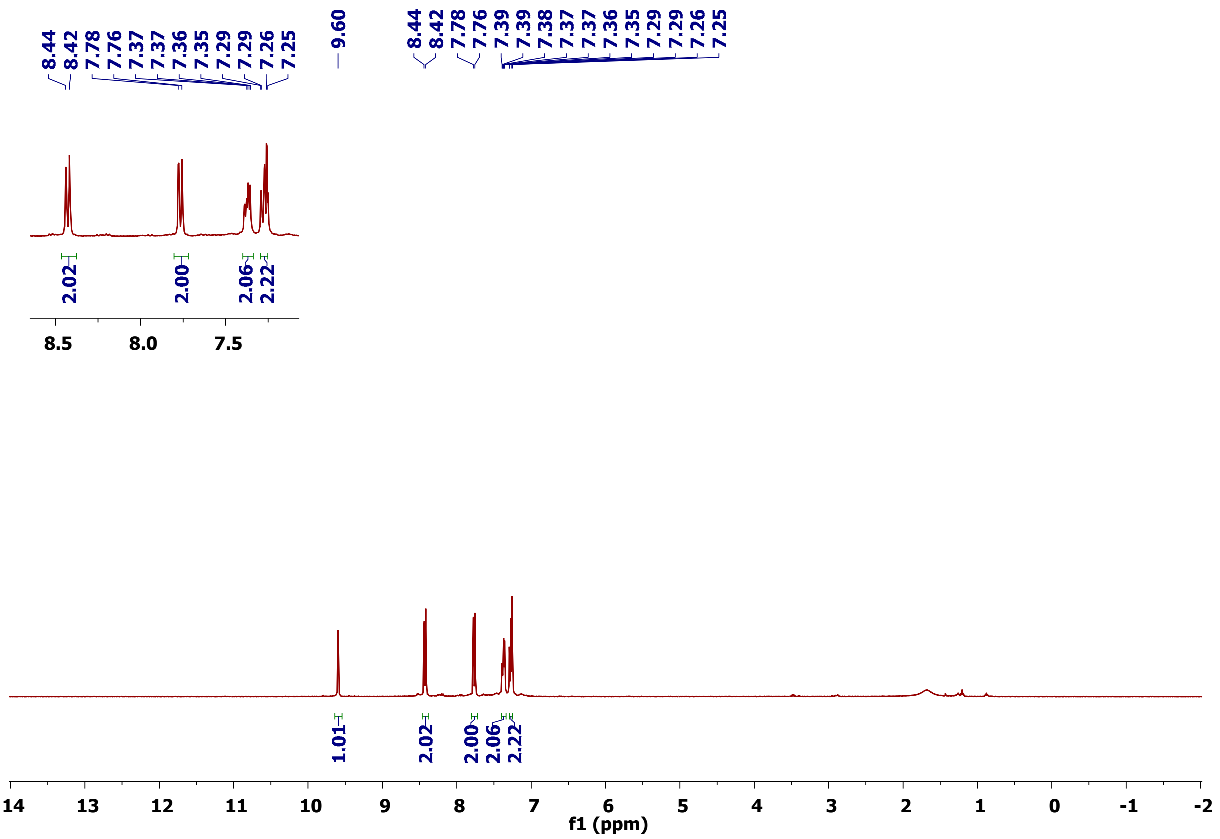

^{13}C NMR ($CDCl_3$) spectrum of 3-(4-fluorophenyl)-5-imino-1-(4-nitrophenyl)imidazolidine-2,4-dithione

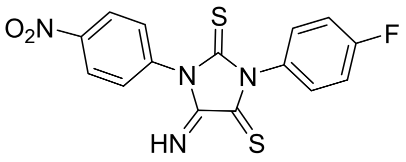

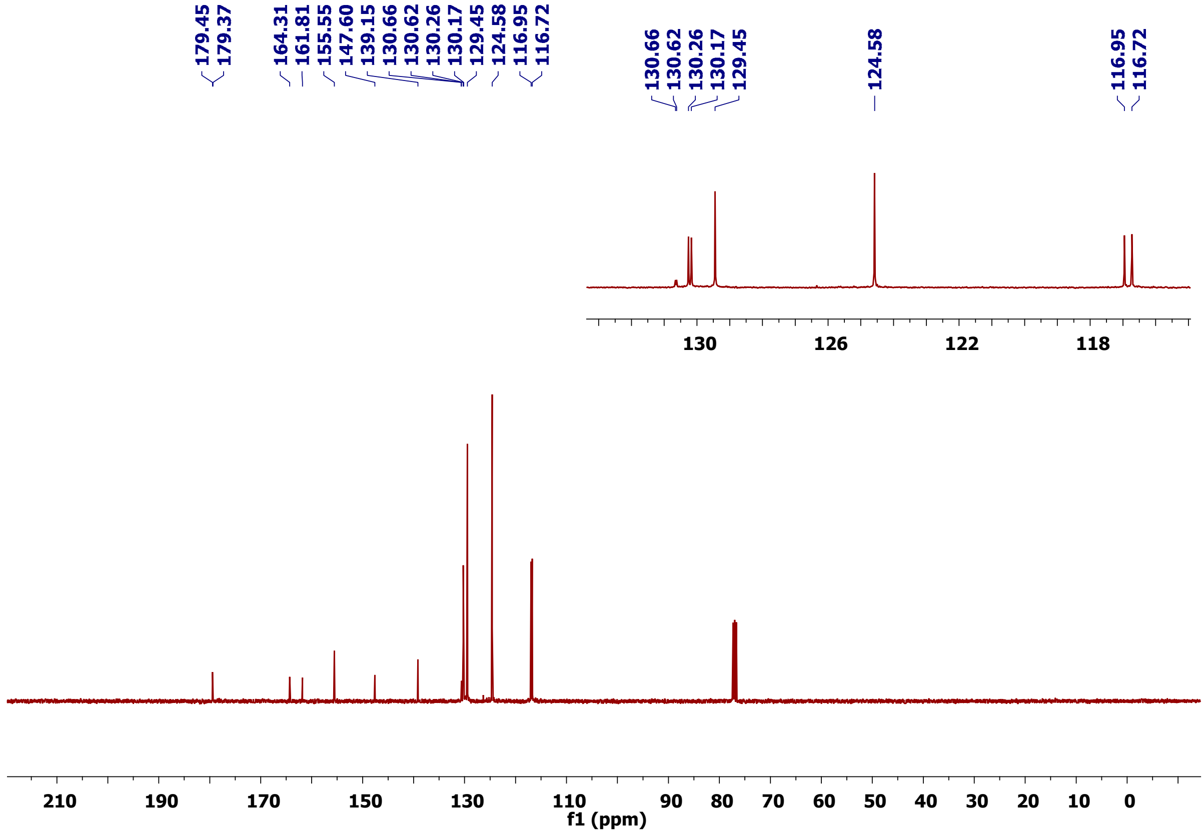

^{13}C-CRAPT NMR ($CDCl_3$) spectrum of 3-(4-fluorophenyl)-5-imino-1-(4-nitrophenyl)imidazolidine-2,4-dithione

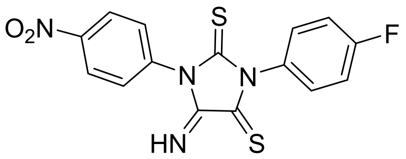

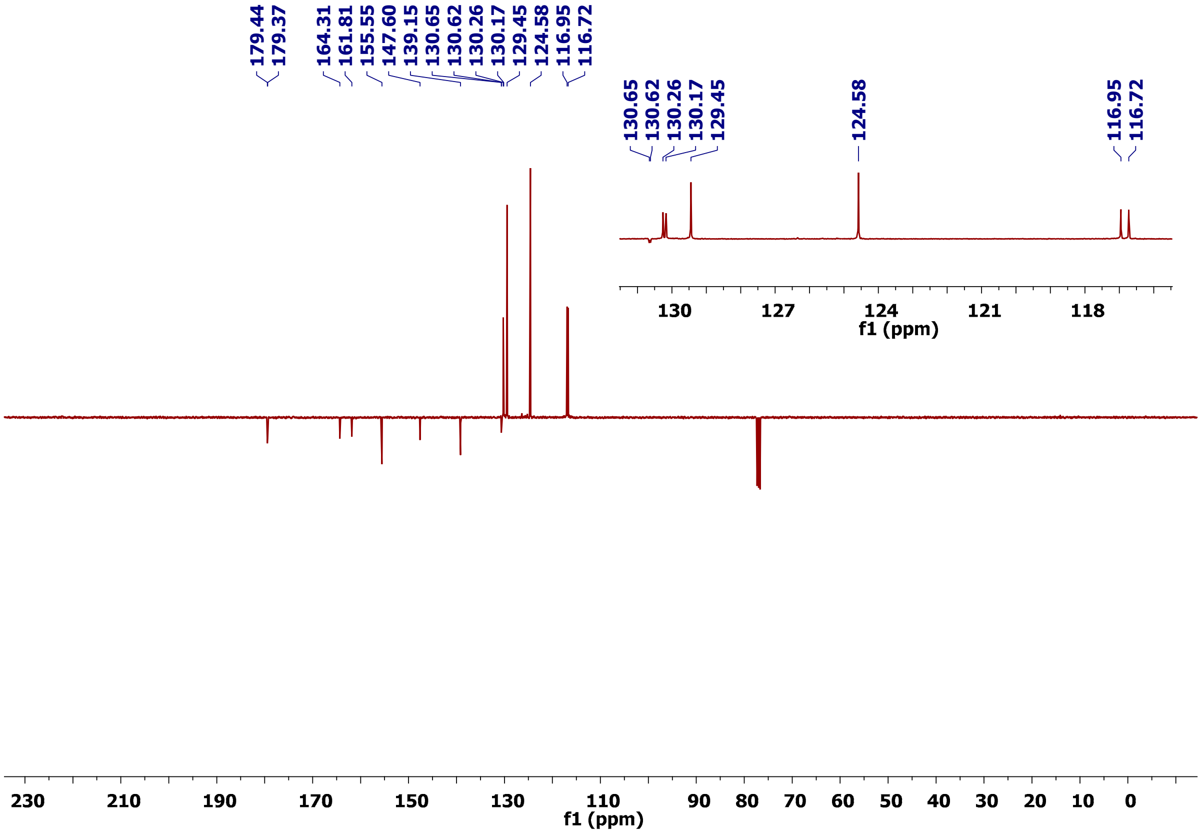

^{1}H-^{1}H-gCOSYAD NMR ($CDCl_3$) spectrum of 3-(4-fluorophenyl)-5-imino-1-(4-nitrophenyl)imidazolidine-2,4-dithione

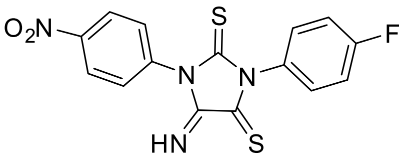

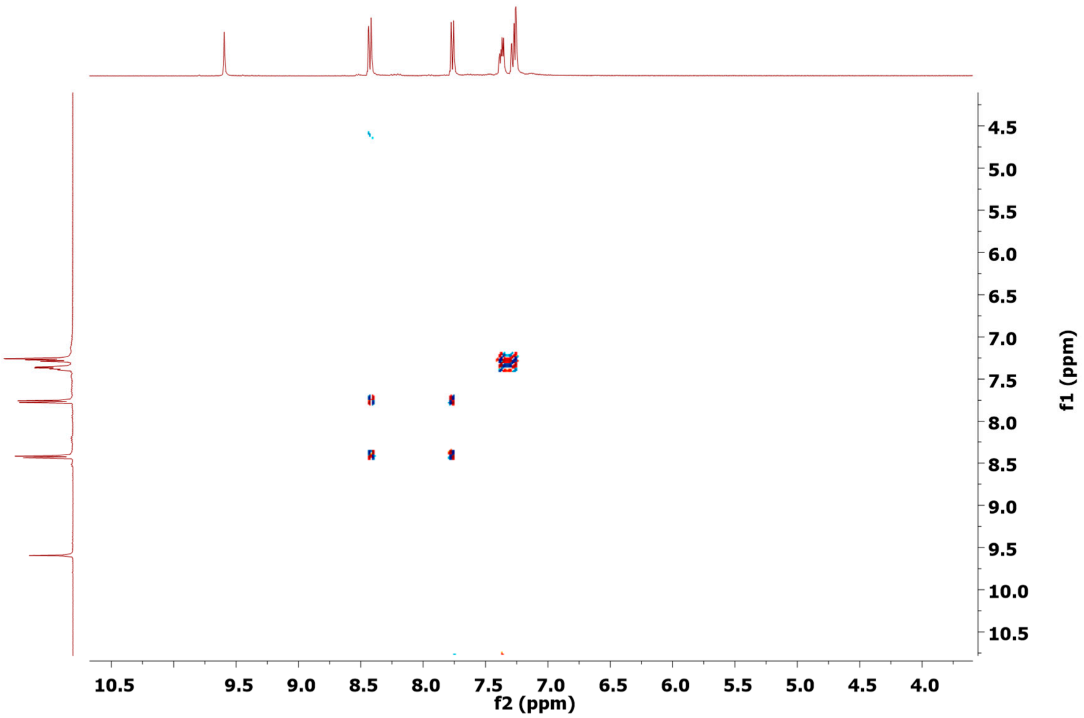

^{1}H-^{13}C-gHSQCAD NMR ($CDCl_3$) spectrum of 3-(4-fluorophenyl)-5-imino-1-(4-nitrophenyl)imidazolidine-2,4-dithione

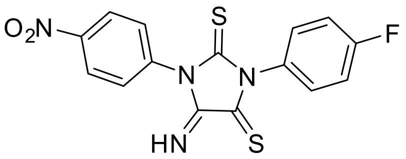

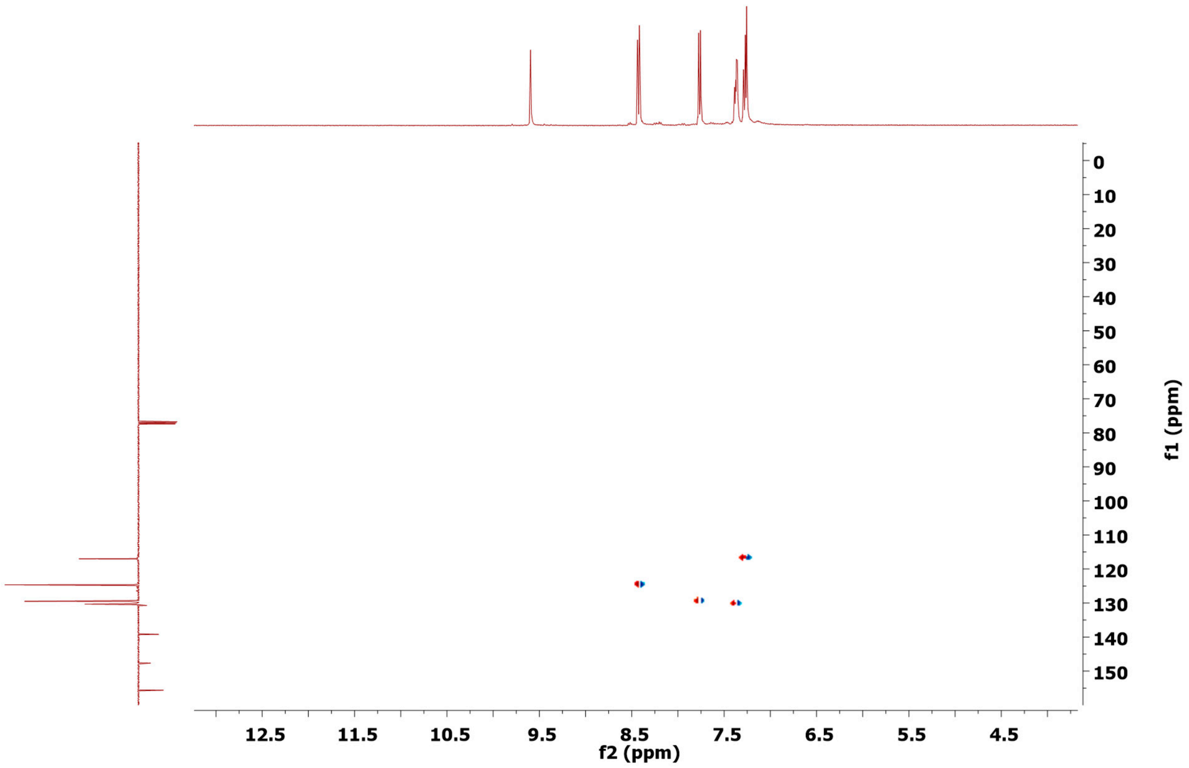

^{1}H-^{13}C-gHMBC NMR ($CDCl_3$) spectrum of 3-(4-fluorophenyl)-5-imino-1-(4-nitrophenyl)imidazolidine-2,4-dithione

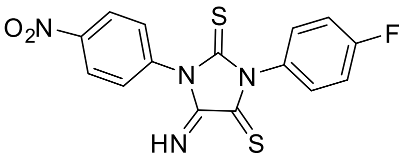

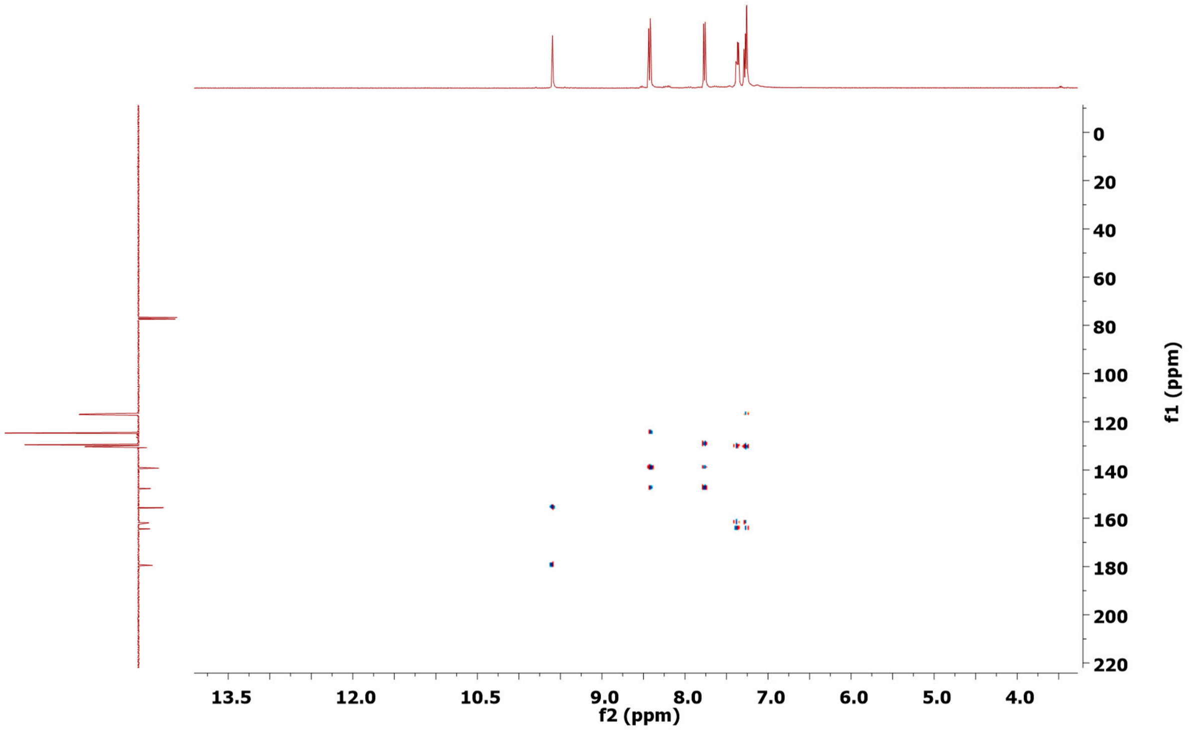

^{1}H NMR ($CDCl_3$) spectrum of 1-(3-fluorophenyl)-5-imino-3-(p-tolyl)imidazolidine-2,4-dithione (18f'')

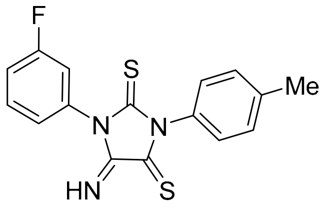

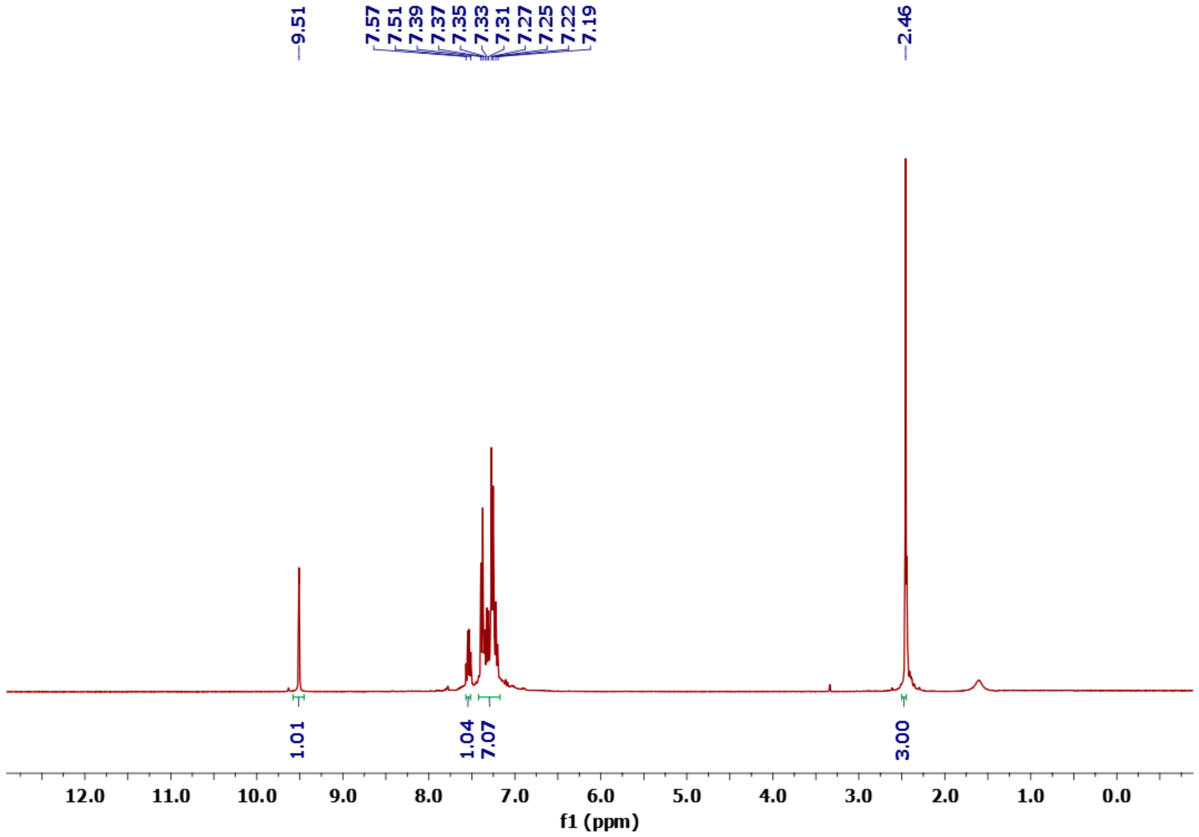

^{13}C-CRAPT NMR ($CDCl_3$) spectrum of 1-(3-fluorophenyl)-5-imino-3-(p-tolyl)imidazolidine-2,4-dithione

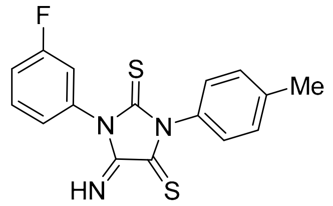

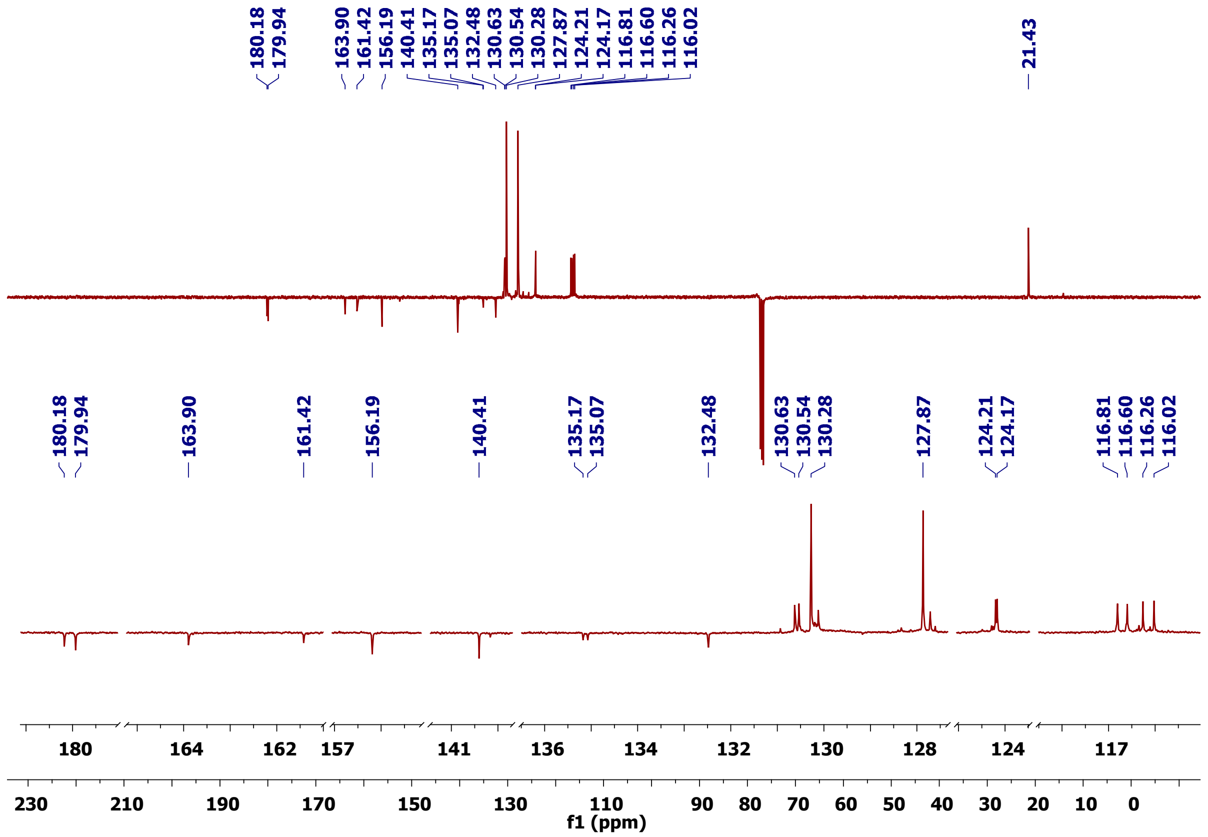

^{13}C NMR ($CDCl_3$) spectrum of 1-(3-fluorophenyl)-5-imino-3-(p-tolyl)imidazolidine-2,4-dithione

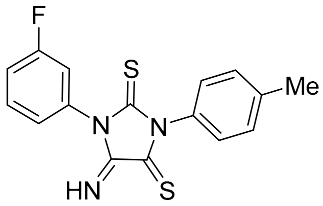

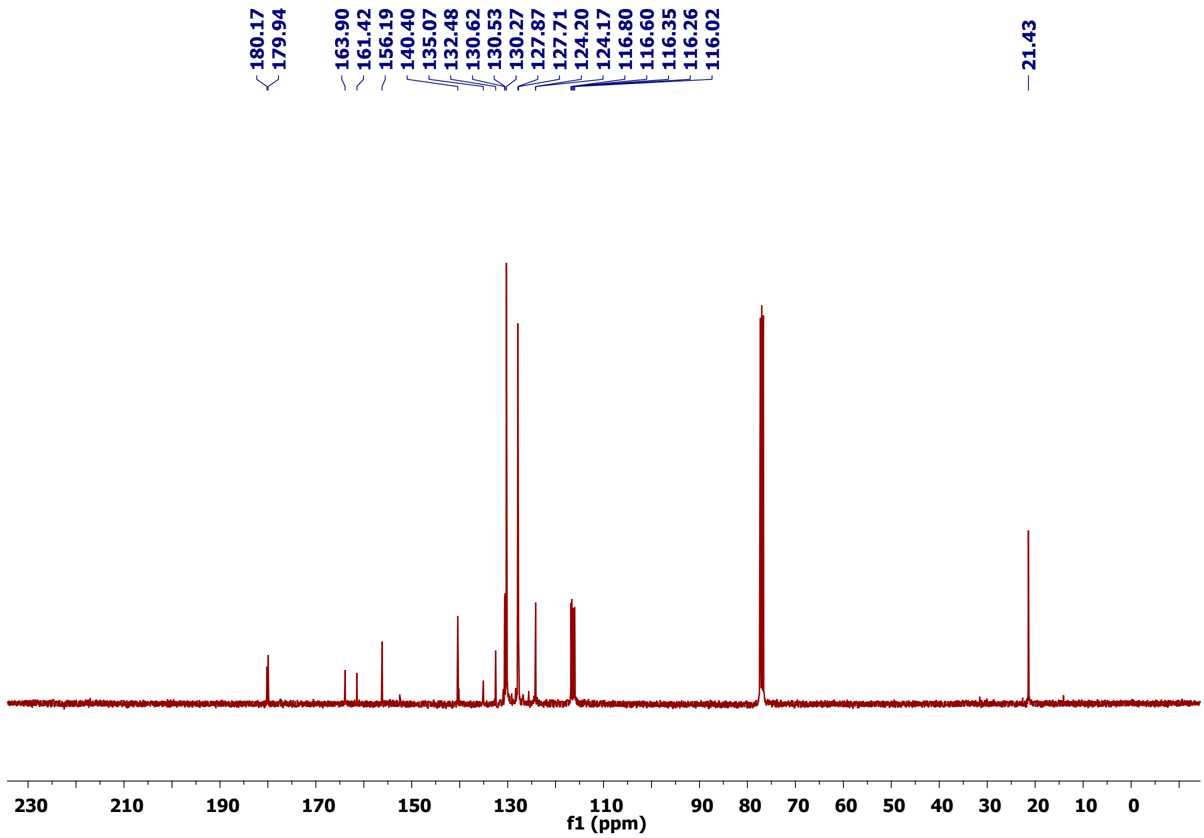

^{1}H-^{1}H-gCOSYAD NMR ($CDCl_3$) spectrum of 1-(3-fluorophenyl)-5-imino-3-(p-tolyl)imidazolidine-2,4-dithione

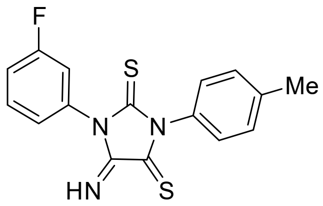

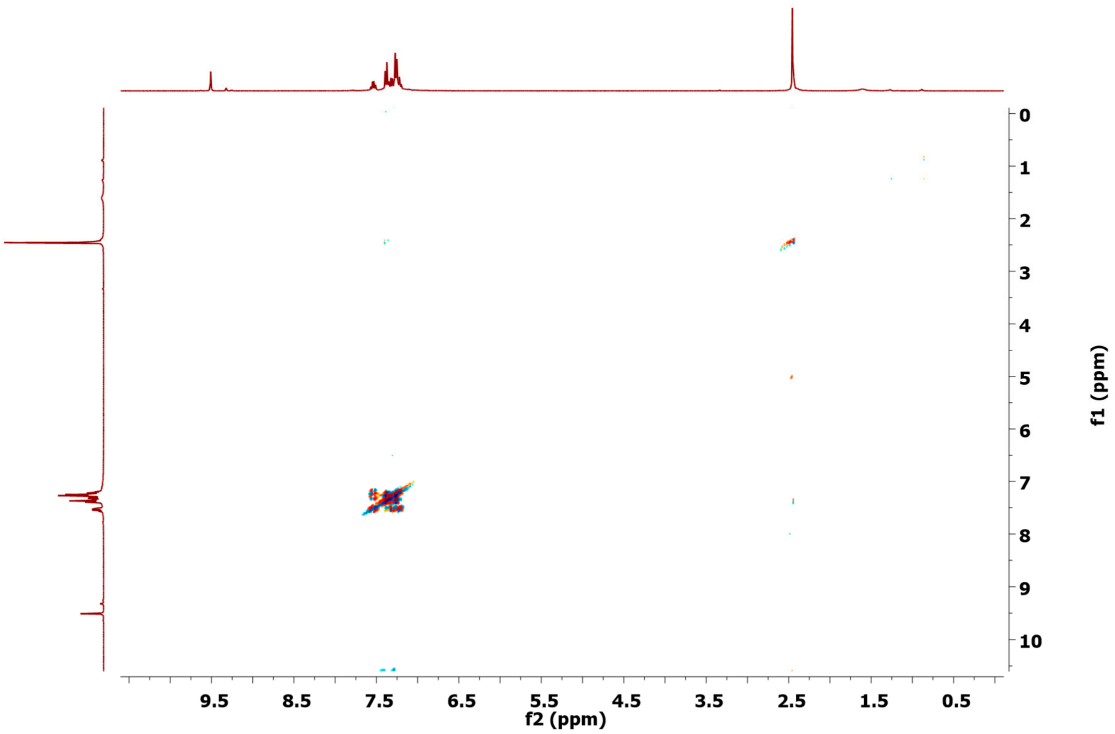

^{1}H-^{13}C-gHSQCAD NMR ($CDCl_3$) spectrum of 1-(3-fluorophenyl)-5-imino-3-(p-tolyl)imidazolidine-2,4-dithione

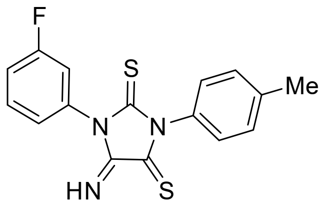

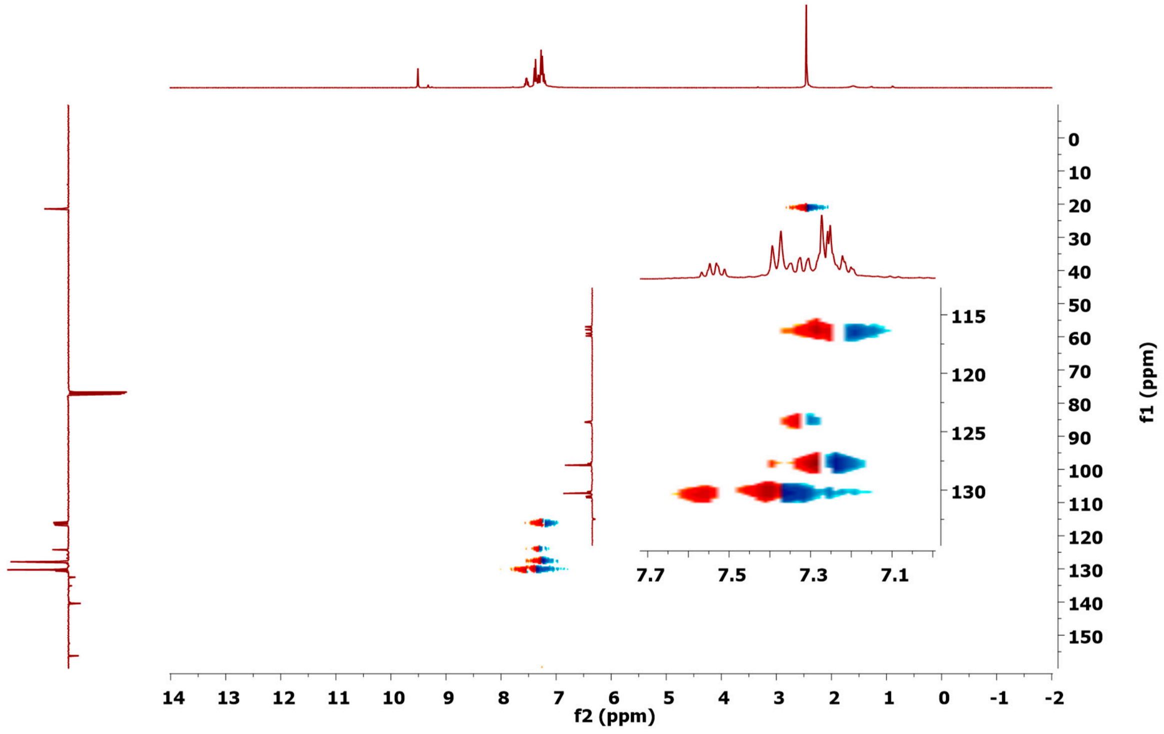

^{1}H-^{13}C-gHMBC NMR ($CDCl_3$) spectrum of 1-(3-fluorophenyl)-5-imino-3-(p-tolyl)imidazolidine-2,4-dithione

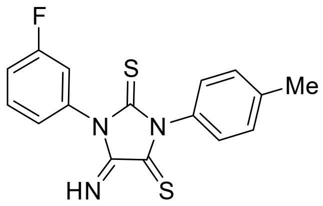

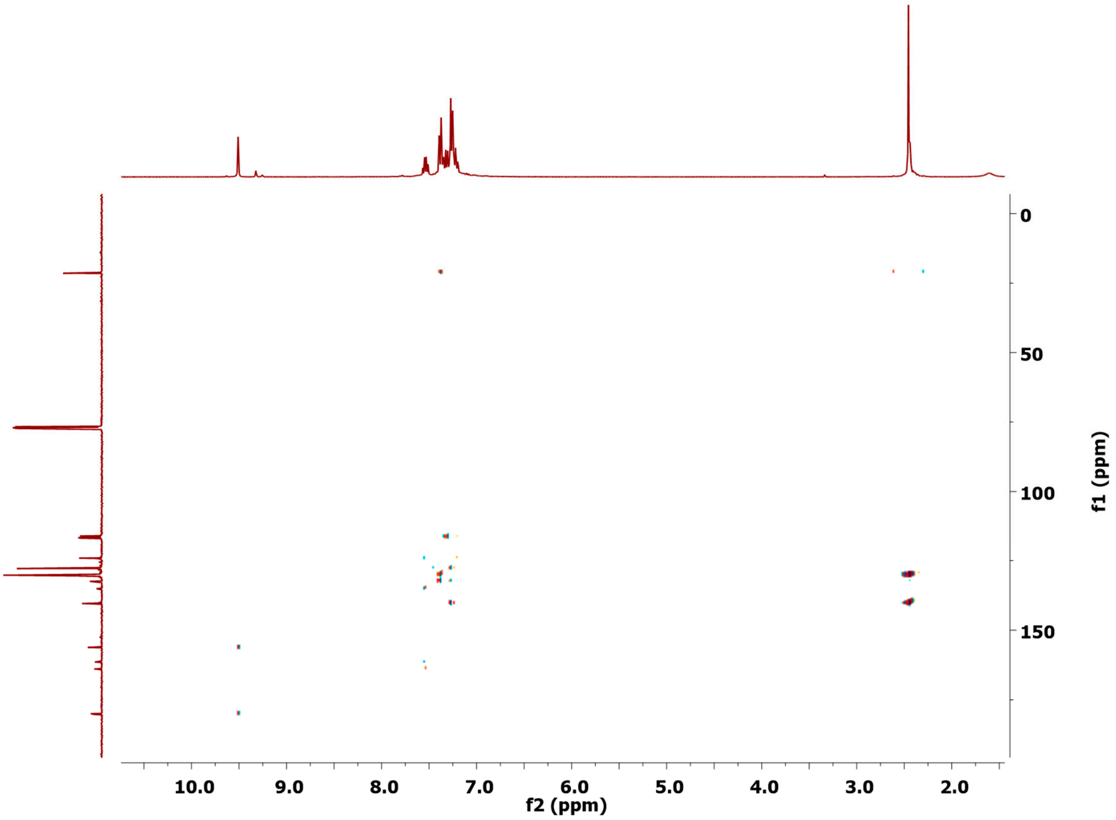

Ratio of 5-imino-1,3-diphenylimidazolidine-2,4-dithione and (Z)-4-imino-3-phenyl-5-(phenylimino)thiazolidine-2-thione in nitromethane as solvent

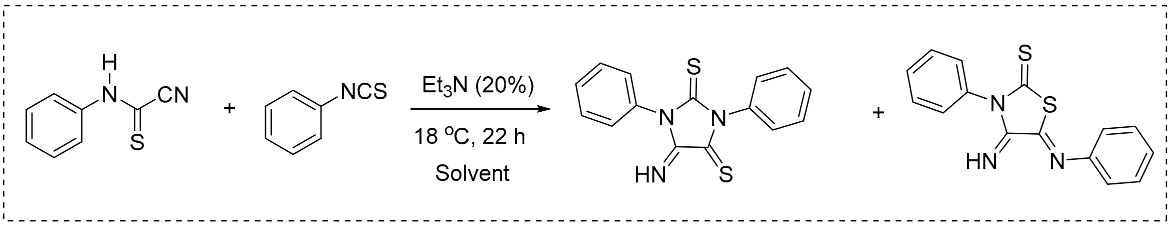

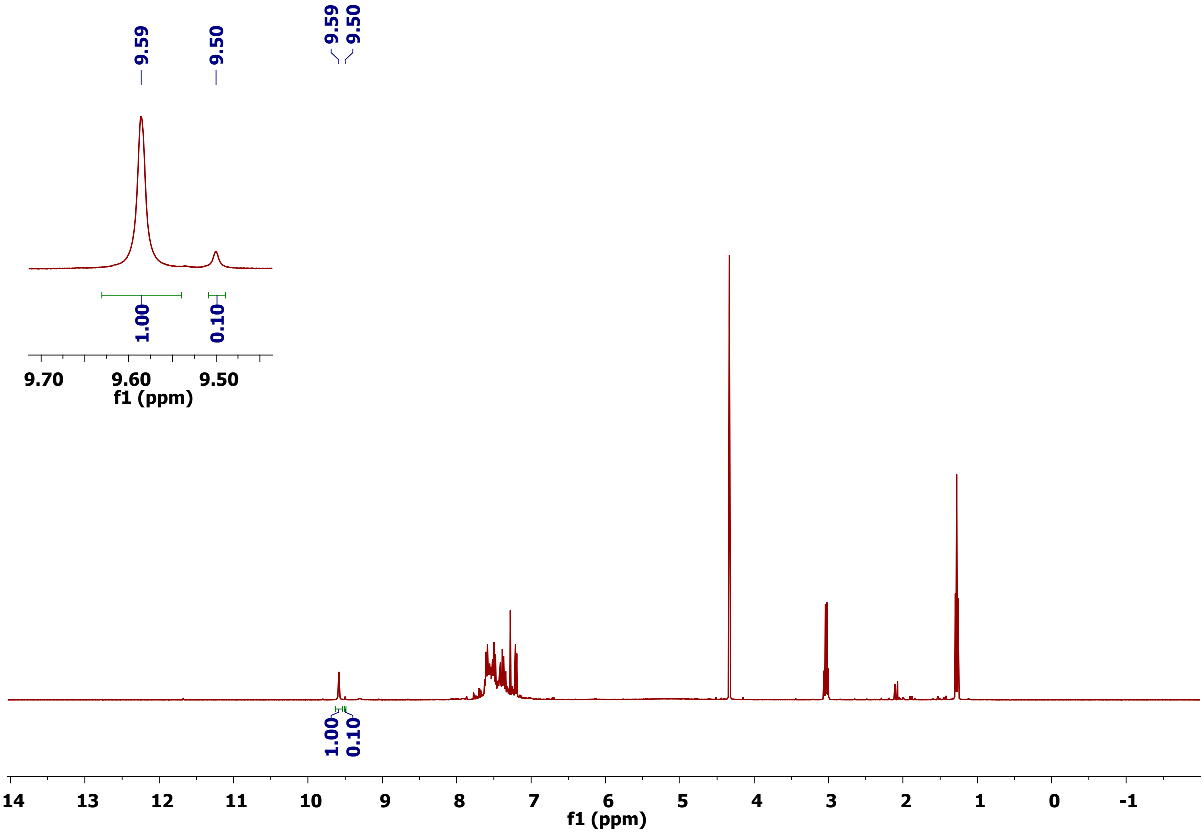

Ratio of 5-imino-1,3-diphenylimidazolidine-2,4-dithione and (Z)-4-imino-3-phenyl-5-(phenylimino)thiazolidine-2-thione in dioxane as solvent

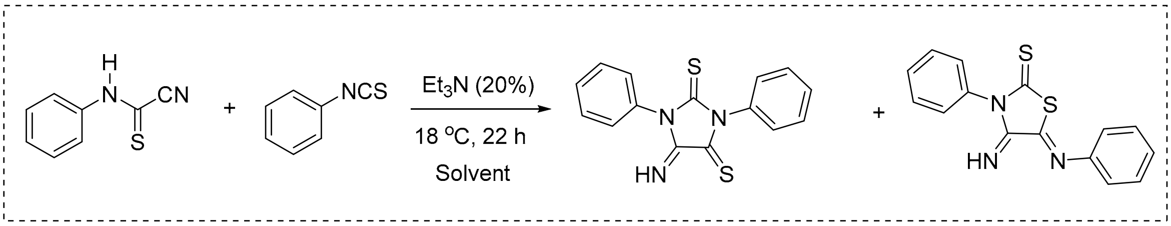

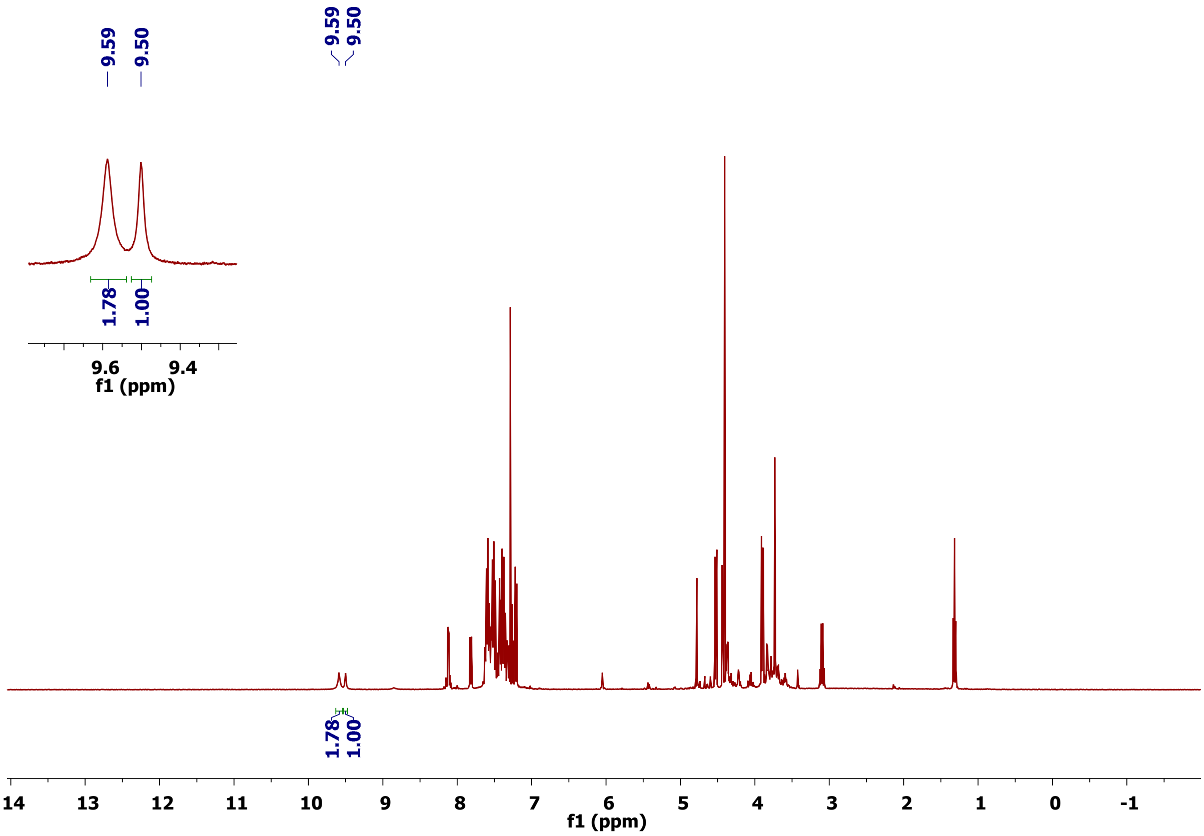

Ratio of 5-imino-1,3-diphenylimidazolidine-2,4-dithione and (Z)-4-imino-3-phenyl-5-(phenylimino)thiazolidine-2-thione in benzene as solvent

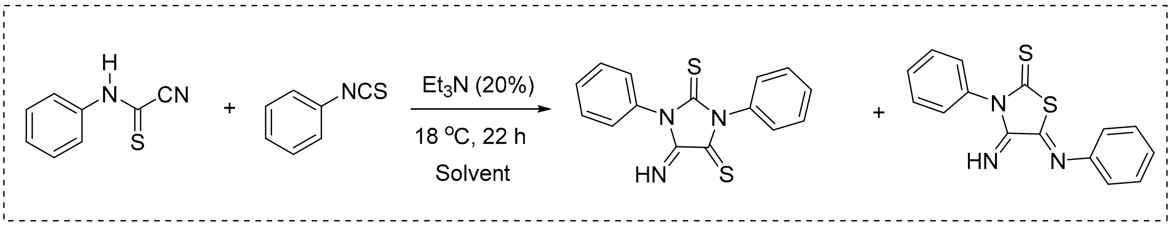

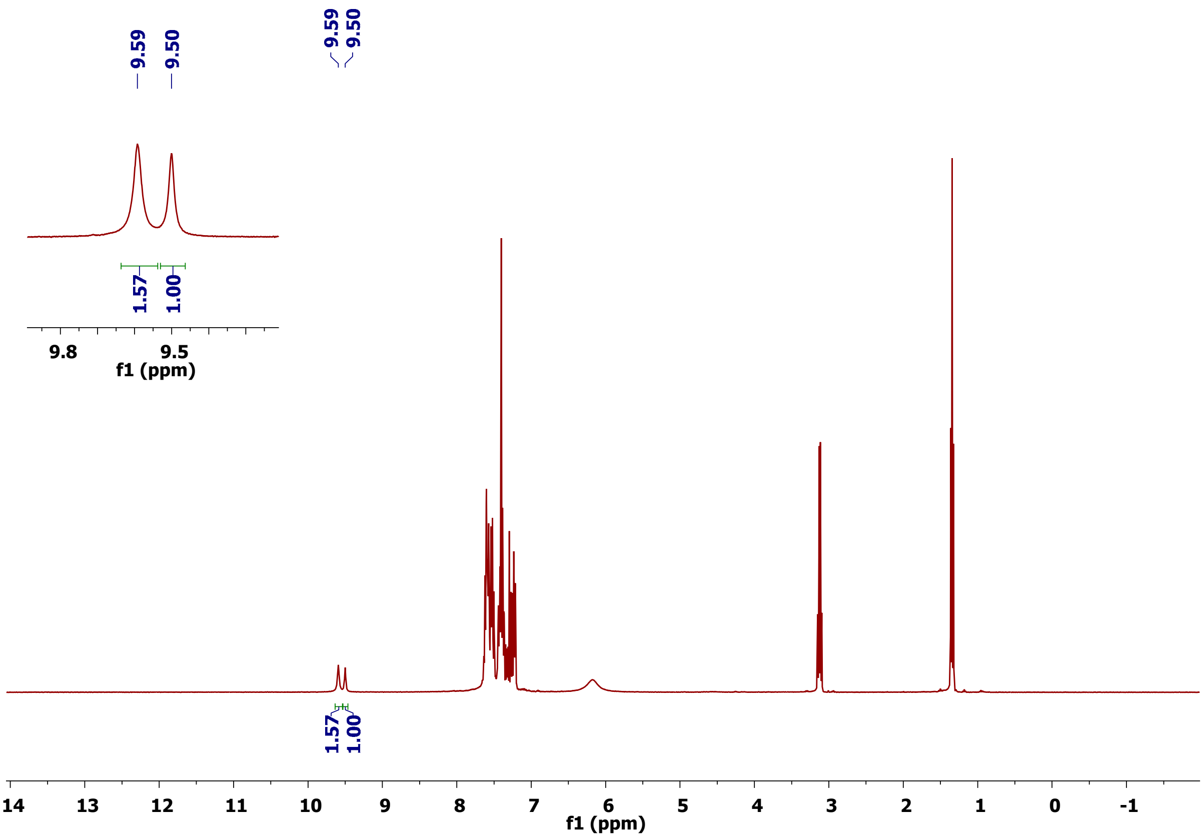

Ratio of 5-imino-1,3-diphenylimidazolidine-2,4-dithione and (Z)-4-imino-3-phenyl-5-(phenylimino)thiazolidine-2-thione in toluene as solvent

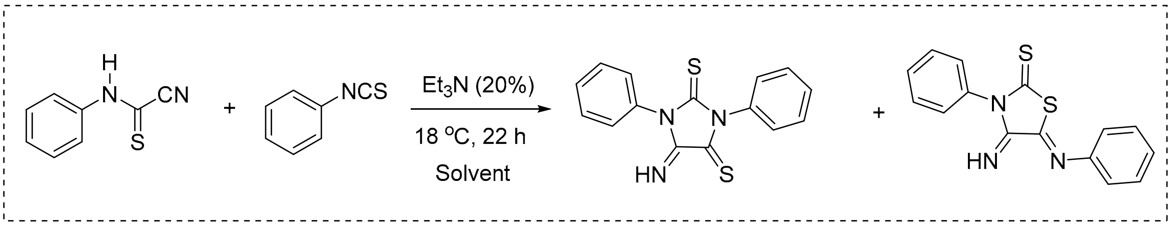

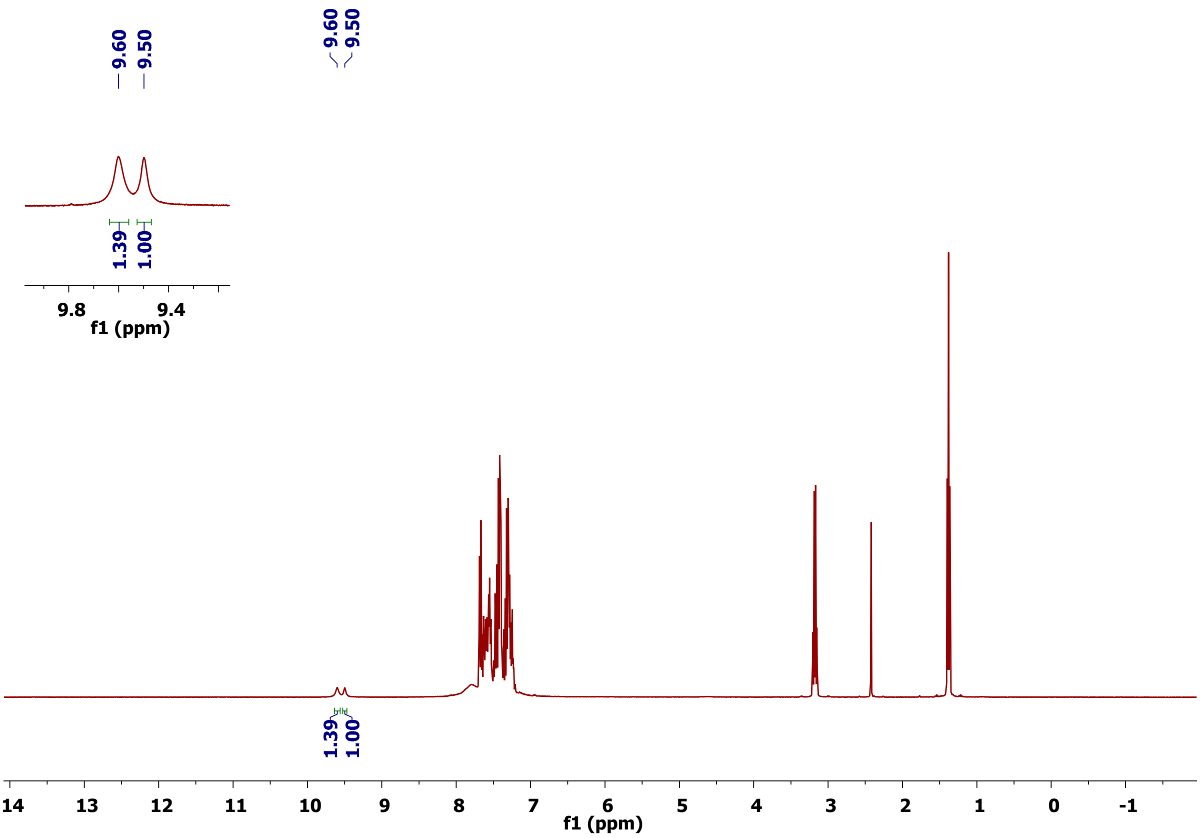

Ratio of 5-imino-1,3-diphenylimidazolidine-2,4-dithione and (Z)-4-imino-3-phenyl-5-(phenylimino)thiazolidine-2-thione in THF as solvent

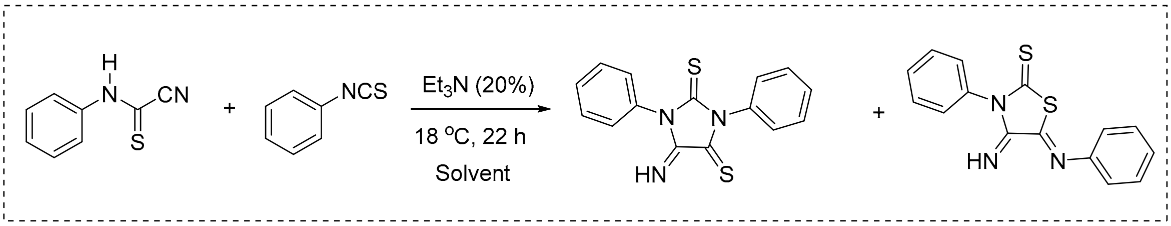

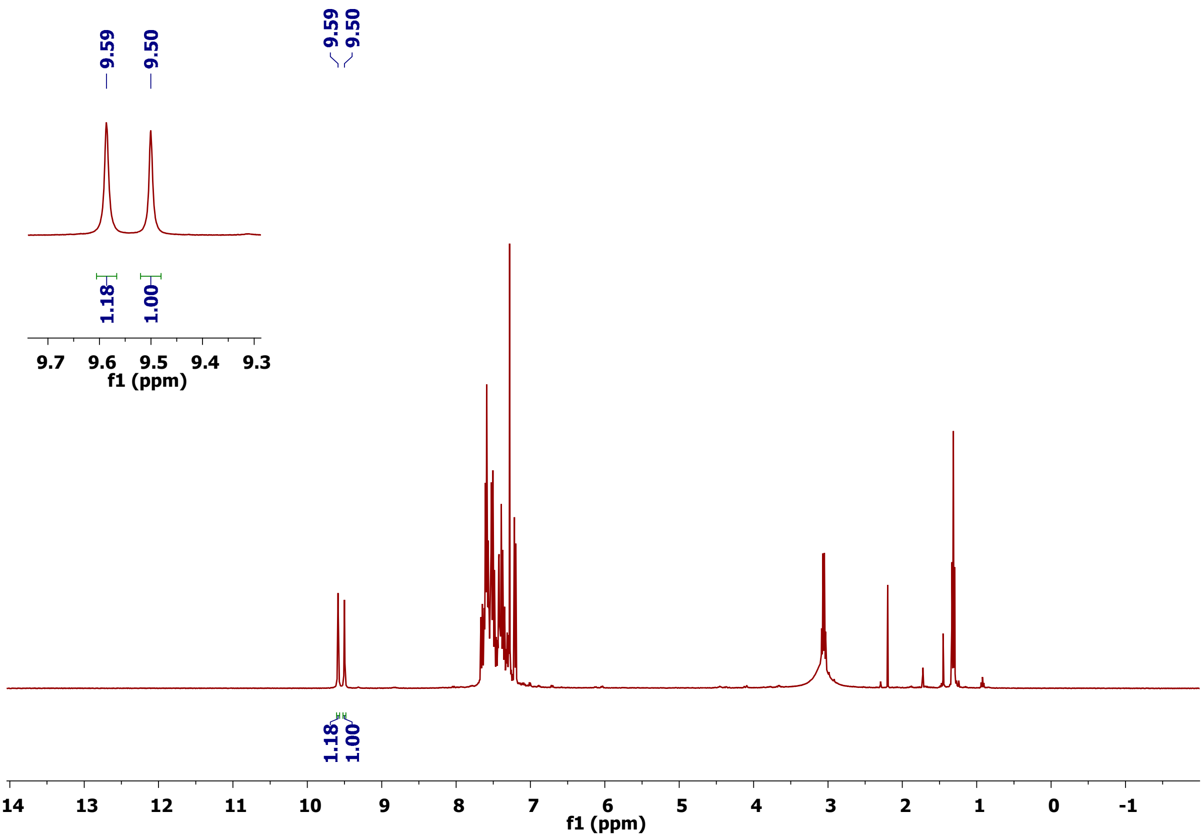

Ratio of 5-imino-1,3-diphenylimidazolidine-2,4-dithione and (Z)-4-imino-3-phenyl-5-(phenylimino)thiazolidine-2-thione in ethyl acetate as solvent

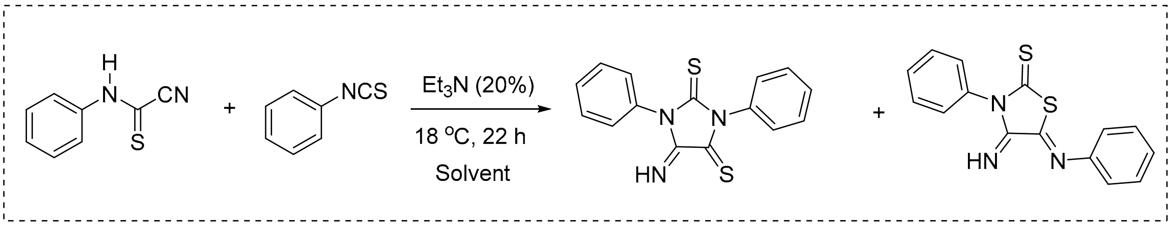

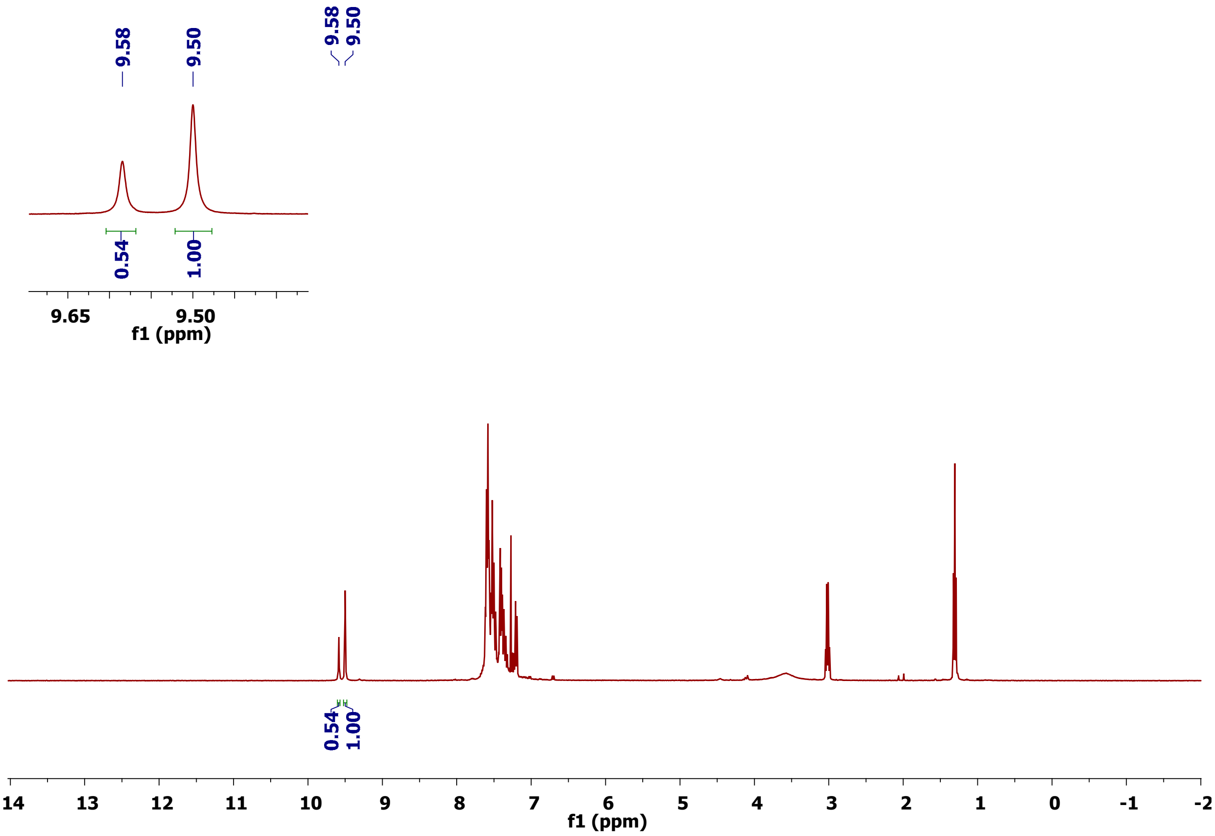

Ratio of 5-imino-1,3-diphenylimidazolidine-2,4-dithione and (Z)-4-imino-3-phenyl-5-(phenylimino)thiazolidine-2-thione in diethyl ether as solvent

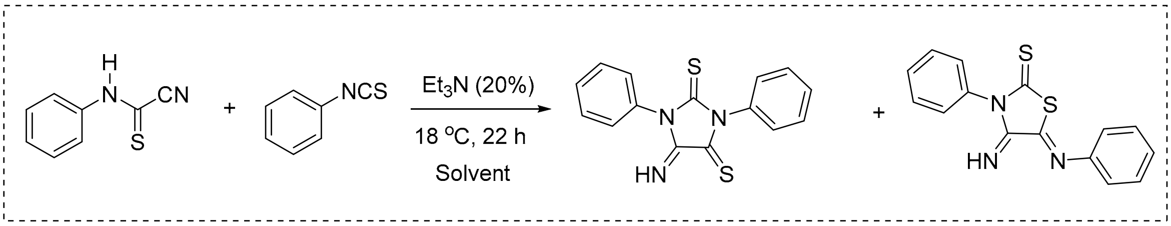

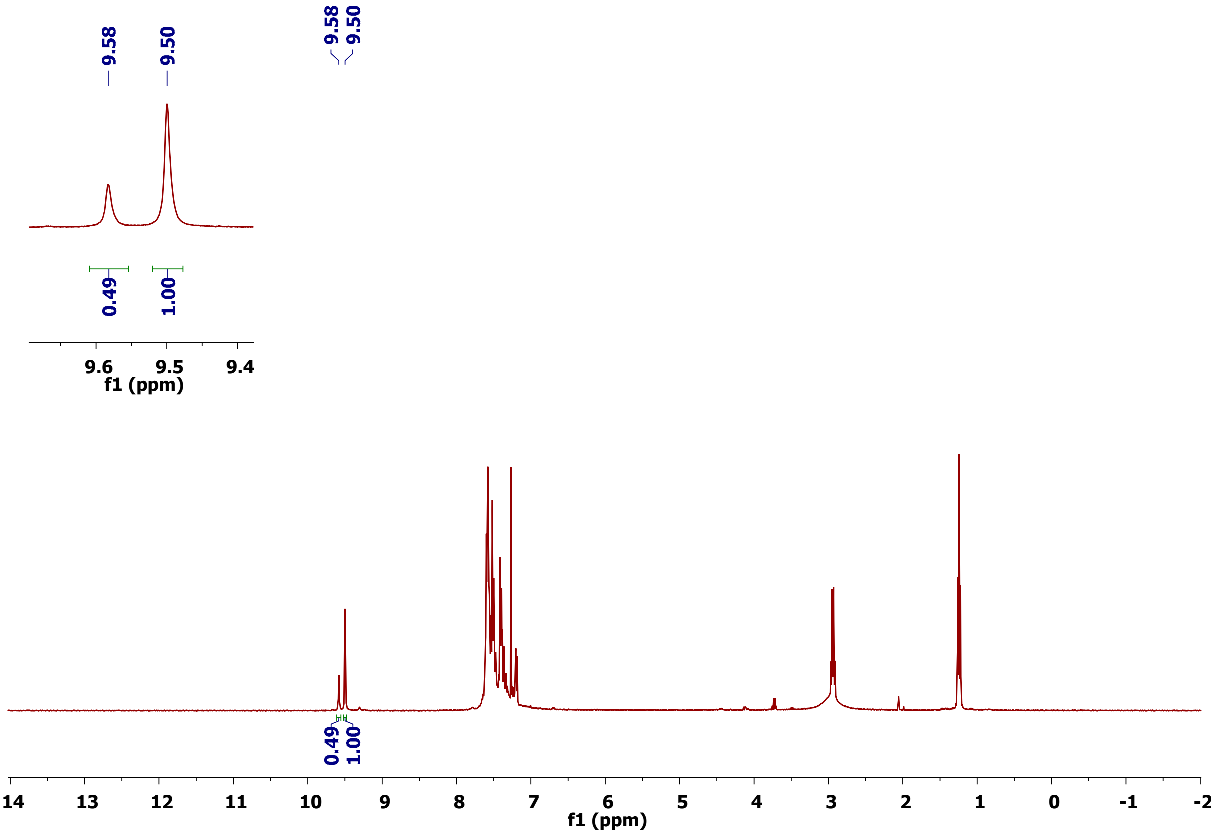

Ratio of 5-imino-1,3-diphenylimidazolidine-2,4-dithione and (Z)-4-imino-3-phenyl-5-(phenylimino)thiazolidine-2-thione in chloroform as solvent

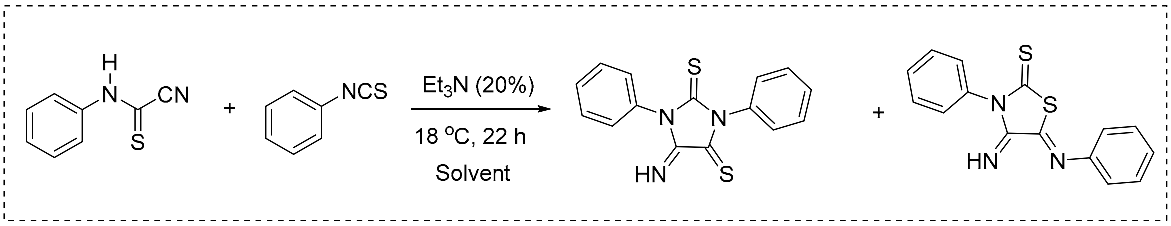

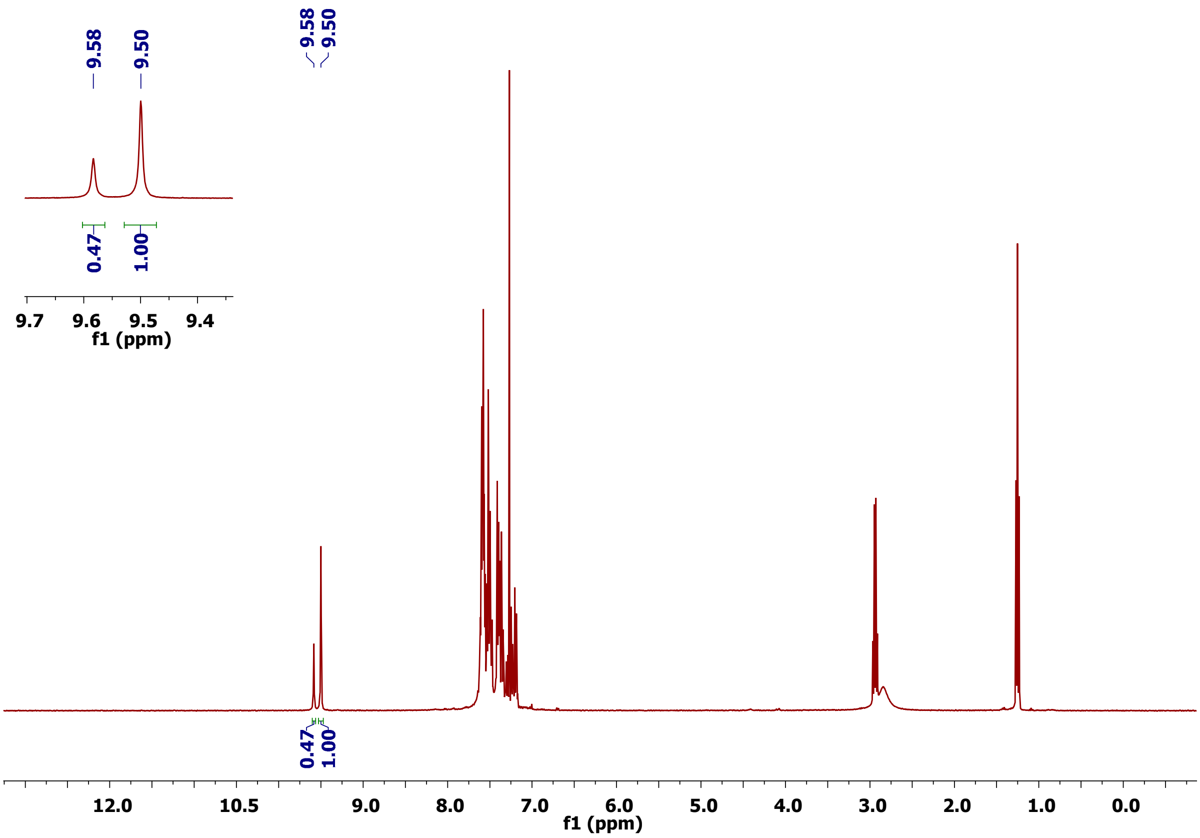

Ratio of 5-imino-1,3-diphenylimidazolidine-2,4-dithione and (Z)-4-imino-3-phenyl-5-(phenylimino)thiazolidine-2-thione in methanol as solvent

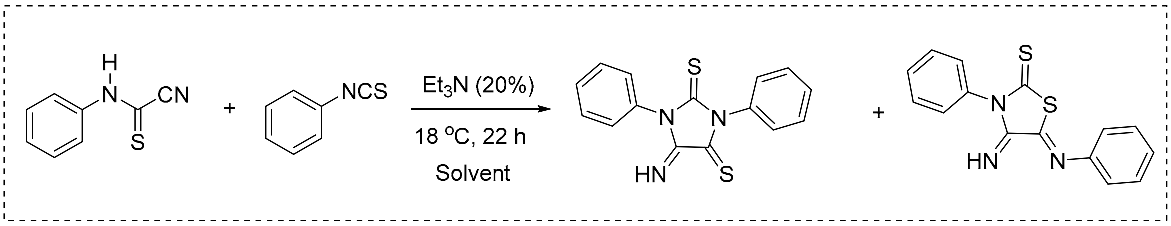

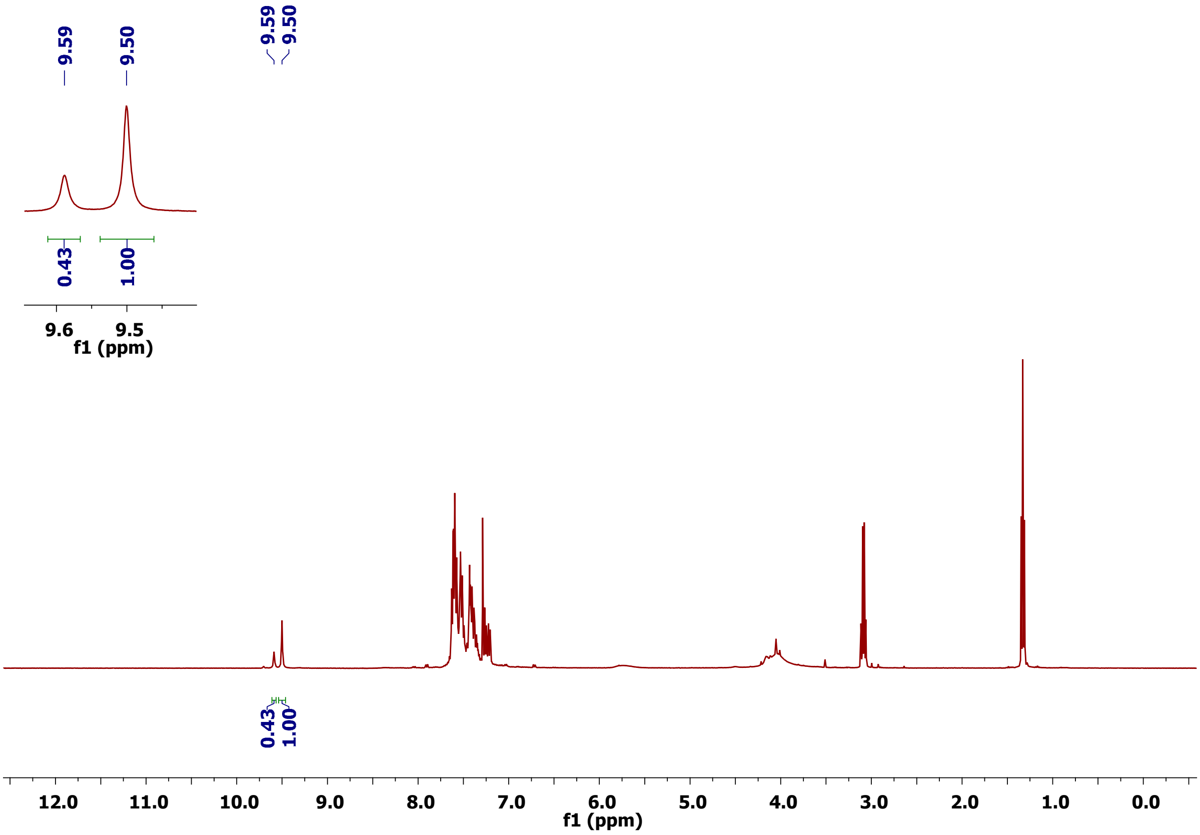

Ratio of 5-imino-1,3-diphenylimidazolidine-2,4-dithione and (Z)-4-imino-3-phenyl-5-(phenylimino)thiazolidine-2-thione in ethanol as solvent

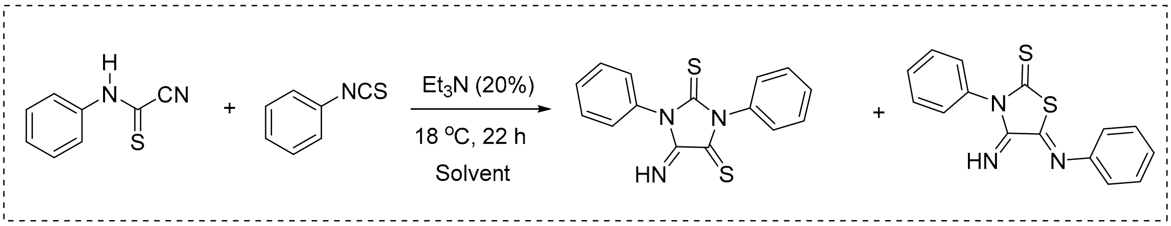

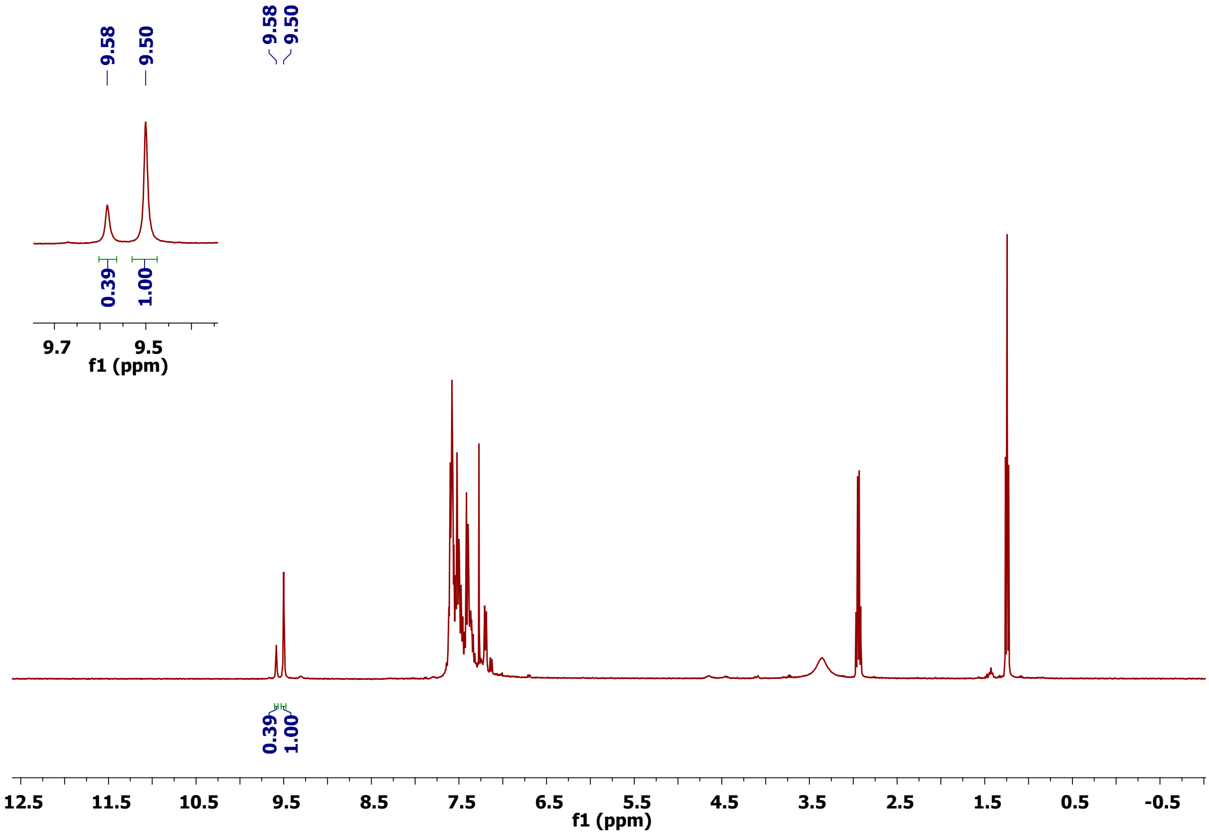

Ratio of 5-imino-1,3-diphenylimidazolidine-2,4-dithione and (Z)-4-imino-3-phenyl-5-(phenylimino)thiazolidine-2-thione in dichloromethane as solvent

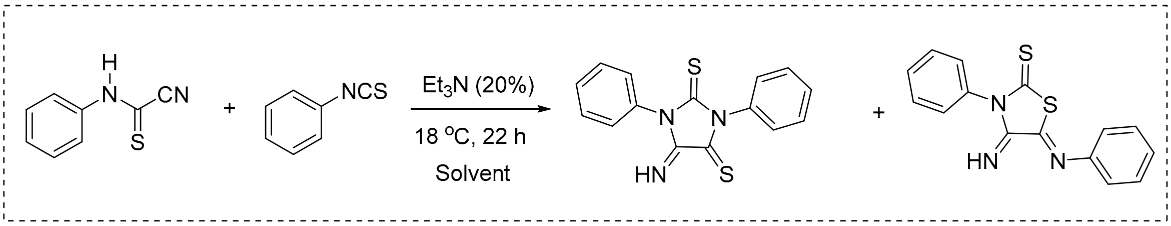

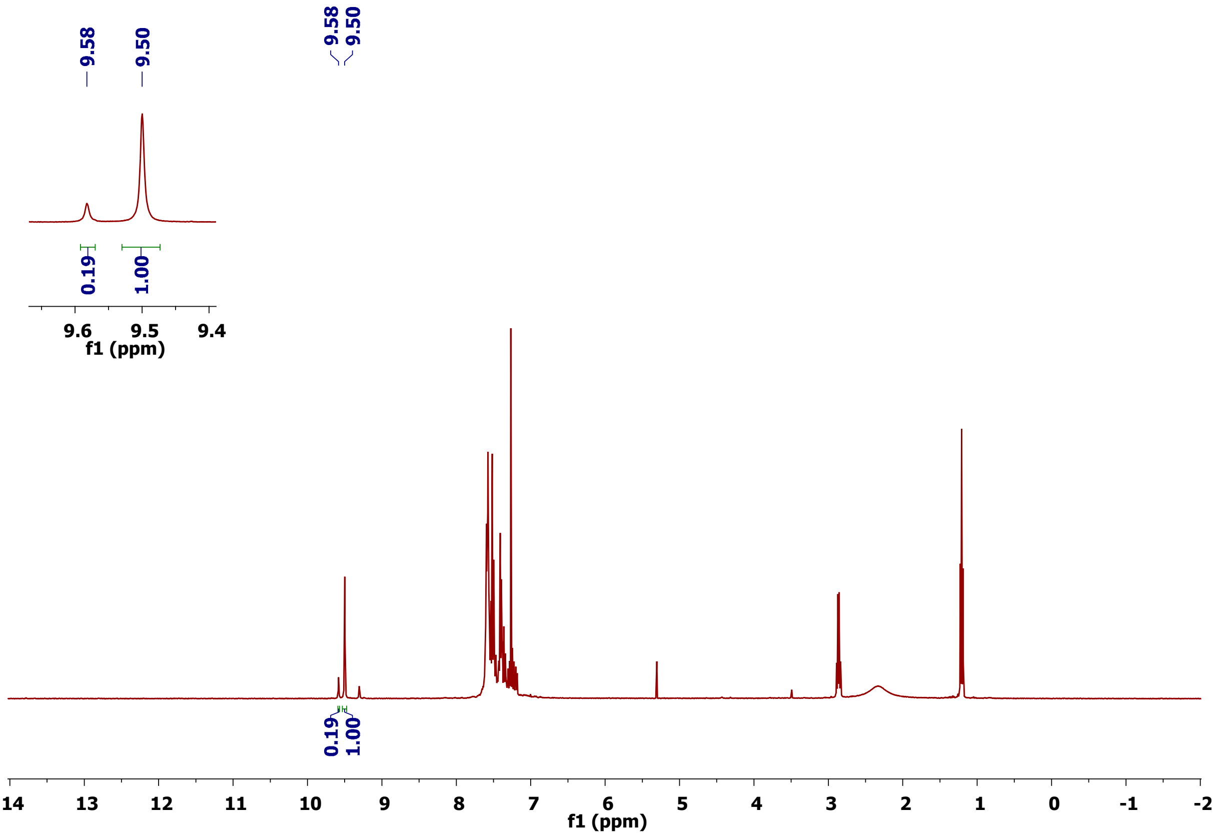

Ratio of 5-imino-1,3-diphenylimidazolidine-2,4-dithione and (Z)-4-imino-3-phenyl-5-(phenylimino)thiazolidine-2-thione in acetonitrile as solvent

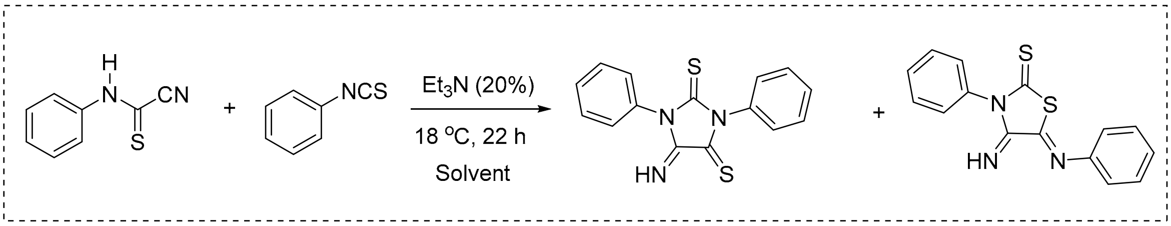

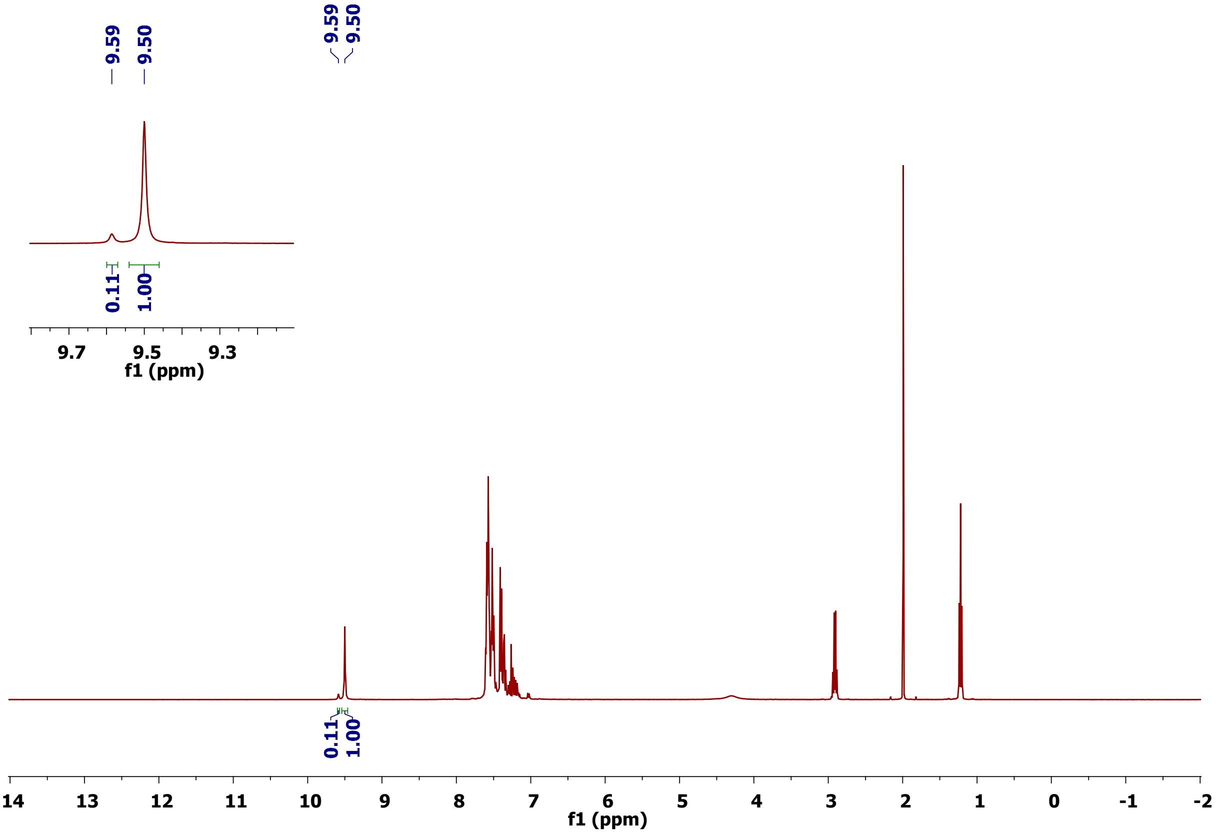

Ratio of 5-imino-1,3-diphenylimidazolidine-2,4-dithione and (Z)-4-imino-3-phenyl-5-(phenylimino)thiazolidine-2-thione in acetone as solvent

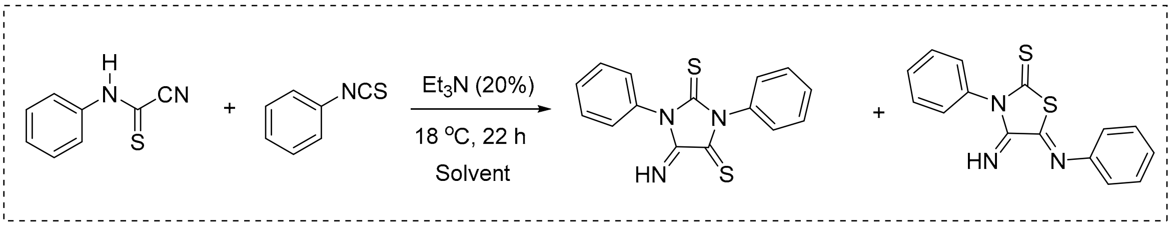

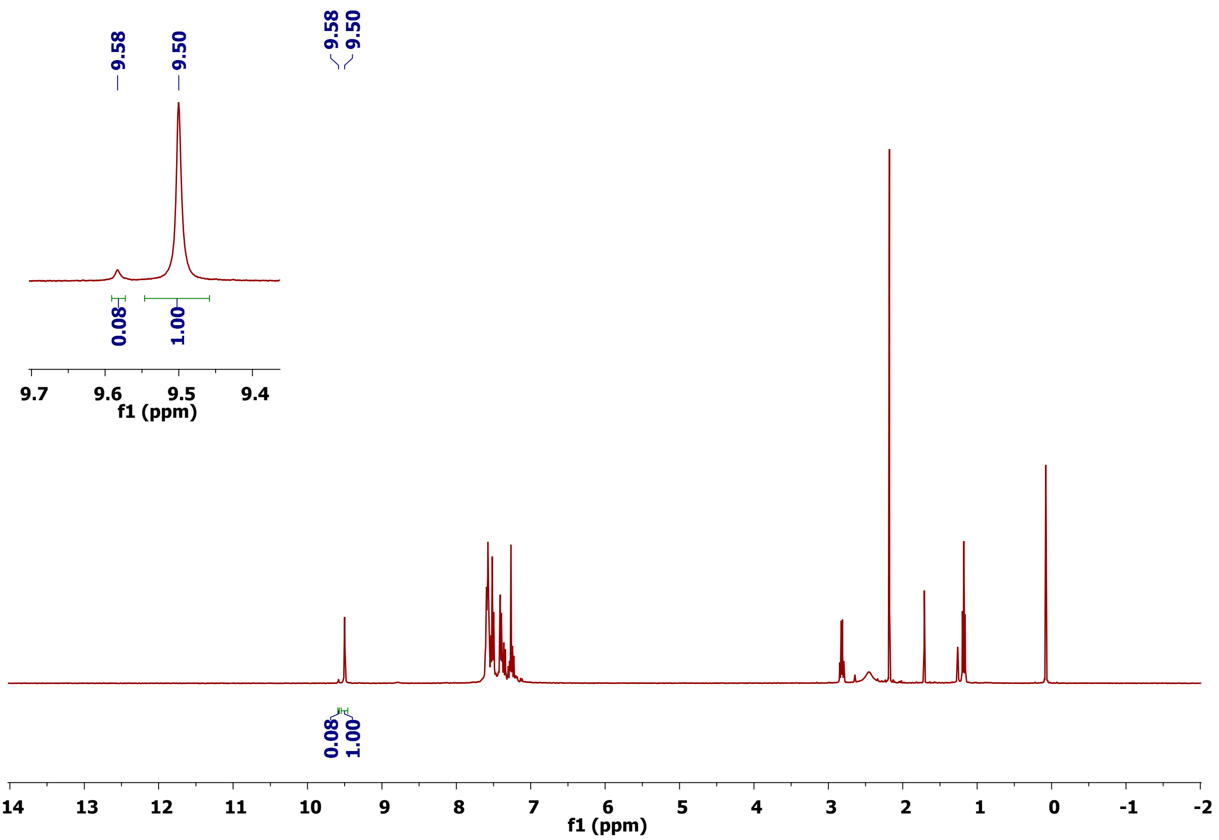

Ratio of 5-imino-1,3-diphenylimidazolidine-2,4-dithione and (Z)-4-imino-3-phenyl-5-(phenylimino)thiazolidine-2-thione in DMSO as solvent

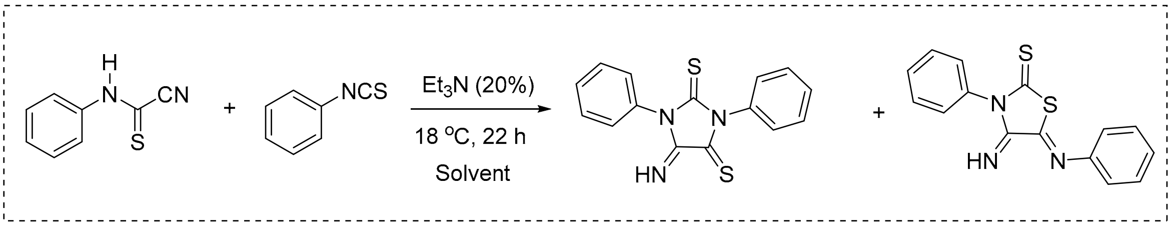

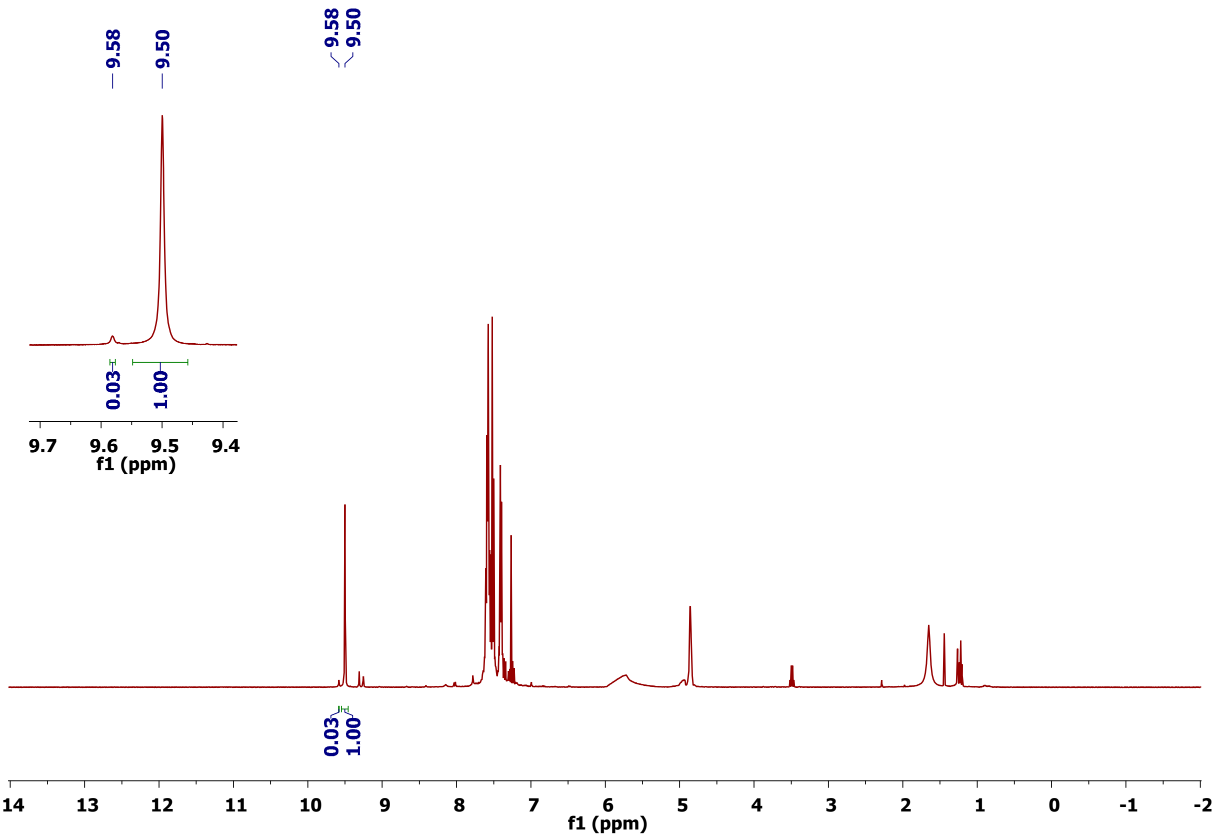

Ratio of 5-imino-1,3-diphenylimidazolidine-2,4-dithione and (Z)-4-imino-3-phenyl-5-(phenylimino)thiazolidine-2-thione in DMF as solvent

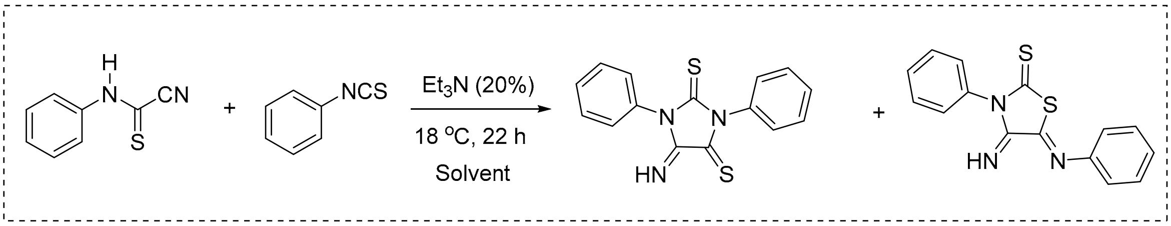

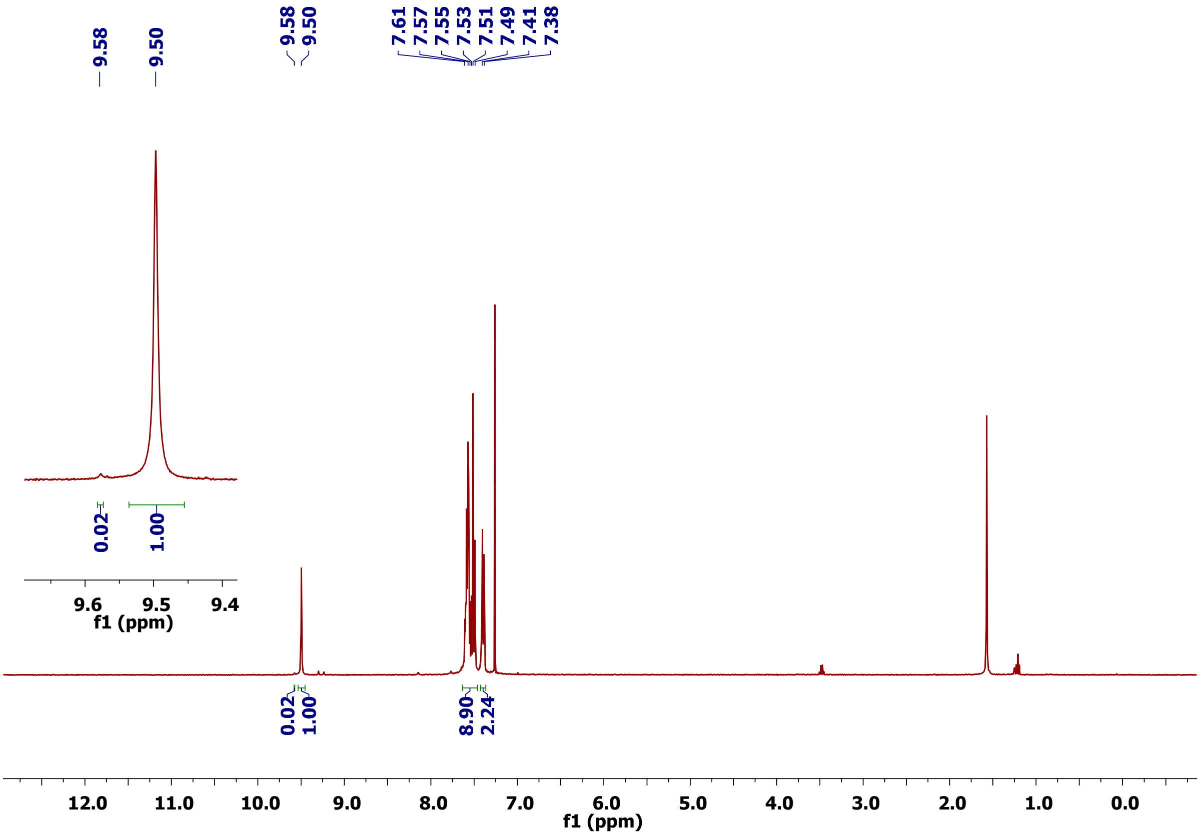

Ratio of 1-(2-fluorophenyl)-5-imino-3-phenylimidazolidine-2,4-dithione and (Z)-5-((2-fluorophenyl)imino)-4-imino-3-phenylthiazolidine-2-thione in nitromethane as solvent

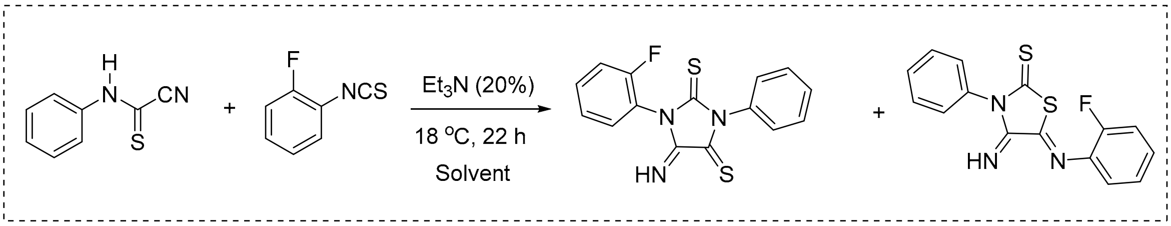

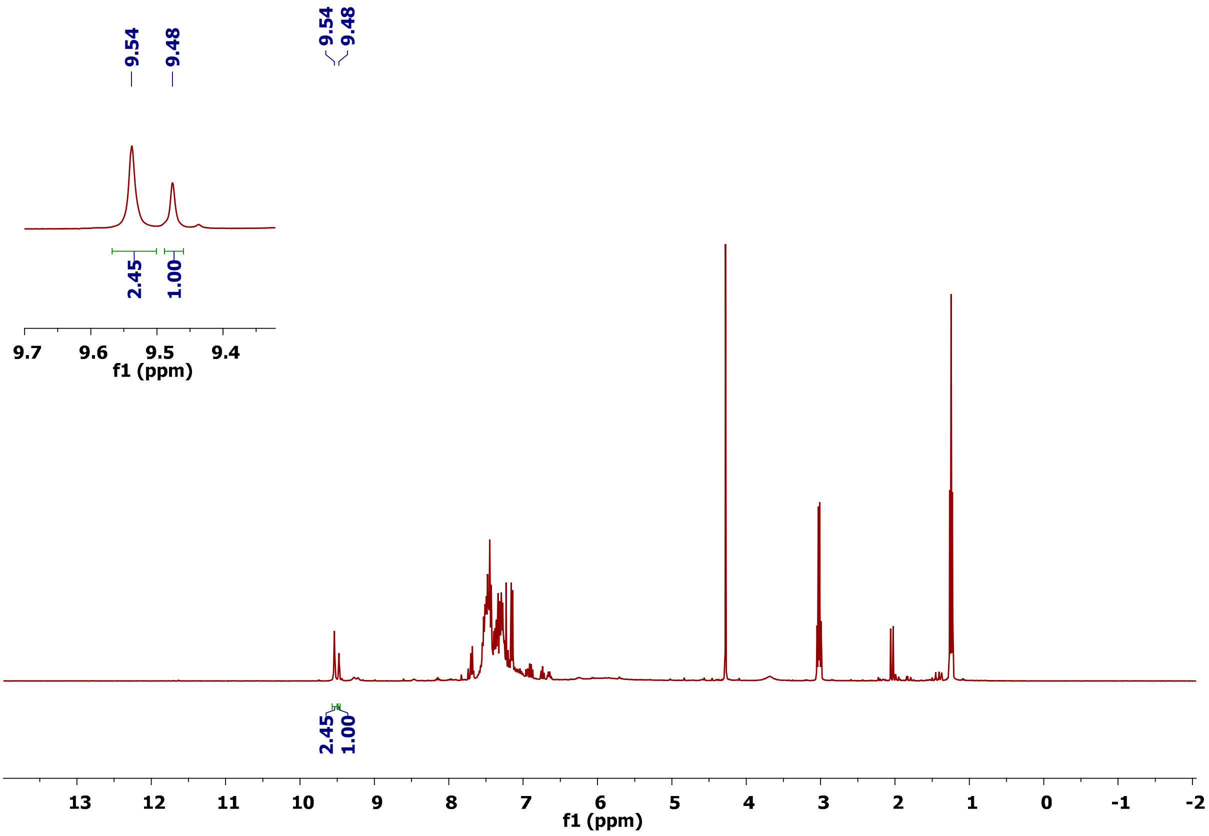

Ratio of 1-(2-fluorophenyl)-5-imino-3-phenylimidazolidine-2,4-dithione and (Z)-5-((2-fluorophenyl)imino)-4-imino-3-phenylthiazolidine-2-thione in ethyl acetate as solvent

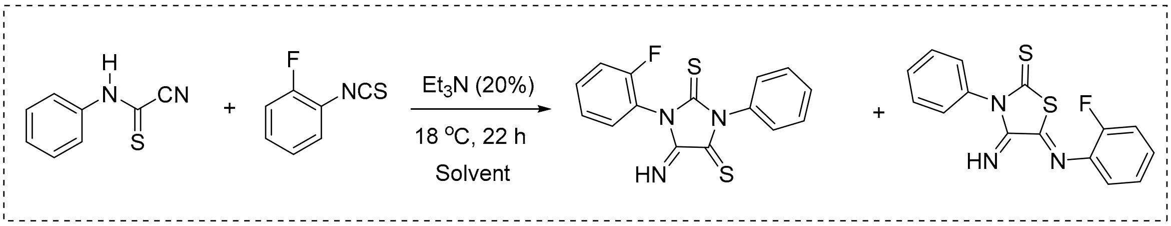

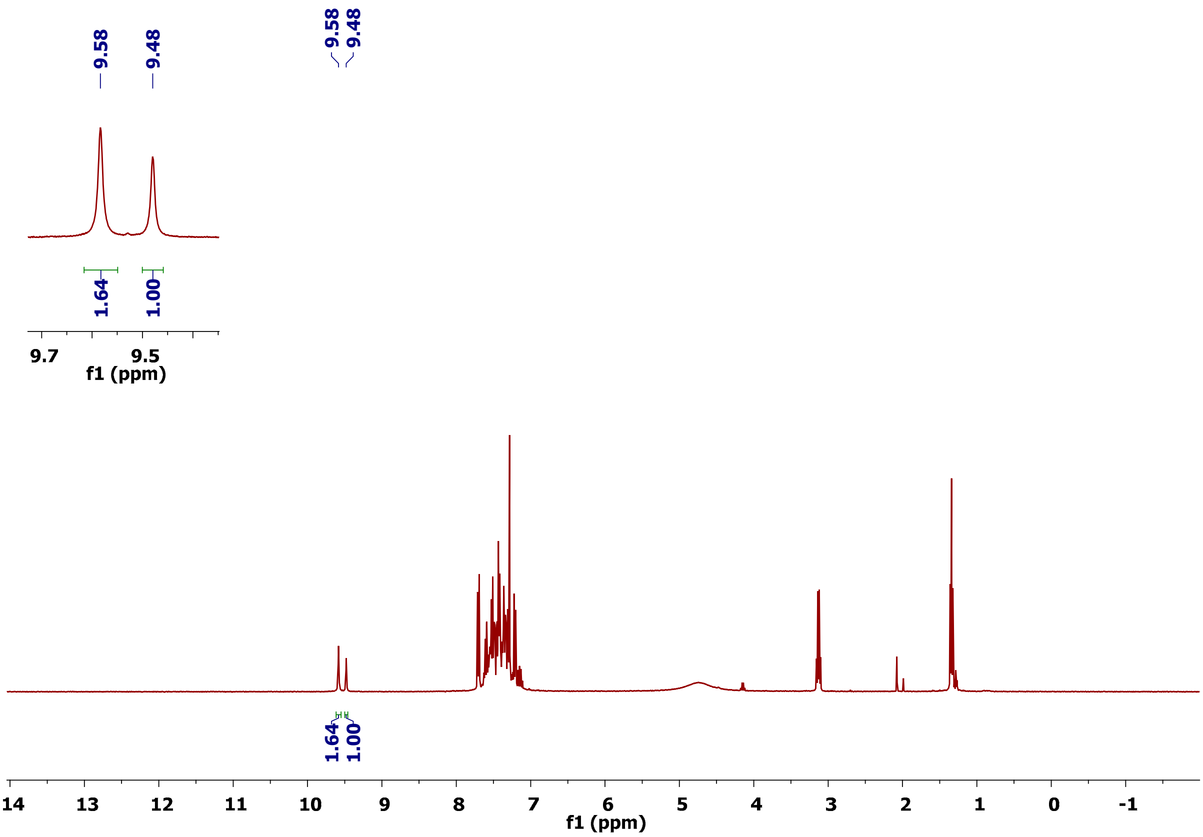

Ratio of 1-(2-fluorophenyl)-5-imino-3-phenylimidazolidine-2,4-dithione and (Z)-5-((2-fluorophenyl)imino)-4-imino-3-phenylthiazolidine-2-thione in THF as solvent

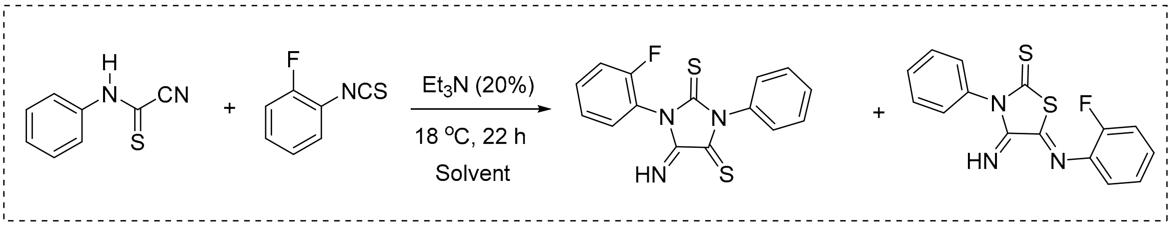

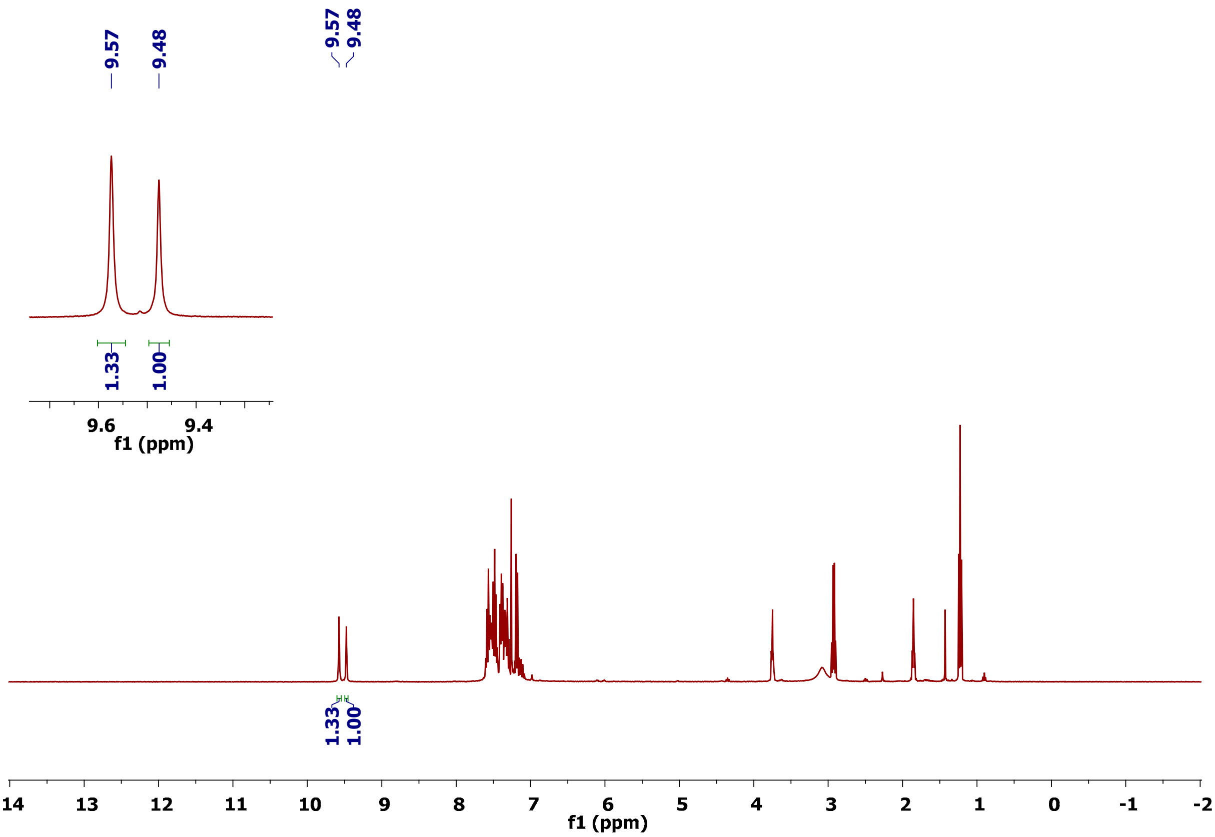

Ratio of 1-(2-fluorophenyl)-5-imino-3-phenylimidazolidine-2,4-dithione and (Z)-5-((2-fluorophenyl)imino)-4-imino-3-phenylthiazolidine-2-thione in methanol as solvent

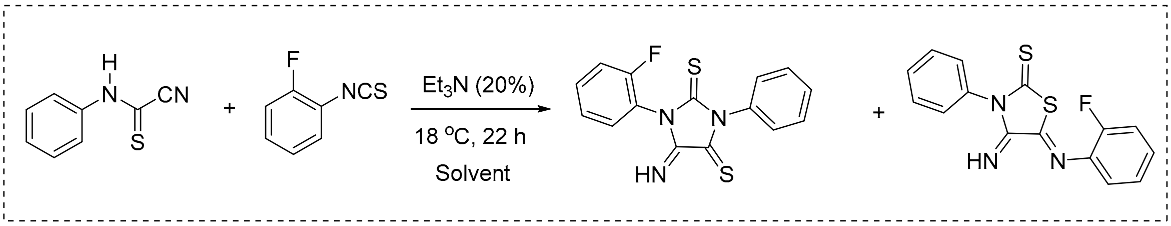

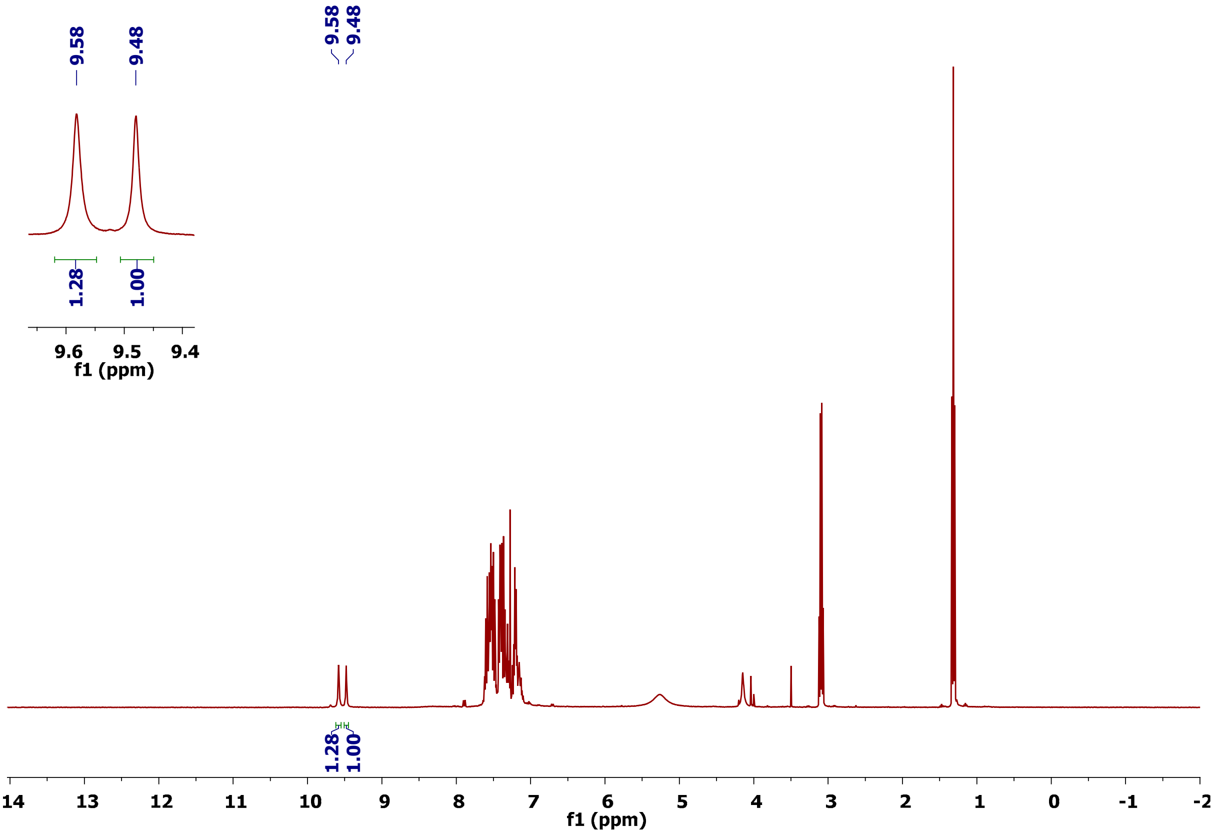

Ratio of 1-(2-fluorophenyl)-5-imino-3-phenylimidazolidine-2,4-dithione and (Z)-5-((2-fluorophenyl)imino)-4-imino-3-phenylthiazolidine-2-thione in diethyl ether as solvent

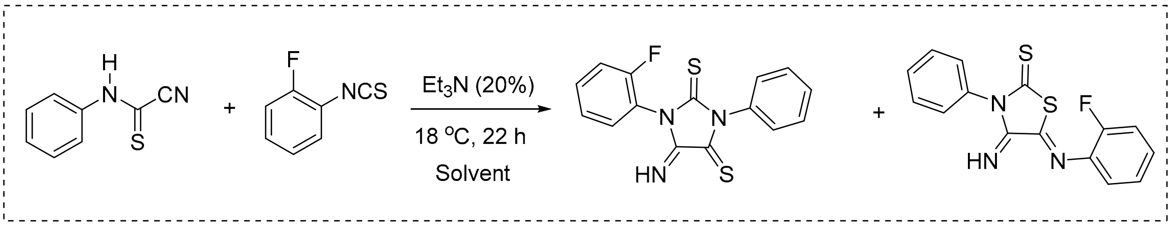

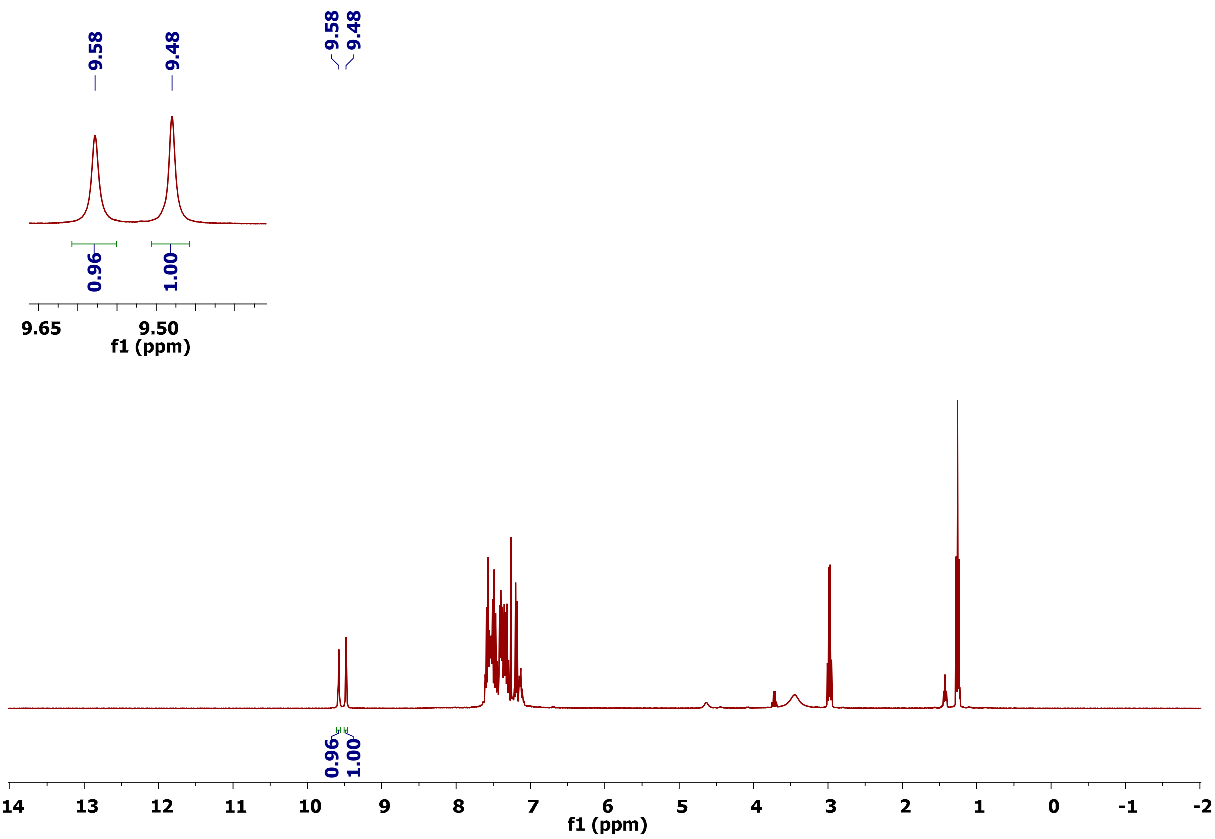

Ratio of 1-(2-fluorophenyl)-5-imino-3-phenylimidazolidine-2,4-dithione and (Z)-5-((2-fluorophenyl)imino)-4-imino-3-phenylthiazolidine-2-thione in ethanol as solvent

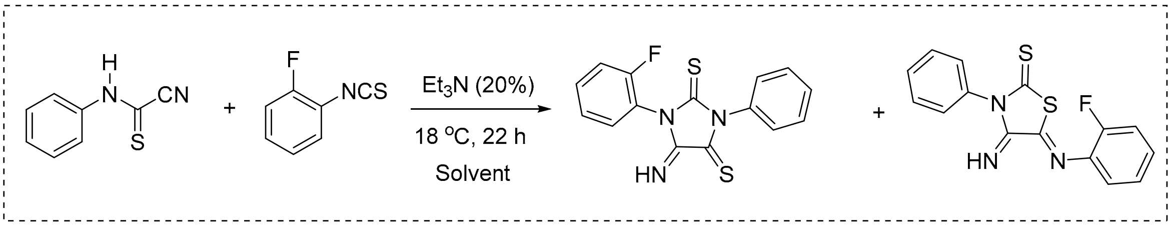

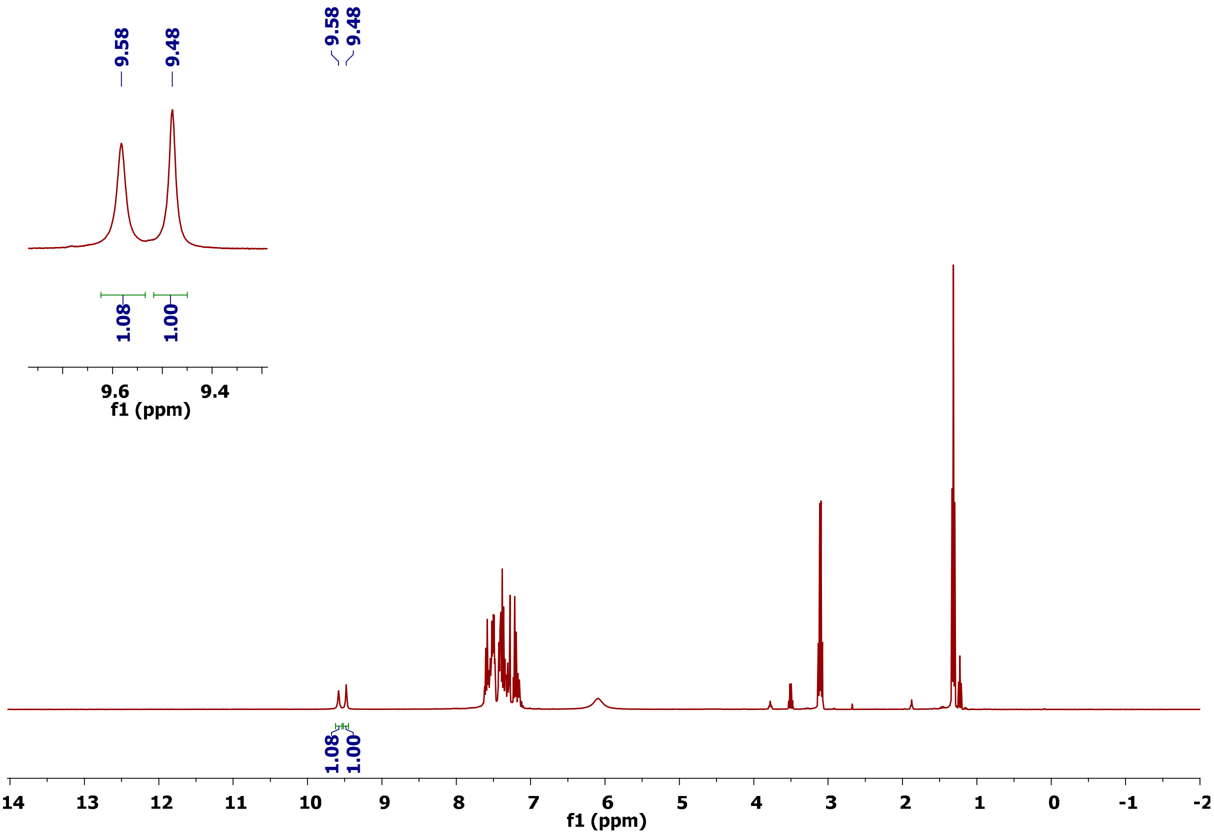

Ratio of 1-(2-fluorophenyl)-5-imino-3-phenylimidazolidine-2,4-dithione and (Z)-5-((2-fluorophenyl)imino)-4-imino-3-phenylthiazolidine-2-thione in toluene as solvent

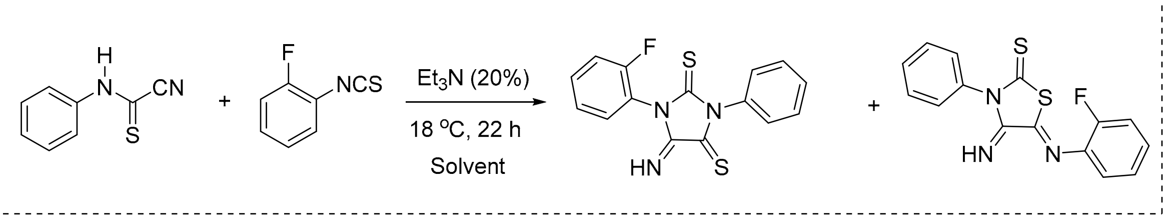

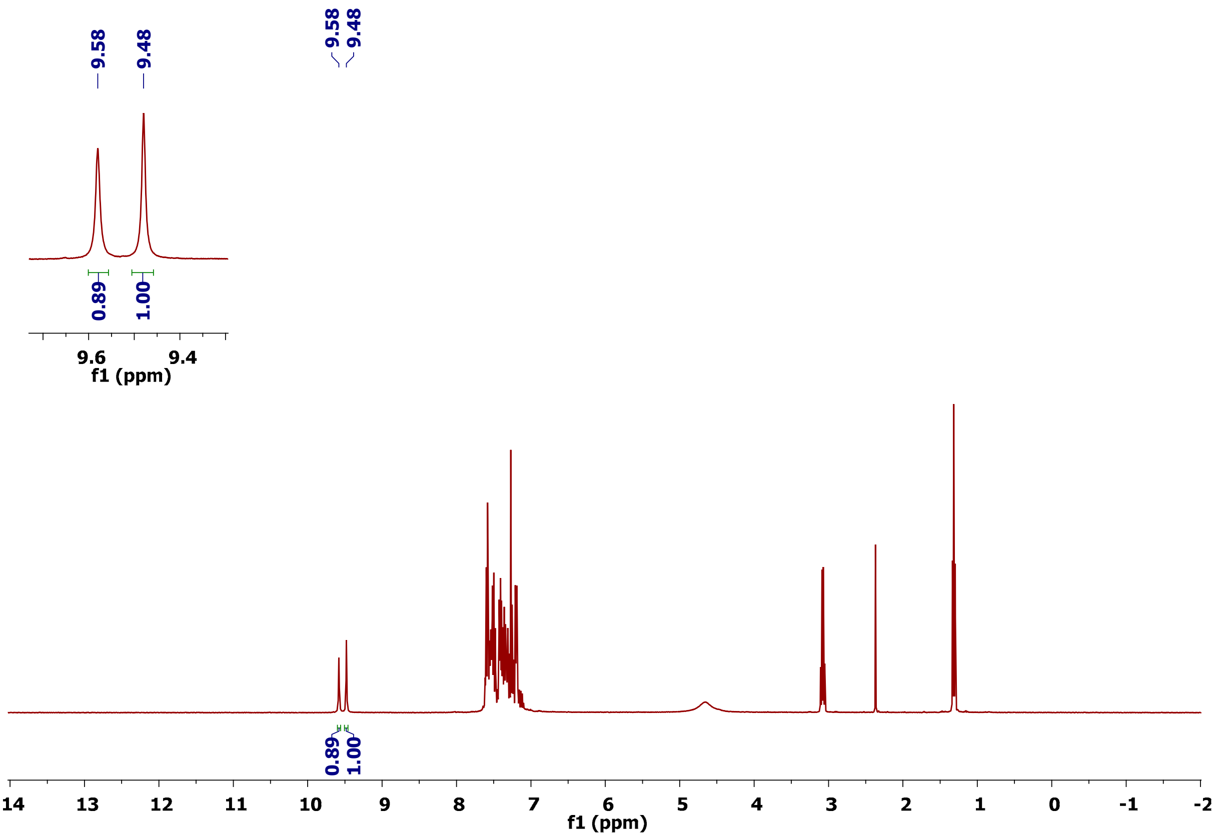

Ratio of 1-(2-fluorophenyl)-5-imino-3-phenylimidazolidine-2,4-dithione and (Z)-5-((2-fluorophenyl)imino)-4-imino-3-phenylthiazolidine-2-thione in dioxane as solvent

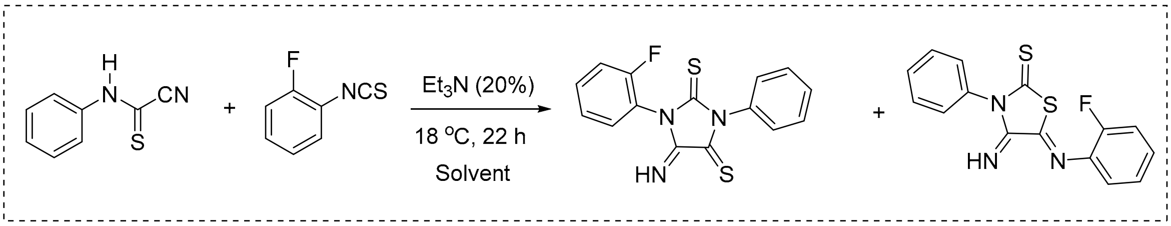

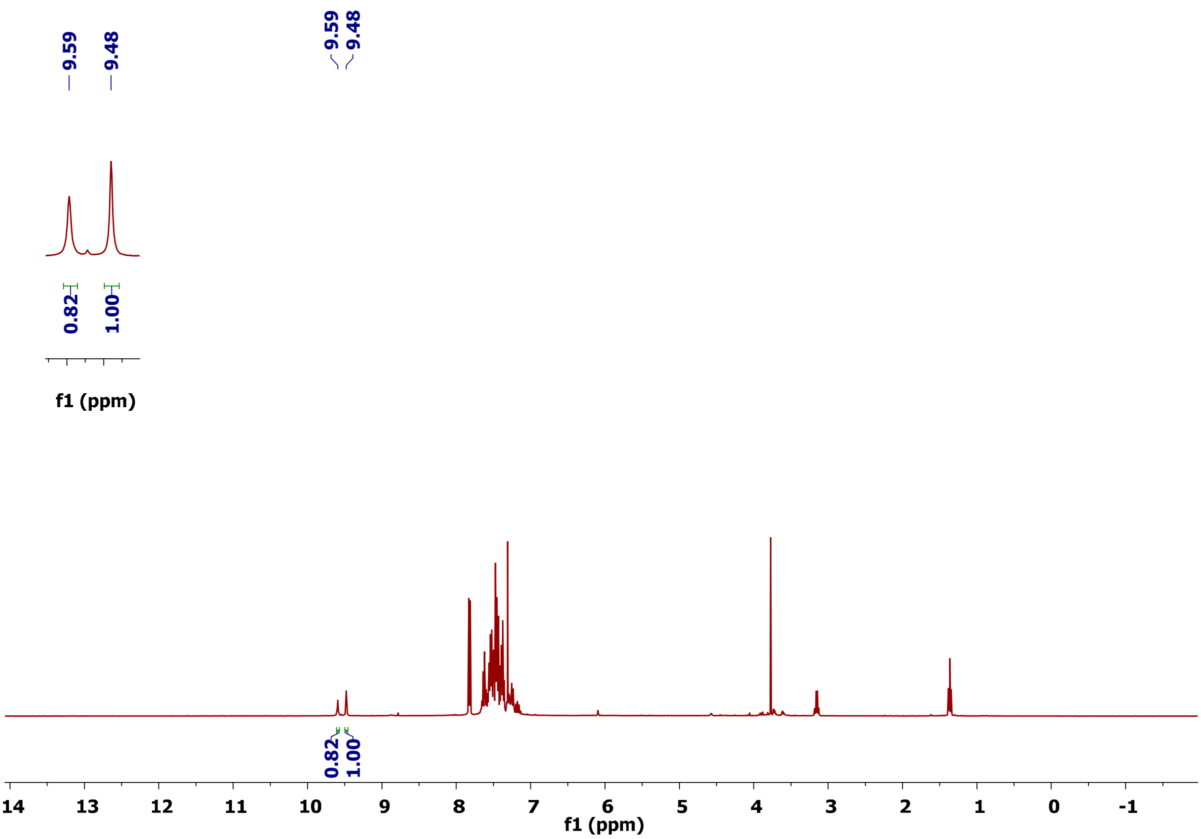

Ratio of 1-(2-fluorophenyl)-5-imino-3-phenylimidazolidine-2,4-dithione and (Z)-5-((2-fluorophenyl)imino)-4-imino-3-phenylthiazolidine-2-thione in benzene as solvent

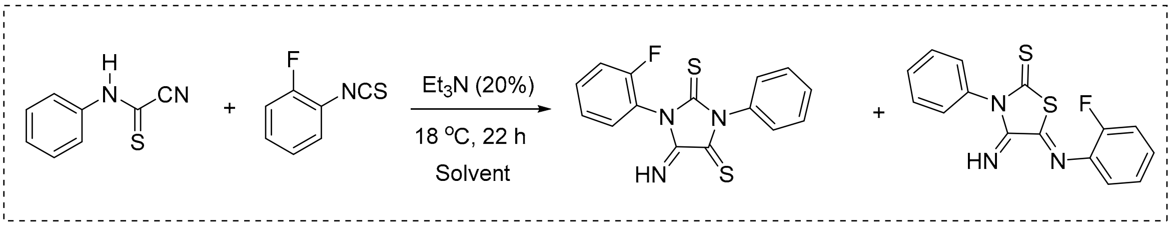

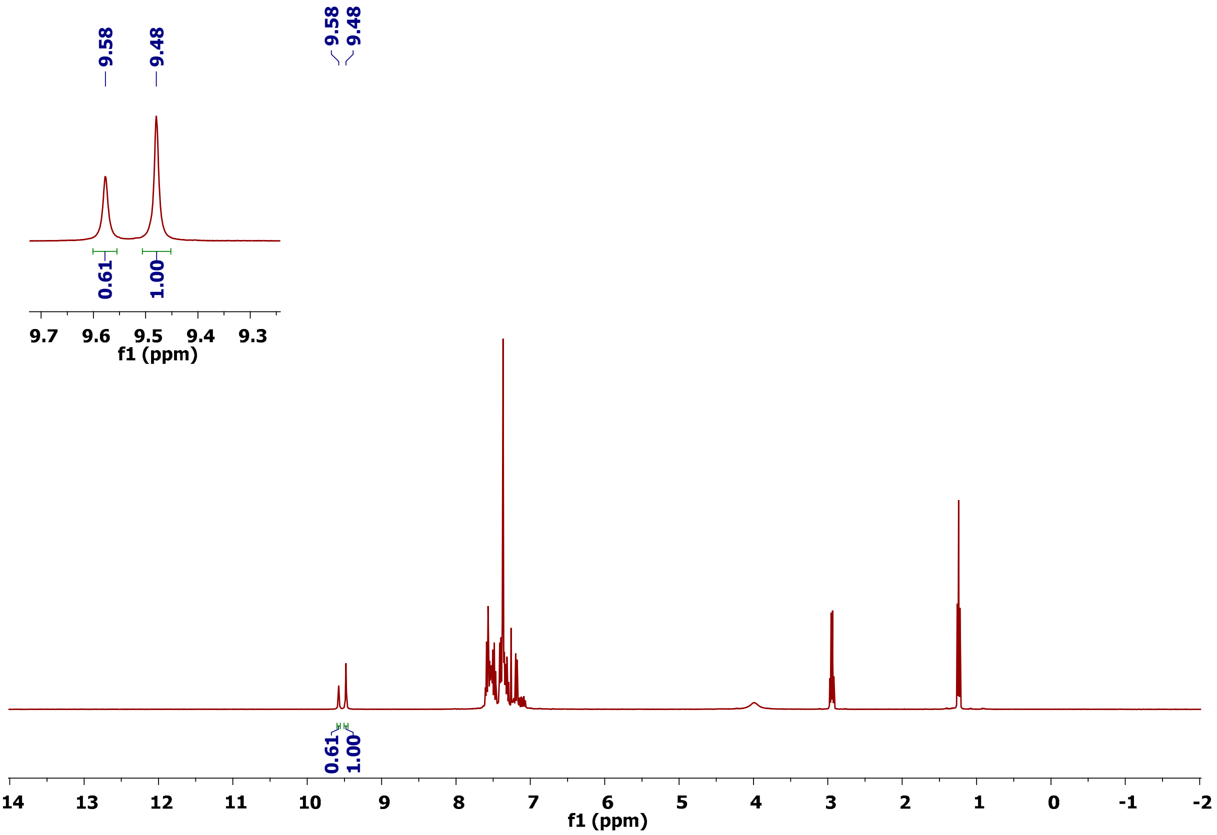

Ratio of 1-(2-fluorophenyl)-5-imino-3-phenylimidazolidine-2,4-dithione and (Z)-5-((2-fluorophenyl)imino)-4-imino-3-phenylthiazolidine-2-thione in DCM as solvent

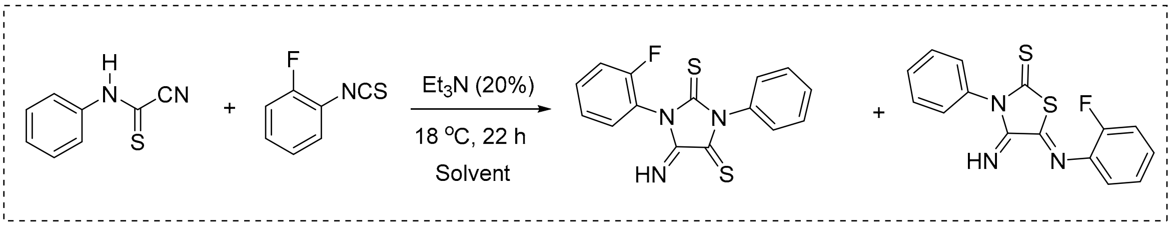

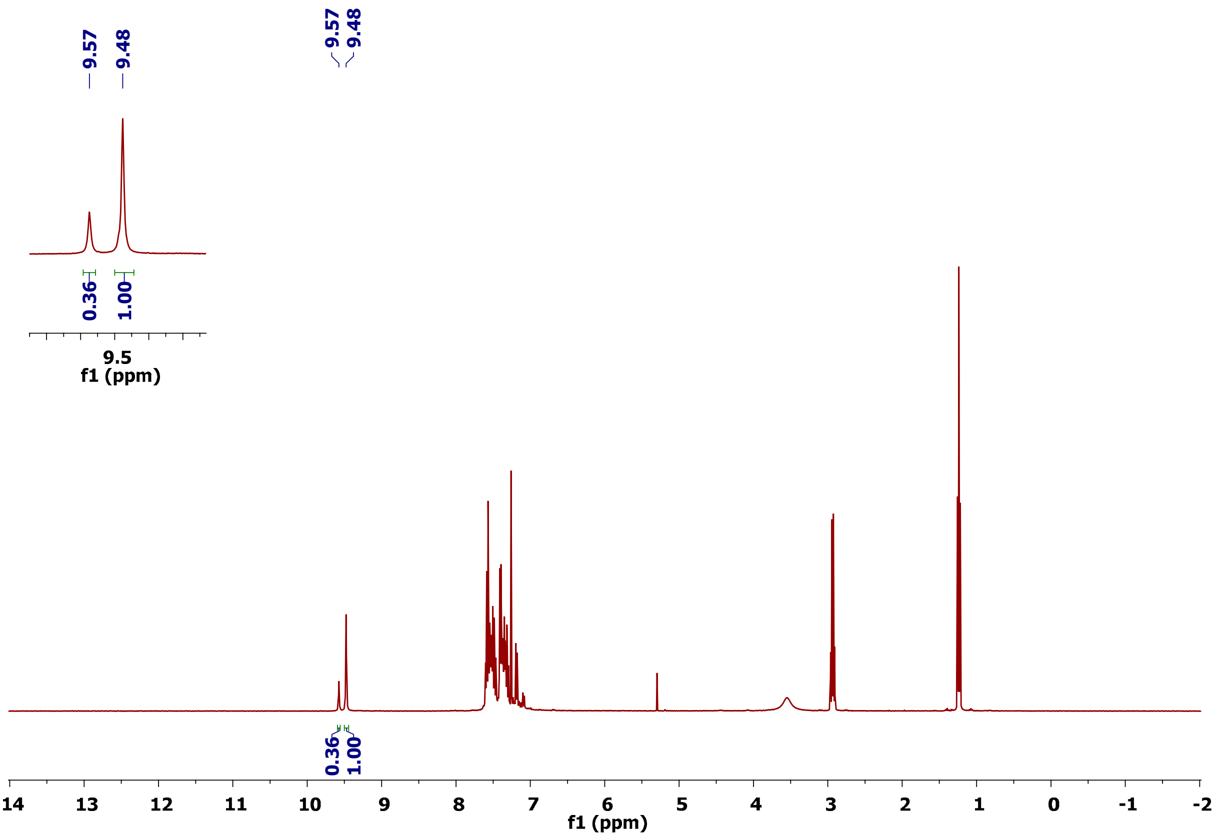

Ratio of 1-(2-fluorophenyl)-5-imino-3-phenylimidazolidine-2,4-dithione and (Z)-5-((2-fluorophenyl)imino)-4-imino-3-phenylthiazolidine-2-thione in chloroform as solvent

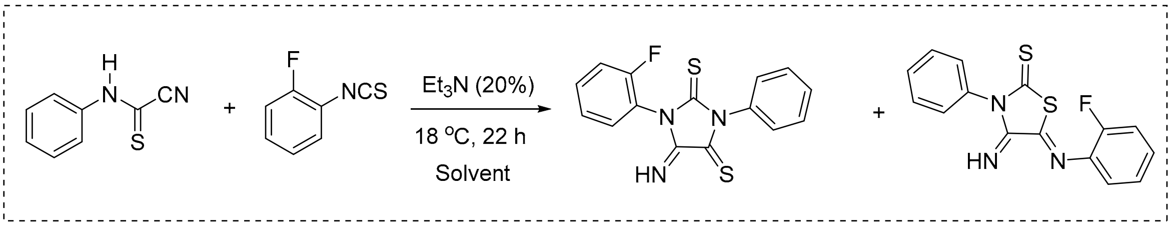

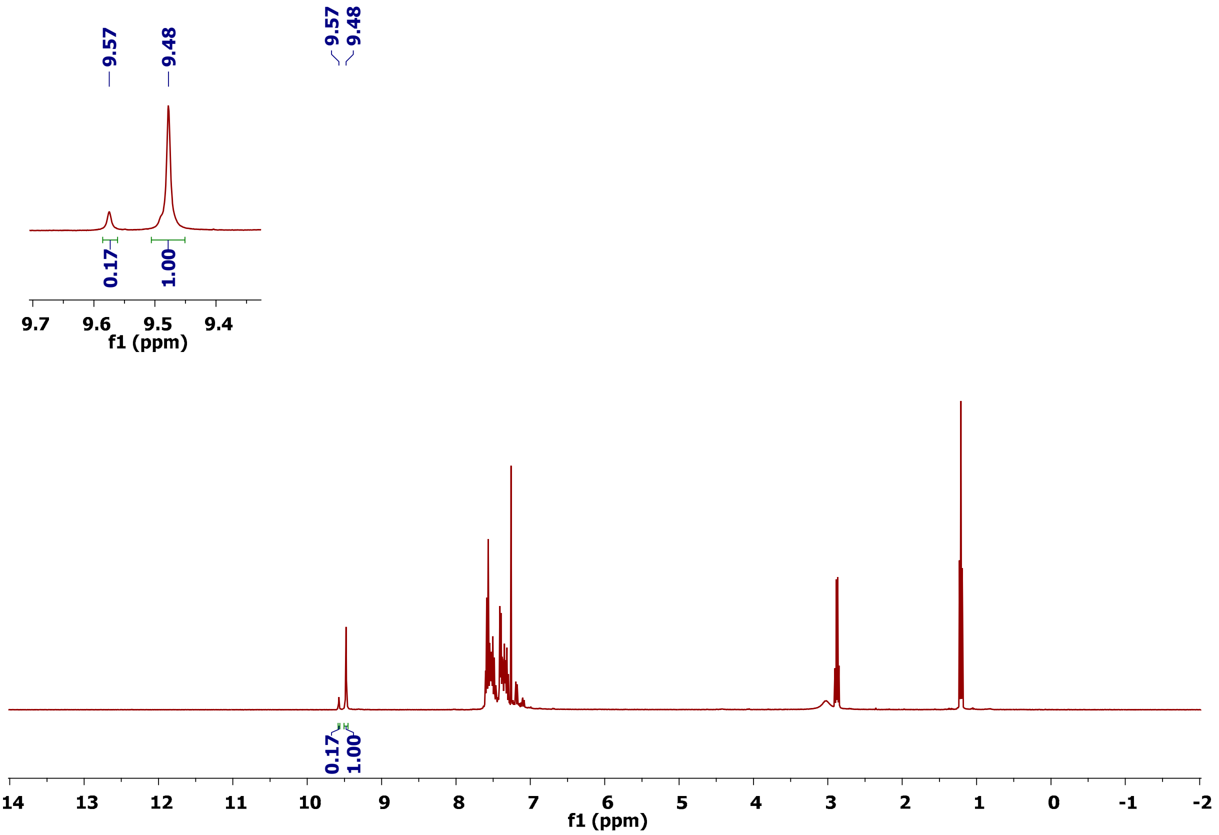

Ratio of 1-(2-fluorophenyl)-5-imino-3-phenylimidazolidine-2,4-dithione and (Z)-5-((2-fluorophenyl)imino)-4-imino-3-phenylthiazolidine-2-thione in acetone as solvent

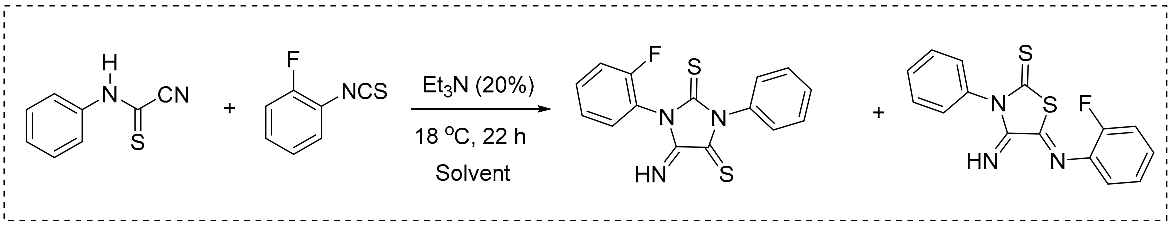

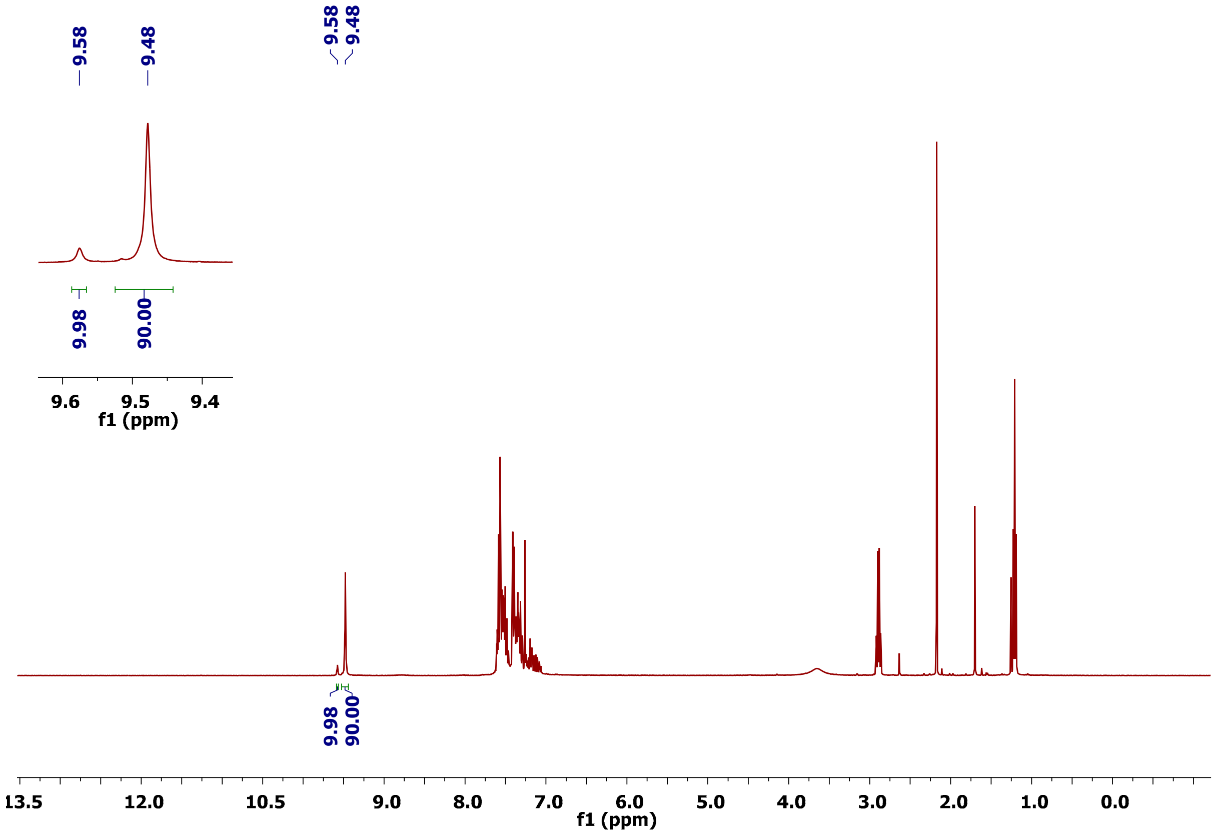

Ratio of 1-(2-fluorophenyl)-5-imino-3-phenylimidazolidine-2,4-dithione and (Z)-5-((2-fluorophenyl)imino)-4-imino-3-phenylthiazolidine-2-thione in acetonitrile as solvent

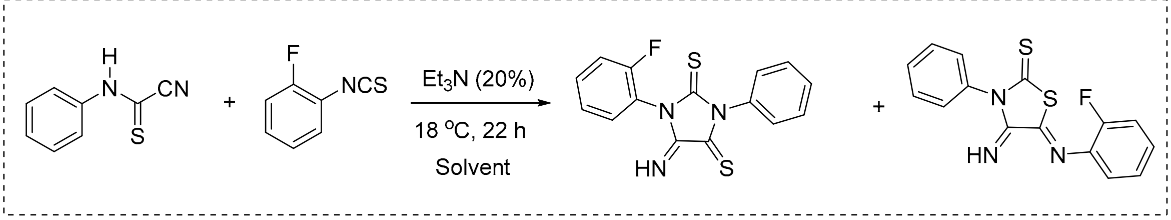

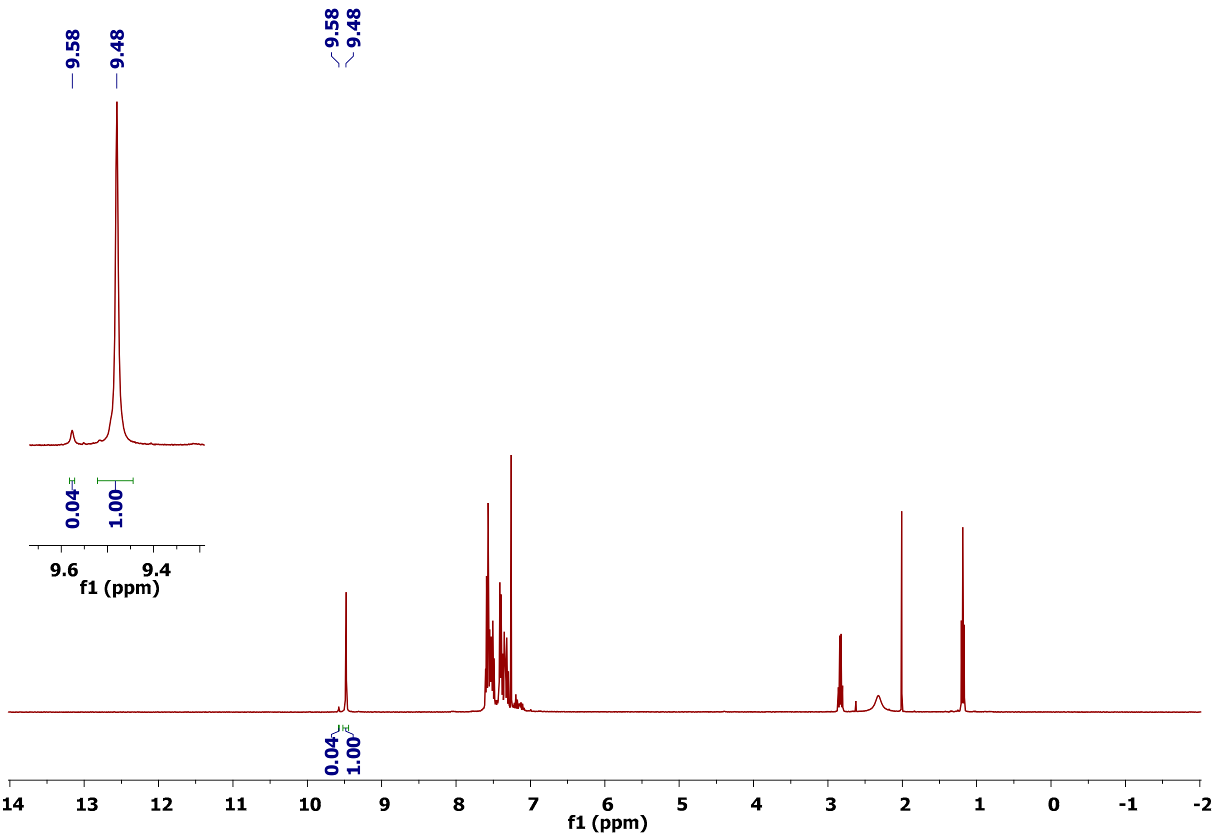

Ratio of 1-(2-fluorophenyl)-5-imino-3-phenylimidazolidine-2,4-dithione and (Z)-5-((2-fluorophenyl)imino)-4-imino-3-phenylthiazolidine-2-thione in DMSO as solvent

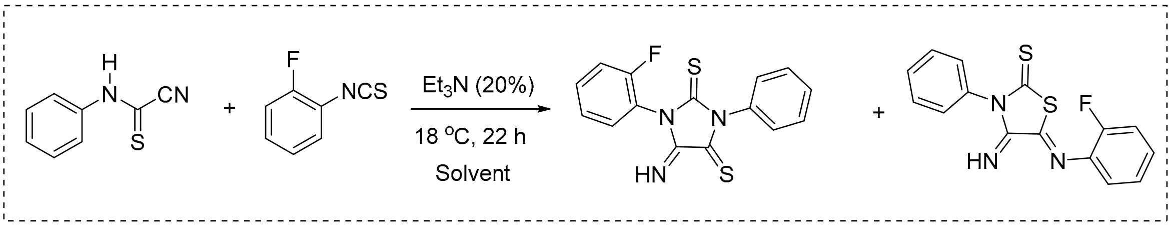

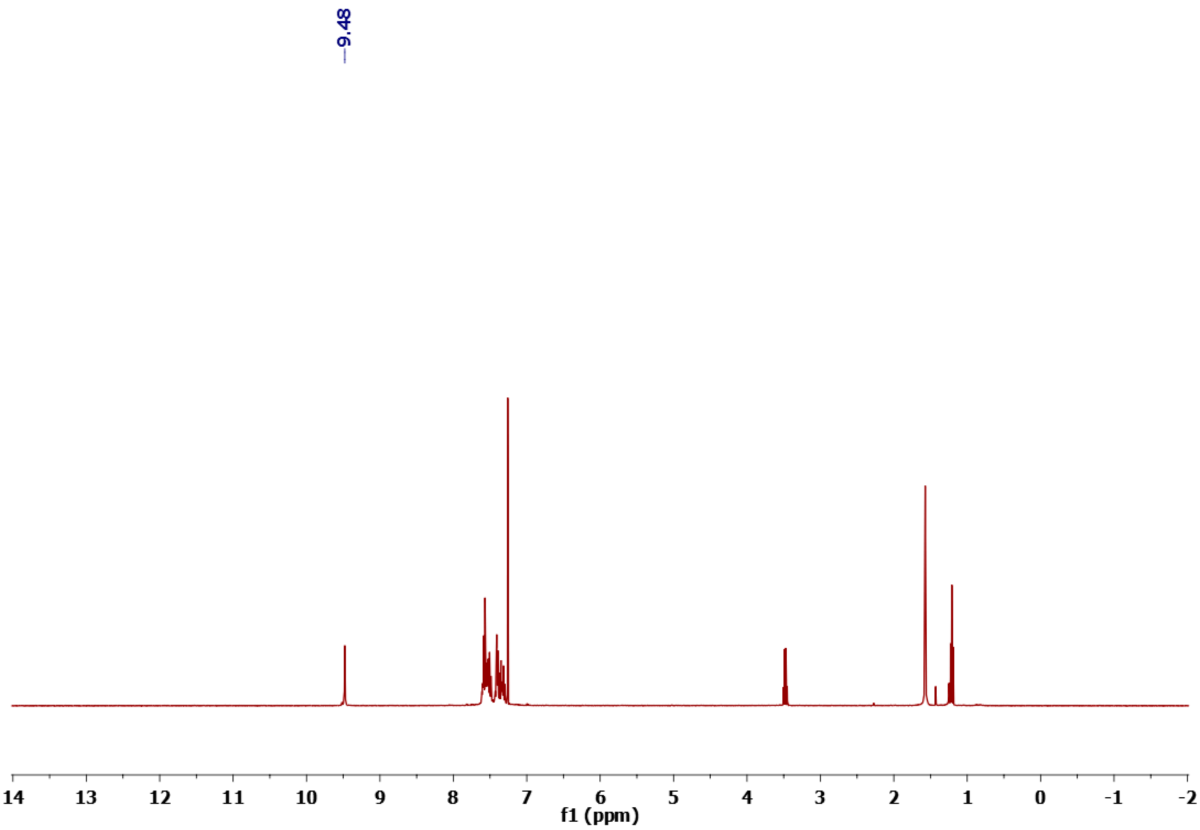

Ratio of 1-(2-fluorophenyl)-5-imino-3-phenylimidazolidine-2,4-dithione and (Z)-5-((2-fluorophenyl)imino)-4-imino-3-phenylthiazolidine-2-thione in DMF as solvent

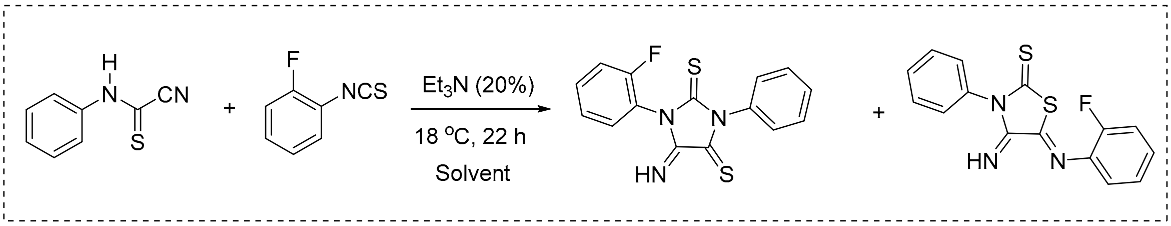

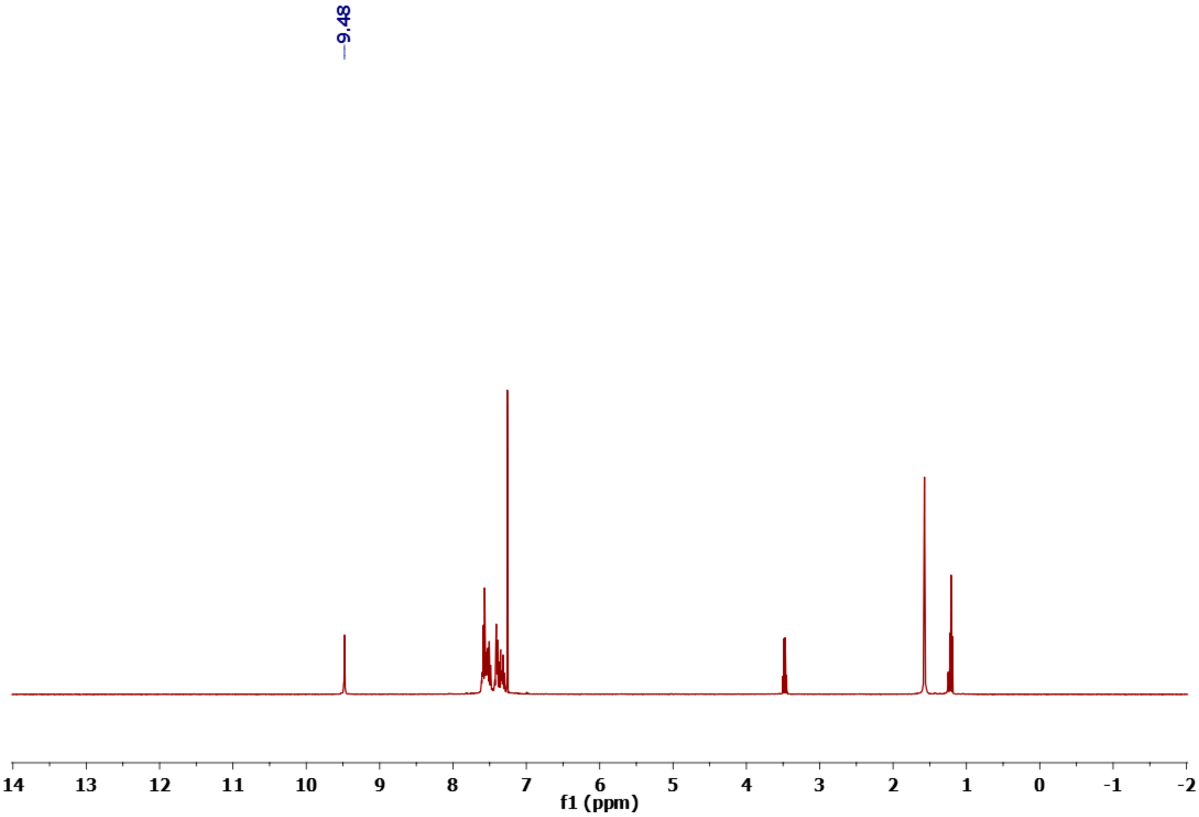

Ratio of 3-(2-fluorophenyl)-5-imino-1-phenylimidazolidine-2,4-dithione and (Z)-3-(2-fluorophenyl)-4-imino-5-(phenylimino)thiazolidine-2-thione in nitromethane as solvent

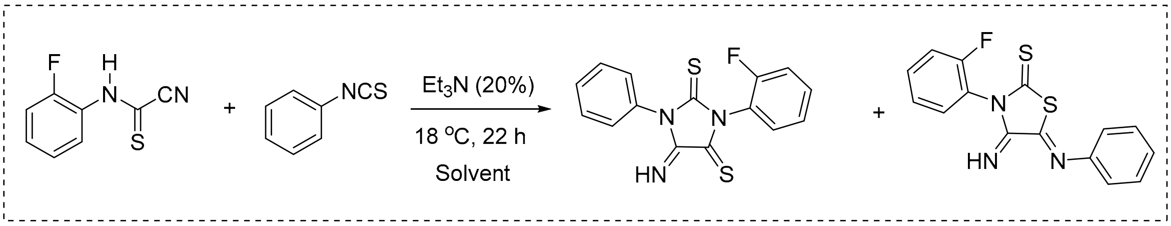

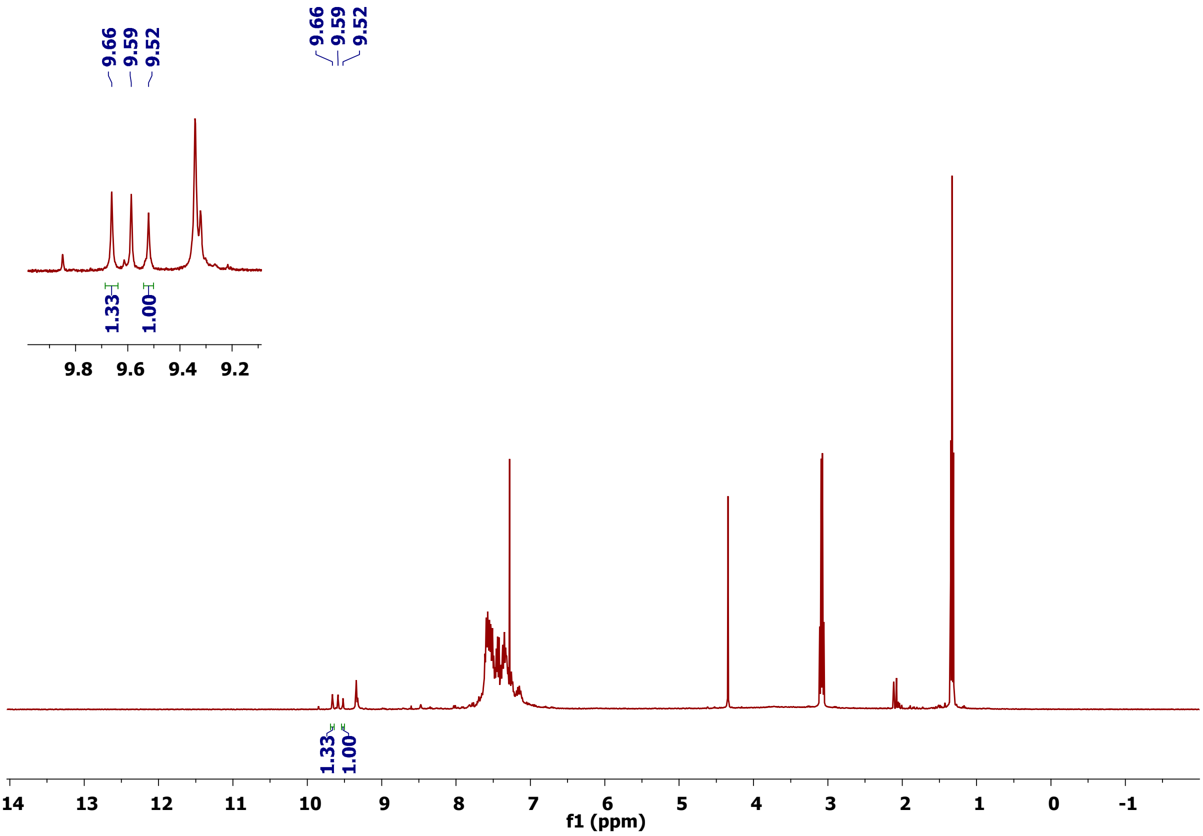

Ratio of 3-(2-fluorophenyl)-5-imino-1-phenylimidazolidine-2,4-dithione and (Z)-3-(2-fluorophenyl)-4-imino-5-(phenylimino)thiazolidine-2-thione in ethyl acetate as solvent

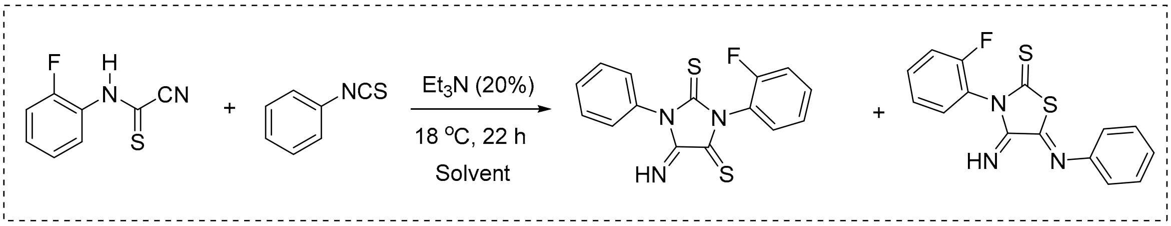

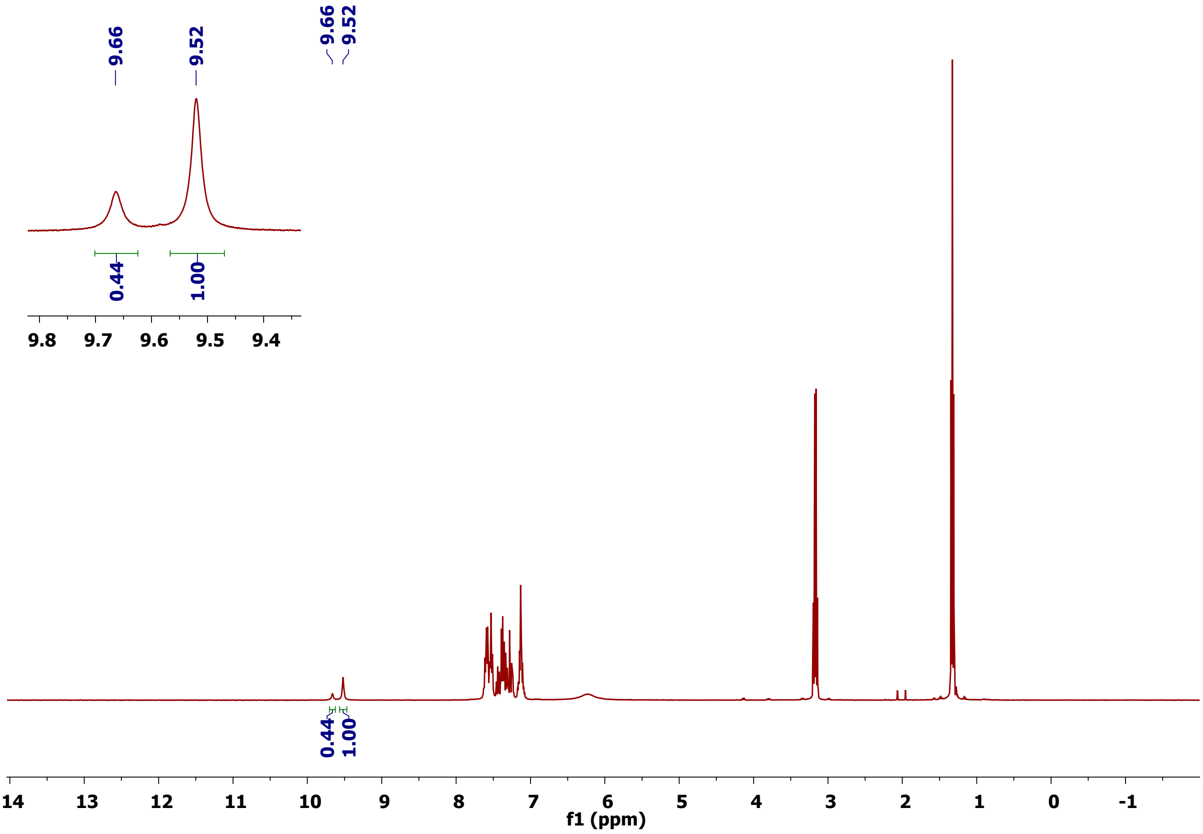

Ratio of 3-(2-fluorophenyl)-5-imino-1-phenylimidazolidine-2,4-dithione and (Z)-3-(2-fluorophenyl)-4-imino-5-(phenylimino)thiazolidine-2-thione in THF as solvent

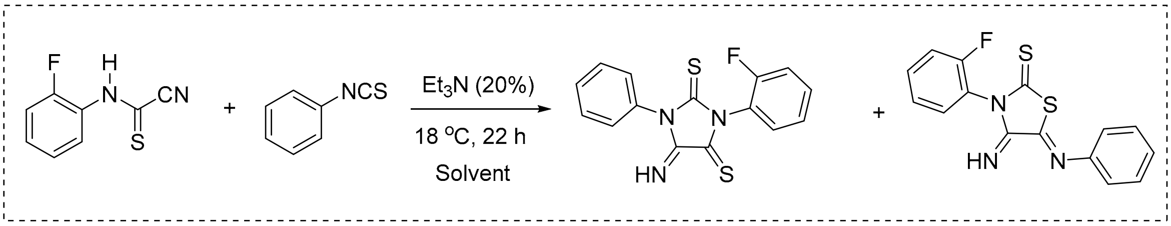

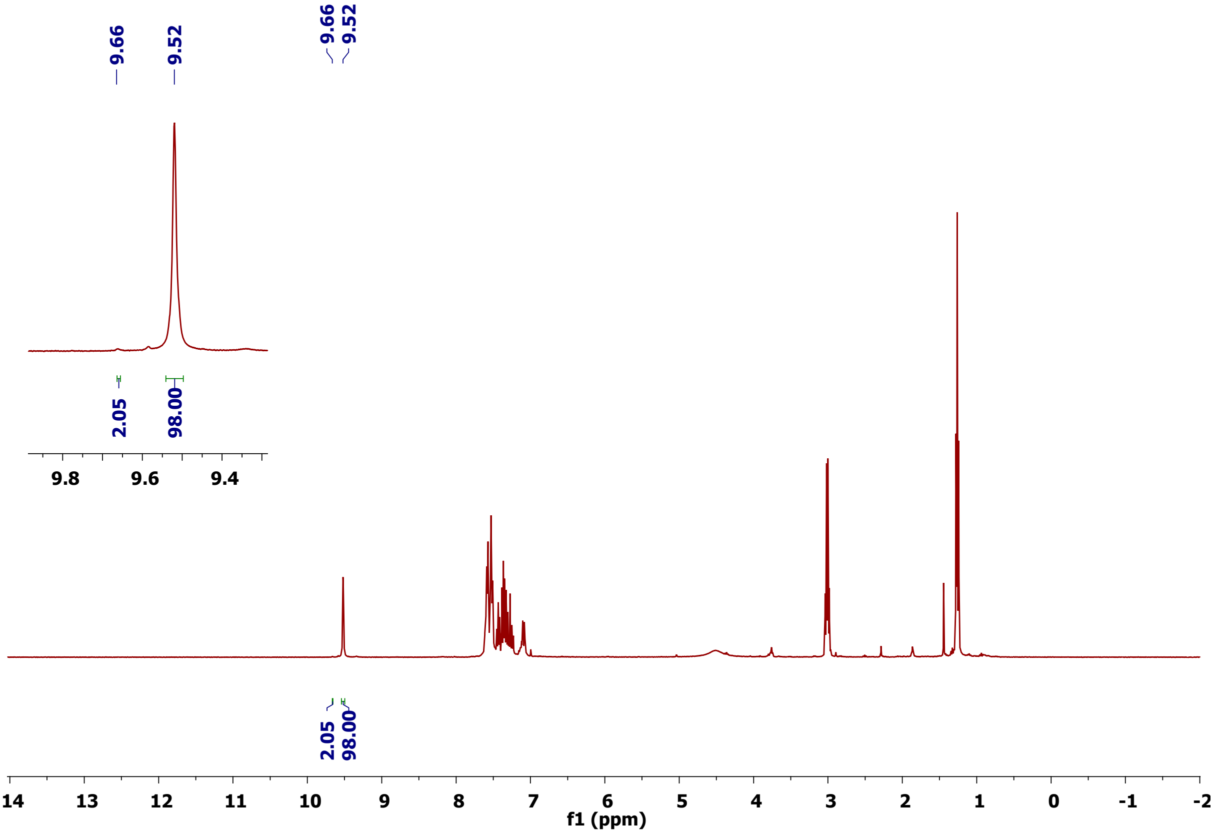

Ratio of 3-(2-fluorophenyl)-5-imino-1-phenylimidazolidine-2,4-dithione and (Z)-3-(2-fluorophenyl)-4-imino-5-(phenylimino)thiazolidine-2-thione in methanol as solvent

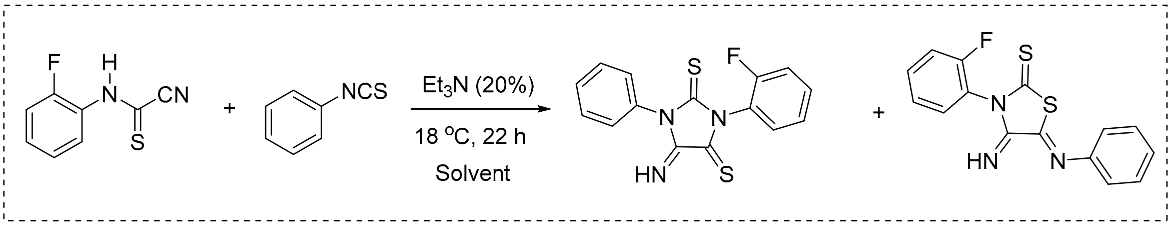

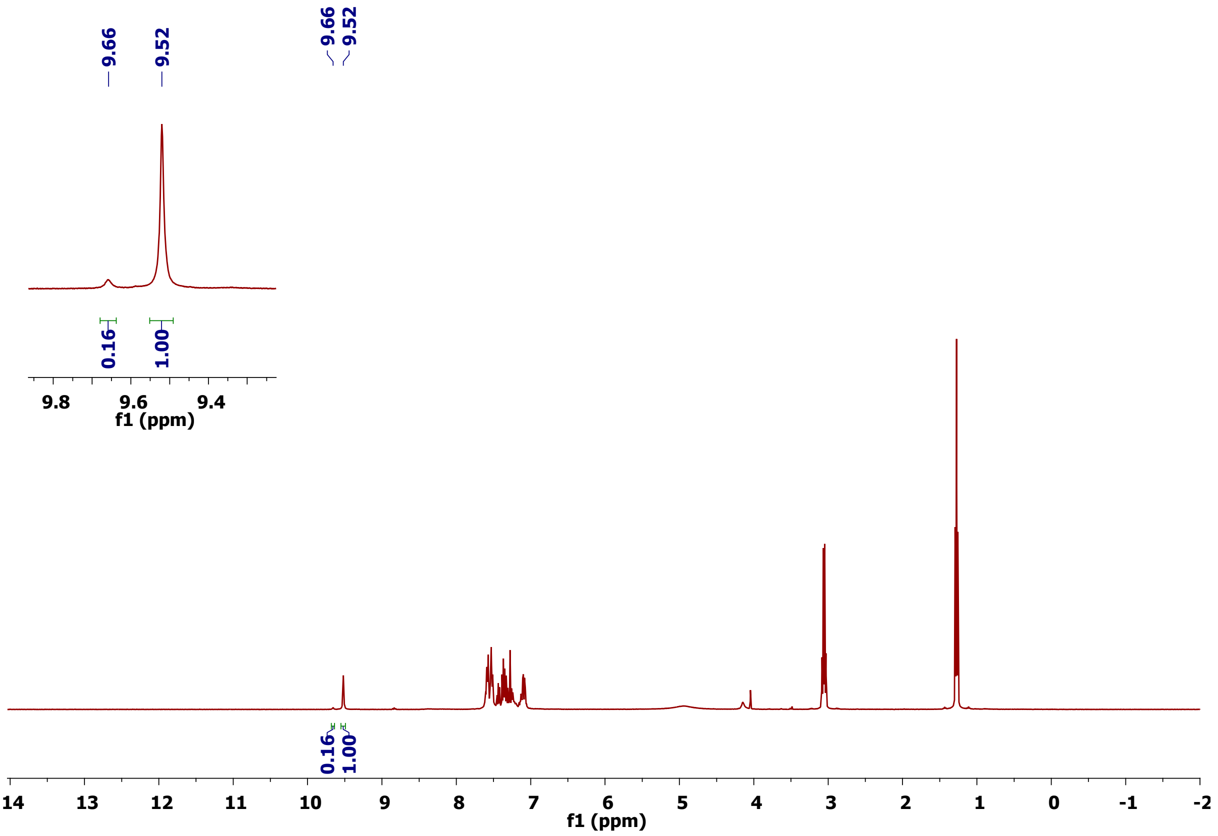

Ratio of 3-(2-fluorophenyl)-5-imino-1-phenylimidazolidine-2,4-dithione and (Z)-3-(2-fluorophenyl)-4-imino-5-(phenylimino)thiazolidine-2-thione in diethyl ether as solvent

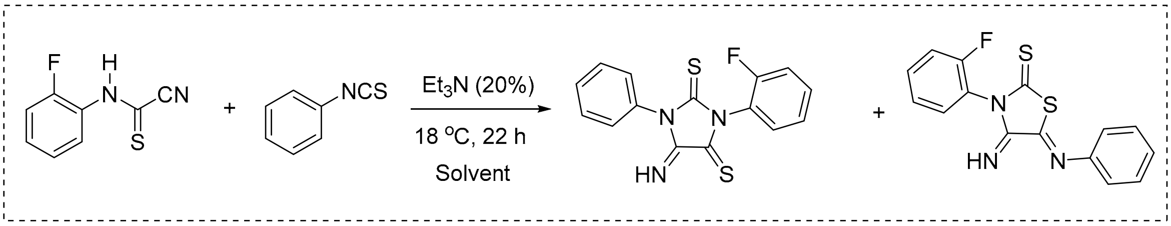

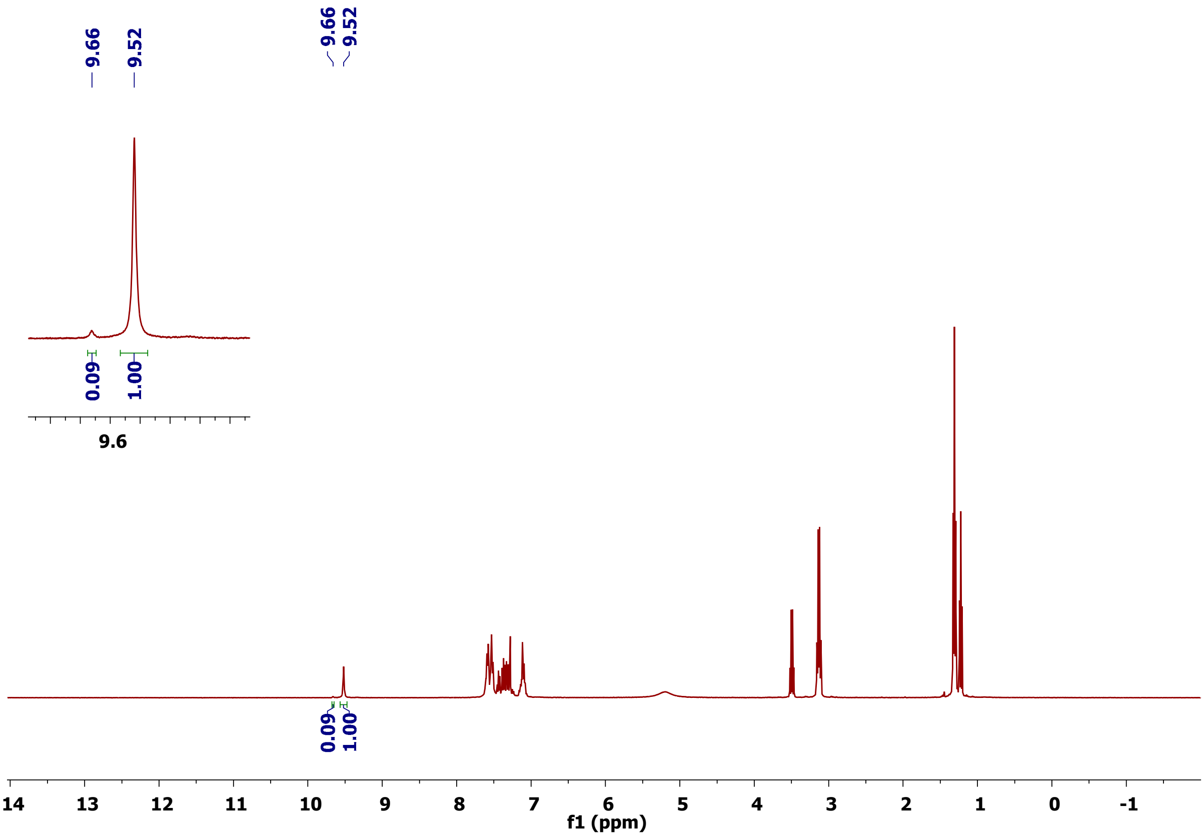

Ratio of 3-(2-fluorophenyl)-5-imino-1-phenylimidazolidine-2,4-dithione and (Z)-3-(2-fluorophenyl)-4-imino-5-(phenylimino)thiazolidine-2-thione in ethanol as solvent

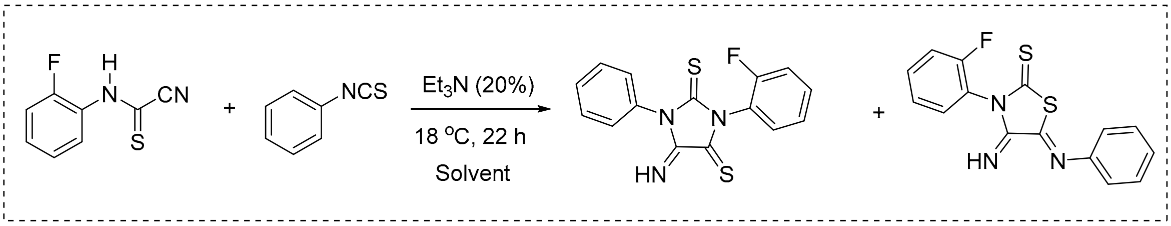

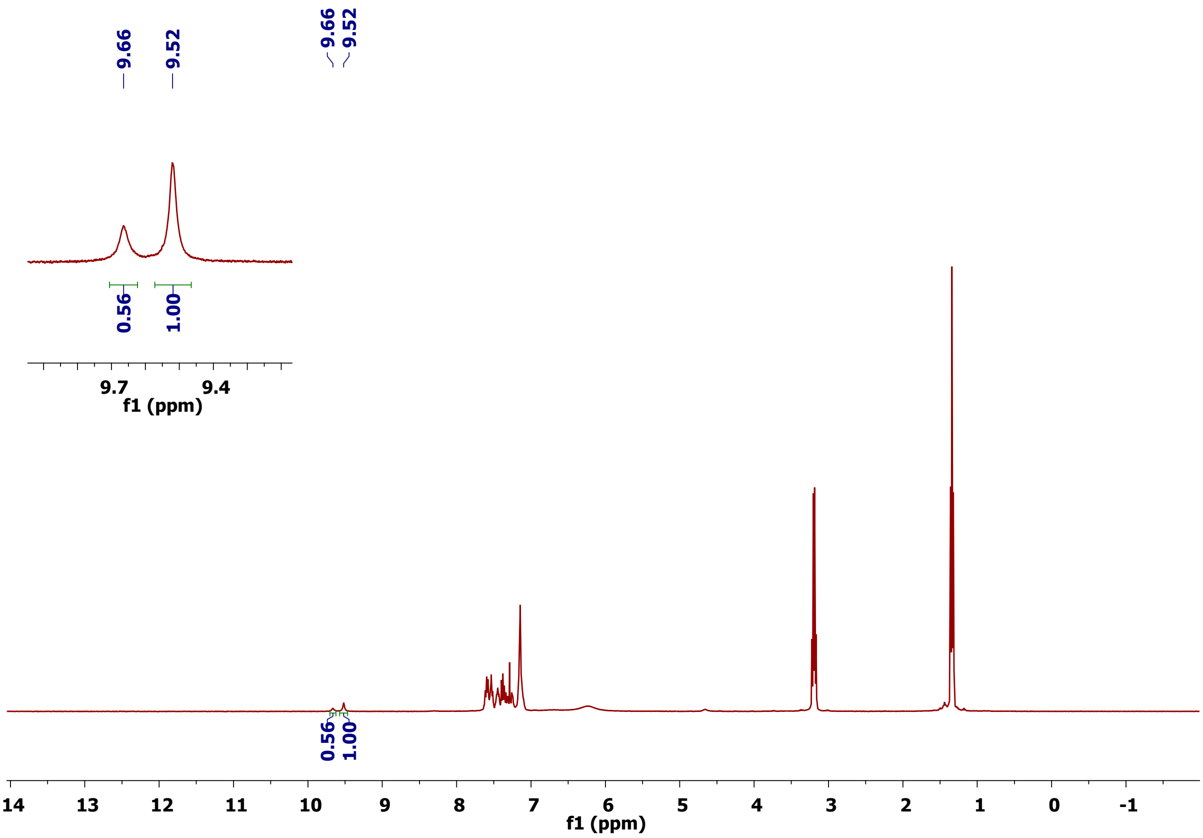

Ratio of 3-(2-fluorophenyl)-5-imino-1-phenylimidazolidine-2,4-dithione and (Z)-3-(2-fluorophenyl)-4-imino-5-(phenylimino)thiazolidine-2-thione in toluene as solvent

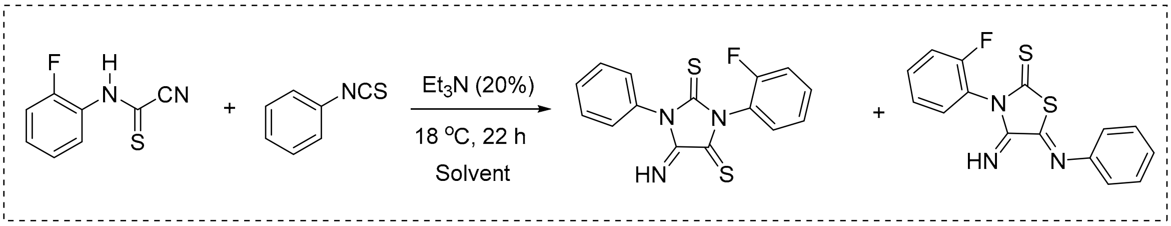

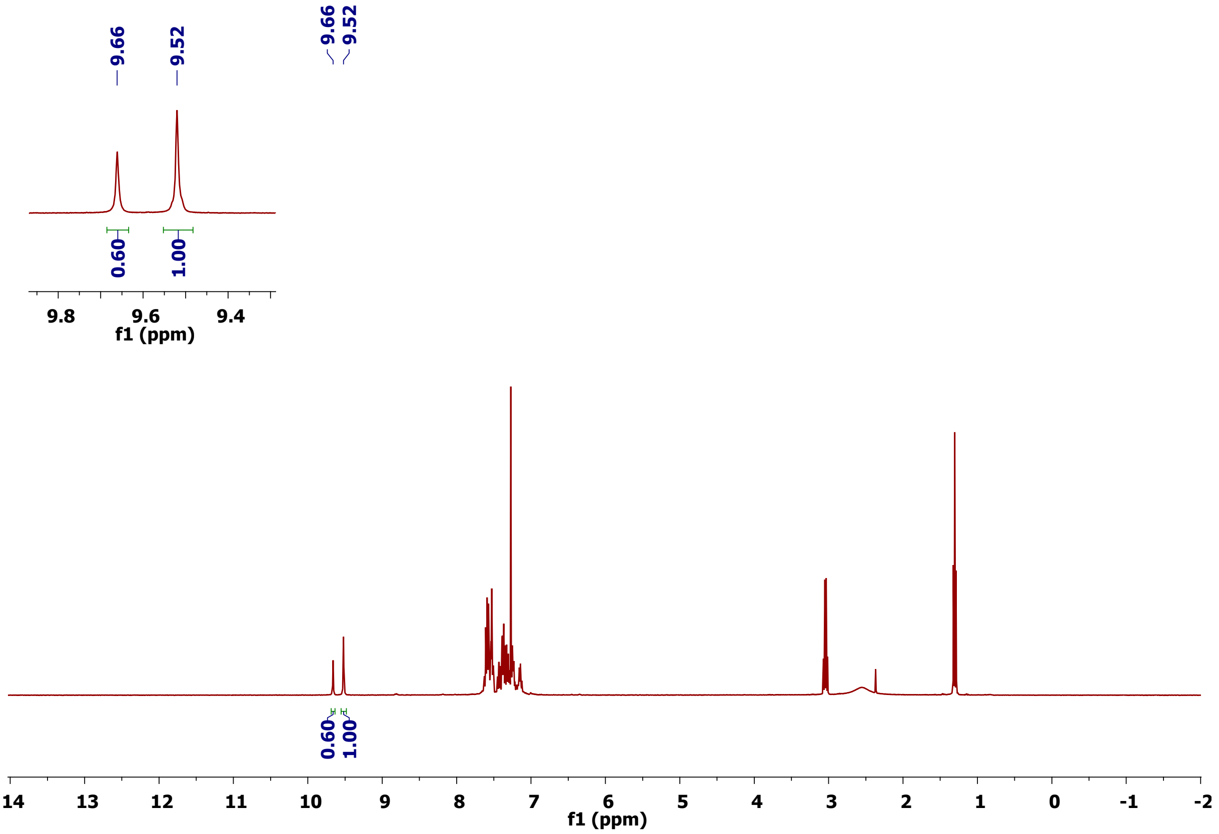

Ratio of 3-(2-fluorophenyl)-5-imino-1-phenylimidazolidine-2,4-dithione and (Z)-3-(2-fluorophenyl)-4-imino-5-(phenylimino)thiazolidine-2-thione in dioxane as solvent

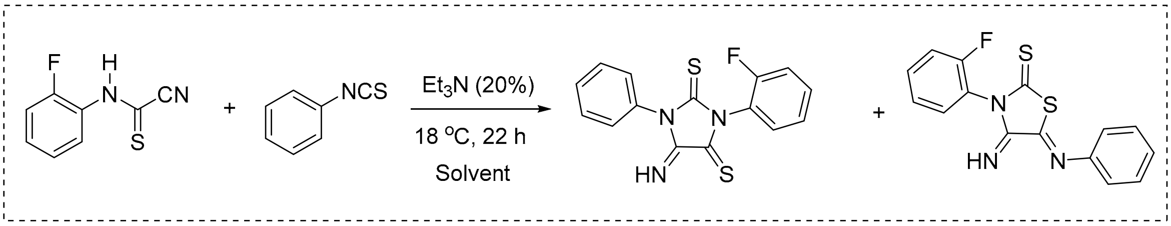

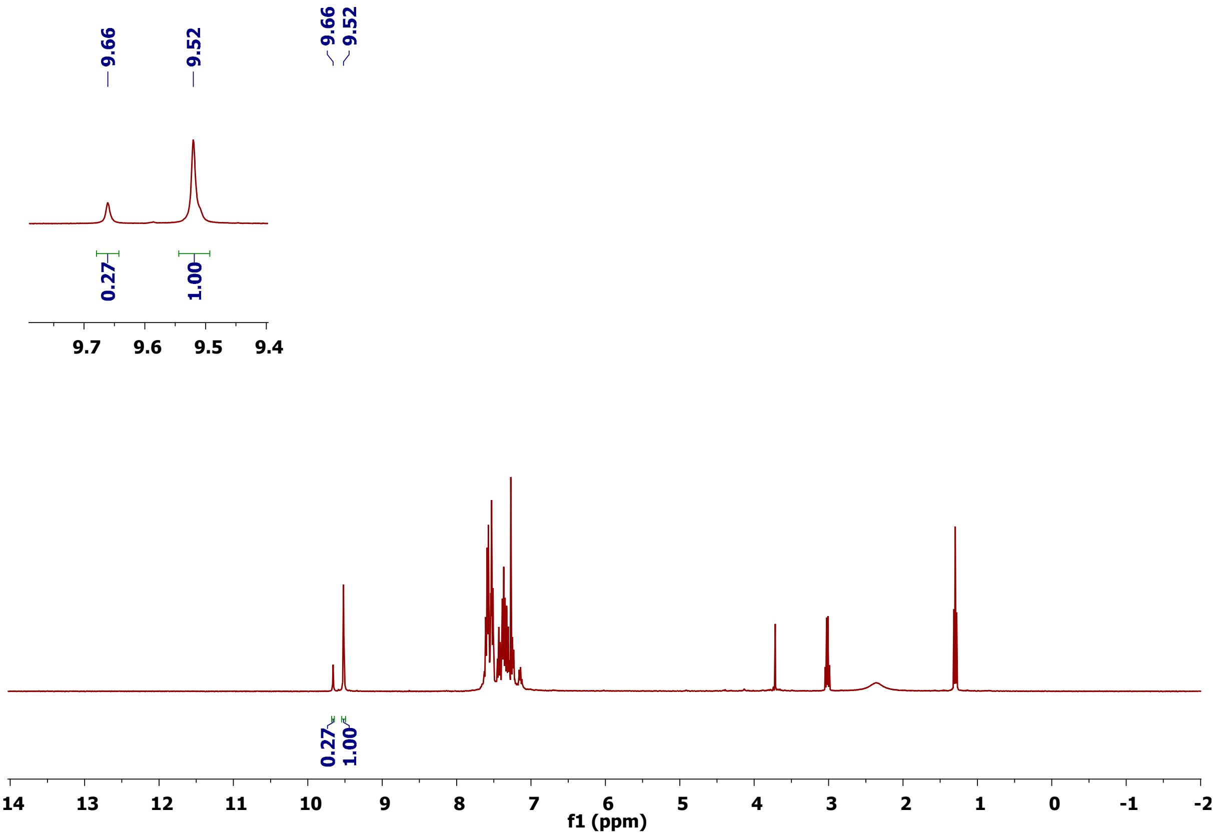

Ratio of 3-(2-fluorophenyl)-5-imino-1-phenylimidazolidine-2,4-dithione and (Z)-3-(2-fluorophenyl)-4-imino-5-(phenylimino)thiazolidine-2-thione in benzene as solvent

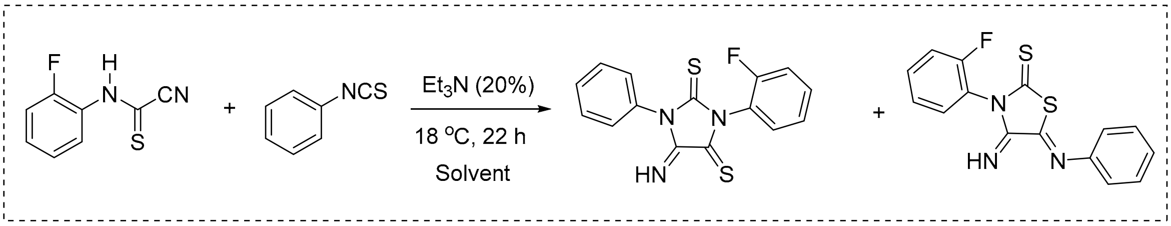

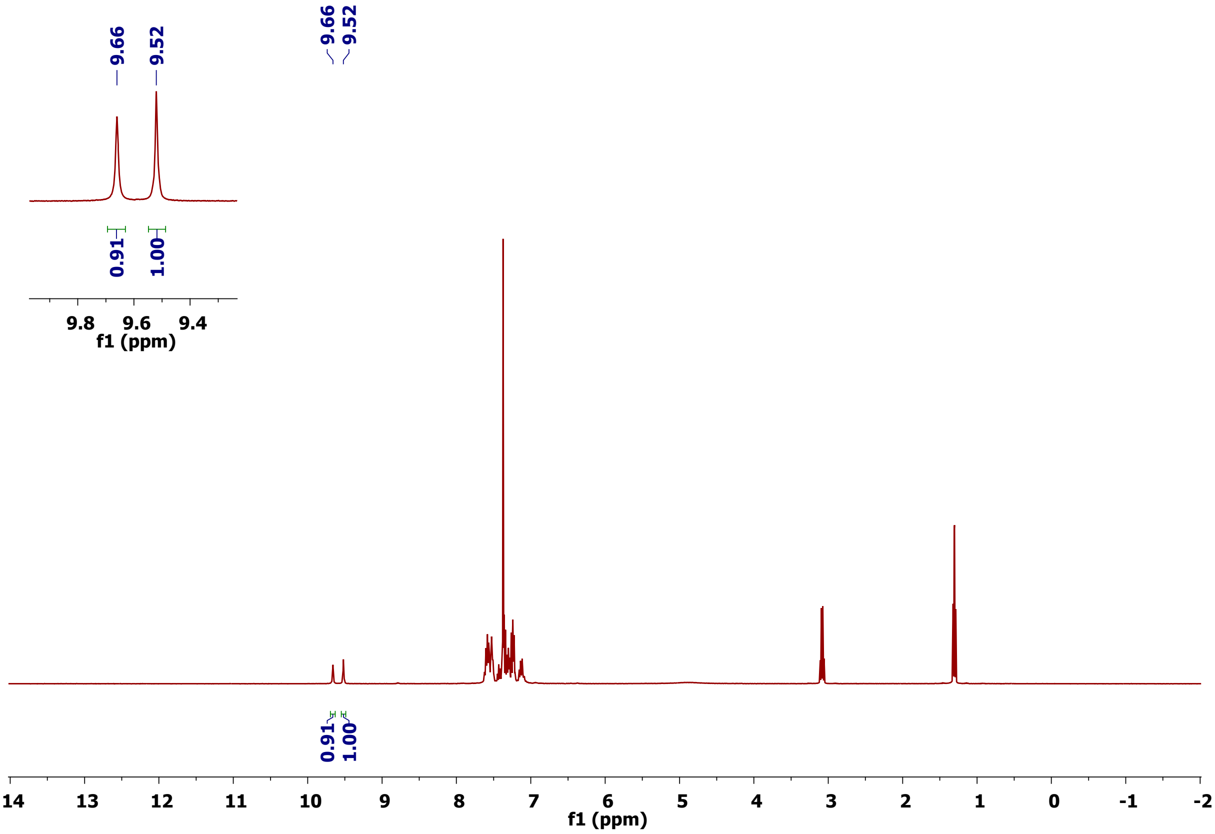

Ratio of 3-(2-fluorophenyl)-5-imino-1-phenylimidazolidine-2,4-dithione and (Z)-3-(2-fluorophenyl)-4-imino-5-(phenylimino)thiazolidine-2-thione in DCM as solvent

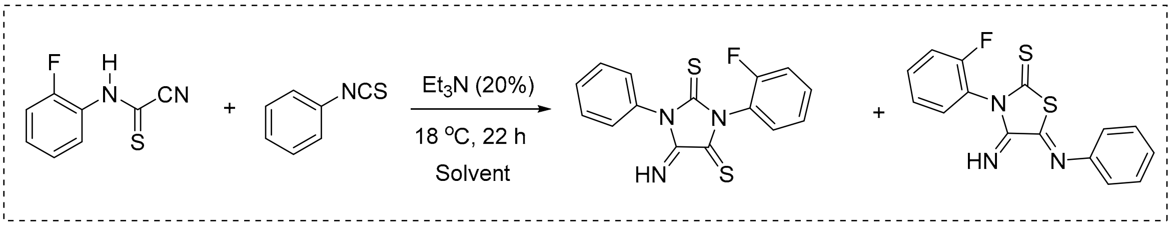

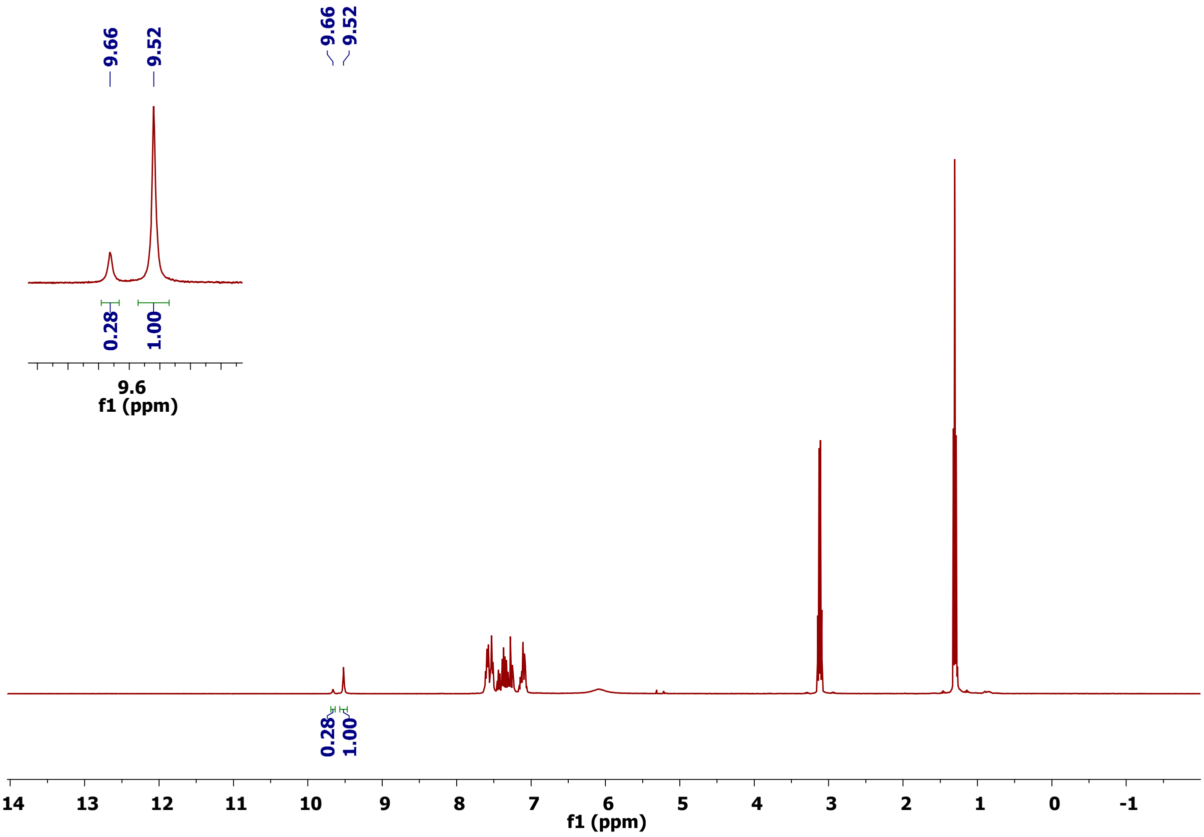

Ratio of 3-(2-fluorophenyl)-5-imino-1-phenylimidazolidine-2,4-dithione and (Z)-3-(2-fluorophenyl)-4-imino-5-(phenylimino)thiazolidine-2-thione in chloroform as solvent

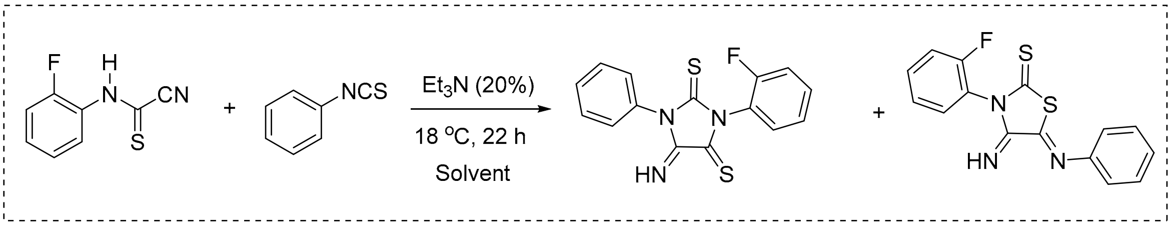

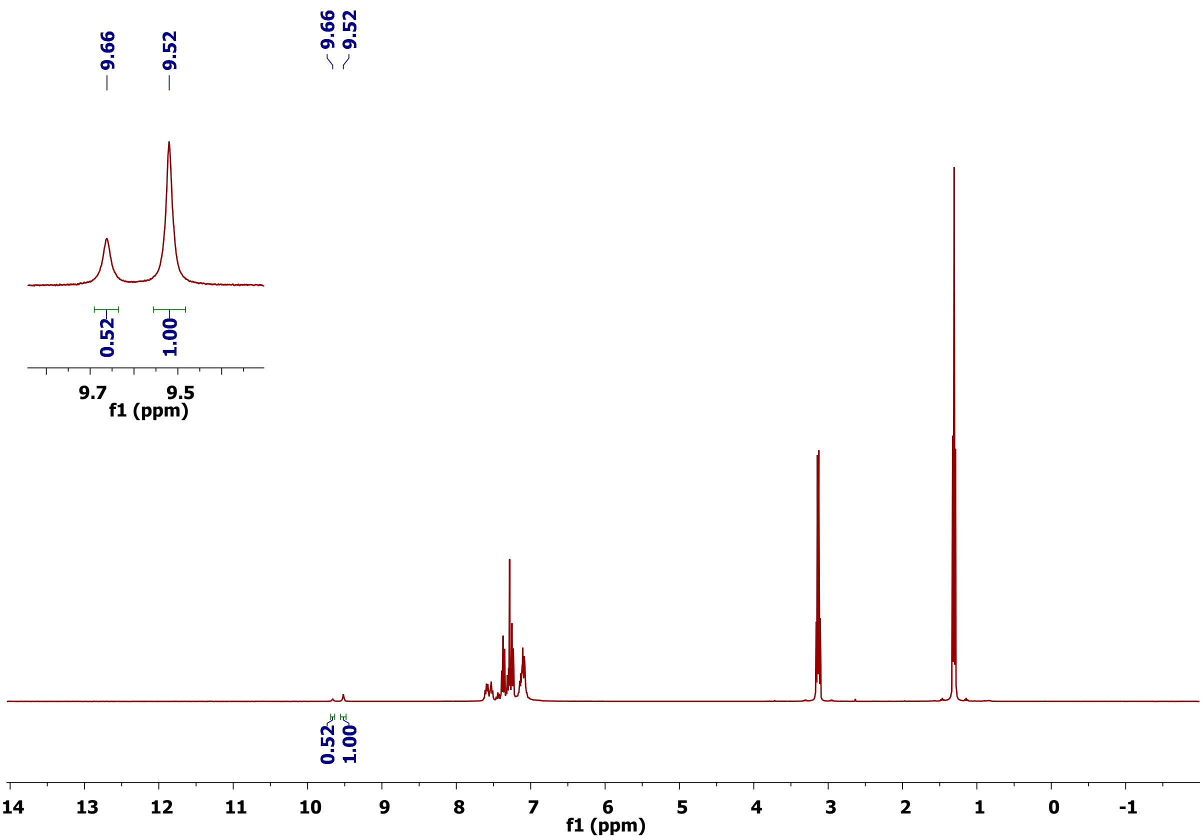

Ratio of 3-(2-fluorophenyl)-5-imino-1-phenylimidazolidine-2,4-dithione and (Z)-3-(2-fluorophenyl)-4-imino-5-(phenylimino)thiazolidine-2-thione in acetonitrile as solvent

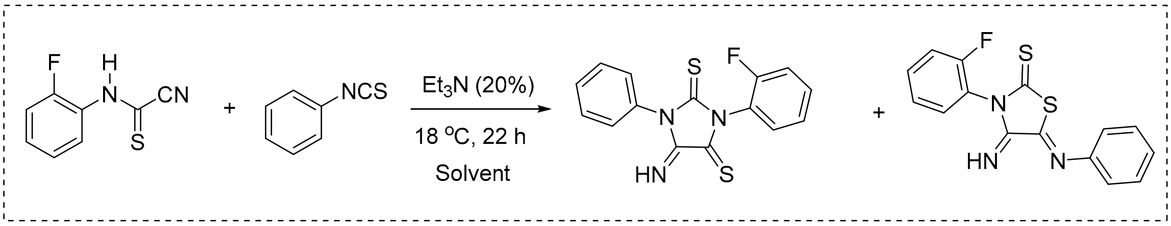

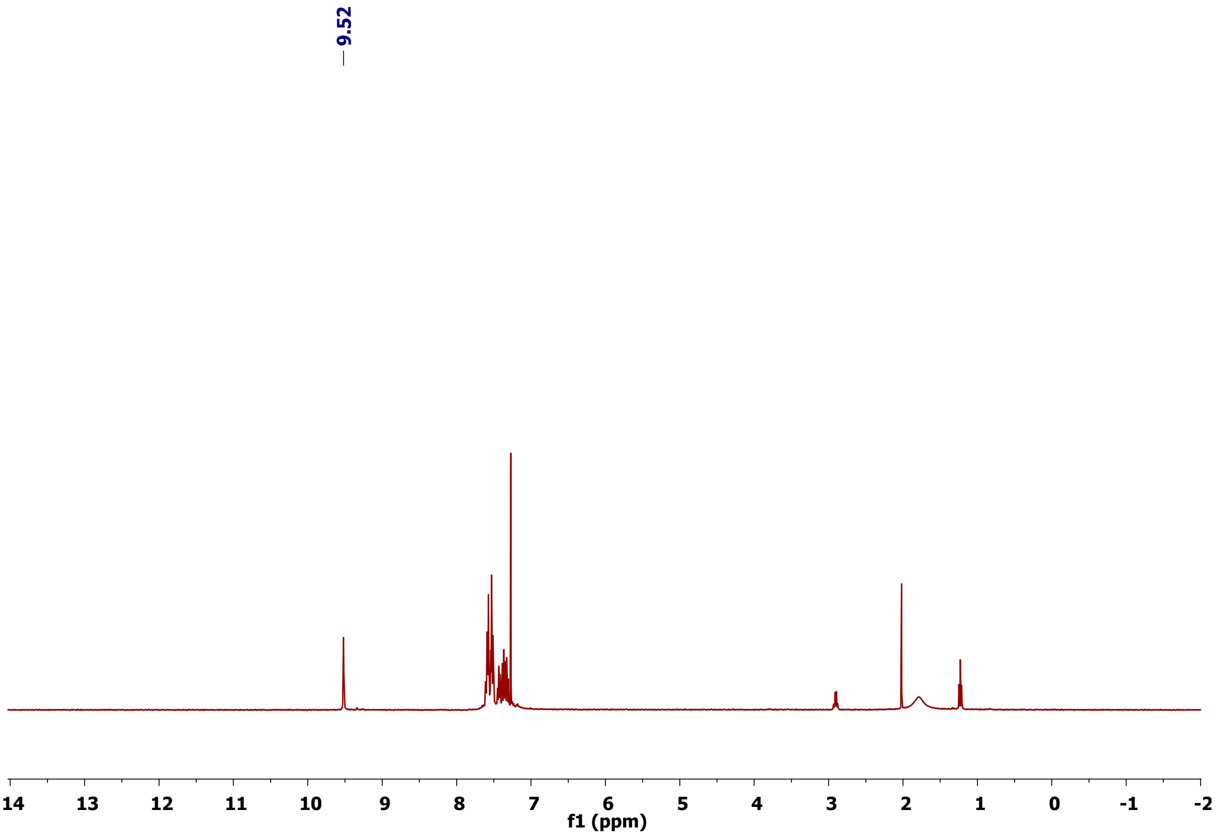

Ratio of 3-(2-fluorophenyl)-5-imino-1-phenylimidazolidine-2,4-dithione and (Z)-3-(2-fluorophenyl)-4-imino-5-(phenylimino)thiazolidine-2-thione in acetone as solvent

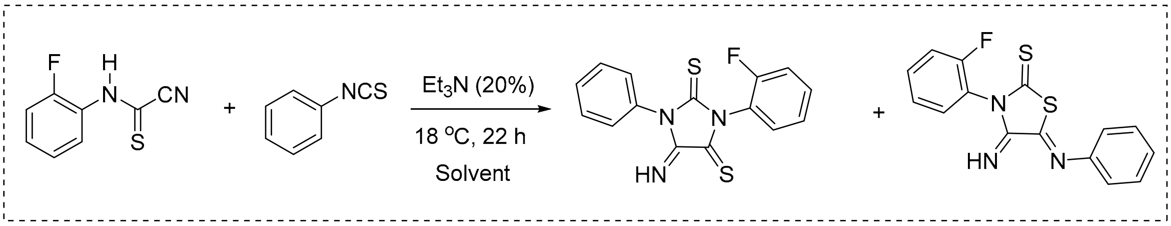

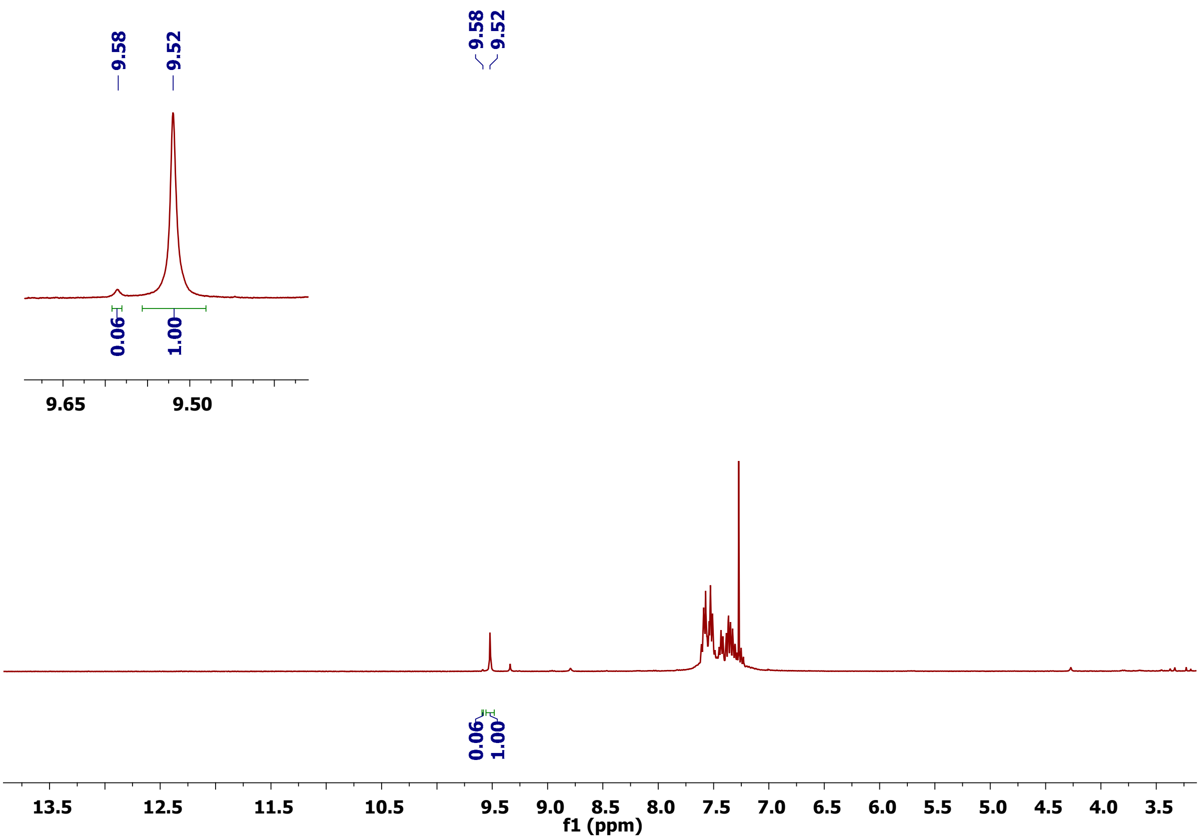

Ratio of 3-(2-fluorophenyl)-5-imino-1-phenylimidazolidine-2,4-dithione and (Z)-3-(2-fluorophenyl)-4-imino-5-(phenylimino)thiazolidine-2-thione in DMSO as solvent

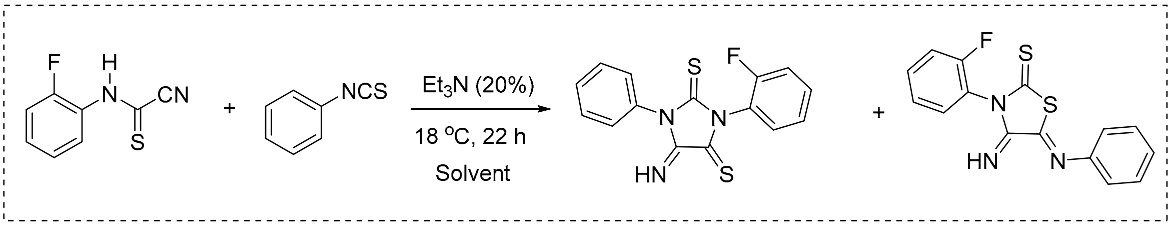

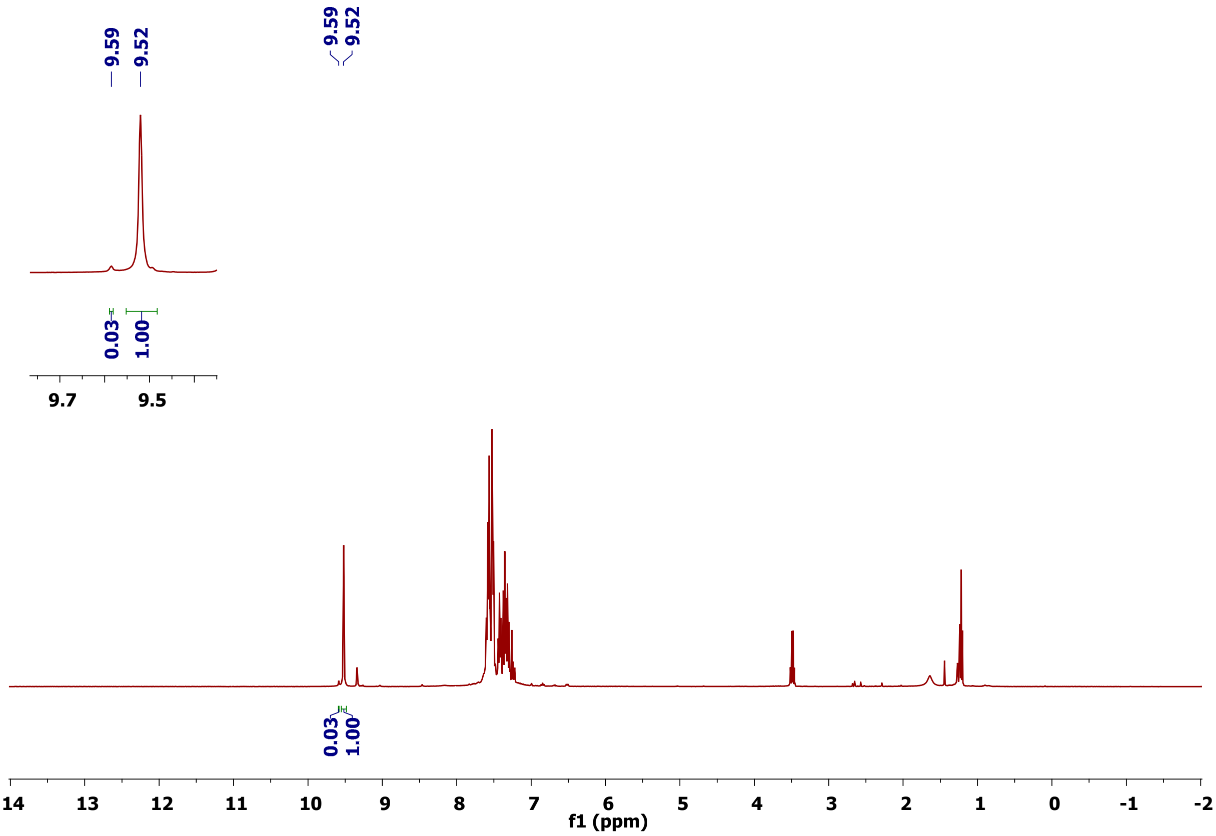

C

Ratio of 3-(2-fluorophenyl)-5-imino-1-phenylimidazolidine-2,4-dithione and (Z)-3-(2-fluorophenyl)-4-imino-5-(phenylimino)thiazolidine-2-thione in DMF as solvent

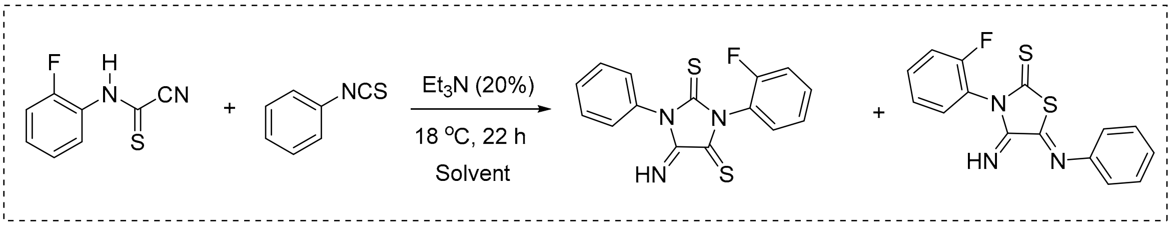

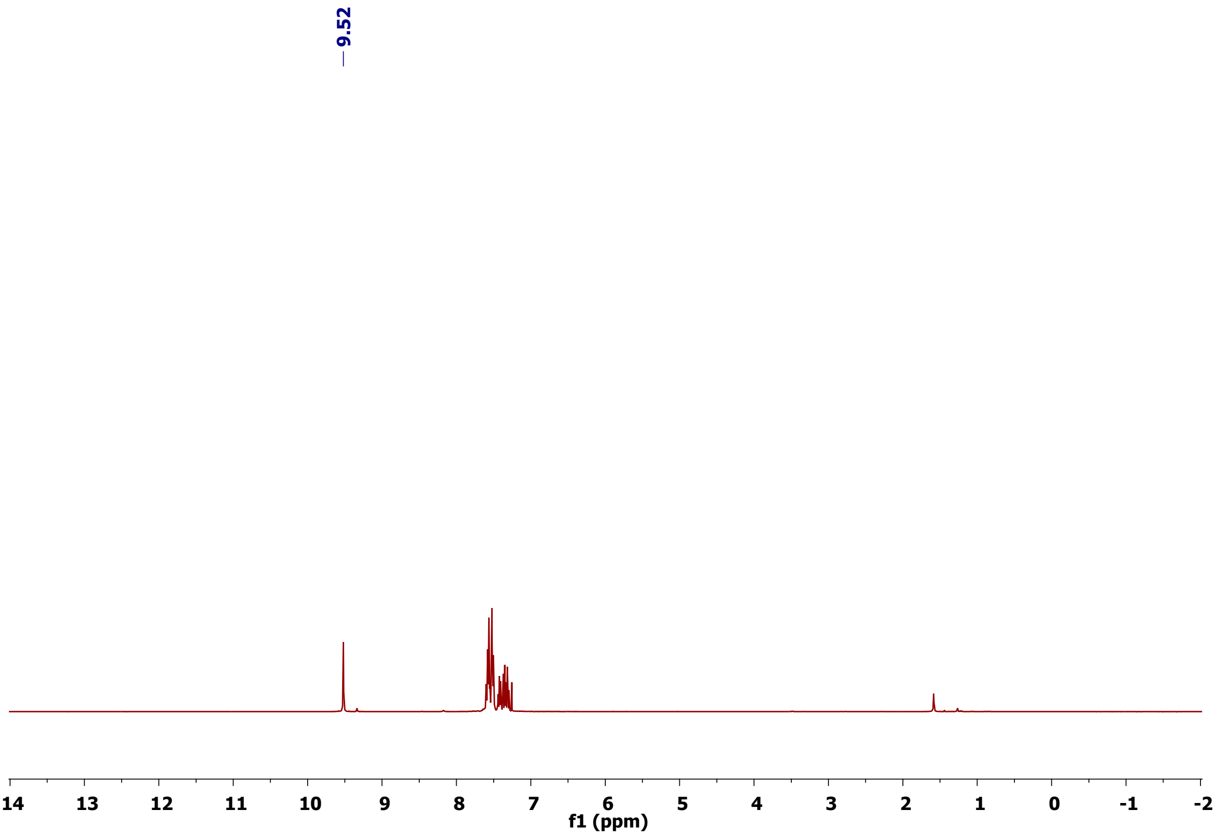

Crystal-structure data for (4-methoxyphenyl)carbamothioyl cyanide (**1g**)

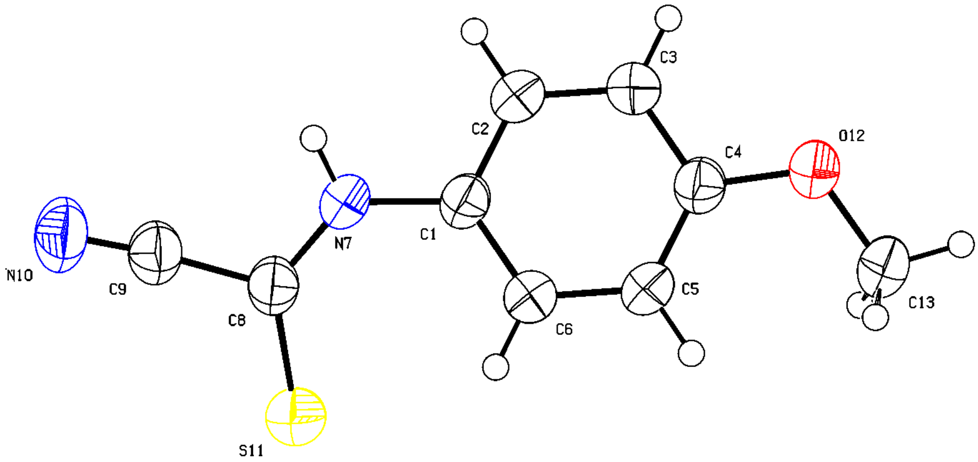

Figure S1. Thermal ellipsoid plots of (4-methoxyphenyl)carbamothioyl cyanide (**1g**) (Deposition Number **2246321**) with ellipsoids drawn at 50% probability level. No absorption correction was applied. Selected bond distances (Å) and angles (deg) for **1g:** S(11)-C(8) = 1.647(2), C(4)-C(5) = 1.378(3), O(12)-C(4) = 1.365(2), C(4)-C(3) = 1.390(3), O(12)-C(13) = 1.427(3), N(10)-C(9) = 1.138(3), N(7)-C(1) = 1.420(2), C(5)-C(6) = 1.391(3), N(7)-C(8) = 1.332(3), C(2)-C(3) = 1.373(3), C(1)-C(2) = 1.390(3), C(8)-C(9) = 1.456(3), C(1)-C(6) = 1.380(3), C(4)-O(12)-C(13) = 117.09(17), C(4)-C(5)-C(6) = 120.43(19), C(8)-N(7)-C(1) = 129.61(17), C(3)-C(2)-C(1) = 120.4(2), C(2)-C(1)-N(7) = 116.85(18), N(7)-C(8)-S(11) = 131.32(16), C(6)-C(1)-N(7) = 123.71(19), N(7)-C(8)-C(9) =112.89(19), C(6)-C(1)-C(2) = 119.39(19), C(9)-(C)8-S(11) = 115.78(17), O(12)-C(4)-C(5) = 124.69(19), C(1)-C(6)-C(5) = 120.1(2), O(12)-C(4)-C(3) = 116.07(19), C(2)-C(3)-C(4) = 120.4(2), C(5)-C(4)-C(3) = 119.23(19), N(10)-C(9)-C(8) = 175.4(3).

exp_1g_auto

Table S1 Crystal data and structure refinement for exp_1g_auto.

Identification code	exp_1g_auto
Empirical formula	$C_9H_8N_2OS$
Formula weight	192.23
Temperature/K	297.00(10)
Crystal system	monoclinic
Space group	$P2_1/n$
a/Å	10.4448(5)
b/Å	7.3755(4)
c/Å	12.1568(6)
α/°	90
β/°	100.367(5)
γ/°	90
Volume/Å^3	921.22(8)

Z	4
ρ_{calc}g/cm^3	1.386
μ/mm^{-1}	2.794
F(000)	400.0
Crystal size/mm^3	0.143 × 0.072 × 0.057
Radiation	Cu Kα (λ = 1.54184)
2Θ range for data collection/°	10.292 to 155.116
Index ranges	-12 ≤ h ≤ 13, -8 ≤ k ≤ 8, -12 ≤ l ≤ 15
Reflections collected	8594
Independent reflections	1834 [R_{int} = 0.0381, R_{sigma} = 0.0278]
Data/restraints/parameters	1834/0/119
Goodness-of-fit on F^2	1.051
Final R indexes [I>=2σ (I)]	R_1 = 0.0474, wR_2 = 0.1330
Final R indexes [all data]	R_1 = 0.0561, wR_2 = 0.1400
Largest diff. peak/hole / e Å^{-3}	0.30/-0.29

Table S2 Fractional Atomic Coordinates (×10^4) and Equivalent Isotropic Displacement Parameters (Å^2×10^3) for exp_1g_auto. U_{eq} is defined as 1/3 of the trace of the orthogonalised U_{IJ} tensor.

Atom	*x*	*y*	*z*	U(eq)
S11	5902.1(6)	2064.3(9)	2596.6(6)	69.3(3)
O12	9044.3(14)	8812(2)	5864.0(14)	57.7(4)
N7	4867.6(16)	5026(2)	3437.6(14)	45.8(4)
C1	5982.3(18)	5888(3)	4061.9(16)	43.3(5)
C4	8069(2)	7768(3)	51g.8(17)	46.5(5)
N10	2429(2)	2563(3)	1953(2)	68.2(6)
C5	8149(2)	5923(3)	5134.8(18)	50.8(5)
C2	5887(2)	7737(3)	4254(2)	53.0(6)
C8	4778(2)	3485(3)	2857.5(17)	46.1(5)
C6	7110(2)	4981(3)	4514.4(18)	50.3(5)
C3	6916(2)	8665(3)	4861.2(19)	55.3(6)
C9	3444(2)	3033(3)	2354.0(19)	52.5(5)
C13	10323(2)	8056(4)	6063(2)	61.2(6)

Table S3 Anisotropic Displacement Parameters (Å^2×10^3) for exp_1g_auto. The Anisotropic displacement factor exponent takes the form: $-2\pi^2[h^2a^{*2}U_{11}+2hka^*b^*U_{12}+\ldots]$.

Atom	U_{11}	U_{22}	U_{33}	U_{23}	U_{13}	U_{12}
S11	55.3(4)	61.9(4)	85.0(5)	-20.7(3)	-2.7(3)	9.7(3)
O12	41.2(8)	55.9(10)	70.9(10)	-17.6(8)	-3.3(7)	1.1(7)
N7	33.8(8)	49.2(10)	51.5(9)	-2.3(8)	-0.3(7)	2.5(7)

Table S3 Anisotropic Displacement Parameters (Å²×10³) for exp_1g_auto. The Anisotropic displacement factor exponent takes the form: $-2\pi^2[h^2a^{*2}U_{11}+2hka^*b^*U_{12}+\ldots]$.

Atom	U_{11}	U_{22}	U_{33}	U_{23}	U_{13}	U_{12}
C1	36.2(9)	48.0(11)	43.5(10)	-0.8(9)	1.3(8)	1.1(8)
C4	40.5(11)	50.8(12)	46.2(11)	-7.2(9)	2.7(9)	0.3(9)
N10	49.1(12)	74.2(14)	76.4(13)	-2.8(11)	-1.6(10)	-16.4(10)
C5	39.2(10)	49.6(12)	58.8(13)	-4.3(10)	-4.4(9)	7.4(9)
C2	42.6(12)	47.7(12)	63.3(13)	-3.8(10)	-5.3(10)	7.8(9)
C8	42.7(11)	45.7(11)	47.0(10)	2.2(9)	0.2(8)	-5.1(9)
C6	43.1(11)	42.6(11)	61.0(13)	-3.8(9)	-2.0(9)	3.3(9)
C3	47.7(12)	44.0(12)	68.5(14)	-9.6(10)	-4.7(10)	5.9(9)
C9	47.9(12)	52.6(13)	54.5(12)	1.6(10)	2.8(10)	-7.7(10)
C13	39.3(11)	64.5(15)	75.0(15)	-13.3(12)	-2.7(11)	0.5(10)

Table S4 Bond Lengths for exp_1g_auto.

Atom	Atom	Length/Å	Atom	Atom	Length/Å
S11	C8	1.647(2)	C4	C5	1.378(3)
O12	C4	1.365(2)	C4	C3	1.390(3)
O12	C13	1.427(3)	N10	C9	1.138(3)
N7	C1	1.420(2)	C5	C6	1.391(3)
N7	C8	1.332(3)	C2	C3	1.373(3)
C1	C2	1.390(3)	C8	C9	1.456(3)
C1	C6	1.380(3)			

Table S5 Bond Angles for exp_1g_auto.

Atom	Atom	Atom	Angle/°	Atom	Atom	Atom	Angle/°
C4	O12	C13	117.09(17)	C4	C5	C6	120.43(19)
C8	N7	C1	129.61(17)	C3	C2	C1	120.4(2)
C2	C1	N7	116.85(18)	N7	C8	S11	131.32(16)
C6	C1	N7	123.71(19)	N7	C8	C9	112.89(19)
C6	C1	C2	119.39(19)	C9	C8	S11	115.78(17)
O12	C4	C5	124.69(19)	C1	C6	C5	120.1(2)
O12	C4	C3	116.07(19)	C2	C3	C4	120.4(2)
C5	C4	C3	119.23(19)	N10	C9	C8	175.4(3)

Table S6 Torsion Angles for exp_1g_auto.

A	B	C	D	Angle/°	A	B	C	D	Angle/°
O12	C4	C5	C6	-178.7(2)	C5	C4	C3	C2	-2.6(3)
O12	C4	C3	C2	178.9(2)	C2	C1	C6	C5	-1.4(3)
N7	C1	C2	C3	179.2(2)	C8	N7	C1	C2	158.1(2)
N7	C1	C6	C5	-178.68(19)	C8	N7	C1	C6	-24.6(3)
C1	N7	C8	S11	-3.2(4)	C6	C1	C2	C3	1.8(3)
C1	N7	C8	C9	178.45(19)	C3	C4	C5	C6	3.0(3)
C1	C2	C3	C4	0.2(4)	C13	O12	C4	C5	17.2(3)
C4	C5	C6	C1	-1.0(3)	C13	O12	C4	C3	-164.4(2)

Table S7 Hydrogen Atom Coordinates (Å×10[4]) and Isotropic Displacement Parameters (Å[2]×10[3]) for exp_1g_auto.

Atom	*x*	*y*	*z*	U(eq)
H7	4142.92	5582.03	3433.52	55
H5	8901.98	5302.99	5447.9	61
H2	5120.2	8349.6	3968.35	64
H6	7175.11	3739.42	4404.29	60
H3	6842.37	9900.86	4987.58	66
H13A	10548.23	7683.46	5365.12	92
H13B	10346.23	7024.9	6548.03	92
H13C	10933.25	8949.59	6407.99	92

1D/2D NMR structural analysis of a typical imidazolidineiminodithione **18g'**

Using 5-imino-1-(4-nitrophenyl)-3-(p-tolyl)imidazolidine-2,4-dithione (**18g'**) as a representative model example, the relevant NMR spectra that were used for structural elucidation and chemical shift assignments are shown in Figure S2. Examination of the ^{13}C-CRAPT NMR spectrum (Figure S2, spectrum b) established the presence of the expected 12 signals (4 aromatic CH's, 4 phenyl quaternary carbons and 1 aromatic methyl, as well as 3 heterocyclic quaternary carbons that include 2 C=S carbons, and 1 C=N, which is consistent with 4 pairs of carbon atoms being magnetically equivalent. The most prominent feature of the ^{13}C-CRAPT NMR of **18g'** is the presence of 4 aromatic signals with highest intensity and negative phase corresponding to the 4-nitrophenyl and 4-tolyl ring CH's. This led to a quick identification and matching of the observed chemical shifts of 4-tolyl to the respective aromatic ring carbons. The 4-tolyl protons $H_{3''}$ and $H_{2''}$ (δ (ppm) 7.39 & 7.26 ppm, respectively) (Figure S2a), which correlate to the same spin system in the ^{1}H-^{1}H-gDQFCOSY spectrum (4-contour yellow square in the aromatic region, Figure S2c), were matched to $C_{3''}$ (δ 130.3), and $C_{2''}$ (δ 127.8), respectively, based on the ^{1}H-^{13}C-gHSQC spectrum (Figure S2d). Identification of the distinctive signal of $C_{5'}$-H_3 (δ 2.46), confirmed the preceding assignment of the 4-tolyl methines. In particular, the tolyl methyl shows strong correlation cross peak (^3J) in the ^{1}H-^{13}C-gHMBC spectrum (Figure S2e) with $C_{3''}$ (δ 130.3 ppm). In return, the $C_{3''}$-H triggers the identification of the quaternary $C_{1''}$ (δ 132.2 ppm) which shows a strong correlation cross peak (^3J) with $C_{3''}$-H in the ^{1}H-^{13}C-gHMBC spectrum (Figure S2e). Further, $C_{5'}$-H_3 shows strong HMBC correlation contour (^2J) with $C_{4''}$ (δ 140.6 ppm), completing the assignment and matching of the 4-toltyl ring chemical shift values. The two remaining methine aromatic signals in the HSQC with negative phases and similar intensities at δ 129.5 and 124.6 ppm were traced to the 4-nitrophenyl $H_{3'}$ (δ 8.43) and $H_{2'}$ (δ 7.77) doublets, respectively, based on strong correlation cross peaks observed in the ^{1}H-^{13}C-gHSQC spectrum (Figure S2d). Clearly, $C_{3'}$H and $C_{2'}$H comprise one spin system as further supported by ^{1}H-^{1}H-gDQFCOSY which shows them as a totally correlated system (4-contour blue square in the aromatic region (Figure S2c). The scalar coupling between the vicinal $C_{3'}$H and $C_{2'}$H is 8.0 Hz. Identification of the distinctive signal of $C_{4'}$-NO_2 (δ 147.6 ppm), confirmed the preceding assignment of the nitrophenyl methines. Particularly, $H_{2'}$ at δ 7.77 ppm shows strong correlation cross peak (^3J) in the ^{1}H-^{13}C-gHMBC spectrum (Figure S2e) with $C_{4'}$-NO_2 at δ 147.6 ppm. On the other hand, $H_{3'}$ at δ 8.43 ppm shows strong HMBC correlation contour (^3J) with $C_{1'}$ at δ 139.3 ppm, completing the assignment and matching of the nitrophenyl ring chemical shift values.

Having completely paired the chemical shift values to the protons of the two independent aromatic spin systems of the *p*-tolyl and *p*-nitrophenyl, the quaternary imine and thione carbons were identified through the ^{1}H-^{13}C-gHMBC spectrum (Figure S2e). The imine NH ($N_{5'''}$-H; ^{1}H NMR *δ* 9.57 ppm) group tethered to C_5 of the imidazolidineiminodithione ring (Figure S2) offered the only entry point to provide unambiguous matching of the observed chemical shifts appearing in the ^{13}C-CRAPT NMR to the respective heterocyclic quaternary carbons. In this regard, the ^{1}H-^{13}C-gHMBC NMR spectrum (Figure S2e) shows two strong long-range correlation contours between the N(6) proton (*δ (ppm)* 9.57 ppm) and the most deshielded thione (C=S) carbon C_4 (*δ (ppm)* 179.5 ppm) as well as C_5 (*δ (ppm)* 155.7 ppm). Interestingly, these two correlations prove the formation of the new imidazolidineiminodithione N-C_4=S and N-C_5=NH quaternary centers and is an indication of a successful heterocyclization reaction between the *N*-arylcyanothioformamide and the isothiocyanate starting material. Lastly, recognition of C_4=S prompted the identification of C_2=S as the signal at *δ* 179.6 ppm. Notably, this was the only signal that is expected not to show any HMBC long-range coupling as would be expected for such an isolated carbon far removed from all protons.

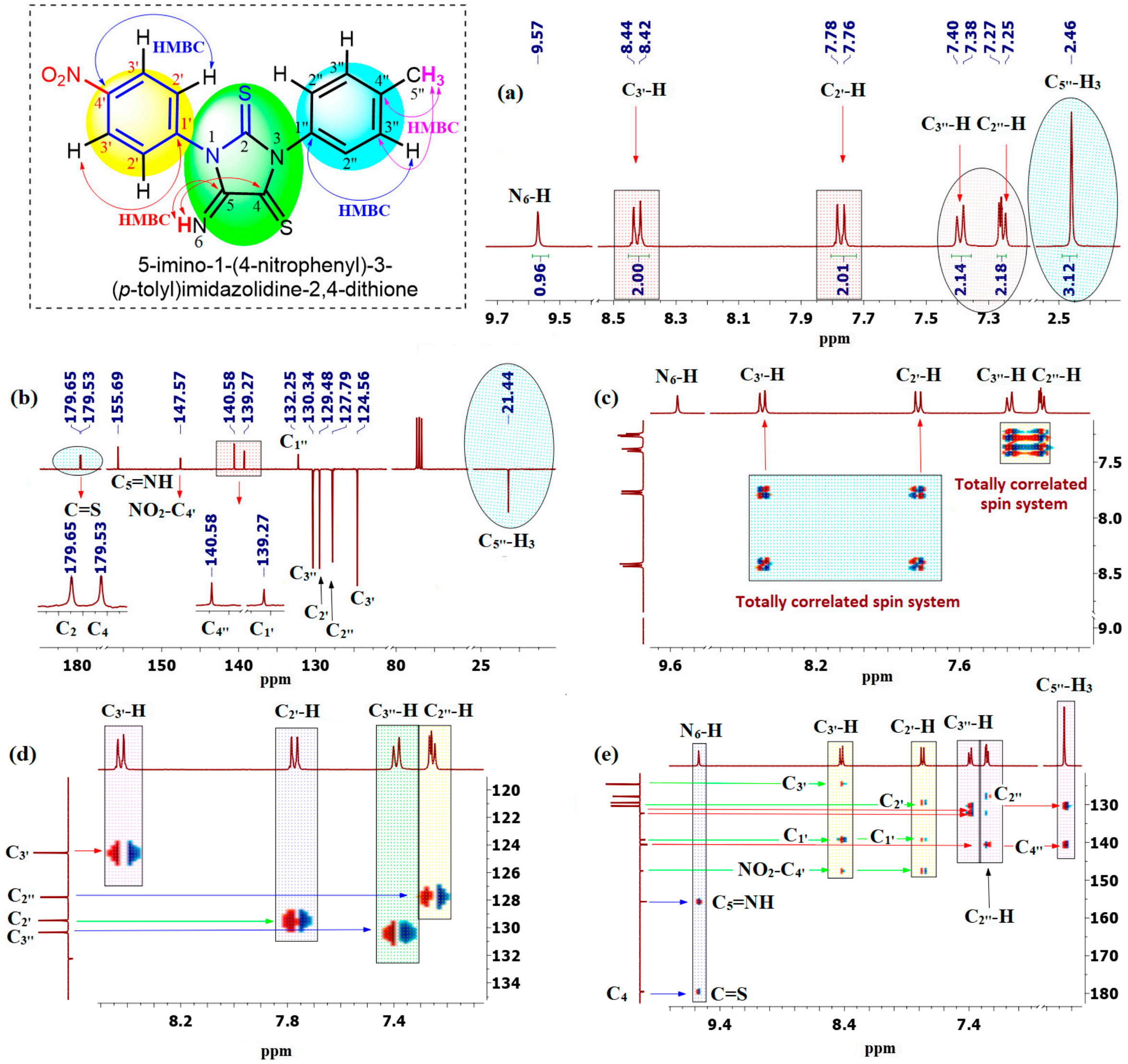

Figure S2. Truncated 1D and 2D NMR spectra of 5-imino-1-(4-nitrophenyl)-3-(p-tolyl)imidazolidine-2,4-dithione (**18g'**): (a) ^{1}H-NMR; (b) ^{13}C-CRAPT NMR; (c) ^{1}H-^{1}H-gDQFCOSY NMR; (d) ^{1}H-^{13}C-gHSQC NMR; (e) ^{1}H-^{13}C-gHMBC NMR.

Crystal-structure data for 5-imino-1-(4-nitrophenyl)-3-(p-tolyl)imidazolidine-2,4-dithione (**18g'**).

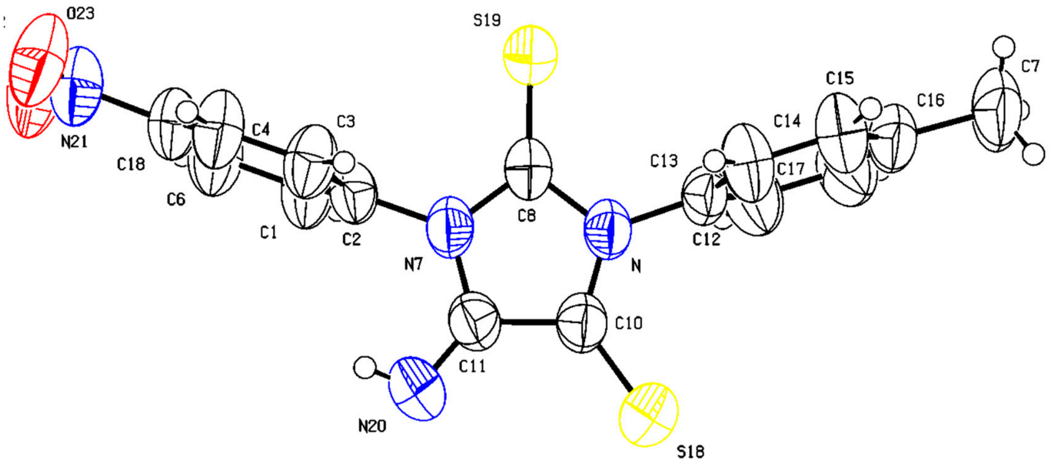

Figure S3. Thermal ellipsoid plots of (**18g'**) with ellipsoids drawn at 50% probability level (Deposition Number **2246320**). No absorption correction was applied. Selected bond distances (Å) and angles (deg), S19-C8 = 1.614(4), C2-C3 = 1.370(6), S18-C10 = 1.609(4), C2-C1 = 1.371(6), N7-C11 = 1.382(7), C18-C4 = 1.391(6), N7-C2 = 1.443(5), C18-C6 = 1.357(6), N7-C8 = 1.375(5), C3-C4 = 1.371(6), O23-N21 = 1.220(6), C13-C14 = 1.371(6), N21-C18 = 1.457(5), C13-C12 = 1.360(6), N21-O22 = 1.205(7), C14-C15 = 1.389(6), N-C10 = 1.372(6), C16-C17 = 1.375(8), N-C8 = 1.406(5), C16-C15 = 1.361(7), N-C13 = 1.442(4), C16-C7 = 1.517(7), N20-C11 = 1.256(6), C12-C17 = 1.382(7), C11-C10 = 1.507(5), C1-C6 = 1.378(6), C11-N7-C2 = 124.2(3), N-C10-C11 = 104.5(4), C8-N7-C11 = 112.3(3), C11-C10-S18 = 126.5(4), C8-N7-C2 =123.4(4), N7-C8-S19 = 127.8(3), O23-N21-C18 = 119.2(4), N7-C8-N = 105.6(3), O22- N21-O23 = 121.6(5), N-C8-S19 = 126.6(3), O22-N21-C18 = 119.1(5), C2-C3-C4 = 120.0(4), C10-N-C8 = 112.3(3), C14-C13-N = 119.4(4), C10-N-C13 = 124.7(3), C12-C13-N = 120.2(4), C8-N-C13 = 123.0(3), C12-C13-C14 = 120.4(4), N7-C11-C10 = 105.3(4), C3-C4-C18 = 118.1(4), N20-C11-N7 = 125.4(4), C13-C14- C15 = 118.7(4), N20-C11-C10 = 129.3(5), C17-C16-C7 = 120.6(5), C3-C2-N7 = 119.5(4), C15-C16-C17 = 118.3(4), C3-C2-C1 = 121.5(4), C15-C16-C7 = 121.1(6), C1-C2-N7 = 119.0(3), C13-C12-C17 = 120.1(4), C4-C18-N21 = 118.3(4), C2-C1-C6 = 118.8(4), C6-C18-N21 = 120.1(4), C18-C6-C1 = 119.9(4), C6-C18-C4 = 121.5(4), C16-C17-C12 = 120.7(5), N-C10-S18 = 129.0(3), C16-C15-C14 = 121.8(5).

exp_18g'_auto

Table S8 Crystal data and structure refinement for exp_18g'_auto.

Identification code	exp_18g'_auto
Empirical formula	$C_{16}H_{16}N_4O_4S_2$
Formula weight	392.45
Temperature/K	297.00(10)
Crystal system	orthorhombic
Space group	$Pca2_1$
a/Å	20.3332(7)

b/Å	12.9594(4)
c/Å	7.3008(3)
α/°	90
β/°	90
γ/°	90
Volume/Å^3	1923.81(12)
Z	4
ρ_{calc}g/cm^3	1.355
μ/mm^{-1}	2.765
F(000)	816.0
Crystal size/mm^3	0.6 × 0.4 × 0.3
Radiation	Cu Kα (λ = 1.54184)
2Θ range for data collection/°	11.062 to 137.998
Index ranges	-15 ≤ h ≤ 24, -15 ≤ k ≤ 15, -8 ≤ l ≤ 8
Reflections collected	9817
Independent reflections	3239 [R_{int} = 0.0296, R_{sigma} = 0.0290]
Data/restraints/parameters	3239/1/218
Goodness-of-fit on F^2	0.645
Final R indexes [I>=2σ (I)]	R_1 = 0.0447, wR_2 = 0.1384
Final R indexes [all data]	R_1 = 0.0509, wR_2 = 0.1528
Largest diff. peak/hole / e Å^{-3}	0.40/-0.16
Flack parameter	0.063(12)

Table S9 Fractional Atomic Coordinates (×10^4) and Equivalent Isotropic Displacement Parameters (Å^2×10^3) for exp_245_auto. U_{eq} is defined as 1/3 of the trace of the orthogonalised U_{IJ} tensor.

Atom	*x*	*y*	*z*	U(eq)
S19	4541.3(5)	5377.0(8)	8267.5(18)	77.8(4)
S18	4131.2(7)	7218.3(9)	1783(2)	90.0(4)
N7	3887.3(15)	4958(2)	5158(5)	66.4(8)
O23	3377(2)	321(3)	7282(9)	122.8(17)
N21	3028(2)	1070(3)	7560(8)	96.1(14)
N	4402.3(14)	6464(2)	5136(5)	63.5(8)
N20	3443(2)	4944(4)	2194(7)	91.4(12)
C11	3771.2(18)	5369(3)	3441(8)	69.8(10)
C2	3661.3(16)	3956(3)	5746(6)	63.4(9)
C18	3243(2)	2081(3)	6928(8)	76.3(11)
C10	4116.1(17)	6398(3)	3437(7)	66.1(9)
C8	4281.8(18)	5583(3)	6210(6)	61.6(9)
C3	4052(2)	3109(3)	5470(8)	78.3(12)
O22	2523(3)	1003(3)	8418(12)	160(3)

Table S9 Fractional Atomic Coordinates (×10^4) and Equivalent Isotropic Displacement Parameters (Å^2×10^3) for exp_245_auto. U_{eq} is defined as 1/3 of the trace of the orthogonalised U_{IJ} tensor.

Atom	*x*	*y*	*z*	U(eq)
C13	4791.3(18)	7324(3)	5766(6)	62.7(8)
C4	3840(2)	2151(3)	6006(9)	84.8(14)
C14	5461(2)	7302(4)	5546(9)	84.9(14)
C16	5536(3)	9005(3)	6828(8)	84.6(12)
C12	4495(2)	8154(4)	6554(10)	91.4(15)
C1	3064(2)	3877(3)	6607(9)	82.5(13)
C6	2853(2)	2919(3)	7176(9)	86.5(15)
C17	4867(3)	8994(3)	7090(11)	101.1(18)
C15	5827(3)	8148(4)	6118(9)	90.7(15)
C7	5940(4)	9943(5)	7362(11)	121(2)

Table S10 Anisotropic Displacement Parameters (Å^2×10^3) for exp_18g'_auto. The Anisotropic displacement factor exponent takes the form: $-2\pi^2[h^2a^{*2}U_{11}+2hka^*b^*U_{12}+\ldots]$.

Atom	U_{11}	U_{22}	U_{33}	U_{23}	U_{13}	U_{12}
S19	82.8(6)	64.6(6)	86.2(7)	6.7(5)	-13.7(6)	-11.2(4)
S18	104.7(9)	67.9(6)	97.4(8)	11.9(6)	-13.3(7)	-5.5(5)
N7	56.8(15)	50.8(15)	92(2)	4.0(16)	-6.6(16)	-8.5(13)
O23	119(3)	62(2)	187(5)	22(2)	26(3)	-5.9(18)
N21	90(2)	70(2)	129(4)	4(2)	13(3)	-21(2)
N	58.5(15)	46.1(15)	86(2)	-0.7(15)	-5.9(15)	-3.5(12)
N20	88(2)	87(2)	100(3)	-1(2)	-25(2)	-24(2)
C11	62.0(19)	60(2)	87(3)	0(2)	-13(2)	-5.7(15)
C2	50.1(16)	53.6(19)	86(2)	-3.6(17)	0.2(17)	-7.6(14)
C18	65(2)	63(2)	101(3)	0(2)	17(2)	-14.5(17)
C10	61.0(19)	53.0(18)	84(2)	1.2(19)	-5(2)	-2.7(14)
C8	59.5(18)	48.6(17)	77(2)	-4.9(16)	6.2(16)	-6.3(14)
C3	61(2)	59(2)	115(4)	6(2)	19(2)	-0.6(16)
O22	138(3)	96(3)	248(7)	8(4)	97(5)	-35(3)
C13	68.1(19)	41.4(15)	79(2)	-0.5(16)	-4.8(19)	-5.3(14)
C4	72(2)	53(2)	129(4)	0(2)	20(3)	-4.2(17)
C14	76(2)	66(2)	113(4)	-25(3)	16(3)	-12(2)
C16	108(3)	52(2)	94(3)	-1(2)	-2(3)	-19(2)
C12	74(2)	69(2)	132(5)	-26(3)	-6(3)	9.6(19)
C1	61(2)	58(2)	128(4)	-3(2)	18(3)	-2.9(16)
C6	61(2)	73(2)	125(4)	1(3)	28(2)	-10.6(18)
C17	109(4)	57(2)	137(5)	-26(3)	-23(4)	12(2)

Table S10 Anisotropic Displacement Parameters (Å^2×10^3) for exp_18g'_auto. The Anisotropic displacement factor exponent takes the form: $-2\pi^2[h^2a^{*2}U_{11}+2hka^*b^*U_{12}+\ldots]$.

Atom	U_{11}	U_{22}	U_{33}	U_{23}	U_{13}	U_{12}
C15	81(3)	73(3)	118(4)	-20(3)	15(3)	-22(2)
C7	164(6)	62(3)	135(5)	-9(3)	-4(5)	-40(3)

Table S11 Bond Lengths for exp_18g'_auto.

Atom	Atom	Length/Å	Atom	Atom	Length/Å
S19	C8	1.614(4)	C2	C3	1.370(6)
S18	C10	1.609(4)	C2	C1	1.371(6)
N7	C11	1.382(7)	C18	C4	1.391(6)
N7	C2	1.443(5)	C18	C6	1.357(6)
N7	C8	1.375(5)	C3	C4	1.371(6)
O23	N21	1.220(6)	C13	C14	1.371(6)
N21	C18	1.457(5)	C13	C12	1.360(6)
N21	O22	1.205(7)	C14	C15	1.389(6)
N	C10	1.372(6)	C16	C17	1.375(8)
N	C8	1.406(5)	C16	C15	1.361(7)
N	C13	1.442(4)	C16	C7	1.517(7)
N20	C11	1.256(6)	C12	C17	1.382(7)
C11	C10	1.507(5)	C1	C6	1.378(6)

Table S12 Bond Angles for exp_18g'_auto.

Atom	Atom	Atom	Angle/°	Atom	Atom	Atom	Angle/°
C11	N7	C2	124.2(3)	N	C10	C11	104.5(4)
C8	N7	C11	112.3(3)	C11	C10	S18	126.5(4)
C8	N7	C2	123.4(4)	N7	C8	S19	127.8(3)
O23	N21	C18	119.2(4)	N7	C8	N	105.6(3)
O22	N21	O23	121.6(5)	N	C8	S19	126.6(3)
O22	N21	C18	119.1(5)	C2	C3	C4	120.0(4)
C10	N	C8	112.3(3)	C14	C13	N	119.4(4)
C10	N	C13	124.7(3)	C12	C13	N	120.2(4)
C8	N	C13	123.0(3)	C12	C13	C14	120.4(4)
N7	C11	C10	105.3(4)	C3	C4	C18	118.1(4)
N20	C11	N7	125.4(4)	C13	C14	C15	118.7(4)
N20	C11	C10	129.3(5)	C17	C16	C7	120.6(5)

Table S12 Bond Angles for exp_18g'_auto.

Atom	Atom	Atom	Angle/°	Atom	Atom	Atom	Angle/°
C3	C2	N7	119.5(4)	C15	C16	C17	118.3(4)
C3	C2	C1	121.5(4)	C15	C16	C7	121.1(6)
C1	C2	N7	119.0(3)	C13	C12	C17	120.1(4)
C4	C18	N21	118.3(4)	C2	C1	C6	118.8(4)
C6	C18	N21	120.1(4)	C18	C6	C1	119.9(4)
C6	C18	C4	121.5(4)	C16	C17	C12	120.7(5)
N	C10	S18	129.0(3)	C16	C15	C14	121.8(5)

Table S13 Torsion Angles for exp_18g'_auto.

A	B	C	D	Angle/°	A	B	C	D	Angle/°
N7	C11	C10	S18	-178.9(3)	C8	N7	C11	N20	177.9(5)
N7	C11	C10	N	0.9(4)	C8	N7	C11	C10	-2.2(5)
N7	C2	C3	C4	-179.3(5)	C8	N7	C2	C3	-85.7(6)
N7	C2	C1	C6	-179.6(5)	C8	N7	C2	C1	92.9(5)
O23	N21	C18	C4	0.8(9)	C8	N	C10	S18	-179.6(3)
O23	N21	C18	C6	178.1(6)	C8	N	C10	C11	0.6(4)
N21	C18	C4	C3	-177.8(5)	C8	N	C13	C14	86.4(6)
N21	C18	C6	C1	178.8(5)	C8	N	C13	C12	-94.3(5)
N	C13	C14	C15	178.4(5)	C3	C2	C1	C6	-1.0(9)
N	C13	C12	C17	-177.4(5)	O22	N21	C18	C4	177.4(8)
N20	C11	C10	S18	1.1(7)	O22	N21	C18	C6	-5.3(9)
N20	C11	C10	N	-179.2(5)	C13	N	C10	S18	-0.4(6)
C11	N7	C2	C3	89.8(5)	C13	N	C10	C11	179.8(3)
C11	N7	C2	C1	-91.6(6)	C13	N	C8	S19	0.3(5)
C11	N7	C8	S19	-178.9(3)	C13	N	C8	N7	178.9(3)
C11	N7	C8	N	2.5(5)	C13	C14	C15	C16	-2.3(10)
C2	N7	C11	N20	2.0(7)	C13	C12	C17	C16	0.3(11)
C2	N7	C11	C10	-178.1(3)	C4	C18	C6	C1	-4.0(10)
C2	N7	C8	S19	-2.9(6)	C14	C13	C12	C17	1.9(9)
C2	N7	C8	N	178.5(3)	C12	C13	C14	C15	-0.9(9)
C2	C3	C4	C18	-3.9(9)	C1	C2	C3	C4	2.1(8)
C2	C1	C6	C18	1.9(9)	C6	C18	C4	C3	4.9(9)
C10	N	C8	S19	179.5(3)	C17	C16	C15	C14	4.4(10)
C10	N	C8	N7	-1.9(4)	C15	C16	C17	C12	-3.4(10)
C10	N	C13	C14	-92.8(6)	C7	C16	C17	C12	178.0(6)
C10	N	C13	C12	86.5(6)	C7	C16	C15	C14	-177.0(6)

Table S14 Hydrogen Atom Coordinates (Å×10^4) and Isotropic Displacement Parameters (Å^2×10^3) for exp_18g'_auto.

**Atom**	***x***	***y***	***z***	**U(eq)**
H20	3422.24	4321.8	2388.16	137
H3	4461.77	3184.21	4918.14	94
H4	4088.81	1564.71	5760.53	102
H14	5665.83	6732.21	5024.26	102
H12	4042.41	8156.02	6732.23	110
H1	2806.5	4459.8	6803.11	99
H6	2443.42	2846.67	7729.55	104
H17	4661.81	9557.49	7634.49	121
H15	6282.27	8128.23	6012.65	109
H7A	6119.84	10255.62	6280.72	181
H7B	6291.08	9734.68	8159.07	181
H7C	5663.86	10431.18	7983.91	181

Table S15 Solvent masks information for exp_18g'_auto.

**Number**	**X**	**Y**	**Z**	**Volume**	**Electron count**	**Content**
1	0.250	0.760	-0.997	178.2	38.2	4 H2O
2	0.750	0.240	-0.855	178.2	38.1	4 H2O

Crystal-structure data for 5-imino-1-(4-nitrophenyl)-3-phenylimidazolidine-2,4-dithione (**18h**).

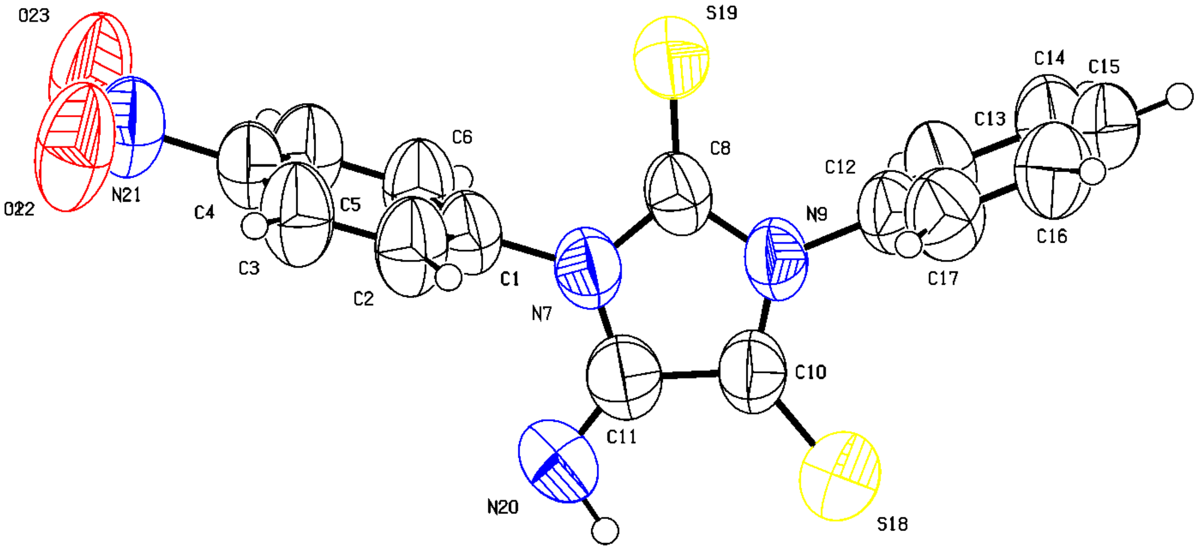

Figure S4. Thermal ellipsoid plots of (**18h**) with ellipsoids drawn at 50% probability level (Deposition Number **2267721**). No absorption correction was applied. Selected bond distances (Å) and angles (deg), S(19)-C(8) = 1.618(5), C(1)-C(6) = 1.359(6), S(18)-C(10) = 1.609(5), C(1)-C(2) = 1.372(5), N(7)-C(11) = 1.383(7), C(6)-C(5) = 1.375(6), N(7)-C(1) = 1.448(5), C(4)-C(5) = 1.345(6), N(7)-C(8) = 1.378(5), C(4)-C(3) = 1.371(5), N(21)-O(23) = 1.205(5), C(2)-C(3) = 1.386(6), N(21)-O(22) = 1.204(5), C(12)-C(17) = 1.374(6), N(21)-C(4) = 1.485(5), C(12)-C(13) = 1.336(6), N(9)-C(10) = 1.380(7), C(15)-C(16) = 1.350(8), N(9)-C(8) = 1.386(5), C(15)-C(14) = 1.362(8), N(9)-C(12) = 1.453(5), C(17)-C(16) = 1.374(7), C(11)-C(10) = 1.499(6), C(13)-C(14) = 1.394(7), C(11)-N(20) = 1.264(6), C(1)-N(7)-C(11) = 124.9(3), C(11)-C(10)-N(9) = 104.3(4), C(8)-N(7)-C(11) = 112.2(3), C(5)-C(4)-N(21) = 118.6(4), C(8)-N(7)-C(1) = 122.9(4), C(3)-C(4)-N(21) = 117.7(3), O(22)-N(21)-O(23) = 123.5(4), C(3)-C(4)-C(5) = 123.6(4), C(4)-N(21)-O(23) = 117.5(3), C(4)-C(5)-C(6) = 119.0(4), C(4)-N(21)-O(22) = 119.0(4), C(3)-C(2)-C(1) = 119.2(4), C(8)-N(9)-C(10) = 112.7(3), C(2)-C(3)-C(4) = 117.1(3), C(12)-N(9)-C(10) = 124.0(4), N(7)-C(8)-S(19) = 127.4(3), C(12)-N(9)-C(8) = 123.2(4), N(9)-C(8)-S(19) = 127.0(3), C(10)-C(11)-N(7) = 105.2(4), N(9)-C(8)-N(7) = 105.6(4), N(20)-C(11)-N(7) = 124.6(4), C(17)-C(12)-N(9) = 118.3(4), N(20)-C(11)-C(10) = 130.2(5), C(13)-C(12)-N(9) = 119.5(4), C(6)-C(1)-N(7) = 118.8(3), C(13)-C(12)-C(17) = 122.2(4), C(2)-C(1)-N(7) = 119.0(3), C(14)-C(15)-C(16) = 120.7(4), C(2)-C(1)-C(6) = 122.1(4), C(16)-C(17)-C(12) = 117.9(4), C(5)-C(6)-C(1) = 118.8(4), C(14)-C(13)-C(12) = 119.1(5), N(9)-C(10)-S(18) = 129.0(3), C(17)-C(16)-C(15) = 120.8(5), C(11)-C(10)-S(18) = 126.7(4), C(13)-C(14)-C(15) = 119.2(5)

exp_18h_auto

Table S16 Crystal data and structure refinement for exp_18h_auto.

Identification code	exp_18h_auto

Empirical formula	$C_{16}H_{10}N_3O_2S_2$
Formula weight	340.409
Temperature/K	297.00(10)
Crystal system	orthorhombic
Space group	$Pca2_1$
a/Å	20.7656(17)
b/Å	11.9873(11)
c/Å	7.2772(8)
α/°	90
β/°	90
γ/°	90
Volume/Å^3	1811.5(3)
Z	4
ρ_{calc}g/cm^3	1.248
μ/mm^{-1}	2.763
F(000)	704.8
Crystal size/mm^3	0.5 × 0.3 × 0.2
Radiation	Cu Kα (λ = 1.54184)
2Θ range for data collection/°	7.38 to 136.98
Index ranges	-26 ≤ h ≤ 19, -14 ≤ k ≤ 14, -7 ≤ l ≤ 8
Reflections collected	9449
Independent reflections	2568 [R_{int} = 0.0367, R_{sigma} = 0.0352]
Data/restraints/parameters	2568/1/208
Goodness-of-fit on F^2	1.000
Final R indexes [I>=2σ (I)]	R_1 = 0.0449, wR_2 = 0.1285
Final R indexes [all data]	R_1 = 0.0604, wR_2 = 0.1438
Largest diff. peak/hole / e Å^{-3}	0.15/-0.17
Flack parameter	-0.034(17)

Table S17 Fractional Atomic Coordinates (×10^4) and Equivalent Isotropic Displacement Parameters (Å^2×10^3) for exp_18h_auto. U_{eq} is defined as 1/3 of the trace of the orthogonalised U_{IJ} tensor.

Atom	*x*	*y*	*z*	U(eq)
S$^{(19)}$	-4559.1(5)	-4555.6(9)	-1826.2(19)	100.9(4)
S$^{(18)}$	-4202.4(8)	-2444.2(11)	-818h(2)	124.0(5)
N$^{(7)}$	-3905.6(14)	-4912(3)	-4960(6)	85.6(9)
N$^{(21)}$	-2924.7(18)	-9044(3)	-2643(7)	109.1(14)
N$^{(9)}$	-4441.8(14)	-3328(2)	-4903(6)	82.7(9)
O$^{(23)}$	-3287.3(18)	-9829(3)	-2663(8)	158(2)
C$^{(11)}$	-3809.0(19)	-4443(4)	-6673(8)	92.9(12)
O$^{(22)}$	-2376.4(17)	-9100(3)	-2114(8)	153.4(19)

Table S17 Fractional Atomic Coordinates (×10^4) and Equivalent Isotropic Displacement Parameters (Å^2×10^3) for exp_18h_auto. U_{eq} is defined as 1/3 of the trace of the orthogonalised U_{IJ} tensor.

Atom	*x*	*y*	*z*	U(eq)
C(1)	-3648.5(16)	-5977(3)	-4372(6)	81.8(11)
C(6)	-4015.6(19)	-6906(3)	-4585(8)	95.6(14)
C(10)	-4167.3(18)	-3358(3)	-6629(7)	88.7(11)
N(20)	-3468.5(19)	-4870(4)	-7930(7)	117.6(14)
C(4)	-3180.8(17)	-7951(3)	-3265(7)	86.3(11)
C(5)	-3771.4(19)	-7917(4)	-4023(8)	100.7(15)
C(2)	-3042.0(17)	-6016(3)	-3626(8)	101.3(15)
C(3)	-2789.9(17)	-7036(3)	-3088(9)	105.8(16)
C(8)	-4300.7(16)	-4268(3)	-3869(7)	81.1(11)
C(12)	-4857.1(19)	-2435(3)	-4254(7)	83.9(10)
C(15)	-5616(3)	-752(4)	-3036(9)	115.7(17)
C(17)	-4578(2)	-1545(4)	-3370(9)	107.4(16)
C(13)	-5492(2)	-2507(4)	-4517(8)	100.9(14)
C(16)	-4973(3)	-701(4)	-2770(9)	122.6(18)
C(14)	-5886(3)	-1640(5)	-3914(9)	119.2(18)

Table S18 Anisotropic Displacement Parameters (Å^2×10^3) for exp_18h_auto. The Anisotropic displacement factor exponent takes the form: $-2\pi^2[h^2a^{*2}U_{11}+2hka^*b^*U_{12}+\ldots]$.

Atom	U_{11}	U_{22}	U_{33}	U_{12}	U_{13}	U_{23}
S(19)	101.3(6)	84.7(6)	116.5(9)	10.5(5)	16.7(7)	5.3(7)
S(18)	145.5(10)	93.4(8)	133.0(11)	14.7(7)	21.5(10)	14.7(8)
N(7)	73.1(16)	71.8(17)	112(2)	10.4(15)	8.5(19)	1(2)
N(21)	91(2)	81(2)	155(4)	10.7(19)	-13(2)	5(2)
N(9)	79.4(16)	56.7(16)	112(3)	7.6(13)	6.7(19)	-4.8(17)
O(23)	119(2)	83(2)	273(7)	-7.8(19)	-23(3)	28(3)
C(11)	81(2)	80(2)	119(4)	-0.1(19)	6(3)	-3(3)
O(22)	101(2)	105(2)	255(6)	12.2(18)	-49(3)	27(3)
C(1)	65.0(17)	68(2)	113(3)	8.5(15)	0.8(19)	-5(2)
C(6)	77.6(19)	70(2)	140(4)	-0.5(18)	-14(2)	-6(3)
C(10)	82(2)	67(2)	117(3)	7.1(16)	7(3)	2(3)
N(20)	110(2)	113(3)	130(3)	27(2)	39(3)	1(3)
C(4)	77.2(19)	64.4(19)	117(3)	8.4(16)	-3(2)	-2(2)
C(5)	78(2)	75(2)	149(4)	-2.3(18)	-16(3)	-1(3)
C(2)	76(2)	71(2)	157(5)	-3.5(17)	-14(3)	-5(3)
C(3)	69.9(18)	82(2)	166(5)	2.0(18)	-20(3)	-9(3)
C(8)	62.7(15)	69(2)	112(3)	3.9(16)	1(2)	-8(2)

Table S18 Anisotropic Displacement Parameters (Å^2×10^3) for exp_18h_auto. The Anisotropic displacement factor exponent takes the form: $-2\pi^2[h^2a^{*2}U_{11}+2hka^*b^*U_{12}+\ldots]$.

Atom	U_{11}	U_{22}	U_{33}	U_{12}	U_{13}	U_{23}
C(12)	92(2)	59.2(18)	101(3)	5.2(18)	2(2)	-9.1(19)
C(15)	152(5)	75(3)	120(4)	33(3)	30(4)	5(3)
C(17)	107(3)	74(2)	141(5)	-10(2)	9(3)	-15(3)
C(13)	86(2)	89(3)	127(4)	17(2)	-1(3)	-17(3)
C(16)	162(5)	64(2)	143(5)	1(3)	20(4)	-19(3)
C(14)	111(3)	99(3)	148(5)	31(3)	3(4)	-4(3)

Table S19 Bond Lengths for exp_18h_auto.

Atom	Atom	Length/Å	Atom	Atom	Length/Å
S(19)	C(8)	1.618(5)	C(1)	C(6)	1.359(6)
S(18)	C(10)	1.609(5)	C(1)	C(2)	1.372(5)
N(7)	C(11)	1.383(7)	C(6)	C(5)	1.375(6)
N(7)	C(1)	1.448(5)	C(4)	C(5)	1.345(6)
N(7)	C(8)	1.378(5)	C(4)	C(3)	1.371(5)
N(21)	O(23)	1.205(5)	C(2)	C(3)	1.386(6)
N(21)	O(22)	1.204(5)	C(12)	C(17)	1.374(6)
N(21)	C(4)	1.485(5)	C(12)	C(13)	1.336(6)
N(9)	C(10)	1.380(7)	C(15)	C(16)	1.350(8)
N(9)	C(8)	1.386(5)	C(15)	C(14)	1.362(8)
N(9)	C(12)	1.453(5)	C(17)	C(16)	1.374(7)
C(11)	C(10)	1.499(6)	C(13)	C(14)	1.394(7)
C(11)	N(20)	1.264(6)			

Table S20 Bond Angles for exp_18h_auto.

Atom	Atom	Atom	Angle/°	Atom	Atom	Atom	Angle/°
C(1)	N(7)	C(11)	124.9(3)	C(11)	C(10)	N(9)	104.3(4)
C(8)	N(7)	C(11)	112.2(3)	C(5)	C(4)	N(21)	118.6(4)
C(8)	N(7)	C(1)	122.9(4)	C(3)	C(4)	N(21)	117.7(3)
O(22)	N(21)	O(23)	123.5(4)	C(3)	C(4)	C(5)	123.6(4)
C(4)	N(21)	O(23)	117.5(3)	C(4)	C(5)	C(6)	119.0(4)
C(4)	N(21)	O(22)	119.0(4)	C(3)	C(2)	C(1)	119.2(4)
C(8)	N(9)	C(10)	112.7(3)	C(2)	C(3)	C(4)	117.1(3)
C(12)	N(9)	C(10)	124.0(4)	N(7)	C(8)	S(19)	127.4(3)
C(12)	N(9)	C(8)	123.2(4)	N(9)	C(8)	S(19)	127.0(3)
C(10)	C(11)	N(7)	105.2(4)	N(9)	C(8)	N(7)	105.6(4)

Table S20 Bond Angles for exp_18h_auto.

Atom	Atom	Atom	Angle/°	Atom	Atom	Atom	Angle/°
$N^{(20)}$	$C^{(11)}$	$N^{(7)}$	124.6(4)	$C^{(17)}$	$C^{(12)}$	$N^{(9)}$	118.3(4)
$N^{(20)}$	$C^{(11)}$	$C^{(10)}$	130.2(5)	$C^{(13)}$	$C^{(12)}$	$N^{(9)}$	119.5(4)
$C^{(6)}$	$C^{(1)}$	$N^{(7)}$	118.8(3)	$C^{(13)}$	$C^{(12)}$	$C^{(17)}$	122.2(4)
$C^{(2)}$	$C^{(1)}$	$N^{(7)}$	119.0(3)	$C^{(14)}$	$C^{(15)}$	$C^{(16)}$	120.7(4)
$C^{(2)}$	$C^{(1)}$	$C^{(6)}$	122.1(4)	$C^{(16)}$	$C^{(17)}$	$C^{(12)}$	117.9(4)
$C^{(5)}$	$C^{(6)}$	$C^{(1)}$	118.8(4)	$C^{(14)}$	$C^{(13)}$	$C^{(12)}$	119.1(5)
$N^{(9)}$	$C^{(10)}$	$S^{(18)}$	129.0(3)	$C^{(17)}$	$C^{(16)}$	$C^{(15)}$	120.8(5)
$C^{(11)}$	$C^{(10)}$	$S^{(18)}$	126.7(4)	$C^{(13)}$	$C^{(14)}$	$C^{(15)}$	119.2(5)

Table S21 Torsion Angles for exp_18h_auto.

A	B	C	D	Angle/°	A	B	C	D	Angle/°
$S^{(19)}$	$C^{(8)}$	$N^{(7)}$	$C^{(11)}$	176.5(4)	$N^{(7)}$	$C^{(8)}$	$N^{(9)}$	$C^{(12)}$	179.2(3)
$S^{(19)}$	$C^{(8)}$	$N^{(7)}$	$C^{(1)}$	-1.2(4)	$N^{(21)}$	$C^{(4)}$	$C^{(5)}$	$C^{(6)}$	179.3(5)
$S^{(19)}$	$C^{(8)}$	$N^{(9)}$	$C^{(10)}$	-177.3(3)	$N^{(21)}$	$C^{(4)}$	$C^{(3)}$	$C^{(2)}$	-178.2(5)
$S^{(19)}$	$C^{(8)}$	$N^{(9)}$	$C^{(12)}$	0.3(4)	$N^{(9)}$	$C^{(10)}$	$C^{(11)}$	$N^{(20)}$	-179.1(3)
$S^{(18)}$	$C^{(10)}$	$N^{(9)}$	$C^{(8)}$	-179.4(4)	$N^{(9)}$	$C^{(12)}$	$C^{(17)}$	$C^{(16)}$	179.6(5)
$S^{(18)}$	$C^{(10)}$	$N^{(9)}$	$C^{(12)}$	3.1(5)	$N^{(9)}$	$C^{(12)}$	$C^{(13)}$	$C^{(14)}$	-179.1(5)
$S^{(18)}$	$C^{(10)}$	$C^{(11)}$	$N^{(7)}$	178.0(4)	$C^{(1)}$	$C^{(6)}$	$C^{(5)}$	$C^{(4)}$	0.7(6)
$S^{(18)}$	$C^{(10)}$	$C^{(11)}$	$N^{(20)}$	-0.0(5)	$C^{(1)}$	$C^{(2)}$	$C^{(3)}$	$C^{(4)}$	-2.9(6)
$N^{(7)}$	$C^{(11)}$	$C^{(10)}$	$N^{(9)}$	-1.1(3)	$C^{(6)}$	$C^{(5)}$	$C^{(4)}$	$C^{(3)}$	-3.0(7)
$N^{(7)}$	$C^{(1)}$	$C^{(6)}$	$C^{(5)}$	-179.9(4)	$C^{(12)}$	$C^{(17)}$	$C^{(16)}$	$C^{(15)}$	0.4(7)
$N^{(7)}$	$C^{(1)}$	$C^{(2)}$	$C^{(3)}$	-179.0(5)	$C^{(12)}$	$C^{(13)}$	$C^{(14)}$	$C^{(15)}$	-1.4(7)
$N^{(7)}$	$C^{(8)}$	$N^{(9)}$	$C^{(10)}$	1.6(4)					

Table S22 Hydrogen Atom Coordinates (Å×10^4) and Isotropic Displacement Parameters (Å^2×10^3) for exp_18h_auto.

Atom	*x*	*y*	*z*	U(eq)
$H^{(6)}$	-4424.5(19)	-6860(3)	-5101(8)	114.8(16)
$H^{(20)}$	-3474.0(19)	-4374(4)	-9034(7)	176(2)
$H^{(5)}$	-4010.8(19)	-8566(4)	-4165(8)	120.9(18)
$H^{(2)}$	-2802.9(17)	-5365(3)	-3482(8)	121.5(19)
$H^{(3)}$	-2373.6(17)	-7096(3)	-2628(9)	127.0(19)
$H^{(15)}$	-5877(3)	-176(4)	-2615(9)	139(2)
$H^{(17)}$	-4135(2)	-1515(4)	-3182(9)	128.8(19)
$H^{(13)}$	-5669(2)	-3127(4)	-5096(8)	121.1(17)
$H^{(16)}$	-4796(3)	-87(4)	-2174(9)	147(2)

Table S22 Hydrogen Atom Coordinates (Å×10^4) and Isotropic Displacement Parameters (Å^2×10^3) for exp_18h_auto.

Atom	*x*	*y*	*z*	U(eq)
$H^{(14)}$	-6329(3)	-1667(5)	-4108(9)	143(2)

Table S23 Solvent masks information for exp_18h_auto.

Number	X	Y	Z	Volume	Electron count	Content
1	0.250	0.235	-0.727	163.2	39.24	H_2O
2	0.750	0.765	-0.453	163.2	39.44	H_2O

The X-ray structure of 5-imino-1-(4-nitrophenyl)-3-phenylimidazolidine-2,4-dithione (**18h**) reveals a compound with a similar molecular framework to its analog **18g**' (Figure S4) (see supporting information S378-S383 for compete crystal data).. In this structure, the imidazolidine ring is substituted with a phenyl group and a 4-nitrophenyl group, along with two exocyclic sulfur atoms and an imine function. Comparing the two structures, we observe similarities and differences in the bond lengths and angles, providing insights into the structural features and potential reactivity of this compound. The N(7)-C(8) and N(9)-C(8) bond lengths are found to be 1.378(5) Å and 1.386(5) Å, respectively, which are shorter than the typical C-N single bond length. This phenomenon is attributed to the strong resonance interactions involving N(7) and the attached 4-nitrophenyl, phenyl, and C=S or imine groups. The shorter N(7)-C(8) bond can be attributed to its conjugation with the more electronegative 4-nitrophenyl and imine groups. Similar to the previous structure, the nitro group in this compound also adopts a planar conformation relative to the phenyl group, indicating a favorable arrangement for resonance and mesomeric effects. This planarity facilitates electron delocalization and contributes to the overall stability of the molecule. The dihedral angles N(7)-N(11)-C(10)-N(9) (-1.1(3)°) and N(7)-C(8)-N(9)-C(10) (1.6(4)°) suggest a near planar conformation for the imidazolidine ring. However, the 4-nitrophenyl and phenyl rings show an orthogonal orientation with respect to the plane of the 5-membered ring. This arrangement minimizes non-bonded interactions between the vicinal N-aryl groups and the C=S(18)/C=N(20) functionalities. To further alleviate repulsive forces, the angles around the heterocyclic atoms deviate from their ideal values. For instance, the observed angles for C(8)-N(7)-C(1) (122.9(4)°), C(11)-N(7)-C(1) (124.9(3)°), C(8)-N(9)-C(12) (123.2(4)°), C(10)-N(9)-C(12) (124.0(4)°), N(7)-C(8)-S(19) (127.4(3)°), N(9)-C(8)-S(19) (127.0(3)°), C(11)-C(10)-S(18) (126.7(4)°), and N-C(10)-S(18) (129.0(3)°) deviate significantly from their expected values based on ideal geometries. The C(11)=N(20) group exhibits a short bond length of 1.264(6) Å and a trigonal planar geometry, as reflected in the angles C(10)-C(11)-N(20) = 130.2(5)° and N(7)-C(11)-N(20) = 124.6(4)°. Noteworthy, the complimentary internal angle C(10)-C(11)-N(7) = 105.2(4) and the N(9)-C(8)-N(7) = 105.6(4), are very strained to accomodate larger external angles needed to relieve steric interactions between the *N*-substituents and exocyclic groups. The imine adopts a *E* geometry, contrary to the observed configuration in **18g'**, with the H-atom oriented away from the aromatic group. Due to the small and exchangeable nature of the imine proton, indicated by its broad absorption in the ^{1}H NMR spectrum, steric factors are negligible, allowing for the possibility of either configuration. This observation of the exchangeable nature of the N-H

has significant implications for its reactivity. The imine is expected to be highly prone to deprotonation, acting as a reactive nucleophile.

Computational Details

S385. Relaxed Geometry, Atomic Charges and Bond-Order Analysis of anion 1

The *N*-arylcyanothioformamide anion (anion of **1**) was investigated computationally at the B3LYP level in DMF as implicit solvent. Briefly, the relaxed geometry of anion **1** (see Supplementary "1.xyz") features the following key geometrical parameters (refer to Supplementary Table S24 for the atom indices): bond N13C14 (1.1539 Å), angle C14C3N1 (113.161°), angle C3N1C2 (121.055°), bond C3N1 (1.2956 Å), and bond C3S0 (1.71492 Å). The calculated Mayer bond orders (BO) are N13C14 (3.0564) consistent with a triple bond for the nitrile group; meanwhile S0C3 (1.4099) and N1C3 (1.6587) bonds are both midway between double and single bond, which is expected for a thioamide plane owing to its extended π character and resonance forms. The bond N1C2 (BO=1.0583) is consistent with a single bond. The calculated atomic charges (in units of *e*) are included in the Table S24. All definitions place a negative charge on sulfur S0 atom. Since N1 is deprotonated it has anionic character. The anionic character of N1 atom is best described by Bader definition (-1.17*e*), with Löwdin charge giving (erroneously) the opposite sign. Another important reactive site is C14 which is assigned a positive charge according to NPA, Bader and ADCH definitions. Most charge definitions also predict the nitrile N13 and C14 atoms being negative and positive, respectively, and the C3 atom being positive. Finally, anion **1** is very polar and with the origin of the dipole at the center of mass (the system is anionic so its electric dipole is origin-dependent) the magnitude is 10.28 Debye.

Table S24. Calculated atomic charges for phenyl cyanothioformamide **1** computed at the B3LYP-D4/def2-TZVP level of theory in implicit DMF as solvent. The total charge for each definition adds up to -1 for this anion. Atom indices are shown in the ball-and-stick model with atom color code: yellow=S, grey=C, blue=N, and white=H.

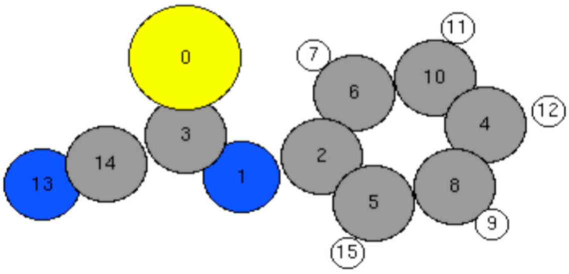

Atom labels in phenylcyano-thioformamide anion (compound 1)	Mulliken	Löwdin	NPA	Bader	ADCH
S0	-0.578	-0.028	-0.399	-0.314	-0.643
N1	-0.285	0.108	-0.490	-1.170	-0.531
C2	0.247	-0.233	0.123	0.351	0.082
C3	0.218	-0.622	-0.039	0.526	0.527
C4	-0.178	-0.170	-0.251	-0.060	-0.157
C5	-0.283	-0.132	-0.247	-0.055	-0.114

C6	-0.264	-0.131	-0.239	-0.047	-0.293
H7	0.129	0.150	0.223	0.053	0.155
C8	-0.127	-0.156	-0.214	-0.050	-0.166
H9	0.143	0.149	0.220	0.047	0.143
C10	-0.131	-0.156	-0.215	-0.054	-0.041
H11	0.144	0.149	0.220	0.049	0.142
H12	0.144	0.147	0.220	0.044	0.133
N13	-0.111	-0.024	-0.427	-1.307	-0.419
C14	-0.193	-0.201	0.294	0.937	0.059
H15	0.126	0.149	0.221	0.050	0.125

S386. Relaxed Geometry, Atomic Charges and Bond-Order Analysis of 16
We now continue with the computational characterization of the other reactant species, compound **16** (phenyl isothiocyanate or PITC) in its neutral form. Its relaxed geometry, found in Supplementary “16.xyz”, features an isothiocyanate group (R-N=C=S) of nearly linear geometry. The computed bond angles for C3N1C2 and N1C2S0 in **16** are 156.8° and 176.5°, respectively. The calculated Mayer bond orders (BO) for this group (Ph-NCS) are 1.983 and 2.227 for the C2S0 and N1C2 bonds, respectively, consistent with double bonds but with the latter being stronger than the former. This correlates with the optimized bond lengths of 1.586 and 1.181 Å for the C2S0 and N1C2 bonds, respectively. Finally, the C3-N1 bond has a distance of 1.380 Å and a bond order of 1.002, consistent with a single C-C bond. This compound is highly polar with a calculated dipole moment of 4.542 Debye directed along the N=C=S group. The calculated stretching frequency (scaled down by the recommended B3LYP factor of 0.959) of the R-N=C=S group appears at about 899.91 cm^{-1} and is strongly coupled with the phenyl ring breathing modes. From Table S25, most charge definitions (except the Löwdin method which seems to predict erroneously the opposite sign for all atomic charges) predict that N1 atom holds a negative partial charge while C2 atom is positive. These two atoms are the most relevant sites for the reaction to be described below. All phenyl carbons are predicted consistently to be negative.

Table S25. Calculated atomic charges for phenyl isothiocyanate (PITC) **16** computed at the B3LYP-D4/def2-TZVP level of theory in implicit DMF as solvent. Atom indices are shown in the ball-and-stick model with atom color code: yellow=S, grey=C, blue=N, and white=H.

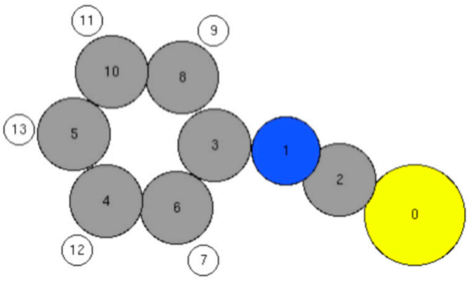

Atom labels in phenyl isothiocyanate	Mulliken	Löwdin	NPA	Bader	ADCH

(compound 16)					
S0	-0.259	0.278	-0.071	0.337	-0.237
N1	-0.029	0.274	-0.378	-1.449	-0.158
C2	0.090	-0.534	0.229	0.470	0.222
C3	0.254	-0.203	0.095	0.424	0.071
C4	-0.114	-0.133	-0.204	-0.033	-0.124
C5	-0.154	-0.136	-0.212	-0.040	-0.130
C6	-0.231	-0.097	-0.209	-0.019	-0.127
H7	0.165	0.160	0.236	0.081	0.151
C8	-0.231	-0.100	-0.204	-0.017	-0.135
H9	0.156	0.159	0.236	0.080	0.148
C10	-0.112	-0.133	-0.206	-0.033	-0.123
H11	0.154	0.155	0.230	0.067	0.149
H12	0.156	0.156	0.230	0.068	0.149
H13	0.154	0.153	0.228	0.064	0.146

S387. Molecular Electrostatic Potential (MEP)

In this section we start exploring the regioselectivity of the reaction by looking at the molecular electrostatic potential (MEP) computed at the B3LYP-D4/def2-TZVPP in implicit DMF as solvent. In Figure S5, we plotted the MEP on the unreacted complex consisting of thioformamide anion **1** and **16** compounds in DMF implicit solvent to rationalize their relative orientation prior the reaction. We see that the Ph-N=C=S linkage of **16** is close to the N5 atom of the compound **1** as the space between them (red region, see yellow arrow in Figure S2 and Figure S3 for the atom indices) represents a region of mutual electrostatic attraction. More specifically, the relative orientation of the molecules in the unreacted complex has the following logic: the N6 atom of **16** (phenyl isothiocyanate) is positively charged and is at 3.341 Å from the negative N7 atom of the nitrile group of **1** molecule (*N*-phenyl-cyano-thioformamide anion), whereas its N5 atom is negative and is at a 4.506 Å from the mildly negative C8 of compound **16**. The MEP plot reveals that N5 (yellow arrow) is the site with the highest attraction to a positive test charge. This means that N5 is the site most likely to be blocked via hydrogen bonding with protic solvents, whereby hindering the production of intermediate **1-N** and confirms that is the first atom to initiate the nucleophilic attack in absence of polar protic solvents. Finally, the thioformamide sulfur S3 and the nitrile N7 of compound **1** (see Figure S5) both display negative regions (red lobes) that indicates accumulation of electron density corresponding to lone pairs.

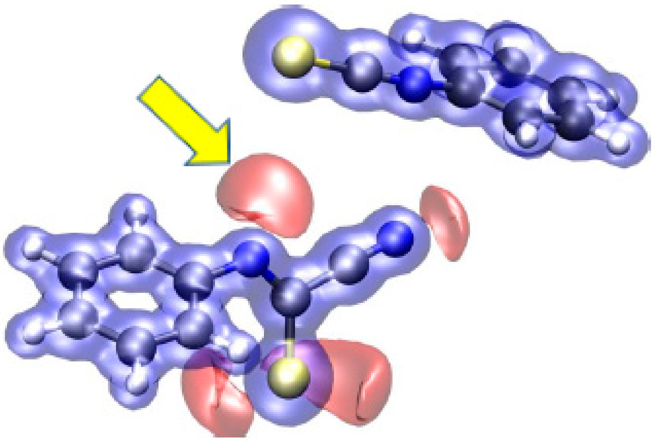

Figure S5. Isosurface representation of the molecular electrostatic potential (MEP) for the unreacted complex of **16** (above molecule) with deprotonated **1** (below) in implicit DMF as solvent. The level of theory was B3LYP-D4/def2-TZVP. The isosurface value is ±0.2 a.u., with the blue and red regions correspond to positive and negative signs, respectively. The yellow, blue, white and grey spheres represent the S, N, H, and C atoms. A prominent negative region (red lobe, see yellow arrow) is observed above the N atom bonded to phenyl ring of the bottom molecule (anion **1**).

S389. Electron Density Isosurface Representation for the Reaction

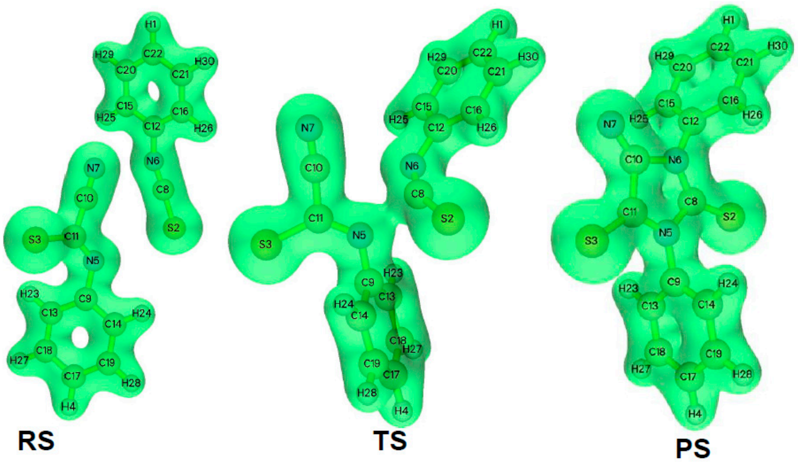

Figure S6. Electron density isosurface representation of the reactant (RS), transition (TS) and product (PS) states for the addition of **1** anion (bottom) to **16** (top) molecule. The level of theory was B3LYP-D4/def2-TZVP and implicit DMF as solvent. The isosurface value is 0.050 a.u.. Each molecular system was rotated for visual clarity.

S390 Kohn-Sham Molecular Orbitals

Here we complete the regioselectivity description by looking at some relevant Frontier Molecular Orbitals (FMO) of the combined unreacted complex. The atoms with the largest magnitude of the MO coefficient will determine the reactivity. In Figure S1, we clearly see that the HOMO and LUMO of the complex are localized mostly on compound **1**, while the LUMO+3 (#81, -0.3184 eV) resides on the isothiocyanate **16** moiety. The HOMO (#77, -5.361081 eV) is dominated mostly by the lone pair on the S3 atom with some minor contribution of N5 whereas LUMO (#78, -1.26738 eV) has a π* character involving the thioformamide plane, with both MOs residing on the **1** molecular subunit. The HOMO, HOMO-1 (#76, -5.4869 eV) and HOMO-2 (#75, -6.1579 eV) have sizable components on the *2p* atomic orbital of N5 atom and have the right orientation towards C8 for the formation of an incipient intermolecular N5—C8 bond. Both LUMO+1 (#79, -1.1992 eV, not shown) and LUMO+3 have extended π* MO's delocalized all over the **16** subunit and are made from the antibonding combination of *p* atomic orbitals perpendicular to the molecular plane. They have the right symmetry for the formation of N5-C8 intermolecular σ bond.

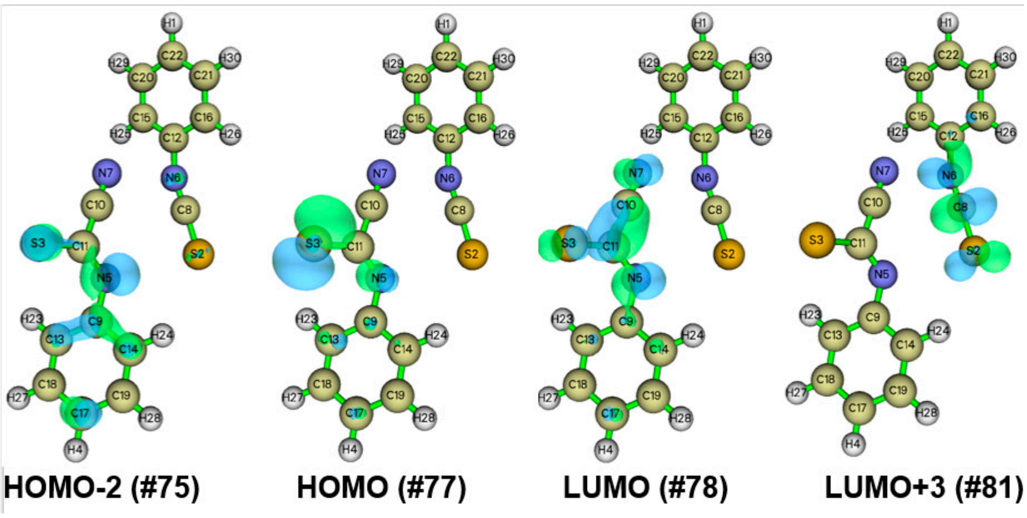

Figure S7. Isosurface representation of the relevant Frontier Molecular Orbitals (FMO) for the unreacted complex made of anion molecule **1** (bottom) with **16** (top) compounds. The level of theory was B3LYP-D4/def2-TZVP and implicit DMF. The isosurface value is ±0.070 a.u., with the green and blue regions corresponding to the positive and negative signs of the MO, respectively. The yellow, blue, white and grey spheres represent the S, N, H and C atoms. The orbital numbering is indicated in parentheses, with MO indices starting at 1.
